# MSI2 promotes translation of multiple IRES-containing oncogenes and virus to induce self-renewal of tumor initiating stem-like cells

**DOI:** 10.1038/s41420-023-01427-9

**Published:** 2023-04-28

**Authors:** Da-Wei Yeh, Xuyao Zhao, Hifzur R. Siddique, Mengmei Zheng, Hye Yeon Choi, Tatsuya Machida, Padmini Narayanan, Yi Kou, Vasu Punj, Stanley M. Tahara, Douglas E. Feldman, Lin Chen, Keigo Machida

**Affiliations:** 1 Department of Molecular Microbiology and Immunology, University of Southern California Keck School of Medicine, Los Angeles, 90033 USA; 2 Viterbi School of Engineering, University of Southern California, Los Angeles, 90089 USA; 3 Department of Medicine, Keck School of Medicine, University of Southern California, Los Angeles, 90033 USA; 4 Southern California Research Center for ALPD and Cirrhosis, Los Angeles, 90033 USA; 5 Present Address: Molecular Cancer Genetics & Translational Research Lab, Section of Genetics, Department of Zoology, Aligarh Muslim University, Aligarh, 202002 India

**Keywords:** Infection, Cancer stem cells

## Abstract

RNA-binding protein Musashi 2 (MSI2) is elevated in several cancers and is linked to poor prognosis. Here, we tested if MSI2 promotes MYC and viral mRNA translation to induce self-renewal via an internal ribosome entry sequence (IRES). We performed RIP-seq using anti-MSI2 antibody in tumor-initiating stem-like cells (TICs). MSI2 binds the internal ribosome entry site (IRES)-containing oncogene mRNAs including *MYC, JUN and VEGFA* as well as HCV IRES to increase their synthesis and promote self-renewal and tumor-initiation at the post-transcriptional level. MSI2 binds a lncRNA to interfere with processing of a miRNA that reduced *MYC* translation in basal conditions. Deregulation of this integrated MSI2-lncRNA-*MYC* regulatory loop drives self-renewal and tumorigenesis through increased IRES-dependent translation of *MYC* mRNA. Overexpression of MSI2 in TICs promoted their self-renewal and tumor-initiation properties. Inhibition of *MSI2*-RNA binding reduced HCV IRES activity, viral replication and liver hyperplasia in humanized mice predisposed by virus infection and alcohol high-cholesterol high-fat diet. Together MSI2, integrating the MYC oncogenic pathway, can be employed as a therapeutic target in the treatment of HCC patients.

**Figure:**
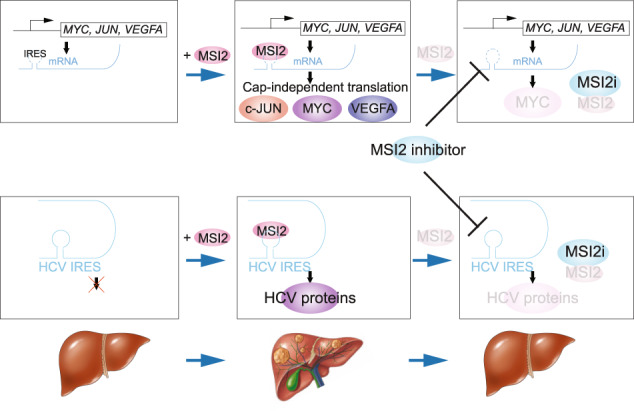
A hypothetical model shows that MSI2 binds and activates cap-independent translation of MYC, c-JUN mRNA and HCV through MSI2-binding to Internal Ribosome Entry Sites (IRES) resulting in upregulated MYC, c-JUN and viral protein synthesis and subsequent liver oncogenesis. Inhibitor of the interaction between *MYC* IRES and MSI2 reduces liver hyperplasia, viral mRNA translation and tumor formation.

A hypothetical model shows that MSI2 binds and activates cap-independent translation of MYC, c-JUN mRNA and HCV through MSI2-binding to Internal Ribosome Entry Sites (IRES) resulting in upregulated MYC, c-JUN and viral protein synthesis and subsequent liver oncogenesis. Inhibitor of the interaction between *MYC* IRES and MSI2 reduces liver hyperplasia, viral mRNA translation and tumor formation.

## Introduction

MSI2 belongs to the Musashi family of RNA binding proteins [1]. These Musashi members participate in post-transcriptional regulation of proliferation and differentiation in progenitor and stem cells via binding to specific mRNAs [2]. Like MSI1, MSI2 binds via tandem ribonucleoprotein-type RNA recognition motifs near its N-terminus to consensus ligand motifs of target mRNAs. The MSI2 expression is associated with aggressive phenotypes in a range of solid tumors, including gliomas, breast, colorectal, lung, and pancreatic cancer and hematologic malignancies as well [2].

Primary liver cancers include cholangiocarcinomas, hepatoblastomas, and hepatocellular carcinomas (HCC), of which the latter accounts for > 90% of primary liver cancers. The estimated incidence of new cases worldwide is about 500,000-1,000,000 each year, causing 600,000 deaths globally [3]. HCC is the fifth most common cancer and the second leading cause of cancer-related mortality. Hepatitis B virus (HBV), Hepatitis C virus (HCV) infection, chronic alcoholic consumption, high fat diet, dietary aflatoxin and tobacco are the major predisposing, and causal factors of HCC [4]. Tumor-initiating stem-like cells (TICs) promote the progression of HCC and chemoresistance. In fact, TICs represent an important component of therapy resistance in the treatment of HCC [5, 6].

Endogenous MYC levels are negatively regulated through turnover of *MYC* mRNA in non-transformed cells [7, 8]. This negative feedback regulation is frequently disrupted during oncogenic transformation, enabling transformed cells to express elevated MYC leading to proliferation [7, 9]. The underlying molecular mechanism(s) in control of MYC expression and its disruption during transformation are poorly understood. Using RIP-seq analysis, *MIR22HG* was identified as an RNA binding target for MSI2. We hypothesized that MSI2 promotes MYC expression by blocking *MIR22HG* processing to mature *miR-22*. Our present data showed that *miR-22* directly suppressed MYC expression and the repression of *miR-22* maturation by MSI2 led to increased MYC expression and contributed to overall tumorigenesis. Concomitantly, MSI2 upregulation also promoted the translation of *MYC* mRNA through an IRES-dependent mechanism. Overall, our study uncovered roles for MSI2 and *miR-22* in both positive and negative feedback loops that controlled MYC expression. Deregulation of this tightly integrated MSI2-*MIR22HG-miR-22*-*MYC* regulatory schema during hepatocellular carcinogenesis involved repressing *miR-22* maturation with increased IRES-dependent translation of *MYC* mRNA and oncogenesis. Together, these events affect the self-renewal and the tumorigenic properties of TICs. We fully expect this study will facilitate the future development of new treatment strategies targeted towards MYC+ TICs that arise in chemoresistant liver cancer patients.

## Results

### RIP-seq analysis identifies MSI2 target RNAs, MYC and *MIR22HG*

**Fig. 1:**
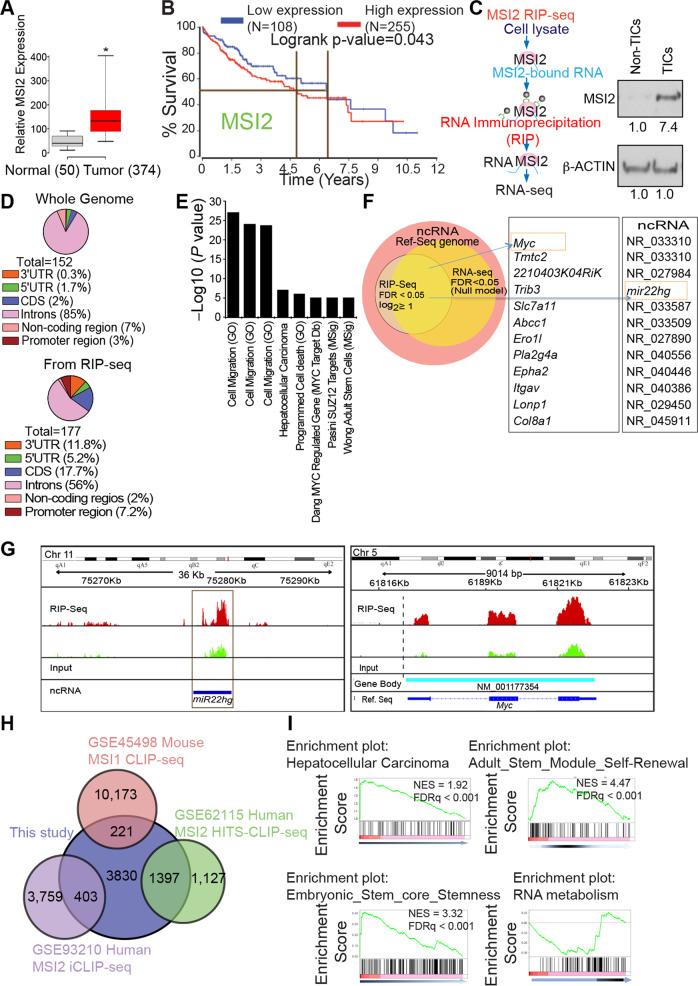
Genome-wide binding of MSI2 to different regions of its target genes. **A** MSI2 is upregulated in HCC patients’ tumors vs. normal samples listed in TCGA datasets. Representative box-whisker plots showing MSI2 expression across 374 tumors and 50 normal samples. Nonoverlapping confidence intervals at 95% indicate that expression levels between two groups differ in the population means (*). Numbers in parentheses represent patients in the corresponding group. **B** MSI2 overexpression is correlated with poor survival of HCC patients in TCGA data analysis. **C** The diagram shows the step-by-step workflow for RNA-Immuno-Precipitation sequencing. Immunoblot uncropped file was included in Supplementary Information. **D** The pie charts show the genome-wide binding pattern of MSI2. Significant binding was observed within introns, coding regions as well as 3′UTRs and 5′UTRs. **E** Pathway enrichment analysis in 177 identified MSI2 binding partners as detected by MSI2-RIP-seq (FDR *p* < 0.05, log2 ratio of enrichment ≥ ±1). A ranked *p*-value was computed for each pathway based on hypergeometric distribution along with Benjamin–Hochberg correction (*p* < 0.05). **F** A Venn diagram shows the identification of a set of 11 significant MSI2 binding targets from genome-wide RIP-seq and RNA-seq approaches; of these 11 genes MYC and *miR-22* are reported to be associated in various cancers. **G** MSI2 binds to *MYC* mRNA and *miR22hg*. Enrichment analysis using Burrows-Wheeler Aligner (BWA) from data for MSI2 shows enriched binding in the exonic regions of *MYC* and *miR22hg*. Representative genomic snap shots of enriched binding within the *MYC* (coding region) and *miR-22* (ncRNA). Vertical dashed lines in *MYC* snapshot indicate transcription start sites (TSS). **H** Venn diagram shows number of MSI2-interacting genes shared among present study and previously published MSI2 RIP-seq results. MSI2-interacting genes shared among the present study and previously published MSI1 or MSI2 iCLIP-seq results (10,394 – 221 = 10,173 genes: MSI1 CLIP-Seq data: GSE54598). MSI2 iCLIP-seq results (4162 – 403 = 3759 genes: MSI2 iCLIP-Seq data. **I** Gene set enrichment analysis (GSEA) shows enrichment for HCC oncogenesis, self-renewal, and stemness as well as RNA metabolism pathways in MSI2 RIP-seq analyses.

TCGA datasets analysis display a higher level of MSI2 expression in HCC tumors (Fig. 1A). The higher expression of MSI2 in HCCs is associated with decreased survival of almost two years (Fig. 1B). To better understand novel interacting partners of MSI2, tumor initiating stem-like cells (TICs) were sorted from mouse CD133^+^ HCC tumor cells for enrichment of MSI2 levels. Accordingly, cell lysates were pre-cleared of ribosomes then subjected to RNA immunoprecipitation (RIP) with antibodies specific to MSI2 followed by deep sequencing of isolated RNA (RIP-seq) (Fig. 1C). After appropriate normalization, as detailed in Materials and Methods, the distribution of genome-wide enrichment of sequencing tags identified by RIP-seq indicated that MSI2 binding was predominantly observed within UTR regions as well as in annotated promoters and regions of both coding and noncoding genes (Fig. 1D).

A pathway analysis of the 177 most highly enriched targets identified by RIP-seq (FDR *p* < 0.05, log_2_ enrichment ratio ≥ log_2_1) indicated that in addition to general liver cancer metastatic pathways, the MYC pathway was significantly represented and presumably regulated by the identified MSI2 binding partners (Fig. 1E). We concluded that RNA binding activity of MSI2 is vital in cell culture. As such, MYC-related, well-known partner molecules associated with various cancers were also identified by this analysis (Fig. 1F**, inset**). From a search of the compendium of RNA-binding proteins in vertebrates two motifs MOD (RNCMPT00140) and SNRNP70K (RNCMPT00143) [10] showed a significant enrichment (*E* < 0.0001) in anti-MSI2, RIP-sequenced regions. From a genome-wide perspective, a significant subset of reference genes and non-coding targets with increased enrichment by RIP-seq analysis (FDR *p* < 0.05) were identified, over and above the input control, as well as a significant upregulation of their transcripts (Fig. 1F). Significantly, *MYC* mRNA was identified as one of the cellular mRNA targets of MSI2 (Fig. 1G) among others (GRP137b and RSPH3a/b) (Supplementary Fig. 1A). Interestingly, miR22hg was the most enriched binding partner among the various non-coding RNAs (Fig. 1G). Furthermore, in order to identify the binding motif of MSI2, we extended the peak of sequence hits ± 25 nts to obtain a consensus motif. These sequences are present in all vertebrates as well as in a database of consensus sequences for RNA-binding proteins (Supplementary Table 2, S5). Therefore, our study focused on discerning the regulatory mechanism of miR22hg and MYC in HCC development by using a liver cancer model.

We carefully examined the MSI2 HITS-CLIP [11] dataset to strengthen the mechanistic insight of our results (Fig. 1H, I). A Venn diagram shows the number of MSI2-interacting genes shared among this study and previously published MSI2 RIP-seq results (Fig. 1H). Gene set enrichment analysis (GSEA) showed a correlation with HCC oncogenesis, self-renewal, stemness as well as RNA metabolism pathways by MSI2 RIP-seq analysis (Fig. 1I). Data from other studies that correlate with the results in this paper were reanalyzed to generalize our RIP-seq data. Supplementary tables include a list of genes that are commonly enriched in analyses of current and previous studies (Supplementary Tables 1, 2, 3). These results indicated that MSI2 binds not only a common RNA sequence motif, but also binds various RNA species differentially, indicating that MSI2 function is affected by cellular context-dependent binding.

### MSI2 binds MYC mRNA and *MIR22HG*

**Fig. 2:**
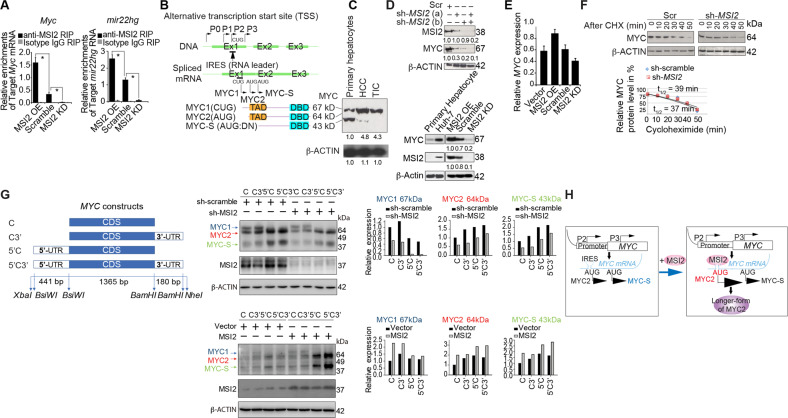
MSI2 promotes MYC protein expression through 5′UTR and 3′UTR-dependent processes. **A** Validation of MSI2-RIP-seq data by RT-qPCR. RIP was performed with anti-MSI2 monoclonal antibody and candidate mRNAs were quantitated using RT-qPCR. Expression analysis showed the enrichment for MSI2-target *MYC* and *MIR22HG* gene products was augmented in MSI2 overexpressing (OE) Huh7 cells and that was reduced in sh-MSI2-silenced cells. **B** Diagram of transcription start sites for MYC and corresponding protein products. (Inset) Western blot results showed an increase in longer MYC protein expression in HCC and TICs whereas primary hepatocytes showed relatively low level of longer MYC isoform. **C** Effect of MSI2 silencing on MYC level. Two different shRNAs against MSI2 were tested as shown. Uncropped film images are shown in separate files. **D** Basal expression of MYC and MSI2 in primary hepatocytes vs. Huh7 cells (Left panel). Expression level of MSI2 and corresponding effects on MYC. Western Blot results showed an increase in MYC protein expression in MSI2 overexpressing cells whereas a decrease in MYC protein level was seen in MSI2-silenced cells. β-ACTIN was used as loading control (Right panel). **E** Overexpression and knockdown of MSI2 exerts minimal effect on *MYC* mRNA level. RT-qPCR was employed for examination of expression levels of *MYC* mRNA. *GAPDH* was used for normalization. **F** MSI2 silencing did not affect protein stability of MYC. Huh7 cells were transduced with scramble shRNA (sh-scr) or shRNA targeting MSI2. Seventy-two hours after transduction, cycloheximide (CHX) was added and cells were harvested at the indicated times. MYC protein levels were detected by immunoblotting (Top panel). Results of a representative experiment (*n* = 3) are plotted as percentage of starting MYC protein level for half-life determination (Bottom panel). **G** MSI2 regulates MYC translation through the 5′- and 3′-untranslated regions of the *MYC* mRNA. Schematic illustration of MYC expression constructs which contain coding sequence only (**C**) and in addition to coding sequence either the 3′UTR (C3′) or the 5′UTR (5′C) or a combination of both (5′C3′), as indicated (Left panel). Huh7 cells stably expressing sh-scramble or sh-MSI2 were transfected with the indicated MYC constructs (Center top panel). Huh7 cells were co-transfected with vector or MSI2 and the indicated MYC cDNA constructs (Center bottom panel). Cell lysates harvested 48 h after transfection were subjected to SDS-PAGE and immunoblotting analysis with MYC and MSI2 antibodies. The densitogram of immunoblots for MYC and MSI2 protein level were quantified by ImageJ. (Right panel). **H** A hypothetical model demonstrates that MSI2 binding stimulates IRES-dependent initiation of MYC translation.

To validate the RIP-Seq data, we performed RT-qPCR of RNA extracts enriched by anti-MSI2 RIP for analysis of MSI2-associated RNAs obtained from MSI2-overexpressing and MSI2-silenced cell lysates (Fig. 2A). The RIP RT-qPCR analysis confirmed the enrichment of MSI2 binding to *MYC* mRNA (Fig. 2A, Left panel) as well as *MIR22HG* (Fig. 2A, Right panel) and was directly proportional to MSI2 expression levels. Thus, these data indicated that both *MYC* mRNA and *MIR22HG* are MSI2-bound RNA targets.

### MSI2 regulates *MYC* at the post-transcriptional level

Transcription initiation of *MYC* occurs from multiple promoters—P0, P1, P2, and P3. Translation initiation of P1 and P2 (P1 UTR = 1205 nt, P2 UTR = 525 nt) transcripts begins at CTG and ATG. The P3 transcript (UTR = 555 nt) initiates translation from ATG; however, this is a minor transcript as the majority of mRNAs initiate from promoter P2 in both normal and transformed cells (Fig. 2B) [12]. The *MYC* IRES is 341 or 353 nucleotides long (exon 1) and is located downstream of promoter P1 such that the IRES is present in P0, P1 and P2 transcripts [13]. Two major MYC proteins MYC1 (67 kDa) and MYC2 (64 kDa) are translated from alternative start codons in the promoter P2 transcript. MYC-S (43 kDa) is expressed from the promoter P3 transcript. Thus, MYC-S (43 kDa) is the shortest MYC isoform translated from mRNA initiated from P3 which does not contain an IRES.

We first analyzed normal hepatocytes, HCC cells and TICs for the expression of different MYC variants. We observed that MYC-S (43 kDa) was highly expressed in primary hepatocytes but present at very low levels in HCC and TICs. By contrast, the longer form of MYC (MYC1) was higher in transformed cells (HCC and TICs) and lower in primary hepatocytes (Fig. 2B, inset). We further demonstrated the relationship between MYC and MSI2 by MSI2 knockdown Huh7 cells. This was done by retroviral delivery of two different MSI2-targeting shRNAs and resulted in decreased protein levels of both MSI2 and MYC (Fig. 2C). Next, we examined if MSI2 regulated MYC expression at the post-transcriptional level. Immunoblot analysis indicated MYC protein expression was increased in MSI2 overexpressing Huh7 cells but was reduced in MSI2 silenced cells (Fig. 2D). However, RT-qPCR analysis showed that either depletion of MSI2 or ectopic MSI2 expression had little effect on *MYC* mRNA level (Fig. 2E). This result was consistent with our model of a regulatory relationship between MSI2 and MYC indicating that MSI2 altered MYC translation but did not affect its transcription.

To elucidate the post-transcriptional effect of MSI2 on MYC protein turnover, we added cycloheximide to block protein synthesis in Huh7 cells stably transduced with either scrambled shRNA or specific shRNA to silence MSI2. Cell lysates were obtained at different time points and probed with anti-MYC antibody (Fig. 2F). While overexpression of MSI2 increased the level of MYC (Fig. 2D), there was no difference in rate of MYC turnover between control and MSI2-silenced cells (Fig. 2F, Left. vs. Right panel). Although overall MYC synthesis was reduced by knockdown of MSI2, MSI2 silencing had no effect on overall MYC stability (Fig. 2F, right panel). These results indicated that MSI2 functioned through translational regulation of MYC synthesis.

To determine how the translational activity of *MYC* mRNA was modulated by MSI2, we generated *MYC* cDNA constructs that had only the *MYC* mRNA coding sequence (CDS) and CDS variants that included CDS with only the 3′UTR, CDS with only the5′UTR or CDS with both the 5′ and 3′UTRs (as shown in Fig. 2G, left panel). MSI2 expression enhanced the protein level of MYC (Fig. 2G, center panel). Ectopic expression of *MYC* constructs that included either the coding sequence with 5′UTR or the coding sequence with 5′UTR and 3′UTR led to robustly elevated levels of MYC. Synthesis of both the second-longest-form of MYC2 (p64) and MYC-S (p42) was stimulated by the 5′UTR although there was no evidence showing the longest-form of MYC (p67) was stimulated by the 5′UTR (Fig. 2G, center and right panels**)**.

This experiment was repeated in HepG2 and Hep3B cells to establish whether this was a more general property of MSI2. Accordingly, translational activity of endogenous *MYC* in HepG2 and Hep3B cells was examined by immunoblotting. (Supplementary Fig. 2I, Left panel). Similar to Huh7 cells, the immunoblot showed the MSI2 effect on MYC protein synthesis was dependent on the presence of the 5′UTR and 3′UTR of *MYC* mRNA. MSI2 promoted translation of the longer isoform of MYC in HepG2, while MSI2 silencing in Hep3B reduced levels of the longer isoform of MYC. (Supplementary Fig. 2I, Middle and Right panels). Many cellular IRES-containing genes were identified and enriched by the currently reported MSI2 RIP-seq analyses. JUN and VEGFA are important onco-drivers identified by our MSI2 ChIP-seq results. The immunoblots and RT-qPCR results showed that MSI2 post-transcriptionally increased JUN and VEGFA expression (Supplementary Fig. 2J, K). Therefore, the impact of MSI2 on IRES region of *JUN* and *VEGFA* mRNA and their translational activities were further examined in dual luciferase assays.

### MSI2 expression stimulates the *MYC* IRES in vitro

**Fig. 3:**
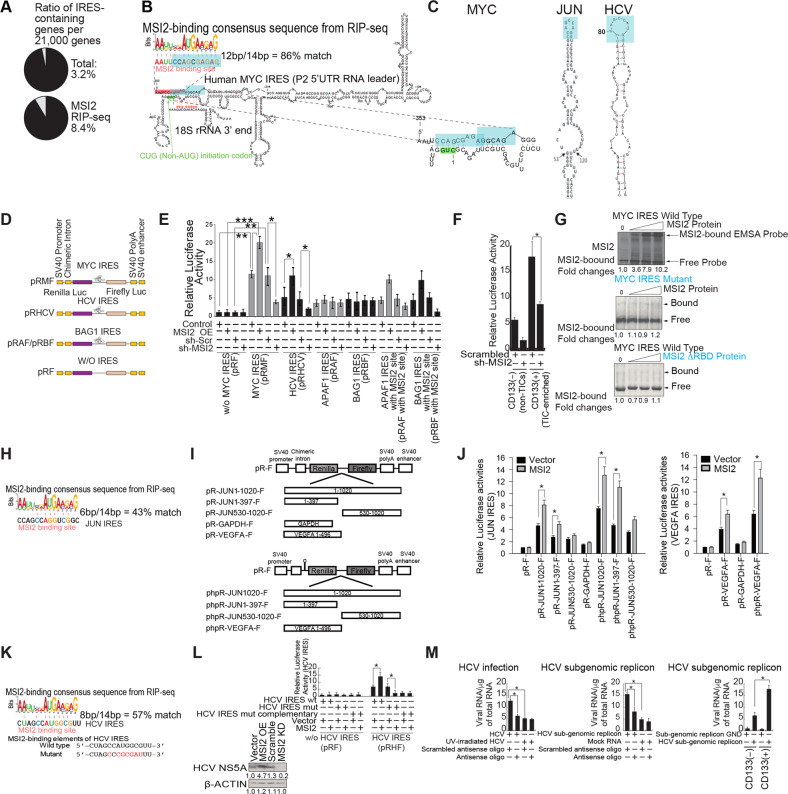
MSI2 expression stimulates the *MYC, JUN and* HCV IRES activity in Huh7 cells. **A** Ratio of IRES-containing genes per 21,000 genes was increased after anti-MSI2 RIP-seq enrichment. **B** Molecular modeling of MSI2 binding motif on the *MYC* IRES. The MSI2 binding consensus sequence has complementarity to the CUG initiation codon (non-AUG) of *MYC* IRES and antagonizes binding to the 3′ end of 18S ribosomal RNA. **C** Comparison of the MSI2 binding sites (in color) of IRES elements of *MYC* and *JUN* mRNAs and HCV RNA. The representative secondary structures are shown. **D** Schematic representation of the reporter constructs pRMF, pRHCV, pRAF/pRBF and pRF. pRF is the parental bicistronic reporter vector lacking an IRES element. pRMF is the construct containing the *MYC*-IRES. Other bicistronic constructs contained IRES elements from HCV (pRHCV), *APAF1* (pRAF) and *BAG1* (pRBF) as indicated. **E** Dependence of IRES-driven luciferase activity on MSI2 binding motif and response to MSI2 expression. Luciferase activity from various IRES reporter plasmids as indicated were assayed under conditions of MSI2 overexpression (OE) or silencing. pRMF reporter activity showed the greatest dependence on MSI2 for maximal relative luminescence activity compared to other IRES reporters. Little or negligible change of luciferase activity was observed for reporter vectors without IRES (pRF) or with *APAF-1* or *BAG-1* IRES elements. Complementation of MSI2 binding motif to *APAF-1* or *BAG-1* IRES restored IRES-driven luciferase activity upon MSI2 expression. **F** CD133(+) TICs have elevated levels of MYC IRES activity, indicating that MSI2^High^ cells support corresponding elevated levels of synthesized MYC protein. **G** EMSA assays were performed with recombinant MSI2 protein or MSI2 protein with deleted RNA-binding domain (ΔRBD) in the presence or absence of wild type or mutant *MYC* IRES RNA probes. **H** The MSI2 binding consensus sequence has homology to *JUN* IRES. **I** Schematic representation of the reporter constructs pR-JUN-F and pR-VEGFA-F. phpR-JUN-F and phpR-VEGFA-F were constructed by inserting the hairpin sequence (−55 Kcal/mol) upstream of Renilla luciferase coding sequence. These hairpin-containing plasmids were made to inhibit translational initiation by ribosome scanning from the 5′ cap structure and to exclude the impact of leaky SV40 promoter activity on mRNA levels. **J** Luciferase activities were determined in the presence or absence of MSI2. Data represent mean ± S.D. from three independent experiments. *P < 0.05. **K** The MSI2 binding consensus sequence has homology to HCV IRES. **L** Reduction of HCV IRES activity with mutations of HCV IRES MSI2-binding sites. Reduction of HCV IRES activity after mutation of HCV IRES MSI2-binding sites. **M** (Left panel) Luciferase assays were performed in the presence or absence of mutations in HCV IRES MSI2-binding sites. Synthetic MSI2-binding site-mimetic oligonucleotides were transduced into HCV infected or sub-genomic replicon Huh7 cells. (Right panel) Repression of HCV replication and sub-genomic replicon by antisense oligos to HCV IRES MSI2-binding sites. RT-qPCR results of HCV RNA level in HCV infected or sub-genomic replicon Huh7 cells. CD133(+) TICs have elevated levels of HCV RNAs, indicating that MSI2^High^ cells supported elevated levels of HCV RNA.

The ratio of IRES-containing genes detected per 21,000 genes was increased after anti-MSI2 RIP-seq enrichment (Fig. 3A). As described above, the expression of the second longest isoform of MYC (64-kDa-MYC: MYC2) in Huh7 cells was positively regulated by MSI2. Accordingly, we observed higher expression of the longest isoform of MYC (67-kDa-MYC: MYC1) in TICs (Fig. 2B). An examination of the consensus MSI2 binding motif showed that it overlapped with the 5′-end of the *MYC* IRES region (Fig. 3B; Supplementary Fig. 2). The secondary structures of the *MYC*-IRES and the MSI2 RNA binding site adjacent to the IRES were examined to identify possible structural motifs important for function. The MSI2-binding motif forms a stem loop structure sequestering the noncanonical CUG (non-AUG) initiation site (Fig. 3B). The 40S ribosome recruits the *MYC* IRES region during initiation leading to the 40S preinitiation complex and subsequent 60S ribosome binding to assemble the 80S initiation complex for mRNA translation (Fig. 3B). The proposed secondary structure suggests a plausible mechanism for the activation of MYC mRNA translation. Namely, MSI2 binding to the single-strand motif could activate IRES-dependent translation of MYC by uncovering the CUG initiation site. To generalize MSI2-mediated cellular IRES activation, 14 different cellular IRES sequences were bioinformatically aligned with the MSI2-binding consensus sequences. Representative MSI2 binding motif are shown in secondary structures of the *MYC*, *JUN* mRNAs and HCV IRES RNA by molecular modeling (Fig. 3C).

We examined if MSI2 regulated MYC expression by binding to the *MYC* mRNA 5′ UTR sequence of the P2 transcript, especially to the predicted site within the IRES element. Various luciferase reporter plasmids were employed expressing luciferase behind the *MYC* (pRMF) IRES for comparison to the cap-dependent translation of *MYC* (without *MYC* IRES) (pRF). The reporter constructs containing IRES elements from *APAF-1* (pRAF), or *BAG-1* (pRBF) mRNAs (examples of cellular IRESs) and HCV (+)-strand RNA (pRHCV) were used as specificity controls as described earlier [13, 14] (Fig. 3D). MSI2-overexpressing or sh-MSI2 stably transduced Huh7 cells were then transfected with IRES-containing reporter plasmids. In cells with MSI2 overexpression, we observed a 110% increase in *MYC* and 60% change in HCV, IRES-mediated translation activity in the pRMF- and pRHCV-transfected cells, respectively. In the presence of sh-MSI2 the luciferase activity was reduced to the basal level for both MYC and HCV reporters. The responsiveness of the HCV IRES to MSI2 level may suggest an alternative mechanism for translational initiation (not the same as for MYC) [15]. *APAF-1* and *BAG-1* IRES elements did not show significant changes in luciferase activity, suggesting that MSI2 was specific for *MYC* IRES but not for other cellular IRES elements. However, addition of the MSI2 binding motif to *APAF-1* and *BAG-1* IRES reporters made them responsive to MSI2 expression (Fig. 3E). CD133(+) TICs have an elevated level of *MYC* IRES activity, indicating that MSI2^High^ cells supported increased levels of MYC protein synthesis through IRES-initiated, cap-independent translation to meet an adverse tumor microenvironment (Fig. 3F).

Next, we deleted the putative MSI2 binding region within the *MYC* IRES by site-directed mutagenesis. The importance of the MSI2 interaction motifs within the IRES was underscored as mutation of this region resulted in sharply attenuated reporter activity in Huh7 cells (Fig. 3G). MSI2 stimulated IRES-dependent translation while MSI2 was not stimulatory for cap-dependent translation in reporter constructs (Supplementary Fig. S2F).

To examine if a bona fide interaction exists between MSI2 protein and *MYC* mRNA, UV cross-linking and electrophoretic mobility shift assay (EMSA) with purified MSI2-His_6_ was performed with both ^32^P-labeled *MYC* IRES or just the MSI2 binding motif in the presence or absence of cold probes, as previously described [16]. The ^32^P-labeled RNA transcripts generated from pSK-ML (*MYC* IRES expression construct) or pSK-GAP-L (GAPDH vector for specificity control) were incubated with purified recombinant MSI2 as indicated, then subjected to EMSA. The EMSA results demonstrated that MSI2-bound RNA probe complexes increased in a MSI2 dose-dependent manner (Fig. 3G, Top panel), but no MSI2-*MYC* IRES RNA binding was observed with *MYC* IRES mutant probe (Fig. 3G, Middle panel) nor with mutant MSI2 RNA-binding protein and wildtype IRES probe (Fig. 3G, Bottom panel), even with increasing amounts of binding protein. For comparison with other RNA binding proteins, hnRNPK and YB1 were also tested for binding activity towards the *MYC* IRES [17] by EMSA but showed no such activity (Supplementary Fig. 3H, I). Other control proteins (i.e., MSI1 and PTB) were tested for IRES binding activity but also showed no such binding activity (Supplementary Fig. 3A-S3G). Several truncated mutant probes were used for EMSA made from different restriction digested DNA templates to further confirm that MSI2 binding motif was located near the 5′-end of the IRES element (Supplementary Fig. 3H). MSI2 recombinant protein bound to *MYC* IRES in a *MYC* IRES sequence dependent manner (Supplementary Fig. 3I). These results were consistent with our model that MSI2 binds the *MYC* IRES to augment MYC translation from the P2 *MYC* mRNA variant. This further supported the specificity of our model for MSI2 binding to the *MYC* IRES. (Fig. 3G, Middle panel).

We further examined whether MSI2 increases translational efficiency of *MYC* mRNA by polysome fractionation analysis. Polysome profiling assays were performed to see if MSI2 specifically augmented *MYC* mRNA translation rather than act as a global activator of mRNA translation. Sucrose density gradient ultracentrifugation was employed, followed by RNA isolation and RT-qPCR analysis of polysome fractions from different sucrose gradients (Supplementary Fig. 3J–L). Polysome profiling showed knockdown of MSI2 decreased but overexpression of MSI2 increased translational activity of *MYC* mRNA. This was evident from MSI2 dependent increase of *MYC* mRNA in the polysomal regions of the sucrose gradients. The experiments indicated MSI2 positively regulates MYC expression at this post-transcriptional level (Supplementary Fig. 3J-S3L).

We observed protein expression but not mRNA levels of *JUN* and *VEGFA* that have MSI2-consensus-binding sites (Supplementary Fig. 2K) changed proportionally with ectopic expression or knockdown of MSI2 (Supplementary Fig. 2H–J). We further examined their IRES activities by luciferase reporter assay with or without ectopic MSI2 expression in HepG2 cells. These results displayed that ectopic MSI2 stimulated *JUN* or *VEGFA* IRES reporter activities (Fig. 3I, J). Several cellular mRNAs or viral RNA containing IRES elements, e.g., *MYC*, *JUN*, *VEGFA* and HCV IRES were shown to respond positively to MSI2 overexpression (Fig. 3H–J).

To further investigate if MSI2 promotes HCV RNA replication via the HCV IRES, HCV IRES reporter assays were performed in the presence or absence of mutations within the presumptive MSI2-binding sites of the HCV IRES. HCV IRES reporter activity was increased by ectopic MSI2 expression, but was compromised by mutations in the MSI2-binding sites (Fig. 3K, L). Antisense MSI2-binding site oligonucleotides (complementary to the MSI2 RNA motif) were synthesized and introduced into HCV infected or sub-genomic replicon transduced Huh7 cells. The antisense oligonucleotide treatments reduced HCV replication and subgenomic replicon levels (Fig. 3M), indicating that MSI2-HCV IRES interaction may enhance HCV replication, but this observation requires further validation. Nonetheless our results support the positive regulation of cellular IRES-containing proto-oncogenes by MSI2 (i.e. *MYC*, *JUN* and *VEGFA* mRNAs and viral (HCV) RNA).

### MSI2 suppresses the level of mature *miR-22* and enhances the activity of *MYC*

*MIR22HG* RNA was identified from our RIP-Seq analysis as a critical binding partner of MSI2 (Fig. 1F; Fig. 2A, Right panel). This noncoding, *MIR22HG* RNA is the host gene for pri-*miR-22* and mature *miR-22*. Previously, it was shown that the RNA binding protein HuR recruits *let-7* to repress MYC expression [18]. Furthermore, it was shown that MSI2 and HuR inhibit the processing of pre-*miR-7* [19]. For these reasons, we next examined whether the physical interaction between MSI2 and *MIR22HG* influenced the level of mature *miR-22*.

**Fig. 4:**
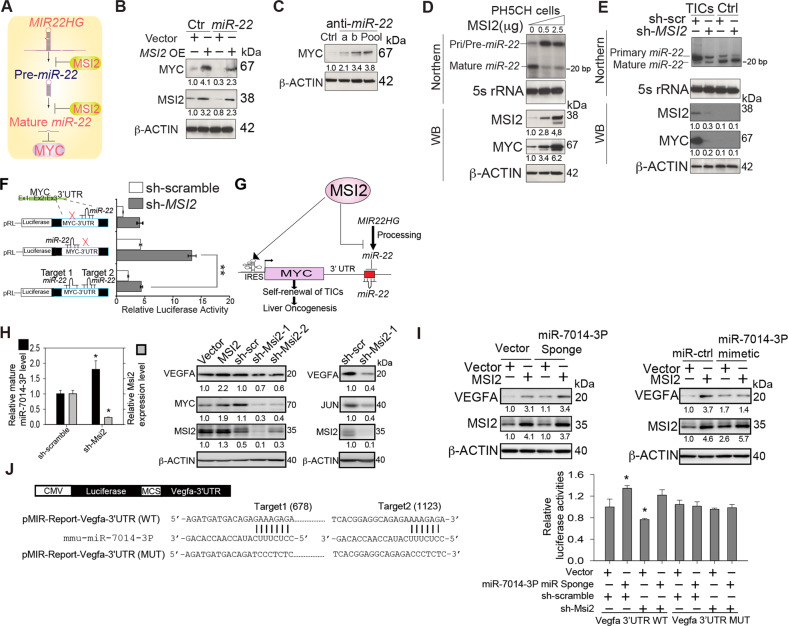
*miR-22* binds the 3′UTR of MYC to inhibit its expression; MSI2 antagonizes *MIR22HG* processing. **A** Hypothetical model for MSI2 regulation of MYC via inhibition of mature *miR-22*. **B** Anti-*miR-22* restored MYC expression. PH5CH cells were transiently transfected with either of two different anti-*miR-22* oligonucleotides or combined anti-*miR-22s* as indicated. Cells were lysed 72 h after transfection and immunoblots were probed with MYC antibodies. **C** MYC levels were reduced by *miR-22* but MSI2 reversed *miR-22* suppression of MYC. Huh7 cells were transiently transfected with *miR-22* mimic oligonucleotides, or with appropriate control *miR* oligonucleotides as indicated. Cells were lysed 72 h after transfection and immunoblots were probed with MYC antibodies. **D** PH5CH cells were transfected with increasing amounts of synthetic *MSI2* expression vector or a vector control. Total RNA was isolated as described in methods for northern blotting and probed with *miR-22* and 5 S RNA probes as indicated. A separate aliquot of cells was lysed and analyzed by western blotting with specific antibodies as indicated. **E** Mouse TICs were transfected with scramble shRNA or shRNA targeting MSI2. Cells were processed and examined by northern and western blotting as above. Northern blot revealed the reduction of mature *miR-22* in HCC cells. **F** *miR-22* repressed *MYC* 3′UTR-driven luciferase activity. Mutation of putative Target 2 *miR-22* binding site abrogated repressive effect of *miR-22*. Activities were expressed as relative luminescence units (RLU) normalized to the activity of co-transfected *Renilla* luciferase. Data represent mean ± S.D. from three independent experiments. *P < 0.05. **G** A hypothetical model shows that MSI2 binding to *MIR22HG* and *MYC* mRNA suppresses *miR-22* maturation and stabilizes mRNA, rendering unimpeded translation of MYC mRNA to promote self-renewal of TICs and liver oncogenesis. Overexpression of MSI2 inhibits *MIR22HG* RNA processing. **H** RT-qPCR analysis of mature *miR-7014-3P* and *Msi2* mRNA expression in Hepa1-6 murine HCC cell line stably transduced with sh-scramble or sh-Msi2 (Left panel). Protein levels for Vegfa, Myc, Jun, Msi2 in Hepa1-6 ectopic expressing Msi2 or stably transduced with sh-Msi2 was analyzed by immunoblotting (Center and Right panels). Data represent mean ± S.D. from three independent experiments. *P < 0.05. **I** Protein level for Vegfa, and Msi2 in Hepa1-6 ectopic expressing either vector or *miR-7014-3P*-sponge (Left panel) or miR-ctrl or *miR-7014-3P* mimic (Right panel) in the presence or absence of MSI2 expression was analyzed by immunoblotting. **J** Schematic representation of *Vegfa* 3′-UTR luciferase reporter plasmids wild-type (WT) and mutant (MUT) with wild-type or mutant of *miR-7014-3P* binding sites, as indicated respectively (Left panel). Hepa1-6 cells stably transduced with sh-scramble or sh-Msi2 were co-transfected with vector or miR*-*7014-3P-sponge, and *Vegfa* 3′-UTR-WT or -MUT luciferase reporter plasmids, as indicated. Relative luciferase activities were measured 24 h post-transfection (right panel). Data represent mean ± S.D. from three independent experiments. *P < 0.05.

The 3′UTR of *MYC* has a binding site for *miR-22*. Given that *MIR22HG* was immunoprecipitated with anti-MSI2, we reasoned that MSI2 might regulate MYC expression via *miR-22*. As noted earlier, mature miRNA is processed from non-coding *MIR22HG* RNA. Transient expression of *miR-22* mimetic oligonucleotides lowered expression of endogenous MYC (Fig. 4B). Conversely, transient expression of anti-*miR-22* enhanced expression of endogenous MYC, confirming that endogenous *miR-22* reduced the level of MYC (Fig. 4C). Finally, expression of *miR-22* not only lowered the expression of endogenous MYC but also negated the effect of endogenous MSI2 on MYC expression (Fig. 4B). From these results MSI2 was permissive for MYC expression by positively regulating its translation through the *MYC* IRES while concomitantly suppressing the amount of mature miR-22.

Northern blotting analysis in HCC cells showed that overexpression of MSI2 significantly reduced the level of mature *miR-22* in both PH5CH cells (Fig. 4D) and mouse TICs (Fig. 4E). Reduction of *miR-22* correlated with increased level of *MIR22HG* RNA as would be expected for inhibition of precursor-processing. A similar reduction of *miR-22* was observed by RT-qPCR detection (Supplementary Fig. 4A, B). We next tested if MSI2 modulated the luciferase activity of the mouse *Myc*-3′UTR reporter. We compared the wildtype *Myc* 3′UTR with two constructs mutated within each of the two *miR-22* target sites (the mouse Target-2 *miR-22*-binding site is conserved in the human 3′ UTR) (Fig. S4C, D**)**. Accordingly, mutation of the Target-2 site resulted in mitigation of *miR-22* activity (Fig. 4F). By contrast, single mutation of the Target-1 site had no effect on luciferase activity and was the same as observed for the wt *MYC* 3′UTR construct. As indicated above, the human *MYC* mRNA has a single *miR-22* target site (homologous to the mouse *Myc* mRNA Target 2 site by relative position). Thus, these data indicated that MSI2 is capable of indirectly augmenting translation of mouse MYC by antagonizing *MIR22HG* processing resulting in decreased level of *miR-22*. Although human *MYC* 3′UTR has only a single *miR-22* site, we strongly believe that it is functional based on the sum of experimental results we have presented thus far. This model is summarized in Fig. 4G.

In addition, our MSI2 RIP-Seq analysis identified *miR-7014-3P* among the MSI2-bound miRs may potentially regulate VEGFA expression. An online database (miRDB.org) predicted putative *miR-7014-3P* binding sites located in the 3′UTR of *VEGFA* mRNA (Supplementary Fig. 4E). We first examined the mature *miR-7014-3P* level in Hepa1-6 murine HCC cells stably transduced with sh-Msi2 by RT-qPCR. The level of mature *miR-7014-3P* increased while *Msi2* was knocked down in Hepa1-16 cells (Fig. 4H, left panel). Protein levels analyzed by immunoblotting showed ectopic expression of MSI2 increased protein levels for VEGFA, and MYC while MSI2 silencing reduced those for VEGFA, MYC, JUN in Hepa1-6 (Fig. 4H, center and right panels). We further tested the impact of *miR-7014-3P* on protein expression of VEGFA by treatment of Hepa1-6 with either *miR-7014-3P* sponge or *miR-7014-3P* mimic. Overexpression of *miR-7014-3P*-sponge in Hepa1-6 increased protein level for VEGFA, and co-expression of MSI2 and *miR-7014-3P*-sponge further enhanced VEGFA expression (Fig. 4I, left panel). In contrast, *miR-7014-3P* mimetic RNA reduced VEGFA while ectopic MSI2 expression restored VEGFA protein levels downregulated by *miR-7014-3P* mimetic (Fig. 4I, right panel). To investigate if VEGFA expression was subjected to *miR-7014-3P* binding to its 3′UTR, we further generated *VEGFA* 3′UTR luciferase reporter plasmids (Fig. 4J, left panel) and performed *VEGFA* 3′UTR reporter assay in Hepa1-6. The experiment revealed that *miR-7014-3P* sponge stabilized reporter expression while shRNA against MSI2 decreased the luciferase activity regulated by *VEGFA* 3′UTR-WT. MSI2 knockdown antagonized this effect of *miR-7014-3P* sponge. In contrast, either *miR-7014-3P* sponge or MSI2 knockdown failed to exert any effect on luciferase activity regulated by *VEGFA* 3′-UTR mutant containing the mutant *miR-7014-3P* binding site (Fig. 4J, right panel). These results revealed that MSI2 through binding to two different RNA targets, affected miR maturation and subsequent translation efficiency and mRNA stability of MYC and other potential targets including VEGFA.

### MSI2 expression increases colony formation and self-renewal of cells

**Fig. 5:**
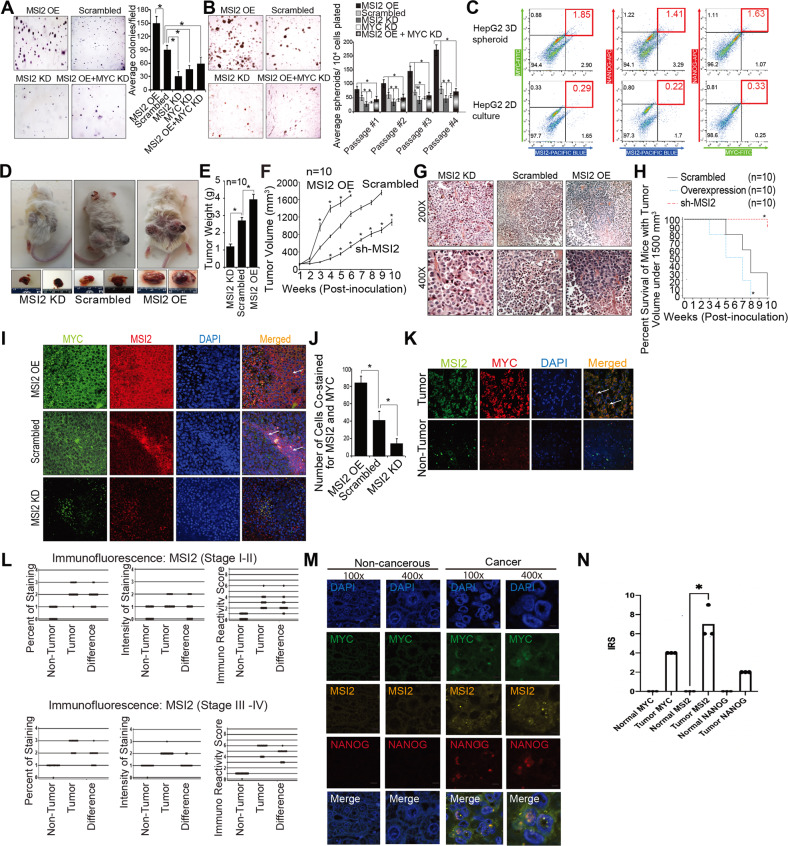
MSI2 upregulation and MYC coexpression promote self-renewal, tumor-initiation abilities of TICs and liver oncogenesis. **A** MSI2 overexpression increased colony formation whereas shRNA silencing of endogenous MSI2 reduced colony formation in Huh7 cells. Combined MSI2 overexpression with MYC knockdown reduced colony formation compared to MSI2 overexpression only. Quantitation of colony numbers for each treatment (Right panel). Data represent mean ± S.D. from three independent experiments. *P < 0.05. **B** In vitro self-renewal was tested by spheroid formation assay; overexpressing MSI2 resulted in larger numbers of spheroids as compared to MSI2-silenced TICs. Spheroid formation data are summarized in the histogram (Right panel). Data represent mean ± S.D. from three independent experiments. *P < 0.05. **C** Flow cytometric analysis for MSI2, MYC and NANOG expression in HepG2 spheroids vs. normal culture cells. Representative dot plots for population analysis of the MSI2^+^, MYC^+^, and NANOG^+^ stemness-enriched cells from three independent experiments. NANOG^high^/MSI2^high^ and NANOG^high^/MYC^high^- pertinent quadrants are labeled in red squares. **D** Mice implanted with Huh7 cells transduced with lentiviral vectors expressing either shRNA for MSI2 knockdown or cDNA for MSI2 overexpression. Representative tumor growth at day 42 post-xenograft implantation is shown. **E** Tumor weights for xenotransplants as per panel (**D**). Total tumor weights for each animal were recorded weekly as indicated. (mean ± S.D., *n* = 10) *p < 0.05. **F** Tumor volume for xenotransplants as per panel (**D**). Tumor volumes were monitored for up to ten weeks. Surviving mice were euthanized on day 70. (mean ± S.D., *n* = 10) *p < 0.05. **G** Xenotransplant tumor histology. H&E staining of representative tumor xenograft tissues is shown. **H** Summary—Kaplan–Meier plot of remaining mice with tumor volumes <1500 mm^3^ as indicated. n=number of animals. *p < 0.05. **I** MSI2 and MYC co-immunostaining of xenograft tumor specimens. Immunofluorescent microscopy demonstrated that increased expression of MSI2 positively correlated with MYC expression in xenograft tissue samples. **J** Scoring of cells co-staining positively for MSI2 and MYC at 20⨯ magnification, *N* = 30 microscopic fields for all groups, *p < 0.05. **K** Immunofluorescent microscopy detection of increased expression and co-localization of MSI2 and MYC in human HCC samples. **L** Immunoreactivity Score (IRS) of MSI2 in non-tumor and tumor regions of clinical HCC specimens. (Top Row) Percent of staining, the intensity of staining, and Immunoreactivity Score (product of the two) for MSI2 protein in Stages I-II (Top Row) and Stages III-IV (Bottom Row). **M** Representative immunofluorescent microscopy detection of MYC, MSI2, and TIC marker NANOG in normal and HCC tissues, indicating that NANOG+ TICs have elevated levels of MSI2 and MYC. **N** Immunoreactivity score (IRS) of MYC, MSI2 and NANOG in normal and HCC tumor tissue, *p < 0.05.

We further examined the in vitro tumorigenic activity of MSI2 in TICs in vitro. Colony growth in soft agar is indicative of the tumorigenic potential of many cell types. We observed that MSI2 overexpressing cells showed increased colony numbers from TICs, whereas MSI2 silenced cells showed a reduction in colony formation. Furthermore, to assess if MSI2 overexpression influenced colony formation through MYC, we silenced MYC in MSI2 overexpressed cells. We observed a significant decrease in colony formation when MYC expression was abrogated (Fig. 5A). These quantitative results are summarized in the histogram (Fig. 5A, Right panel). We extended this result to examine if the in vitro self-renewal capacity of Huh7 cells was altered by MSI2 overexpression in the spheroid formation assay. As shown in Fig. 5B, Left panel), MSI2 overexpression quantitatively increased spheroid formation compared to MSI2 knockdown alone or in the presence of MYC knockdown. Spheroid formation in ultra-low adhesion plates was monitored over 15 days. After serial passages, we observed a similar trend as observed with colony formation, i.e., increased sphere formation as a function of passage and time in culture. Extended culturing led to increased spheroid formation. This time-dependent increase also mimics the in vivo behavior of recurrent HCC (Fig. 5B, Right panel). Thus, these data indicated MSI2 increased the tumor forming capacity of TICs in vitro by increasing overall MYC expression.

To better understand the MSI2 regulation of MYC in TICs vs. HCC, we further examined the expression levels for MSI2, MYC and stemness marker NANOG in HepG2 subjected to spheroid culture vs. normal culture. By using flow cytometry analysis, we checked if expression of MSI2, MYC, and NANOG increased in stemness enriched spheroid cells and if elevated MSI2 positively correlated with MYC expression in HCC spheroid cells (Fig. 5C). In addition to elevated levels of all three markers observed in spheroid cells, FACS analysis indicated that MSI2 potentiates cap-independent MYC translation in spheroid cells, with higher frequency of MSI2^+^/MYC^+^ in the total MSI2^+^ population (39%; 1.85/(1.85 + 2.9)) than in normal cultured cells (15%; 0.29/(0.29 + 1.65)). Furthermore, the ratio of MSI2^+^/MYC^+^ in the total MYC^+^ population (68%; 1.85/(0.88 + 1.85)) was higher than that of MSI2^+^/NANOG^+^ in the total NANOG^+^ population (53%; 1.41/(1.22 + 1.41)) of spheroid cells, demonstrating that upregulated MYC compared to NANOG was more likely subjected to IRES/MSI2 regulation (NANOG^high^/MSI2^high^ and NANOG^high^/MYC^high^-quadrants (labeled as red quadrant populations of Fig. 5C).

### MSI2 expression increases tumorigenesis in vivo

Based on the in vitro data, we next determined the influence of MSI2 on the expression of MYC proteins in xenograft tumor tissues. NSG mice were subcutaneously implanted with Huh7 cells that overexpressed MSI2, that were shRNA-silenced for MSI2, or treated with scrambled shRNA. Mice were monitored for tumor growth for 10 weeks whereupon we observed that tumor volumes were significantly larger in MSI2 overexpressing, Huh7 cell-derived tumors compared to the MSI2-silenced Huh7 mice recipients (Fig. 5D–F). This showed that MSI2 promoted the tumorigenicity of Huh7 cells. Post-mortem examination of tumors by H&E staining showed that tumors were of hepatocyte origin. The histology of MSI2 knockdown tumors displayed more epithelial type cells while histology of MSI2 overexpression tumors displayed an increased nuclear/cytoplasm ratio, which was indicative of malignant tumor types (Fig. 5G). In addition, the expression of MSI2 had a significant negative effect on survival when compared across the three tumor groups (*p* < 0.05). Pairwise comparisons showed that mice in the MSI2 knockdown group lived significantly longer than the control (scramble) group or the MSI2 overexpressing group. However, there was no significant difference in survival between the scrambled group and the MSI2 overexpressing group (*p* = 0.22) (Fig. 5H). Immunohistochemical analysis of tumor tissues showed that modulation of MSI2 in tumor cells had a direct relationship to MYC expression level (Fig. 5I). MSI2 overexpression was observed to significantly increase the level of MYC in tumors whereas MYC expression was reduced when MSI2 expression decreased (Fig. 5I). These data showed that increased MSI2, by modulating MYC expression, increased tumorigenicity in vivo. Furthermore, the observed co-expression of MSI2 and MYC in the same cells was consistent for this mode of tumorigenesis. (Fig. 5I).

### MSI2 and MYC co-expression in xenograft tissue specimen and HCC specimens

Based on the mouse tumorigenesis studies we assessed the clinical relevance of our findings in human HCC clinical samples. These data are presented in Supplementary Table 4. Expression of MYC was significantly higher in tumor tissues than in non-tumor tissues (*p* < 0.001) (Fig. 5J and Supplementary Fig. 5A, B). Additionally, there was a significant difference in immunoreactivity scores (IRS) between tumor tissues vs. non-tumor tissues, from patients with low staged disease (*p* < 0.001) and patients with high staged disease (*p* < 0.001) (Supplementary Fig. 5A, B). The magnitude of the differences in immunoreactivity (IRS) score between tumor vs. non-tumor tissues was largest for patients with the higher staged disease compared to patients with lower staged disease (*p* < 0.01). Nonetheless for all patients, the IRS scores were always higher in the tumor tissues than in the non-tumor tissues.

Immunofluorescent staining of human HCC showed increased expression of MSI2 and MYC with co-localization of both only in cancerous tissues. The non-cancerous tissues had minimal staining for MYC and little staining for MSI2 (Fig. 5K). The analysis of human tumor specimens for MSI2 correlated with MYC expression and MSI2 expression was elevated in late-stage HCC (stage III–IV) (Fig. 5L and Supplementary Fig. 5C). Immune reactivity scores (IRS) were obtained from fluorescently stained images. Quantitative analysis of MSI2 and MYC staining showed that tumor regions of human HCC tissue sections had significantly higher levels of MYC and MSI2 (Fig. 5K and Supplementary Fig. 5C), which was consistent with TCGA data regarding *MYC* and *MSI2* mRNA levels (Fig. 1). The observed percentage of cells co-staining with both MYC and MSI2 was significantly higher than the expected percentage if the marker expression was mutually independent. This result indicated a significant correlation of expression and location of the two markers in tumors. This was true for both low staged tumors and the high staged tumors (see Fig. 5J, L and Supplementary Fig. 5C). HCC patient specimens were stained for MSI2, MYC and NANOG to test if MSI2-mediated MYC translation is TIC-specific or if non-TIC bulk cancers also show a similar tendency. Staining data showed that MSI2 was specific in HCC tissues and colocalized with MYC expression. Some of the MSI2-MYC double positive cells colocalized with NANOG (Fig. 5M, N), indicating that human TICs exhibited the MSI2-MYC axis. The cells of the non-TIC, bulk cancer regions were weakly positive for MSI2 and/or MYC. Therefore, the TIC tumor sections likely had preferential MSI2-dependent increase of MYC translation.

### Postulated 3D structure models of MSI2-RNA interactions and inhibition by small molecule inhibitors

**Fig. 6:**
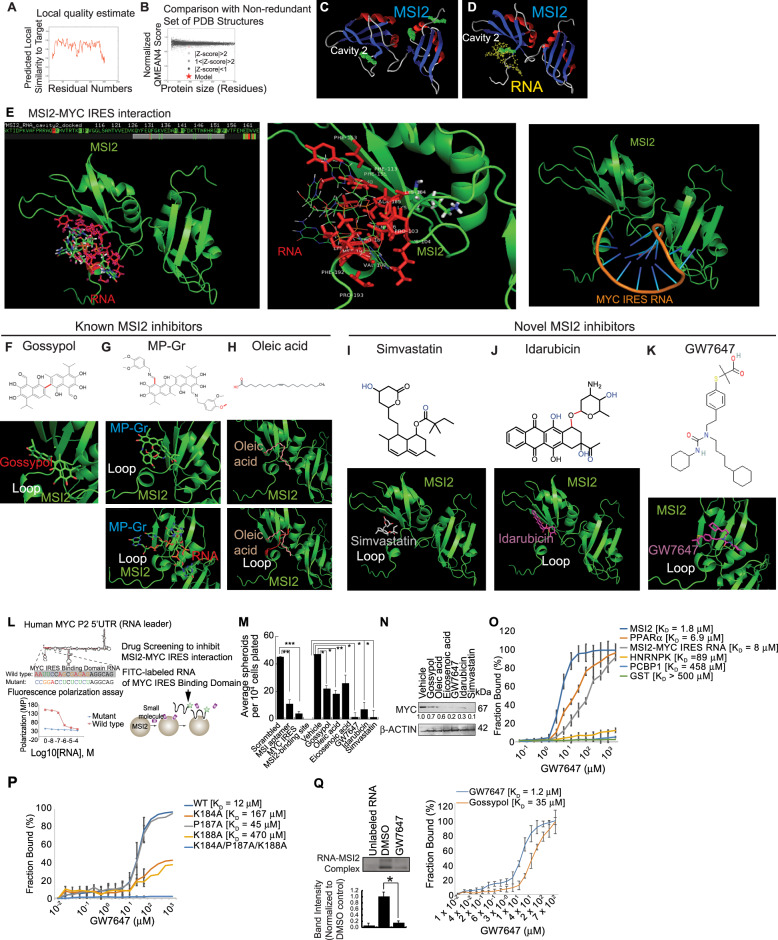
MSI2 small molecule inhibitors identified in 3D structure models of MSI2-RNA interactions reduce MYC expression. **A** Local quality estimate. Human MSI2 structure was simulated by using the template of 2mssA and 2lyvA. **B** Comparison with non-redundant set of PDB structures. The simulated structure has three potential binding sites searched by Van der Waals radii over the molecular surface. **C** For RNA AUUGG, each binding site was simulated 1500 times with redundant radius searching for the lowest energy of interaction. These results were then combined to return the lowest energy from these parameters. **D** Cavity 2 which had the lowest energy was chosen as the RNA docking site. **E** The RNA interacting residues from MSI2 were found based on hydrophilic, hydrophobic and charge-π interactions within 4 Å of the RNA binding site (Top panel). The interacting residues from (**E**, Top) are: Pro103, Lys104, Lys111, Phe113, Met 140, Met 142, Phe153, Phe155, Lys184, Ala185, Gln186, Pro187, Lys188, Val190, Met191, Phe 192 and Pro193. Each compound was initially tested by energy minimized force field analysis and then docked to Cavity 2 individually. The shared interacting residues on MSI2 (for all compounds tested with RNA): Pro103 Lys111 Phe113 Met 142, Phe153, Phe155, Lys184, Ala185, Gln186, Pro187, Lys188 and Met191 (Middle panel). The RNA (5′AAUUCCAGCGAGAG3′) molecular structure was simulated by force field using Monte Carlo analysis for sampling various conformations; the minimized lowest energy conformation was chosen. The RNA was docked to MSI2 protein via template-based modeling. MSI2 is shown in green, whereas the RNA molecule is shown in the cartoon model as blue and brown. The interacting residues from MSI2 to RNA were selected based on polar, charge, hydrophobic or polar-hydrophobic interactions, within the range of 4 Å: Phe24, Asp55, Pro56, Arg62, Phe64, Lys94, Arg100, Ala101, Gln102, Pro103, Lys104, Val106, Thr107, Gln186, Val190, Met191, Phe192, Pro193 (Bottom panel). **F** Gossypol binds to Cavity 2 by torsion of the central C–C bond. **H** Oleic acid binding to Cavity 2. **G** For binding of MP-Gr, the torsion occurs across the methanimine linkage. Also, the methoxyl oxygen forms additional polar contacts. Red is used for labeling the key binding structures besides interactions with surrounding residues. For the rest of the compounds, they all take similar conformations to mimic the RNA which goes around this loop. The RNA ligand surrounding the loop of MSI2 is docked to polar residues outside as this was the best molecular interaction with the lowest energy. **I** Simvastatin: Due to its small structure and relatively inflexible rings, Simvastatin resides right on the RNA binding site. The polar interaction groups with the MSI2 are shown in blue. The residues interacting with Simvastatin from MSI2 are: Gln102, Pro103, Met105, Lys111, Phe113’, Met142, Phe153, Phe155, Lys183, Lys184, Ala185, Gln186, Pro187, Lys188, Met191. **J** Idarubicin: The 3-ring structure occupies the RNA binding groove with the ether linkage of Idarubicin crossing over the loop from MSI2. Polar groups that further improve binding stability are shown in blue. The residues interacting with Idarubicin from MSI2 are: Gln102, Pro103, Lys104, Met105, Phe113, Phe153, Lys183, Lys184, Ala185, Gln186, Met191. **K** GW7647 docking structure with MSI2, which is also another predicted good inhibitor with lower energy. The potential interacting site is also suitable for long alkyl chain fatty acids, e.g. oleic acid (Top panel). Any compound that could bend over this loop and pair with polar groups (the interacting red loop from MSI2 contains Lys184, Ala185, Gln186, Pro187 and Lys188) towards the end or along the side could be potential good inhibitors. The long-carbon-chain acyl compounds can form this type of “C” shape with potential polar interactions, therefore they are expected to be potential inhibitors. If their carbon chains at the C curve possess negatively charged/polar residues (such as in Gossypol), these would be potentially the better candidates to bind to the lysine residues and inhibit MSI2-binding ability, which mimic the phosphates from the RNA molecule (Bottom panel). **L** Screening for selective inhibitors of MSI2-*MYC* IRES interaction. Wild type and mutant *MYC* IRES-binding RNA sequences were labeled with FITC and tested in fluorescence polarization assays for inhibition of MSI2 binding activity. (Right panel) Diagram of drug screening by fluorescence polarization assays. **M** Results of drug inhibition of spheroid formation using Huh7 cells. **N** Effect of drug panel on MYC expression in Huh7 cells. The immunoblot is shown; β-ACTIN included as loading control. Expression of MSI2 promoted self-renewal via increased MYC expression. **O** Microscale thermophoresis (MST) assay of GW7647 was performed with full-length wild-type MSI2 with other RNA-binding proteins and non-RNA-binding protein (GST). **P** Microscale Thermophoresis (MST) assay was performed with full-length wild type MSI2: K184A, P187A and K188A mutants. **Q** Representative images of EMSA with GW7647 in the presence of MSI2 and IRES RNA probe (Left panel). Microscale Thermophoresis (MST) assay was performed with full-length wild type MSI2 in the presence of different concentration of GW7647 (Right panel). A ligand binding curve of the drugs is shown (Right panel). Two-tailed paired *t*-test: *P < 0.05.

To simulate the docking site(s) between MSI2 and mRNA regions, local quality of binding was estimated (Fig. 6A). Human MSI2 structure was simulated by using the template of 2mssA and 2lyvA and compared against non-redundant sets of PDB structures. The simulated structure has three potential RNA binding sites (Fig. 6B). For RNA AUUGG, each MSI2 binding site was simulated 1500 times with redundant radius searching for lowest energy, the results were combined to return the lowest energy from these parameters (Fig. 6C). Cavity 2 which has the lowest energy for RNA binding was chosen (Fig. 6D). *MYC* IRES RNA binds the MSI2 RNA-binding domain, but also other MSI2 domains, indicating *MYC* IRES contacts MSI2 via several domains in addition to the RBD (Cavity 2) (Fig. 6E, top panel). The RNA interacting residues of MSI2 were found based on hydrophilic, hydrophobic and charge-π interactions within 4 Å of the RNA binding site (Fig. 6E, middle panel). RNA 5′AAUUCCAGCGAGAG3′ molecular structure was simulated by force field via Monte Carlo analysis for sampling the putative MSI2 conformation, from this the minimized lowest energy conformation was chosen (Fig. 6E, left panel). The RNA (shown in the cartoon model of blue and brown) is docked to the MSI2 protein (shown in green) via template-based modeling.

The potential interacting residues between MSI2 to RNA were selected based on polar, charge, hydrophobic or polar-hydrophobic interactions, within the range of 4 Å, these were (Fig. 6E, middle): Phe24, Asp55, Pro56, Arg62, Phe64, Lys94, Arg100, ALla101, Gln102, Pro103, Lys104, Val106, Thr107, Gln186, Val190, Met191, Phe192, Pro193. The best interacting residues were identified as: Pro103, Lys104, Lys111, Phe113, Met 140, Met 142, Phe153, Phe155, Lys184, Ala185, Gln186, Pro187, Lys188, Val190, Met191, Phe 192 and Pro193. Therefore, Cavity 2 was chosen as the docking site for the candidate compounds.

Binding of inhibitor compounds to MSI2 was modeled by docking to Cavity 2. First, each inhibitor compound was energy-minimized by force field and then was individually docked to Cavity 2 (Fig. 6E). The shared interacting residues of Cavity 2 (for all these compounds and RNA) were identified as: Pro103 Lys111 Phe113 Met 142, Phe153, Phe155, Lys184, Ala185, Gln186, Pro187, Lys188 and Met191 (Fig. 6E, middle). Gossypol binds to this site structurally by the help of the torsion of the middle C-C bond (Fig. 6F). For MP-Gr, the torsion comes from the methanimine linkage (Fig. 6G). The additional methoxyl oxygen helps the binding by forming another polar contact (Fig. 6G). The other compounds shown all take an assumed similar conformation to mimic the RNA which goes around this loop (Fig. 6G, H). The conformation where RNA surrounds the loop of MSI2 with the polar residues exposed yields the best docking result with the most stability (Fig. 6G). Other compounds were identified as novel MSI2 inhibitors based on modeling studies. Due to its small structure and relatively inflexible rings, Simvastatin resides completely within the RNA binding site. The polar interaction groups with MSI2 are shown in blue (Fig. 6I). The residues predicted to interact with Simvastatin from MSI2 were: Gln102, Pro103, Met105, Lys111, Phe113, Met142, Phe153, Phe155, Lys183, Lys184, Ala185, Gln186, Pro187, Lys188, Met191. For Idarubicin, its 3-ring structure occupies the RNA binding groove of MSI2 with the ether linkage of Idarubicin crossing over the loop from MSI2 (Fig. 6I). Polar groups that further improve binding stability are shown in blue (Fig. 6J). The residues predicted to interact with Idarubicin from MSI2 were: Gln102, Pro103, Lys104, Met105, Phe113, Phe153, Phe155, Lys183, Lys184, Ala185, Gln186, Met191 (Fig. 6J).

We identified GW7647 as a potential inhibitor of MSI2 (Fig. 6K, top panel) since it interacted with the loop from MSI2 that contains many lysines: Lys184, Ala185, Gln186, Pro187 and Lys188 (Fig. 6K, bottom panel).

Structurally, compounds with long alkyl chain acids (e.g., oleic acid) that can form this type of C-shape were generally predicted as potential inhibitors. Potentially if their carbon chain at the C curve could have negative charged/polar residues (such as those in Gossypol) that would structurally complement those polar residues of the MSI2 loop would be predicted to be better inhibitors. These residues could perhaps mimic the phosphates of an RNA ligand molecule and improve binding (Fig. 6K, bottom panel).

### Small molecule inhibitor disruption of MSI2-RNA interaction to reduce MYC protein expression

The importance of MSI2 in HCC in our studies prompted the drug modeling studies as a search for possible inhibitors of its function. We initiated a limited inhibitor screening using FDA approved drugs and compounds. The putative MSI2 antagonists were validated to have activity in cells other assays by demonstrating selectivity towards TICs. Other groups have developed MSI1/MSI2 inhibitors such as Gossypol and MP-Gr [20], sodium oleate, eicosenoic acid and erucic acid [21]. We developed a small molecule inhibitor screen for MSI2 and further validated these drugs in other assays. We employed FITC-tagged *MYC* IRES-MSI2 ligand RNA (wild-type) or mutant IRES RNA as probes for fluorescence polarization binding assays with recombinant MSI2. Inhibition of binding activity was tested using the selected, small molecule library assayed in 384-well plates (Fig. 6L). The mutant IRES ligand served as a negative control since it could not interact with MSI2. The drug candidates from the latter screen were passed to two different screenings to further winnow these inhibitors: 1) Huh7 cell viability screening with primary hepatocytes as a control and 2) an EMSA-based ligand binding assay to select the top 10% candidates which inhibited solution binding of *MYC* IRES to MSI2. Viability screening showed that most of the candidate compounds showed toxicity towards Huh7 cells and primary hepatocytes (*R*^2^ = 0.82) except a subgroup of chemicals that stood out with selective toxicity toward Huh7 cells alone (Fig. 6L, M). The MYC protein levels were reduced in MSI2-inhibitor-treated Huh7 cells (Fig. 6N), indicating that pharmacological MSI2 inhibition resulted in reduced MYC protein levels.

Microscale thermophoresis (MST) assay with GW7647 was performed using full-length, wild type MSI2 and single substitution mutants of MSI2: K184A, P187A and K188A. The MSI2 K184A and K188A mutations partially abrogated inhibitor binding activity but to a much lesser degree than for the P187A mutant. Additional microscale thermophoresis (MST) assays were performed in the presence of pharmacological inhibitor GW7647 and full-length wild type MSI2 or substitution mutants; included in this analysis were other RNA-binding proteins and non-RNA-binding protein (GST) (Fig. 6O, P). EMSA incubations with MSI2 are shown for RNA binding in the presence of GW4746 (Fig. 6Q, left panel). Approximated K_d_ was determined from the concentration of half maximum binding (Fig. 6Q, right). Binding of full length, wild-type MSI2 to increasing concentrations of GW7647 is displayed as a ligand binding curve to estimate its K_d_ (Fig. 6Q, right panel). The results showed the K_d_ of GW7647 was 1.2 μM vs. 35 μM for Gossypol indicating that its tight ligand binding activity was consistent with its observed activity as an effective inhibitor of MSI2 RNA binding.

### Disruption of MSI2-RNA interaction inhibited HCV replication and reduced liver hyperplasia and cancer development

**Fig. 7:**
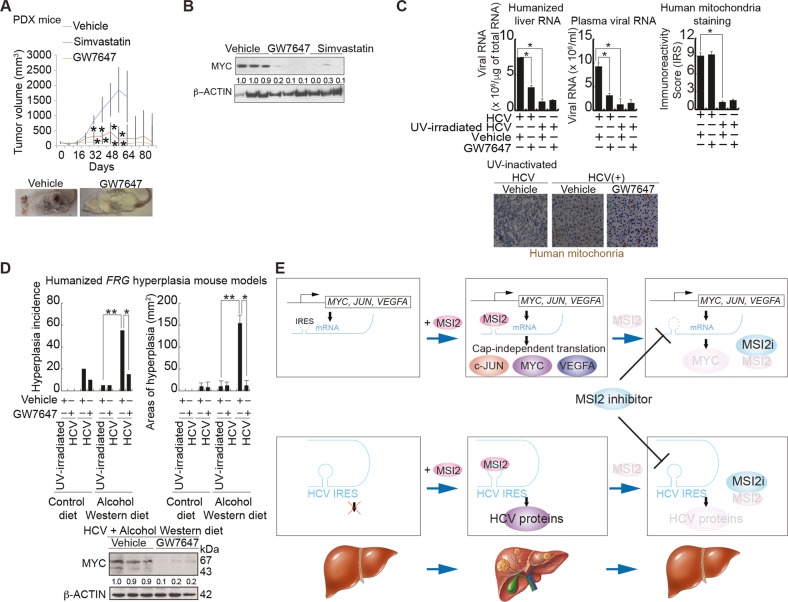
Inhibition of MSI2-RNA interaction inhibited tumor growth in PDX mice, HCV replication and HCV-associated liver hyperplasia in humanized *FRG* mice. **A** MSI2 inhibitors reduced tumor growth in PDX mice. *NSG* mice were used as xenograft recipients for treatment with GW7647 and Simvastatin. Tumor growth was recorded every eight days as tumor volume. Data points correspond to range of tumor volumes for each treatment cohort. (Inset) Representative tumor growth in mouse xenograft recipients from each treatment cohort. **B** Both GW7647 and Simvastatin suppressed MYC protein level of patient HCC xenografts as indicated by immunoblotting of MYC in xenograft tumors from drug treatment groups. **C** Inhibition of MSI2-RNA interaction inhibited HCV replication and HCV-associated liver hyperplasia. *FRG* mice were infected with HCV and fed alcohol western diet for six months. Treatment with GW7647 reduced incidence and size of liver hyperplasia in humanized *FRG* mice (Top panel). Treatment with GW7647 reduced HCV RNA levels, but did not affect the humanization process of livers in *FRG* mice (Bottom panel). **D** Approximately 60% of humanized *FRG* mice developed liver hyperplasia. Treatment with GW7647 inhibited MSI2-RNA interaction and reduced liver hyperplasia in humanized *FRG* mice (Top panel). Treatment with GW7647 reduced MYC protein level in liver of humanized *FRG* mice (Bottom panel). **E** A hypothetical model shows that MSI2 binds and activates MYC mRNA resulting in upregulated MYC translation and subsequent liver oncogenesis. Inhibitor of the interaction between *MYC* IRES and MSI2 reduces liver hyperplasia, viral mRNA translation and tumor formation.

We tested the therapeutic efficacy of the pharmacological inhibitor candidates in the PDX model of HCC. The NSG^TM^ mice were transplanted with a small tissue piece (62.5 mm^3^) from a single patient HCC; this patient had prior etiology of alcoholism and/or HCV infection. These xenografts formed rapidly growing tumors after 16 to 35 days post-implantation and reached an average size of 2,000 mm^3^. Simvastatin treatment (administered i.p. 40 mg/kg, 5 days a week) almost completely suppressed further tumor growth, as compared to control vehicle treatment (Fig. 7A). Furthermore, an injection of GW7647 or Simvastatin reduced sizes of the HCC tissues implanted in NSG mice (PDX mouse model) (Fig. 7A).

We examined whether GW7647 or Simvastatin treatment reduced tumor growth via inhibition of MYC protein expression. Xenograft tumors were analyzed for MYC protein expression levels. Comparison of drug-treated tumors to vehicle-treated tumors indicated that GW7647 and Simvastatin indeed significantly reduced sizes of xenografted tumors (Fig. 7A) and reduced MYC protein levels (Fig. 7B), supporting the expectation that the therapeutic effects of Simvastatin and GW7647 were based on suppression of the MYC pathway mediated by inhibition of the *MYC* mRNA-MSI2 interaction.

We further examined if MSI2 inhibitor GW7647 suppresses HCV replication and HCV-associated liver hyperplasia. Humanized-liver FRG mice were infected with HCV and fed with alcohol western diet for six months. Approximately 60% of humanized FRG mice developed liver hyperplasia (not shown). GW7647 treatment reduced the HCV RNA levels (Fig. 7C, left and middle), but did not affect the humanization process in FRG mouse livers based on mitochondrial staining (Fig. 7C, right). In addition, GW7647 reduced the incidence and sizes of liver hyperplasia in *FRG* mice (Fig. 7D). These results indicated that disruption of MSI2-RNA interaction inhibited HCV replication and reduced liver hyperplasia and cancer development.

## Discussion

The results from our studies demonstrated that MSI2 is one of the key trans-acting factors for IRES-mediated MYC expression. The importance of this study is the observation that MSI2 suppressed the level of mature miR-22 in HCC cells by interrupting its processing from pre-*miR-22* and thus indirectly enhanced overall MYC expression (Fig. 7E). Prior work shows the small molecule inhibitor for MSI2 (Ro 08–2750) also targets MYC expression [22]. By overexpression and knockdown strategies in both mouse and human systems we showed that MSI2 promoted self-renewal and differentiation of TICs. Moreover, overexpression of MSI2 was associated with the aggressive proliferation of tumor xenografts. Additionally, several other factors that regulate TICs self-renewal, such as the transcription factors NANOG and MYC, as well as other microRNAs, have been implicated in oncogenesis [6, 23, 24]. It is possible that MSI2 may act in concert with these factors by regulating TIC self-renewal with the maintenance of an undifferentiated gene expression program in HCC, thus contributing to the poor clinical outcome of such cancers. Other ways to target MSI2 includes ASOs [25] or small molecule inhibitors for MSI2 as above, which were also shown to target MYC [22].

Increased expression of MSI2 is considered a prognostic indicator for poor clinical outcomes in different cancers including HCC [26]. MSI family proteins are well known for their function as a translation inhibitor, but they can also act as a translation activator [27]. MSI2 also maintains the self-renewal program in cancer by directly increasing the translation of MYC and other proteins, without significantly increasing their mRNA levels [11]. However, the mechanistic link between these proteins and MSI2 is largely unknown. Therefore, our findings are very important as we observed that MSI2 directly increased the translation of *MYC* in an IRES-dependent manner without changing its mRNA level in HCC. These findings will contribute towards better understanding the prognostic significance of MSI2 in HCC for efficient therapy.

The proposed MSI2 sites of *JUN* mRNA and HCV RNA are in loops which are single-stranded. We propose that MSI2 binding melts out the extended stems of *JUN* mRNA and HCV RNA, which would be mechanistically different from the *MYC* model. Figure 3B shows 18S rRNA interacting with the dsRNA region. We propose that the end of 18S interacts with the IRES element by base-pairing. To validate this speculative model, further investigation is warranted.

## Conclusions

Taken together, MSI2 enhanced MYC expression by association with the IRES region and suppressed processing of pre-*miR-22* to mature *miR-22* which acted on the *MYC* 3′UTR to impede MYC expression. Targeting MSI2 expression or function may ultimately allow selective suppression of HCC stem cell populations and provide a new therapeutic strategy in the treatment of HCC.

## Materials and methods

- KEY RESOURCES TABLE (Supplementary Table)
- EXPERIMENTAL MODEL AND SUBJECT DETAILS
  - In vivo animal studies
  - Human patients’ tissue samples

- Human patients’ tissue samples: The clinical and pathological characteristics of the patients are summarized in Supplementary Table 4. Thirty formalin-fixed, paraffin-embedded paired primary HCC tissues or non-tumor (adjacent liver) tissues were obtained. 30 patients were diagnosed as having HCC based on the clinicopathologic findings at the University of Southern California Norris Cancer Hospitals. At the time of surgical resection, the tumor area was dissected from the surrounding tissue. Part of the resected tissue was fixed in formalin and embedded in paraffin for histological diagnosis, another part of the resected material was snap frozen in liquid nitrogen for storage at –80 °C for molecular analysis. All tissues were collected with patient informed consent that was granted before surgery by a protocol approved by the Institutional Review Board. These tissues were de-identified prior to use in this study. For immunostaining and immunoblotting analysis of MYC and MSI2 in human HCC, autopsy or surgically excised HCC tissues from 30 patients with or without HCV infection, with or without a history of alcoholism, with or without Obesity/Diabetes/ BMI > 30 were obtained as cryo-preserved samples and paraffin embedded tissue sections according to the approved University Institutional Review Board (IRB) protocol. Many of the specimens were obtained from the Liver Tissue Cell Distribution System at University of Minnesota. Samples were obtained from both genders between the ages of 42–80. Histologically, they all had varying degrees of steatosis (microvesicular and macrovesicular) and inflammation in addition to different stages of HCC. Completely normal liver tissues from 2 patients with accidental death or stroke, but without an apparent liver pathology, were also obtained for immunostaining or immunoblotting. Metastatic Brain tissue was provided by Dr. Zin Htway at HCA Los Robles Hospital and Medical Center. Allotransplants from 15 cryopreserved different mouse metastatic HCC cell lines were also studied for drug susceptibility.
- Cells and cell lines.
- Plasmids, lentivirus and retrovirus vectors, and production of lentivirus & retroviruses
- Isolation of mouse TICs using MACS
- RNA-Immunoprecipitation (RIP): 2 × 10^7^ TICs overexpressing Flag-MSI2 and stably transduced shRNA against MSI2 were subjected to RNA immunoprecipitation (RIP) using the MBL RIP kit (MBL International, MA). In brief, cells were washed with cold PBS and lysed with provided RIP lysis buffer. Anti-Flag M2 Ab (Sigma-Aldrich), anti-rabbit Ab, or anti-MSI2 Ab (EMD Millipore), pre-incubated with magnetic beads (5 μg), were used to immunoprecipitate Flag–MSI2–RNA complexes. Immunoprecipitated complexes were washed and treated with proteinase K. Finally, RNA was extracted with the reagents provided in the kit.
- Bioinformatics analysis of RNA sequencing
- Bioinformatics analysis of binding motif from MSI2 RIP-seq
- Reverse transcription and quantitative PCR (RT-qPCR)
- SDS-PAGE and immunoblot analysis
- Site-directed mutagenesis
- Luciferase reporter assays
- UV cross-linking and EMSA
- Sucrose density gradient centrifugation and RT-qPCR analysis
- MicroRNA isolation, cDNA synthesis, and RT-qPCR

- Northern blots for *miR22hg*

- Spheroid Formation
- Serial Spheroid Formation
- In silico 3D structure prediction
- Fluorescence Polarization assay
- Histology & Immunohistochemistry
- Immunofluorescence Staining
- Statistical Analysis
- QUANTIFICATION AND STATISTICAL ANALYSIS
- DATA AND CODE AVAILABILITY

## Supplementary information

Cover Image

Cover Image

Supplementary Table 4

Supplementary Information

Uncropped films from each Figure

Supplementary Table 1.

Supplementary Table 2.

Supplementary Table 3
