# Supplementary figures and images for: MSI2 promotes translation of multiple IRES-containing oncogenes and virus to induce self-renewal of tumor initiating stem-like cells
Source: Cell Death Discov. 2023 Apr 28;9:141. doi: 10.1038/s41420-023-01427-9 (PMC10147607; doi:10.1038/s41420-023-01427-9)

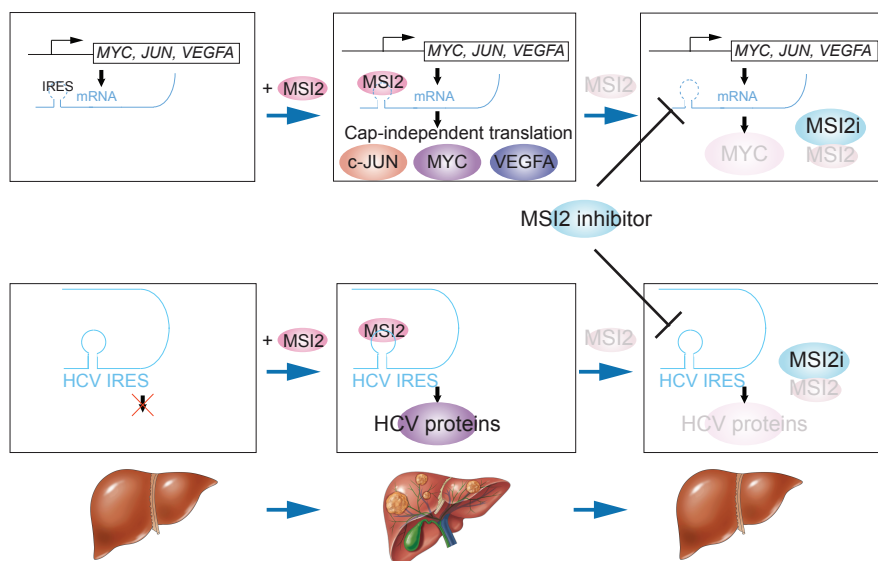

Supplement: Supplementary file 1 — Cover Image [file 41420_2023_1427_MOESM1_ESM.pdf]

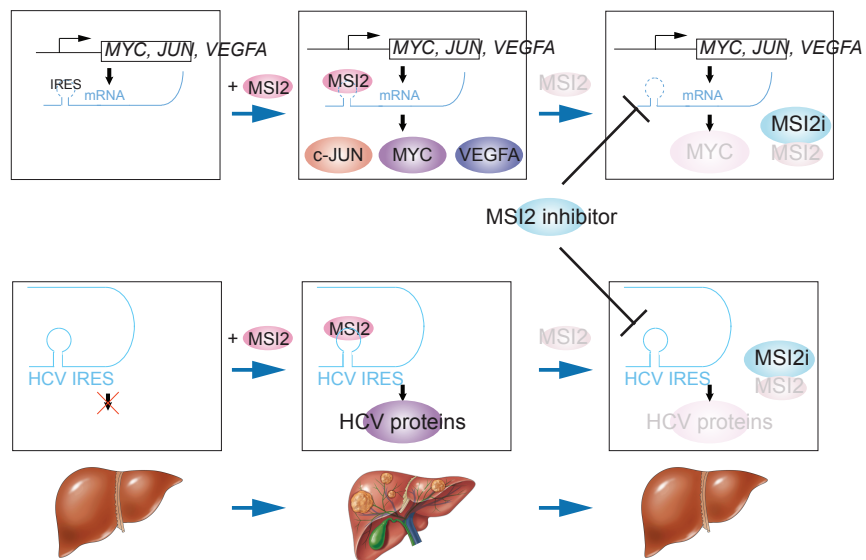

Supplement: Supplementary file 2 — Cover Image [file 41420_2023_1427_MOESM2_ESM.pdf]

**Extended Data Fig. 6. Uncropped films from each Figure.**
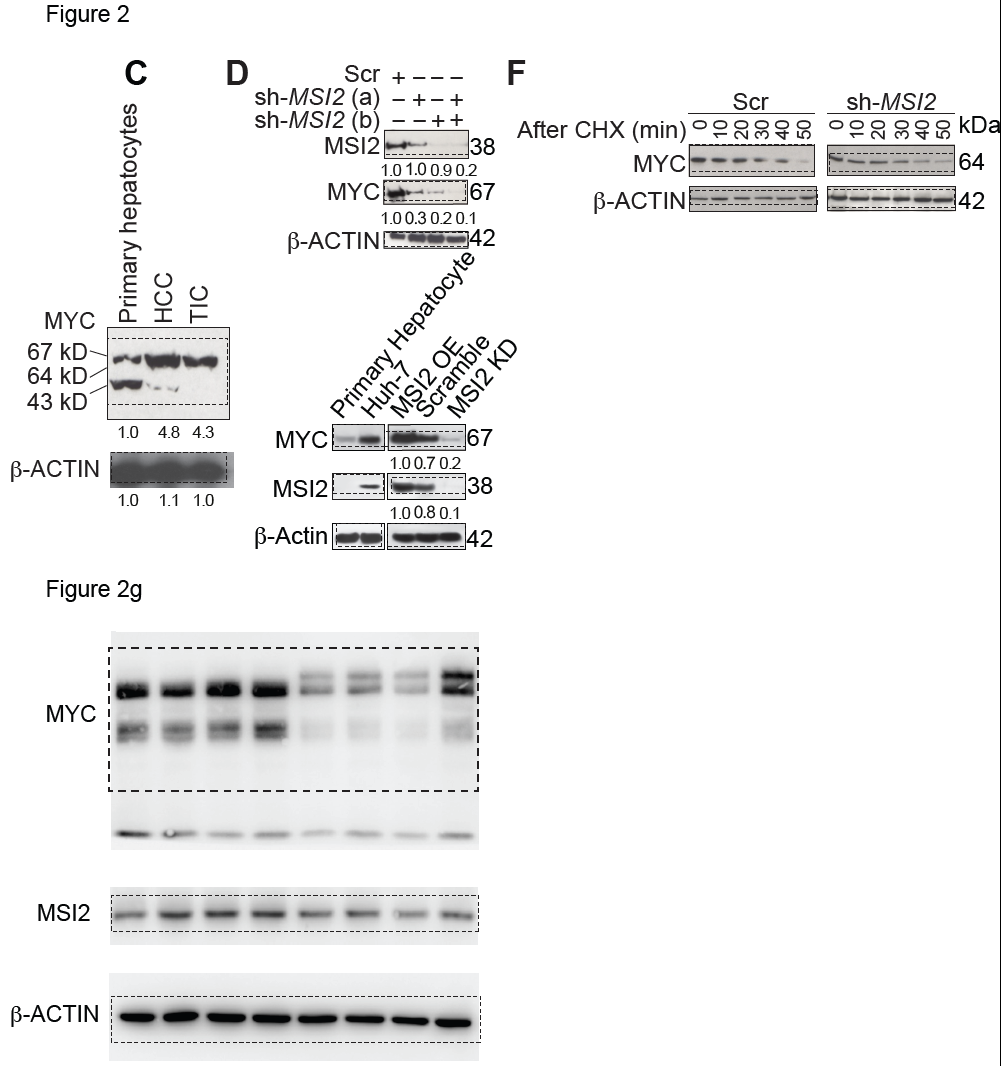


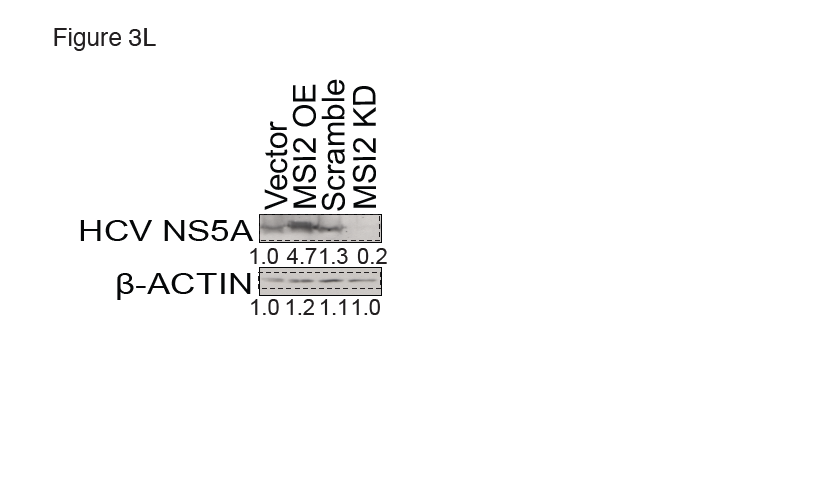


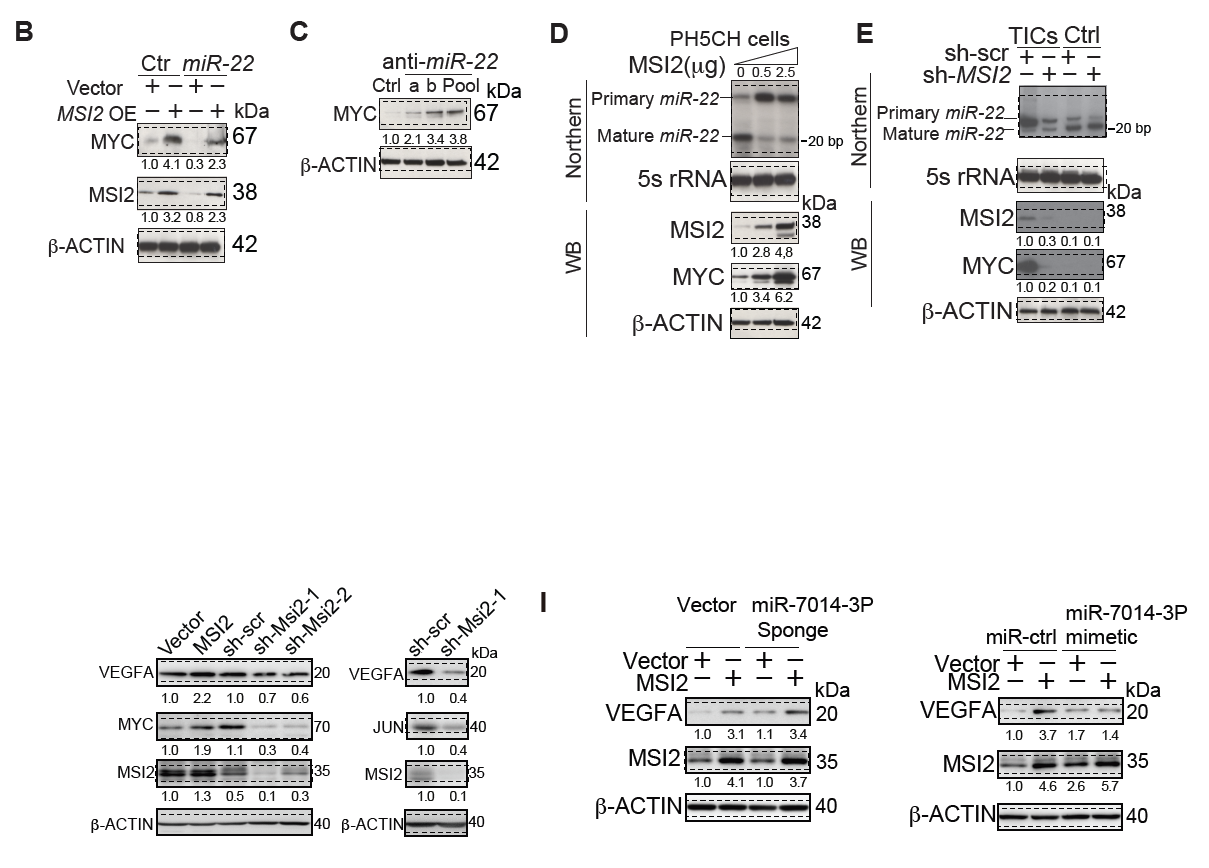


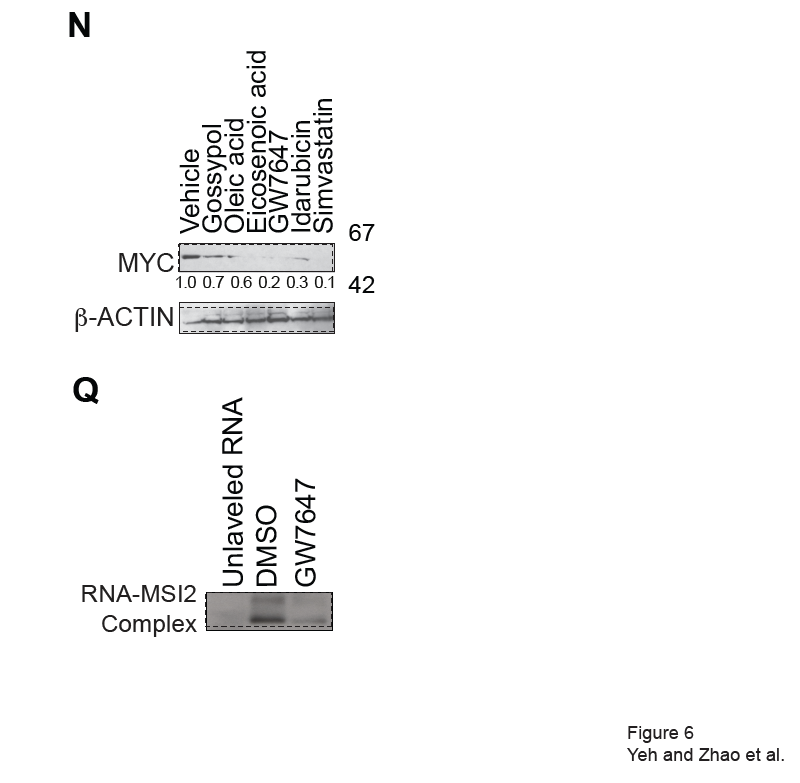

Supplement: Supplementary file 5 — Uncropped films from each Figure [file 41420_2023_1427_MOESM5_ESM.docx]
