# Supplementary material for: MSI2 promotes translation of multiple IRES-containing oncogenes and virus to induce self-renewal of tumor initiating stem-like cells
Source: Cell Death Discov. 2023 Apr 28;9:141. doi: 10.1038/s41420-023-01427-9 (PMC10147607; doi:10.1038/s41420-023-01427-9)
Supplement: Supplementary file 3 — Supplementary Table 4 [file 41420_2023_1427_MOESM3_ESM.pdf]

#chrom	start	end	gene	NA	strand	paek name	TSS2pCenter
chr1	107417716	107560253	Pign	0	-	MACS_peak_588	-7685
chr1	107560437	107651708	2310035C	0	+	MACS_peak_588|MACS_peak_588	7869|7869
chr1	108616986	108656319	Kdsr	0	-	MACS_peak_590|MACS_peak_592	-6501|12113
chr1	108667364	108693302	Vps4b	0	-	MACS_peak_592|MACS_peak_593	-24870|-4989
chr1	109425579	109445848	Serpinb10-	0	+	MACS_peak_594|MACS_peak_594|MACS_	13442|13442|9289|9289|6785|6785|6241|6241|5861|5861|5123|5123|4177|4177|3612|3612
chr1	109407999	109422176	Serpinb2	0	+	MACS_peak_594|MACS_peak_594|MACS_	-4138|-4051|-8291|-8204|-10795|-10708|-11339|-11252|-11719|-11632|-12457|-12370|-13403|-13316|-13968|-13881
chr1	120355245	120355842	2900060B	0	-	MACS_peak_608	5308
chr1	121804477	121809745	Tmem177	0	-	MACS_peak_616	-2171
chr1	129670740	129701414	Ccnt2	0	+	MACS_peak_635	-17501
chr1	130140757	130175954	Ubxn4	0	+	MACS_peak_639	-180
chr1	130484775	130488876	Cxcr4	0	-	MACS_peak_647	-2756
chr1	133049783	133070048	Lgtn	0	+	MACS_peak_652|MACS_peak_652|MACS_	-27395|-27395|-27996|-27996
chr1	133181827	133423938	Srgap2	0	-	MACS_peak_656	-1906
chr1	133424565	133436449	Fam72a	0	+	MACS_peak_656	2533
chr1	133724588	133745438	Slc41a1	0	+	MACS_peak_659|MACS_peak_660|MACS_	-94|-312|-2227|-2753|-11162|-12915|-14048|-14978
chr1	133763853	133769462	Rab7l1	0	+	MACS_peak_663|MACS_peak_664|MACS_	28103|26350|25217|24287
chr1	134010123	134036262	Cdk18	0	-	MACS_peak_670|MACS_peak_672	-25378|-19688
chr1	134212714	134230065	Nuak2	0	+	MACS_peak_676|MACS_peak_677|MACS_	13531|13061|-14496
chr1	134195202	134203934	Klhdc8a	0	+	MACS_peak_676|MACS_peak_677	-3981|-4451
chr1	134402806	134405216	Tmem81	0	+	MACS_peak_685	12355
chr1	134373943	134402242	Rbbp5	0	+	MACS_peak_685	-16508
chr1	134886421	134921925	Mdm4	0	-	MACS_peak_686	-23982
chr1	135500447	135506857	Snrpe	0	-	MACS_peak_687|MACS_peak_688	16737|27163
chr1	135552455	135557462	Zbed6	0	-	MACS_peak_688	-23442
chr1	135516447	135557957	Zc3h11a	0	-	MACS_peak_688	-23937
chr1	136302566	136308315	Cyb5r1	0	+	MACS_peak_691|MACS_peak_692|MACS_	-373|-643|-1060|-2074|-3651|-4625|-5326
chr1	136312043	136328918	Adipor1	0	+	MACS_peak_691|MACS_peak_692|MACS_	9104|8834|8417|7403|5826|4852|4151
chr1	136282516	136287560	4933406M	0	+	MACS_peak_691|MACS_peak_692|MACS_	-20423|-20693|-21110|-22124|-23701|-24675|-25376
chr1	136543257	136645994	Syt2	0	+	MACS_peak_718|MACS_peak_721	18007|14173
chr1	136676299	136700175	9530009M	0	-	MACS_peak_722	-414
chr1	137278891	137327068	Ipo9	0	-	MACS_peak_725	22747
chr1	137331156	137481932	Nav1	0	-	MACS_peak_726	-662
chr1	13775765	13777010	Gm5523	0	-	MACS_peak_32	-28658
chr1	137767971	137815601	Pkp1	0	-	MACS_peak_733|MACS_peak_734	-65|125
chr1	141351349	141390665	Aspm	0	+	MACS_peak_738	11918
chr1	141318959	141349584	Zbtb41	0	+	MACS_peak_738	-20472
chr1	151676751	151808414	Pla2g4a	0	-	MACS_peak_773|MACS_peak_774	-28578|-47
chr1	151947253	151955142	Ptgs2	0	+	MACS_peak_775|MACS_peak_776|MACS_	-5|-908|-1195|-1942|-2564|-3594|-3871|-4269|-4925
chr1	151923790	151946896	7530420F	0	-	MACS_peak_775|MACS_peak_776|MACS_	362|1265|1552|2299|2921|3951|4228|4626|5282
chr1	153214828	153275565	1200016B	0	-	MACS_peak_787|MACS_peak_788|MACS_	-25401|-17310|24663
chr1	153275777	153305312	1190005F	0	+	MACS_peak_787|MACS_peak_788|MACS_	25613|17522|-24451
chr1	153602503	153669458	Edem3	0	+	MACS_peak_799|MACS_peak_800|MACS_	-15516|-20528|-22097|-27068
chr1	155587482	155592598	Rgs16	0	+	MACS_peak_843	-1694
chr1	155596555	155611350	Rnasel	0	+	MACS_peak_843	7379
chr1	157405249	157434924	Acbd6	0	+	MACS_peak_866|MACS_peak_866|MACS_	-9517|-9517|-9517
chr1	157625284	157660029	Qsox1	0	-	MACS_peak_873|MACS_peak_873|MACS_	-21850|-21850|-20659|-20659|-18303|-18303
chr1	158488917	158579750	Abl2	0	+	MACS_peak_884|MACS_peak_884	-137|-137
chr1	158769153	158791209	Angptl1	0	+	MACS_peak_896	10818
chr1	159029278	159038776	1700057K	0	-	MACS_peak_897|MACS_peak_898	27041|28442
chr1	162133051	162142908	Cacybp	0	-	MACS_peak_905|MACS_peak_910|MACS_	7979|11191|13803
chr1	162125390	162131317	Mrps14	0	+	MACS_peak_905|MACS_peak_910	-25497|-28709
chr1	162947886	162964390	Zbtb37	0	-	MACS_peak_915	-14199
chr1	162965296	162968668	Gas5	0	+	MACS_peak_915	15105
chr1	162968222	162968287	Snord47	0	+	MACS_peak_915	18031
chr1	163001022	163016472	Cenpl	0	+	MACS_peak_916|MACS_peak_916	-24741|-24741
chr1	162970743	163000763	Dars2	0	-	MACS_peak_916	25000
chr1	163018508	163061610	Klhl20	0	-	MACS_peak_917	-24701
chr1	163746244	163806792	AI848100	0	-	MACS_peak_926|MACS_peak_927	-22880|-19777
chr1	164147753	164155681	Dnm3os	0	+	MACS_peak_929	-781
chr1	164153498	164153608	Mir214	0	+	MACS_peak_929	4964
chr1	164147944	164148053	Mir199a-2	0	+	MACS_peak_929	-590
chr1	164500958	164529209	Vamp4	0	+	MACS_peak_930|MACS_peak_931	22526|-26458
chr1	164463802	164478476	Mettl13	0	-	MACS_peak_930	-44
chr1	164577379	164588350	7420461P	0	-	MACS_peak_933|MACS_peak_934|MACS_	14550|17496|18493|23079|24197
chr1	164601915	164670687	Bat2l2	0	-	MACS_peak_948	-24235
chr1	164846682	164867356	Fmo6	0	-	MACS_peak_962|MACS_peak_963	21247|29729
chr1	164883930	164914622	Fmo3	0	-	MACS_peak_962|MACS_peak_963|MACS_	-26019|-17537|-16631|-8021
chr1	165860194	165885257	Scyl3	0	+	MACS_peak_972	-6414
chr1	166205728	166217976	4930455F	0	+	MACS_peak_973|MACS_peak_975|MACS_	11239|-16003|-16436|-26904
chr1	166179185	166195499	Slc19a2	0	+	MACS_peak_973	-15304
chr1	166219930	166237615	Blzf1	0	-	MACS_peak_975|MACS_peak_975|MACS_	-15884|-15884|-15884|-15451|-15451|-15451|-4983|-4983|-4983
chr1	166237805	166367425	Nme7	0	+	MACS_peak_975|MACS_peak_975|MACS_	16074|16074|15641|15641|5173|5173
chr1	167225896	167251982	Gpr161	0	+	MACS_peak_981|MACS_peak_982|MACS_	13517|-10634|-21526
chr1	167391338	167411345	Brp44	0	+	MACS_peak_999	859
chr1	167259633	167390594	Dcaf6	0	-	MACS_peak_999	-115
chr1	167415313	167506904	Adcy10	0	+	MACS_peak_999	24834
chr1	167522311	167564672	Mpzl1	0	-	MACS_peak_1002|MACS_peak_1002	-28825|-28825
chr1	168323741	168339959	Pogk	0	-	MACS_peak_1007|MACS_peak_1007|MA	-6299|-6299|-1358|-1358
chr1	168309297	168323749	Tada1	0	+	MACS_peak_1007|MACS_peak_1008	-24363|-29304
chr1	171879667	171899336	Hsd17b7	0	-	MACS_peak_1018|MACS_peak_1019	17856|28848
chr1	171902437	172019075	Ddr2	0	-	MACS_peak_1023	21759
chr1	172634587	172797902	Atf6	0	-	MACS_peak_1030	-26991
chr1	173147933	173152645	Tomm40l	0	-	MACS_peak_1034	28125
chr1	173159702	173164480	Fcer1g	0	-	MACS_peak_1034|MACS_peak_1035|MA	16290|25463|25982
chr1	173155184	173156510	Apoa2	0	+	MACS_peak_1034	-25586
chr1	173200458	173207026	B4galt3	0	+	MACS_peak_1034|MACS_peak_1035|MA	19688|10515|9996|-3714|-6115|-10400|-10702
chr1	173164990	173177243	Ndufs2	0	-	MACS_peak_1034|MACS_peak_1035|MA	3527|12700|13219|26929|29330
chr1	173180552	173190053	Adamts4	0	+	MACS_peak_1034|MACS_peak_1035|MA	-218|-9391|-9910|-23620|-26021
chr1	173207122	173211317	Ppox	0	-	MACS_peak_1035|MACS_peak_1036|MA	-21374|-20855|-7145|-4744|-459|-157
chr1	173212085	173218092	Usp21	0	-	MACS_peak_1035|MACS_peak_1036|MA	-28149|-27630|-13920|-11519|-7234|-6932
chr1	173218694	173225113	Ufc1	0	-	MACS_peak_1039|MACS_peak_1040|MA	-20941|-18540|-14255|-13953
chr1	173367691	173394724	F11r	0	+	MACS_peak_1050	-24300
chr1	173934501	173957399	Vangl2	0	-	MACS_peak_1055	-24293
chr1	174078145	174126524	Dcaf8	0	+	MACS_peak_1059	-27552
chr1	174526770	174532682	Fcrl6	0	-	MACS_peak_1068	-20281
chr1	174511507	174520699	Slamf8	0	-	MACS_peak_1068	-8298
chr1	174486068	174493847	Vsig8	0	+	MACS_peak_1068|MACS_peak_1068	-26333|-22365
chr1	17591981	17621019	Pi15	0	+	MACS_peak_40|MACS_peak_42|MACS_pe	-95|-17951|-27051
chr1	178702455	178702554	Mir350	0	-	MACS_peak_1079|MACS_peak_1080|MA	-22382|-16352|-11001|2245|7916|9907|16083
chr1	178663783	178737255	Cep170	0	-	MACS_peak_1083|MACS_peak_1084|MA	-26785|-24794|-18618
chr1	178744942	178950563	Sdccag8	0	+	MACS_peak_1085	26305
chr1	179374791	179380897	Zfp238	0	+	MACS_peak_1092|MACS_peak_1092|MA	-2709|-1549|-3604|-2444|-4504|-3344|-5358|-4198
chr1	182157134	182186431	Psen2	0	-	MACS_peak_1099|MACS_peak_1099|MA	-29013|-18628|-21188|-10803
chr1	182260606	182353790	Itpkb	0	+	MACS_peak_1101	-3426
chr1	182257099	182260680	Gm5069	0	-	MACS_peak_1101	3352
chr1	182656173	182684334	Acbd3	0	+	MACS_peak_1103	-12508
chr1	182865169	182868532	Lefty1	0	+	MACS_peak_1104	-2971
chr1	182872648	182905235	Tmem63a	0	+	MACS_peak_1104	4508
chr1	183023553	183074288	Nvl	0	-	MACS_peak_1115	-14591
chr1	183081061	183099125	Cnih4	0	+	MACS_peak_1115	21364
chr1	184339297	184392567	Trp53bp2	0	+	MACS_peak_1118	-19831
chr1	185858339	185899515	Dusp10	0	+	MACS_peak_1119	-54
chr1	185856910	185857877	1700056E	0	-	MACS_peak_1119	516
chr1	186551025	186556372	Hlx	0	-	MACS_peak_1127	-32
chr1	187028046	187110624	Rab3gap2	0	+	MACS_peak_1129	-73
chr1	187186973	187252234	Eprs	0	+	MACS_peak_1130|MACS_peak_1131|MA	-54|-4477|-6523|-7872|-9891|-15745|-16545|-18791|-21902|-23993
chr1	187278726	187292640	Slc30a10	0	+	MACS_peak_1149	26679
chr1	191552146	191700572	Ptpn14	0	+	MACS_peak_1150	-209
chr1	192596757	192735649	Rps6kc1	0	-	MACS_peak_1157	-23065
chr1	193130675	193141997	Nenf	0	-	MACS_peak_1161|MACS_peak_1162	7900|20773
chr1	193149843	193176804	Tmem206	0	+	MACS_peak_1161|MACS_peak_1162	-54|-12927

chr1	193730659	193737126	Slc30a1	0 +	MACS_peak_1167	-4564
chr1	193717951	193731406	1700034H	0 -	MACS_peak_1167	3817
chr1	194336709	194597413	Hhat	0 -	MACS_peak_1172	-65
chr1	196868093	196918442	Cd46	0 -	MACS_peak_1175|MACS_peak_1176|MA	4406|5288|6082|25431
chr1	196929981	196957764	Cr1l	0 -	MACS_peak_1178	-13891
chr1	23298539	23298623	Mir30c-2	0 +	MACS_peak_55	-11406
chr1	24017596	24107180	Fam135a	0 -	MACS_peak_57	-14878
chr1	33818597	33871272	Zfp451	0 -	MACS_peak_72	-27942
chr1	33802328	33814595	Bag2	0 -	MACS_peak_72	28735
chr1	34068669	34365497	Dst	0 +	MACS_peak_73|MACS_peak_73|MACS_pe	5878|5878|5534|5534
chr1	36364577	36380874	Arid5a	0 +	MACS_peak_128|MACS_peak_128|MACS_	-8524|-8524|-8524|-8524|-9541|-9541|-9541|-9541|-12068|-12068|-12068|-12068
chr1	36392574	36426026	4632411B	0 -	MACS_peak_131	-25864
chr1	36480030	36502084	Lman2l	0 -	MACS_peak_133|MACS_peak_135	-20346|27193
chr1	36528441	36565621	Cnnm4	0 +	MACS_peak_135	-836
chr1	36568720	36585082	Cnnm3	0 +	MACS_peak_137|MACS_peak_137|MACS_	4252|4252|-8293|-8293|-9391|-9391
chr1	36587378	36592574	Ankrd23	0 -	MACS_peak_137|MACS_peak_138|MACS_	-28106|-15561|-14463|13971|17556|18003|20283
chr1	36595017	36604046	Ankrd39	0 -	MACS_peak_138|MACS_peak_139|MACS_	-27033|-25935|2499|6084|6531|8811
chr1	36605486	36615226	Sema4c	0 -	MACS_peak_140|MACS_peak_142|MACS_	-8681|-5096|-4649|-2369
chr1	37426065	37461284	Inpp4a	0 +	MACS_peak_155	11304
chr1	37487016	37495969	Unc50	0 +	MACS_peak_156|MACS_peak_157|MACS_	-1294|-2457|-8649
chr1	37473929	37486948	6330578E1	0 -	MACS_peak_156|MACS_peak_157|MACS_	1362|2525|8717
chr1	40323430	40373022	Il1r1	0 +	MACS_peak_166	-233
chr1	40497472	40522259	Il1rl1	0 +	MACS_peak_175|MACS_peak_175|MACS_	-11143|-12122|-21913|-22892
chr1	40522396	40548423	Il18r1	0 +	MACS_peak_175|MACS_peak_175|MACS_	13781|13850|14235|3011|3080|3465|-29844
chr1	40572206	40606142	Il18rap	0 +	MACS_peak_191|MACS_peak_192	19512|16463
chr1	40738556	40825730	Slc9a2	0 +	MACS_peak_196	-169
chr1	40828884	40847502	Mfsd9	0 -	MACS_peak_203|MACS_peak_204|MACS_	-28678|-27072|-25029|-24388|-17693
chr1	43104113	43155467	Tgfbrap1	0 -	MACS_peak_210|MACS_peak_212	-22926|29783
chr1	43155554	43172791	AI597479	0 +	MACS_peak_210|MACS_peak_212	23013|-29696
chr1	44163390	44175618	Kdelc1	0 -	MACS_peak_219|MACS_peak_220|MACS_	-11772|-10957|-7933|-4298|-3975|-1633|-202
chr1	44143461	44159233	1700029F	0 -	MACS_peak_219|MACS_peak_220|MACS_	4613|5428|8452|12087|12410|14752|16183
chr1	44176812	44201616	Bivm	0 +	MACS_peak_219|MACS_peak_220|MACS_	12966|12151|9127|5492|5169|2827|1396
chr1	44204588	44238105	Ercc5	0 +	MACS_peak_227	29172
chr1	45431175	45560127	Col5a2	0 -	MACS_peak_237	-275
chr1	55109613	55129546	Coq10b	0 +	MACS_peak_262|MACS_peak_262	-8587|-8587
chr1	55144991	55148161	Hspe1	0 +	MACS_peak_262	26791
chr1	55134677	55144776	Hspd1	0 -	MACS_peak_262	-26576
chr1	58086812	58163254	Aox1	0 +	MACS_peak_266	-27551
chr1	58169979	58257296	Aox3	0 +	MACS_peak_270	15209
chr1	58504476	58519120	Nif3l1	0 +	MACS_peak_271	-17836
chr1	58487836	58502330	Ppil3	0 -	MACS_peak_271|MACS_peak_271	19982|19982
chr1	58770129	58790071	Cflar	0 +	MACS_peak_275|MACS_peak_275|MACS_	-19019|-19019|-26962|-26962
chr1	60237442	60391549	Nbeal1	0 +	MACS_peak_293	-14519
chr1	64579377	64651122	Creb1	0 +	MACS_peak_330|MACS_peak_330|MACS_	-18113|-18113|-18113
chr1	65205189	65233053	Idh1	0 -	MACS_peak_341|MACS_peak_341	10224|17561
chr1	65233258	65325270	Pikfyve	0 +	MACS_peak_341	-10019
chr1	67047091	67085408	Lancl1	0 -	MACS_peak_353|MACS_peak_353|MACS_	-28853|-28891|-28891
chr1	72205807	72258881	Mreg	0 -	MACS_peak_404	-20194
chr1	72305748	72330888	Pecr	0 -	MACS_peak_405	-16847
chr1	72330946	72349569	Tmem169	0 +	MACS_peak_405	16905
chr1	74331607	74400266	Pnkd	0 +	MACS_peak_407|MACS_peak_407|MACS_	-6210|-6210|-10287|-10287
chr1	74326414	74331312	Aamp	0 -	MACS_peak_407|MACS_peak_407|MACS_	6505|6505|10582|10582
chr1	74334821	74350910	Tmbim1	0 -	MACS_peak_407|MACS_peak_408	-13093|-9016
chr1	74325173	74326163	Gpbar1	0 +	MACS_peak_407|MACS_peak_408	-12644|-16721
chr1	74552633	74577416	Rqcd1	0 +	MACS_peak_410	-110
chr1	74838592	74850747	Wnt10a	0 +	MACS_peak_413	9381
chr1	74818465	74831893	Wnt6	0 +	MACS_peak_413	-10746
chr1	75131788	75138942	1810031K	0 -	MACS_peak_419|MACS_peak_420|MACS_	-4992|-2920|-1415|-833|2001
chr1	75013919	75121800	Nhej1	0 -	MACS_peak_419|MACS_peak_420|MACS_	12150|14222|15727|16309|19143
chr1	75139359	75144483	Fam134a	0 +	MACS_peak_419|MACS_peak_420|MACS_	5409|3337|1832|1250|-1584
chr1	75122115	75130464	Slc23a3	0 -	MACS_peak_419|MACS_peak_420|MACS_	3486|5558|7063|7645|10479
chr1	75165219	75168199	Zfand2b	0 +	MACS_peak_420|MACS_peak_420|MACS_	29197|29197|29286|27692|27692|27781|27110|27110|27199|24276|24276|24365|-23750|-23750|-23661|-26544|-26544|-26455|-29908|-
chr1	75177439	75188497	Atg9a	0 -	MACS_peak_430|MACS_peak_431|MACS_	472|3266|6630
chr1	75211548	75215828	Tuba4a	0 -	MACS_peak_430|MACS_peak_431|MACS_	-26859|-24065|-20701|17272
chr1	75207403	75212181	Stk16	0 +	MACS_peak_430|MACS_peak_431|MACS_	18434|15640|12276|-25697
chr1	75168214	75176857	Abcb6	0 -	MACS_peak_430|MACS_peak_431|MACS_	12112|14906|18270
chr1	75188708	75195960	Ankzf1	0 +	MACS_peak_430|MACS_peak_431|MACS_	-261|-3055|-6419
chr1	75194811	75207353	Glb1l	0 -	MACS_peak_430|MACS_peak_431|MACS_	-18384|-15590|-12226|25747
chr1	75232997	75242265	Dnajb2	0 +	MACS_peak_433|MACS_peak_433|MACS_	-103|-103|176|-103|-103|-16384|-16384|-16105|-16384|-16384|-19342|-19342|-19063|-19342|-19342|-21702|-21702|-21423|-21702|-2170
chr1	75243615	75260783	Ptprn	0 -	MACS_peak_433|MACS_peak_436|MACS_	-27683|-11402|-8444|-6084
chr1	75268776	75274955	Resp18	0 -	MACS_peak_436|MACS_peak_439|MACS_	-25574|-22616|-20256
chr1	75305139	75314212	Dnpep	0 -	MACS_peak_441|MACS_peak_441	-3239|-2619
chr1	75432517	75439751	Gmppa	0 +	MACS_peak_444	-206
chr1	75447084	75470915	Accn4	0 +	MACS_peak_444	14361
chr1	77363759	77511663	Epha4	0 -	MACS_peak_454	-87
chr1	78421410	78485421	Farsb	0 -	MACS_peak_455|MACS_peak_456	-20844|-16286
chr1	78654399	78664175	Utp14b	0 +	MACS_peak_457|MACS_peak_457|MACS_	-159|456|-5390|-4775|-10713|-10098|-23976|-23361|-29885
chr1	78654399	78704317	Acsl3	0 +	MACS_peak_457|MACS_peak_457|MACS_	-159|-159|-159|-5390|-5390|-5390|-10713|-10713|-10713|-23976|-23976|-23976
chr1	79772592	79778020	Mrpl44	0 +	MACS_peak_471|MACS_peak_474	-20849|-26928
chr1	82335623	82441941	Rhbdd1	0 +	MACS_peak_480|MACS_peak_480|MACS_	-3745|-26215|-29287
chr1	87791084	87805273	Itm2c	0 +	MACS_peak_506	-8566
chr1	88051389	88149066	Armc9	0 +	MACS_peak_511|MACS_peak_511	-9944|-9979
chr1	89223603	89347385	Gigyf2	0 +	MACS_peak_520|MACS_peak_520|MACS_	-28130|-28161|-29474|-29505
chr1	89282858	89291304	Kcnj13	0 -	MACS_peak_522|MACS_peak_523	-20669|22177
chr1	89373403	89406937	Ngef	0 -	MACS_peak_525	-20700
chr1	89366838	89372033	3110079O	0 +	MACS_peak_525	-19399
chr1	89652645	89688997	Atg16l1	0 +	MACS_peak_527	23443
chr1	89999831	90115572	Ugt1a6b	0 +	MACS_peak_528	14977
chr1	89991575	90116577	Ugt1a7c	0 +	MACS_peak_528	6721
chr1	89967353	90116577	Ugt1a9	0 +	MACS_peak_528	-17501
chr1	92100577	92112863	Cxcr7	0 +	MACS_peak_549	-26
chr1	92950020	93013899	Lrrfip1	0 +	MACS_peak_552|MACS_peak_552	-145|-145
chr1	93829331	94044970	Hdac4	0 -	MACS_peak_568	3
chr1	95375569	95406307	02-Sep	0 +	MACS_peak_570|MACS_peak_570|MACS_	-29731|-29731|-29663|-29731
chr1	95302516	95375385	Hdlbp	0 -	MACS_peak_570	29915
chr1	95408680	95518551	Farp2	0 +	MACS_peak_570	3380
chr1	95517327	95532304	Stk25	0 -	MACS_peak_571|MACS_peak_572	-10016|-8650
chr1	95689152	95698511	Dtymk	0 -	MACS_peak_575|MACS_peak_575|MACS_	-17117|-17117|24500|24500
chr1	95700541	95718677	Ing5	0 +	MACS_peak_575|MACS_peak_576	19147|-22470
chr1	95601967	95651415	Thap4	0 -	MACS_peak_575	29979
chr1	95651609	95686106	Atg4b	0 +	MACS_peak_575	-29785
chr1	95721816	95748751	D2hgdh	0 +	MACS_peak_576	-1195
chr1	9658915	9690290	Mybl1	0 -	MACS_peak_21	21343
chr1	9713277	9738463	Vcpip1	0 -	MACS_peak_21	-26830
chr10	110735734	110738792	Bbs10	0 +	MACS_peak_1821|MACS_peak_1822	21387|16500
chr10	114515698	114622078	Tph2	0 -	MACS_peak_1832	24589
chr10	114726917	114752647	Rab21	0 -	MACS_peak_1833|MACS_peak_1836	-28126|-360
chr10	114822014	114869827	Zfc3h1	0 +	MACS_peak_1844|MACS_peak_1846	641|-15872
chr10	114807021	114821491	Thap2	0 -	MACS_peak_1844|MACS_peak_1846	-118|16395
chr10	114777795	114799318	Tmem19	0 -	MACS_peak_1844	22055
chr10	117125960	117147772	Mdm2	0 -	MACS_peak_1856	-8500
chr10	118296404	118305959	Dyrk2	0 -	MACS_peak_1857	-90
chr10	120802145	120834301	Gns	0 +	MACS_peak_1863|MACS_peak_1866	-173|-26338
chr10	121024435	121063372	Xpot	0 +	MACS_peak_1869|MACS_peak_1870|MA	-23990|-14729|-13007|-12740|-9674|-9186
chr10	120983514	121023850	Tbk1	0 -	MACS_peak_1869|MACS_peak_1870|MA	15532|24793|26515|26782|29848
chr10	121078756	121130970	D930020B	0 +	MACS_peak_1871|MACS_peak_1872|MA	28391|28124|25058|24570
chr10	121518315	121534158	Tmem5	0 -	MACS_peak_1875	-2462
chr10	121515167	121516935	Gm9079	0 -	MACS_peak_1875	14761
chr10	121556752	121567614	Gm4489	0 +	MACS_peak_1875	25056
chr10	126324304	126338427	Xrcc6bp1	0 -	MACS_peak_1886|MACS_peak_1886	-9692|-9692
chr10	126619577	126626879	D10Ertd61	0 -	MACS_peak_1905|MACS_peak_1905|MA	-3702|-3933|19093|18862
chr10	126602280	126609386	B4galnt1	0 +	MACS_peak_1905|MACS_peak_1905	-20897|-20291

chr10	126627433	126632765	Dtx3	0	-	MACS_peak_1905|MACS_peak_1908	-9588|13207
chr10	126634123	126648678	Pip4k2c	0	-	MACS_peak_1905|MACS_peak_1908	-25501|-2706
chr10	126609483	126617701	Slc26a10	0	-	MACS_peak_1905|MACS_peak_1908	5476|28271
chr10	126733277	126748842	Mars	0	-	MACS_peak_1912|MACS_peak_1912|MA	-14232|-14232|-11779|-11779|-11291|-11291|-8934|-8934|-7502|-7502|-6248|-6248|-5991|-5991|-1722|-1722|-1521|-1521
chr10	126727848	126733344	Ddit3	0	+	MACS_peak_1912|MACS_peak_1913|MA	-6762|-9215|-9703|-12060|-13492|-14746|-15003|-19272|-19473
chr10	126719011	126725827	Mbd6	0	-	MACS_peak_1912|MACS_peak_1913|MA	8783|11236|11724|14081|15513|16767|17024|21293|21494
chr10	126760782	126766999	Arhgap9	0	+	MACS_peak_1912|MACS_peak_1913|MA	26172|23719|23231|20874|19442|18188|17931|13662|13461
chr10	126975213	127058204	Lrp1	0	-	MACS_peak_2006	-13143
chr10	127520362	127527444	Atp5b	0	+	MACS_peak_2011	-29391
chr10	127529838	127566359	Baz2a	0	+	MACS_peak_2011|MACS_peak_2012|MA	-19915|-21773|-23310|-29595
chr10	127522341	127522450	Mir677	0	+	MACS_peak_2011|MACS_peak_2012	-27412|-29270
chr10	127759514	127764243	Cnpy2	0	+	MACS_peak_2021	9253
chr10	127774887	127799535	Cs	0	+	MACS_peak_2021	24626
chr10	127733195	127735140	Il23a	0	-	MACS_peak_2021	15121
chr10	127740390	127758403	Pan2	0	+	MACS_peak_2021	-9871
chr10	127832989	127838280	Slc39a5	0	-	MACS_peak_2022|MACS_peak_2022|MA	13985|14743|14692|27419|28177|28126
chr10	127848671	127878496	Rnf41	0	+	MACS_peak_2022|MACS_peak_2022|MA	-3594|-3594|-17028|-17028|-24920|-24920|-27125|-27125
chr10	127838450	127846852	Obfc2b	0	-	MACS_peak_2022|MACS_peak_2023|MA	5413|18847|26739|28944
chr10	127896291	127927230	Smarcc2	0	+	MACS_peak_2024|MACS_peak_2024|MA	22700|22700|22700|20495|20495|20495
chr10	127927916	127930881	Myl6	0	-	MACS_peak_2026|MACS_peak_2027|MA	18094|23213|23864|25812|27142
chr10	127947305	127962915	Esyt1	0	-	MACS_peak_2026|MACS_peak_2027|MA	-13940|-8821|-8170|-6222|-4892|-186
chr10	127931212	127935741	Myl6b	0	-	MACS_peak_2026|MACS_peak_2027|MA	13234|18353|19004|20952|22282|26988
chr10	127985165	127986224	Rpl41	0	-	MACS_peak_2030|MACS_peak_2031|MA	-29531|-28201|-23495
chr10	127980620	127984800	Zc3h10	0	-	MACS_peak_2030|MACS_peak_2031|MA	-28107|-26777|-22071
chr10	128185577	128203619	Wibg	0	+	MACS_peak_2040|MACS_peak_2040|MA	17114|16471|17114|15170|14527|15170|4601|3958|4601
chr10	128134994	128142107	Cdk2	0	-	MACS_peak_2040|MACS_peak_2040|MA	26356|26356|28300|28300
chr10	128157191	128181112	Dgka	0	-	MACS_peak_2040|MACS_peak_2042|MA	-12649|-10705|-136
chr10	128143313	128157294	Si	0	+	MACS_peak_2040|MACS_peak_2042	-25150|-27094
chr10	128227965	128237880	Mmp19	0	+	MACS_peak_2051|MACS_peak_2051	-16022|-15979
chr10	128254512	128258687	Ormdl2	0	-	MACS_peak_2051	-14700
chr10	128258826	128314693	Sarnp	0	+	MACS_peak_2051	14839
chr10	128242731	128256502	Dnajc14	0	+	MACS_peak_2051	-1256
chr10	128237091	128241426	1110012D	0	-	MACS_peak_2051	2561
chr10	128345974	128349874	Cd63	0	+	MACS_peak_2054|MACS_peak_2054	-9085|-8138
chr10	128370868	128395340	Itga7	0	+	MACS_peak_2054|MACS_peak_2055|MA	15809|-8062|-26777
chr10	128321601	128328774	Gdf11	0	-	MACS_peak_2054	26285
chr10	128356969	128360580	Bloc1s1	0	-	MACS_peak_2054|MACS_peak_2055	-5521|18350
chr10	128350646	128356353	Rdh5	0	-	MACS_peak_2054|MACS_peak_2055	-1294|22577
chr10	128395332	128398044	Mettl7b	0	-	MACS_peak_2055|MACS_peak_2058	-19114|-399
chr10	128426969	128427908	Olfr9	0	+	MACS_peak_2058	29324
chr10	17516024	17565469	Txlnb	0	+	MACS_peak_1272	-21432
chr10	18189786	18253695	Nhsl1	0	+	MACS_peak_1277	-28109
chr10	18259928	18265882	Hebp2	0	-	MACS_peak_1278|MACS_peak_1279|MA	-21253|-20394|-19477|-19235
chr10	20672390	20800235	Ahi1	0	+	MACS_peak_1289|MACS_peak_1290|MA	-13387|-19498|-26925
chr10	22081699	22093945	Raet1d	0	+	MACS_peak_1314|MACS_peak_1315	18351|17478
chr10	22423684	22451253	Tbpl1	0	-	MACS_peak_1316|MACS_peak_1317|MA	-26552|-22271|-19444
chr10	24635012	24647276	Arg1	0	-	MACS_peak_1329	-25359
chr10	29915814	29920346	Cenpw	0	-	MACS_peak_1332	-20457
chr10	33646741	33671018	Zufsp	0	-	MACS_peak_1335	-1405
chr10	39089604	39285180	Fyn	0	+	MACS_peak_1337|MACS_peak_1337|MA	-192|-192|-192
chr10	39721378	39745074	AI317395	0	-	MACS_peak_1353	15424
chr10	40552698	40602949	Cdc40	0	-	MACS_peak_1357	-15273
chr10	40603339	40658375	Wasf1	0	+	MACS_peak_1357	15663
chr10	41196119	41206834	Mical1	0	+	MACS_peak_1364|MACS_peak_1364|MA	-8827|-8827|-12995|-12995|-13196|-13196
chr10	41206977	41210146	Smpd2	0	-	MACS_peak_1364|MACS_peak_1366|MA	-5200|-1032|-831
chr10	41210244	41234094	Ppil6	0	+	MACS_peak_1364|MACS_peak_1366|MA	5298|1130|929
chr10	41239305	41250848	Cd164	0	+	MACS_peak_1367	29990
chr10	42481301	42552320	Sec63	0	+	MACS_peak_1375	-29741
chr10	43593995	43621542	Qrsl1	0	-	MACS_peak_1378	6085
chr10	43621612	43667668	Rtn4ip1	0	+	MACS_peak_1378	-6015
chr10	4541075	4547383	Fbxo5	0	+	MACS_peak_1182	18272
chr10	4522597	4534654	Mtrf1l	0	+	MACS_peak_1182	-206
chr10	45297634	45432151	Hace1	0	+	MACS_peak_1381	22288
chr10	4795848	5009592	Syne1	0	+	MACS_peak_1184|MACS_peak_1194	-8615|-21733
chr10	52262123	52265545	Zfa	0	-	MACS_peak_1398	-477
chr10	53316766	53329021	Asf1a	0	+	MACS_peak_1400	-28863
chr10	53257129	53350245	Mcm9	0	-	MACS_peak_1400	-4616
chr10	53625838	53795602	Man1a	0	-	MACS_peak_1410|MACS_peak_1411	-952|-179
chr10	56097105	56110225	Gja1	0	+	MACS_peak_1413	-112
chr10	57235583	57252335	Serinc1	0	-	MACS_peak_1416	-13053
chr10	57857120	57887438	Lims1	0	+	MACS_peak_1417|MACS_peak_1418	-15248|-18101
chr10	58604374	58684595	10-Sep	0	-	MACS_peak_1421|MACS_peak_1421|MA	700|700|3926|3926
chr10	58684669	58689181	Ankrd57	0	+	MACS_peak_1421|MACS_peak_1422	-626|-3852
chr10	58786043	58836051	P4ha1	0	+	MACS_peak_1423|MACS_peak_1426|MA	-13808|-25002|-28873
chr10	58866432	58884724	Pla2g12b	0	+	MACS_peak_1434	16968
chr10	5958432	5979467	Zbtb2	0	+	MACS_peak_1199	19752
chr10	5891400	5913936	1700052N	0	-	MACS_peak_1199	24744
chr10	5914188	5943372	Rmnd1	0	+	MACS_peak_1199	-24492
chr10	5917374	5943370	Gm5512	0	+	MACS_peak_1199	-21306
chr10	59809598	59835432	4632428N	0	+	MACS_peak_1452|MACS_peak_1452	-145|-145
chr10	60552090	60557077	Pcbd1	0	+	MACS_peak_1465|MACS_peak_1466|MA	-12406|-15196|-16128
chr10	60659859	60736186	Adamts14	0	-	MACS_peak_1474	-2385
chr10	60760583	60767011	Prf1	0	+	MACS_peak_1474	26782
chr10	61143068	61156045	Sar1a	0	+	MACS_peak_1476	-3809
chr10	61158261	61165521	Tysnd1	0	+	MACS_peak_1476	11384
chr10	61201394	61246612	H2afy2	0	-	MACS_peak_1480	726
chr10	62042994	62065046	Ddx21	0	-	MACS_peak_1490	22548
chr10	62078770	62113946	Ddx50	0	-	MACS_peak_1490|MACS_peak_1491	-26352|-4143
chr10	62442970	62481490	Rufy2	0	+	MACS_peak_1492	-2552
chr10	62477411	62486597	Hnrnph3	0	-	MACS_peak_1493|MACS_peak_1494|MA	-6566|-5016|-4393
chr10	62487259	62521560	3110049J2	0	+	MACS_peak_1493|MACS_peak_1494|MA	7228|5678|5055
chr10	68996455	69490184	Ank3	0	+	MACS_peak_1508|MACS_peak_1508|MA	-321|-321|-321|-321|-321|-321|-321|27264|27264
chr10	7309597	7345273	Lrp11	0	+	MACS_peak_1204	-13076
chr10	7401006	7436259	Lats1	0	+	MACS_peak_1205	-20531
chr10	7445797	7482948	Katna1	0	+	MACS_peak_1205	24260
chr10	7398768	7400956	BC020402	0	-	MACS_peak_1205	20581
chr10	74523640	74647668	Bcr	0	+	MACS_peak_1529	-249
chr10	74420221	74495331	Rtdr1	0	-	MACS_peak_1529|MACS_peak_1529|MA	28558|28591|28591|28558
chr10	74499833	74516845	Rab36	0	+	MACS_peak_1529	-24056
chr10	75233977	75236119	Ddt	0	-	MACS_peak_1532	-21843
chr10	75236866	75244159	Gstt3	0	-	MACS_peak_1532	-29883
chr10	75108854	75227102	Cabin1	0	-	MACS_peak_1532	-12826
chr10	75359513	75384359	Smarcb1	0	-	MACS_peak_1533|MACS_peak_1533|MA	2059|2059|3935|3935|4910|4910|10798|10798|14338|14338|15766|15766|16029|16029
chr10	75385968	75395208	Mmp11	0	-	MACS_peak_1533|MACS_peak_1534|MA	-8790|-6914|-5939|-51|3489|4917|5180
chr10	75411056	75412391	Vpreb3	0	+	MACS_peak_1533|MACS_peak_1534|MA	24638|22762|21787|15899|12359|10931|10668
chr10	75398317	75400479	Chchd10	0	+	MACS_peak_1533|MACS_peak_1534|MA	11899|10023|9048|3160|-380|-1808|-2071
chr10	75417258	75472334	Gm5134	0	+	MACS_peak_1534|MACS_peak_1535|MA	28964|27989|22101|18561|17133|16870
chr10	75716580	75724064	S100b	0	+	MACS_peak_1544	-22803
chr10	75813998	75905657	Pcnt	0	-	MACS_peak_1545	27683
chr10	75931715	75978602	Mcm3ap	0	+	MACS_peak_1545	-1625
chr10	75911825	75923963	2610028H	0	+	MACS_peak_1545	-21515
chr10	75922311	75931859	A130042E	0	-	MACS_peak_1545	1481
chr10	77441394	77448183	1810043G	0	+	MACS_peak_1597	21924
chr10	77370467	77432617	Trpm2	0	-	MACS_peak_1597	-13147
chr10	77633654	77647894	Pwp2	0	-	MACS_peak_1600	-8104
chr10	77624811	77632513	D10Jhu81e	0	-	MACS_peak_1600	7277
chr10	78047247	78054709	Syde1	0	-	MACS_peak_1604|MACS_peak_1606	-14570|-10841
chr10	78037244	78047245	Ilvbl	0	+	MACS_peak_1604|MACS_peak_1606	-2895|-6624
chr10	78989173	78996532	Ppap2c	0	-	MACS_peak_1608	15914
chr10	79002989	79017836	Mier2	0	-	MACS_peak_1608	-5390
chr10	79179378	79198853	Hcn2	0	+	MACS_peak_1612|MACS_peak_1613|MA	-19925|-24017|-24288
chr10	79217863	79219706	Fgf22	0	+	MACS_peak_1612|MACS_peak_1613|MA	18560|14468|14197|-22205

chr10	79198869	79209326	Polrmt	0	-	MACS_peak_1612|MACS_peak_1613|MA	-10023|-5931|-5660
chr10	79221259	79229666	Rnf126	0	-	MACS_peak_1613|MACS_peak_1613|MA	-26271|-26291|-26000|-26020|10402|10382
chr10	79244218	79251730	Prssl1	0	-	MACS_peak_1615	-11662
chr10	79256316	79283641	Palm	0	+	MACS_peak_1615|MACS_peak_1615	16248|16248
chr10	79240018	79245375	Fstl3	0	+	MACS_peak_1615	-50
chr10	79439864	79447075	ORF61	0	-	MACS_peak_1619|MACS_peak_1622	20124|27637
chr10	79479416	79494216	Hmha1	0	+	MACS_peak_1619|MACS_peak_1619|MA	12217|15220|4704|7707
chr10	79451344	79458145	Cnn2	0	+	MACS_peak_1619|MACS_peak_1622	-15855|-23368
chr10	79460359	79478317	Abca7	0	+	MACS_peak_1619|MACS_peak_1622	-6840|-14353
chr10	79498697	79502404	Polr2e	0	-	MACS_peak_1622	-27692
chr10	79630585	79634398	Cirbp	0	+	MACS_peak_1624	17845
chr10	79642226	79652753	Efna2	0	+	MACS_peak_1624	29486
chr10	79593178	79602112	Dos	0	-	MACS_peak_1624	10628
chr10	79611034	79621112	Midn	0	+	MACS_peak_1624	-1706
chr10	79605059	79608563	Atp5d	0	+	MACS_peak_1624	-7681
chr10	79635688	79637864	1600002K	0	+	MACS_peak_1624	22948
chr10	79803460	79811191	Adamtsl5	0	-	MACS_peak_1626|MACS_peak_1626|MA	-14680|-14646|-13150|-13116|-12498|-12464|-6312|-6278|-4783|-4749
chr10	79784029	79792218	Pcsk4	0	-	MACS_peak_1626|MACS_peak_1627|MA	4293|5823|6475|12661|14190
chr10	79780999	79783293	2310011J	0	-	MACS_peak_1626|MACS_peak_1627|MA	13218|14748|15400|21586|23115
chr10	79792914	79799186	Reep6	0	+	MACS_peak_1626|MACS_peak_1627|MA	-3597|-5127|-5779|-11965|-13494
chr10	79819203	79828234	Plk5	0	+	MACS_peak_1626|MACS_peak_1627|MA	22692|21162|20510|14324|12795
chr10	80128073	80134721	Mknk2	0	-	MACS_peak_1638	8
chr10	80105361	80119816	Btbd2	0	-	MACS_peak_1638	14913
chr10	80147997	80164565	Mobkl2a	0	-	MACS_peak_1638	-29836
chr10	80411368	80464110	Gng7	0	-	MACS_peak_1642|MACS_peak_1642	28896|15336
chr10	80506817	80522899	Sgta	0	-	MACS_peak_1642	-29893
chr10	80491284	80496657	Slc39a3	0	-	MACS_peak_1642	-3651
chr10	80482334	80488122	Diras1	0	-	MACS_peak_1642	4884
chr10	80695907	80714868	Zfr2	0	+	MACS_peak_1653	-18367
chr10	80720289	80725726	Matk	0	+	MACS_peak_1653	6015
chr10	80730916	80735991	Apba3	0	+	MACS_peak_1653	16642
chr10	80727466	80729671	Mrpl54	0	-	MACS_peak_1653	-15397
chr10	80667257	80693524	Atcay	0	-	MACS_peak_1653	20750
chr10	81038276	81053590	Tle2	0	+	MACS_peak_1654	-17254
chr10	81053649	81063645	Tle6	0	-	MACS_peak_1654	-8115
chr10	81069052	81072580	BC025920	0	+	MACS_peak_1654	13522
chr10	82321950	82360469	Txnrd1	0	+	MACS_peak_1656|MACS_peak_1656|MA	-18124|-16989|-18124|-20040|-18905|-20040|-22632|-21497|-22632|-24758|-23623|-24758|-25815|-24680|-25815|-26034|-24899|-26034
chr10	82329370	82330674	Eid3	0	+	MACS_peak_1656|MACS_peak_1657|MA	-10704|-12620|-15212|-17338|-18395|-18614
chr10	82950191	82996885	Aldh1l2	0	-	MACS_peak_1673|MACS_peak_1674|MA	-29981|-27465|-26057|-25471|-21779|-20512|-18026|-15751|-13793|-6769|-95
chr10	83006685	83059218	A230046K	0	+	MACS_peak_1680|MACS_peak_1681|MA	27826|25551|23593|16569|9895
chr10	84582179	84590772	Mterfd3	0	-	MACS_peak_1708|MACS_peak_1709|MA	15074|15716|18467|18782|19923|20676|23259
chr10	84594444	84647799	Cry1	0	-	MACS_peak_1715	-535
chr10	86168555	86239588	BC030307	0	+	MACS_peak_1725|MACS_peak_1725|MA	3960|3960|10914
chr10	86153585	86168189	Hsp90b1	0	-	MACS_peak_1725	-3594
chr10	86148271	86152699	1810014B	0	+	MACS_peak_1725	-16324
chr10	86241749	86301134	Nt5dc3	0	+	MACS_peak_1726|MACS_peak_1727	-238|-25873
chr10	88192285	88193846	Gm4925	0	-	MACS_peak_1733	19614
chr10	88194158	88205947	Arl1	0	+	MACS_peak_1733	-19302
chr10	89036764	89083994	Slc17a8	0	-	MACS_peak_1735|MACS_peak_1736	18273|19149
chr10	89102851	89149030	Scyl2	0	-	MACS_peak_1739|MACS_peak_1740	-28414|-19500
chr10	89174717	89195040	Actr6	0	-	MACS_peak_1742|MACS_peak_1743	-15132|12849
chr10	89207735	89282614	Uhrf1bp1l	0	+	MACS_peak_1742|MACS_peak_1743	27827|-154
chr10	90452055	90545488	Apaf1	0	-	MACS_peak_1753|MACS_peak_1753|MA	-24355|-24355|-22574|-22574|-3150|-3150|-243|-243
chr10	90545783	90561403	Ikbip	0	+	MACS_peak_1753|MACS_peak_1753|MA	24650|24650|22869|22869|3445|3445|538|538
chr10	92623620	92704087	Cdk17	0	+	MACS_peak_1760	-64
chr10	92625529	92625647	Mir1931	0	+	MACS_peak_1760	1845
chr10	92916140	92947641	Lta4h	0	+	MACS_peak_1762	-21130
chr10	92951512	92979488	Hal	0	+	MACS_peak_1762	14242
chr10	93426512	93426608	Mir331	0	-	MACS_peak_1767	16543
chr10	93610675	93659959	Nr2c1	0	+	MACS_peak_1771	-23215
chr10	93661753	93683693	Ndufa12	0	+	MACS_peak_1771	27863
chr11	100277007	100286153	Fkbp10	0	+	MACS_peak_2939|MACS_peak_2939	29412|29412
chr11	100231932	100259053	Jup	0	-	MACS_peak_2939	-11458
chr11	100270063	100276133	1110036O	0	-	MACS_peak_2939	-28538
chr11	100601692	100621092	Kcnh4	0	-	MACS_peak_2942|MACS_peak_2943	8708|10409
chr11	100623006	100624245	Hcrt	0	-	MACS_peak_2942|MACS_peak_2943	5555|7256
chr11	100627645	100632271	Ghdc	0	-	MACS_peak_2942|MACS_peak_2943	-2471|-770
chr11	100642044	100711899	Stat5b	0	-	MACS_peak_2944	14853
chr11	100721797	100746482	Stat5a	0	+	MACS_peak_2944|MACS_peak_2944	-4955|-6088
chr11	100948603	100953520	Mlx	0	+	MACS_peak_2953|MACS_peak_2953|MA	24079|24079|24079|3897|3897|3897
chr11	100939724	100941819	Hsd17b1	0	+	MACS_peak_2953|MACS_peak_2958	15200|-4982
chr11	100931407	100938983	Naglu	0	+	MACS_peak_2953|MACS_peak_2958	6883|-13299
chr11	100943938	100947933	Coasy	0	+	MACS_peak_2953|MACS_peak_2958	19414|-768
chr11	100953470	100956715	Psmc3ip	0	-	MACS_peak_2958	-12009
chr11	101414171	101443259	Nbr1	0	+	MACS_peak_2973|MACS_peak_2974|MA	-2987|-3328|-12884|-15646|-19700|-20021
chr11	101350077	101413269	Brca1	0	-	MACS_peak_2973|MACS_peak_2974|MA	3889|4230|13786|16548|20602|20923
chr11	101443555	101453099	Tmem106a	0	+	MACS_peak_2973|MACS_peak_2974|MA	26397|26056|16500|13738|9684|9363
chr11	101594269	101628671	Dhx8	0	+	MACS_peak_2991	-18029
chr11	102291635	102298121	Grn	0	+	MACS_peak_3001	-13754
chr11	102314610	102331197	Itga2b	0	-	MACS_peak_3001	-25808
chr11	102298295	102308977	Fam171a2	0	-	MACS_peak_3001	-3588
chr11	102963001	102973513	Acbd4	0	+	MACS_peak_3003|MACS_peak_3007	29987|-1316
chr11	102931609	102962972	Plcd3	0	-	MACS_peak_3003|MACS_peak_3007	-29958|1345
chr11	102977638	102981038	Hexim1	0	+	MACS_peak_3007	13321
chr11	103032451	103060214	Fmnl1	0	+	MACS_peak_3010|MACS_peak_3010|MA	-113|-113|-10611|-10611|-10988|-10988|-22857|-22857|-23590|-23590|-27528|-27528
chr11	103069440	103079746	4933400C	0	-	MACS_peak_3019|MACS_peak_3020|MA	-24438|-23705|-19767
chr11	103192813	103206016	Arhgap27	0	-	MACS_peak_3027|MACS_peak_3028	-12728|-4353
chr11	103221050	103224698	Sh3d20	0	-	MACS_peak_3028|MACS_peak_3029	-23035|16907
chr11	105153959	105212459	Mrc2	0	+	MACS_peak_3035	11016
chr11	106060669	106077681	Ccdc47	0	-	MACS_peak_3093|MACS_peak_3094|MA	-14291|-11392|-8079|-4632
chr11	106078239	106110454	Ddx42	0	+	MACS_peak_3093|MACS_peak_3094|MA	14849|11950|8637|5190
chr11	106024649	106054862	Strada	0	-	MACS_peak_3093|MACS_peak_3094|MA	8528|11427|14740|18187
chr11	106238969	106243955	Icam2	0	-	MACS_peak_3099|MACS_peak_3101|MA	17648|23150|24887|27374
chr11	106236139	106238428	2310007L	0	+	MACS_peak_3099	-25464
chr11	108256610	108275710	Apoh	0	+	MACS_peak_3131|MACS_peak_3132|MA	-4926|-7464|-8051|-8389
chr11	108286579	108721929	Ccdc46	0	+	MACS_peak_3131|MACS_peak_3132|MA	25043|22505|21918|21580
chr11	109287259	109299462	Amz2	0	+	MACS_peak_3137|MACS_peak_3138	-1089|-3805
chr11	109434834	109472703	Wipi1	0	-	MACS_peak_3141	-28229
chr11	113106168	113427129	Slc39a11	0	-	MACS_peak_3149|MACS_peak_3149	-3753|-3753
chr11	113588163	113613129	Cdc42ep4	0	-	MACS_peak_3151|MACS_peak_3151	-992|-337
chr11	115048800	115108339	Tmem104	0	+	MACS_peak_3156	246
chr11	115024654	115042492	Slc9a3r1	0	+	MACS_peak_3156	-23900
chr11	115044145	115048630	Nat9	0	-	MACS_peak_3156	-76
chr11	115110482	115128557	Grin2c	0	-	MACS_peak_3159	-20855
chr11	115196047	115208240	Otop3	0	+	MACS_peak_3161|MACS_peak_3162|MA	-14141|-14425|-15814|-20312|-23992|-24297|-25048|-25674|-26974
chr11	115209022	115229033	C630004H	0	-	MACS_peak_3161|MACS_peak_3162|MA	-18845|-18561|-17172|-12674|-8994|-8689|-7938|-7312|-6012|-105|23217
chr11	115176505	115183232	Ush1g	0	-	MACS_peak_3161|MACS_peak_3162|MA	26956|27240|28629
chr11	115243229	115257446	Cdr2l	0	+	MACS_peak_3166|MACS_peak_3169|MA	26870|23190|22885|22134|21508|20208|14301|-9021
chr11	115265079	115272227	Ict1	0	+	MACS_peak_3175	12829
chr11	115277010	115281233	Atp5h	0	-	MACS_peak_3175	-28983
chr11	115281439	115292587	Kctd2	0	+	MACS_peak_3175	29189
chr11	115465464	115468938	Mrps7	0	+	MACS_peak_3176	-16806
chr11	115476296	115489453	Slc25a19	0	-	MACS_peak_3176	-7183
chr11	115469234	115473817	Mif4gd	0	-	MACS_peak_3176|MACS_peak_3176	8453|8003
chr11	115445568	115465220	Gga3	0	-	MACS_peak_3176	17050
chr11	115626746	115660347	2310067B	0	+	MACS_peak_3177	-29288
chr11	115676052	115684416	Tsen54	0	+	MACS_peak_3177	20018
chr11	115660665	115674906	Caskin2	0	-	MACS_peak_3177	-18872
chr11	115685371	115717094	Llgl2	0	+	MACS_peak_3177	29337
chr11	115773330	115775231	1110017F	0	+	MACS_peak_3178|MACS_peak_3179|MA	17602|-16183|-21039
chr11	115761452	115767582	2210020M	0	+	MACS_peak_3178|MACS_peak_3179	5724|-28061
chr11	115794972	115826848	Sap30bp	0	+	MACS_peak_3179|MACS_peak_3180	5459|603

<table>
<tr><td>chr11</td><td>115753990</td><td>115794320</td><td>Recql5</td><td>0</td><td>-</td><td>MACS_peak_3179|MACS_peak_3180</td><td>-4807|49</td></tr>
<tr><td>chr11</td><td>115836038</td><td>115869725</td><td>Itgb4</td><td>0</td><td>+</td><td>MACS_peak_3183|MACS_peak_3183|MA(</td><td>-5992|-5992|-14227|-14227</td></tr>
<tr><td>chr11</td><td>115869670</td><td>115874033</td><td>Galk1</td><td>0</td><td>-</td><td>MACS_peak_3189</td><td>-23768</td></tr>
<tr><td>chr11</td><td>115923409</td><td>115939275</td><td>Unc13d</td><td>0</td><td>-</td><td>MACS_peak_3206</td><td>3277</td></tr>
<tr><td>chr11</td><td>115939886</td><td>115948278</td><td>Wbp2</td><td>0</td><td>-</td><td>MACS_peak_3206</td><td>-5726</td></tr>
<tr><td>chr11</td><td>115967139</td><td>115971330</td><td>Trim47</td><td>0</td><td>-</td><td>MACS_peak_3206</td><td>-28778</td></tr>
<tr><td>chr11</td><td>115993130</td><td>116000182</td><td>Mrpl38</td><td>0</td><td>-</td><td>MACS_peak_3208</td><td>20054</td></tr>
<tr><td>chr11</td><td>116003598</td><td>116029492</td><td>Fbf1</td><td>0</td><td>-</td><td>MACS_peak_3208</td><td>-9256</td></tr>
<tr><td>chr11</td><td>115986021</td><td>115992442</td><td>Trim65</td><td>0</td><td>-</td><td>MACS_peak_3208</td><td>27794</td></tr>
<tr><td>chr11</td><td>116142252</td><td>116145252</td><td>Galr2</td><td>0</td><td>+</td><td>MACS_peak_3209|MACS_peak_3212</td><td>15496|-24018</td></tr>
<tr><td>chr11</td><td>116081872</td><td>116099405</td><td>Evpl</td><td>0</td><td>-</td><td>MACS_peak_3209</td><td>27351</td></tr>
<tr><td>chr11</td><td>116106479</td><td>116135531</td><td>Srp68</td><td>0</td><td>-</td><td>MACS_peak_3209</td><td>-8775</td></tr>
<tr><td>chr11</td><td>116149311</td><td>116168052</td><td>Exoc7</td><td>0</td><td>-</td><td>MACS_peak_3212|MACS_peak_3212</td><td>-1782|-1782</td></tr>
<tr><td>chr11</td><td>116197658</td><td>116274346</td><td>Rnf157</td><td>0</td><td>-</td><td>MACS_peak_3214|MACS_peak_3215|MA(</td><td>-23377|-22199|-19342</td></tr>
<tr><td>chr11</td><td>116332129</td><td>116351660</td><td>Prpsap1</td><td>0</td><td>-</td><td>MACS_peak_3218</td><td>-2202</td></tr>
<tr><td>chr11</td><td>116455000</td><td>116458993</td><td>Aanat</td><td>0</td><td>+</td><td>MACS_peak_3223|MACS_peak_3223|MA(</td><td>12592|12592|-5129|-5129|-7988|-7988|-8556|-8556|-10085|-10085|-11651|-11651</td></tr>
<tr><td>chr11</td><td>116399066</td><td>116442761</td><td>Ube2o</td><td>0</td><td>-</td><td>MACS_peak_3223|MACS_peak_3224|MA(</td><td>-353|17368|20227|20795|22324|23890</td></tr>
<tr><td>chr11</td><td>116459478</td><td>116485566</td><td>Rhbdf2</td><td>0</td><td>-</td><td>MACS_peak_3224|MACS_peak_3224|MA(</td><td>-25437|-28204|-22578|-25345|-22010|-24777|-20481|-23248|-18915|-21682</td></tr>
<tr><td>chr11</td><td>116518421</td><td>116529701</td><td>Gm11744</td><td>0</td><td>+</td><td>MACS_peak_3233</td><td>-15152</td></tr>
<tr><td>chr11</td><td>116538018</td><td>116555974</td><td>St6galnac2</td><td>0</td><td>-</td><td>MACS_peak_3233</td><td>-22401</td></tr>
<tr><td>chr11</td><td>116506908</td><td>116515627</td><td>Cygb</td><td>0</td><td>-</td><td>MACS_peak_3233</td><td>17946</td></tr>
<tr><td>chr11</td><td>116532973</td><td>116537110</td><td>1810032O(</td><td>0</td><td>+</td><td>MACS_peak_3233|MACS_peak_3233|MA(</td><td>-600|-600|-600</td></tr>
<tr><td>chr11</td><td>116535460</td><td>116535537</td><td>Snord1b</td><td>0</td><td>+</td><td>MACS_peak_3233</td><td>1887</td></tr>
<tr><td>chr11</td><td>116533818</td><td>116533896</td><td>Snord1c</td><td>0</td><td>+</td><td>MACS_peak_3233</td><td>245</td></tr>
<tr><td>chr11</td><td>116535910</td><td>116535986</td><td>Snord1a</td><td>0</td><td>+</td><td>MACS_peak_3233</td><td>2337</td></tr>
<tr><td>chr11</td><td>116711210</td><td>116714408</td><td>Srsf2</td><td>0</td><td>-</td><td>MACS_peak_3234</td><td>-12618</td></tr>
<tr><td>chr11</td><td>116664713</td><td>116689360</td><td>Mxra7</td><td>0</td><td>-</td><td>MACS_peak_3234</td><td>12430</td></tr>
<tr><td>chr11</td><td>116704828</td><td>116711053</td><td>1110005A(</td><td>0</td><td>+</td><td>MACS_peak_3234</td><td>3038</td></tr>
<tr><td>chr11</td><td>116698745</td><td>116704763</td><td>Jmjd6</td><td>0</td><td>-</td><td>MACS_peak_3234</td><td>-2973</td></tr>
<tr><td>chr11</td><td>116715328</td><td>116736951</td><td>Mfsd11</td><td>0</td><td>+</td><td>MACS_peak_3234</td><td>13538</td></tr>
<tr><td>chr11</td><td>116976485</td><td>117020580</td><td>Sec14l1</td><td>0</td><td>+</td><td>MACS_peak_3235|MACS_peak_3235|MA(</td><td>-26323|-26323|-26323|-26323</td></tr>
<tr><td>chr11</td><td>117658973</td><td>117668622</td><td>6030468B:</td><td>0</td><td>+</td><td>MACS_peak_3241|MACS_peak_3243|MA(</td><td>28219|21963|19333|18681</td></tr>
<tr><td>chr11</td><td>117627299</td><td>117641997</td><td>Tmc6</td><td>0</td><td>-</td><td>MACS_peak_3241|MACS_peak_3241|MA(</td><td>-11243|-11181|-4987|-4925|-2357|-2295|-1705|-1643</td></tr>
<tr><td>chr11</td><td>117644363</td><td>117654342</td><td>Tmc8</td><td>0</td><td>+</td><td>MACS_peak_3241|MACS_peak_3243|MA(</td><td>13609|7353|4723|4071</td></tr>
<tr><td>chr11</td><td>118120967</td><td>118151558</td><td>Usp36</td><td>0</td><td>-</td><td>MACS_peak_3247|MACS_peak_3248|MA(</td><td>-27809|-17243|-10989</td></tr>
<tr><td>chr11</td><td>118025479</td><td>118109906</td><td>Cyth1</td><td>0</td><td>-</td><td>MACS_peak_3247|MACS_peak_3247|MA(</td><td>13843|13843|24409|24409</td></tr>
<tr><td>chr11</td><td>119004357</td><td>119089813</td><td>Tbc1d16</td><td>0</td><td>-</td><td>MACS_peak_3263</td><td>-17893</td></tr>
<tr><td>chr11</td><td>119089885</td><td>119126526</td><td>Ccdc40</td><td>0</td><td>+</td><td>MACS_peak_3263</td><td>17965</td></tr>
<tr><td>chr11</td><td>119129280</td><td>119147012</td><td>Gaa</td><td>0</td><td>+</td><td>MACS_peak_3269|MACS_peak_3269|MA(</td><td>-6734|-6693|-16173|-16132</td></tr>
<tr><td>chr11</td><td>119149676</td><td>119161357</td><td>Eif4a3</td><td>0</td><td>-</td><td>MACS_peak_3269|MACS_peak_3280</td><td>-25343|-15904</td></tr>
<tr><td>chr11</td><td>119204802</td><td>119216824</td><td>Sgsh</td><td>0</td><td>-</td><td>MACS_peak_3283</td><td>24963</td></tr>
<tr><td>chr11</td><td>119216870</td><td>119242390</td><td>Slc26a11</td><td>0</td><td>+</td><td>MACS_peak_3283</td><td>-24917</td></tr>
<tr><td>chr11</td><td>119251785</td><td>119251873</td><td>Mir1932</td><td>0</td><td>+</td><td>MACS_peak_3283</td><td>9998</td></tr>
<tr><td>chr11</td><td>119352660</td><td>119372779</td><td>A730011L(</td><td>0</td><td>+</td><td>MACS_peak_3295|MACS_peak_3295|MA(</td><td>-17127|-16854|-17713|-17440</td></tr>
<tr><td>chr11</td><td>119775123</td><td>119780866</td><td>Chmp6</td><td>0</td><td>+</td><td>MACS_peak_3298|MACS_peak_3299</td><td>28837|19450</td></tr>
<tr><td>chr11</td><td>119960247</td><td>119961738</td><td>1810043H(</td><td>0</td><td>+</td><td>MACS_peak_3301|MACS_peak_3303|MA(</td><td>26476|-11366|-18006</td></tr>
<tr><td>chr11</td><td>119868631</td><td>119908029</td><td>Aatk</td><td>0</td><td>-</td><td>MACS_peak_3301</td><td>25742</td></tr>
<tr><td>chr11</td><td>119925743</td><td>119948141</td><td>Azi1</td><td>0</td><td>-</td><td>MACS_peak_3301|MACS_peak_3303</td><td>-14370|23472</td></tr>
<tr><td>chr11</td><td>119951835</td><td>119960045</td><td>2410002I0</td><td>0</td><td>-</td><td>MACS_peak_3301|MACS_peak_3303|MA(</td><td>-26274|11568|18208</td></tr>
<tr><td>chr11</td><td>119965264</td><td>120012665</td><td>Slc38a10</td><td>0</td><td>-</td><td>MACS_peak_3305|MACS_peak_3305|MA(</td><td>-22118|-22118|-22118|-22118|-22118|-22118</td></tr>
<tr><td>chr11</td><td>120489666</td><td>120494861</td><td>Mafg</td><td>0</td><td>-</td><td>MACS_peak_3307|MACS_peak_3308|MA(</td><td>2654|3574|6368|7675|8206|8669</td></tr>
<tr><td>chr11</td><td>120471400</td><td>120479204</td><td>Pcyt2</td><td>0</td><td>-</td><td>MACS_peak_3307|MACS_peak_3308|MA(</td><td>18311|19231|22025|23332|23863|24326</td></tr>
<tr><td>chr11</td><td>120507345</td><td>120509651</td><td>Myadml2</td><td>0</td><td>-</td><td>MACS_peak_3307|MACS_peak_3308|MA(</td><td>-12136|-11216|-8422|-7115|-6584|-6121</td></tr>
<tr><td>chr11</td><td>120497025</td><td>120504984</td><td>Pycr1</td><td>0</td><td>-</td><td>MACS_peak_3307|MACS_peak_3308|MA(</td><td>-7469|-6549|-3755|-2448|-1917|-1454</td></tr>
<tr><td>chr11</td><td>120479685</td><td>120486316</td><td>Sirt7</td><td>0</td><td>-</td><td>MACS_peak_3307|MACS_peak_3308|MA(</td><td>11199|12119|14913|16220|16751|17214</td></tr>
<tr><td>chr11</td><td>120469790</td><td>120470414</td><td>Npb</td><td>0</td><td>+</td><td>MACS_peak_3307|MACS_peak_3308</td><td>-27725|-28645</td></tr>
<tr><td>chr11</td><td>120515118</td><td>120522151</td><td>Notum</td><td>0</td><td>-</td><td>MACS_peak_3307|MACS_peak_3308|MA(</td><td>-24636|-23716|-20922|-19615|-19084|-18621</td></tr>
<tr><td>chr11</td><td>121065746</td><td>121083969</td><td>Hexdc</td><td>0</td><td>+</td><td>MACS_peak_3322|MACS_peak_3322|MA(</td><td>-1001|2209|-19018|-15808|-24744|-21534</td></tr>
<tr><td>chr11</td><td>121038906</td><td>121065962</td><td>1110031I0</td><td>0</td><td>-</td><td>MACS_peak_3322|MACS_peak_3323|MA(</td><td>785|18802|24528</td></tr>
<tr><td>chr11</td><td>121083902</td><td>121090579</td><td>BC017643</td><td>0</td><td>-</td><td>MACS_peak_3322|MACS_peak_3323|MA(</td><td>-23832|-5815|-89</td></tr>
<tr><td>chr11</td><td>121098549</td><td>121117170</td><td>Narf</td><td>0</td><td>+</td><td>MACS_peak_3323|MACS_peak_3325</td><td>13785|8059</td></tr>
<tr><td>chr11</td><td>121296266</td><td>121304819</td><td>Fn3k</td><td>0</td><td>+</td><td>MACS_peak_3330|MACS_peak_3330</td><td>5861|5861</td></tr>
<tr><td>chr11</td><td>121313262</td><td>121478484</td><td>Tbcd</td><td>0</td><td>+</td><td>MACS_peak_3330</td><td>22857</td></tr>
<tr><td>chr11</td><td>121282686</td><td>121292080</td><td>Fn3krp</td><td>0</td><td>+</td><td>MACS_peak_3330</td><td>-7719</td></tr>
<tr><td>chr11</td><td>121563740</td><td>121578703</td><td>Metrnl</td><td>0</td><td>+</td><td>MACS_peak_3332</td><td>-155</td></tr>
<tr><td>chr11</td><td>121477522</td><td>121534465</td><td>B3gntl1</td><td>0</td><td>-</td><td>MACS_peak_3332</td><td>29430</td></tr>
<tr><td>chr11</td><td>20101604</td><td>20126859</td><td>Rab1</td><td>0</td><td>+</td><td>MACS_peak_2175</td><td>-24114</td></tr>
<tr><td>chr11</td><td>20127039</td><td>20149427</td><td>Cep68</td><td>0</td><td>-</td><td>MACS_peak_2175</td><td>-23709</td></tr>
<tr><td>chr11</td><td>20202182</td><td>20232716</td><td>Slc1a4</td><td>0</td><td>-</td><td>MACS_peak_2177|MACS_peak_2179|MA(</td><td>-26168|-24195|-18716|-394</td></tr>
<tr><td>chr11</td><td>20531979</td><td>20553026</td><td>Sertad2</td><td>0</td><td>+</td><td>MACS_peak_2182</td><td>-19483</td></tr>
<tr><td>chr11</td><td>21394732</td><td>21411183</td><td>4932414J0</td><td>0</td><td>-</td><td>MACS_peak_2185</td><td>-19541</td></tr>
<tr><td>chr11</td><td>23473775</td><td>23533631</td><td>0610010F(</td><td>0</td><td>-</td><td>MACS_peak_2207</td><td>-13301</td></tr>
<tr><td>chr11</td><td>29445841</td><td>29448040</td><td>Rps27a</td><td>0</td><td>-</td><td>MACS_peak_2215|MACS_peak_2215</td><td>-9313|-9313</td></tr>
<tr><td>chr11</td><td>29411756</td><td>29415033</td><td>Prdxdd1</td><td>0</td><td>-</td><td>MACS_peak_2215|MACS_peak_2215</td><td>23694|23694</td></tr>
<tr><td>chr11</td><td>29447949</td><td>29478352</td><td>1700034F(</td><td>0</td><td>+</td><td>MACS_peak_2215|MACS_peak_2215</td><td>9222|9222</td></tr>
<tr><td>chr11</td><td>29426456</td><td>29445255</td><td>Mtif2</td><td>0</td><td>+</td><td>MACS_peak_2215</td><td>-12271</td></tr>
<tr><td>chr11</td><td>29618562</td><td>29644414</td><td>Rtn4</td><td>0</td><td>+</td><td>MACS_peak_2216|MACS_peak_2216|MA(</td><td>25228|439|-437|-437|-437|-15174</td></tr>
<tr><td>chr11</td><td>30006842</td><td>30098257</td><td>Spnb2</td><td>0</td><td>-</td><td>MACS_peak_2236|MACS_peak_2236</td><td>21387|-128</td></tr>
<tr><td>chr11</td><td>30854397</td><td>31002704</td><td>Asb3</td><td>0</td><td>+</td><td>MACS_peak_2242</td><td>-119</td></tr>
<tr><td>chr11</td><td>30829783</td><td>30854131</td><td>Erlec1</td><td>0</td><td>-</td><td>MACS_peak_2242</td><td>385</td></tr>
<tr><td>chr11</td><td>31259440</td><td>31270061</td><td>Stc2</td><td>0</td><td>-</td><td>MACS_peak_2245|MACS_peak_2246</td><td>-4579|-2267</td></tr>
<tr><td>chr11</td><td>3190459</td><td>3208520</td><td>Patz1</td><td>0</td><td>+</td><td>MACS_peak_2065</td><td>-17905</td></tr>
<tr><td>chr11</td><td>3230733</td><td>3242974</td><td>Pik3ip1</td><td>0</td><td>+</td><td>MACS_peak_2065|MACS_peak_2066</td><td>22369|-143</td></tr>
<tr><td>chr11</td><td>32088996</td><td>32100279</td><td>Il9r</td><td>0</td><td>-</td><td>MACS_peak_2249|MACS_peak_2249</td><td>21942|21942</td></tr>
<tr><td>chr11</td><td>32109585</td><td>32122293</td><td>Rhbdf1</td><td>0</td><td>-</td><td>MACS_peak_2249</td><td>-72</td></tr>
<tr><td>chr11</td><td>32126504</td><td>32132701</td><td>Mpg</td><td>0</td><td>+</td><td>MACS_peak_2249</td><td>4283</td></tr>
<tr><td>chr11</td><td>32105414</td><td>32108993</td><td>Snrnp25</td><td>0</td><td>+</td><td>MACS_peak_2249</td><td>-16807</td></tr>
<tr><td>chr11</td><td>3243385</td><td>3256144</td><td>Limk2</td><td>0</td><td>-</td><td>MACS_peak_2066</td><td>-25268</td></tr>
<tr><td>chr11</td><td>32433265</td><td>32524595</td><td>Stk10</td><td>0</td><td>+</td><td>MACS_peak_2250</td><td>-21935</td></tr>
<tr><td>chr11</td><td>32422732</td><td>32427574</td><td>Efcab9</td><td>0</td><td>-</td><td>MACS_peak_2250</td><td>27626</td></tr>
<tr><td>chr11</td><td>32542874</td><td>32646814</td><td>Fbxw11</td><td>0</td><td>+</td><td>MACS_peak_2258</td><td>27026</td></tr>
<tr><td>chr11</td><td>3417525</td><td>3439295</td><td>Smtn</td><td>0</td><td>-</td><td>MACS_peak_2070|MACS_peak_2070</td><td>-12873|-12873</td></tr>
<tr><td>chr11</td><td>3414704</td><td>3417354</td><td>Selm</td><td>0</td><td>+</td><td>MACS_peak_2070</td><td>-11718</td></tr>
<tr><td>chr11</td><td>3394274</td><td>3404824</td><td>Inpp5j</td><td>0</td><td>-</td><td>MACS_peak_2070</td><td>21598</td></tr>
<tr><td>chr11</td><td>35610881</td><td>35612386</td><td>Fbll1</td><td>0</td><td>-</td><td>MACS_peak_2311|MACS_peak_2312|MA(</td><td>9603|10371|10830|16760|17765|18521|21077|22238|25616|27120|27643|28527|29840</td></tr>
<tr><td>chr11</td><td>35621883</td><td>35648030</td><td>Rars</td><td>0</td><td>-</td><td>MACS_peak_2311|MACS_peak_2312|MA(</td><td>-26041|-25273|-24814|-18884|-17879|-17123|-14567|-13406|-10028|-8524|-8001|-7117|-5804|-1199</td></tr>
<tr><td>chr11</td><td>35595897</td><td>35595983</td><td>Mir103-1</td><td>0</td><td>+</td><td>MACS_peak_2311|MACS_peak_2312|MA(</td><td>-26092|-26860|-27319</td></tr>
<tr><td>chr11</td><td>3817082</td><td>3832081</td><td>Tcn2</td><td>0</td><td>-</td><td>MACS_peak_2075|MACS_peak_2075|MA(</td><td>-12732|-12615|-12698|-8276|-8159|-8242|-4596|-4479|-4562</td></tr>
<tr><td>chr11</td><td>3786090</td><td>3795129</td><td>4921536K2</td><td>0</td><td>-</td><td>MACS_peak_2075|MACS_peak_2076</td><td>24220|28676</td></tr>
<tr><td>chr11</td><td>3807024</td><td>3814667</td><td>Slc35e4</td><td>0</td><td>-</td><td>MACS_peak_2075|MACS_peak_2076|MA(</td><td>4682|9138|12818</td></tr>
<tr><td>chr11</td><td>3795242</td><td>3801299</td><td>Dusp18</td><td>0</td><td>+</td><td>MACS_peak_2075|MACS_peak_2076</td><td>-24107|-28563</td></tr>
<tr><td>chr11</td><td>4041128</td><td>4060296</td><td>Ccdc157</td><td>0</td><td>-</td><td>MACS_peak_2079|MACS_peak_2079|MA(</td><td>-24798|-24798|-11542|-11542</td></tr>
<tr><td>chr11</td><td>4060356</td><td>4082544</td><td>Sf3a1</td><td>0</td><td>+</td><td>MACS_peak_2079|MACS_peak_2080</td><td>24858|11602</td></tr>
<tr><td>chr11</td><td>4035162</td><td>4041195</td><td>Rnf215</td><td>0</td><td>+</td><td>MACS_peak_2079|MACS_peak_2080</td><td>-336|-13592</td></tr>
<tr><td>chr11</td><td>3997042</td><td>4018732</td><td>Sec14l2</td><td>0</td><td>-</td><td>MACS_peak_2079</td><td>16766</td></tr>
<tr><td>chr11</td><td>40492815</td><td>40506115</td><td>Mat2b</td><td>0</td><td>-</td><td>MACS_peak_2326</td><td>-7332</td></tr>
<tr><td>chr11</td><td>43233751</td><td>43239750</td><td>Pttg1</td><td>0</td><td>-</td><td>MACS_peak_2327|MACS_peak_2327|MA(</td><td>11192|11459|19165|19432</td></tr>
<tr><td>chr11</td><td>43247232</td><td>43261483</td><td>Slu7</td><td>0</td><td>+</td><td>MACS_peak_2327|MACS_peak_2327|MA(</td><td>-3710|-3710|-11683|-11683</td></tr>
<tr><td>chr11</td><td>43287845</td><td>43305027</td><td>C1qtnf2</td><td>0</td><td>+</td><td>MACS_peak_2331</td><td>28930</td></tr>
<tr><td>chr11</td><td>4380870</td><td>4494818</td><td>Mtmr3</td><td>0</td><td>-</td><td>MACS_peak_2088</td><td>-144</td></tr>
<tr><td>chr11</td><td>4537795</td><td>4583388</td><td>Ascc2</td><td>0</td><td>+</td><td>MACS_peak_2089|MACS_peak_2090|MA(</td><td>-8845|-9364|-12016|-18498</td></tr>
<tr><td>chr11</td><td>4525379</td><td>4530014</td><td>Gm11961</td><td>0</td><td>-</td><td>MACS_peak_2089|MACS_peak_2089|MA(</td><td>16626|18871|17145|19390|19797|22042|26279|28524</td></tr>
<tr><td>chr11</td><td>4604680</td><td>4637669</td><td>Zmat5</td><td>0</td><td>+</td><td>MACS_peak_2099|MACS_peak_2100|MA(</td><td>25187|24552|20389</td></tr>
<tr><td>chr11</td><td>4601970</td><td>4604347</td><td>Uqcr10</td><td>0</td><td>-</td><td>MACS_peak_2099|MACS_peak_2100|MA(</td><td>-24854|-24219|-20056</td></tr>
<tr><td>chr11</td><td>45961656</td><td>45979861</td><td>Nipal4</td><td>0</td><td>-</td><td>MACS_peak_2342|MACS_peak_2343|MA(</td><td>-28946|-27467|-23380|-20809</td></tr>
<tr><td>chr11</td><td>4795345</td><td>4828868</td><td>Thoc5</td><td>0</td><td>+</td><td>MACS_peak_2102</td><td>-17475</td></tr>
<tr><td>chr11</td><td>49017001</td><td>49032318</td><td>Zfp62</td><td>0</td><td>+</td><td>MACS_peak_2381|MACS_peak_2381</td><td>-12955|-12955</td></tr>
<tr><td>chr11</td><td>49057692</td><td>49076526</td><td>Mgat1</td><td>0</td><td>+</td><td>MACS_peak_2381|MACS_peak_2381</td><td>27736|27736</td></tr>
<tr><td>chr11</td><td>48981826</td><td>49000591</td><td>Btnl9</td><td>0</td><td>-</td><td>MACS_peak_2381</td><td>29365</td></tr>
<tr><td>chr11</td><td>49715336</td><td>49793725</td><td>Rasgef1c</td><td>0</td><td>+</td><td>MACS_peak_2385</td><td>18322</td></tr>
<tr><td>chr11</td><td>49988352</td><td>50013616</td><td>3010026O(</td><td>0</td><td>+</td><td>MACS_peak_2387</td><td>22848</td></tr>
<tr><td>chr11</td><td>49944861</td><td>49986287</td><td>Tbc1d9b</td><td>0</td><td>+</td><td>MACS_peak_2387</td><td>-20643</td></tr>
</table>

chr11	50049973	50051973	Ltc4s	0	-	MACS_peak_2400|MACS_peak_2401	-7689|-6390
chr11	50013653	50024292	Sqstm1	0	-	MACS_peak_2400|MACS_peak_2401	19992|21291
chr11	50038836	50048605	Mgat4b	0	+	MACS_peak_2400|MACS_peak_2401	-5448|-6747
chr11	50069136	50105838	Maml1	0	-	MACS_peak_2403|MACS_peak_2404	3035|19357
chr11	50107458	50139175	Canx	0	-	MACS_peak_2404|MACS_peak_2404|MA	-13980|-13980|-13980
chr11	51437174	51449398	Rmnd5b	0	-	MACS_peak_2405	10198
chr11	51433274	51437216	Nhp2	0	+	MACS_peak_2405	-26322
chr11	51456590	51464575	C330016O	0	-	MACS_peak_2405|MACS_peak_2407	-4979|16476
chr11	51464455	51471182	D930048N	0	+	MACS_peak_2405|MACS_peak_2407	4859|-16596
chr11	51498886	51502136	0610009B	0	-	MACS_peak_2407|MACS_peak_2409|MA	-21085|4979|12226|18581
chr11	51505764	51570336	Sec24a	0	-	MACS_peak_2412|MACS_peak_2413	-27324|-20389
chr11	51577164	51605455	Sar1b	0	+	MACS_peak_2413|MACS_peak_2414	27217|-27961
chr11	51817722	51851667	Cdkl3	0	+	MACS_peak_2418|MACS_peak_2418|MA	3861|3861|3861|3861|3861|3861
chr11	51798648	51813968	Ube2b	0	-	MACS_peak_2418	-107
chr11	53246787	53251404	Gdf9	0	+	MACS_peak_2420	21340
chr11	53242449	53244333	Uqcrq	0	-	MACS_peak_2420	-18886
chr11	53235682	53236638	Leap2	0	-	MACS_peak_2420	-11191
chr11	53333301	53357598	08-Sep	0	+	MACS_peak_2422	-15234
chr11	53380880	53417746	Kif3a	0	+	MACS_peak_2426|MACS_peak_2427|MA	-12435|-16528|-16981|-26953
chr11	53425964	53432167	Il4	0	-	MACS_peak_2429|MACS_peak_2429	-24334|-23007
chr11	53764325	53793529	Slc22a21	0	-	MACS_peak_2432|MACS_peak_2434|MA	6560|10705|17435
chr11	53914425	53945169	P4ha2	0	+	MACS_peak_2440|MACS_peak_2440|MA	-9341|-9341|-10574|-10574|-24071|-24071|-25376|-25376|-28131|-28131|-28592|-28592
chr11	5420969	5425875	Xbp1	0	+	MACS_peak_2108	-20981
chr11	5469686	5589340	Ankrd36	0	+	MACS_peak_2108	27736
chr11	5428890	5442220	Ccdc117	0	-	MACS_peak_2108	-270
chr11	54911618	54924400	Gm2a	0	+	MACS_peak_2457	-10859
chr11	55208002	55233582	Sparc	0	-	MACS_peak_2468	-221
chr11	5613419	5662379	Urgcp	0	-	MACS_peak_2116	29488
chr11	5688485	5700983	Dbnl	0	+	MACS_peak_2116|MACS_peak_2116|MA	-3382|-3382|-3382
chr11	5701639	5703799	Pgam2	0	-	MACS_peak_2116	-11932
chr11	5662152	5684713	2210015D	0	+	MACS_peak_2116	-29715
chr11	57296493	57332146	Fam114a2	0	-	MACS_peak_2472|MACS_peak_2472|MA	-5370|-5370|-5370
chr11	57332166	57347319	Mfap3	0	+	MACS_peak_2472|MACS_peak_2472	5390|5390
chr11	57615138	57624117	Sap30l	0	+	MACS_peak_2473	19377
chr11	57997344	58003700	Gm12250	0	+	MACS_peak_2475|MACS_peak_2476	16070|11977
chr11	57933503	57982041	Gemin5	0	-	MACS_peak_2475|MACS_peak_2475|MA	-767|-767|-767|-767|3326|3326|3326|3326
chr11	57985155	57993080	Mrpl22	0	+	MACS_peak_2475|MACS_peak_2476	3881|-212
chr11	58013057	58021093	Igtp	0	+	MACS_peak_2476	27690
chr11	59120431	59147054	Wnt9a	0	+	MACS_peak_2483	-25797
chr11	59220651	59263458	Snap47	0	-	MACS_peak_2486	12559
chr11	59274698	59285976	Zfp867	0	-	MACS_peak_2486|MACS_peak_2488|MA	-9959|14206|19697
chr11	59263589	59271570	Jmjd4	0	+	MACS_peak_2486	-12428
chr11	59299022	59320142	Zkscan17	0	-	MACS_peak_2488|MACS_peak_2488|MA	-19960|-19960|-14469|-14469
chr11	59319186	59324569	4933439C	0	+	MACS_peak_2488|MACS_peak_2489	19004|13513
chr11	60012590	60034106	Srebf1	0	-	MACS_peak_2492|MACS_peak_2493|MA	21251|22162|24517
chr11	60040215	60166407	Tom1l2	0	-	MACS_peak_2496|MACS_peak_2496|MA	-81|-81|-81
chr11	60166881	60207835	Lrrc48	0	+	MACS_peak_2496	555
chr11	60541899	60546453	Smcr7	0	+	MACS_peak_2498|MACS_peak_2500|MA	23694|12186|9511|8616|8285|-4305
chr11	60513191	60527689	Llgl1	0	+	MACS_peak_2498|MACS_peak_2498|MA	-5014|-5014|-5014|-16522|-16522|-16522|-19197|-19197|-19197|-20092|-20092|-20092|-20423|-20423|-20423
chr11	60527650	60540723	Flii	0	-	MACS_peak_2498|MACS_peak_2500|MA	-22518|-11010|-8335|-7440|-7109|5481
chr11	60591026	60601789	Smcr8	0	+	MACS_peak_2508|MACS_peak_2508	18170|18170
chr11	60553560	60590867	Top3a	0	-	MACS_peak_2508	-18011
chr11	61302313	61307705	Mapk7	0	-	MACS_peak_2526	-412
chr11	61318673	61326429	B9d1	0	+	MACS_peak_2526	11380
chr11	61298945	61302206	Mfap4	0	+	MACS_peak_2526	-8348
chr11	6191599	6259096	Ogdh	0	+	MACS_peak_2141|MACS_peak_2143	17814|16842
chr11	6157547	6167732	Ddx56	0	-	MACS_peak_2141|MACS_peak_2143	6053|7025
chr11	6170716	6174840	Tmed4	0	-	MACS_peak_2141|MACS_peak_2143	-1055|-83
chr11	62271961	62327402	Pigl	0	+	MACS_peak_2540	326
chr11	62130191	62270834	Ncor1	0	-	MACS_peak_2540	801
chr11	62660624	62690964	Fbxw10	0	+	MACS_peak_2546	4857
chr11	62633754	62656450	Trim16	0	+	MACS_peak_2546	-22013
chr11	67779984	68020650	Stx8	0	+	MACS_peak_2552	-6824
chr11	67738307	67779144	Wdr16	0	-	MACS_peak_2552	7664
chr11	68634950	68666633	Ndel1	0	-	MACS_peak_2556|MACS_peak_2557	-13190|-9234
chr11	68913659	68923457	Per1	0	+	MACS_peak_2561|MACS_peak_2561|MA	-2077|-3279|-3460|-4662
chr11	68902029	68905883	Vamp2	0	+	MACS_peak_2561|MACS_peak_2563	-13707|-15090
chr11	68939878	68962616	Aloxe3	0	+	MACS_peak_2561|MACS_peak_2563	24142|22759
chr11	68933954	68936761	Hes7	0	+	MACS_peak_2561|MACS_peak_2563	18218|16835
chr11	69212019	69227177	Kdm6b	0	-	MACS_peak_2570|MACS_peak_2573	-12091|-6142
chr11	69207113	69208848	Cyb5d1	0	-	MACS_peak_2570|MACS_peak_2573	6238|12187
chr11	69210017	69211736	Tmem88	0	-	MACS_peak_2570|MACS_peak_2573	3350|9299
chr11	69209292	69210173	Lsmd1	0	+	MACS_peak_2570|MACS_peak_2573	-5794|-11743
chr11	69480437	69485925	Eif4a1	0	-	MACS_peak_2576|MACS_peak_2576|MA	-28526|-28526|-23579|-23579|-21467|-21467|-20165|-20165|-19503|-19503|-7587|-7587
chr11	69468874	69470123	Sox15	0	+	MACS_peak_2576|MACS_peak_2577|MA	11475|6528|4416|3114|2452|-9464
chr11	69477872	69479564	Cd68	0	-	MACS_peak_2576|MACS_peak_2577|MA	-22165|-17218|-15106|-13804|-13142|-1226
chr11	69428317	69431407	Shbg	0	-	MACS_peak_2576	25992
chr11	69446472	69466799	Fxr2	0	+	MACS_peak_2576|MACS_peak_2577|MA	-10927|-15874|-17986|-19288|-19950
chr11	69470205	69476144	Mpdu1	0	-	MACS_peak_2576|MACS_peak_2577|MA	-18745|-13798|-11686|-10384|-9722|2194
chr11	69435610	69437371	Sat2	0	+	MACS_peak_2576|MACS_peak_2577|MA	-21789|-26736|-28848
chr11	69448901	69449021	Mir467f	0	-	MACS_peak_2576|MACS_peak_2577|MA	8378|13325|15437|16739|17401|29317
chr11	69476544	69476627	Mir1934	0	+	MACS_peak_2576|MACS_peak_2577|MA	19145|14198|12086|10784|10122|-1794
chr11	69486613	69495586	Senp3	0	-	MACS_peak_2579|MACS_peak_2579|MA	-29826|-29712|-29164|-29050|-17248|-17134
chr11	69496078	69499056	Tnfsf13	0	-	MACS_peak_2581|MACS_peak_2581	-20718|-20718
chr11	69617098	69619126	Tmem102-	0	-	MACS_peak_2590|MACS_peak_2591|MA	-21227|-12830|-9971|-8470|-8164
chr11	69547911	69571725	Polr2a	0	-	MACS_peak_2590	26174
chr11	69597537	69609439	Chrnb1	0	-	MACS_peak_2590|MACS_peak_2591|MA	-11540|-3143|-284|1217|1523
chr11	69611703	69615127	Fgf11	0	-	MACS_peak_2590|MACS_peak_2591|MA	-17228|-8831|-5972|-4471|-4165
chr11	69573386	69575346	Amac1	0	-	MACS_peak_2590	22553
chr11	69579413	69597528	Zbtb4	0	+	MACS_peak_2590|MACS_peak_2591|MA	-18486|-26883|-29742
chr11	69617170	69619540	G630025P	0	+	MACS_peak_2590|MACS_peak_2591|MA	19271|10874|8015|6514|6208
chr11	69634372	69635667	Spem1	0	-	MACS_peak_2591|MACS_peak_2592|MA	-29371|-26512|-25011|-24705|27913
chr11	69630067	69631942	4933402P	0	-	MACS_peak_2591|MACS_peak_2592|MA	-25646|-22787|-21286|-20980
chr11	69659873	69665560	Plscr3	0	+	MACS_peak_2604|MACS_peak_2604|MA	-3707|-3462|-3435
chr11	69652026	69653060	1810027O	0	+	MACS_peak_2604	-11554
chr11	69664064	69672232	Tnk1	0	-	MACS_peak_2604	-8652
chr11	69636624	69648351	Nlgn2	0	-	MACS_peak_2604	15229
chr11	69980275	69984336	Clec10a	0	+	MACS_peak_2605	-24992
chr11	70027411	70029916	Slc16a11	0	+	MACS_peak_2605|MACS_peak_2606	22144|9089
chr11	70030293	70034496	Slc16a13	0	-	MACS_peak_2605|MACS_peak_2606	-29229|-16174
chr11	70037629	70043300	Bcl6b	0	-	MACS_peak_2606	-24978
chr11	70048218	70048301	Mir497	0	+	MACS_peak_2606	29896
chr11	70376382	70427984	Mink1	0	+	MACS_peak_2612|MACS_peak_2612|MA	-133|-133|-133|-133
chr11	70353665	70371612	Pld2	0	+	MACS_peak_2612	-22850
chr11	70429395	70430392	4930544D	0	+	MACS_peak_2614|MACS_peak_2615	10802|6994
chr11	70428384	70432696	Chrne	0	-	MACS_peak_2614|MACS_peak_2615	-14103|-10295
chr11	70482964	70501607	Camta2	0	-	MACS_peak_2617|MACS_peak_2617|MA	14356|14356|14356|14356
chr11	70514049	70545472	Kif1c	0	+	MACS_peak_2617	-1914
chr11	70501862	70513657	Inca1	0	-	MACS_peak_2617	2306
chr11	72254879	72265052	Mybbp1a	0	+	MACS_peak_2621|MACS_peak_2622|MA	-88|-345|-2446
chr11	72249027	72251906	Ggt6	0	+	MACS_peak_2621|MACS_peak_2622|MA	-5940|-6197|-8298
chr11	72203615	72225215	Smtnl2	0	-	MACS_peak_2621	29752
chr11	72503503	72585648	Ankfy1	0	+	MACS_peak_2625	16989
chr11	74644425	74659227	Mnt	0	+	MACS_peak_2633|MACS_peak_2634|MA	-6743|-11750|-22921
chr11	74711581	74722842	Tsr1	0	+	MACS_peak_2640	29433
chr11	74662765	74710582	Sgsm2	0	-	MACS_peak_2640	-28434
chr11	75281580	75284146	Tlcd2	0	+	MACS_peak_2649|MACS_peak_2650|MA	-2446|-2624|-25845|-27248|-27626|-28369
chr11	75300278	75322949	Prpf8	0	+	MACS_peak_2649|MACS_peak_2650|MA	16252|16074|-7147|-8550|-8928|-9671|-13158|-13804|-14073|-14353|-16610|-16904|-17757|-17976|-18413|-19679|-19957|-21567|-21812
chr11	75254445	75268219	Wdr81	0	-	MACS_peak_2649|MACS_peak_2650	15807|15985
chr11	75277217	75277312	Mir22	0	+	MACS_peak_2649|MACS_peak_2650	-6809|-6987
chr11	75275040	75280192	2210403K	0	+	MACS_peak_2649|MACS_peak_2650	-8986|-9164
chr11	75327042	75340082	Scarf1	0	+	MACS_peak_2651|MACS_peak_2652|MA	19617|18214|17836|17093|13606|12960|12691|12411|10154|9860|9007|8788|8351|7085|6807|5197|4952|4673

chr11	75323595	75326668	Rilp	0	+	MACS_peak_2651|MACS_peak_2652|MA	16170|14767|14389|13646|10159|9513|9244|8964|6707|6413|5560|5341|4904|3638|3360|1750|1505|1226
chr11	75345211	75388543	Slc43a2	0	+	MACS_peak_2659|MACS_peak_2660|MA	28323|28029|27176|26957|26520|25254|24976|23366|23121|22842
chr11	75465651	75488136	Myo1c	0	+	MACS_peak_2669|MACS_peak_2669|MA	15148|19013|14507|-16335|-12470|-16976
chr11	75444521	75462367	Inpp5k	0	+	MACS_peak_2669	-5982
chr11	75492811	75519594	Crk	0	+	MACS_peak_2672	10825
chr11	75832700	75841284	Fam101b	0	-	MACS_peak_2673	-6219
chr11	75813413	75817071	1700016K	0	+	MACS_peak_2673	-21652
chr11	76891565	76891674	Mir423	0	-	MACS_peak_2679	-29772
chr11	77277414	77283003	Coro6	0	+	MACS_peak_2681|MACS_peak_2681|MA	13771|13771|13771
chr11	77342663	77421317	Taok1	0	-	MACS_peak_2684	-22459
chr11	77796317	77844035	Phf12	0	+	MACS_peak_2688	-1297
chr11	78006893	78019265	Proca1	0	+	MACS_peak_2689|MACS_peak_2690	-15554|-19393
chr11	78001928	78005695	Rab34	0	+	MACS_peak_2689|MACS_peak_2689|MA	-20519|-20198|-24358|-24037
chr11	77994436	77997086	Rpl23a	0	-	MACS_peak_2689|MACS_peak_2690	25361|29200
chr11	77995188	77995258	Snord4a	0	-	MACS_peak_2689	27189
chr11	78059247	78068997	Sdf2	0	+	MACS_peak_2692|MACS_peak_2693|MA	20341|20008|18782|14681|13629
chr11	78020250	78059205	Supt6h	0	-	MACS_peak_2692|MACS_peak_2693|MA	-20299|-19966|-18740|-14639|-13587
chr11	78075255	78104128	2610507B	0	+	MACS_peak_2698	29637
chr11	78137699	78140262	Aldoc	0	+	MACS_peak_2703	-737
chr11	78157023	78162658	Unc119	0	+	MACS_peak_2703	18587
chr11	78115092	78135955	Spag5	0	+	MACS_peak_2703	-23344
chr11	78141923	78156278	Pigs	0	+	MACS_peak_2703	3487
chr11	78380669	78510927	Nlk	0	-	MACS_peak_2711	-1467
chr11	79314483	79317584	Omg	0	+	MACS_peak_2716	-28247
chr11	80242116	80285635	Psmd11	0	+	MACS_peak_2722	4421
chr11	83116198	83218535	Ap2b1	0	+	MACS_peak_2727|MACS_peak_2727|MA	4236|4236|-13777|-13777
chr11	83088613	83100228	Slfn14-ps	0	-	MACS_peak_2727|MACS_peak_2728	11734|29747
chr11	83108146	83112479	Pex12	0	-	MACS_peak_2727|MACS_peak_2728	-517|17496
chr11	83107805	83107901	Snord7	0	+	MACS_peak_2727|MACS_peak_2728	-4157|-22170
chr11	83105200	83108138	AA465934	0	+	MACS_peak_2727|MACS_peak_2728	-6762|-24775
chr11	83106931	83108431	AI450353	0	+	MACS_peak_2727|MACS_peak_2728	-5031|-23044
chr11	83235403	83242957	Gas2l2	0	-	MACS_peak_2736	14380
chr11	83251195	83254734	1700020L	0	+	MACS_peak_2736|MACS_peak_2739	-6142|-25209
chr11	83286609	83320242	Taf15	0	+	MACS_peak_2736|MACS_peak_2739	29272|10205
chr11	83255377	83276463	Mmp28	0	-	MACS_peak_2736|MACS_peak_2736|MA	-19126|-19126|-59|-59
chr11	84689814	84693787	Pigw	0	-	MACS_peak_2745|MACS_peak_2745	-10253|-10163
chr11	84693721	84724633	Myo19	0	+	MACS_peak_2745	10187
chr11	84645862	84684240	Ggnbp2	0	-	MACS_peak_2745	-706
chr11	85004811	85048622	Appbp2	0	-	MACS_peak_2747	-18639
chr11	85048667	85051805	D630032N	0	+	MACS_peak_2747	18684
chr11	85871639	86014695	Brip1	0	-	MACS_peak_2755|MACS_peak_2757	267|28049
chr11	86024184	86071070	Ints2	0	-	MACS_peak_2757|MACS_peak_2758	-28326|-25531
chr11	86079216	86171027	Med13	0	-	MACS_peak_2761	671
chr11	86397568	86397660	Mir21	0	-	MACS_peak_2764	23075
chr11	86497582	86505959	Ptrh2	0	+	MACS_peak_2768|MACS_peak_2768|MA	79|-19|-18222|-18320|-18605|-18703|-19854|-19952
chr11	86397366	86497324	Tmem49	0	-	MACS_peak_2768|MACS_peak_2770|MA	179|18480|18863|20112
chr11	86847295	86887639	Gdpd1	0	-	MACS_peak_2789|MACS_peak_2791	-28324|-164
chr11	86891233	86900279	1200011M	0	-	MACS_peak_2791	-12804
chr11	87861172	87867259	Srsf1	0	+	MACS_peak_2792|MACS_peak_2792	-20701|-20701
chr11	87881842	87898231	Vezf1	0	+	MACS_peak_2792	-31
chr11	88692105	88725900	Akap1	0	-	MACS_peak_2799|MACS_peak_2799	-23346|-10263
chr11	88785335	88816756	Scpep1	0	-	MACS_peak_2806|MACS_peak_2808|MA	-20818|-14123|-8270|-83
chr11	88825980	88828231	Gm15698	0	-	MACS_peak_2808|MACS_peak_2811|MA	-25598|-19745|-11558
chr11	88835248	88852927	Coil	0	+	MACS_peak_2811|MACS_peak_2813	26762|18575
chr11	88833951	88834328	2210409E	0	+	MACS_peak_2811|MACS_peak_2813	25465|17278
chr11	9018010	9036172	Upp1	0	+	MACS_peak_2156|MACS_peak_2156|MA	-101|398|-6|-600|-101|-505|-11538|-11039|-11443|-13699|-13200|-13604|-15259|-14760|-15164|-16794|-16295|-16699|-17225|-16726|-17
chr11	93720556	93747080	Utp18	0	-	MACS_peak_2819	23109
chr11	93747532	93808298	Mbtd1	0	+	MACS_peak_2819	-22657
chr11	93811130	93817002	Nme2	0	-	MACS_peak_2820|MACS_peak_2820	-11382|-11701
chr11	93820238	93829835	Nme1	0	-	MACS_peak_2820	-24215
chr11	93905518	93987396	Spag9	0	+	MACS_peak_2823|MACS_peak_2823|MA	-24785|-24785|-24785
chr11	94887572	94902685	Pdk2	0	-	MACS_peak_2831|MACS_peak_2832|MA	5171|5611|12451|12783|14378|14524|15408|15756|17995|18254|18502|20624|21806|26938|27374
chr11	94905795	94938028	Itga3	0	-	MACS_peak_2832|MACS_peak_2834|MA	-29732|-22892|-22560|-20965|-20819|-19935|-19587|-17348|-17089|-16841|-14719|-13537|-8405|-7969|-243
chr11	95135572	95171515	Myst2	0	-	MACS_peak_2853	-18612
chr11	95246235	95252966	Slc35b1	0	+	MACS_peak_2854	4215
chr11	96678727	96690816	Nfe2l1	0	-	MACS_peak_2861|MACS_peak_2861|MA	373|5748|5748|5862|5862|-93
chr11	96711189	96722516	Copz2	0	+	MACS_peak_2861	20000
chr11	96711706	96711779	Mir152	0	+	MACS_peak_2861	20517
chr11	96912133	96930218	Osbpl7	0	+	MACS_peak_2864	17078
chr11	96902899	96910527	Mrpl10	0	+	MACS_peak_2864	7844
chr11	96895915	96902683	Lrrc46	0	-	MACS_peak_2864	-7628
chr11	96874882	96886052	Sp6	0	+	MACS_peak_2864	-20173
chr11	96891265	96895272	Scrn2	0	+	MACS_peak_2864	-3790
chr11	97311473	97363714	Arhgap23	0	+	MACS_peak_2865|MACS_peak_2866	-1593|-4102
chr11	97524725	97546772	Mllt6	0	+	MACS_peak_2867|MACS_peak_2869	18767|-28474
chr11	97488700	97491030	E130012A	0	-	MACS_peak_2867	14928
chr11	97547265	97549939	Cisd3	0	+	MACS_peak_2869	-5934
chr11	97550136	97560698	Pcgf2	0	-	MACS_peak_2869|MACS_peak_2869|MA	-7499|-7763|-8612
chr11	97564747	97574814	Psmb3	0	+	MACS_peak_2869	11548
chr11	97606784	97627927	Cwc25	0	-	MACS_peak_2870	-20495
chr11	97632794	97637232	1700001P	0	-	MACS_peak_2870	-29800
chr11	97576470	97606018	Pip4k2b	0	-	MACS_peak_2870	1414
chr11	98273797	98299030	Erbb2	0	+	MACS_peak_2901|MACS_peak_2902|MA	-21500|-22392|-23571
chr11	98308147	98316687	Grb7	0	+	MACS_peak_2901|MACS_peak_2902|MA	12850|11958|10779
chr11	98299021	98300302	1810046J1	0	-	MACS_peak_2901|MACS_peak_2902|MA	-5005|-4113|-2934
chr11	98442607	98448559	Ormdl3	0	-	MACS_peak_2906|MACS_peak_2907	-3211|-54
chr11	98629248	98636556	Nr1d1	0	-	MACS_peak_2910	-12246
chr11	98603186	98626425	Thra	0	+	MACS_peak_2910	-21124
chr11	98698098	98714319	Rapgefl1	0	+	MACS_peak_2919	9182
chr11	98671121	98695121	Casc3	0	+	MACS_peak_2919	-17795
chr11	98659622	98671403	Gm12359	0	-	MACS_peak_2919	17513
chr11	98769202	98785256	Cdc6	0	+	MACS_peak_2920|MACS_peak_2920|MA	16323|16585|15452|15714|7820|8082
chr11	98724911	98766892	Wipf2	0	+	MACS_peak_2920|MACS_peak_2921	-27968|-28839
chr11	98902573	98913957	Igfbp4	0	+	MACS_peak_2927|MACS_peak_2928	-25575|-25954
chr11	98926992	98950620	Tns4	0	-	MACS_peak_2927|MACS_peak_2928	-22472|-22093
chr12	101538979	101759032	Ttc7b	0	-	MACS_peak_3903	29322
chr12	102984774	103057265	Fbln5	0	-	MACS_peak_3914	18516
chr12	103075582	103151381	Trip11	0	-	MACS_peak_3915|MACS_peak_3916	7695|19852
chr12	103171133	103196453	Atxn3	0	-	MACS_peak_3916|MACS_peak_3916	-25220|-25220
chr12	103214183	103244203	Cpsf2	0	+	MACS_peak_3917	-29066
chr12	103708119	103736117	Golga5	0	+	MACS_peak_3929	-2374
chr12	103793178	103803237	Chga	0	+	MACS_peak_3930	-24174
chr12	104646185	104663990	Ddx24	0	-	MACS_peak_3931|MACS_peak_3931	-20102|-20189
chr12	104626891	104644560	Otub2	0	+	MACS_peak_3931|MACS_peak_3931|MA	-16997|-16998|-28785
chr12	104672398	104678455	Ifi27l1	0	+	MACS_peak_3931|MACS_peak_3931|MA	28510|28510|28510|28510|28510
chr12	106842300	106843638	Bdkrb1	0	+	MACS_peak_3944|MACS_peak_3945	-18081|-22187
chr12	106851748	106923451	Atg2b	0	-	MACS_peak_3949|MACS_peak_3951|MA	-27183|-22589|-18558
chr12	106923561	106941267	4933433P	0	+	MACS_peak_3949|MACS_peak_3951|MA	27293|22699|18668
chr12	110031520	110054842	Yy1	0	+	MACS_peak_3958|MACS_peak_3959	-23553|-25885
chr12	110064087	110074086	Slc25a29	0	-	MACS_peak_3958|MACS_peak_3959|MA	-19013|-16681|-8573|-140|24855|27017
chr12	110075182	110075278	Mir345	0	+	MACS_peak_3958|MACS_peak_3959|MA	20109|17777|9669|1236|-23759|-25921|-29183
chr12	110089338	110095024	Slc25a47	0	+	MACS_peak_3961|MACS_peak_3962|MA	23825|15392|-9603|-11765|-15027|-16596|-24076|-24560
chr12	110098239	110132384	Wars	0	-	MACS_peak_3965|MACS_peak_3965|MA	-28019|-27043|-27056|-26450|-25474|-25487|-18970|-17994|-18007|-18486|-17510|-17523|-11378|-10402|-10415|-5717|-4741|-4754|-907
chr12	110132481	110266660	Wdr25	0	+	MACS_peak_3965|MACS_peak_3966|MA	28116|26547|19067|18583|11475|5814|1004
chr12	111839604	111905154	Dync1h1	0	+	MACS_peak_3974|MACS_peak_3975|MA	-278|-12735|-15414|-17885|-20206|-24084|-24388|-24583|-25584|-26893|-27384|-27614|-27955|-28514|-29182|-29451
chr12	111829583	111830889	B930059L	0	+	MACS_peak_3974|MACS_peak_3975|MA	-10299|-22756|-25435|-27906
chr12	111905898	111920829	1700001K	0	-	MACS_peak_4021|MACS_peak_4022|MA	-28422|-27781|-27100|-26229|-25499|-24705|-24466|-24151|-23880|-23677|-23447|-23142|-21675|-21411|-20798|-20065|-19664|-19396|-
chr12	112680871	112693229	Tnfaip2	0	+	MACS_peak_4051|MACS_peak_4052|MA	-5657|-7165|-8505
chr12	113052480	113066287	Zfyve21	0	+	MACS_peak_4059	18657
chr12	113041403	113052052	Xrcc3	0	-	MACS_peak_4059	-18229
chr12	113858257	113879566	Adssl1	0	+	MACS_peak_4071|MACS_peak_4075	19853|-13053
chr12	113826994	113853768	Inf2	0	+	MACS_peak_4071	-11410

chr12	113883038	113887363	Siva1	0	+	MACS_peak_4075|MACS_peak_4075	11728|11728
chr12	113960384	113984802	AW555464	0	+	MACS_peak_4081|MACS_peak_4082|MA	-13414|-16491|-23446
chr12	113998865	114007197	Pld4	0	+	MACS_peak_4081|MACS_peak_4082|MA	25067|21990|15035
chr12	114058445	114067600	Cdca4	0	-	MACS_peak_4085	-17137
chr12	114047185	114054456	BC022687	0	+	MACS_peak_4085	-3278
chr12	114146800	114167706	Jag2	0	-	MACS_peak_4088|MACS_peak_4089|MA	-19875|-17750|-16921|-13831|-13107|-12847
chr12	114172943	114180329	Nudt14	0	-	MACS_peak_4090|MACS_peak_4091|MA	-29544|-26454|-25730|-25470
chr12	117445522	117501498	Wdr60	0	-	MACS_peak_4096	-21294
chr12	13275932	13590616	Nbas	0	+	MACS_peak_3368	-16121
chr12	14154403	14158844	Fam84a	0	-	MACS_peak_3395	-3660
chr12	16542474	16596576	Lpin1	0	-	MACS_peak_3405|MACS_peak_3405|MA	-25192|-25192|-25192
chr12	16999766	17006924	Pqlc3	0	-	MACS_peak_3409|MACS_peak_3409|MA	-2439|-2439|-197|-197
chr12	21275666	21292098	Itgb1bp1	0	-	MACS_peak_3436|MACS_peak_3437|MA	-14134|8887|22715
chr12	21292157	21320917	Cpsf3	0	+	MACS_peak_3436|MACS_peak_3437|MA	14193|-8828|-22656
chr12	21322252	21329468	Iah1	0	+	MACS_peak_3437|MACS_peak_3438	21267|7439
chr12	25183445	25243436	Taf1b	0	+	MACS_peak_3463|MACS_peak_3463	-10543|-10543
chr12	25350702	25366660	Cys1	0	-	MACS_peak_3466|MACS_peak_3466|MA	-26620|-26620|-26620
chr12	25336235	25347645	Klf11	0	+	MACS_peak_3466	-3805
chr12	25659796	25744562	Kidins220	0	+	MACS_peak_3468|MACS_peak_3469|MA	-14844|-15806|-28130
chr12	29334466	29344454	Rnaseh1	0	+	MACS_peak_3477|MACS_peak_3478|MA	15320|-8041|-25699
chr12	29315711	29320818	Rps7	0	-	MACS_peak_3477|MACS_peak_3478	-1672|21689
chr12	29279037	29308155	Colec11	0	-	MACS_peak_3477	10991
chr12	29360071	29367039	Adi1	0	+	MACS_peak_3478|MACS_peak_3479	17564|-94
chr12	31758832	31764805	Fam110c	0	+	MACS_peak_3488	-10745
chr12	31950158	32014504	Lamb1-1	0	+	MACS_peak_3490|MACS_peak_3492|MA	-1484|-7175|-13474|-24093
chr12	32171763	32184405	Cbll1	0	-	MACS_peak_3523|MACS_peak_3524	-7406|-4916
chr12	32339733	32622495	Cog5	0	+	MACS_peak_3525	24020
chr12	32280218	32319523	Bcap29	0	-	MACS_peak_3525|MACS_peak_3525	-3810|-3749
chr12	32324919	32339691	Dus4l	0	-	MACS_peak_3525	-23978
chr12	3247429	3309969	Rab10	0	-	MACS_peak_3335	-341
chr12	3365131	3406494	Kif3c	0	+	MACS_peak_3336|MACS_peak_3337	-1389|-25366
chr12	3426883	3506849	Asxl2	0	+	MACS_peak_3338	23485
chr12	3403882	3426747	1110002L	0	-	MACS_peak_3338	-23349
chr12	36818421	36883412	Bzw2	0	-	MACS_peak_3543	13702
chr12	36883710	36923878	Ankmy2	0	+	MACS_peak_3543	-13404
chr12	3806979	3914443	Dnmt3a	0	+	MACS_peak_3340	24670
chr12	40902972	40925902	Gm889	0	-	MACS_peak_3553|MACS_peak_3554	14710|17871
chr12	40929716	40949776	Ifrd1	0	-	MACS_peak_3553|MACS_peak_3554	-9164|-6003
chr12	40949299	40949398	Mir1938	0	-	MACS_peak_3553|MACS_peak_3554	-8786|-5625
chr12	4211671	4234294	Cenpo	0	-	MACS_peak_3341|MACS_peak_3342	13384|25005
chr12	45370140	45414422	Pnpla8	0	+	MACS_peak_3561	-13986
chr12	4869243	4881165	Mfsd2b	0	-	MACS_peak_3349	20227
chr12	4924158	5054216	Atad2b	0	+	MACS_peak_3349	22766
chr12	4885837	4914326	Ubxn2a	0	-	MACS_peak_3349	-12934
chr12	56331464	56364007	Srp54c	0	+	MACS_peak_3578|MACS_peak_3579	-16787|-21691
chr12	60059370	60113004	Sec23a	0	-	MACS_peak_3596|MACS_peak_3597|MA	-29394|-22606|-14810|-10081|-6731
chr12	60114379	60128975	Sip1	0	+	MACS_peak_3597|MACS_peak_3598|MA	23981|16185|11456|8106
chr12	60232439	60291207	Ctage5	0	+	MACS_peak_3602|MACS_peak_3602|MA	1650|-57|-57|-15451|-17158|-17158|-18059|-19766|-19766|-26858|-28565|-28565
chr12	66018897	66025578	Gm527	0	+	MACS_peak_3616	-22026
chr12	66043426	66066523	Klhl28	0	-	MACS_peak_3616	-25600
chr12	66066728	66123559	Fam179b	0	+	MACS_peak_3616	25805
chr12	70397667	70411674	Klhdc2	0	+	MACS_peak_3621|MACS_peak_3622	12565|-8988
chr12	72116953	72200338	Arid4a	0	+	MACS_peak_3633	-23929
chr12	72212414	72224208	Tomm20l	0	+	MACS_peak_3638	23887
chr12	73187109	73201829	Jkamp	0	+	MACS_peak_3652|MACS_peak_3653	12199|6556
chr12	73174414	73186300	2810055F1	0	-	MACS_peak_3652|MACS_peak_3653	-11390|-5747
chr12	73170604	73171978	Gpr135	0	-	MACS_peak_3652|MACS_peak_3653	2932|8575
chr12	73637343	73681200	1810048J1	0	+	MACS_peak_3656	-20502
chr12	73751340	73765815	Dhrs7	0	-	MACS_peak_3664	-123
chr12	74387841	74453915	Slc38a6	0	+	MACS_peak_3669	14223
chr12	74381396	74387698	Trmt5	0	-	MACS_peak_3669	-14080
chr12	76512706	76517768	Gphb5	0	-	MACS_peak_3670	-25016
chr12	77505162	77507923	Hspa2	0	+	MACS_peak_3697|MACS_peak_3697	-600|-406
chr12	77518585	77540478	Gm70	0	+	MACS_peak_3697	12823
chr12	77634546	77680026	Plekhg3	0	+	MACS_peak_3699	-165
chr12	78340007	78576974	Fut8	0	+	MACS_peak_3705|MACS_peak_3706	925|25
chr12	79327641	79785754	Gphn	0	+	MACS_peak_3717|MACS_peak_3717	468|468
chr12	79989683	80007925	Plek2	0	-	MACS_peak_3723	-12819
chr12	80257004	80273445	Vti1b	0	-	MACS_peak_3724	2847
chr12	80276537	80292806	Rdh11	0	-	MACS_peak_3724	-16514
chr12	80333333	80397269	Zfyve26	0	-	MACS_peak_3725	-15745
chr12	80398268	80915677	Rad51l1	0	+	MACS_peak_3725	16744
chr12	81492601	81492724	Scarna3b	0	-	MACS_peak_3746|MACS_peak_3747	-15052|12897
chr12	81564081	81599122	Exd2	0	+	MACS_peak_3749|MACS_peak_3750	-21211|-29615
chr12	81619976	81704883	Galntl1	0	+	MACS_peak_3750|MACS_peak_3751	26280|21982
chr12	82571690	82586097	Cox16	0	-	MACS_peak_3757	14813
chr12	8320238	8350630	Hs1bp3	0	+	MACS_peak_3353	-22885
chr12	84866998	84882940	Dcaf4	0	+	MACS_peak_3777	-29162
chr12	85350451	85358619	Acot1	0	+	MACS_peak_3785	18910
chr12	85328810	85334823	Acot2	0	+	MACS_peak_3785	-2731
chr12	85487080	85489439	Pnma1	0	-	MACS_peak_3791	13897
chr12	85658079	85684674	Zfp410	0	+	MACS_peak_3792	-12756
chr12	86311879	86324246	Fcf1	0	+	MACS_peak_3807	-11354
chr12	86259098	86311836	1110018G	0	-	MACS_peak_3807	11397
chr12	86337270	86411465	Ylpm1	0	+	MACS_peak_3807	14037
chr12	86507590	86518235	Pgf	0	-	MACS_peak_3812	-5527
chr12	86476545	86492214	Rps6kl1	0	-	MACS_peak_3812	20494
chr12	86613347	86621385	Acyp1	0	-	MACS_peak_3818	27516
chr12	86629540	86640308	Fam164c	0	+	MACS_peak_3818|MACS_peak_3820	-19361|-21876
chr12	86640463	86680312	Nek9	0	-	MACS_peak_3820	-28896
chr12	88438480	88443489	Ngb	0	-	MACS_peak_3838	5256
chr12	88985075	88985570	Gm16381	0	-	MACS_peak_3842	-22980
chr12	88959858	88963093	Gm4027	0	+	MACS_peak_3842	-2732
chr12	88959817	88963905	BB287469	0	+	MACS_peak_3842	-2773
chr12	88943637	88949795	Oog1	0	+	MACS_peak_3842	-18953
chr12	92871449	93024876	Ston2	0	-	MACS_peak_3873	27475
chr12	93044482	93087597	Sel1l	0	-	MACS_peak_3880|MACS_peak_3880|MA	-29080|-29080|-25595|-25595|-24262|-24262|-23821|-23821|-22622|-22622|-17492|-17492|-7416|-7416|-116|-116
chr12	99914950	99975615	Ptpn21	0	-	MACS_peak_3896|MACS_peak_3896	-28568|-25808
chr13	100718486	100785594	Mccc2	0	-	MACS_peak_4715	-25326
chr13	100787948	100874025	Bdp1	0	-	MACS_peak_4719	5006
chr13	100877973	100884188	Serf1	0	+	MACS_peak_4719	-1058
chr13	100894809	100907653	Smn1	0	+	MACS_peak_4719	15778
chr13	101365911	101386926	Marveld2	0	-	MACS_peak_4720|MACS_peak_4720	-26713|-26686
chr13	101505894	101514614	Mrps36	0	-	MACS_peak_4721|MACS_peak_4721	-24035|-24035
chr13	101466979	101500897	Cdk7	0	-	MACS_peak_4721	-10318
chr13	104932232	104968546	2410002O	0	-	MACS_peak_4734|MACS_peak_4734|MA	-4144|-4144|-4144|-4144
chr13	104969177	104992128	Trim23	0	+	MACS_peak_4734	4775
chr13	105421406	105607033	Cwc27	0	-	MACS_peak_4736|MACS_peak_4737	-14787|338
chr13	105607318	105628739	Srsf12ip1	0	+	MACS_peak_4736|MACS_peak_4737	15072|-53
chr13	111185251	111191051	Plk2	0	+	MACS_peak_4776|MACS_peak_4781|MA	-1905|-3695|-4073
chr13	113657987	113717588	Skiv2l2	0	-	MACS_peak_4798	-6987
chr13	113718000	113759395	Dhx29	0	+	MACS_peak_4798	7399
chr13	113832970	113836596	Gpx8	0	-	MACS_peak_4800	-825
chr13	113827741	113827831	Mir449a	0	+	MACS_peak_4800	-8030
chr13	113826190	113826299	Mir449c	0	+	MACS_peak_4800	-9581
chr13	113827626	113827706	Mir449b	0	+	MACS_peak_4800	-8145
chr13	115242469	115248938	Fst	0	-	MACS_peak_4811	-2300
chr13	117643630	117814323	Parp8	0	-	MACS_peak_4819	-277
chr13	120276845	120284312	Gm7120	0	+	MACS_peak_4826|MACS_peak_4826|MA	13170|13991|12074|12895
chr13	120251565	120274924	4833420G	0	+	MACS_peak_4826|MACS_peak_4826|MA	-12110|-12110|-13206|-13206
chr13	120276065	120277191	3110070M	0	-	MACS_peak_4826|MACS_peak_4827	-13516|-12420
chr13	12487641	12531160	Heatr1	0	+	MACS_peak_4152	-12194

chr13	12531685	12553757	Lgals8	0	-	MACS_peak_4156	-8733
chr13	12658149	12701795	Ero1lb	0	+	MACS_peak_4157	-15824
chr13	12706332	12742662	Gpr137b-p	0	-	MACS_peak_4169	-19683
chr13	13275048	13284107	Prl2c5	0	+	MACS_peak_4179	-7911
chr13	13529868	13604542	Nid1	0	+	MACS_peak_4195|MACS_peak_4196|MA	-163|-26265|-27821
chr13	14046940	14091335	B3galnt2	0	+	MACS_peak_4229|MACS_peak_4231	-11711|-25974
chr13	14090217	14131867	Tbce	0	-	MACS_peak_4239	-20989
chr13	16106307	16119044	Inhba	0	+	MACS_peak_4255	-13567
chr13	21454688	21464171	Zscan12	0	+	MACS_peak_4261	-6782
chr13	21438798	21439749	Olfr1367	0	+	MACS_peak_4261	-22672
chr13	24843713	24853806	Gmnn	0	-	MACS_peak_4285	-29201
chr13	24923527	24934021	Tdp2	0	+	MACS_peak_4289	21746
chr13	24893525	24904768	BC005537	0	+	MACS_peak_4289	-8256
chr13	24909823	24923358	Acot13	0	-	MACS_peak_4289	-21577
chr13	27941270	27949577	Prl2c1	0	+	MACS_peak_4296	-7482
chr13	31038712	31039543	Hus1b	0	-	MACS_peak_4306	-12113
chr13	31650038	31652843	Foxq1	0	+	MACS_peak_4309	281
chr13	31657360	31674382	1700018A	0	-	MACS_peak_4309	-24625
chr13	33924213	33935277	Serpinb6e	0	-	MACS_peak_4317	25139
chr13	34009788	34027952	Serpinb6a	0	-	MACS_peak_4319|MACS_peak_4319|MA	-17279|-17279|-17279|17353|17353|17353
chr13	33971684	33997577	Serpinb6c	0	-	MACS_peak_4319	13096
chr13	34056527	34080334	Nqo2	0	+	MACS_peak_4321|MACS_peak_4321|MA	11222|11222|13716|11222
chr13	34254845	34270041	Psmg4	0	+	MACS_peak_4324|MACS_peak_4324	-18274|-18274
chr13	34719709	34744550	1300014I0	0	-	MACS_peak_4330	-24309
chr13	37980739	38043871	Rreb1	0	+	MACS_peak_4334|MACS_peak_4334	-27664|-28101
chr13	38129163	38153298	Riok1	0	+	MACS_peak_4337	14384
chr13	38063269	38086059	Ssr1	0	-	MACS_peak_4337	28720
chr13	38097920	38128806	Cage1	0	-	MACS_peak_4337	-14027
chr13	40811043	40825812	Tcfap2a	0	-	MACS_peak_4344|MACS_peak_4344|MA	-9121|-12501|-7207|-10587
chr13	41746130	41752146	Gm5082	0	+	MACS_peak_4352|MACS_peak_4352	12282|10963
chr13	45650261	46060345	Atxn1	0	-	MACS_peak_4369	-72
chr13	47118911	47138586	Tpmt	0	-	MACS_peak_4375	19846
chr13	47138907	47179982	Kdm1b	0	+	MACS_peak_4375	-19525
chr13	48356795	48359405	Id4	0	+	MACS_peak_4377	-115
chr13	49777498	49829636	Iars	0	+	MACS_peak_4391	-6106
chr13	50513245	50529138	Fbxw17	0	+	MACS_peak_4397	29406
chr13	54605165	54652651	4732471D	0	+	MACS_peak_4414	-21551
chr13	54676373	54682488	Arl10	0	+	MACS_peak_4415	24427
chr13	54652579	54666743	4833439L1	0	-	MACS_peak_4415	-14797
chr13	54802823	54838022	Cdhr2	0	+	MACS_peak_4417|MACS_peak_4418|MA	20834|19299|13840
chr13	54780761	54789169	Rnf44	0	-	MACS_peak_4417|MACS_peak_4417|MA	-7180|-7180|-7572|-7180|-13332|-13332|-5645|-5645|-6037|-5645|-11797|-11797|-186|-186|-578|-186|-6338|-6338
chr13	55565627	55574516	Prr7	0	+	MACS_peak_4431|MACS_peak_4432|MA	-13359|-13539|-17346|-17693|-17953|-18183|-18488
chr13	55574788	55589437	Dbn1	0	-	MACS_peak_4431|MACS_peak_4431|MA	-10451|-10451|-10451|-10271|-10271|-10271|-6464|-6464|-6464|-6117|-6117|-6117|-5857|-5857|-5857|-5627|-5627|-5627|-5322|-5322|-5
chr13	55640676	55671336	Fam193b	0	-	MACS_peak_4439|MACS_peak_4439|MA	-15688|-16833|-154|-1299
chr13	55624595	55629899	Dok3	0	-	MACS_peak_4439	25749
chr13	55631770	55638019	Ddx41	0	-	MACS_peak_4439	17629
chr13	55694495	55699055	Tmed9	0	+	MACS_peak_4440|MACS_peak_4442	23313|-11071
chr13	55724365	55733777	Caml	0	+	MACS_peak_4442	18799
chr13	55701471	55711315	B4galt7	0	+	MACS_peak_4442	-4095
chr13	58259009	58266054	5133401N	0	+	MACS_peak_4446|MACS_peak_4446|MA	-19460|-19460|-23211|-23211
chr13	58277516	58317014	Ubqln1	0	-	MACS_peak_4448|MACS_peak_4448	26038|26038
chr13	5860734	5869639	Klf6	0	+	MACS_peak_4125	-5212
chr13	59856775	59871150	Isca1	0	-	MACS_peak_4451	27922
chr13	59873239	59924508	Zcchc6	0	-	MACS_peak_4451|MACS_peak_4454	-25436|-7514
chr13	59871326	59875041	Etohd2	0	+	MACS_peak_4451	-27746
chr13	63612840	63666828	Ptch1	0	-	MACS_peak_4486	-23281
chr13	64393143	64414018	1110018J1	0	-	MACS_peak_4489	-455
chr13	64533860	64541029	Cdk20	0	+	MACS_peak_4499|MACS_peak_4501	89|-26037
chr13	65380161	65399103	Zfp369	0	+	MACS_peak_4504|MACS_peak_4505|MA	-13236|-17575|-21652|-23611
chr13	6579119	6647970	Pfkp	0	-	MACS_peak_4130|MACS_peak_4131	-26724|-76
chr13	67006350	67010102	Gm10767	0	+	MACS_peak_4532	-6886
chr13	67033765	67099337	Ptdss1	0	+	MACS_peak_4532	20529
chr13	67013033	67034008	Mterfd1	0	-	MACS_peak_4532	-20772
chr13	67001556	67006286	Uqcrb	0	-	MACS_peak_4532	6950
chr13	67139531	67162106	Zfp712	0	-	MACS_peak_4533	-19475
chr13	67541367	67552560	Zfp874	0	-	MACS_peak_4534|MACS_peak_4535|MA	-2209|20129|20328|23293
chr13	67506657	67522354	Zfp459	0	-	MACS_peak_4534	27997
chr13	67591102	67601458	Zfp58	0	-	MACS_peak_4535|MACS_peak_4536|MA	-28769|-28570|-25605
chr13	67572448	67585189	9630025I2	0	-	MACS_peak_4535|MACS_peak_4536|MA	-12500|-12301|-9336
chr13	67717937	67738690	A530054K	0	-	MACS_peak_4538|MACS_peak_4539	-17517|-14237
chr13	68736315	68753108	1700001L1	0	+	MACS_peak_4591	22216
chr13	68721124	68731156	Fastkd3	0	+	MACS_peak_4591	7025
chr13	68699656	68720998	Mtrr	0	-	MACS_peak_4591	-6899
chr13	69636837	69672742	Papd7	0	-	MACS_peak_4592|MACS_peak_4592	-18052|-18052
chr13	73901144	73954190	Slc12a7	0	+	MACS_peak_4595	-28972
chr13	74777319	74829323	Erap1	0	+	MACS_peak_4600	22538
chr13	76236181	76325431	Ttc37	0	+	MACS_peak_4617	-6001
chr13	76197869	76236108	Arsk	0	-	MACS_peak_4617	6074
chr13	77847950	78305499	Fam172a	0	+	MACS_peak_4628|MACS_peak_4628|MA	-91|-91|-91|-91
chr13	78164162	78165557	Pou5f2	0	+	MACS_peak_4630	18463
chr13	85329081	85353328	Ccnh	0	+	MACS_peak_4632|MACS_peak_4633	-26315|-29971
chr13	8971723	8995258	Gtpbp4	0	-	MACS_peak_4134	-23056
chr13	8952108	8960181	Idi2	0	+	MACS_peak_4134	-20094
chr13	89794914	89882117	Vcan	0	-	MACS_peak_4654|MACS_peak_4654|MA	-11015|-11015|-11015|-11015|-11015
chr13	93219576	93253986	Fam151b	0	-	MACS_peak_4660|MACS_peak_4661	10985|24202
chr13	93257703	93300765	Zfyve16	0	-	MACS_peak_4661|MACS_peak_4662	-22577|-8124
chr13	94204156	94269644	Jmy	0	-	MACS_peak_4673|MACS_peak_4674	-1178|-457
chr13	97907891	97951150	Gfm2	0	+	MACS_peak_4688|MACS_peak_4688	-25184|-25184
chr13	97899381	97907881	Nsa2	0	-	MACS_peak_4688	25194
chr13	98011059	98022994	Enc1	0	+	MACS_peak_4697	-9508
chr13	99033196	99043868	Ankra2	0	+	MACS_peak_4707	-3109
chr13	99016799	99032947	Utp15	0	-	MACS_peak_4707	3358
chr13	99404932	99407365	Tmem174	0	-	MACS_peak_4709	-23092
chr14	103469432	103476847	Cln5	0	+	MACS_peak_5388	-29085
chr14	103479455	103498726	Fbxl3	0	-	MACS_peak_5388	-209
chr14	106291163	106296036	Spry2	0	-	MACS_peak_5412|MACS_peak_5413	-3596|-14
chr14	118881913	119076153	Abcc4	0	-	MACS_peak_5442|MACS_peak_5443|MA	-22675|-8531|-48|29240|-48
chr14	122506303	122558825	Tm9sf2	0	+	MACS_peak_5454	-195
chr14	122497160	122506196	A330035P	0	-	MACS_peak_5454	302
chr14	122580915	122801454	Clybl	0	+	MACS_peak_5456	29673
chr14	123290081	123312387	A2ld1	0	-	MACS_peak_5461	14444
chr14	17197719	17263301	Top2b	0	+	MACS_peak_4891	-17933
chr14	21852964	21871664	Ap3m1	0	-	MACS_peak_4917	-22609
chr14	21871854	22267768	Adk	0	+	MACS_peak_4917	22799
chr14	22569752	22617947	Samd8	0	+	MACS_peak_4922	-29605
chr14	25267915	25306268	Polr3a	0	-	MACS_peak_4935|MACS_peak_4936|MA	-16442|-11238|-7872|-4592|-4168
chr14	25309902	25315368	Rps24	0	+	MACS_peak_4935|MACS_peak_4935|MA	20076|20076|20076|14872|14872|14872|11506|11506|11506|8226|8226|8226|7802|7802|7802
chr14	26406289	26410147	4931406H	0	+	MACS_peak_4941|MACS_peak_4942	10617|5183
chr14	26847658	26862145	Plac9	0	-	MACS_peak_4954|MACS_peak_4956	-18683|-16549
chr14	31948038	31952310	Nt5dc2	0	+	MACS_peak_4977	-25436
chr14	31952202	31981827	Stab1	0	-	MACS_peak_4977	-8353
chr14	3224445	3234345	D830030K	0	+	MACS_peak_4829	13358
chr14	32291983	32308163	Mettl6	0	-	MACS_peak_4978	-1196
chr14	32308264	32323043	Eaf1	0	+	MACS_peak_4978	1297
chr14	33326706	33394175	Ercc6	0	+	MACS_peak_5004	-12961
chr14	34191083	34260344	Mapk8	0	-	MACS_peak_5005	-18344
chr14	37688121	37748754	Gcap14	0	-	MACS_peak_5012	-17058
chr14	37934003	37948452	Ghitm	0	-	MACS_peak_5015|MACS_peak_5016|MA	-10034|-9272|-4564
chr14	37891034	37911497	Cdhr1	0	-	MACS_peak_5015|MACS_peak_5016	26921|27683
chr14	45607785	45623495	Ptger2	0	+	MACS_peak_5030|MACS_peak_5033	14192|-14818
chr14	45839391	45900651	Gpr137c	0	+	MACS_peak_5045	426
chr14	45754122	45839069	Txndc16	0	-	MACS_peak_5045	-104

chr14	45903995	45938202	Ero1l	0	-	MACS_peak_5049|MACS_peak_5051|MA(	-26133|-24211|-18624|-13238|-11955|-73
chr14	45949498	45968746	Psmc6	0	+	MACS_peak_5052|MACS_peak_5054|MA(	29920|24534|23251|11369
chr14	45996095	46008471	Gnpnat1	0	-	MACS_peak_5058|MACS_peak_5059|MA(	-10859|-7162|-4142
chr14	45970860	45996559	Styx	0	+	MACS_peak_5058	-26752
chr14	47502639	47725492	Samd4	0	+	MACS_peak_5069|MACS_peak_5069	29542|29542
chr14	47451918	47473865	Cgrrf1	0	+	MACS_peak_5069	-21179
chr14	47773569	47809077	Gch1	0	-	MACS_peak_5074	-22897
chr14	48160568	48188109	Atg14	0	-	MACS_peak_5078	-21130
chr14	48283430	48356239	Ktn1	0	+	MACS_peak_5080|MACS_peak_5081|MA(	-3608|-6457|-11055|-19684|-21027|-26272
chr14	51427620	51440975	Parp2	0	+	MACS_peak_5100	-29162
chr14	51427121	51427446	Rpph1	0	+	MACS_peak_5100	-29661
chr14	51443735	51490229	Tep1	0	-	MACS_peak_5102|MACS_peak_5104|MA(	-28920|-24016|-22849|-17494|-16912|-16329
chr14	51710751	51721682	Ang	0	+	MACS_peak_5113|MACS_peak_5113	-10539|-5616
chr14	51710751	51725826	Rnase4	0	+	MACS_peak_5113|MACS_peak_5113	-10539|-5616
chr14	51748742	51750794	Rnase6	0	+	MACS_peak_5113	27452
chr14	51736227	51737466	Eddm3b	0	+	MACS_peak_5113	14937
chr14	52654849	52655784	Olfr221	0	-	MACS_peak_5116|MACS_peak_5117	-29481|-27186
chr14	52636539	52639462	G630016D	0	+	MACS_peak_5116|MACS_peak_5117	10236|7941
chr14	52604507	52625922	E130112L2	0	+	MACS_peak_5116|MACS_peak_5116|MA(	-21796|-21796|-21796|-24091|-24091|-24091
chr14	52625751	52640408	Zfp219	0	-	MACS_peak_5116|MACS_peak_5117	-14105|-11810
chr14	52688989	52689043	Snord58b	0	+	MACS_peak_5118|MACS_peak_5121	-487|-28808
chr14	52730577	52781013	Rpgrip1	0	+	MACS_peak_5121|MACS_peak_5121	12780|12780
chr14	52693054	52723703	Hnrnpc	0	-	MACS_peak_5121|MACS_peak_5121|MA(	-5906|-5906|-5906|-5906|-5906
chr14	52780093	52816914	Supt16h	0	-	MACS_peak_5122|MACS_peak_5123|MA(	7912|19105|20603
chr14	52829461	52829559	Snord8	0	-	MACS_peak_5122|MACS_peak_5123|MA(	-4733|6460|7958
chr14	52817825	52857247	Chd8	0	-	MACS_peak_5123|MACS_peak_5124	-21228|-19730
chr14	52930855	52948345	Sall2	0	-	MACS_peak_5126	-14074
chr14	52914516	52924799	Mettl3	0	-	MACS_peak_5126	9472
chr14	55082983	55089128	Lrp10	0	+	MACS_peak_5147|MACS_peak_5149	-3373|-4952
chr14	55094936	55099271	Rem2	0	+	MACS_peak_5147|MACS_peak_5149	8580|7001
chr14	55341051	55400723	Slc7a8	0	-	MACS_peak_5156	-14448
chr14	55494433	55496375	Ppp1r3e	0	-	MACS_peak_5157	29315
chr14	55502261	55507071	Bcl2l2	0	+	MACS_peak_5157	-23429
chr14	55535379	55545625	Efs	0	-	MACS_peak_5157	-19935
chr14	55512979	55517764	Pabpn1	0	+	MACS_peak_5157	-12711
chr14	55525563	55531969	Slc22a17	0	-	MACS_peak_5157	-6279
chr14	55555306	55558114	Cmtm5	0	+	MACS_peak_5157	29616
chr14	55551531	55554671	Il25	0	+	MACS_peak_5157	25841
chr14	56138360	56143802	Nrl	0	-	MACS_peak_5158|MACS_peak_5158|MA(	17582|18356|18720|19494|19050|19824|19471|20245|20339|21113|22799|23573
chr14	56159102	56168854	Pck2	0	+	MACS_peak_5158|MACS_peak_5159|MA(	-2282|-3420|-3750|-4171|-5039|-7499|-22695
chr14	56178868	56188902	Dcaf11	0	+	MACS_peak_5158|MACS_peak_5159|MA(	17484|16346|16016|15595|14727|12267|-2929
chr14	56194510	56195789	Fitm1	0	+	MACS_peak_5164|MACS_peak_5166|MA(	27909|12713|-23466
chr14	56206276	56209938	Psme2	0	-	MACS_peak_5166|MACS_peak_5166|MA(	-28141|-28061|8038|8118
chr14	56197330	56200364	Psme1	0	+	MACS_peak_5166|MACS_peak_5171	15533|-20646
chr14	56200360	56204091	Fam158a	0	-	MACS_peak_5166|MACS_peak_5171	-22294|13885
chr14	56210626	56222508	Rnf31	0	+	MACS_peak_5166|MACS_peak_5171	28829|-7350
chr14	56222821	56228866	Irf9	0	+	MACS_peak_5171|MACS_peak_5171|MA(	4845|5439|4845
chr14	56237006	56244227	Rec8	0	+	MACS_peak_5171	19030
chr14	57145597	57190683	Cenpj	0	-	MACS_peak_5184|MACS_peak_5187	18582|26533
chr14	57194455	57278635	Parp4	0	+	MACS_peak_5184|MACS_peak_5187	-14810|-22761
chr14	57506630	57579186	Zmym2	0	+	MACS_peak_5201|MACS_peak_5203	21203|-26268
chr14	58445075	58447582	Mrp63	0	+	MACS_peak_5207|MACS_peak_5208	-14637|-17012
chr14	58425397	58445000	F630043A(	0	-	MACS_peak_5207|MACS_peak_5208	14712|17087
chr14	63295164	63379949	Ints6	0	-	MACS_peak_5212	-17161
chr14	66149292	66211847	Elp3	0	-	MACS_peak_5226	-2451
chr14	66772093	66899889	Ptk2b	0	-	MACS_peak_5249|MACS_peak_5249|MA(	16287|16287|23068|23068
chr14	66915861	66930261	Trim35	0	+	MACS_peak_5249|MACS_peak_5250	-315|-7096
chr14	67604076	67627714	Bnip3l	0	-	MACS_peak_5262	7268
chr14	70167278	70184210	Tnfrsf10b	0	+	MACS_peak_5272|MACS_peak_5273|MA(	-5960|-7670|-15404
chr14	70184796	70205352	Rhobtb2	0	-	MACS_peak_5274|MACS_peak_5276|MA(	-22670|-5811|-405
chr14	70580274	70607444	Sorbs3	0	-	MACS_peak_5283	2726
chr14	70617704	70689256	Ppp3cc	0	-	MACS_peak_5284|MACS_peak_5285	14796|16487
chr14	70703273	70751231	Slc39a14	0	-	MACS_peak_5286|MACS_peak_5286|MA(	-130|2861|-130
chr14	70838556	70843034	Polr3d	0	-	MACS_peak_5287|MACS_peak_5287	-164|-408
chr14	70772286	70828901	Piwil2	0	-	MACS_peak_5287	13969
chr14	70857323	70868631	Phyhip	0	+	MACS_peak_5287	14453
chr14	70843316	70843398	Mir320	0	+	MACS_peak_5287	446
chr14	71289936	71379645	Gfra2	0	+	MACS_peak_5289	-5832
chr14	73637697	73640165	Lpar6	0	+	MACS_peak_5309	-26440
chr14	75132151	75150250	Esd	0	+	MACS_peak_5321	-10975
chr14	75684179	75744233	Zc3h13	0	+	MACS_peak_5330|MACS_peak_5331|MA(	-86|-7254|-21464|-25336
chr14	76102157	76154300	Cog3	0	-	MACS_peak_5340|MACS_peak_5342	-13911|8871
chr14	76161805	76186844	Slc25a30	0	-	MACS_peak_5342	-23673
chr14	76510697	76537186	Nufip1	0	+	MACS_peak_5354	15837
chr14	76486038	76510622	1200011I1	0	-	MACS_peak_5354	-15762
chr14	76815627	76907573	Tsc22d1	0	+	MACS_peak_5355	-2641
chr14	8650470	8784101	Flnb	0	+	MACS_peak_4842	-265
chr14	9058025	9091353	Acox2	0	-	MACS_peak_4873|MACS_peak_4873	-7240|-7420
chr14	93412919	94287951	Pcdh9	0	-	MACS_peak_5379	-3718
chr14	99475852	99498989	Dis3	0	-	MACS_peak_5381	1390
chr14	99498651	99653712	Pibf1	0	+	MACS_peak_5381|MACS_peak_5381	-1728|-1728
chr14	99697909	99712628	Klf5	0	+	MACS_peak_5383|MACS_peak_5386	-296|-15380
chr15	100058289	100091679	Atf1	0	+	MACS_peak_6241	20073
chr15	100195015	100214386	Higd1c	0	+	MACS_peak_6244	-24301
chr15	100223088	100253486	Slc11a2	0	-	MACS_peak_6245|MACS_peak_6245|MA(	-21719|-21719|-20715|-20715
chr15	100958967	100975767	Acvrl1	0	+	MACS_peak_6250|MACS_peak_6251	-8895|-14863
chr15	100946185	100950455	Ankrd33	0	+	MACS_peak_6250|MACS_peak_6251	-21677|-27645
chr15	101004555	101043032	Acvr1b	0	+	MACS_peak_6253|MACS_peak_6254	-20876|-27951
chr15	101054637	101063187	Grasp	0	+	MACS_peak_6253|MACS_peak_6254|MA(	29206|22131|-8053
chr15	101097276	101105223	Nr4a1	0	+	MACS_peak_6257	-17583
chr15	101114731	101121365	9430023L2	0	+	MACS_peak_6257	-128
chr15	101123642	101127857	6030408B1	0	+	MACS_peak_6257	8783
chr15	101904203	101927604	Eif4b	0	+	MACS_peak_6261	-12256
chr15	101933418	101946832	Tenc1	0	+	MACS_peak_6261|MACS_peak_6268	16959|-6750
chr15	101946958	101966646	Spryd3	0	-	MACS_peak_6268	-26478
chr15	102065370	102076783	Rarg	0	-	MACS_peak_6293|MACS_peak_6293	-14471|-25602
chr15	102046425	102062366	Itgb7	0	-	MACS_peak_6293	-54
chr15	102038697	102046032	Zfp740	0	+	MACS_peak_6293	-23615
chr15	102289600	102293236	Prr13	0	+	MACS_peak_6300|MACS_peak_6300	25623|25623
chr15	102236746	102266835	Sp1	0	+	MACS_peak_6300	-27231
chr15	102275797	102285070	Amhr2	0	+	MACS_peak_6300	11820
chr15	103103348	103130295	Copz1	0	+	MACS_peak_6320	-17798
chr15	103138665	103140869	Gpr84	0	-	MACS_peak_6320	-19723
chr15	103115555	103115651	Mir148b	0	+	MACS_peak_6320	-5591
chr15	103174719	103197179	Itga5	0	-	MACS_peak_6322|MACS_peak_6326|MA(	-20736|-16335|-16134|-14572|-14213|-13514|-10370|-7596|-359
chr15	103144325	103170517	Zfp385a	0	-	MACS_peak_6322|MACS_peak_6326|MA(	5926|10327|10528|12090|12449|13148|16292|19066|26303
chr15	10433701	10488423	Ttc23l	0	-	MACS_peak_5518	13738
chr15	10530047	10541406	Gm10389	0	+	MACS_peak_5518|MACS_peak_5521|MA(	27886|539|-9756
chr15	11313417	11329413	Tars	0	-	MACS_peak_5524|MACS_peak_5525|MA(	-15745|-14515|-8516|-5339
chr15	12101246	12101329	Mir1898	0	+	MACS_peak_5528	11808
chr15	31370964	31383444	Ropn1l	0	-	MACS_peak_5571|MACS_peak_5572|MA(	3898|8322|11631|15919|22092|28868|29175
chr15	3230626	3253526	Ccdc152	0	-	MACS_peak_5476|MACS_peak_5478	22392|29908
chr15	33334963	33335694	1700084J1	0	-	MACS_peak_5605	-24547
chr15	34236435	34365995	Matn2	0	+	MACS_peak_5615	-9903
chr15	34370262	34373031	Rpl30	0	-	MACS_peak_5626|MACS_peak_5626	-10177|-10126
chr15	34383066	34403646	BC030476	0	+	MACS_peak_5626	20212
chr15	34370260	34372988	Gm12191	0	-	MACS_peak_5626	-10134
chr15	3277524	3533230	Ghr	0	-	MACS_peak_5484|MACS_peak_5484	-149|-149
chr15	37853707	37890810	Rrm2b	0	-	MACS_peak_5645|MACS_peak_5646|MA(	18759|23033|24876|27257
chr15	38417184	38449021	Azin1	0	-	MACS_peak_5655|MACS_peak_5655|MA(	-29947|-29947|-27718|-27718|-23955|-23955|-11998|-11998|-187|-187
chr15	39614243	39689016	Dpys	0	-	MACS_peak_5661|MACS_peak_5661|MA(	14878|14878|15250|15250
chr15	40486587	40936138	Zfpm2	0	+	MACS_peak_5663	-15763

chr15	41621060	41692593	Oxr1	0	+	MACS_peak_5666|MACS_peak_5666|MA	-11028|-11509|-11028|-22411
chr15	41696838	41701266	Abra	0	-	MACS_peak_5667	-16352
chr15	43698240	43701575	Tmem74	0	-	MACS_peak_5669|MACS_peak_5670|MA	-8087|-7853|-6010|-5151|-4367|-3483|-2565
chr15	5093860	5131899	Prkaa1	0	+	MACS_peak_5486|MACS_peak_5487	-77|-20903
chr15	5066612	5069140	Rpl37	0	+	MACS_peak_5486	-27325
chr15	5068420	5068479	Snord72	0	+	MACS_peak_5486	-25517
chr15	5135559	5168332	Ttc33	0	+	MACS_peak_5487	20796
chr15	51708986	51716168	Utp23	0	+	MACS_peak_5684|MACS_peak_5685	-6979|-16842
chr15	51618108	51697007	Eif3h	0	-	MACS_peak_5684|MACS_peak_5685	18958|28821
chr15	5183405	5193682	Ptger4	0	-	MACS_peak_5488|MACS_peak_5488	-19019|-19168
chr15	57701056	57723973	Derl1	0	-	MACS_peak_5699	-12341
chr15	58765364	58771044	Ndufb9	0	+	MACS_peak_5711|MACS_peak_5712|MA	-11612|-12133|-22702
chr15	58721707	58765285	Tatdn1	0	-	MACS_peak_5711|MACS_peak_5712|MA	11691|12212|22781
chr15	59146646	59162748	Sqle	0	+	MACS_peak_5719|MACS_peak_5720|MA	-506|-6311|-7791|-8800
chr15	60650550	60656635	Fam84b	0	-	MACS_peak_5726|MACS_peak_5728	-5661|-4149
chr15	60654519	60662954	9930014A1	0	+	MACS_peak_5726|MACS_peak_5728	3545|2033
chr15	61816895	61821916	Myc	0	+	MACS_peak_5731|MACS_peak_5731|MA	-335|-335|-335|-335|-2411|-2411|-2411|-2411
chr15	61869541	62082530	Pvt1	0	+	MACS_peak_5734	-6037
chr15	62049095	62051006	H2afy3	0	-	MACS_peak_5740|MACS_peak_5743	-4838|25376
chr15	6336747	6390709	Dab2	0	+	MACS_peak_5490|MACS_peak_5490|MA	-48|-48|-48
chr15	66760879	66801203	Ndrg1	0	-	MACS_peak_5749|MACS_peak_5755	-27601|-9118
chr15	75577272	75578352	Mafa	0	-	MACS_peak_5794	6835
chr15	75900613	75911300	Puf60	0	-	MACS_peak_5805|MACS_peak_5805|MA	-19708|-19784|-19784|5206|5130|5130|5572|5496|5496
chr15	75877615	75900160	Scrib	0	-	MACS_peak_5805|MACS_peak_5806|MA	-8568|16346|16712
chr15	75916023	75920443	Nrbp2	0	-	MACS_peak_5805|MACS_peak_5806|MA	-28851|-3937|-3571
chr15	76001404	76036751	Plec	0	-	MACS_peak_5810|MACS_peak_5810|MA	-22617|-16131|-24549|-25461|-24949|-24549|-22617|-16903|-16131|-15702|-14505|-12006|-20964|-14478|-22896|-23808|-23296|-22896|-
chr15	76046041	76046103	Mir1942	0	-	MACS_peak_5812|MACS_peak_5813|MA	-29728|-29468|-27130|-26670|-26339|-25524|-24376|-24149|-23408|-21287|-20097
chr15	76127032	76137675	Oplah	0	-	MACS_peak_5834|MACS_peak_5836|MA	-8768|-5110|-2051
chr15	76157826	76161100	Exosc4	0	+	MACS_peak_5834|MACS_peak_5836|MA	28919|25261|22202|-25529
chr15	76156446	76157963	LOC100302	0	-	MACS_peak_5834|MACS_peak_5836|MA	-29056|-25398|-22339|25392
chr15	76161723	76165329	Gpaa1	0	+	MACS_peak_5836|MACS_peak_5838|MA	29158|26099|-21632
chr15	76181723	76184808	Maf1	0	+	MACS_peak_5842|MACS_peak_5842|MA	-1632|-1632|-1632
chr15	76199327	76201841	Brp16	0	+	MACS_peak_5842|MACS_peak_5847|MA	15972|-21172|-24981
chr15	76177469	76181540	Sharpin	0	-	MACS_peak_5842	1815
chr15	76173952	76176364	Cyc1	0	+	MACS_peak_5842	-9403
chr15	76210942	76283469	Heatr7a	0	+	MACS_peak_5842|MACS_peak_5847|MA	27587|-39|-9557|-3848|-13366|-18426|-27944
chr15	76202387	76205368	Tssk5	0	-	MACS_peak_5842|MACS_peak_5847|MA	-22013|15131|18940
chr15	76287867	76289898	Scx	0	+	MACS_peak_5859|MACS_peak_5860|MA	21379|20124|10175|7289|5552|5152
chr15	76307874	76331402	Hsf1	0	+	MACS_peak_5864|MACS_peak_5865|MA	27296|25559|25159|-25080|-26741
chr15	76283425	76307699	Bop1	0	-	MACS_peak_5864|MACS_peak_5865|MA	-27121|-25384|-24984|25255|26916
chr15	76332444	76342248	Dgat1	0	-	MACS_peak_5867|MACS_peak_5869	-9294|-7633
chr15	76346632	76352559	Scrt1	0	-	MACS_peak_5867|MACS_peak_5869	-19605|-17944
chr15	76426238	76438021	Cpsf1	0	-	MACS_peak_5870|MACS_peak_5870|MA	-6851|-6851|-6598|-6598|-5627|-5627
chr15	76452517	76456457	Vps28	0	-	MACS_peak_5870|MACS_peak_5871|MA	-25287|-25034|-24063
chr15	76442812	76447282	Slc39a4	0	-	MACS_peak_5870|MACS_peak_5871|MA	-16112|-15859|-14888
chr15	76406788	76426241	Adck5	0	+	MACS_peak_5870|MACS_peak_5871|MA	-24382|-24635|-25606
chr15	76531971	76534669	Mfsd3	0	+	MACS_peak_5875	-20219
chr15	76534170	76540906	Recql4	0	-	MACS_peak_5875	11284
chr15	76541169	76545521	Lrrc14	0	+	MACS_peak_5875	-11021
chr15	76551895	76554275	C030006K1	0	-	MACS_peak_5875|MACS_peak_5875	-2085|-2085
chr15	76527193	76530105	Gpt	0	+	MACS_peak_5875	-24997
chr15	76545705	76552603	Lrrc24	0	-	MACS_peak_5875	-413
chr15	77579043	77585659	Apol8	0	-	MACS_peak_5886|MACS_peak_5889|MA	8044|9358|10536
chr15	77591018	77672545	Myh9	0	-	MACS_peak_5919	-82
chr15	77770953	77787152	Foxred2	0	-	MACS_peak_5923|MACS_peak_5923|MA	-9646|-9646|-8275|-8275|10683|10683
chr15	77789427	77801254	Eif3d	0	-	MACS_peak_5923|MACS_peak_5924|MA	-23748|-22377|-3419
chr15	77745480	77759424	Txn2	0	-	MACS_peak_5923|MACS_peak_5924	18082|19453
chr15	78730215	78742482	Sh3bp1	0	+	MACS_peak_5934|MACS_peak_5935|MA	9944|-120|-8224
chr15	78744348	78749947	Pdxp	0	+	MACS_peak_5934|MACS_peak_5935|MA	24077|14013|5909
chr15	78707619	78725014	Gga1	0	+	MACS_peak_5934|MACS_peak_5935	-12652|-22716
chr15	78757154	78760895	Lgals1	0	+	MACS_peak_5935|MACS_peak_5936	26819|18715
chr15	78765362	78772340	Nol12	0	+	MACS_peak_5936	26923
chr15	79059811	79079896	Pick1	0	+	MACS_peak_5939|MACS_peak_5939	-420|-628
chr15	79081445	79085178	Slc16a8	0	-	MACS_peak_5939	-24947
chr15	79116657	79158598	Pla2g6	0	-	MACS_peak_5943|MACS_peak_5944|MA	-23806|-10286|28833
chr15	79088624	79115939	Baiap2l2	0	-	MACS_peak_5943	18853
chr15	79178107	79189506	Maff	0	+	MACS_peak_5944|MACS_peak_5946|MA	29795|-9324|-29466
chr15	79205657	79205730	Mir1943	0	-	MACS_peak_5946|MACS_peak_5949	-18299|1843
chr15	79191113	79233733	Tmem184b	0	-	MACS_peak_5949	-26160
chr15	79358125	79377171	Ddx17	0	-	MACS_peak_5951|MACS_peak_5951|MA	-6293|7531|-6111|-6293
chr15	79346837	79358168	Kdelr3	0	+	MACS_peak_5951	-24041
chr15	79521325	79551909	Gtpbp1	0	+	MACS_peak_5952	15680
chr15	79489656	79498090	Cby1	0	+	MACS_peak_5952	-15989
chr15	79504679	79518302	Josd1	0	-	MACS_peak_5952	-12657
chr15	79440005	79489386	4933432B0	0	-	MACS_peak_5952	16259
chr15	79501297	79503292	Tomm22	0	+	MACS_peak_5952	-4348
chr15	79554499	79572960	Sun2	0	-	MACS_peak_5958|MACS_peak_5964|MA	-15416|-8362|-3941
chr15	79921763	79943870	Syngr1	0	+	MACS_peak_5975|MACS_peak_5975	-27802|-27802
chr15	79963583	79992132	Tab1	0	+	MACS_peak_5975	14018
chr15	80085613	80087971	Atf4	0	+	MACS_peak_5976	-386
chr15	80091043	80094736	Rps19bp1	0	-	MACS_peak_5976	-8737
chr15	80064509	80083799	Smcr7l	0	+	MACS_peak_5976	-21490
chr15	7823054	8049209	Wdr70	0	-	MACS_peak_5505	17276
chr15	8059312	8109859	Nup155	0	+	MACS_peak_5505	-7173
chr15	8119105	8221157	2410089E0	0	+	MACS_peak_5506	-17014
chr15	81416643	81482507	Ep300	0	+	MACS_peak_5990	596
chr15	81641843	81657291	Tef	0	+	MACS_peak_5995|MACS_peak_5995	-243|-8984
chr15	81745459	81756643	Polr3h	0	-	MACS_peak_5998|MACS_peak_5999|MA	-22284|-12969|-11357|25149|29487
chr15	81767188	81781370	Csdc2	0	+	MACS_peak_5999|MACS_peak_6000|MA	23514|21902|-14604|-18942
chr15	81781535	81791297	Pmm1	0	-	MACS_peak_6001|MACS_peak_6002	-9505|-5167
chr15	81810974	81811993	1700029P1	0	+	MACS_peak_6001|MACS_peak_6002	29182|24844
chr15	81977698	82035390	Srebf2	0	+	MACS_peak_6005	-22675
chr15	82028551	82028620	Mir33	0	+	MACS_peak_6005|MACS_peak_6007	28178|1893
chr15	82052173	82054766	Tnfrsf13c	0	-	MACS_peak_6007	-28108
chr15	82946278	82953231	Rrp7a	0	-	MACS_peak_6026	26462
chr15	82983931	83002638	Cyb5r3	0	-	MACS_peak_6026	-22945
chr15	82956407	82979766	Poldip3	0	-	MACS_peak_6026	-73
chr15	82980074	82980224	Rnu12	0	+	MACS_peak_6026	381
chr15	83130169	83180677	Arfgap3	0	-	MACS_peak_6034|MACS_peak_6035	-22614|-17520
chr15	83184277	83197727	1700001L0	0	-	MACS_peak_6037	19448
chr15	83357291	83375065	Bik	0	+	MACS_peak_6038	21572
chr15	83314198	83341337	Ttll1	0	-	MACS_peak_6038	-5618
chr15	84062472	84146039	Parvb	0	+	MACS_peak_6040	-79
chr15	84156548	84173408	Parvg	0	+	MACS_peak_6041|MACS_peak_6041	11806|10407
chr15	85365869	85411159	Wnt7b	0	-	MACS_peak_6052|MACS_peak_6052|MA	-21721|-19062|-22813
chr15	88635673	88649748	Alg12	0	-	MACS_peak_6059|MACS_peak_6059	-3175|-3175
chr15	88650075	88657111	Creld2	0	+	MACS_peak_6059	3502
chr15	88692623	88696156	Pim3	0	+	MACS_peak_6060|MACS_peak_6062	-1196|-2936
chr15	88929527	88953580	Tubgcp6	0	-	MACS_peak_6065|MACS_peak_6067	-27460|-24486
chr15	88906493	88917505	Trabd	0	+	MACS_peak_6065|MACS_peak_6067	-19627|-22601
chr15	88919536	88930770	1300018J1	0	+	MACS_peak_6065|MACS_peak_6067	-6584|-9558
chr15	88985978	89004280	Plxnb2	0	-	MACS_peak_6074|MACS_peak_6074|MA	-13155|-20093|-12096|-19034|-9586|-16524|-7420|-14358|-7179|-14117|-6380|-13318
chr15	88972913	88980036	Mapk11	0	-	MACS_peak_6074|MACS_peak_6075|MA	11089|12148|14658|16824|17065|17864
chr15	88961014	88971133	Mapk12	0	-	MACS_peak_6074|MACS_peak_6075|MA	19992|21051|23561|25727|25968|26767
chr15	89012642	89026905	Fam116b	0	-	MACS_peak_6078|MACS_peak_6079	-29804|-29005
chr15	89118667	89145742	Sbf1	0	-	MACS_peak_6081|MACS_peak_6081|MA	-8884|-8884|7753|7753|8016|8016
chr15	89164903	89167438	Miox	0	+	MACS_peak_6081|MACS_peak_6083|MA	28045|11408|11145
chr15	89153402	89155161	Adm2	0	+	MACS_peak_6081|MACS_peak_6083|MA	16544|-93|-356
chr15	90721826	90728907	Spn-ps	0	-	MACS_peak_6094	-15396
chr15	91503654	91646555	Lrrk2	0	+	MACS_peak_6097|MACS_peak_6099|MA	-105|-9914|-12569|-15672|-26719
chr15	93228780	93322343	Pphln1	0	+	MACS_peak_6143|MACS_peak_6143|MA	-1050|-1050|-1050|-22044|-22044|-22044
chr15	93216543	93228721	Zcrb1	0	-	MACS_peak_6143|MACS_peak_6144	1109|22103

chr15	96517822	96530129	Slc38a2	0	-	MACS_peak_6150|MACS_peak_6152|MA	-8536|-7936|-7342|-6603|-4667|-4446|-1494|-987|-159
chr15	97541449	97561836	Endou	0	-	MACS_peak_6171|MACS_peak_6171|MA	15689|25944|18955|29210|22442|28600|29764
chr15	97575200	97598097	Rapgef3	0	-	MACS_peak_6171|MACS_peak_6171|MA	-20572|-20572|-20572|-17306|-17306|-17306|-13819|-13819|-13819|-7661|-7661|-7661|-6497|-6497|-6497|16845|16845|16845
chr15	97614795	97623121	Slc48a1	0	+	MACS_peak_6182|MACS_peak_6184|MA	24359|23195|-147
chr15	98349531	98364652	2310037I2	0	-	MACS_peak_6201|MACS_peak_6202	-2414|-138
chr15	98350147	98350265	Snora34	0	-	MACS_peak_6201|MACS_peak_6202	11973|14249
chr15	98356778	98356890	Snora2b	0	-	MACS_peak_6201|MACS_peak_6202	5348|7624
chr15	98420420	98438064	Adcy6	0	-	MACS_peak_6203|MACS_peak_6204|MA	-14901|-9213|-8937|-7667|-6647
chr15	98373641	98398067	Ccnt1	0	-	MACS_peak_6203	25096
chr15	98397754	98401295	9330020H	0	+	MACS_peak_6203	-25409
chr15	98475937	98493320	Ddx23	0	-	MACS_peak_6208|MACS_peak_6209|MA	7205|7683|9714|13641|14462
chr15	98499635	98507892	Rnd1	0	-	MACS_peak_6208|MACS_peak_6209|MA	-7367|-6889|-4858|-931|-110
chr15	98554798	98558629	Fkbp11	0	-	MACS_peak_6213|MACS_peak_6214	-3696|-484
chr15	98538657	98553764	Ccdc65	0	+	MACS_peak_6213|MACS_peak_6214	-16276|-19488
chr15	98723457	98728971	Dhh	0	-	MACS_peak_6217|MACS_peak_6218	6210|9401
chr15	98761861	98764821	Tuba1b	0	-	MACS_peak_6217|MACS_peak_6218	-29640|-26449
chr15	98708191	98711845	Rhebl1	0	-	MACS_peak_6217|MACS_peak_6218	23336|26527
chr15	98734351	98748529	Lmbr1l	0	-	MACS_peak_6217|MACS_peak_6218	-13348|-10157
chr15	99101448	99117611	Fam186b	0	-	MACS_peak_6219|MACS_peak_6220|MA	18401|19319|21955
chr15	99125839	99147438	Prpf40b	0	+	MACS_peak_6219|MACS_peak_6220|MA	-10173|-11091|-13727
chr15	99409486	99414976	Aqp2	0	+	MACS_peak_6230|MACS_peak_6232	2741|382
chr15	99421458	99425260	Aqp5	0	+	MACS_peak_6230|MACS_peak_6232	14713|12354
chr15	99431830	99435908	Aqp6	0	+	MACS_peak_6230|MACS_peak_6232	25085|22726
chr15	99984826	100049904	Dip2b	0	+	MACS_peak_6238	12309
chr16	10281841	10314061	Emp2	0	-	MACS_peak_6378|MACS_peak_6379|MA	-29529|-28444|-21625
chr16	13109828	13152102	Ercc4	0	+	MACS_peak_6388	-20941
chr16	13358513	13417621	Mkl2	0	+	MACS_peak_6389	-27274
chr16	13671950	13703701	Bfar	0	+	MACS_peak_6393|MACS_peak_6393|MA	-13377|-13377|-15632|-15632
chr16	13672112	13678478	3110001I2	0	+	MACS_peak_6393|MACS_peak_6394	-13215|-15470
chr16	13538056	13668263	Parn	0	-	MACS_peak_6393|MACS_peak_6394	17064|19319
chr16	13780791	13814934	Rrn3	0	+	MACS_peak_6396	15119
chr16	13834022	13903228	Pdxdc1	0	-	MACS_peak_6400|MACS_peak_6400	-27066|-27066
chr16	14110625	14159367	4921513D	0	-	MACS_peak_6402|MACS_peak_6403	-20189|-17557
chr16	14163367	14193016	Nde1	0	+	MACS_peak_6402|MACS_peak_6402|MA	24189|24189|21557|21557
chr16	14159718	14159784	Mir484	0	+	MACS_peak_6402|MACS_peak_6403	20540|17908
chr16	14361652	14474967	Abcc1	0	+	MACS_peak_6406|MACS_peak_6407	-28335|-29823
chr16	14705951	14709475	Snai2	0	+	MACS_peak_6434	-1163
chr16	15889319	16146926	2310008H	0	-	MACS_peak_6440	-27944
chr16	16860763	16864078	Igll1	0	-	MACS_peak_6442|MACS_peak_6443	19509|21878
chr16	16870983	16893079	Top3b	0	+	MACS_peak_6442|MACS_peak_6443	-12604|-14973
chr16	16868495	16869348	Vpreb1	0	-	MACS_peak_6442|MACS_peak_6443	14239|16608
chr16	16903413	16927455	Ppm1f	0	+	MACS_peak_6442|MACS_peak_6443	19826|17457
chr16	17070403	17086781	Ypel1	0	+	MACS_peak_6444	25825
chr16	20141135	20232646	Yeats2	0	+	MACS_peak_6462|MACS_peak_6462|MA	-9432|-9432|-9432
chr16	20176222	20176307	Mir684-1	0	-	MACS_peak_6462|MACS_peak_14731	-25740|22998
chr16	20517136	20532260	Dvl3	0	+	MACS_peak_6466	-14289
chr16	20535576	20544126	Ap2m1	0	+	MACS_peak_6466	4151
chr16	20548675	20561676	Abcf3	0	+	MACS_peak_6466	17250
chr16	20545186	20548629	Gm15760	0	-	MACS_peak_6466	-17204
chr16	20724528	20730671	Thpo	0	-	MACS_peak_6468|MACS_peak_6469|MA	-26999|-17211|-21124
chr16	20733199	20742457	Chrd	0	+	MACS_peak_6468|MACS_peak_6469	29527|19739
chr16	20703038	20716709	Clcn2	0	-	MACS_peak_6468|MACS_peak_6469	-13037|-3249
chr16	20695129	20703109	Fam131a	0	+	MACS_peak_6468|MACS_peak_6469	-8543|-18331
chr16	20717898	20722338	Polr2h	0	+	MACS_peak_6468|MACS_peak_6469	14226|4438
chr16	20684328	20684384	Snord66	0	+	MACS_peak_6468|MACS_peak_6469	-19344|-29132
chr16	21204867	21223377	Ephb3	0	+	MACS_peak_6476|MACS_peak_6477	-17160|-17927
chr16	22245813	22266002	Tra2b	0	-	MACS_peak_6481	-6835
chr16	22381385	22439643	Etv5	0	-	MACS_peak_6485|MACS_peak_6486|MA	-27789|-26638|-3068
chr16	22920294	22939841	Fetub	0	+	MACS_peak_6488|MACS_peak_6488|MA	-17691|-19531|-17691
chr16	22951144	22961733	Hrg	0	+	MACS_peak_6488	13159
chr16	23224834	23360420	St6gal1	0	+	MACS_peak_6496	-147
chr16	23451857	23520663	Masp1	0	-	MACS_peak_6502	-25905
chr16	23965137	23988698	Bcl6	0	-	MACS_peak_6509	-11021
chr16	29579427	29652576	Opa1	0	+	MACS_peak_6522	-8955
chr16	30307778	30310865	Gp5	0	-	MACS_peak_6527|MACS_peak_6528|MA	2398|12131|18224
chr16	30269387	30283340	Lrrc15	0	-	MACS_peak_6527	29923
chr16	30955596	31081518	AI480653	0	-	MACS_peak_6531|MACS_peak_6532	25664|29102
chr16	31878895	31899105	Mfi2	0	+	MACS_peak_6534	6302
chr16	31959755	32003373	Senp5	0	-	MACS_peak_6535|MACS_peak_6536	-13642|15806
chr16	32179885	32187030	Bex6	0	+	MACS_peak_6538|MACS_peak_6539|MA	28420|27787|27460
chr16	32142910	32165562	Lrrc33	0	-	MACS_peak_6538|MACS_peak_6539|MA	-14097|-13464|-13137
chr16	32777504	32797521	Muc20	0	-	MACS_peak_6543|MACS_peak_6543	29365|29365
chr16	33185156	33243397	Osbpl11	0	+	MACS_peak_6548|MACS_peak_6549	-815|-20894
chr16	33251541	33299648	Snx4	0	+	MACS_peak_6550	22399
chr16	35983448	36036248	Kpna1	0	+	MACS_peak_6596	-25990
chr16	36792969	36828345	Eaf2	0	-	MACS_peak_6597|MACS_peak_6597|MA	3655|23209|19295|-27272|6530|12497
chr16	36828485	36872799	Iqcb1	0	+	MACS_peak_6597|MACS_peak_6599	-3515|-19155
chr16	36885096	36933169	Golgb1	0	+	MACS_peak_6601|MACS_peak_6604	3654|-2313
chr16	36935068	36963298	Hcls1	0	+	MACS_peak_6612|MACS_peak_6613|MA	9166|8832|6300|5662
chr16	38089086	38246165	Gsk3b	0	+	MACS_peak_6619	-1051
chr16	38085149	38089346	BC031361	0	-	MACS_peak_6619	791
chr16	38445478	38452769	Adprh	0	-	MACS_peak_6625	6162
chr16	38459012	38487014	Cd80	0	+	MACS_peak_6625	81
chr16	38396202	38433225	Pla1a	0	-	MACS_peak_6625	25706
chr16	38497924	38522747	4930455C	0	-	MACS_peak_6630	-28041
chr16	38562971	38591497	Tmem39a	0	+	MACS_peak_6631|MACS_peak_6632|MA	-10305|-12930|-19190|-22522|-28208
chr16	38525263	38550266	Ktelc1	0	-	MACS_peak_6631|MACS_peak_6632	23010|25635
chr16	43640767	43640850	Mir568	0	+	MACS_peak_6637|MACS_peak_6647|MA	29813|18090|2206
chr16	43648973	43664297	Tigit	0	-	MACS_peak_6656	-25736
chr16	44085516	44139132	Atp6v1a	0	-	MACS_peak_6662	-27417
chr16	44139921	44163477	Naa50	0	+	MACS_peak_6662	28206
chr16	4616465	4624946	Magmas	0	-	MACS_peak_6341|MACS_peak_6342	2698|15103
chr16	4639944	4651166	Vasn	0	+	MACS_peak_6341|MACS_peak_6342	12300|-105
chr16	4684069	4707690	Dnaja3	0	+	MACS_peak_6345|MACS_peak_6345	-3235|-3235
chr16	4626883	4679720	Coro7	0	-	MACS_peak_6345	7584
chr16	48283847	48294405	Dppa4	0	+	MACS_peak_6665|MACS_peak_6665	-27928|-27928
chr16	48310386	48319626	Dppa2	0	+	MACS_peak_6665	-1389
chr16	50191957	50432502	Bbx	0	-	MACS_peak_6685	-112
chr16	5013994	5050003	Glyr1	0	-	MACS_peak_6348|MACS_peak_6348|MA	-755|-755|20755|20755
chr16	5050160	5086378	Ubn1	0	+	MACS_peak_6348|MACS_peak_6349	912|-20598
chr16	52249108	52453110	Alcam	0	-	MACS_peak_6702	-328
chr16	55811490	55822251	Nfkbiz	0	-	MACS_peak_6704|MACS_peak_6704|MA	-221|-16724|-16724
chr16	55840065	55895392	Fam55c	0	-	MACS_peak_6705|MACS_peak_6705|MA	6636|6630|10326|10320
chr16	55924476	55934961	Cep97	0	-	MACS_peak_6706|MACS_peak_6706|MA	-29243|-29243|-29243|-29243
chr16	56015620	56029830	Pcnp	0	-	MACS_peak_6708	-21095
chr16	56033832	56037887	Rg9mtd1	0	-	MACS_peak_6708	-29152
chr16	57156777	57167445	Nit2	0	-	MACS_peak_6710	10647
chr16	57302112	57606980	2610528E	0	-	MACS_peak_6712	-46
chr16	58408647	58469858	Dcbld2	0	+	MACS_peak_6717	-16142
chr16	58670320	58680502	Cpox	0	+	MACS_peak_6719|MACS_peak_6720|MA	15435|-438|-4201|-9176
chr16	58635374	58638516	E330017A	0	-	MACS_peak_6719	16369
chr16	58717547	58718837	Gpr15	0	-	MACS_peak_6723	14796
chr16	58760265	58761353	Olfr172	0	-	MACS_peak_6723	-27720
chr16	58728156	58734350	Cldnd1	0	+	MACS_peak_6723	-5477
chr16	62854159	62929166	Pros1	0	+	MACS_peak_6727|MACS_peak_6728	27042|23560
chr16	62793514	62846866	Arl13b	0	-	MACS_peak_6727|MACS_peak_6728	-19749|-16267
chr16	62814501	62822548	Stx19	0	+	MACS_peak_6727|MACS_peak_6728	-12616|-16098
chr16	78301935	78340996	Cxadr	0	+	MACS_peak_6747|MACS_peak_6747|MA	-23548|-23548|-27370|-27370
chr16	78540582	78576913	D16Ertd47	0	-	MACS_peak_6758	-680
chr16	8830192	8859017	1810013L	0	+	MACS_peak_6375	-14886
chr16	90220986	90226569	Sod1	0	+	MACS_peak_6796	-28897
chr16	90751772	90810658	Urb1	0	-	MACS_peak_6797	-28308

chr16	90936345	91011340	Synj1	0	-	MACS_peak_6798|MACS_peak_6798	11619|11406
chr16	91014281	91044624	Gcfc1	0	-	MACS_peak_6798	-21665
chr16	91011493	91016748	4930404I0	0	+	MACS_peak_6798	-11466
chr16	91621641	91647217	Gart	0	-	MACS_peak_6806	4744
chr16	91648068	91663563	Son	0	+	MACS_peak_6806|MACS_peak_6806	-3893|-3893
chr16	92601710	92697573	Runx1	0	-	MACS_peak_6812|MACS_peak_6812	-8274|-8274
chr16	93608081	93610594	Cbr1	0	+	MACS_peak_6815	-998
chr16	93583705	93604060	Setd4	0	-	MACS_peak_6815	5019
chr16	94586059	94592317	Pigp	0	-	MACS_peak_6823|MACS_peak_6823|MA	14703|14398|14863|15083|15083|14398
chr16	94592345	94690828	Ttc3	0	+	MACS_peak_6823	-14675
chr16	95924013	95942654	Ets2	0	+	MACS_peak_6828|MACS_peak_6829|MA	-149|-7419|-9321|-13764
chr16	96201541	96212510	Psmg1	0	-	MACS_peak_6835	26747
chr16	96223154	96304035	Brwd1	0	-	MACS_peak_6838|MACS_peak_6838|MA	-21371|-21371|-21371|-17576|-17576|-17576|-16321|-16321|-16321
chr16	96343194	96349332	Hmgn1	0	-	MACS_peak_6842	-13774
chr16	98168598	98183786	Zfp295	0	-	MACS_peak_6844|MACS_peak_6844|MA	-16744|-16744|-16744
chr16	98076816	98144243	C2cd2	0	-	MACS_peak_6844	22799
chr17	12841740	12868704	Slc22a1	0	-	MACS_peak_6896|MACS_peak_6897|MA	17340|18244|21599|22749|24600
chr17	12934176	13008423	Airn	0	+	MACS_peak_6913|MACS_peak_6913|MA	27258|27258|27258|27258|25350|25350|25350|25350|24667|24667|24667|24667|-28224|-28224|-28224|-28224
chr17	12875271	12962572	Igf2r	0	-	MACS_peak_6922	-172
chr17	13547437	13570089	Gm9880	0	+	MACS_peak_6924	22185
chr17	15080188	15082944	1600012H	0	+	MACS_peak_6937|MACS_peak_6937|MA	-11669|-11669|-11669|-11669|-11919|-11919|-11919|-11919
chr17	15101152	15115776	Gm3448	0	-	MACS_peak_6937|MACS_peak_6938|MA	-23919|-23669|5157|-26215|3659|-27716|3939|-27436|4793|-26582|9720|-21655|4280
chr17	15101152	15115776	Gm3417	0	-	MACS_peak_6937|MACS_peak_6938|MA	-23919|-23669|5157|-26215|3659|-27716|3939|-27436|4793|-26582|9720|-21655|4280
chr17	15115827	15128541	9030025P	0	+	MACS_peak_6937|MACS_peak_6938|MA	23970|23720|-5106
chr17	15082001	15098237	Phf10	0	-	MACS_peak_6937|MACS_peak_6938|MA	-6380|-6130|22696
chr17	14955678	15080129	Wdr27	0	-	MACS_peak_6937|MACS_peak_6938	11728|11978
chr17	15084742	15085828	LOC10674	0	-	MACS_peak_6937|MACS_peak_6938	6029|6279
chr17	15146803	15159413	Gm3435	0	+	MACS_peak_6941|MACS_peak_6945|MA	25870|-4004|-4284|-5138|-10065
chr17	15178575	15201196	2210404J1	0	+	MACS_peak_6945|MACS_peak_6946|MA	27768|27488|26634|21707|7219|-4228
chr17	15164117	15178523	Tcte3	0	-	MACS_peak_6945|MACS_peak_6945|MA	-27716|-27716|-27436|-27436|-26582|-26582|-21655|-21655|4280|4280
chr17	15504317	15512787	Dll1	0	-	MACS_peak_6956|MACS_peak_6957|MA	-7996|-7291|-6889|-5524|-5217|-328
chr17	15533209	15570545	Fam120b	0	+	MACS_peak_6956|MACS_peak_6956|MA	28418|28374|27713|27669|27311|27267|25946|25902|25639|25595|20750|20706
chr17	15487776	15491425	Gm10510	0	-	MACS_peak_6956|MACS_peak_6957|MA	13366|14071|14473|15838|16145|21034
chr17	15658538	15664265	Pdcd2	0	-	MACS_peak_6966|MACS_peak_6968	-4437|17789
chr17	15612684	15635240	Psmb1	0	-	MACS_peak_6966	24588
chr17	15636851	15654391	Tbp	0	+	MACS_peak_6966	-22977
chr17	15680043	15700287	Prdm9	0	-	MACS_peak_6968	-18233
chr17	17201066	17226593	BC018101	0	+	MACS_peak_6981|MACS_peak_6981	24628|24628
chr17	21704009	21725649	BC049807	0	+	MACS_peak_7019	9817
chr17	21672502	21699565	Zfp52	0	+	MACS_peak_7019	-21690
chr17	21844707	21862603	Zfp760	0	+	MACS_peak_7021|MACS_peak_7022|MA	-14483|-15487|-17219
chr17	21870690	21885935	Zfp229	0	+	MACS_peak_7021|MACS_peak_7022|MA	11500|10496|8764
chr17	22983663	23004101	A630033E	0	-	MACS_peak_7027|MACS_peak_7027	-5326|-5326
chr17	23805585	23810737	Thoc6	0	-	MACS_peak_7033	2843
chr17	23812413	23814416	Tnfrsf12a	0	-	MACS_peak_7033|MACS_peak_7033	-836|-836
chr17	23816331	23819411	Cldn6	0	+	MACS_peak_7033	2751
chr17	23819551	23820993	Cldn9	0	-	MACS_peak_7033	-7413
chr17	23797489	23805586	Ccdc64b	0	+	MACS_peak_7033	-16091
chr17	23810574	23812491	Hcfc1r1	0	+	MACS_peak_7033	-3006
chr17	24184686	24210452	Kctd5	0	-	MACS_peak_7034	24606
chr17	24401223	24484197	Abca17	0	-	MACS_peak_7035|MACS_peak_7036|MA	18890|27134|29473
chr17	24488990	24547146	Abca3	0	+	MACS_peak_7035|MACS_peak_7035|MA	-14097|-14097|-22341|-22341|-24680|-24680|-25734|-25734
chr17	24551619	24562842	Rnps1	0	+	MACS_peak_7040|MACS_peak_7040|MA	28354|28354|28354|7001|7001|7001|4901|4901|4901
chr17	24546425	24551458	D330041H	0	-	MACS_peak_7040|MACS_peak_7044|MA	-28193|-6840|-4740
chr17	24563627	24576261	Dci	0	+	MACS_peak_7044|MACS_peak_7045|MA	19009|16909|-24786
chr17	24580723	24592256	E4f1	0	-	MACS_peak_7047	-3843
chr17	24610496	24616023	Mlst8	0	-	MACS_peak_7047	-27610
chr17	24577712	24580046	Dnase1l2	0	-	MACS_peak_7047	8367
chr17	24607417	24608541	Pgp	0	+	MACS_peak_7047	19004
chr17	24610828	24612414	9930021D	0	+	MACS_peak_7047	22415
chr17	24645794	24664883	Traf7	0	-	MACS_peak_7048|MACS_peak_7048|MA	22387|22387|22387
chr17	24686894	24733459	Pkd1	0	+	MACS_peak_7048	-376
chr17	24665999	24670692	Rab26	0	-	MACS_peak_7048	16578
chr17	24732881	24769574	Tsc2	0	-	MACS_peak_7049|MACS_peak_7049|MA	-2709|-2709|-1679|-1679|2171|2171|17493|17493
chr17	24794274	24795370	Npw	0	-	MACS_peak_7049|MACS_peak_7050|MA	-28505|-27475|-23625|-8303
chr17	24769626	24775782	Nthl1	0	+	MACS_peak_7049|MACS_peak_7050|MA	2761|1731|-2119|-17441
chr17	24776226	24787250	Slc9a3r2	0	-	MACS_peak_7049|MACS_peak_7049|MA	-20385|-15037|-19355|-14007|-15505|-10157|-183|5165
chr17	24806696	24818961	Zfp598	0	+	MACS_peak_7052	19629
chr17	24987611	25001393	Hagh	0	+	MACS_peak_7055|MACS_peak_7055	-6007|-6184
chr17	25015714	25018953	Igfals	0	+	MACS_peak_7055|MACS_peak_7057|MA	22096|-5070|-24312|-25966
chr17	25019555	25023295	Nubp2	0	-	MACS_peak_7055|MACS_peak_7057|MA	-29677|-2511|16731|18385|27236
chr17	24985840	24987247	Fahd1	0	-	MACS_peak_7055	6371
chr17	25029097	25032032	Eme2	0	-	MACS_peak_7057|MACS_peak_7058|MA	-11248|7994|9648|18499
chr17	25023618	25029092	Spsb3	0	+	MACS_peak_7057|MACS_peak_7057|MA	2834|2834|3641|-16408|-16408|-15601|-18062|-18062|-17255|-26913|-26913|-26106
chr17	25033444	25034472	Nme3	0	+	MACS_peak_7057|MACS_peak_7058|MA	12660|-6582|-8236|-17087
chr17	25032064	25033218	Mrps34	0	+	MACS_peak_7057|MACS_peak_7058|MA	11280|-7962|-9616|-18467
chr17	25034451	25073922	Mapk8ip3	0	-	MACS_peak_7061|MACS_peak_7061|MA	-23391|-23391|-23391|-23391|-23391|-23391|-23391
chr17	25079414	25097568	Hn1l	0	-	MACS_peak_7063|MACS_peak_7064	8370|12928
chr17	25299405	25308858	Ccdc154	0	+	MACS_peak_7067	7037
chr17	25270338	25299044	Clcn7	0	+	MACS_peak_7067	-22030
chr17	25321505	25324606	BC003965	0	+	MACS_peak_7067	29137
chr17	25885557	25891272	Msln	0	-	MACS_peak_7069|MACS_peak_7079	-25729|28737
chr17	25854116	25856047	Gng13	0	+	MACS_peak_7069	-11427
chr17	25864695	25868401	Rpusd1	0	+	MACS_peak_7069	-848
chr17	25855974	25864360	Chtf18	0	-	MACS_peak_7069	1183
chr17	25872984	25885275	Mslnl	0	+	MACS_peak_7069	7441
chr17	25946029	25958208	Fbxl16	0	+	MACS_peak_7079|MACS_peak_7080|MA	26020|-15102|-17209
chr17	25923524	25927458	Ccdc78	0	+	MACS_peak_7079	3515
chr17	25910720	25920277	Narfl	0	+	MACS_peak_7079	-9289
chr17	25919769	25922278	Haghl	0	-	MACS_peak_7079	-2269
chr17	25931515	25933990	Metrn	0	-	MACS_peak_7079|MACS_peak_7080|MA	-13981|27141|29248
chr17	25927450	25929229	Fam173a	0	-	MACS_peak_7079	-9220
chr17	25967580	25970306	Stub1	0	-	MACS_peak_7080|MACS_peak_7081	-9175|-7068
chr17	25965987	25968787	Jmjd8	0	+	MACS_peak_7080|MACS_peak_7081	4856|2749
chr17	25971409	25974072	Rhbdl1	0	-	MACS_peak_7080|MACS_peak_7081	-12941|-10834
chr17	25975782	25981796	Rhot2	0	-	MACS_peak_7080|MACS_peak_7081	-20665|-18558
chr17	25960571	25965675	Wdr24	0	+	MACS_peak_7080|MACS_peak_7081	-560|-2667
chr17	26014572	26017803	Wfikkn1	0	-	MACS_peak_7082|MACS_peak_7083|MA	-5240|-3823|3829
chr17	25981678	25998460	Wdr90	0	-	MACS_peak_7082|MACS_peak_7083|MA	14103|15520|23172
chr17	26000642	26005683	Fam195a	0	-	MACS_peak_7082|MACS_peak_7083|MA	6880|8297|15949
chr17	26012444	26014108	0610011F	0	+	MACS_peak_7082|MACS_peak_7083|MA	-119|-1536|-9188
chr17	26038800	26039172	Gm10012	0	-	MACS_peak_7082|MACS_peak_7083|MA	-26609|-25192|-17540
chr17	26339906	26348269	Rgs11	0	+	MACS_peak_7088	-17469
chr17	26332943	26336032	Pdia2	0	-	MACS_peak_7088	21343
chr17	26336127	26338295	Arhgdig	0	-	MACS_peak_7088	19080
chr17	26349657	26381086	Itfg3	0	-	MACS_peak_7088|MACS_peak_7089|MA	-23711|-60|10015
chr17	26389854	26417668	Luc7l	0	+	MACS_peak_7089|MACS_peak_7089|MA	8828|8828|-1247|-1247
chr17	27070071	27074835	Phf1	0	+	MACS_peak_7108|MACS_peak_7109	-1150|-2856
chr17	27074916	27076413	Cuta	0	-	MACS_peak_7108|MACS_peak_7108|MA	-5192|-5202|-3486|-3496
chr17	27054035	27069524	Kifc1	0	+	MACS_peak_7108|MACS_peak_7109	-17186|-18892
chr17	27194248	27259168	Itpr3	0	+	MACS_peak_7115	-29613
chr17	27259609	27270836	2900010M	0	-	MACS_peak_7129|MACS_peak_7130|MA	-29978|-29347|-26539|-26121|-21952|-20309|-20022|-19442|-18998|-18812|-18635|-18166|-17905|-17650|-17197|-16608|-16032|-15489|-
chr17	27326544	27341383	Lemd2	0	-	MACS_peak_7156|MACS_peak_7157|MA	-11468|-11284|-10527|-105
chr17	27280915	27304709	Ip6k3	0	-	MACS_peak_7156|MACS_peak_7157|MA	25206|25390|26147
chr17	27700713	27702672	AI413582	0	-	MACS_peak_7161	-6128
chr17	27693518	27700617	Hmga1	0	+	MACS_peak_7161|MACS_peak_7161|MA	-3026|-3026|-3026|-3026|-2996|-3026|-3026|-3026|-3026|-3026|-3026|-3026|-3026|-2996
chr17	27693597	27700615	Hmga1-rs1	0	+	MACS_peak_7161|MACS_peak_7161	-2947|-2947
chr17	27993451	28036985	Uhrf1bp1	0	+	MACS_peak_7164	-23725
chr17	28038072	28044669	Taf11	0	-	MACS_peak_7164|MACS_peak_7165|MA	-27493|-20928|-11561|-9173
chr17	28046284	28199581	Anks1	0	+	MACS_peak_7164|MACS_peak_7165|MA	29108|22543|13176|10788
chr17	28203691	28217584	Tcp11	0	-	MACS_peak_7171|MACS_peak_7171|MA	-21297|-21242|-19523|-19468
chr17	28279470	28308290	Scube3	0	+	MACS_peak_7173	-14613

chr17	28314362	28342830	Zfp523	0	+	MACS_peak_7173	20279
chr17	28369698	28438414	Ppard	0	+	MACS_peak_7175	-4299
chr17	28344722	28365553	Def6	0	+	MACS_peak_7175	-29275
chr17	28450474	28463519	Fance	0	+	MACS_peak_7176|MACS_peak_7176|MA	14721|14721|14721|14721
chr17	28465415	28467978	Rpl10a	0	+	MACS_peak_7176	29662
chr17	29169617	29180311	Srsf3	0	+	MACS_peak_7185|MACS_peak_7186	-6965|-27002
chr17	29194418	29215906	LOC10049	0	-	MACS_peak_7186	-19287
chr17	31038811	31073455	Glp1r	0	+	MACS_peak_7199	-23745
chr17	31091627	31147655	Umodl1	0	+	MACS_peak_7199|MACS_peak_7200|MA	29071|17736|15970|15426
chr17	32257837	32303797	Notch3	0	-	MACS_peak_7227|MACS_peak_7232|MA	-22368|-15500|-8834
chr17	32458368	32487522	Akap8l	0	-	MACS_peak_7240|MACS_peak_7243	-28640|12292
chr17	32440620	32458098	Akap8	0	-	MACS_peak_7240	784
chr17	32490996	32525895	Wiz	0	-	MACS_peak_7243|MACS_peak_7243|MA	-26081|-24948|-26570
chr17	33042014	33054023	Cyp4f14	0	-	MACS_peak_7246	13516
chr17	33061632	33084306	Cyp4f13	0	-	MACS_peak_7246	-16767
chr17	34132956	34137278	H2-K1	0	-	MACS_peak_7263|MACS_peak_7264	29582|29837
chr17	34165210	34168635	Slc39a7	0	-	MACS_peak_7263|MACS_peak_7263|MA	-1775|-1775|-1520|-1520|-575|-575|5203|5203
chr17	34157738	34161625	Ring1	0	-	MACS_peak_7263|MACS_peak_7264|MA	5235|5490|6435|12213
chr17	34176381	34203187	Col11a2	0	+	MACS_peak_7263|MACS_peak_7264|MA	9521|9266|8321|2543
chr17	34168796	34175344	Rxrb	0	+	MACS_peak_7263|MACS_peak_7264|MA	1936|1681|736|-5042
chr17	34162977	34165000	H2-Ke6	0	-	MACS_peak_7263|MACS_peak_7264|MA	1860|2115|3060|8838
chr17	34161927	34162037	Mir219-1	0	-	MACS_peak_7263|MACS_peak_7264|MA	4823|5078|6023|11801
chr17	34248963	34257328	Brd2	0	-	MACS_peak_7272|MACS_peak_7272|MA	-5909|-7273|-4645|-6009
chr17	34229323	34232179	H2-Oa	0	+	MACS_peak_7272|MACS_peak_7274	-22096|-23360
chr17	34272615	34275480	H2-DMa	0	+	MACS_peak_7272|MACS_peak_7274	21196|19932
chr17	34282360	34288040	H2-DMb2	0	+	MACS_peak_7274	29677
chr17	35093373	35096183	Hspa1b	0	-	MACS_peak_7288|MACS_peak_7289|MA	-27798|-27275|11677|18439
chr17	35068197	35074242	Neu1	0	+	MACS_peak_7288|MACS_peak_7289	-188|-711
chr17	35051410	35067381	Slc44a4	0	+	MACS_peak_7288|MACS_peak_7289	-16975|-17498
chr17	35087182	35089416	1110038B	0	-	MACS_peak_7288|MACS_peak_7288|MA	-21031|-21028|-20508|-20505|18444|18447|25206|25209
chr17	35087894	35087953	Snord52	0	-	MACS_peak_7288|MACS_peak_7289|MA	-19568|-19045|19907|26669
chr17	35118804	35122838	Lsm2	0	+	MACS_peak_7292|MACS_peak_7292|MA	10944|11214|4182|4452|-23441|-23171
chr17	35106303	35109101	Hspa1a	0	-	MACS_peak_7292|MACS_peak_7293	-1241|5521
chr17	35137851	35153274	Vars	0	+	MACS_peak_7292|MACS_peak_7293|MA	29991|23229|-4394|-12349|-12954|-14134
chr17	35109647	35116173	Hspa1l	0	+	MACS_peak_7292|MACS_peak_7293	1787|-4975
chr17	35162921	35164961	Ng23	0	-	MACS_peak_7294|MACS_peak_7294|MA	-22716|-22716|-14761|-14761|-14156|-14156|-12976|-12976
chr17	35153523	35163686	D17H6S56	0	+	MACS_peak_7294|MACS_peak_7297|MA	11278|3323|2718|1538
chr17	35199637	35203129	AU023871	0	-	MACS_peak_7300|MACS_peak_7300|MA	-7005|-7005|-5538|-5538|-4632|-4632
chr17	35165551	35183668	Msh5	0	-	MACS_peak_7300|MACS_peak_7300|MA	12456|12573|13923|14040|14829|14946
chr17	35217482	35222540	Ly6g6f	0	-	MACS_peak_7300|MACS_peak_7302|MA	-26416|-24949|-24043
chr17	35195979	35199044	Ddah2	0	+	MACS_peak_7300|MACS_peak_7300|MA	-145|-145|-1612|-1612|-2518|-2518
chr17	35204269	35206993	Ly6g6c	0	+	MACS_peak_7300|MACS_peak_7302|MA	8145|6678|5772
chr17	35213886	35215749	Ly6g6e	0	+	MACS_peak_7300|MACS_peak_7302|MA	17762|16295|15389
chr17	35187187	35195664	Clic1	0	+	MACS_peak_7300|MACS_peak_7302|MA	-8937|-10404|-11310
chr17	35208292	35211409	Ly6g6d	0	-	MACS_peak_7300|MACS_peak_7302|MA	-15285|-13818|-12912
chr17	35201381	35202541	G6b	0	+	MACS_peak_7300|MACS_peak_7302|MA	5257|3790|2884
chr17	35226235	35239932	Bat5	0	+	MACS_peak_7302|MACS_peak_7303	28644|27738
chr17	35258440	35261760	Bat4	0	+	MACS_peak_7305|MACS_peak_7305|MA	-1753|-297|-2079|-623|-5529|-4073
chr17	35253139	35258392	Csnk2b	0	-	MACS_peak_7305|MACS_peak_7306|MA	1801|2127|5577
chr17	35265941	35268697	Apom	0	-	MACS_peak_7305|MACS_peak_7306|MA	-8504|-8178|-4728
chr17	35264059	35264941	D17H6S53	0	+	MACS_peak_7305|MACS_peak_7306|MA	3866|3540|90
chr17	35272186	35284181	Bat3	0	+	MACS_peak_7305|MACS_peak_7306|MA	11993|11667|8217
chr17	35250889	35252345	Ly6g5b	0	-	MACS_peak_7305|MACS_peak_7306|MA	7848|8174|11624
chr17	35245244	35248898	Ly6g5c	0	+	MACS_peak_7305|MACS_peak_7306|MA	-14949|-15275|-18725
chr17	35516561	35521619	H2-gs10	0	+	MACS_peak_7315	-21382
chr17	35531043	35532577	H2-Q8	0	+	MACS_peak_7315	-6900
chr17	35561821	35565306	H2-Q6	0	+	MACS_peak_7315	23878
chr17	35772699	35780640	Dpcr1	0	-	MACS_peak_7328	23565
chr17	35786652	35787514	Sfta2	0	+	MACS_peak_7328	-17553
chr17	35804683	35810627	Gtf2h4	0	-	MACS_peak_7328|MACS_peak_7335	-6422|15150
chr17	35792579	35804537	Vars2	0	-	MACS_peak_7328|MACS_peak_7335	-332|21240
chr17	35818511	35841084	Ddr1	0	-	MACS_peak_7335|MACS_peak_7335	-15307|-15307
chr17	35978442	35996615	Mdc1	0	+	MACS_peak_7343|MACS_peak_7344|MA	16862|16608|10820
chr17	35960301	35969732	Flot1	0	+	MACS_peak_7343|MACS_peak_7344|MA	-1279|-1533|-7321
chr17	35970864	35975246	Tubb5	0	-	MACS_peak_7343|MACS_peak_7344|MA	-13666|-13412|-7624
chr17	35958657	35959856	Ier3	0	+	MACS_peak_7343|MACS_peak_7344|MA	-2923|-3177|-8965
chr17	36029621	36034323	2310061I0	0	-	MACS_peak_7350|MACS_peak_7351|MA	4067|20122|26704
chr17	36034540	36047013	2610110G	0	-	MACS_peak_7350|MACS_peak_7350|MA	-8623|-8623|-8623|7432|7432|7432|14014|14014|14014|17895|17895|17895
chr17	36054140	36069226	Ppp1r10	0	+	MACS_peak_7350|MACS_peak_7350|MA	15750|15465|-305|-590|-6887|-7172|-10768|-11053
chr17	36047329	36053314	Mrps18b	0	-	MACS_peak_7350|MACS_peak_7351|MA	-14924|1131|7713|11594
chr17	36016722	36029611	Dhx16	0	+	MACS_peak_7350	-21668
chr17	36054833	36054914	Mir1894	0	+	MACS_peak_7350|MACS_peak_7351|MA	16443|388|-6194|-10075
chr17	36084098	36090260	Gm8801	0	+	MACS_peak_7351|MACS_peak_7352|MA	29653|23071|19190
chr17	36114702	36116412	Prr3	0	-	MACS_peak_7355|MACS_peak_7355|MA	15556|15556|15198|16000
chr17	36116899	36126407	Gnl1	0	+	MACS_peak_7355	-15069
chr17	36142639	36157505	H2-T24	0	-	MACS_peak_7355	-25537
chr17	36093763	36106695	Abcf1	0	-	MACS_peak_7355	25273
chr17	36131448	36175119	A930015D	0	+	MACS_peak_7355	-520
chr17	36997635	37004132	Trim15	0	-	MACS_peak_7367|MACS_peak_7367	-10497|-10520
chr17	36974085	36996325	Trim26	0	+	MACS_peak_7367	-19550
chr17	37006518	37014776	Trim10	0	+	MACS_peak_7367	12883
chr17	37147684	37160343	Mog	0	-	MACS_peak_7370	25140
chr17	37182910	37211250	Gabbr1	0	+	MACS_peak_7370	-2573
chr17	44740949	44873597	Runx2	0	-	MACS_peak_7395	-11824
chr17	45767152	45796258	Capn11	0	-	MACS_peak_7404|MACS_peak_7405	4283|18657
chr17	45823320	45835444	Mrpl14	0	+	MACS_peak_7404|MACS_peak_7405	22779|8405
chr17	45797125	45823167	Tmem63b	0	-	MACS_peak_7404|MACS_peak_7405	-22626|-8252
chr17	46297980	46306318	Gtpbp2	0	+	MACS_peak_7411|MACS_peak_7411|MA	-747|-747|-983|-983|-3724|-3724|-4106|-4106|-4485|-4485|-4855|-4855|-5345|-5345|-6313|-6313|-6802|-6802|-7663|-7663
chr17	46284334	46290500	Mad2l1bp	0	-	MACS_peak_7411|MACS_peak_7412|MA	8227|8463|11204|11586|11965|12335|12825|13793|14282|15143
chr17	46266225	46281147	Rsph9	0	-	MACS_peak_7411|MACS_peak_7412|MA	17580|17816|20557|20939|21318|21688|22178|23146|23635|24496
chr17	46783196	46787081	Mrpl2	0	+	MACS_peak_7440|MACS_peak_7443	4025|-26138
chr17	46787286	46801313	Cul7	0	+	MACS_peak_7440|MACS_peak_7443|MA	8115|-22048|-27682
chr17	46767579	46782093	Klc4	0	-	MACS_peak_7440|MACS_peak_7443	-2922|27241
chr17	46701399	46766453	Ptk7	0	-	MACS_peak_7440	12718
chr17	46811501	46817879	Klhdc3	0	-	MACS_peak_7443|MACS_peak_7443|MA	-8545|-8545|-2911|-2911
chr17	46818085	46820054	Mea1	0	+	MACS_peak_7443|MACS_peak_7444	8751|3117
chr17	46804404	46811204	Rrp36	0	-	MACS_peak_7443|MACS_peak_7444	-1870|3764
chr17	46819940	46841951	Ppp2r5d	0	-	MACS_peak_7444|MACS_peak_7447	-26983|16803
chr17	46862612	46866114	Gnmt	0	-	MACS_peak_7447	-7360
chr17	46848411	46862490	Pex6	0	+	MACS_peak_7447	-10343
chr17	46935064	46968362	BC032203	0	-	MACS_peak_7451	-19439
chr17	46910855	46919605	Rpl7l1	0	-	MACS_peak_7451	29318
chr17	47047433	47061875	Prph2	0	+	MACS_peak_7453	-18954
chr17	47065240	47147481	Ubr2	0	-	MACS_peak_7463|MACS_peak_7463|MA	-29132|-29132|-205|-205
chr17	47641999	47736637	Ccnd3	0	+	MACS_peak_7467|MACS_peak_7467	3686|3686
chr17	47624998	47639236	Taf8	0	-	MACS_peak_7467	-923
chr17	48526209	48549145	Nfya	0	-	MACS_peak_7469|MACS_peak_7469|MA	-21670|-21670|-17854|-17854
chr17	48499240	48506471	Treml1	0	+	MACS_peak_7469	-28235
chr17	48549404	48556591	AI314976	0	+	MACS_peak_7469|MACS_peak_7470	21929|18113
chr17	49567688	49594755	Mocs1	0	+	MACS_peak_7472|MACS_peak_7472|MA	-11139|-11139|-21136|-21136
chr17	53723450	53812046	Kat2b	0	+	MACS_peak_7485	-14419
chr17	56551848	56615903	Ptprs	0	-	MACS_peak_7522|MACS_peak_7525	-22224|-85
chr17	56702364	56724006	Safb2	0	-	MACS_peak_7526|MACS_peak_7527	29204|29871
chr17	56724404	56745716	Safb	0	+	MACS_peak_7526|MACS_peak_7527|MA	-28806|-29473|-29879
chr17	56752817	56753669	Rpl36	0	+	MACS_peak_7526|MACS_peak_7527|MA	-393|-1060|-1466|-1938|-2182|-2605|-2797|-3001|-4488|-4993|-5918|-6628|-6896|-8646|-9099|-9788|-13289
chr17	56753721	56766326	Lonp1	0	-	MACS_peak_7526|MACS_peak_7527|MA	-13116|-12449|-12043|-11571|-11327|-10904|-10712|-10508|-9021|-8516|-7591|-6881|-6613|-4863|-4410|-3721|-220
chr17	56746874	56749194	2410015M	0	-	MACS_peak_7526|MACS_peak_7527|MA	4016|4683|5089|5561|5805|6228|6420|6624|8111|8616|9541|10251|10519|12269|12722|13411|16912
chr17	56767565	56803877	Tmem146	0	+	MACS_peak_7526|MACS_peak_7527|MA	14355|13688|13282|12810|12566|12143|11951|11747|10260|9755|8830|8120|7852|6102|5649|4960|1459
chr17	6270474	6305783	Tmem181a	0	+	MACS_peak_6867	-20134
chr17	6310546	6317474	Dynlt1a	0	-	MACS_peak_6867|MACS_peak_6868|MA	-26866|-13188|-11504|-10257|-9302
chr17	62952305	63230666	Efna5	0	-	MACS_peak_7562|MACS_peak_7562	908|908
chr17	6429259	6435443	Dynlt1b	0	+	MACS_peak_6875	-13478

chr17	6438149	6450142	Tmem181b	0	-	MACS_peak_6875	-7405
chr17	64950988	65104450	Man2a1	0	+	MACS_peak_7574	291
chr17	66272405	66300527	Twsg1	0	-	MACS_peak_7585|MACS_peak_7586|MA	-21508|18201|19072
chr17	6648836	6655064	Dynlt1f	0	-	MACS_peak_6878	-12438
chr17	6648841	6654956	Dynlt1c	0	-	MACS_peak_6878	-12330
chr17	6635807	6644102	Tmem181c	0	+	MACS_peak_6878	-6819
chr17	6635807	6644102	Tmem181c	0	+	MACS_peak_6878	-6819
chr17	71193546	71202872	Tgif1	0	-	MACS_peak_7625|MACS_peak_7625|MA	-2019|-221|1723|4736|303
chr17	7109064	7152705	Rsph3b	0	-	MACS_peak_6881	8493
chr17	7183208	7202542	Rnaset2b	0	+	MACS_peak_6881|MACS_peak_6882|MA	22010|-149|-19161
chr17	7159313	7165505	Tagap1	0	-	MACS_peak_6881|MACS_peak_6882	-4307|17852
chr17	71601515	71660305	Emilin2	0	-	MACS_peak_7641|MACS_peak_7642	-758|-183
chr17	71965554	72008371	Wdr43	0	+	MACS_peak_7643	17880
chr17	71906366	71948101	Trmt61b	0	-	MACS_peak_7643|MACS_peak_7643|MA	-427|-427|53
chr17	7229891	7291946	Gm1604b	0	+	MACS_peak_6884	27522
chr17	73267644	73291286	Lbh	0	+	MACS_peak_7647	-23103
chr17	74794971	74823569	Slc30a6	0	+	MACS_peak_7686	6354
chr17	79053303	79135900	Strn	0	-	MACS_peak_7738|MACS_peak_7739	-91|28841
chr17	79152245	79234721	Heatr5b	0	-	MACS_peak_7740|MACS_peak_7741	-9067|-1218
chr17	79234855	79247648	Ccdc75	0	+	MACS_peak_7740|MACS_peak_7741	9201|1352
chr17	79249844	79251884	Gm6548	0	-	MACS_peak_7740|MACS_peak_7741	-26230|-18381
chr17	79451245	79489583	Qpct	0	+	MACS_peak_7746|MACS_peak_7747	-27616|-29908
chr17	80623828	80627837	Gemin6	0	+	MACS_peak_7751	-26865
chr17	80689551	80696814	Morn2	0	+	MACS_peak_7754	29896
chr17	80771196	80772882	Gm10190	0	-	MACS_peak_7759	24533
chr17	8138478	8172421	Rsph3a	0	+	MACS_peak_6885	11076
chr17	8118864	8127761	Tagap	0	+	MACS_peak_6885	-8538
chr17	83614622	83624409	Pkdcc	0	+	MACS_peak_7768	-9223
chr17	84230693	84246130	Haao	0	-	MACS_peak_7773	-25294
chr17	84589395	84865548	Thada	0	-	MACS_peak_7775	-1768
chr17	85025838	85054904	Dync2li1	0	+	MACS_peak_7777	25770
chr17	85427686	85463581	Slc3a1	0	+	MACS_peak_7778	11398
chr17	85462818	85489607	Prepl	0	-	MACS_peak_7780|MACS_peak_7780|MA	-1634|-1634|-1641|-1634
chr17	85490039	85857920	1700106N	0	+	MACS_peak_7780	2066
chr17	87682225	87781110	Ttc7	0	+	MACS_peak_7787	-9981
chr17	87653782	87665287	Mcfd2	0	-	MACS_peak_7787|MACS_peak_7787	26919|26919
chr17	87674225	87682154	4833418N	0	-	MACS_peak_7787	10052
chr17	88374389	88390223	Msh6	0	+	MACS_peak_7788|MACS_peak_7789	-16495|-27809
chr17	95126419	95149232	Mettl4	0	-	MACS_peak_7799	-14475
chr17	95149439	95174469	2700099C	0	+	MACS_peak_7799	14682
chr18	10725622	10812215	Mib1	0	+	MACS_peak_7871	-21775
chr18	11052507	11085633	Gata6	0	+	MACS_peak_7875	-10573
chr18	12327238	12348478	3110002H	0	+	MACS_peak_7886|MACS_peak_7888|MA	-10809|-25450|-26406|-29824
chr18	12348202	12394895	Npc1	0	-	MACS_peak_7899|MACS_peak_7900|MA	-24712|-24153|-17135
chr18	12410865	12464229	Ankrd29	0	-	MACS_peak_7906	-15764
chr18	21159841	21186724	Rnf138	0	+	MACS_peak_7922|MACS_peak_7922|MA	-7471|-7471|-26634|-26634
chr18	34936661	34999024	Kdm3b	0	+	MACS_peak_7966	-26741
chr18	35000311	35007109	Reep2	0	+	MACS_peak_7968|MACS_peak_7969|MA	20819|17098|6374
chr18	35020860	35024610	Egr1	0	+	MACS_peak_7970	26923
chr18	35097068	35114005	Hspa9	0	-	MACS_peak_7975|MACS_peak_7976|MA	-14112|-13455|-7775|-6385|-3050|-94
chr18	35062438	35091657	Etf1	0	-	MACS_peak_7975|MACS_peak_7976|MA	8236|8893|14573|15963|19298|22254
chr18	35368662	35374678	Lrrtm2	0	-	MACS_peak_7985	24412
chr18	35774258	35786881	Slc23a1	0	-	MACS_peak_7996	-16277
chr18	35758320	35776839	Paip2	0	+	MACS_peak_7996	-12284
chr18	35806919	35809021	2010001M	0	-	MACS_peak_7998|MACS_peak_7999	21926|22306
chr18	35819669	35821840	Spata24	0	-	MACS_peak_7998|MACS_peak_7998|MA	9107|9107|9487|9487
chr18	36664580	36675459	Hbegf	0	-	MACS_peak_8002|MACS_peak_8003|MA	-10153|-8279|-3158|-632|-173
chr18	36687805	36704262	Slc4a9	0	+	MACS_peak_8002|MACS_peak_8003|MA	22499|20625|15504|12978|12519
chr18	36926183	36942859	Hars	0	-	MACS_peak_8008	-27472
chr18	36884720	36886308	Cd14	0	-	MACS_peak_8008	29079
chr18	36902019	36904202	Ndufa2	0	-	MACS_peak_8008	11185
chr18	36904309	36917293	Ik	0	+	MACS_peak_8008	-11078
chr18	36919892	36923362	Wdr55	0	+	MACS_peak_8008	4505
chr18	36894723	36902045	Tmco6	0	+	MACS_peak_8008	-20664
chr18	36942933	36952214	Hars2	0	+	MACS_peak_8008	27546
chr18	36923325	36925868	Dnd1	0	-	MACS_peak_8008	-10481
chr18	37840111	38001524	Pcdhgb1	0	+	MACS_peak_8009|MACS_peak_8010	-1190|-28467
chr18	37849512	38001526	Pcdhgb2	0	+	MACS_peak_8009|MACS_peak_8010	8211|-19066
chr18	37821598	38001524	Pcdhga1	0	+	MACS_peak_8009	-19703
chr18	37828758	38001524	Pcdhga2	0	+	MACS_peak_8009	-12543
chr18	37833988	38001526	Pcdhga3	0	+	MACS_peak_8009	-7313
chr18	37845053	38001524	Pcdhga4	0	+	MACS_peak_8009|MACS_peak_8010	3752|-23525
chr18	37854154	38001524	Pcdhga5	0	+	MACS_peak_8009|MACS_peak_8010	12853|-14424
chr18	37866882	38001524	Pcdhga6	0	+	MACS_peak_8009|MACS_peak_8010	25581|-1696
chr18	37880207	38001524	Pcdhgb4	0	+	MACS_peak_8010	11629
chr18	37890807	38001524	Pcdhgb5	0	+	MACS_peak_8010	22229
chr18	37874487	38001526	Pcdhga7	0	+	MACS_peak_8010	5909
chr18	37885359	38001526	Pcdhga8	0	+	MACS_peak_8010	16781
chr18	37896589	38001517	Pcdhga9	0	+	MACS_peak_8010	28011
chr18	38004478	38095065	Diap1	0	-	MACS_peak_8024|MACS_peak_8025	22655|24000
chr18	38096624	38114642	Hdac3	0	-	MACS_peak_8024|MACS_peak_8025	3078|4423
chr18	38115212	38118832	Rell2	0	+	MACS_peak_8024|MACS_peak_8025	-2508|-3853
chr18	38117087	38129385	Fchsd1	0	-	MACS_peak_8024|MACS_peak_8025	-11665|-10320
chr18	38356347	38369416	Pcdh1	0	-	MACS_peak_8026|MACS_peak_8027	-11626|-6589
chr18	38578628	38624060	Ndfip1	0	+	MACS_peak_8030	-28780
chr18	39570198	39646899	Nr3c1	0	-	MACS_peak_8033	2408
chr18	40364518	40379053	Yipf5	0	-	MACS_peak_8034	-13767
chr18	42338328	42356363	Plac8l1	0	-	MACS_peak_8035|MACS_peak_8036|MA	5839|13328|14502|15851|18136|20208|20799|23213|24861|29074
chr18	42362002	42421725	Lars	0	-	MACS_peak_8050|MACS_peak_8051|MA	-27423|-25450|-20057|-17210|-4942|-25
chr18	42435006	42501192	Rbm27	0	+	MACS_peak_8055|MACS_peak_8056	18223|13306
chr18	42434040	42434851	Gm4013	0	-	MACS_peak_8055|MACS_peak_8056	-18068|-13151
chr18	43483880	43532926	Dpysl3	0	-	MACS_peak_8060	-18498
chr18	44988318	45049373	Ythdc2	0	+	MACS_peak_8063	-17092
chr18	4634926	4682867	9430020K	0	+	MACS_peak_7805	-14318
chr18	46757357	46769879	Eif1a	0	+	MACS_peak_8067	-11169
chr18	46745581	46757189	Tmed7	0	-	MACS_peak_8067	11337
chr18	47118529	47247648	Commd10	0	+	MACS_peak_8069	-1585
chr18	47404907	47528522	Sema6a	0	-	MACS_peak_8073	-441
chr18	5046586	5119291	Svil	0	+	MACS_peak_7813|MACS_peak_7814|MA	-12761|-15695|-16893
chr18	52625346	52650392	Srfbp1	0	+	MACS_peak_8077	-9947
chr18	53841376	53904201	Cep120	0	-	MACS_peak_8092|MACS_peak_8093	-29465|-21354
chr18	56591785	56663446	Gramd3	0	+	MACS_peak_8101	-96
chr18	60685874	60718416	Dctn4	0	+	MACS_peak_8111	-76
chr18	60633844	60661637	2010002N	0	-	MACS_peak_8111	24313
chr18	60845629	60873043	Ndst1	0	-	MACS_peak_8119	-211
chr18	6201002	6241522	Kif5b	0	-	MACS_peak_7845	-28282
chr18	62112728	62175373	Sh3tc2	0	+	MACS_peak_8122	-24937
chr18	62663712	62708397	Fbxo38	0	-	MACS_peak_8130	-22569
chr18	62708564	62821042	Spink10	0	+	MACS_peak_8130	22736
chr18	64659306	64676211	Nars	0	-	MACS_peak_8145|MACS_peak_8145|MA	-16360|-16360|-15067|-15067|-14764|-14764|-11610|-11610|-11301|-11301|-4719|-4719|-1190|-1190
chr18	64616203	64648720	Fech	0	-	MACS_peak_8145|MACS_peak_8146|MA	11131|12424|12727|15881|16190|22772|26301
chr18	65047409	65377480	Nedd4l	0	+	MACS_peak_8152	-230
chr18	66143340	66162289	Lman1	0	-	MACS_peak_8165|MACS_peak_8165|MA	-18405|-18405|-11269|-11269|-11045|-11045|-9495|-9495|-5005|-5005
chr18	66115375	66129832	Cplx4	0	-	MACS_peak_8165|MACS_peak_8167|MA	14052|21188|21412|22962|27452
chr18	67550384	67562403	Tubb6	0	+	MACS_peak_8183|MACS_peak_8184|MA	-14418|-18770|-23404
chr18	67564417	67608790	Afg3l2	0	-	MACS_peak_8186|MACS_peak_8188	-26115|-6782
chr18	67624502	67640235	Slmo1	0	+	MACS_peak_8188	22494
chr18	6765202	6790229	Rab18	0	+	MACS_peak_7856	-24109
chr18	68092910	68415203	D18Ertd65	0	+	MACS_peak_8189	-848
chr18	68460008	68484506	Rnmt	0	+	MACS_peak_8191|MACS_peak_8191	-18849|-18849
chr18	68497256	68499365	Mc5r	0	+	MACS_peak_8191	18399
chr18	68419838	68459987	4933403F	0	-	MACS_peak_8191	18870

chr18	68566560	68588913	Mc2r	0	-	MACS_peak_8192	-16082
chr18	69505374	69847621	Tcf4	0	+	MACS_peak_8194|MACS_peak_8194|MA	-128|-1357|-3386|-4615
chr18	70668334	70689792	Poli	0	-	MACS_peak_8199|MACS_peak_8199|MA	-20460|-20643|-12694|-12877
chr18	74427941	74442338	Mbd1	0	+	MACS_peak_8205	-8285
chr18	74442753	74519638	Ccdc11	0	+	MACS_peak_8205|MACS_peak_8205	6527|6527
chr18	75178425	75446620	Dym	0	+	MACS_peak_8208	-24403
chr18	75527018	75555588	Smad7	0	+	MACS_peak_8212|MACS_peak_8213	-217|-8540
chr18	78240352	78254007	Siglec15	0	-	MACS_peak_8232|MACS_peak_8233|MA	-29880|-28389|-23825|-23277|-22676
chr18	78296830	78320165	Slc14a1	0	-	MACS_peak_8242|MACS_peak_8242|MA	-6993|-25686|-6993
chr18	80323873	80348221	Adnp2	0	-	MACS_peak_8243	-74
chr18	80426496	80443841	Hsbp1l1	0	-	MACS_peak_8244	9775
chr18	80451983	80489463	Pqlc1	0	+	MACS_peak_8244|MACS_peak_8244|MA	-1633|-572|-652|-572|-652|-572
chr18	84181018	84255954	Tshz1	0	-	MACS_peak_8255|MACS_peak_8256	-497|1695
chr18	84257549	84266906	Zadh2	0	+	MACS_peak_8255|MACS_peak_8256	2092|-100
chr18	90679545	90712659	Tmx3	0	+	MACS_peak_8270	-13425
chr18	9618416	9619467	Cetn1	0	-	MACS_peak_7865	11826
chr18	9958177	9995482	Thoc1	0	+	MACS_peak_7866	-12100
chr19	10116037	10134161	Fads3	0	+	MACS_peak_8478|MACS_peak_8479|MA	-10709|-14491|-14707|-17448
chr19	10282761	10315238	Gm98	0	-	MACS_peak_8482|MACS_peak_8483|MA	4866|7301|24311|29019
chr19	10463579	10527671	Syt7	0	+	MACS_peak_8487|MACS_peak_8487|MA	-24287|-24287|-24287
chr19	10530578	10531937	Lrrc10b	0	-	MACS_peak_8489	-21831
chr19	10680114	10704311	Ddb1	0	+	MACS_peak_8497|MACS_peak_8500|MA	27854|8652|-21516
chr19	10624955	10630728	Tmem216	0	-	MACS_peak_8497	21532
chr19	10645374	10651587	Tmem138	0	-	MACS_peak_8497|MACS_peak_8500	673|19875
chr19	10666686	10678748	Dak	0	-	MACS_peak_8497|MACS_peak_8500|MA	-26488|-7286|22882
chr19	10652212	10664320	Cybasc3	0	+	MACS_peak_8497|MACS_peak_8500	-48|-19250
chr19	10708722	10739700	Vwce	0	+	MACS_peak_8508	7092
chr19	11822048	11837937	Gif	0	+	MACS_peak_8510	-18915
chr19	11844904	11849436	Mrpl16	0	+	MACS_peak_8510	3941
chr19	11970512	11971442	Olfr1420	0	+	MACS_peak_8512	-27391
chr19	11986888	12019585	Patl1	0	+	MACS_peak_8512	-11015
chr19	12040333	12068604	Osbp	0	+	MACS_peak_8514	-27131
chr19	12840496	12870612	Zfp91	0	-	MACS_peak_8521	-243
chr19	12873098	12908298	Lpxn	0	+	MACS_peak_8521	2729
chr19	12838018	12870613	Zfp91-Cntf	0	-	MACS_peak_8521	-244
chr19	15979621	15999515	Psat1	0	-	MACS_peak_8525	-19618
chr19	18654853	18706303	Ostf1	0	-	MACS_peak_8547|MACS_peak_8548	-84|10405
chr19	18706505	18726674	BC016495	0	+	MACS_peak_8547|MACS_peak_8548	286|-10203
chr19	18745269	18779282	2410127L1	0	+	MACS_peak_8548	28561
chr19	20676471	20717952	Aldh1a1	0	+	MACS_peak_8565	-22792
chr19	26679649	26852811	Smarca2	0	+	MACS_peak_8590	-14611
chr19	27291509	27328721	Vldlr	0	+	MACS_peak_8599|MACS_peak_8599|MA	-137|-137|-19253|-19253|-22708|-22708|-29614|-29614
chr19	27463191	27504310	D19Bwg13	0	-	MACS_peak_8619	-3911
chr19	27504526	27507121	C030016D	0	+	MACS_peak_8619	4127
chr19	29175864	29218333	Rcl1	0	+	MACS_peak_8622	-19929
chr19	29209768	29209865	Mir101b	0	+	MACS_peak_8622	13975
chr19	29684372	29722910	Ermp1	0	-	MACS_peak_8631|MACS_peak_8632	-21475|-19708
chr19	30105002	30168214	Uhrf2	0	+	MACS_peak_8638|MACS_peak_8639	-27448|-28749
chr19	32059140	32177630	Asah2	0	-	MACS_peak_8657	20524
chr19	3259075	3283010	Ighmbp2	0	-	MACS_peak_8289|MACS_peak_8290|MA	-21761|-17661|-10050|-6176|-3433|-1527
chr19	3283046	3292837	Mrpl21	0	+	MACS_peak_8289|MACS_peak_8290|MA	21797|17697|10086|6212|3469|1563
chr19	34315580	34329826	Acta2	0	-	MACS_peak_8661|MACS_peak_8662|MA	-28689|-25205|-21199|-16508
chr19	36992039	36994089	Fgfbp3	0	-	MACS_peak_8673	-27498
chr19	37343230	37405103	Ide	0	-	MACS_peak_8675	-18483
chr19	3851772	3867549	Chka	0	+	MACS_peak_8296|MACS_peak_8296|MA	-352|-352|-12940|-12940
chr19	3896049	3907133	Tcirg1	0	-	MACS_peak_8298|MACS_peak_8298|MA	-22610|-22460|-20707|-21208|-21058|-19305|-20675|-20525|-18772|-19766|-19616|-17863|-18026|-17876|-16123|-14978|-14828|-13075|-
chr19	3908869	3912717	Ndufs8	0	-	MACS_peak_8298|MACS_peak_8299|MA	-28194|-26792|-26259|-25350|-23610|-20562|-20016
chr19	38862617	38893727	Noc3l	0	-	MACS_peak_8691	-20305
chr19	40296728	40346106	Pdlim1	0	-	MACS_peak_8699|MACS_peak_8701|MA	-169|21120|24957
chr19	40366529	40476800	Sorbs1	0	-	MACS_peak_8707	-28748
chr19	40624747	40662953	Aldh18a1	0	-	MACS_peak_8710|MACS_peak_8710|MA	-14177|-14177|-2911|-2911
chr19	40670935	40686705	Tctn3	0	-	MACS_peak_8711	-26663
chr19	4125959	4132307	Tmem134	0	+	MACS_peak_8318|MACS_peak_8318|MA	24766|24766|22916|22916|17211|17211
chr19	4100116	4113964	Pitpnm1	0	+	MACS_peak_8318|MACS_peak_8318|MA	-1077|-572|-2927|-2422|-8632|-8127
chr19	4083518	4087337	Cabp2	0	+	MACS_peak_8318|MACS_peak_8318|MA	-17675|-18706|-17675|-19525|-20556|-19525|-25230|-26261|-25230
chr19	4114445	4125827	Aip	0	-	MACS_peak_8318|MACS_peak_8319|MA	-24634|-22784|-17079
chr19	4097350	4099017	Cdk2ap2	0	+	MACS_peak_8318|MACS_peak_8319|MA	-3843|-5693|-11398
chr19	42197764	42203483	Avpi1	0	-	MACS_peak_8717	-24074
chr19	42164924	42196708	Pi4k2a	0	+	MACS_peak_8717	-14485
chr19	42149428	42160860	Morn4	0	-	MACS_peak_8717	18549
chr19	4270179	4283137	Ankrd13d	0	-	MACS_peak_8326|MACS_peak_8330	-17474|-14042
chr19	4261667	4269172	Ssh3	0	-	MACS_peak_8326|MACS_peak_8330	-3509|-77
chr19	42666769	42687296	Loxl4	0	-	MACS_peak_8722|MACS_peak_8722	-8545|-8545
chr19	42829685	42854466	Hps1	0	-	MACS_peak_8724|MACS_peak_8725	-22105|-12783
chr19	42800347	42827265	Pyroxd2	0	-	MACS_peak_8724|MACS_peak_8725	5096|14418
chr19	43574242	43599095	Got1	0	-	MACS_peak_8728|MACS_peak_8730	-20003|-16672
chr19	43764178	43808343	Entpd7	0	+	MACS_peak_8732	-1392
chr19	43738291	43749371	Slc25a28	0	-	MACS_peak_8732	16199
chr19	44079511	44103737	Cyp2c44	0	-	MACS_peak_8734|MACS_peak_8734|MA	6808|6808|9965|9965|18437|18437|19904|19904|26900|26900
chr19	44109433	44144265	Erlin1	0	-	MACS_peak_8737|MACS_peak_8737|MA	-22091|-22101|-22002|-20624|-20634|-20535|-13628|-13638|-13539|-2134|-2144|-2045|-444|-454|-355
chr19	45122065	45130873	Sfxn3	0	+	MACS_peak_8746|MACS_peak_8746|MA	-2252|-2252|-2252|-2707|-2707|-2707
chr19	45150628	45153779	Kazald1	0	+	MACS_peak_8746|MACS_peak_8747	26311|25856
chr19	45822223	45824053	Npm3	0	-	MACS_peak_8750	24146
chr19	45824748	45857781	Mgea5	0	-	MACS_peak_8750	-9582
chr19	46379226	46386578	Nfkb2	0	+	MACS_peak_8758|MACS_peak_8758|MA	18804|19997|19762|-6632|-5439|-5674
chr19	46404301	46413150	Cuedc2	0	-	MACS_peak_8759|MACS_peak_8759|MA	-27292|-27292|-27292|-27292|-27292|-27292|-27292
chr19	46386576	46401646	Psd	0	-	MACS_peak_8759	-15788
chr19	46402673	46404936	Fbxl15	0	+	MACS_peak_8759	16815
chr19	46836098	46953070	Cnnm2	0	+	MACS_peak_8760|MACS_peak_8760	-1732|-1732
chr19	4886883	4906627	Bbs1	0	-	MACS_peak_8331	17085
chr19	4907228	4928287	Dpp3	0	-	MACS_peak_8331	-4575
chr19	4931854	4943092	Peli3	0	-	MACS_peak_8331	-19380
chr19	5024005	5031972	Slc29a2	0	+	MACS_peak_8333	-28340
chr19	5068077	5070639	Cd248	0	+	MACS_peak_8333|MACS_peak_8334	15732|12158
chr19	5041403	5049917	Brms1	0	+	MACS_peak_8333|MACS_peak_8334	-10942|-14516
chr19	5050807	5057071	Rin1	0	+	MACS_peak_8333|MACS_peak_8334	-1538|-5112
chr19	5038825	5041134	B3gnt1	0	+	MACS_peak_8333|MACS_peak_8334	-13520|-17094
chr19	5079336	5085477	Tmem151a	0	-	MACS_peak_8334	-29558
chr19	53065672	53113032	Xpnpep1	0	-	MACS_peak_8770	-6485
chr19	5298330	5308738	Gal3st3	0	+	MACS_peak_8338	-26840
chr19	5344704	5349574	Cst6	0	-	MACS_peak_8338	-24404
chr19	5273920	5295455	Sf3b2	0	-	MACS_peak_8338	29715
chr19	5335740	5344153	Catsper1	0	+	MACS_peak_8338	10570
chr19	53384995	53450300	Mxi1	0	+	MACS_peak_8778|MACS_peak_8778|MA	-20917|-977|-2010
chr19	53453703	53465063	Smndc1	0	-	MACS_peak_8780	-10560
chr19	53977840	54004351	Pdcd4	0	+	MACS_peak_8789|MACS_peak_8789	-22900|-22900
chr19	54018795	54107768	Shoc2	0	+	MACS_peak_8789|MACS_peak_8789	18055|18625
chr19	54004155	54019117	Ncrna0008	0	-	MACS_peak_8789	-18377
chr19	54119671	54123472	Adra2a	0	+	MACS_peak_8794	17368
chr19	5474734	5481854	Efemp2	0	+	MACS_peak_8339|MACS_peak_8339|MA	26810|26765|24500|24455
chr19	5465239	5468498	Ctsw	0	-	MACS_peak_8339|MACS_peak_8340	-20574|-18264
chr19	5447697	5455938	Fosl1	0	+	MACS_peak_8339|MACS_peak_8340	-227|-2537
chr19	5460693	5464994	Fibp	0	+	MACS_peak_8339|MACS_peak_8340	12769|10459
chr19	5422823	5424916	Drap1	0	-	MACS_peak_8339|MACS_peak_8340	23008|25318
chr19	5425143	5427314	AI837181	0	+	MACS_peak_8339|MACS_peak_8339|MA	-22781|-22781|-25091|-25091
chr19	5453162	5457549	Ccdc85b	0	-	MACS_peak_8339|MACS_peak_8339|MA	-9625|-9625|-7315|-7315
chr19	55144223	55173937	Gpam	0	-	MACS_peak_8795	-21552
chr19	55816299	56008144	Tcf7l2	0	+	MACS_peak_8796|MACS_peak_8796|MA	-620|-620|-620|-620|-620|-620|-620
chr19	5651184	5663707	Sipa1	0	-	MACS_peak_8342|MACS_peak_8342|MA	-25972|-25972|-25972|-25972|-25972|21938|21938|21938|21938|21938|22778|22778|22778|22778|22778
chr19	5637489	5648130	Rela	0	+	MACS_peak_8342	-246
chr19	5603417	5610029	Kat5	0	-	MACS_peak_8342	27706
chr19	5704475	5707101	Kcnk7	0	+	MACS_peak_8343|MACS_peak_8344|MA	18830|17990|-12647|-15616|-21618

chr19	5689130	5702864	Map3k11	0	+	MACS_peak_8343|MACS_peak_8344|MA(	3485|2645|-27992
chr19	5664634	5688908	Pcnxl3	0	-	MACS_peak_8343|MACS_peak_8344|MA(	-3263|-2423|28214
chr19	5707373	5726317	Ehbp1l1	0	-	MACS_peak_8346|MACS_peak_8346|MA(	-9195|-9195|-9195|-9195|-6226|-6226|-6226|-6226|-224|-224|-224|-224|20084|20084|20084|20084|25501|25501|25501|25501
chr19	5740903	5758532	Ltbp3	0	+	MACS_peak_8346|MACS_peak_8347|MA(	23781|20812|14810|-5498|-10915
chr19	5730305	5731721	Sssca1	0	-	MACS_peak_8346|MACS_peak_8347|MA(	-14599|-11630|-5628|14680|20097
chr19	5728087	5729666	Mtvr2	0	-	MACS_peak_8346|MACS_peak_8346|MA(	-12544|-12544|-9575|-9575|-3573|-3573|16735|16735|22152|22152
chr19	57435498	57464084	Fam160b1	0	+	MACS_peak_8807|MACS_peak_8808|MA(	-10225|-12334|-17685|-18068|-20100
chr19	5758426	5771401	Scyl1	0	-	MACS_peak_8352|MACS_peak_8358	-25000|-19583
chr19	5795689	5802671	Malat1	0	-	MACS_peak_8371|MACS_peak_8377|MA(	24675|27640|29300
chr19	5842301	5845478	Neat1	0	-	MACS_peak_8371|MACS_peak_8377|MA(	-18132|-15167|-13507|-11727|-10626|-8259
chr19	5897134	5912866	Dpf2	0	-	MACS_peak_8394|MACS_peak_8395|MA(	-14846|-11643|-10169|-6289
chr19	5850973	5875208	Frmd8	0	-	MACS_peak_8394|MACS_peak_8395|MA(	22812|26015|27489
chr19	5917560	5918993	Cdc42ep2	0	-	MACS_peak_8394|MACS_peak_8395|MA(	-20973|-17770|-16296|-12416
chr19	5878465	5885768	Slc25a45	0	+	MACS_peak_8394|MACS_peak_8395|MA(	-19555|-22758|-24232|-28112
chr19	5891137	5894107	Tigd3	0	-	MACS_peak_8394|MACS_peak_8395|MA(	3913|7116|8590|12470
chr19	59978788	60019267	Rab11fip2	0	-	MACS_peak_8832|MACS_peak_8832	-6124|-6414
chr19	6067841	6077187	1110014N:	0	-	MACS_peak_8398|MACS_peak_8401|MA(	-28658|-5558|3820|4716|5249
chr19	6057887	6059524	Fau	0	+	MACS_peak_8398|MACS_peak_8398|MA(	9358|9437|9358|-13742|-13663|-13742|-23120|-23041|-23120|-24016|-23937|-24016|-24549|-24470|-24549
chr19	6047144	6053717	Syvn1	0	+	MACS_peak_8398|MACS_peak_8398|MA(	-1385|-1954|-24485|-25054
chr19	6061206	6062468	Znhit2-ps	0	+	MACS_peak_8398|MACS_peak_8401|MA(	12677|-10423|-19801|-20697|-21230
chr19	6053629	6057751	Mrpl49	0	-	MACS_peak_8398|MACS_peak_8401|MA(	-9222|13878|23256|24152|24685
chr19	6062820	6067850	Tm7sf2	0	-	MACS_peak_8398|MACS_peak_8401|MA(	-19321|3779|13157|14053|14586
chr19	6080037	6080785	BC048609	0	+	MACS_peak_8401|MACS_peak_8402|MA(	8408|-970|-1866|-2399
chr19	6080762	6084891	Zfpl1	0	-	MACS_peak_8401|MACS_peak_8402|MA(	-13262|-3884|-2988|-2455
chr19	6085096	6091773	Cdca5	0	+	MACS_peak_8401|MACS_peak_8402|MA(	13467|4089|3193|2660
chr19	6105797	6115555	Naaladl1	0	+	MACS_peak_8402|MACS_peak_8403|MA(	24790|23894|23361|-18087
chr19	60837018	60866596	Eif3a	0	-	MACS_peak_8836|MACS_peak_8845	-23077|-8433
chr19	60831889	60835817	Nanos1	0	+	MACS_peak_8836|MACS_peak_8845	-11630|-26274
chr19	60850232	60850288	Snora19	0	-	MACS_peak_8836|MACS_peak_8845	-6769|7875
chr19	60887472	60912130	Fam45a	0	+	MACS_peak_8845|MACS_peak_8845|MA(	29309|29309|-10789|-10789
chr19	6134388	6141137	Arl2	0	-	MACS_peak_8408	-17253
chr19	6119403	6128215	Snx15	0	-	MACS_peak_8408	-4331
chr19	6116003	6118586	Sac3d1	0	-	MACS_peak_8408	5298
chr19	6226400	6227767	Gpha2	0	+	MACS_peak_8409|MACS_peak_8410|MA(	-2892|-6144|-24933
chr19	6241667	6262304	Atg2a	0	+	MACS_peak_8409|MACS_peak_8410|MA(	12375|9123|-9666
chr19	6227766	6235840	Ppp2r5b	0	-	MACS_peak_8409|MACS_peak_8410|MA(	-6548|-3296|15493
chr19	6276895	6300096	Ehd1	0	+	MACS_peak_8413	25562
chr19	6264843	6264932	Mir192	0	+	MACS_peak_8413	13510
chr19	6264642	6264728	Mir194-2	0	+	MACS_peak_8413	13309
chr19	6306456	6325652	Cdc42bpg	0	+	MACS_peak_8415|MACS_peak_8416|MA(	-3829|-8284|-9922|-11190|-18365
chr19	6334978	6340894	Men1	0	+	MACS_peak_8415|MACS_peak_8415|MA(	24693|24727|24753|24753|20238|20272|20298|20298|18600|18634|18660|18660|17332|17366|17392|17392|10157|10191|10217|1021
chr19	6341249	6353527	Map4k2	0	+	MACS_peak_8416|MACS_peak_8417|MA(	26509|24871|23603|16428
chr19	7095185	7114516	Stip1	0	-	MACS_peak_8427	25489
chr19	7131257	7272552	Macrod1	0	+	MACS_peak_8427	-8748
chr19	7938879	8040517	AB056442	0	-	MACS_peak_8434|MACS_peak_8435	11026|11843
chr19	8781371	8797859	Slc3a2	0	-	MACS_peak_8444|MACS_peak_8444|MA(	-13618|-4146|12866|22338|18829|28301|25129
chr19	8810328	8815114	Wdr74	0	+	MACS_peak_8444|MACS_peak_8448|MA(	26087|-397|-6360|-12660
chr19	8797976	8800816	Snhg1	0	+	MACS_peak_8444|MACS_peak_8448|MA(	13735|-12749|-18712|-25012
chr19	8800355	8800481	Snord22	0	+	MACS_peak_8444|MACS_peak_8448|MA(	16114|-10370|-16333|-22633
chr19	8815913	8830131	Stx5a	0	+	MACS_peak_8448|MACS_peak_8448|MA(	5188|5567|-775|-396|-7075|-6696
chr19	8815207	8815714	1700092M	0	+	MACS_peak_8448|MACS_peak_8449|MA(	4482|-1481|-7781
chr19	8831592	8845400	Nxf1	0	+	MACS_peak_8448|MACS_peak_8449|MA(	20867|14904|8604
chr19	8845485	8846965	Tmem223	0	+	MACS_peak_8449|MACS_peak_8451	28797|22497
chr19	8847011	8848957	Tmem179b	0	-	MACS_peak_8451|MACS_peak_8451|MA(	-25969|-25969|-25969
chr19	8848974	8851452	Gm2518	0	+	MACS_peak_8451	25986
chr19	8925274	8928399	Lrrn4cl	0	+	MACS_peak_8454	-252
chr19	8911956	8923172	Bscl2	0	+	MACS_peak_8454|MACS_peak_8454	-13570|-11709
chr19	8954505	8955423	AI462493	0	-	MACS_peak_8454|MACS_peak_8459	-29897|28589
chr19	8911418	8913736	Gng3	0	-	MACS_peak_8454	11790
chr19	8946048	8950146	Ubxn1	0	+	MACS_peak_8454	20522
chr19	8972600	8991155	Ganab	0	+	MACS_peak_8459|MACS_peak_8462	-11412|-16872
chr19	9001871	9003846	Rom1	0	-	MACS_peak_8459|MACS_peak_8462|MA(	-19834|-14374|18203|22509|29886
chr19	8994882	9001726	B3gat3	0	+	MACS_peak_8459|MACS_peak_8462|MA(	10870|5410|-27167
chr19	8963392	8965230	1810009A:	0	+	MACS_peak_8459|MACS_peak_8462	-20620|-26080
chr19	9004183	9016072	Eml3	0	+	MACS_peak_8459|MACS_peak_8462|MA(	20171|14711|-17866|-22172|-29549
chr19	8967476	8972379	Ints5	0	+	MACS_peak_8459|MACS_peak_8462	-16536|-21996
chr19	8962878	8963260	5730408K(	0	-	MACS_peak_8459|MACS_peak_8462	20752|26212
chr19	9016409	9026790	Mta2	0	+	MACS_peak_8462|MACS_peak_8464|MA(	26937|-5640|-9946|-17323|-20661|-23682
chr19	9041530	9052670	Eef1g	0	+	MACS_peak_8464|MACS_peak_8465|MA(	19481|15175|7798|4460|1439
chr19	9028339	9040700	Tut1	0	+	MACS_peak_8464|MACS_peak_8465|MA(	6290|1984|-5393|-8731|-11752
chr19	9063773	9151409	Ahnak	0	+	MACS_peak_8467|MACS_peak_8467|MA(	26703|26703|23682|23682
chr1_rando	249689	270356	0610010K(	0	-	MACS_peak_8856|MACS_peak_8857|MA(	-16179|-14478|-26
chr1_rando	270407	297849	Aida	0	+	MACS_peak_8856|MACS_peak_8857|MA(	16230|14529|77
chr1_rando	299583	342913	Mia3	0	-	MACS_peak_8868	-25253
chr2	101478410	101489689	Rag1	0	-	MACS_peak_9427	-20588
chr2	101464904	101472685	Rag2	0	+	MACS_peak_9427	-4197
chr2	101400937	101469143	B230118H(	0	-	MACS_peak_9427	-42
chr2	103294060	103325310	Cat	0	-	MACS_peak_9434|MACS_peak_9437|MA(	-24857|-14785|-8319|-101
chr2	103561415	103601407	Nat10	0	-	MACS_peak_9444	-10997
chr2	105226104	105239476	Rcn1	0	-	MACS_peak_9450	-10209
chr2	109857973	109885480	Ccdc34	0	+	MACS_peak_9457	3921
chr2	113167892	113556924	Fmn1	0	+	MACS_peak_9462|MACS_peak_9462	-24886|-24886
chr2	113588831	113598805	Grem1	0	-	MACS_peak_9464|MACS_peak_9465	-9374|-67
chr2	115686999	115890794	Meis2	0	-	MACS_peak_9468|MACS_peak_9468|MA(	580|580|580|1017|1017|580
chr2	115900283	115901832	2810405F1	0	-	MACS_peak_9468	-10458
chr2	118301578	118305432	Srp14	0	-	MACS_peak_9472	-28228
chr2	118579880	118588397	A430105I1	0	-	MACS_peak_9473|MACS_peak_9474	-14761|23627
chr2	118598504	118603900	Phgr1	0	+	MACS_peak_9473|MACS_peak_9474|MA(	24868|-13520|-20205|-20677
chr2	118533252	118554174	Plcb2	0	-	MACS_peak_9473	19462
chr2	118571497	118574543	5430417L2	0	+	MACS_peak_9473	-2139
chr2	118639738	118661947	D2Ertd750	0	+	MACS_peak_9474|MACS_peak_9475|MA(	27714|21029|20557
chr2	118605454	118620911	Disp2	0	+	MACS_peak_9474|MACS_peak_9475|MA(	-6570|-13255|-13727
chr2	118727350	118750260	Bahd1	0	+	MACS_peak_9477|MACS_peak_9478|MA(	28385|23701|-19182
chr2	118687735	118707093	Ivd	0	+	MACS_peak_9477|MACS_peak_9478	-11230|-15914
chr2	118752232	118754317	Chst14	0	+	MACS_peak_9485	5700
chr2	118962734	118982770	Fam82a2	0	-	MACS_peak_9488|MACS_peak_9489	-10660|-3590
chr2	119000244	119003311	Gm14137	0	+	MACS_peak_9488|MACS_peak_9489	28134|21064
chr2	118993523	118998125	Gchfr	0	+	MACS_peak_9488|MACS_peak_9489	21413|14343
chr2	119094938	119096962	Rhov	0	-	MACS_peak_9490	21514
chr2	119114477	119124189	Vps18	0	+	MACS_peak_9490	-3999
chr2	119151519	119161402	Dll4	0	+	MACS_peak_9491|MACS_peak_9492	-25653|-27972
chr2	119176977	119180060	Chac1	0	+	MACS_peak_9491|MACS_peak_9492	-195|-2514
chr2	119146934	119151933	Gm14207	0	-	MACS_peak_9491|MACS_peak_9492	25239|27558
chr2	119589695	119613273	Rpap1	0	-	MACS_peak_9495|MACS_peak_9495|MA(	-75|-75|6291|6291
chr2	119577061	119584261	Ltk	0	-	MACS_peak_9495|MACS_peak_9495|MA(	28937|27031|28937|27031
chr2	119625249	119643839	Tyro3	0	+	MACS_peak_9495|MACS_peak_9496	12051|5685
chr2	119798434	119853138	Mapkbp1	0	+	MACS_peak_9497	-360
chr2	119853218	119858340	Jmjd7	0	+	MACS_peak_9498	5319
chr2	119859168	119868768	Pla2g4b	0	+	MACS_peak_9498	11269
chr2	120125692	120139901	Pla2g4f	0	-	MACS_peak_9501|MACS_peak_9503	8937|19288
chr2	120142201	120178869	Vps39	0	-	MACS_peak_9503|MACS_peak_9503|MA(	-19680|-19680|-19680
chr2	120429975	120435244	Lrrc57	0	-	MACS_peak_9505|MACS_peak_9505|MA(	6416|6416|6566|6609|6416
chr2	120435171	120446554	Haus2	0	+	MACS_peak_9505	-6489
chr2	120541889	120557253	Cdan1	0	-	MACS_peak_9506	-3125
chr2	120834143	120842648	Ccndbp1	0	+	MACS_peak_9507	-126
chr2	120843952	120862491	Epb4.2	0	-	MACS_peak_9507	-28222
chr2	121022464	121097122	Trp53bp1	0	-	MACS_peak_9510	-23156
chr2	121287401	121299759	Mfap1b	0	-	MACS_peak_9516|MACS_peak_9518	18076|20948
chr2	121318416	121332392	Mfap1a	0	-	MACS_peak_9516|MACS_peak_9518|MA(	-14557|-11685|25362
chr2	121332458	121370595	Wdr76	0	+	MACS_peak_9516|MACS_peak_9518|MA(	14623|11751|-25296
chr2	121879262	121944122	Spg11	0	-	MACS_peak_9521	-25310
chr2	122060574	122091073	Sord	0	+	MACS_peak_9524	-22071

chr2	122463622	122466812	AA467197	0	+	MACS_peak_9527	6809
chr2	122420208	122437013	Gatm	0	-	MACS_peak_9527	19800
chr2	122466538	122466617	Mir147	0	+	MACS_peak_9527	9725
chr2	12268890	12341087	Fam188a	0	-	MACS_peak_8903	-5138
chr2	124436031	124493505	Sema6d	0	+	MACS_peak_9538|MACS_peak_9538|MA(	-570|-570|-570|-570|-570
chr2	126419403	126444401	Hdc	0	-	MACS_peak_9541	20564
chr2	126896392	126922820	Blvra	0	+	MACS_peak_9547	-26276
chr2	127034139	127066187	Snrnp200	0	+	MACS_peak_9549|MACS_peak_9553|MA(	-3974|-18642|-19547|-21558|-24596|-24864|-28152|-28418
chr2	127012090	127034016	1810024B(	0	-	MACS_peak_9549|MACS_peak_9553|MA(	4097|18765|19670|21681|24719|24987|28275|28541
chr2	127066673	127073552	Ciao1	0	-	MACS_peak_9553|MACS_peak_9554|MA(	-20771|-19866|-17855|-14817|-14549|-11261|-10995|-9237|-7929
chr2	127073710	127086500	Tmem127	0	+	MACS_peak_9553|MACS_peak_9554|MA(	20929|20024|18013|14975|14707|11419|11153|9395|8087
chr2	127951773	127988283	Bcl2l11	0	+	MACS_peak_9567|MACS_peak_9567|MA(	-2636|-2636|-2636
chr2	130121674	130196217	Ebf4	0	+	MACS_peak_9587	11950
chr2	130100147	130105049	Nop56	0	+	MACS_peak_9587	-9577
chr2	130105044	130110187	Idh3b	0	-	MACS_peak_9587	-463
chr2	130103761	130103823	Snord57	0	+	MACS_peak_9587	-5963
chr2	130101250	130101307	Snord110	0	+	MACS_peak_9587	-8474
chr2	130243419	130250377	Fam113a	0	-	MACS_peak_9588|MACS_peak_9588|MA(	-1016|-663|15242|15595|16306|16659
chr2	130231031	130231809	1700020A2	0	+	MACS_peak_9588|MACS_peak_9588	-18330|-18367
chr2	130276013	130380036	Ptpra	0	+	MACS_peak_9588|MACS_peak_9588|MA(	26652|26652|10394|10394|9330|9330
chr2	130216511	130223365	Cpxm1	0	-	MACS_peak_9588	25996
chr2	130232257	130233530	4933425O2	0	+	MACS_peak_9588	-17104
chr2	130250055	130270005	Vps16	0	+	MACS_peak_9588|MACS_peak_9589|MA(	694|-15564|-16628
chr2	130389492	130391130	Mrps26	0	+	MACS_peak_9594	18761
chr2	130509843	130523255	Slc4a11	0	-	MACS_peak_9597|MACS_peak_9598|MA(	-13182|-12809|-12567|-11953|-11347|-10091|-9682|-9438|-6624|-6103|-5285|-817|-106|11129|12468|22383
chr2	130493576	130507350	Itpa	0	+	MACS_peak_9597|MACS_peak_9598|MA(	-16497|-16870|-17112|-17726|-18332|-19588|-19997|-20241|-23055|-23576|-24394|-28862|-29573
chr2	130479818	130490381	Ddrgk1	0	-	MACS_peak_9597|MACS_peak_9598|MA(	19692|20065|20307|20921|21527|22783|23192|23436|26250|26771|27589
chr2	130627703	130629837	Gm14057	0	-	MACS_peak_9616	-293
chr2	130972060	130985756	1700037H(	0	-	MACS_peak_9620|MACS_peak_9621	-12795|-6375
chr2	130995996	131000546	Spef1	0	-	MACS_peak_9620|MACS_peak_9621	-27585|-21165
chr2	130953147	130971719	Hspa12b	0	+	MACS_peak_9620|MACS_peak_9621	-19814|-26234
chr2	131003024	131005748	Cenpb	0	-	MACS_peak_9621	-26367
chr2	131036175	131039250	2310035K2	0	+	MACS_peak_9624	-23804
chr2	131059873	131073760	Mavs	0	+	MACS_peak_9624|MACS_peak_9629	-106|-18249
chr2	131088235	131124923	Pank2	0	+	MACS_peak_9624|MACS_peak_9629	28256|10113
chr2	131735663	131764167	Prnp	0	+	MACS_peak_9633	-9358
chr2	132672401	132692504	Crls1	0	+	MACS_peak_9641|MACS_peak_9641	-201|867
chr2	134420241	134469857	Tmx4	0	-	MACS_peak_9642	-23505
chr2	136907193	136942067	Jag1	0	-	MACS_peak_9649|MACS_peak_9650|MA(	-26489|-25397|-24794|-20070|-14666
chr2	138082319	138113158	Btbd3	0	+	MACS_peak_9654|MACS_peak_9654|MA(	-23207|-1298|-27383|-5474
chr2	139945616	139996294	Esf1	0	-	MACS_peak_9661|MACS_peak_9662|MA(	-21723|-15691|-13445|-11983|-10118|-6192|-2457|23066
chr2	139996381	140030988	2310003L2	0	+	MACS_peak_9661|MACS_peak_9662|MA(	21810|15778|13532|12070|10205|6279|2544|-22979
chr2	144194718	144199072	Pet117	0	+	MACS_peak_9681	-25284
chr2	144194767	144233411	Csrp2bp	0	+	MACS_peak_9681|MACS_peak_9681	-25235|-25232
chr2	144368000	144376595	Rbbp9	0	-	MACS_peak_9683|MACS_peak_9684|MA(	-7236|-2815|8468
chr2	144382012	144416479	Sec23b	0	+	MACS_peak_9683|MACS_peak_9684|MA(	12653|8232|-3051
chr2	144353480	144367515	Polr3f	0	+	MACS_peak_9683|MACS_peak_9684	-15879|-20300
chr2	144296292	144353134	6330439K1	0	-	MACS_peak_9683|MACS_peak_9684	16225|20646
chr2	145728976	145742160	Naa20	0	+	MACS_peak_9690|MACS_peak_9690	-15194|-15194
chr2	145743217	145760436	Crnkl1	0	-	MACS_peak_9690	-16266
chr2	145760519	145789962	4930529M	0	+	MACS_peak_9690	16349
chr2	148230206	148233924	Thbd	0	-	MACS_peak_9693|MACS_peak_9694	29529|29711
chr2	148262386	148269271	Cd93	0	-	MACS_peak_9693|MACS_peak_9694|MA(	-5818|-5636|-4199|-3866|-3099|-2921|-2397
chr2	148498376	148501762	Nxt1	0	+	MACS_peak_9700|MACS_peak_9700	-163|-189
chr2	148506855	148518683	Gzf1	0	+	MACS_peak_9700	8316
chr2	14916759	14976499	Nsun6	0	-	MACS_peak_8914|MACS_peak_8914|MA(	626|6186|626|6186|27153|27153
chr2	14976988	15000818	Arl5b	0	+	MACS_peak_8914|MACS_peak_8915	-137|-26664
chr2	149940142	149962414	Zfp120	0	-	MACS_peak_9707|MACS_peak_9707	-60|-60
chr2	151398367	151415735	Sdcbp2	0	+	MACS_peak_9711|MACS_peak_9712|MA(	-13780|-14773|-17063
chr2	151907264	151921537	Scrt2	0	+	MACS_peak_9714	-28642
chr2	151931465	151937089	Srxn1	0	+	MACS_peak_9714	-4441
chr2	152142069	152158161	Rbck1	0	-	MACS_peak_9716|MACS_peak_9716|MA(	5630|5416|7459|7245|10755|10541
chr2	152163160	152169796	Trib3	0	-	MACS_peak_9716|MACS_peak_9717|MA(	-6005|-4176|-880
chr2	152171404	152201058	Gm14164	0	+	MACS_peak_9716|MACS_peak_9717|MA(	7613|5784|2488
chr2	152219347	152223782	Sox12	0	-	MACS_peak_9720	15268
chr2	152237691	152240780	Zcchc3	0	-	MACS_peak_9720	-1730
chr2	152241322	152270064	6820408C1	0	+	MACS_peak_9720	2272
chr2	152987036	153036199	Tm9sf4	0	+	MACS_peak_9725	-26044
chr2	153048113	153051177	Tspyl3	0	-	MACS_peak_9729	-23071
chr2	154161121	154198455	Cdk5rap1	0	-	MACS_peak_9734|MACS_peak_9735	-16818|-14149
chr2	154377511	154379012	1700007I0	0	+	MACS_peak_9736	20451
chr2	154370140	154384589	Necab3	0	-	MACS_peak_9736	-27529
chr2	154374639	154375784	1700003F1	0	+	MACS_peak_9736	17579
chr2	154959216	155052590	Itch	0	+	MACS_peak_9750	-24374
chr2	155062268	155076013	Dynlrb1	0	+	MACS_peak_9754	10018
chr2	155216400	155266519	Ncoa6	0	-	MACS_peak_9757	-46
chr2	155460013	155518120	Trpc4ap	0	-	MACS_peak_9759|MACS_peak_9759	21018|21018
chr2	155527408	155555211	Edem2	0	-	MACS_peak_9759|MACS_peak_9760	-16073|23994
chr2	155601079	155644102	Mmp24	0	+	MACS_peak_9760	21874
chr2	155576952	155581213	Procr	0	+	MACS_peak_9760	-2253
chr2	155645572	155652661	Eif6	0	-	MACS_peak_9763	-403
chr2	155654918	155660590	Fam83c	0	-	MACS_peak_9763|MACS_peak_9764	-8332|23300
chr2	155643469	155644939	BC029722	0	-	MACS_peak_9763	7319
chr2	155826179	155830163	6430550D2	0	-	MACS_peak_9765|MACS_peak_9765	12404|8854
chr2	155833860	155844015	Ergic3	0	+	MACS_peak_9765	-8707
chr2	155969970	155971529	Romo1	0	+	MACS_peak_9767|MACS_peak_9767|MA(	16518|16436|16518|16627
chr2	155949372	155969922	Nfs1	0	-	MACS_peak_9767	-16470
chr2	155920617	155937701	Rbm12	0	-	MACS_peak_9767|MACS_peak_9767	15751|15751
chr2	155897577	155937701	Cpne1	0	-	MACS_peak_9767|MACS_peak_9767	15751|15751
chr2	157250028	157297574	Src	0	+	MACS_peak_9774|MACS_peak_9774	-2324|-2324
chr2	157385845	157388255	Nnat	0	+	MACS_peak_9775|MACS_peak_9775	2753|2753
chr2	157382097	157392097	Blcap	0	-	MACS_peak_9775	-9005
chr2	158235588	158324989	Ralgapb	0	+	MACS_peak_9786	-20636
chr2	158328347	158333934	Adig	0	+	MACS_peak_9787|MACS_peak_9788	26420|5291
chr2	158338531	158376362	Arhgap40	0	+	MACS_peak_9788	15475
chr2	158436493	158441483	Slc32a1	0	+	MACS_peak_9789|MACS_peak_9790	-20831|-28225
chr2	158450648	158464947	Actr5	0	+	MACS_peak_9789|MACS_peak_9790	-6676|-14070
chr2	158492870	158592069	Ppp1r16b	0	+	MACS_peak_9790|MACS_peak_9790	28152|27750
chr2	160557045	160601496	Plcg1	0	+	MACS_peak_9791|MACS_peak_9793|MA(	-20350|-21959|-22385|-22608|-23314|-25283
chr2	163449008	163470879	Serinc3	0	-	MACS_peak_9807|MACS_peak_9808|MA(	-18771|-17181|-15970|-12968|-8237
chr2	163428049	163444749	Ttpal	0	+	MACS_peak_9807|MACS_peak_9807|MA(	-24059|-24059|-25649|-25649|-26860|-26860|-29862|-29862
chr2	163470585	163471536	0610039K1	0	+	MACS_peak_9807|MACS_peak_9808|MA(	18477|16887|15676|12674|7943
chr2	163484121	163551894	Pkig	0	+	MACS_peak_9809|MACS_peak_9809|MA(	29212|29212|29284|29212|26210|26210|26282|26210|21479|21479|21551|21479
chr2	165102055	165113327	Slc35c2	0	-	MACS_peak_9822|MACS_peak_9823	-10312|-6955
chr2	167318144	167341666	Rnf114	0	+	MACS_peak_9846	8559
chr2	167306635	167318374	Spata2	0	-	MACS_peak_9846	-8789
chr2	167433138	167457505	Ube2v1	0	-	MACS_peak_9848	15002
chr2	167468724	167487044	Tmem189	0	-	MACS_peak_9848	-14537
chr2	167806285	167836093	Fam65c	0	-	MACS_peak_9851	-10234
chr2	168006464	168032562	Adnp	0	-	MACS_peak_9853	-23972
chr2	168750860	168781087	Zfp64	0	-	MACS_peak_9856	-15615
chr2	172073991	172076614	Mc3r	0	+	MACS_peak_9858|MACS_peak_9859|MA(	-14799|-27527|-27678
chr2	173485345	173527683	Rab22a	0	+	MACS_peak_9868	-1633
chr2	173404820	173485040	Ppp4r1l-ps	0	-	MACS_peak_9868	1938
chr2	174276195	174283383	Tubb1	0	+	MACS_peak_9871|MACS_peak_9872	21567|11814
chr2	174241304	174253003	Th1l	0	+	MACS_peak_9871|MACS_peak_9872	-13324|-23077
chr2	174252994	174264493	Ctsz	0	-	MACS_peak_9871|MACS_peak_9872	-9865|-112
chr2	174286575	174289602	Atp5e	0	-	MACS_peak_9872	-25221
chr2	175197962	175210780	Gm14431	0	+	MACS_peak_9874|MACS_peak_9874	-10458|-10458
chr2	175197962	175210780	Gm8898	0	+	MACS_peak_9874|MACS_peak_9874	-10458|-10458
chr2	177677315	177691994	Gm14326	0	-	MACS_peak_9885	-14268
chr2	177757988	177770563	Etohi1	0	+	MACS_peak_9887|MACS_peak_9887	-14722|-14722

chr2	179906292	179910988	Adrm1	0	+	MACS_peak_9893|MACS_peak_9895|MA	16253|9506|-5554|-6525|-10075|-11455|-14300|-15302|-15637|-21392|-21878|-22237|-22435|-23589|-23838|-25663
chr2	179911077	179960564	Lama5	0	-	MACS_peak_9955|MACS_peak_9966|MA	-28609|-19087|-4555
chr2	180669476	180689404	Nkain4	0	-	MACS_peak_9972|MACS_peak_9972|MA	12589|12589|29254|29254
chr2	180702004	180717229	Arfgap1	0	+	MACS_peak_9972|MACS_peak_9972|MA	11|-64|-64|-64|-64|-64|-16654|-16729|-16729|-16729|-16729|-16729
chr2	180721239	180752244	Col20a1	0	+	MACS_peak_9972|MACS_peak_9973	19246|2581
chr2	181316807	181327166	Znf512b	0	-	MACS_peak_9977|MACS_peak_9978	15746|29408
chr2	181303858	181316678	Uckl1	0	-	MACS_peak_9977	26234
chr2	181336023	181390366	Prpf6	0	+	MACS_peak_9977|MACS_peak_9978	-6889|-20551
chr2	181329923	181333852	Samd10	0	-	MACS_peak_9977|MACS_peak_9978	9060|22722
chr2	181313184	181319820	Uckl1os	0	+	MACS_peak_9977|MACS_peak_9977	-29728|-28676
chr2	181404541	181406345	Sox18	0	-	MACS_peak_9981|MACS_peak_9982|MA	-26106|-20509|-16788
chr2	181415014	181422756	Tcea2	0	+	MACS_peak_9982|MACS_peak_9983	29178|25457
chr2	181599064	181605531	Polr3k	0	+	MACS_peak_9984|MACS_peak_9987|MA	26410|19912|12712
chr2	181572607	181592155	Pcmtd2	0	+	MACS_peak_9984|MACS_peak_9987|MA	-47|-6545|-13745
chr2	19579688	19582217	Otud1	0	+	MACS_peak_8922	22
chr2	19577432	19579524	Gm3230	0	-	MACS_peak_8922	142
chr2	23062995	23122649	4931423N	0	+	MACS_peak_8940	12699
chr2	24041995	24049087	Il1f9	0	+	MACS_peak_8942	2565
chr2	25089995	25092307	Gm757	0	-	MACS_peak_8946	19097
chr2	25100338	25110934	Ndor1	0	-	MACS_peak_8946	470
chr2	25126558	25127938	Ssna1	0	-	MACS_peak_8946	-16534
chr2	25094338	25095417	2310002J1	0	-	MACS_peak_8946	15987
chr2	25084417	25089727	Slc34a3	0	-	MACS_peak_8946	21677
chr2	25118117	25125406	Tprn	0	+	MACS_peak_8946	6713
chr2	25127985	25141435	Anapc2	0	+	MACS_peak_8946	16581
chr2	25098448	25099781	Rnf208	0	+	MACS_peak_8946	-12956
chr2	25110958	25111872	Tmem203	0	+	MACS_peak_8946	-446
chr2	25279213	25281891	Fut7	0	+	MACS_peak_8951|MACS_peak_8951|MA	-15477|-15477|-15477|-20890|-20890|-20890
chr2	25284193	25304059	Abca2	0	+	MACS_peak_8951|MACS_peak_8952	-10497|-15910
chr2	25312362	25314292	Clic3	0	+	MACS_peak_8951|MACS_peak_8952	17672|12259
chr2	25315007	25316614	BC029214	0	-	MACS_peak_8951|MACS_peak_8952	-21924|-16511
chr2	25322231	25325269	Ptgds	0	-	MACS_peak_8952	-25166
chr2	26055731	26062076	Lhx3	0	-	MACS_peak_8956	15735
chr2	26064649	26092940	Qsox2	0	-	MACS_peak_8956	-15129
chr2	26313421	26359342	Notch1	0	-	MACS_peak_8961	-22758
chr2	26760786	26766162	Med22	0	-	MACS_peak_8962|MACS_peak_8962	5023|5023
chr2	26746291	26758333	Surf6	0	-	MACS_peak_8962	12852
chr2	26775560	26789031	Surf4	0	-	MACS_peak_8962	-17846
chr2	26768897	26772050	Surf1	0	-	MACS_peak_8962	-865
chr2	26771940	26775690	Surf2	0	+	MACS_peak_8962	755
chr2	26766326	26768831	Rpl7a	0	+	MACS_peak_8962	-4859
chr2	26789588	26809016	Gm711	0	+	MACS_peak_8962	18403
chr2	27119154	27282345	Vav2	0	-	MACS_peak_8965	16783
chr2	28496762	28546687	Tsc1	0	+	MACS_peak_8975	-22331
chr2	28695925	28761094	Ddx31	0	+	MACS_peak_8977	-18412
chr2	28677821	28695880	Gtf3c4	0	-	MACS_peak_8977|MACS_peak_8977	18457|18457
chr2	28907416	28910586	1700101E(	0	-	MACS_peak_8978|MACS_peak_8978	14952|14952
chr2	28915782	28943170	Ttf1	0	+	MACS_peak_8978	-9756
chr2	28980511	29037991	Setx	0	+	MACS_peak_8979	-25062
chr2	29821079	29886968	Spna2	0	+	MACS_peak_8986|MACS_peak_8986|MA	-12936|-12936|-12936|-14613|-14613|-14613|-18126|-18126|-18126|-28092|-28092|-28092|-29927|-29927|-29927
chr2	29887077	29904399	Wdr34	0	-	MACS_peak_9002|MACS_peak_9003|MA	-23896|-22808|-22209|-18355|-17676
chr2	29946463	29949155	Zdhhc12	0	-	MACS_peak_9008|MACS_peak_9008|MA	-13993|-13993|7479|7479
chr2	29917562	29927314	Set	0	+	MACS_peak_9008	-17600
chr2	29934285	29946539	Pkn3	0	+	MACS_peak_9008|MACS_peak_9010	-877|-22349
chr2	29952802	29979974	Zer1	0	-	MACS_peak_9010	-23340
chr2	30028966	30033977	D2Wsu81e	0	-	MACS_peak_9013|MACS_peak_9013|MA	7997|7999|8006|9294|9296|9303
chr2	30027043	30029589	Endog	0	+	MACS_peak_9013|MACS_peak_9014	-14931|-16228
chr2	30040650	30061219	Ccbl1	0	-	MACS_peak_9013|MACS_peak_9014	-19245|-17948
chr2	30122068	30137668	Phyhd1	0	+	MACS_peak_9015|MACS_peak_9016|MA	25913|10296|4245|-331|-10481|-10628|-11138|-11338|-12267|-12576|-12939|-13681|-14527
chr2	30093288	30119310	Lrrc8a	0	+	MACS_peak_9015|MACS_peak_9016|MA	-2867|-18484|-24535|-29111
chr2	30092719	30093151	1700084E1	0	-	MACS_peak_9015|MACS_peak_9016|MA	3004|18621|24672|29248
chr2	30139748	30141874	Dolk	0	-	MACS_peak_9017|MACS_peak_9019|MA	-24051|-19475|-9325|-9178|-8668|-8468|-7539|-7230|-6867|-6125|-5279
chr2	30141952	30199782	Nup188	0	+	MACS_peak_9017|MACS_peak_9019|MA	24129|19553|9403|9256|8746|8546|7617|7308|6945|6203|5357
chr2	30700886	30736765	Prrx2	0	+	MACS_peak_9034	22685
chr2	30678617	30683820	Asb6	0	-	MACS_peak_9034	-5619
chr2	30663496	30678534	Mettl11a	0	+	MACS_peak_9034	-14705
chr2	30651090	30657227	1700001O	0	-	MACS_peak_9034|MACS_peak_9034|MA	20974|19024|19024
chr2	30837798	30878175	Usp20	0	+	MACS_peak_9037|MACS_peak_9038	-28836|-29158
chr2	30828352	30837461	BC005624	0	-	MACS_peak_9037|MACS_peak_9038	29173|29495
chr2	31101442	31150991	Ncs1	0	+	MACS_peak_9043	28889
chr2	31544075	31659747	Abl1	0	+	MACS_peak_9046	-893
chr2	31526256	31536827	Exosc2	0	+	MACS_peak_9046	-18712
chr2	32270007	32286671	Slc25a25	0	-	MACS_peak_9054	-15520
chr2	32240156	32243259	Lcn2	0	-	MACS_peak_9054	27892
chr2	32251409	32258260	Ptges2	0	+	MACS_peak_9054	-19742
chr2	32462480	32476324	St6galnac6	0	+	MACS_peak_9055|MACS_peak_9055|MA	9614|2362|9614
chr2	32426377	32429091	Dpm2	0	+	MACS_peak_9055	-26489
chr2	32443009	32455994	St6galnac4	0	+	MACS_peak_9055	-9857
chr2	32431338	32439298	Pip5kl1	0	+	MACS_peak_9055	-21528
chr2	32632932	32639925	1700019L(	0	-	MACS_peak_9058|MACS_peak_9060	-29856|-6058
chr2	32612790	32617764	Tor2a	0	+	MACS_peak_9058|MACS_peak_9060	2721|-21077
chr2	32617697	32630903	Ttc16	0	-	MACS_peak_9058|MACS_peak_9060	-20834|2964
chr2	32631340	32633113	Ptrh1	0	+	MACS_peak_9058|MACS_peak_9060	21271|-2527
chr2	32817231	32819565	Rpl12	0	+	MACS_peak_9071	-13177
chr2	32828508	32837576	Slc2a8	0	-	MACS_peak_9071	-7168
chr2	32780739	32816771	Lrsam1	0	-	MACS_peak_9071	13637
chr2	32818820	32818938	Snora65	0	+	MACS_peak_9071	-11588
chr2	33305807	33324052	Zbtb43	0	-	MACS_peak_9078|MACS_peak_9078	-44|-44
chr2	34627609	34632049	Hspa5	0	+	MACS_peak_9081|MACS_peak_9081|MA	-183|-183|-585|-585|-1197|-1197
chr2	34532501	34610752	Gapvd1	0	-	MACS_peak_9081|MACS_peak_9082|MA	17040|17442|18054
chr2	34634185	34655311	Rabepk	0	-	MACS_peak_9081|MACS_peak_9082|MA	-27519|-27117|-26505|5883|23023
chr2	34659883	34678701	Fbxw2	0	-	MACS_peak_9088|MACS_peak_9088|MA	-17507|-20561|-20561|-20561|-20561|-367|-3421|-3421|-3421|-3421
chr2	35169509	35192529	Stom	0	-	MACS_peak_9113	-13303
chr2	35231081	35252695	4930402F(	0	-	MACS_peak_9118|MACS_peak_9119	10795|16208
chr2	35547513	35586514	Dab2ip	0	+	MACS_peak_9121	-29339
chr2	37205593	37214852	Pdcl	0	-	MACS_peak_9126|MACS_peak_9127	-7735|-3607
chr2	37187268	37188252	Olfr368	0	+	MACS_peak_9126|MACS_peak_9127	-19849|-23977
chr2	37372145	37374801	Gpr21	0	+	MACS_peak_9131	-20712
chr2	4543212	4573132	Prpf18	0	-	MACS_peak_8870	-16196
chr2	4911770	4933837	Mcm10	0	-	MACS_peak_8875	18536
chr2	52547724	52601183	Stam2	0	-	MACS_peak_9149	-26421
chr2	52716901	52993236	Fmnl2	0	+	MACS_peak_9150	-237
chr2	5214502	5635710	Camk1d	0	-	MACS_peak_8887	-404
chr2	56959637	56976414	Nr4a2	0	-	MACS_peak_9165|MACS_peak_9165	-13271|-4306
chr2	5766079	5789782	Nudt5	0	+	MACS_peak_8888	-28750
chr2	5792032	5816399	Sec61a2	0	-	MACS_peak_8888|MACS_peak_8889|MA	-21570|-12726|-8812
chr2	5715339	5766006	Cdc123	0	-	MACS_peak_8888	28823
chr2	59450100	59684206	Tanc1	0	+	MACS_peak_9174	-74
chr2	59693256	59720663	Wdsub1	0	-	MACS_peak_9187|MACS_peak_9187	-4231|-4231
chr2	60090049	60122475	Cd302	0	-	MACS_peak_9191|MACS_peak_9192	8743|23832
chr2	6019545	6051231	5430407P	0	-	MACS_peak_8891|MACS_peak_8891|MA	-29867|-29867|-29384|-29384
chr2	60257095	60391318	Pla2r1	0	-	MACS_peak_9196	24233
chr2	60436349	60560660	Itgb6	0	-	MACS_peak_9197	29874
chr2	60590009	60801261	Rbms1	0	-	MACS_peak_9198	-64
chr2	68973856	69027384	Nostrin	0	+	MACS_peak_9207	26741
chr2	69218518	69241143	Dhrs9	0	+	MACS_peak_9215|MACS_peak_9216|MA	-24975|-25219|-25441|-25760|-25933|-26310|-26601|-26779|-27292
chr2	69561144	69592116	Ppig	0	+	MACS_peak_9225	20325
chr2	69524880	69550663	Fastkd1	0	-	MACS_peak_9225	-9844
chr2	69735302	69862070	Ubr3	0	+	MACS_peak_9226|MACS_peak_9226	-511|-511
chr2	69709251	69723661	Mettl5	0	-	MACS_peak_9226	12152
chr2	71050069	71101351	Dync1i2	0	+	MACS_peak_9231	-16598
chr2	71625139	71694815	Itga6	0	+	MACS_peak_9233|MACS_peak_9234	-113|-29610

chr2	71711328	71741914	Pdk1	0	+	MACS_peak_9245|MACS_peak_9246|MA	27494|27056|19695|16148
chr2	72123757	72245556	B230120H	0	+	MACS_peak_9249|MACS_peak_9249|MA	15065|15001|15065
chr2	73109982	73113826	Sp9	0	+	MACS_peak_9253	-11275
chr2	73121928	73150649	Cir1	0	-	MACS_peak_9253	-29392
chr2	73150708	73175864	Scrn3	0	+	MACS_peak_9253	29451
chr2	6460743	7317825	Celf2	0	-	MACS_peak_8896|MACS_peak_8896	12466|12664
chr2	77733709	77784240	Cwc22	0	-	MACS_peak_9270	-9531
chr2	83652884	83721515	Fam171b	0	+	MACS_peak_9285|MACS_peak_9286|MA	27312|23745|17769
chr2	83795612	83798922	Gm13698	0	+	MACS_peak_9303|MACS_peak_9303|MA	-1361|4449|10259|16069|21879|27689|-17864|-12054|-6244|-434|5376|11186|16996|-20540|-14730|-8920|-3110|2700|8510|14320
chr2	83795612	83798922	Gm13693	0	+	MACS_peak_9303|MACS_peak_9303|MA	-1361|4449|10259|16069|21879|27689|-17864|-12054|-6244|-434|5376|11186|16996|-20540|-14730|-8920|-3110|2700|8510|14320
chr2	83795612	83798922	Gm13696	0	+	MACS_peak_9303|MACS_peak_9303|MA	-1361|4449|10259|16069|21879|27689|-17864|-12054|-6244|-434|5376|11186|16996|-20540|-14730|-8920|-3110|2700|8510|14320
chr2	83795612	83798922	Gm13694	0	+	MACS_peak_9303|MACS_peak_9303|MA	-1361|4449|10259|16069|21879|27689|-17864|-12054|-6244|-434|5376|11186|16996|-20540|-14730|-8920|-3110|2700|8510|14320
chr2	83795612	83798922	Gm13697	0	+	MACS_peak_9303|MACS_peak_9303|MA	-1361|4449|10259|16069|21879|27689|-17864|-12054|-6244|-434|5376|11186|16996|-20540|-14730|-8920|-3110|2700|8510|14320
chr2	83795612	83798922	Gm13691	0	+	MACS_peak_9303|MACS_peak_9303|MA	-1361|4449|10259|16069|21879|27689|-17864|-12054|-6244|-434|5376|11186|16996|-20540|-14730|-8920|-3110|2700|8510|14320
chr2	83795612	83798922	Gm13695	0	+	MACS_peak_9303|MACS_peak_9303|MA	-1361|4449|10259|16069|21879|27689|-17864|-12054|-6244|-434|5376|11186|16996|-20540|-14730|-8920|-3110|2700|8510|14320
chr2	83755235	83781383	Zswim2	0	-	MACS_peak_9303	15590
chr2	84170790	84265423	Calcrl	0	-	MACS_peak_9306|MACS_peak_9307|MA	7755|18811|19562|28635
chr2	84273016	84314331	Tfpi	0	-	MACS_peak_9308|MACS_peak_9309|MA	-29346|-22858|-22874|-20273
chr2	84679564	84703743	Slc43a1	0	+	MACS_peak_9324|MACS_peak_9324|MA	9426|10644|10341
chr2	84667177	84670370	Timm10	0	+	MACS_peak_9324	-2961
chr2	84651332	84662809	Smtnl1	0	-	MACS_peak_9324	7329
chr2	84776812	84798666	Slc43a3	0	+	MACS_peak_9329	-7623
chr2	84820617	84823789	Prg2	0	+	MACS_peak_9337|MACS_peak_9338|MA	-16969|-17384|-17630|-17820|-18380
chr2	84828371	84834043	Prg3	0	+	MACS_peak_9337|MACS_peak_9338|MA	-9215|-9630|-9876|-10066|-10626
chr2	84890616	84913205	Tnks1bp1	0	+	MACS_peak_9342|MACS_peak_9343|MA	28668|25547|17337|-1779|-8309|-12480|-19717|-22359
chr2	84877607	84887263	Ssrp1	0	+	MACS_peak_9342|MACS_peak_9342|MA	15659|15409|12538|12288|4328|4078|-14788|-15038|-21318|-21568|-25489|-25739
chr2	84836708	84875991	P2rx3	0	-	MACS_peak_9342|MACS_peak_9343|MA	-14043|-10922|-2712|16404|22934|27105
chr2	85514367	85518898	Olfr1006	0	-	MACS_peak_9350	21559
chr2	85561563	85562506	Olfr1009	0	+	MACS_peak_9350	21106
chr2	85529587	85530529	Olfr1008	0	+	MACS_peak_9350	-10870
chr2	90269912	90319331	Ptprj	0	-	MACS_peak_9363	-14635
chr2	90894172	90899594	Psmc3	0	+	MACS_peak_9366	-15318
chr2	90936953	90955913	Sfpi1	0	+	MACS_peak_9366	27463
chr2	90901947	90910378	Slc39a13	0	-	MACS_peak_9366	-888
chr2	91105271	91117528	Arfgap2	0	+	MACS_peak_9368|MACS_peak_9368|MA	28349|28349|3825|3825|1767|1767
chr2	91051740	91077223	Ddb2	0	-	MACS_peak_9368|MACS_peak_9369|MA	-301|24223|26281
chr2	91097485	91104836	Pacsin3	0	+	MACS_peak_9368|MACS_peak_9368|MA	20563|20047|-3961|-4477|-6019|-6535
chr2	91077302	91078512	A330069E	0	+	MACS_peak_9368|MACS_peak_9369|MA	380|-24144|-26202
chr2	91483844	91489948	Zfp408	0	-	MACS_peak_9372|MACS_peak_9374	-5756|29562
chr2	91490274	91512475	Arhgap1	0	+	MACS_peak_9372|MACS_peak_9372|MA	6082|9172|6082|6082|6082|-29236|-26146|-29236|-29236|-29236
chr2	91465476	91476571	F2	0	-	MACS_peak_9372	7621
chr2	91570294	91759006	Ambra1	0	+	MACS_peak_9376|MACS_peak_9376	19727|19727
chr2	91514781	91550733	Atg13	0	-	MACS_peak_9376	-166
chr2	91551106	91561723	Harbi1	0	+	MACS_peak_9376	539
chr2	92061718	92204823	Phf21a	0	+	MACS_peak_9389	15
chr2	92032133	92032230	Mir1955	0	+	MACS_peak_9389	-29570
chr2	92205203	92211193	Gyltl1b	0	-	MACS_peak_9397|MACS_peak_9397|MA	-7418|-7418|-6835|-6835
chr2	92215395	92221376	Pex16	0	+	MACS_peak_9397|MACS_peak_9397|MA	11620|11057|11037|10474
chr2	92223074	92223758	1700029I1	0	+	MACS_peak_9397|MACS_peak_9398	19299|18716
chr2	92251416	92274226	Cry2	0	-	MACS_peak_9402|MACS_peak_9402	-11089|-11089
chr2	92223836	92241420	Mapk8ip1	0	-	MACS_peak_9402	21717
chr2	93535787	93662725	Ext2	0	-	MACS_peak_9415	3040
chr2	93675609	93689933	Accs	0	-	MACS_peak_9415	-24168
chr2	94140922	94246846	Ttc17	0	-	MACS_peak_9425	-27919
chr2	94246863	94251835	2810002D	0	+	MACS_peak_9425	27936
chr3	101079830	101091862	Cd2	0	-	MACS_peak_10459|MACS_peak_10460	18680|18795
chr3	101380145	101408580	Atp1a1	0	-	MACS_peak_10476|MACS_peak_10479|N	-27701|-23450|-15596|-14785|-9605
chr3	102581320	102586637	Tshb	0	-	MACS_peak_10495|MACS_peak_10495|N	-11956|-11956|-11956
chr3	102824468	102862111	Csde1	0	+	MACS_peak_10496|MACS_peak_10496|N	17492|17492|-19947|-19947
chr3	102799662	102807837	Sike1	0	+	MACS_peak_10496	-7314
chr3	102862207	102871837	Nras	0	+	MACS_peak_10497	17792
chr3	103539316	103541924	Olfml3	0	-	MACS_peak_10499	3479
chr3	103543737	103595198	Hipk1	0	-	MACS_peak_10500|MACS_peak_10501	-13514|18608
chr3	103604527	103613310	Dclre1b	0	-	MACS_peak_10501|MACS_peak_10501	496|496
chr3	103636618	103642570	Bcl2l15	0	+	MACS_peak_10501|MACS_peak_10501	22812|22812
chr3	103613439	103625946	Ap4b1	0	+	MACS_peak_10501|MACS_peak_10501|N	-367|-367|-367
chr3	104804834	104855871	Cttnbp2nl	0	-	MACS_peak_10511|MACS_peak_10511|N	-20247|-20247|-20440
chr3	105776719	105790341	Ovgp1	0	+	MACS_peak_10513	-6380
chr3	105745780	105763166	Atp5f1	0	-	MACS_peak_10513	19933
chr3	105762415	105772678	Wdr77	0	+	MACS_peak_10513	-20684
chr3	105799874	105804415	1700027A	0	-	MACS_peak_10513	-21316
chr3	106285348	106303972	Dennd2d	0	+	MACS_peak_10514|MACS_peak_10514|N	-20934|-17315|-22156|-18537|-22801|-19182
chr3	106284903	106288831	2010016I1	0	-	MACS_peak_10514|MACS_peak_10515|N	17451|18673|19318
chr3	106350744	106377763	Dram2	0	+	MACS_peak_10517|MACS_peak_10517|N	16617|16617|8428|8428|-2762|-2762|-7410|-7410|-18287|-18287|-26035|-26035
chr3	106305177	106350679	Cept1	0	-	MACS_peak_10517|MACS_peak_10519|N	-16552|-8363|2827|7475|18352|26100
chr3	107129027	107136207	Rbm15	0	-	MACS_peak_10524	-500
chr3	107397948	107408687	Alx3	0	+	MACS_peak_10525	-26932
chr3	107415449	107434628	Fam40a	0	-	MACS_peak_10525	-9748
chr3	107543965	107563387	Csf1	0	-	MACS_peak_10527|MACS_peak_10527|N	-18367|-18367|-18367|-5009|-5009|-5009
chr3	107698771	107701603	Gstm5	0	+	MACS_peak_10529	-2656
chr3	107680147	107695817	Eps8l3	0	+	MACS_peak_10529	-21280
chr3	107691769	107699131	4933431E	0	-	MACS_peak_10529	2296
chr3	108193765	108218412	Celsr2	0	-	MACS_peak_10538|MACS_peak_10538|N	-29143|-29143|11304|11304|12742|12742|13185|13185|13845|13845|16034|16034|17638|17638|18633|18633|20385|20385|22049|220
chr3	108186721	108191149	Psrc1	0	+	MACS_peak_10538|MACS_peak_10538	-2548|-2514
chr3	108167828	108182975	Mybphl	0	+	MACS_peak_10538	-21441
chr3	108229529	108248087	Sars	0	-	MACS_peak_10541|MACS_peak_10543|N	-18371|-16933|-16490|-15830|-13641|-12037|-11042|-9290|-7626|-66
chr3	108260986	108339440	5330417C	0	-	MACS_peak_10553	25605
chr3	108374616	108384986	Taf13	0	+	MACS_peak_10553	9571
chr3	108359342	108365384	Tmem167b	0	-	MACS_peak_10553	-339
chr3	108340501	108347615	1700013F	0	+	MACS_peak_10553	-24544
chr3	108394195	108448637	Wdr47	0	+	MACS_peak_10553	29150
chr3	108357259	108357320	Scarna2	0	-	MACS_peak_10553	7725
chr3	108481555	108525217	Gpsm2	0	-	MACS_peak_10555	-25259
chr3	115413351	115417973	S1pr1	0	-	MACS_peak_10561|MACS_peak_10562|N	-3198|-2603|-228
chr3	115643454	115647675	Gm6649	0	+	MACS_peak_10564	-4174
chr3	116265890	116276030	Lrrc39	0	+	MACS_peak_10565|MACS_peak_10565	14341|14341
chr3	116442916	116511084	Agl	0	-	MACS_peak_10568	13621
chr3	121948509	121969566	Gclm	0	+	MACS_peak_10583	-13656
chr3	121977331	121988189	Dnttip2	0	+	MACS_peak_10583	15166
chr3	122122697	122233098	Bcar3	0	+	MACS_peak_10588	-6822
chr3	122627314	122629112	1810037I1	0	+	MACS_peak_10597|MACS_peak_10599	-9798|-28230
chr3	122636518	122687365	Usp53	0	-	MACS_peak_10600	-20145
chr3	129604342	129617020	Casp6	0	+	MACS_peak_10621	22616
chr3	129581539	129598739	Pla2g12a	0	+	MACS_peak_10621|MACS_peak_10621	-187|2375
chr3	130993408	131006145	Cyp2u1	0	-	MACS_peak_10628	21798
chr3	131021905	131047841	Sgms2	0	-	MACS_peak_10628	-19898
chr3	136333733	136598743	Ppp3ca	0	+	MACS_peak_10637	259
chr3	137581518	137591726	Mapksp1	0	+	MACS_peak_10642	12565
chr3	137711724	137730352	Gm5105	0	-	MACS_peak_10643	23719
chr3	146113456	146128735	Ctbs	0	+	MACS_peak_10652|MACS_peak_10656|N	-3930|-15956|-17238
chr3	152009444	152056578	Usp33	0	+	MACS_peak_10678|MACS_peak_10678|N	11156|11156|-11423|-11423
chr3	151936425	152003371	Fam73a	0	-	MACS_peak_10678|MACS_peak_10678|N	-5083|-5083|17496|17496
chr3	152058973	152125790	Zzz3	0	+	MACS_peak_10680|MACS_peak_10680	16488|17172
chr3	152125778	152331104	Ak5	0	-	MACS_peak_10684	-7518
chr3	152377063	152451975	Pigk	0	+	MACS_peak_10685|MACS_peak_10685	-24157|-24157
chr3	154756397	154857242	Lrriq3	0	+	MACS_peak_10689	8136
chr3	154747882	154756342	Fpgt	0	-	MACS_peak_10689	-8081
chr3	154449254	154718371	Tnni3k	0	-	MACS_peak_10689	29890
chr3	157557211	157588027	Cth	0	-	MACS_peak_10692|MACS_peak_10693|N	-27803|-18820|-17669|-14158|-11397|-6629|-5000|-4095|-100
chr3	157610429	157669801	Ankrd13c	0	+	MACS_peak_10697|MACS_peak_10698|N	29031|27402|26497|22502
chr3	26996144	27052776	Ect2	0	-	MACS_peak_10032|MACS_peak_10032|N	29248|29224|29248
chr3	27081925	27143833	Nceh1	0	+	MACS_peak_10032	-99

chr3	30543428	30571353	Lrriq4	0	+	MACS_peak_10039	26435
chr3	30523188	30546740	Lrrc34	0	-	MACS_peak_10039	-29747
chr3	30495994	30498792	Arpm1	0	-	MACS_peak_10039	18201
chr3	30501008	30517416	Mynn	0	+	MACS_peak_10039	-15985
chr3	30798217	30868337	Phc3	0	-	MACS_peak_10053|MACS_peak_10053|N	-18464|-18464|-18464|-18464
chr3	32335072	32367408	Pik3ca	0	+	MACS_peak_10060	-6629
chr3	36762027	36951955	4932438A:	0	+	MACS_peak_10066	-22350
chr3	37319201	37478017	Spata5	0	+	MACS_peak_10071|MACS_peak_10071	-12927|-12927
chr3	37303905	37318512	Nudt6	0	-	MACS_peak_10071	13616
chr3	37538871	37543521	Spry1	0	+	MACS_peak_10074	-3725
chr3	38348182	38383738	Ankrd50	0	-	MACS_peak_10079	-21806
chr3	40603872	40620805	Plk4	0	+	MACS_peak_10085|MACS_peak_10089	20567|-19533
chr3	40622093	40650776	Mfsd8	0	-	MACS_peak_10089	-27371
chr3	50168857	50247535	Slc7a11	0	-	MACS_peak_10107	-8204
chr3	51028368	51055576	Ccrn4l	0	+	MACS_peak_10110	-17088
chr3	52786044	52821145	Cog6	0	-	MACS_peak_10122|MACS_peak_10123|N	-9934|-6581|-139|24434
chr3	52845468	53065601	Lhfp	0	+	MACS_peak_10124|MACS_peak_10125	24462|-111
chr3	53292714	53311574	Stoml3	0	+	MACS_peak_10131|MACS_peak_10132|N	27083|26408|7448
chr3	53255917	53267180	Nhlrc3	0	-	MACS_peak_10131|MACS_peak_10132|N	-1549|-874|18086
chr3	53267738	53285677	2810046L(	0	+	MACS_peak_10131|MACS_peak_10132|N	2107|1432|-17528
chr3	54497026	54520759	Fam48a	0	+	MACS_peak_10135|MACS_peak_10136|N	13588|-8448|-22558|-29437
chr3	54532600	54539286	Exosc8	0	-	MACS_peak_10137|MACS_peak_10137|N	-19702|-19702|-12823|-12823
chr3	54539460	54553717	Alg5	0	+	MACS_peak_10137|MACS_peak_10139	19876|12997
chr3	54916087	54941253	Spg20	0	+	MACS_peak_10140|MACS_peak_10140|N	-278|-336|-336
chr3	54943959	54976857	A730037C:	0	+	MACS_peak_10140	27594
chr3	55651026	55651987	4933417G(	0	+	MACS_peak_10149	-9912
chr3	57539987	57639155	Rnf13	0	+	MACS_peak_10156|MACS_peak_10156	-28253|-28253
chr3	57645817	57651679	Pfn2	0	-	MACS_peak_10159	-13647
chr3	58478870	58496310	Siah2	0	-	MACS_peak_10161	-16650
chr3	58455963	58457963	Gm8234	0	+	MACS_peak_10161	-23697
chr3	59533707	59556318	Gm5538	0	+	MACS_peak_10164	-7425
chr3	62272563	62310910	Dhx36	0	-	MACS_peak_10167	-11491
chr3	66023808	66029728	Ptx3	0	+	MACS_peak_10173	-4974
chr3	69120839	69359318	Ppm1l	0	+	MACS_peak_10182	-18317
chr3	82859459	82876213	Plrg1	0	+	MACS_peak_10224	-16661
chr3	82846226	82853712	Fgb	0	-	MACS_peak_10224	22408
chr3	83891855	83959708	Mnd1	0	-	MACS_peak_10233	5009
chr3	84247946	84284351	Fhdc1	0	-	MACS_peak_10236|MACS_peak_10237|N	-26876|-23388|-23203|-5970
chr3	84768977	84769502	Dear1	0	-	MACS_peak_10240	12490
chr3	84756132	84783119	Fbxw7	0	+	MACS_peak_10240	-25860
chr3	86882513	86978669	Kirrel	0	-	MACS_peak_10253|MACS_peak_10253	-241|-241
chr3	87422672	87541955	Arhgef11	0	+	MACS_peak_10255	498
chr3	87540844	87552545	4933430H	0	-	MACS_peak_10262|MACS_peak_10263|N	-26211|-23055|-20637|-17382|-14114|-12727|3589|4148
chr3	87553018	87572875	Pear1	0	-	MACS_peak_10270|MACS_peak_10270|N	-16741|-16741|-16741|-16741|-16182|-16182|-16182|-16182
chr3	87752614	87757294	Crabp2	0	+	MACS_peak_10273	26250
chr3	87710242	87720054	Hdgf	0	+	MACS_peak_10273	-16122
chr3	87723465	87727355	Mrpl24	0	+	MACS_peak_10273	-2899
chr3	87726524	87734117	BC023814	0	-	MACS_peak_10273	-7753
chr3	87734235	87744608	Isg20l2	0	+	MACS_peak_10273	7871
chr3	87796435	87804278	Bcan	0	-	MACS_peak_10277|MACS_peak_10277	-21477|-21477
chr3	87775014	87784373	Nes	0	+	MACS_peak_10277	-7787
chr3	88383592	88392335	Ssr2	0	+	MACS_peak_10280	-8477
chr3	88425027	88451974	Arhgef2	0	+	MACS_peak_10282|MACS_peak_10283|N	4499|-8751|-10838|-11871|-13429|-13936|-14872|-18302|-21440|-22077|-22905|-25167|-26792|-28140
chr3	88455819	88457064	Rxfp4	0	-	MACS_peak_10283|MACS_peak_10284|N	-23286|-21199|-20166|-18608|-18101|-17165|-13735|-10597|-9960|-9132|-6870|-5245|-3897
chr3	88639149	88714020	Gon4l	0	+	MACS_peak_10302	21476
chr3	88625931	88636409	1500004A:	0	-	MACS_peak_10302	-18736
chr3	89038098	89049121	Trim46	0	-	MACS_peak_10309|MACS_peak_10309|N	-10449|-11147|-1451|-2149
chr3	89013003	89018257	Mtx1	0	-	MACS_peak_10309|MACS_peak_10309|N	20415|20415|29413|29413
chr3	89032973	89037291	Muc1	0	+	MACS_peak_10309|MACS_peak_10310	-5699|-14697
chr3	89019108	89030757	Thbs3	0	+	MACS_peak_10309|MACS_peak_10310	-19564|-28562
chr3	89050359	89053644	Krtcap2	0	+	MACS_peak_10309|MACS_peak_10310|N	11687|2689|-29842
chr3	89031037	89031120	Mir92b	0	-	MACS_peak_10309|MACS_peak_10310	7552|16550
chr3	89072167	89074492	Rag1ap1	0	-	MACS_peak_10310|MACS_peak_10312	-26822|5709
chr3	89070473	89070999	Dpm3	0	+	MACS_peak_10310|MACS_peak_10312	22803|-9728
chr3	89075651	89084873	Efna1	0	-	MACS_peak_10312|MACS_peak_10312	-4672|-3366
chr3	89799378	89802608	Hax1	0	-	MACS_peak_10322	-1206
chr3	90070455	90084587	Dennd4b	0	+	MACS_peak_10324	-26834
chr3	90145575	90162042	Gatad2b	0	+	MACS_peak_10327|MACS_peak_10329	-9833|-12754
chr3	90189154	90193849	Slc27a3	0	-	MACS_peak_10333	20123
chr3	90195309	90237558	Ints3	0	-	MACS_peak_10333|MACS_peak_10333	-23586|-23586
chr3	90330770	90332755	S100a14	0	+	MACS_peak_10334|MACS_peak_10334|N	11883|11883|11883
chr3	90318681	90328503	S100a13	0	+	MACS_peak_10334	-206
chr3	90314955	90318252	S100a1	0	-	MACS_peak_10334	635
chr3	90302879	90313365	2500003M	0	-	MACS_peak_10334	5522
chr3	90345144	90347073	S100a16	0	+	MACS_peak_10334	26257
chr3	90291948	90294935	Snapin	0	-	MACS_peak_10334	23952
chr3	92019756	92026413	Sprr2a2	0	+	MACS_peak_10338|MACS_peak_10338	-12355|22457
chr3	92019756	92026413	Sprr2a1	0	+	MACS_peak_10338|MACS_peak_10338	-12355|22457
chr3	94641488	94687487	Pogz	0	+	MACS_peak_10343|MACS_peak_10343	-24918|-24918
chr3	94688245	94690880	Psmb4	0	-	MACS_peak_10343|MACS_peak_10344	-24474|-9963
chr3	94836626	94846467	Psmd4	0	-	MACS_peak_10347|MACS_peak_10348|N	16854|21886|24875|29917
chr3	94968295	94977238	Sema6c	0	+	MACS_peak_10360	-2682
chr3	94943442	94946282	Tnfaip8l2	0	-	MACS_peak_10360	24695
chr3	95040841	95045031	Gm128	0	-	MACS_peak_10361	-21310
chr3	95032873	95038499	Cdc42se1	0	+	MACS_peak_10361|MACS_peak_10361	9152|8980
chr3	95022465	95032599	Mllt11	0	-	MACS_peak_10361	-8878
chr3	94985689	95021864	Gabpb2	0	-	MACS_peak_10361|MACS_peak_10361	1857|1857
chr3	95462642	95467101	Mcl1	0	+	MACS_peak_10376	-24644
chr3	95480126	95491781	Adamtsl4	0	-	MACS_peak_10376	-4495
chr3	95682427	95686151	Gm129	0	-	MACS_peak_10390|MACS_peak_10391	-3165|-775
chr3	95701722	95708562	Car14	0	-	MACS_peak_10390|MACS_peak_10391	-25576|-23186
chr3	95634544	95659676	Prpf3	0	-	MACS_peak_10390|MACS_peak_10391	23310|25700
chr3	95666574	95674542	Mrps21	0	-	MACS_peak_10390|MACS_peak_10391	8444|10834
chr3	95697918	95702213	Aph1a	0	+	MACS_peak_10390|MACS_peak_10390|N	14932|14991|12542|12601
chr3	95688895	95695853	BC028528	0	-	MACS_peak_10390|MACS_peak_10391	-12867|-10477
chr3	95976472	95981487	Sf3b4	0	+	MACS_peak_10394	28258
chr3	95965892	95975640	Mtmr11	0	+	MACS_peak_10394	17678
chr3	96361879	96365780	Txnip	0	+	MACS_peak_10412|MACS_peak_10412	-24205|-24205
chr3	96400558	96403701	Ankrd34a	0	+	MACS_peak_10412|MACS_peak_10413	14474|2518
chr3	96405055	96429275	Lix1l	0	+	MACS_peak_10412|MACS_peak_10413	18971|7015
chr3	96381796	96398081	Polr3gl	0	-	MACS_peak_10412|MACS_peak_10413	-11997|-41
chr3	96449506	96468442	Itga10	0	+	MACS_peak_10428	-10906
chr3	96474053	96494957	Ankrd35	0	+	MACS_peak_10428	13641
chr3	96433850	96437714	Rbm8a	0	+	MACS_peak_10428|MACS_peak_10428	-26562|-26562
chr3	96439352	96449304	Pex11b	0	+	MACS_peak_10428|MACS_peak_10428|N	-21060|-20643|-21060|-21060|-21133
chr3	96410584	96433742	6330549D:	0	-	MACS_peak_10428	26670
chr3	96962699	96980499	Acp6	0	+	MACS_peak_10436|MACS_peak_10437	-296|-16932
chr3	97542766	97571937	Pde4dip	0	-	MACS_peak_10441|MACS_peak_10442|N	-16103|-14916|-13821|-12495
chr3	98117093	98143892	Phgdh	0	-	MACS_peak_10450|MACS_peak_10451|N	-26654|-25676|-23470
chr4	102262447	102279866	Pde4b	0	+	MACS_peak_11028|MACS_peak_11028	-11775|-14060
chr4	106584074	106722299	Ssbp3	0	+	MACS_peak_11047|MACS_peak_11047	-1764|-1764
chr4	107854539	107890527	Zyg11a	0	-	MACS_peak_11064	27036
chr4	108551674	108615929	Rab3b	0	+	MACS_peak_11068|MACS_peak_11069	25879|21108
chr4	108507282	108534724	Txndc12	0	+	MACS_peak_11068|MACS_peak_11069	-18513|-23284
chr4	108486899	108506189	Btf3l4	0	-	MACS_peak_11068|MACS_peak_11069	19606|24377
chr4	108520461	108522017	Kti12	0	+	MACS_peak_11068|MACS_peak_11069	-5334|-10105
chr4	108690259	108690334	Mir761	0	+	MACS_peak_11073	-14802
chr4	108733749	108790641	Osbpl9	0	-	MACS_peak_11074|MACS_peak_11074	15743|15743
chr4	109015660	109060415	Eps15	0	+	MACS_peak_11076	-267
chr4	109125461	109149110	Rnf11	0	-	MACS_peak_11077|MACS_peak_11078	-23016|-250
chr4	11413104	11478290	1110037F(	0	+	MACS_peak_10732|MACS_peak_10733	-8871|-22082
chr4	115557004	115560569	Kncn	0	+	MACS_peak_11085|MACS_peak_11086|N	24333|21355|8936|5839

chr4	115511850	115551853	Mknk1	0	+	MACS_peak_11085|MACS_peak_11086	-20821|-23799
chr4	116269334	116272871	Ccdc17	0	+	MACS_peak_11091|MACS_peak_11092|M	25799|22253|6502|5408
chr4	116230331	116266487	Gpbp1l1	0	+	MACS_peak_11091|MACS_peak_11092	-13204|-16750
chr4	116224132	116228548	Tmem69	0	-	MACS_peak_11091|MACS_peak_11092	14987|18533
chr4	116262337	116270235	C530005A1	0	-	MACS_peak_11091|MACS_peak_11092|M	-26700|-23154|-7403|-6309
chr4	116358203	116372605	Prdx1	0	+	MACS_peak_11099	-24594
chr4	116375038	116380990	Mmachc	0	-	MACS_peak_11099	1807
chr4	116381534	116387709	Ccdc163	0	+	MACS_peak_11099	-1263
chr4	116393559	116476853	Tesk2	0	+	MACS_peak_11099	10762
chr4	116692012	116759457	Eif2b3	0	+	MACS_peak_11101|MACS_peak_11101	19115|19115
chr4	116662821	116666980	Urod	0	-	MACS_peak_11101	5917
chr4	116667952	116677882	Hectd3	0	+	MACS_peak_11101	-4945
chr4	116826440	116828737	Rps8	0	-	MACS_peak_11102|MACS_peak_11104|M	-27185|-25808|-25013|-24698|-24425|-24185|-23377|-23140|-22687
chr4	116801259	116806557	Plk3	0	-	MACS_peak_11102|MACS_peak_11104|M	-5005|-3628|-2833|-2518|-2245|-2005|-1197|-960|-507
chr4	116799417	116801335	Tctex1d4	0	+	MACS_peak_11102|MACS_peak_11104|M	-2135|-3512|-4307|-4622|-4895|-5135|-5943|-6180|-6633
chr4	116791822	116798330	Btbd19	0	-	MACS_peak_11102|MACS_peak_11104|M	3222|4599|5394|5709|5982|6222|7030|7267|7720
chr4	116827120	116827179	Snord38a	0	-	MACS_peak_11102|MACS_peak_11104|M	-25627|-24250|-23455|-23140|-22867|-22627|-21819|-21582|-21129
chr4	116828375	116828453	Snord55	0	-	MACS_peak_11102|MACS_peak_11104|M	-26901|-25524|-24729|-24414|-24141|-23901|-23093|-22856|-22403
chr4	116887794	116924522	Gm1661	0	-	MACS_peak_11114|MACS_peak_11114	25866|25866
chr4	116924593	116941185	Tmem53	0	+	MACS_peak_11114	-25795
chr4	117541867	117545075	Ccdc24	0	-	MACS_peak_11125|MACS_peak_11126|M	-29400|-16515|-15717|-15137|-8976|-8712|-7944|-7423|-4628
chr4	117507862	117541910	Slc6a9	0	+	MACS_peak_11125|MACS_peak_11126|M	-7813|-20698|-21496|-22076|-28237|-28501|-29269|-29790
chr4	117545623	117556074	B4galt2	0	-	MACS_peak_11126|MACS_peak_11127|M	-27514|-26716|-26136|-19975|-19711|-18943|-18422|-15627
chr4	117556934	117559934	Atp6v0b	0	-	MACS_peak_11128|MACS_peak_11129|M	-29996|-23835|-23571|-22803|-22282|-19487
chr4	117561247	117564608	Dph2	0	-	MACS_peak_11129|MACS_peak_11130|M	-28509|-28245|-27477|-26956|-24161
chr4	117604757	117807519	St3gal3	0	-	MACS_peak_11139|MACS_peak_11139|M	7550|7574|26837|26861
chr4	117809610	117852648	Kdm4a	0	-	MACS_peak_11145|MACS_peak_11145|M	-18292|-18292|-11562|-11562|-10024|-10024
chr4	118781349	118809934	Slc2a1	0	+	MACS_peak_11152|MACS_peak_11153|M	-120|-23460|-23819|-24441|-27085
chr4	118897743	118905329	AU022252	0	-	MACS_peak_11158|MACS_peak_11161	454|5992
chr4	118928073	118932460	Cldn19	0	+	MACS_peak_11158|MACS_peak_11158|M	22290|22262|16752|16724
chr4	118906433	118921582	Lepre1	0	+	MACS_peak_11158|MACS_peak_11158|M	650|-264|-264|-4888|-5802|-5802
chr4	118877659	118890822	4930538K1	0	-	MACS_peak_11158|MACS_peak_11161	14961|20499
chr4	11885331	11893597	Pdp1	0	-	MACS_peak_10735|MACS_peak_10735|M	-94|-94|643|13862|13862|14599
chr4	120204835	120209266	Slfnl1	0	+	MACS_peak_11169	4006
chr4	120602743	120624306	Zfp69	0	-	MACS_peak_11171	-9061
chr4	120593806	120597610	Dem1	0	-	MACS_peak_11171|MACS_peak_11171	17635|17635
chr4	122782407	122803334	Bmp8b	0	+	MACS_peak_11180	13740
chr4	122793490	122795243	Oxct2b	0	+	MACS_peak_11180	24823
chr4	122989892	123020046	Bmp8a	0	-	MACS_peak_11181	7614
chr4	122999118	123000877	Oxct2a	0	-	MACS_peak_11181	26783
chr4	123080843	123089154	D830031N	0	-	MACS_peak_11184|MACS_peak_11185	-29731|-28752
chr4	124479792	124527044	Mtf1	0	+	MACS_peak_11198	1465
chr4	125781200	125818272	Stk40	0	+	MACS_peak_11201|MACS_peak_11201|M	-21170|-21170|-24986|-24986
chr4	125773896	125775827	Lsm10	0	+	MACS_peak_11201|MACS_peak_11201	-28474|-28474
chr4	125825246	125827118	Fam176b	0	+	MACS_peak_11201|MACS_peak_11202	22876|19060
chr4	125999364	126002443	Tekt2	0	-	MACS_peak_11205|MACS_peak_11207	16436|17852
chr4	125993594	125998947	Adprhl2	0	-	MACS_peak_11205|MACS_peak_11207	19932|21348
chr4	126112258	126145665	Eif2c1	0	-	MACS_peak_11208|MACS_peak_11209	-4660|42
chr4	126430798	126546938	AU040320	0	+	MACS_peak_11210|MACS_peak_11210|M	313|528|528
chr4	126420993	126430673	Ncdn	0	-	MACS_peak_11210	-188
chr4	126393246	126413513	Tcfap2e	0	-	MACS_peak_11210	16972
chr4	126539064	126645167	Zmym4	0	-	MACS_peak_11216	-11
chr4	126724337	126738376	Zmym1	0	-	MACS_peak_11217	27293
chr4	126754626	126801538	Zmym6	0	+	MACS_peak_11217|MACS_peak_11218	-11043|-26100
chr4	126803289	126806888	Gm12942	0	+	MACS_peak_11218	22563
chr4	128782859	128788829	Tmem54	0	+	MACS_peak_11226	29396
chr4	128735514	128761769	Rnf19b	0	+	MACS_peak_11226	-17949
chr4	128828068	128866726	S100pbp	0	-	MACS_peak_11227|MACS_peak_11228|M	-5835|512|7400|16684|18504|20227|25219|29396
chr4	128867038	128896851	Yars	0	+	MACS_peak_11227|MACS_peak_11228|M	6147|-200|-7088|-16372|-18192|-19915|-24907|-29084
chr4	128896821	128925687	C77080	0	-	MACS_peak_11234|MACS_peak_11237	-29565|-22517
chr4	129725083	129776520	Col16a1	0	+	MACS_peak_11249|MACS_peak_11251|M	-7324|-11149|-19406
chr4	129784799	129805378	Pef1	0	+	MACS_peak_11264	17040
chr4	132014643	132019836	Med18	0	-	MACS_peak_11277	29041
chr4	132048721	132066371	Sesn2	0	-	MACS_peak_11277|MACS_peak_11278|M	-17494|-15917|-11126|-10592|-196
chr4	132086469	132089574	Atpif1	0	-	MACS_peak_11281	-23399
chr4	132091473	132109657	Dnajc8	0	+	MACS_peak_11281	25298
chr4	132765254	132768451	Gpr3	0	-	MACS_peak_11288	24746
chr4	132796732	132808843	Map3k6	0	+	MACS_peak_11288|MACS_peak_11290|M	3535|-2069|-2587|-4254|-5015|-5510|-5890|-6113|-6388|-6634|-6829|-7259|-7638|-8027|-8919|-9479|-10196|-10874|-11094|-11620
chr4	132776723	132780469	Cd164l2	0	+	MACS_peak_11288|MACS_peak_11290|M	-16474|-22078|-22596|-24263|-25024|-25519|-25899|-26122|-26397|-26643|-26838|-27268|-27647|-28036|-28928|-29488
chr4	132809004	132819002	Sytl1	0	-	MACS_peak_11288|MACS_peak_11290|M	-25805|-20201|-19683|-18016|-17255|-16760|-16380|-16157|-15882|-15636|-15441|-15011|-14632|-14243|-13351|-12791|-12074|-11396|-
chr4	132821959	132833705	Tmem222	0	-	MACS_peak_11302|MACS_peak_11303|M	-29714|-29335|-28946|-28054|-27494|-26777|-26099|-25879|-25353|18722|19744|22854
chr4	132848380	132895230	Wdtc1	0	-	MACS_peak_11321	-185
chr4	133162906	133189344	Zdhhc18	0	-	MACS_peak_11334	26825
chr4	133217340	133228562	Pigv	0	-	MACS_peak_11334|MACS_peak_11334|M	-12393|-12393|-12393
chr4	133898984	133908580	Slc30a2	0	+	MACS_peak_11337	-14618
chr4	133912287	133928462	Extl1	0	+	MACS_peak_11337	-14860
chr4	134066913	134079837	2610002D1	0	+	MACS_peak_11339|MACS_peak_11340	-18251|-29692
chr4	134093806	134108081	Sepn1	0	-	MACS_peak_11339|MACS_peak_11340|M	-22917|-11476|26167
chr4	134081477	134088667	Fam54b	0	-	MACS_peak_11339|MACS_peak_11340	-3503|7938
chr4	134301326	134323919	Ldlrap1	0	-	MACS_peak_11347|MACS_peak_11348|M	-29824|-29334|-73
chr4	134876398	134909129	Srrm1	0	-	MACS_peak_11364|MACS_peak_11364	-8157|-8157
chr4	134968223	134989720	Rcan3	0	-	MACS_peak_11368|MACS_peak_11369|M	18137|18390|18664|22239
chr4	135004815	135050419	Nipal3	0	-	MACS_peak_11372	-14904
chr4	135051901	135093718	4930555I2	0	+	MACS_peak_11372	16386
chr4	135803871	135830814	Tcea3	0	+	MACS_peak_11375	8664
chr4	135841983	135849857	Zfp46	0	+	MACS_peak_11377	-243
chr4	135866890	135896593	Hnrnpr	0	+	MACS_peak_11377	24664
chr4	137024717	137126545	Hspg2	0	+	MACS_peak_11385	-58
chr4	137009210	137011376	1700013G1	0	+	MACS_peak_11385	-15565
chr4	137126790	137131095	Ldlrad2	0	-	MACS_peak_11440|MACS_peak_11442|M	-29843|-28527|-25122|-24768|-24306|-23323|-22303|-21454|-19993|-18496|-16316|-14717|-12757|-6836|-5866
chr4	137150103	137214452	Usp48	0	+	MACS_peak_11472|MACS_peak_11475	25844|24874
chr4	137418151	137521143	Ece1	0	+	MACS_peak_11484	-76
chr4	137806325	137816883	Sh2d5	0	+	MACS_peak_11507|MACS_peak_11508	-6397|-7249
chr4	137818165	137857885	Kif17	0	+	MACS_peak_11507|MACS_peak_11507|M	5443|5443|4591|4591
chr4	138011062	138016041	Camk2n1	0	+	MACS_peak_11515	14501
chr4	137990586	137998180	Mul1	0	+	MACS_peak_11515	-5975
chr4	138908507	138934645	C230096C1	0	+	MACS_peak_11527|MACS_peak_11527	-9560|-9560
chr4	138936573	139045445	Ubr4	0	+	MACS_peak_11527|MACS_peak_11530|M	18506|-10235|-12428|-19202|-19723|-20713|-29523
chr4	138903359	138908213	Mrto4	0	-	MACS_peak_11527	9854
chr4	140257387	140279135	Rcc2	0	+	MACS_peak_11580|MACS_peak_11581|M	889|-94|-818|-11981|-14479
chr4	140283269	140298558	Padi6	0	-	MACS_peak_11584|MACS_peak_11585	-29190|-26692
chr4	140572552	140616460	Crocc	0	-	MACS_peak_11588|MACS_peak_11588	6535|13419
chr4	140622427	140634260	Necap2	0	-	MACS_peak_11588	-11265
chr4	140644259	140668674	Spata21	0	+	MACS_peak_11588	21264
chr4	140769870	140776029	6330545A(	0	+	MACS_peak_11590	-25826
chr4	140798798	140813477	Arhgef19	0	+	MACS_peak_11590|MACS_peak_11591	3102|-4209
chr4	140857154	140885293	Epha2	0	+	MACS_peak_11596|MACS_peak_11597|M	-104|-5307|-15416|-15798|-17301|-17759|-19283|-20290|-20560|-20853|-21069|-21423|-22254|-23063|-27627
chr4	140924134	140940090	Fam131c	0	+	MACS_peak_11612	-15373
chr4	140940525	140954014	Clcnka	0	-	MACS_peak_11612|MACS_peak_11612	-14507|-15114
chr4	140988817	140992020	Gm694	0	-	MACS_peak_11613	29946
chr4	141000587	141023852	Zbtb17	0	+	MACS_peak_11613	-21379
chr4	141023804	141094512	Spen	0	-	MACS_peak_11615	7664
chr4	141102076	141104228	B330016D1	0	+	MACS_peak_11615	-100
chr4	144802270	144836773	Tnfrsf1b	0	-	MACS_peak_11642	-23333
chr4	146237770	146556724	1700029I0	0	+	MACS_peak_11663	28384
chr4	147234887	147242828	Miip	0	-	MACS_peak_11677	-3492
chr4	147243087	147244467	Fv1	0	+	MACS_peak_11677	3751
chr4	147197094	147222484	2810408P1	0	-	MACS_peak_11677	16852
chr4	147283862	147310885	Plod1	0	-	MACS_peak_11679	-19419
chr4	147315003	147321423	2510039O	0	+	MACS_peak_11679	23537
chr4	147247703	147278928	Mfn2	0	-	MACS_peak_11679	12538
chr4	147374854	147376176	Nppa	0	+	MACS_peak_11683|MACS_peak_11686	14759|-13830

chr4	147359897	147361314	Nppb	0	+	MACS_peak_11683|MACS_peak_11686	-198|-28787
chr4	147415297	147433671	Mthfr	0	+	MACS_peak_11686|MACS_peak_11686|M	26613|24501|26613
chr4	147380592	147412876	Clcn6	0	-	MACS_peak_11686	-24192
chr4	147526908	147533704	Fbxo44	0	-	MACS_peak_11689|MACS_peak_11689|M	-14860|-15329|-15329
chr4	147519824	147526034	Fbxo6	0	-	MACS_peak_11689|MACS_peak_11689|M	-7190|-7190|-7294|-7400|-7190
chr4	147472546	147504807	2610109H	0	-	MACS_peak_11689	14037
chr4	147514598	147519805	Mad2l2	0	+	MACS_peak_11689	-4246
chr4	147534776	147540526	Fbxo2	0	+	MACS_peak_11689	15932
chr4	147822690	147931793	Mtor	0	+	MACS_peak_11690|MACS_peak_11692	-16494|-20453
chr4	147808605	147818860	Ubiad1	0	-	MACS_peak_11690|MACS_peak_11692	20324|24283
chr4	147869388	147874571	Angptl7	0	-	MACS_peak_11693|MACS_peak_11695	-16105|11344
chr4	147932535	147956507	Exosc10	0	+	MACS_peak_11698|MACS_peak_11699	20109|19901
chr4	148524093	148540816	Pgd	0	-	MACS_peak_11702|MACS_peak_11705	-1569|15631
chr4	148502457	148511709	Apitd1	0	-	MACS_peak_11702	27538
chr4	148607842	148681807	Kif1b	0	-	MACS_peak_11712|MACS_peak_11712	-16367|-16367
chr4	148892349	148940546	Ctnnbip1	0	+	MACS_peak_11721|MACS_peak_11724	21728|18544
chr4	148859441	148870776	Lzic	0	+	MACS_peak_11721|MACS_peak_11724	-11180|-14364
chr4	148842895	148859251	Nmnat1	0	-	MACS_peak_11721|MACS_peak_11724	11370|14554
chr4	149089492	149112143	Tmem201	0	-	MACS_peak_11729|MACS_peak_11729|M	6636|6634|15767|15765
chr4	149118144	149148376	Slc25a33	0	-	MACS_peak_11729|MACS_peak_11730|M	-29597|-20466|5118|6359
chr4	149353589	149383132	H6pd	0	-	MACS_peak_11740	-197
chr4	149561124	149575244	Car6	0	-	MACS_peak_11741|MACS_peak_11742|M	-13850|-11958|-8585|-7397|-5026|-3743|-2847
chr4	151382999	151391786	Klhl21	0	+	MACS_peak_11771	-553
chr4	151356747	151363095	Thap3	0	-	MACS_peak_11771|MACS_peak_11771	20457|20457
chr4	151413435	151434142	Nol9	0	+	MACS_peak_11771|MACS_peak_11771	29883|29883
chr4	151402022	151412599	Tas1r1	0	-	MACS_peak_11771	-29047
chr4	151393884	151401780	Zbtb48	0	-	MACS_peak_11771	-18228
chr4	151363739	151370288	Phf13	0	-	MACS_peak_11771	13264
chr4	151660080	151665771	Hes3	0	-	MACS_peak_11777|MACS_peak_11778	-12295|7068
chr4	151671459	151681231	Icmt	0	+	MACS_peak_11777|MACS_peak_11778	17983|-1380
chr4	151648470	151659446	Gpr153	0	+	MACS_peak_11777|MACS_peak_11778	-5006|-24369
chr4	151681131	151692734	Rnf207	0	-	MACS_peak_11778	-19895
chr4	151699986	151708180	Rpl22	0	+	MACS_peak_11778	27147
chr4	153544821	153649830	Megf6	0	+	MACS_peak_11780	12685
chr4	153430357	153514317	Trp73	0	-	MACS_peak_11780	17819
chr4	153516480	153530927	Wdr8	0	+	MACS_peak_11780	-15656
chr4	153531594	153534793	Tprgl	0	-	MACS_peak_11780	-2657
chr4	154441138	154446515	Pex10	0	+	MACS_peak_11781|MACS_peak_11782	-15678|-16183
chr4	154460685	154519616	Morn1	0	+	MACS_peak_11781|MACS_peak_11782	3869|3364
chr4	154448220	154460406	Rer1	0	-	MACS_peak_11781|MACS_peak_11782	-3590|-3085
chr4	154998977	155024041	Cdk11b	0	+	MACS_peak_11783	23239
chr4	154975524	154997450	Slc35e2	0	+	MACS_peak_11783	-214
chr4	155024763	155027493	Mmp23	0	-	MACS_peak_11785|MACS_peak_11788|M	1753|2777|3344|3857|6101|6353|7559|7860
chr4	155028580	155043336	Mib2	0	-	MACS_peak_11785|MACS_peak_11788|M	-14090|-13066|-12499|-11986|-9742|-9490|-8284|-7983
chr4	32744093	32862192	Mdn1	0	+	MACS_peak_10753|MACS_peak_10754	22288|-15794
chr4	32702447	32740240	Casp8ap2	0	+	MACS_peak_10753|MACS_peak_10753	-19358|-19358
chr4	34514029	34562191	Orc3l	0	-	MACS_peak_10762|MACS_peak_10762	15575|15575
chr4	34562206	34607414	Rars2	0	+	MACS_peak_10762	-15560
chr4	3476187	3501915	Tmem68	0	-	MACS_peak_10702|MACS_peak_10703	-24509|-14172
chr4	3502025	3543768	Tgs1	0	+	MACS_peak_10702|MACS_peak_10703	24619|14282
chr4	35138530	35173129	3110043O	0	-	MACS_peak_10766|MACS_peak_10767	-12280|-7214
chr4	40751634	40801031	B4galt1	0	-	MACS_peak_10776	-391
chr4	41039756	41045216	Aqp3	0	-	MACS_peak_10777|MACS_peak_10778	16792|26044
chr4	41083053	41140403	Ube2r2	0	+	MACS_peak_10777|MACS_peak_10778	21045|11793
chr4	41061459	41071372	Nol6	0	-	MACS_peak_10777|MACS_peak_10778	-9364|-112
chr4	41670867	41678174	Arid3c	0	-	MACS_peak_10783	-10315
chr4	41604529	41642477	Cntfr	0	-	MACS_peak_10783|MACS_peak_10783|M	25382|23736|23736
chr4	41661829	41670195	Dctn3	0	-	MACS_peak_10783|MACS_peak_10783	-2336|-2336
chr4	41685368	41688186	Sigmar1	0	-	MACS_peak_10783	-20327
chr4	41659064	41660549	2810432D	0	-	MACS_peak_10783	7310
chr4	43071855	43277745	Unc13b	0	+	MACS_peak_10785|MACS_peak_10785|M	25076|25165|21853|21942
chr4	43015210	43023173	Fancg	0	-	MACS_peak_10785|MACS_peak_10785|M	23606|23606|26829|26829
chr4	43030509	43038628	Pigo	0	-	MACS_peak_10785|MACS_peak_10787	8151|11374
chr4	43040561	43044256	Stoml2	0	-	MACS_peak_10785|MACS_peak_10787	2523|5746
chr4	43045381	43059075	B230312A	0	-	MACS_peak_10785|MACS_peak_10787	-12296|-9073
chr4	43394853	43439958	Rusc2	0	+	MACS_peak_10788|MACS_peak_10788|M	-150|24442|-2308|22284
chr4	43460595	43467498	Cd72	0	-	MACS_peak_10800|MACS_peak_10800|M	-8850|-8850|-8850|-8850
chr4	43439893	43442006	Fam166b	0	-	MACS_peak_10800|MACS_peak_10800	16642|16642
chr4	43455148	43460946	Tesk1	0	+	MACS_peak_10800	-3500
chr4	43527583	43536260	Tpm2	0	-	MACS_peak_10803	29674
chr4	43544384	43575455	Tln1	0	-	MACS_peak_10803|MACS_peak_10806	-9521|2919
chr4	43575505	43579932	Creb3	0	+	MACS_peak_10803|MACS_peak_10806	9571|-2869
chr4	43579801	43591736	Gba2	0	-	MACS_peak_10803|MACS_peak_10806	-25802|-13362
chr4	43591606	43600359	Rgp1	0	+	MACS_peak_10803|MACS_peak_10806	25672|13232
chr4	43596087	43597366	Msmp	0	-	MACS_peak_10806	-18992
chr4	43664600	43666424	Spag8	0	-	MACS_peak_10812|MACS_peak_10813|M	-6938|-6561|-2551
chr4	43681842	43705539	Tmem8b	0	+	MACS_peak_10812|MACS_peak_10813|M	22356|21979|17969|-22133
chr4	43667098	43669317	Hint2	0	-	MACS_peak_10812|MACS_peak_10813|M	-9831|-9454|-5444
chr4	43644806	43664112	Npr2	0	+	MACS_peak_10812|MACS_peak_10813|M	-14680|-15057|-19067
chr4	43672493	43681731	4930412F1	0	-	MACS_peak_10812|MACS_peak_10813|M	-22245|-21868|-17858|22244
chr4	43707871	43713679	Olfr70	0	-	MACS_peak_10817	-9704
chr4	43718499	43719438	Olfr71	0	-	MACS_peak_10817	-15463
chr4	43996375	43998405	Ccin	0	+	MACS_peak_10818	12966
chr4	43970573	43991990	Glipr2	0	+	MACS_peak_10818	-12836
chr4	44025514	44045718	Clta	0	+	MACS_peak_10821|MACS_peak_10821|M	-26360|-26360|-26360|-26360
chr4	44049694	44085545	Gne	0	-	MACS_peak_10823|MACS_peak_10823	-17391|-28895
chr4	44139083	44180410	Rnf38	0	-	MACS_peak_10824	-29926
chr4	45408795	45421638	Mcart1	0	-	MACS_peak_10828	14940
chr4	45436148	45543700	Shb	0	-	MACS_peak_10829	-528
chr4	46000333	46047631	Tdrd7	0	+	MACS_peak_10831	-4768
chr4	46549808	46614801	Coro2a	0	-	MACS_peak_10838|MACS_peak_10839|M	18586|26509|28045
chr4	46617261	46663071	Tbc1d2	0	-	MACS_peak_10838|MACS_peak_10839|M	-29684|-21761|-20225|-368|-113
chr4	48676385	48686364	Murc	0	+	MACS_peak_10848|MACS_peak_10850|M	26601|13159|4663|953
chr4	49644931	49669758	Rnf20	0	+	MACS_peak_10854|MACS_peak_10854|M	-6658|-6658|-18085|-18085|-24281|-24281
chr4	49691618	49694855	Ppp3r2	0	-	MACS_peak_10857	-25643
chr4	53024795	53034931	Nipsnap3b	0	+	MACS_peak_10863|MACS_peak_10864|M	-20608|-21275|-21824
chr4	53792576	53799757	Tal2	0	+	MACS_peak_10878|MACS_peak_10879|M	18231|17259|16319|14952
chr4	56815200	56822477	BC026590	0	+	MACS_peak_10900	29262
chr4	56756293	56757797	Actl7a	0	+	MACS_peak_10900	-29645
chr4	56762551	56815203	Ikbkap	0	-	MACS_peak_10900	-29265
chr4	56823808	56878083	Ctnnal1	0	-	MACS_peak_10901|MACS_peak_10902	9878|14795
chr4	56908100	56908170	Mir32	0	-	MACS_peak_10901|MACS_peak_10902|M	-20209|-15292|3799|4063
chr4	56888884	56960301	D730040F	0	-	MACS_peak_10905	-478
chr4	57867031	57909856	Akap2	0	+	MACS_peak_10908|MACS_peak_10908|M	8898|8898|8898
chr4	59918770	59927928	Slc46a2	0	-	MACS_peak_10920|MACS_peak_10921|M	-29956|-28554|-22550
chr4	61947478	61959445	Slc31a2	0	+	MACS_peak_10928	-21546
chr4	62069816	62088024	Prpf4	0	+	MACS_peak_10930	-5802
chr4	62055622	62069657	Cdc26	0	-	MACS_peak_10930	5961
chr4	62088575	62095760	Rnf183	0	-	MACS_peak_10930	-20142
chr4	63005599	63009196	Orm1	0	+	MACS_peak_10931|MACS_peak_10932	-23031|-23407
chr4	63023482	63026911	Orm2	0	+	MACS_peak_10931|MACS_peak_10932|M	-5148|-5524|-19358
chr4	63017195	63020545	Orm3	0	+	MACS_peak_10931|MACS_peak_10932|M	-11435|-11811|-25645
chr4	63028161	63064479	Akna	0	-	MACS_peak_10933|MACS_peak_10934	-21639|-8045
chr4	63075943	63156985	Whrn	0	-	MACS_peak_10935|MACS_peak_10935|M	-510|-510|-510|-510|-510|-510|-510
chr4	65266019	65277274	Trim32	0	+	MACS_peak_10940|MACS_peak_10940	-9696|-9696
chr4	66488844	66503830	Tlr4	0	+	MACS_peak_10942	-14666
chr4	71778175	71861953	Tle1	0	-	MACS_peak_10950	-475
chr4	71862277	71864964	C630043F	0	+	MACS_peak_10950	799
chr4	73888400	74051768	Kdm4c	0	+	MACS_peak_10952|MACS_peak_10952	14192|23574
chr4	83918445	84320990	Bnc2	0	-	MACS_peak_10969|MACS_peak_10970	23736|24158
chr4	86394458	86476071	Dennd4c	0	+	MACS_peak_10977|MACS_peak_10977	-29286|-29286
chr4	86500002	86503271	Rps6	0	-	MACS_peak_10986|MACS_peak_10987	-27792|-20880

chr4	88783273	88826994	Mtap	0	+	MACS_peak_10993|MACS_peak_10994	14214|13910
chr4	94608731	94645791	Mysm1	0	-	MACS_peak_10998	-8401
chr4	99602055	99659899	Pgm2	0	+	MACS_peak_11014	-1331
chr5	100790668	100845253	Sec31a	0	-	MACS_peak_12333|MACS_peak_12336|M	-20419|4633|10572|13181
chr5	100849860	100858554	5430416N(	0	-	MACS_peak_12336|MACS_peak_12337|M	-8668|-2729|-120
chr5	101083744	101103275	Coq2	0	-	MACS_peak_12341	10780
chr5	101108504	101148702	Hpse	0	-	MACS_peak_12343	-200
chr5	101233820	101249954	Fam175a	0	-	MACS_peak_12344	24528
chr5	101275247	101328121	Agpat9	0	+	MACS_peak_12344|MACS_peak_12346	765|-10952
chr5	104631635	104643121	Dmp1	0	+	MACS_peak_12371	-29
chr5	104937370	104951400	BC005561	0	+	MACS_peak_12377	5968
chr5	104954753	104983229	D930016D	0	+	MACS_peak_12377	23351
chr5	108259271	108260783	1700013N	0	+	MACS_peak_12399	13417
chr5	108173813	108304126	Evi5	0	-	MACS_peak_12400	28648
chr5	108329608	108337069	Rpl5	0	+	MACS_peak_12400	-3166
chr5	108561932	108661968	Ccdc18	0	+	MACS_peak_12402|MACS_peak_12403|M	10102|8492|6926|500|-12544
chr5	108550665	108561610	Tmed5	0	-	MACS_peak_12402|MACS_peak_12403|M	-9780|-8170|-6604|-178|12866
chr5	108741943	108777798	Pigg	0	+	MACS_peak_12407|MACS_peak_12408	-19134|-19990
chr5	108947572	108979046	Cplx1	0	-	MACS_peak_12410	19672
chr5	109058828	109076789	Tmem175	0	+	MACS_peak_12411|MACS_peak_12411|M	4749|4749|4749|-13324|-13324|-13324
chr5	108998432	109058758	Gak	0	-	MACS_peak_12411|MACS_peak_12412	-4679|13394
chr5	109098124	109113577	Idua	0	+	MACS_peak_12412|MACS_peak_12413|M	25972|-12650|-14655
chr5	109076062	109089788	Dgkq	0	-	MACS_peak_12412|MACS_peak_12413|M	-17636|20986|22991
chr5	109123247	109135968	Fgfrl1	0	+	MACS_peak_12413|MACS_peak_12413|M	12473|12473|10468|10468|-11168|-11168
chr5	109099565	109104668	Slc26a1	0	-	MACS_peak_12413|MACS_peak_12414|M	6106|8111|29747
chr5	109424557	109435455	Vmn2r10	0	-	MACS_peak_12422	9635
chr5	109983729	109988012	Crlf2	0	-	MACS_peak_12424|MACS_peak_12424|M	-2893|-2893|-1398|-1398
chr5	110539110	110558813	Zfp605	0	+	MACS_peak_12429|MACS_peak_12430	5093|3349
chr5	110532995	110537216	Gtpbp6	0	-	MACS_peak_12429|MACS_peak_12430	-3199|-1455
chr5	110528987	110534969	Plcxd1	0	+	MACS_peak_12429|MACS_peak_12429|M	-5030|-4440|-6774|-6184
chr5	110564858	110600991	Chfr	0	+	MACS_peak_12430	29097
chr5	110605719	110652174	Golga3	0	+	MACS_peak_12431|MACS_peak_12432|M	-7770|-10963|-16305
chr5	110660059	110685667	Ankle2	0	+	MACS_peak_12436|MACS_peak_12438|M	12888|-19714|-21985|-22806
chr5	110688153	110698918	Pgam5	0	-	MACS_peak_12438|MACS_peak_12438|M	-19145|-19145|-16874|-16874|-16053|-16053
chr5	110783114	110785106	Gm1679	0	+	MACS_peak_12442	-24058
chr5	110790770	110816146	Fbrsl1	0	-	MACS_peak_12442	-8974
chr5	111202686	111209615	Pus1	0	-	MACS_peak_12445|MACS_peak_12445|M	6568|6549|6568|7015|6996|7015
chr5	111213507	111239100	Ulk1	0	-	MACS_peak_12445|MACS_peak_12446|M	-22917|-22470|29490
chr5	111093391	111199736	Ep400	0	-	MACS_peak_12445|MACS_peak_12445|M	16447|16447|16894|16894
chr5	111269035	111303152	Chek2	0	+	MACS_peak_12449	445
chr5	111258088	111268796	Hscb	0	-	MACS_peak_12449	-206
chr5	112782820	112785053	Gm6583	0	-	MACS_peak_12451|MACS_peak_12452|M	-22809|-11489|-6682
chr5	112755388	112767093	Tfip11	0	+	MACS_peak_12451|MACS_peak_12452|M	-6856|-18176|-22983
chr5	112766410	112772060	Srrd	0	-	MACS_peak_12451|MACS_peak_12452|M	-9816|1504|6311
chr5	112772114	112807443	Hps4	0	+	MACS_peak_12451|MACS_peak_12452|M	9870|-1450|-6257
chr5	113504859	113510604	Crybb3	0	-	MACS_peak_12457|MACS_peak_12457	19921|19921
chr5	113515344	113592333	2900026A(	0	-	MACS_peak_12458|MACS_peak_12460|M	18980|21069|27848
chr5	114550341	114572933	Usp30	0	+	MACS_peak_12465	-2114
chr5	114580443	114589330	Ung	0	+	MACS_peak_12465|MACS_peak_12465	27988|28709
chr5	114476921	114541389	Svop	0	-	MACS_peak_12465	11066
chr5	114573942	114578185	Alkbh2	0	-	MACS_peak_12465	-25730
chr5	115072163	115108430	Trpv4	0	-	MACS_peak_12469	-27577
chr5	115560307	115569322	Acads	0	-	MACS_peak_12485	-7944
chr5	115572574	115584984	Unc119b	0	-	MACS_peak_12485	-23606
chr5	116009475	116013736	Rplp0	0	+	MACS_peak_12490	-22885
chr5	116015271	116072663	Gcn1l1	0	+	MACS_peak_12490|MACS_peak_12491	-17089|-25069
chr5	116081995	116097167	Rab35	0	+	MACS_peak_12494|MACS_peak_12495|M	25403|22199|17844|16506|9659
chr5	116081057	116081825	1110006O:	0	-	MACS_peak_12494|MACS_peak_12495|M	-25233|-22029|-17674|-16336|-9489
chr5	116858503	116872873	Hspb8	0	-	MACS_peak_12501|MACS_peak_12502|M	16812|17653|18977
chr5	116888730	116915496	2410137F1	0	+	MACS_peak_12501|MACS_peak_12502|M	-955|-1796|-3120
chr5	118426778	118460200	Fbxo21	0	+	MACS_peak_12511	-3020
chr5	118695235	118713123	2410131K:	0	+	MACS_peak_12513|MACS_peak_12515	-18430|-19491
chr5	118640744	118695034	Rnft2	0	-	MACS_peak_12513|MACS_peak_12513|M	18631|18795|19692|19856
chr5	120120677	120134610	Tbx3	0	+	MACS_peak_12522|MACS_peak_12522|M	-6869|-6869|-9924|-9924
chr5	121063138	121078601	Ddx54	0	+	MACS_peak_12535|MACS_peak_12536	635|-59
chr5	121059071	121062578	1110008J(	0	-	MACS_peak_12535|MACS_peak_12536	-75|619
chr5	121039031	121057122	Iqcd	0	+	MACS_peak_12535|MACS_peak_12536	-23472|-24166
chr5	120984165	121038622	Tpcn1	0	-	MACS_peak_12535|MACS_peak_12536	23881|24575
chr5	121847990	121890122	Naa25	0	+	MACS_peak_12543	-23367
chr5	122161617	122264959	Atxn2	0	+	MACS_peak_12557	27192
chr5	122071037	122110519	Acad10	0	-	MACS_peak_12557	23906
chr5	122110594	122137256	Brap	0	+	MACS_peak_12557	-23831
chr5	122906341	122952234	Atp2a2	0	-	MACS_peak_12564|MACS_peak_12564|M	-12919|-12919|-12919|-471|-471|-471
chr5	123093919	123134293	P2rx7	0	+	MACS_peak_12567|MACS_peak_12567|M	-29475|-29475|-29475|-29475|-29828|-29828|-29828|-29828
chr5	123978770	124023024	Vps33a	0	-	MACS_peak_12572	5639
chr5	124029078	124134300	Clip1	0	-	MACS_peak_12582	25156
chr5	124148310	124171053	Zcchc8	0	-	MACS_peak_12582	-11597
chr5	124818710	124875923	Sbno1	0	-	MACS_peak_12584	-25129
chr5	125921937	125941255	Bri3bp	0	+	MACS_peak_12589|MACS_peak_12590|M	-168|-13207|-14603|-17014|-18155
chr5	125894773	125914418	Dhx37	0	-	MACS_peak_12589|MACS_peak_12590|M	7687|20726|22122|24533|25674
chr5	125956242	125997773	Aacs	0	+	MACS_peak_12590|MACS_peak_12591|M	21098|19702|17291|16150
chr5	128076035	128097762	Slc15a4	0	-	MACS_peak_12594	-8470
chr5	128112631	128187890	Glt1d1	0	+	MACS_peak_12594	23339
chr5	130293260	130322231	Cct6a	0	+	MACS_peak_12597|MACS_peak_12598|M	21659|20894|16715|8287
chr5	130271433	130293129	Psph	0	-	MACS_peak_12597|MACS_peak_12598|M	-21528|-20763|-16584|-8156
chr5	130300436	130300552	Snora15	0	+	MACS_peak_12597|MACS_peak_12598|M	28835|28070|23891|15463
chr5	130695613	130719635	0610007L(	0	+	MACS_peak_12601	10992
chr5	130663049	130690207	Rabgef1	0	+	MACS_peak_12601	-21572
chr5	130732906	130817437	Tyw1	0	+	MACS_peak_12604|MACS_peak_12604|M	6123|4705|1716|298|-9997|-11415
chr5	130721601	130731332	Sbds	0	-	MACS_peak_12604|MACS_peak_12605|M	-4549|-142|11571
chr5	134623927	134652637	Wbscr16	0	-	MACS_peak_12613	19743
chr5	134659907	134694013	Gtf2ird2	0	+	MACS_peak_12613	-12473
chr5	134696129	134705495	Ncf1	0	-	MACS_peak_12615	22278
chr5	135852009	135870416	Pom121	0	-	MACS_peak_12622	14360
chr5	136363788	136365433	Hspb1	0	+	MACS_peak_12645	-22119
chr5	136384248	136410511	Ywhag	0	-	MACS_peak_12645	-24604
chr5	136436093	136450346	Srcrb4d	0	-	MACS_peak_12647	21900
chr5	136455974	136464494	Zp3	0	+	MACS_peak_12647	-16272
chr5	136470746	136508734	Dtx2	0	+	MACS_peak_12647|MACS_peak_12650	-1500|-26903
chr5	136514365	136520863	Upk3b	0	+	MACS_peak_12650|MACS_peak_12651|M	16716|11960|9353
chr5	136533136	136540193	2310043J(	0	+	MACS_peak_12653	28124
chr5	136559785	136587730	Rasa4	0	+	MACS_peak_12657|MACS_peak_12657|M	-20666|-20666|-23248|-23248
chr5	136592560	136598817	Polr2j	0	+	MACS_peak_12657|MACS_peak_12658	12109|9527
chr5	136598935	136611944	Lrwd1	0	-	MACS_peak_12658	-28911
chr5	137791336	137815750	Ephb4	0	+	MACS_peak_12677|MACS_peak_12677|M	-10332|-10332|-11193|-11193|-13183|-13183|-22019|-22019
chr5	137755786	137774810	Slc12a9	0	-	MACS_peak_12677|MACS_peak_12678|M	26858|27719|29709
chr5	138187223	138200975	Tsc22d4	0	+	MACS_peak_12688|MACS_peak_12689|M	14830|14245|10403
chr5	138172190	138181226	6430598A(	0	-	MACS_peak_12688|MACS_peak_12689|M	-8833|-8248|-4406
chr5	138526311	138549050	Zkscan1	0	+	MACS_peak_12693|MACS_peak_12693	-84|-84
chr5	138605816	138613090	Mcm7	0	-	MACS_peak_12699	2941
chr5	138619844	138628414	Taf6	0	-	MACS_peak_12699	-12383
chr5	138602329	138605212	Cops6	0	+	MACS_peak_12699	-13702
chr5	138580929	138596972	Zfp113	0	-	MACS_peak_12699	19059
chr5	138613248	138619913	Ap4m1	0	+	MACS_peak_12699	-2783
chr5	138635541	138636628	Mblac1	0	+	MACS_peak_12699	19510
chr5	138628762	138635122	Cnpy4	0	+	MACS_peak_12699	12731
chr5	138606964	138607046	Mir106b	0	-	MACS_peak_12699	8985
chr5	138606751	138606838	Mir93	0	-	MACS_peak_12699	9193
chr5	138606548	138606632	Mir25	0	-	MACS_peak_12699	9399
chr5	139231034	139286017	Fam20c	0	+	MACS_peak_12703|MACS_peak_12704	-143|-2833
chr5	139452928	139470907	Pdgfa	0	-	MACS_peak_12708|MACS_peak_12710|M	-18298|-8639|-94
chr5	139676623	139725749	Sun1	0	+	MACS_peak_12716|MACS_peak_12718|M	-14536|-26128|-29146

<table>
<tr><td>chr5</td><td>139729450</td><td>139746004</td><td>Get4</td><td>0</td><td>+</td><td>MACS_peak_12718|MACS_peak_12718|N</td><td>26699|25526|23681|22508|18738|17565|17887|16714|12145|10972|5380|4207</td></tr>
<tr><td>chr5</td><td>139865857</td><td>139872368</td><td>Gpr146</td><td>0</td><td>+</td><td>MACS_peak_12727|MACS_peak_12727</td><td>-2822|-12059</td></tr>
<tr><td>chr5</td><td>139882340</td><td>139892046</td><td>C130050O</td><td>0</td><td>+</td><td>MACS_peak_12727</td><td>13661</td></tr>
<tr><td>chr5</td><td>139845603</td><td>139845699</td><td>Mir339</td><td>0</td><td>-</td><td>MACS_peak_12727</td><td>22980</td></tr>
<tr><td>chr5</td><td>139853650</td><td>139856007</td><td>D830046C</td><td>0</td><td>+</td><td>MACS_peak_12727</td><td>-15029</td></tr>
<tr><td>chr5</td><td>139947170</td><td>139960445</td><td>Zfand2a</td><td>0</td><td>-</td><td>MACS_peak_12732|MACS_peak_12732|N</td><td>-9561|-9561|-1739|-1739</td></tr>
<tr><td>chr5</td><td>139835692</td><td>139936488</td><td>3110082I1</td><td>0</td><td>-</td><td>MACS_peak_12732|MACS_peak_12733</td><td>14396|22218</td></tr>
<tr><td>chr5</td><td>140182646</td><td>140212287</td><td>Micall2</td><td>0</td><td>-</td><td>MACS_peak_12735|MACS_peak_12736</td><td>-24700|27747</td></tr>
<tr><td>chr5</td><td>140267489</td><td>140278606</td><td>Mafk</td><td>0</td><td>+</td><td>MACS_peak_12736|MACS_peak_12737|N</td><td>27455|22186|-104</td></tr>
<tr><td>chr5</td><td>140227235</td><td>140251632</td><td>Ints1</td><td>0</td><td>-</td><td>MACS_peak_12736|MACS_peak_12737|N</td><td>-11598|-6329|15961</td></tr>
<tr><td>chr5</td><td>140280906</td><td>140290237</td><td>Tmem184a</td><td>0</td><td>-</td><td>MACS_peak_12738|MACS_peak_12738</td><td>-22644|-21604</td></tr>
<tr><td>chr5</td><td>143039434</td><td>143084706</td><td>Mmd2</td><td>0</td><td>-</td><td>MACS_peak_12749</td><td>29490</td></tr>
<tr><td>chr5</td><td>143105537</td><td>143145324</td><td>Wipi2</td><td>0</td><td>+</td><td>MACS_peak_12749</td><td>-8659</td></tr>
<tr><td>chr5</td><td>144011373</td><td>144031701</td><td>Zfp316</td><td>0</td><td>-</td><td>MACS_peak_12756|MACS_peak_12758</td><td>-29640|-17565</td></tr>
<tr><td>chr5</td><td>143977994</td><td>143987561</td><td>Spdye4</td><td>0</td><td>-</td><td>MACS_peak_12756|MACS_peak_12758</td><td>14500|26575</td></tr>
<tr><td>chr5</td><td>143996886</td><td>144009649</td><td>Zfp12</td><td>0</td><td>+</td><td>MACS_peak_12756|MACS_peak_12758</td><td>-5175|-17250</td></tr>
<tr><td>chr5</td><td>144225360</td><td>144265310</td><td>Daglb</td><td>0</td><td>+</td><td>MACS_peak_12760</td><td>-13974</td></tr>
<tr><td>chr5</td><td>144951154</td><td>144955310</td><td>Bhlha15</td><td>0</td><td>+</td><td>MACS_peak_12762|MACS_peak_12765|N</td><td>3305|-6119|-7663|-8333|-10563|-14400|-16207|-18379|-26676|-27033</td></tr>
<tr><td>chr5</td><td>144956217</td><td>144984447</td><td>Tecpr1</td><td>0</td><td>-</td><td>MACS_peak_12765|MACS_peak_12766|N</td><td>-27174|-25630|-24960|-22730|-18893|-17086|-14914|-6617|-6260</td></tr>
<tr><td>chr5</td><td>145005305</td><td>145025442</td><td>Bri3</td><td>0</td><td>+</td><td>MACS_peak_12781|MACS_peak_12782</td><td>27475|27118</td></tr>
<tr><td>chr5</td><td>145025393</td><td>145118981</td><td>Baiap2l1</td><td>0</td><td>-</td><td>MACS_peak_12788</td><td>-175</td></tr>
<tr><td>chr5</td><td>145529660</td><td>145620642</td><td>Trrap</td><td>0</td><td>+</td><td>MACS_peak_12791|MACS_peak_12793|N</td><td>-2380|-24312|-28260</td></tr>
<tr><td>chr5</td><td>145496783</td><td>145522447</td><td>Tmem130</td><td>0</td><td>-</td><td>MACS_peak_12791</td><td>9593</td></tr>
<tr><td>chr5</td><td>145992583</td><td>145999191</td><td>Zfp655</td><td>0</td><td>+</td><td>MACS_peak_12805|MACS_peak_12805</td><td>-1837|-1793</td></tr>
<tr><td>chr5</td><td>145965427</td><td>145982612</td><td>Zkscan5</td><td>0</td><td>+</td><td>MACS_peak_12805|MACS_peak_12805</td><td>-28993|-28993</td></tr>
<tr><td>chr5</td><td>146106138</td><td>146151381</td><td>Cyp3a57</td><td>0</td><td>+</td><td>MACS_peak_12807</td><td>9229</td></tr>
<tr><td>chr5</td><td>149683160</td><td>149740223</td><td>Katnal1</td><td>0</td><td>-</td><td>MACS_peak_12825|MACS_peak_12826</td><td>-24000|26467</td></tr>
<tr><td>chr5</td><td>150214380</td><td>150234278</td><td>6330406I1</td><td>0</td><td>+</td><td>MACS_peak_12829</td><td>-18248</td></tr>
<tr><td>chr5</td><td>150242280</td><td>150273194</td><td>4930588N</td><td>0</td><td>+</td><td>MACS_peak_12829</td><td>9652</td></tr>
<tr><td>chr5</td><td>150419419</td><td>150438890</td><td>Hsph1</td><td>0</td><td>-</td><td>MACS_peak_12831|MACS_peak_12832|N</td><td>-19067|-7000|-196</td></tr>
<tr><td>chr5</td><td>151374217</td><td>151397100</td><td>N4bp2l1</td><td>0</td><td>-</td><td>MACS_peak_12838|MACS_peak_12839</td><td>-21597|-17815</td></tr>
<tr><td>chr5</td><td>20264481</td><td>20387942</td><td>Phtf2</td><td>0</td><td>-</td><td>MACS_peak_11952</td><td>4401</td></tr>
<tr><td>chr5</td><td>20388270</td><td>20392688</td><td>Tmem60</td><td>0</td><td>+</td><td>MACS_peak_11952</td><td>-4073</td></tr>
<tr><td>chr5</td><td>21263094</td><td>21290983</td><td>Dnajc2</td><td>0</td><td>-</td><td>MACS_peak_11954|MACS_peak_11955</td><td>-28129|-3801</td></tr>
<tr><td>chr5</td><td>21291100</td><td>21309602</td><td>Psmc2</td><td>0</td><td>+</td><td>MACS_peak_11954|MACS_peak_11955</td><td>28246|3918</td></tr>
<tr><td>chr5</td><td>21242977</td><td>21262970</td><td>Pmpcb</td><td>0</td><td>+</td><td>MACS_peak_11954</td><td>-19877</td></tr>
<tr><td>chr5</td><td>22940246</td><td>23010045</td><td>Mll5</td><td>0</td><td>+</td><td>MACS_peak_11959</td><td>-15806</td></tr>
<tr><td>chr5</td><td>23889998</td><td>23897961</td><td>Atg9b</td><td>0</td><td>-</td><td>MACS_peak_11974|MACS_peak_11975|N</td><td>-7425|-6852|-6618|-5720|-5106|-4681|-4376|-2306</td></tr>
<tr><td>chr5</td><td>23870636</td><td>23890292</td><td>Nos3</td><td>0</td><td>+</td><td>MACS_peak_11974|MACS_peak_11975|N</td><td>-19900|-20473|-20707|-21605|-22219|-22644|-22949|-25019</td></tr>
<tr><td>chr5</td><td>23899973</td><td>23915765</td><td>Abcb8</td><td>0</td><td>+</td><td>MACS_peak_11974|MACS_peak_11975|N</td><td>9437|8864|8630|7732|7118|6693|6388|4318</td></tr>
<tr><td>chr5</td><td>23919268</td><td>23923652</td><td>Accn3</td><td>0</td><td>+</td><td>MACS_peak_11974|MACS_peak_11975|N</td><td>28732|28159|27925|27027|26413|25988|25683|23613</td></tr>
<tr><td>chr5</td><td>24071158</td><td>24083285</td><td>Abcf2</td><td>0</td><td>-</td><td>MACS_peak_11990|MACS_peak_11990</td><td>14287|14287</td></tr>
<tr><td>chr5</td><td>24098439</td><td>24107820</td><td>Smarcd3</td><td>0</td><td>-</td><td>MACS_peak_11990</td><td>-10248</td></tr>
<tr><td>chr5</td><td>24092567</td><td>24098304</td><td>Chpf2</td><td>0</td><td>+</td><td>MACS_peak_11990</td><td>-5005</td></tr>
<tr><td>chr5</td><td>24097931</td><td>24098028</td><td>Mir671</td><td>0</td><td>+</td><td>MACS_peak_11990</td><td>359</td></tr>
<tr><td>chr5</td><td>28168486</td><td>28181626</td><td>Htr5a</td><td>0</td><td>+</td><td>MACS_peak_12002</td><td>23398</td></tr>
<tr><td>chr5</td><td>28067204</td><td>28117879</td><td>Paxip1</td><td>0</td><td>-</td><td>MACS_peak_12002</td><td>27209</td></tr>
<tr><td>chr5</td><td>30607913</td><td>30668993</td><td>Gm1060</td><td>0</td><td>+</td><td>MACS_peak_12009</td><td>10769</td></tr>
<tr><td>chr5</td><td>30969274</td><td>30977199</td><td>Cenpa</td><td>0</td><td>+</td><td>MACS_peak_12010</td><td>10910</td></tr>
<tr><td>chr5</td><td>30950311</td><td>30962102</td><td>4930471M</td><td>0</td><td>+</td><td>MACS_peak_12010</td><td>-8053</td></tr>
<tr><td>chr5</td><td>31191378</td><td>31209039</td><td>Agbl5</td><td>0</td><td>+</td><td>MACS_peak_12012|MACS_peak_12012|N</td><td>16894|16740|14411|14257</td></tr>
<tr><td>chr5</td><td>31172019</td><td>31179840</td><td>Tmem214</td><td>0</td><td>+</td><td>MACS_peak_12012|MACS_peak_12013</td><td>-2465|-4948</td></tr>
<tr><td>chr5</td><td>31235515</td><td>31247800</td><td>Cgref1</td><td>0</td><td>-</td><td>MACS_peak_12019|MACS_peak_12019</td><td>14713|14660</td></tr>
<tr><td>chr5</td><td>31254039</td><td>31262734</td><td>Preb</td><td>0</td><td>-</td><td>MACS_peak_12019</td><td>-221</td></tr>
<tr><td>chr5</td><td>31252478</td><td>31257464</td><td>Abhd1</td><td>0</td><td>+</td><td>MACS_peak_12019|MACS_peak_12019</td><td>-10035|-10075</td></tr>
<tr><td>chr5</td><td>31271049</td><td>31279391</td><td>Tcf23</td><td>0</td><td>+</td><td>MACS_peak_12019</td><td>8536</td></tr>
<tr><td>chr5</td><td>31338408</td><td>31350935</td><td>Slc5a6</td><td>0</td><td>-</td><td>MACS_peak_12020|MACS_peak_12020|N</td><td>-4012|-4374|-3529|12671|12309|13154</td></tr>
<tr><td>chr5</td><td>31357183</td><td>31380852</td><td>Cad</td><td>0</td><td>+</td><td>MACS_peak_12020|MACS_peak_12022</td><td>10260|-6423</td></tr>
<tr><td>chr5</td><td>31351012</td><td>31356994</td><td>0610007C</td><td>0</td><td>+</td><td>MACS_peak_12020|MACS_peak_12022</td><td>4089|-12594</td></tr>
<tr><td>chr5</td><td>31554078</td><td>31555570</td><td>Krtcap3</td><td>0</td><td>+</td><td>MACS_peak_12023|MACS_peak_12024|N</td><td>-3609|-5227|-14968</td></tr>
<tr><td>chr5</td><td>31543290</td><td>31553935</td><td>Nrbp1</td><td>0</td><td>+</td><td>MACS_peak_12023|MACS_peak_12024|N</td><td>-14397|-16015|-25756</td></tr>
<tr><td>chr5</td><td>31594618</td><td>31598250</td><td>Fndc4</td><td>0</td><td>-</td><td>MACS_peak_12025|MACS_peak_12027|N</td><td>-29204|-8912|-809</td></tr>
<tr><td>chr5</td><td>31555652</td><td>31593487</td><td>Ift172</td><td>0</td><td>-</td><td>MACS_peak_12025|MACS_peak_12027|N</td><td>-24441|-4149|3954</td></tr>
<tr><td>chr5</td><td>31599953</td><td>31629675</td><td>Gckr</td><td>0</td><td>+</td><td>MACS_peak_12027|MACS_peak_12028</td><td>10615|2512</td></tr>
<tr><td>chr5</td><td>31829367</td><td>31856411</td><td>Slc4a1ap</td><td>0</td><td>+</td><td>MACS_peak_12029|MACS_peak_12030</td><td>-13469|-23758</td></tr>
<tr><td>chr5</td><td>31816942</td><td>31829135</td><td>Supt7l</td><td>0</td><td>-</td><td>MACS_peak_12029|MACS_peak_12030</td><td>13701|23990</td></tr>
<tr><td>chr5</td><td>33131855</td><td>33196879</td><td>C330019G</td><td>0</td><td>-</td><td>MACS_peak_12040</td><td>-24077</td></tr>
<tr><td>chr5</td><td>33721344</td><td>33762403</td><td>4933407H</td><td>0</td><td>+</td><td>MACS_peak_12045</td><td>-9022</td></tr>
<tr><td>chr5</td><td>34679038</td><td>34696079</td><td>Rnf4</td><td>0</td><td>+</td><td>MACS_peak_12051</td><td>-15933</td></tr>
<tr><td>chr5</td><td>34916362</td><td>34974954</td><td>Add1</td><td>0</td><td>+</td><td>MACS_peak_12054|MACS_peak_12054|N</td><td>10778|10778|10778|-27722|-27722|-27722</td></tr>
<tr><td>chr5</td><td>34892336</td><td>34906287</td><td>Sh3bp2</td><td>0</td><td>+</td><td>MACS_peak_12054|MACS_peak_12054|N</td><td>-13248|-19571|-13360</td></tr>
<tr><td>chr5</td><td>35898857</td><td>35917719</td><td>2310079F</td><td>0</td><td>-</td><td>MACS_peak_12060</td><td>17058</td></tr>
<tr><td>chr5</td><td>35925708</td><td>35956450</td><td>Acox3</td><td>0</td><td>+</td><td>MACS_peak_12060</td><td>-9069</td></tr>
<tr><td>chr5</td><td>36833252</td><td>36873272</td><td>Tbc1d14</td><td>0</td><td>-</td><td>MACS_peak_12078</td><td>-22978</td></tr>
<tr><td>chr5</td><td>36816319</td><td>36826934</td><td>Tada2b</td><td>0</td><td>-</td><td>MACS_peak_12078</td><td>23360</td></tr>
<tr><td>chr5</td><td>36827236</td><td>36830820</td><td>Ccdc96</td><td>0</td><td>+</td><td>MACS_peak_12078</td><td>-23058</td></tr>
<tr><td>chr5</td><td>3689999</td><td>3803109</td><td>Ankib1</td><td>0</td><td>-</td><td>MACS_peak_11851</td><td>15495</td></tr>
<tr><td>chr5</td><td>3803164</td><td>3844515</td><td>Krit1</td><td>0</td><td>+</td><td>MACS_peak_11851|MACS_peak_11851|N</td><td>-15440|-15440|-15424</td></tr>
<tr><td>chr5</td><td>3845172</td><td>3866596</td><td>4932412H</td><td>0</td><td>+</td><td>MACS_peak_11851</td><td>26568</td></tr>
<tr><td>chr5</td><td>38430473</td><td>38528057</td><td>Stx18</td><td>0</td><td>+</td><td>MACS_peak_12080</td><td>-157</td></tr>
<tr><td>chr5</td><td>3928185</td><td>4080204</td><td>Akap9</td><td>0</td><td>+</td><td>MACS_peak_11852|MACS_peak_11853|N</td><td>-209|-26693|-29518</td></tr>
<tr><td>chr5</td><td>46110905</td><td>46118817</td><td>Gm3414</td><td>0</td><td>-</td><td>MACS_peak_12095</td><td>8197</td></tr>
<tr><td>chr5</td><td>48374393</td><td>48697017</td><td>Slit2</td><td>0</td><td>+</td><td>MACS_peak_12097|MACS_peak_12098|N</td><td>-227|-684|-997|-1559|-6006</td></tr>
<tr><td>chr5</td><td>48615180</td><td>48615289</td><td>Mir218-1</td><td>0</td><td>+</td><td>MACS_peak_12104|MACS_peak_12108|N</td><td>-810|-20718|-25921</td></tr>
<tr><td>chr5</td><td>52544562</td><td>52581758</td><td>Dhx15</td><td>0</td><td>-</td><td>MACS_peak_12118|MACS_peak_12118</td><td>7881|7881</td></tr>
<tr><td>chr5</td><td>52581919</td><td>52589223</td><td>9230114K</td><td>0</td><td>+</td><td>MACS_peak_12118</td><td>-7720</td></tr>
<tr><td>chr5</td><td>54389761</td><td>54512295</td><td>Stim2</td><td>0</td><td>+</td><td>MACS_peak_12124</td><td>-448</td></tr>
<tr><td>chr5</td><td>5505014</td><td>5514849</td><td>Cldn12</td><td>0</td><td>-</td><td>MACS_peak_11881|MACS_peak_11881|N</td><td>-1654|-1594|-1654|-1781</td></tr>
<tr><td>chr5</td><td>5736321</td><td>5749317</td><td>Steap1</td><td>0</td><td>-</td><td>MACS_peak_11884|MACS_peak_11885|N</td><td>-12815|-9402|-8712</td></tr>
<tr><td>chr5</td><td>65779602</td><td>65782670</td><td>Rpl9</td><td>0</td><td>-</td><td>MACS_peak_12144</td><td>11253</td></tr>
<tr><td>chr5</td><td>65782735</td><td>65800446</td><td>Lias</td><td>0</td><td>+</td><td>MACS_peak_12144</td><td>-11188</td></tr>
<tr><td>chr5</td><td>65928499</td><td>65990228</td><td>Ube2k</td><td>0</td><td>+</td><td>MACS_peak_12146</td><td>-150</td></tr>
<tr><td>chr5</td><td>67651890</td><td>67682700</td><td>Tmem33</td><td>0</td><td>+</td><td>MACS_peak_12161|MACS_peak_12161</td><td>-27896|-27896</td></tr>
<tr><td>chr5</td><td>67698195</td><td>67747384</td><td>Slc30a9</td><td>0</td><td>+</td><td>MACS_peak_12161</td><td>18409</td></tr>
<tr><td>chr5</td><td>69966240</td><td>69983524</td><td>Gnpda2</td><td>0</td><td>-</td><td>MACS_peak_12165</td><td>-12690</td></tr>
<tr><td>chr5</td><td>69948180</td><td>69964869</td><td>Guf1</td><td>0</td><td>+</td><td>MACS_peak_12165</td><td>-22654</td></tr>
<tr><td>chr5</td><td>73039034</td><td>73062317</td><td>Nipal1</td><td>0</td><td>+</td><td>MACS_peak_12168|MACS_peak_12169|N</td><td>-11029|-20164|-21859</td></tr>
<tr><td>chr5</td><td>72994935</td><td>73033991</td><td>Cnga1</td><td>0</td><td>-</td><td>MACS_peak_12168|MACS_peak_12169|N</td><td>16072|25207|26902</td></tr>
<tr><td>chr5</td><td>73684032</td><td>73705316</td><td>Ociad1</td><td>0</td><td>+</td><td>MACS_peak_12172|MACS_peak_12172|N</td><td>-1522|-1522|-1522|-1522</td></tr>
<tr><td>chr5</td><td>74591350</td><td>74595502</td><td>Rasl11b</td><td>0</td><td>+</td><td>MACS_peak_12175</td><td>-11010</td></tr>
<tr><td>chr5</td><td>74600840</td><td>74927774</td><td>Scfd2</td><td>0</td><td>-</td><td>MACS_peak_12177|MACS_peak_12177|N</td><td>10311|10311|15011|15011|15393|15393</td></tr>
<tr><td>chr5</td><td>74931506</td><td>74993149</td><td>Fip1l1</td><td>0</td><td>+</td><td>MACS_peak_12177|MACS_peak_12177|N</td><td>-6579|-6579|-6579|-11279|-11279|-11279|-11661|-11661|-11661</td></tr>
<tr><td>chr5</td><td>75402448</td><td>75440651</td><td>Chic2</td><td>0</td><td>-</td><td>MACS_peak_12186</td><td>-17483</td></tr>
<tr><td>chr5</td><td>75552190</td><td>75594229</td><td>Pdgfra</td><td>0</td><td>+</td><td>MACS_peak_12200</td><td>-29400</td></tr>
<tr><td>chr5</td><td>76569301</td><td>76584496</td><td>Srd5a3</td><td>0</td><td>+</td><td>MACS_peak_12206</td><td>-208</td></tr>
<tr><td>chr5</td><td>76612904</td><td>76638269</td><td>Tmem165</td><td>0</td><td>+</td><td>MACS_peak_12207</td><td>-20316</td></tr>
<tr><td>chr5</td><td>77403725</td><td>77428960</td><td>Srp72</td><td>0</td><td>+</td><td>MACS_peak_12217</td><td>-24332</td></tr>
<tr><td>chr5</td><td>77445047</td><td>77490547</td><td>1700023E</td><td>0</td><td>+</td><td>MACS_peak_12217</td><td>16990</td></tr>
<tr><td>chr5</td><td>77433079</td><td>77438502</td><td>Arl9</td><td>0</td><td>+</td><td>MACS_peak_12217</td><td>5022</td></tr>
<tr><td>chr5</td><td>77694518</td><td>77712722</td><td>Rest</td><td>0</td><td>+</td><td>MACS_peak_12218|MACS_peak_12220</td><td>-9781|-17119</td></tr>
<tr><td>chr5</td><td>77723194</td><td>77739111</td><td>2610024G</td><td>0</td><td>-</td><td>MACS_peak_12220</td><td>-27474</td></tr>
<tr><td>chr5</td><td>77739508</td><td>77778352</td><td>Polr2b</td><td>0</td><td>+</td><td>MACS_peak_12220</td><td>27871</td></tr>
<tr><td>chr5</td><td>81450617</td><td>82224755</td><td>Lphn3</td><td>0</td><td>+</td><td>MACS_peak_12227</td><td>456</td></tr>
<tr><td>chr5</td><td>86441049</td><td>86494608</td><td>Cenpc1</td><td>0</td><td>-</td><td>MACS_peak_12231</td><td>-20189</td></tr>
<tr><td>chr5</td><td>86500852</td><td>86533018</td><td>Stap1</td><td>0</td><td>+</td><td>MACS_peak_12231</td><td>26433</td></tr>
<tr><td>chr5</td><td>88983507</td><td>88985108</td><td>Utp3</td><td>0</td><td>+</td><td>MACS_peak_12237</td><td>10028</td></tr>
<tr><td>chr5</td><td>88948834</td><td>88956922</td><td>Igj</td><td>0</td><td>-</td><td>MACS_peak_12237</td><td>16557</td></tr>
<tr><td>chr5</td><td>8966047</td><td>8997146</td><td>Crot</td><td>0</td><td>-</td><td>MACS_peak_11897|MACS_peak_11898|N</td><td>-28229|-19635|-9310</td></tr>
<tr><td>chr5</td><td>9100736</td><td>9118983</td><td>4930420K</td><td>0</td><td>+</td><td>MACS_peak_11901|MACS_peak_11902</td><td>-20533|-21160</td></tr>
<tr><td>chr5</td><td>91503642</td><td>91522675</td><td>Ereg</td><td>0</td><td>+</td><td>MACS_peak_12256|MACS_peak_12258|N</td><td>22913|-8|-12200|-14543|-17240</td></tr>
<tr><td>chr5</td><td>91456542</td><td>91464238</td><td>Epgn</td><td>0</td><td>+</td><td>MACS_peak_12256</td><td>-24187</td></tr>
<tr><td>chr5</td><td>9118867</td><td>9161776</td><td>Dmtf1</td><td>0</td><td>-</td><td>MACS_peak_11903|MACS_peak_11903|N</td><td>-12822|-12822|-11341|-11341|-96|-96</td></tr>
</table>

chr5	91568640	91577458	Areg	0	+	MACS_peak_12262|MACS_peak_12263|M	-129|-1396|-3927|-4797|-7068|-8685
chr5	91786286	91831939	Btc	0	-	MACS_peak_12272	-144
chr5	92844565	92864225	Nup54	0	-	MACS_peak_12278|MACS_peak_12279	14705|16205
chr5	92872898	92934634	Scarb2	0	-	MACS_peak_12284	-20334
chr5	93032076	93035605	Stbd1	0	+	MACS_peak_12286	-168
chr5	97511347	97540615	Paqr3	0	-	MACS_peak_12306|MACS_peak_12307|M	-23517|-17778|-16707|-15921|-15651|-14070|-11949|-8160|-3360|-224
chr5	98313706	98459981	Antxr2	0	-	MACS_peak_12319	-180
chr6	100523075	100621151	Shq1	0	-	MACS_peak_13392	-118
chr6	100783631	100818711	Ppp4r2	0	+	MACS_peak_13393	28999
chr6	106660372	106699031	Il5ra	0	-	MACS_peak_13394	28975
chr6	106728238	106750068	Crbn	0	-	MACS_peak_13394|MACS_peak_13394|M	-22062|-22062|-20676|-20676|-18020|-18020|-4133|-4133
chr6	106719165	106732463	Trnt1	0	+	MACS_peak_13394|MACS_peak_13395|M	-8841|-10227|-12883|-26770
chr6	108610622	108616919	Bhlhe40	0	+	MACS_peak_13410|MACS_peak_13411	-192|-569
chr6	108778634	108809350	Edem1	0	+	MACS_peak_13414	-28769
chr6	113027632	113103418	Setd5	0	+	MACS_peak_13439	-29585
chr6	113020394	113027238	Gt(ROSA)26	0	-	MACS_peak_13439|MACS_peak_13439|M	29979|29979|29979
chr6	113187836	113231386	Mtmr14	0	+	MACS_peak_13442	-15924
chr6	113232300	113255565	Cpne9	0	+	MACS_peak_13442|MACS_peak_13443	28540|13643
chr6	113276969	113284180	Ogg1	0	+	MACS_peak_13446	-10266
chr6	113284117	113293916	Camk1	0	-	MACS_peak_13446	-6681
chr6	113408888	113420137	Il17re	0	+	MACS_peak_13450|MACS_peak_13450|M	-21473|-21473|-21884
chr6	113433562	113443332	Creld1	0	+	MACS_peak_13450	3201
chr6	113421448	113433132	Il17rc	0	+	MACS_peak_13450	-8913
chr6	113444088	113451812	Prrt3	0	-	MACS_peak_13450	-21451
chr6	114812107	114871770	Vgll4	0	-	MACS_peak_13453	-229
chr6	115881944	115888848	Rho	0	+	MACS_peak_13458|MACS_peak_13459|M	26043|17996|14914
chr6	115894956	115900251	H1foo	0	+	MACS_peak_13460	27926
chr6	116214252	116279644	Anubl1	0	+	MACS_peak_13462	9123
chr6	118452581	118512274	Ankrd26	0	-	MACS_peak_13470	-13366
chr6	11875880	12031197	Phf14	0	+	MACS_peak_12902|MACS_peak_12902	-326|-326
chr6	11850372	11857446	Ndufa4	0	-	MACS_peak_12902	18760
chr6	119852715	119872840	Rad52	0	+	MACS_peak_13479|MACS_peak_13479|M	-10366|-10366|-10366|-10366|-24448|-24448|-24448|-24448
chr6	120274715	120307378	Ccdc77	0	-	MACS_peak_13500	24235
chr6	120314116	120394592	Kdm5a	0	+	MACS_peak_13500	-17497
chr6	120413214	120433744	Il17ra	0	+	MACS_peak_13502|MACS_peak_13503	-18489|-20250
chr6	120438956	120443825	Cecr6	0	-	MACS_peak_13502|MACS_peak_13503	-12122|-10361
chr6	120723785	120744000	Slc25a18	0	+	MACS_peak_13505	-27326
chr6	120745261	120772703	Atp6v1e1	0	-	MACS_peak_13505	-21592
chr6	120843136	120866838	Bid	0	-	MACS_peak_13509	-19527
chr6	122267748	122287033	Phc1	0	-	MACS_peak_13511|MACS_peak_13511|M	-27943|-27943|-79|-79
chr6	122259027	122267695	M6pr	0	+	MACS_peak_13511|MACS_peak_13513	-63|-27927
chr6	122220618	122232851	Klrg1	0	-	MACS_peak_13511	26239
chr6	122770201	122795382	Foxj2	0	+	MACS_peak_13517|MACS_peak_13518|M	-306|-688|-11286
chr6	122797157	122806175	C3ar1	0	-	MACS_peak_13520|MACS_peak_13526|M	-24688|18444|24312|24552|25366
chr6	122824574	122838959	Necap1	0	+	MACS_peak_13526|MACS_peak_13527|M	-45|-5913|-6153|-6967|-12249
chr6	123212124	123225285	Clec4d	0	+	MACS_peak_13531|MACS_peak_13531	-5956|-5956
chr6	123231806	123239889	Clec4e	0	-	MACS_peak_13531	-21809
chr6	123652838	123692257	Vmn2r23	0	+	MACS_peak_13533	9230
chr6	124346833	124365085	Pex5	0	-	MACS_peak_13534|MACS_peak_13534|M	-17554|-17554|-16202|-16202|-10912|-10912
chr6	124670735	124688727	Ptpn6	0	-	MACS_peak_13543|MACS_peak_13543|M	24393|29957|27499
chr6	124692561	124706505	Atn1	0	-	MACS_peak_13543|MACS_peak_13544	6615|9721
chr6	124710072	124719527	Eno2	0	-	MACS_peak_13543|MACS_peak_13544	-6407|-3301
chr6	124689201	124691097	Grcc10	0	-	MACS_peak_13543|MACS_peak_13544	22023|25129
chr6	124719880	124729736	Lrrc23	0	-	MACS_peak_13543|MACS_peak_13544	-16616|-13510
chr6	124690818	124693852	Rnu7	0	-	MACS_peak_13543|MACS_peak_13544	19268|22374
chr6	124999944	125004211	Acrbp	0	+	MACS_peak_13546|MACS_peak_13546	2364|2364
chr6	125021294	125032489	Lpar5	0	+	MACS_peak_13546|MACS_peak_13546	23714|20357
chr6	124989865	124999282	Ing4	0	+	MACS_peak_13546	-7715
chr6	125081900	125094771	Nop2	0	+	MACS_peak_13548|MACS_peak_13549|M	26912|26606|24320|21805|9682|2966|-2755
chr6	125046180	125080519	Chd4	0	+	MACS_peak_13548|MACS_peak_13549|M	-8808|-9114|-11400|-13915|-26038
chr6	125095268	125111794	Iffo1	0	+	MACS_peak_13553|MACS_peak_13553|M	23050|23040|16334|16324|10613|10603
chr6	125182639	125187045	Cd27	0	-	MACS_peak_13557|MACS_peak_13557	4847|4847
chr6	125165598	125172324	Vamp1	0	+	MACS_peak_13557|MACS_peak_13557	-26294|-26294
chr6	125174238	125181878	Tapbpl	0	-	MACS_peak_13557	10014
chr6	125215542	125223795	4930417O	0	+	MACS_peak_13557	23650
chr6	125181293	125189952	E130112N	0	+	MACS_peak_13557	-10599
chr6	125271357	125294958	Scnn1a	0	+	MACS_peak_13559	-28549
chr6	125299740	125312501	Tnfrsf1a	0	+	MACS_peak_13559|MACS_peak_13560|M	-166|-7179|-8067
chr6	125312668	125330522	Plekhg6	0	-	MACS_peak_13560|MACS_peak_13561	-23603|-22715
chr6	127075726	127101066	Ccnd2	0	-	MACS_peak_13572	-2276
chr6	128943037	128956667	BC064078	0	+	MACS_peak_13591	-15884
chr6	129994056	130015320	Klra33	0	-	MACS_peak_13601	-5667
chr6	129994034	130017199	Klra4	0	-	MACS_peak_13601	-7546
chr6	129993749	130017276	Klra18	0	-	MACS_peak_13601	-7623
chr6	131169252	131197380	Klra2	0	-	MACS_peak_13607|MACS_peak_13607|M	-17215|-17215|-2039|-2039
chr6	131234406	131243262	Magohb	0	-	MACS_peak_13610	-1377
chr6	131249161	131263845	Styk1	0	-	MACS_peak_13610	-21960
chr6	135015679	135034724	Gprc5a	0	+	MACS_peak_13640	-1020
chr6	135087536	135118233	Hebp1	0	-	MACS_peak_13644	-4105
chr6	13530688	13558063	Tmem168	0	-	MACS_peak_12937	-25742
chr6	135312948	135333191	Emp1	0	+	MACS_peak_13646	-17270
chr6	142310419	142335607	Recql	0	-	MACS_peak_13679	15613
chr6	142335762	142352378	Golt1b	0	+	MACS_peak_13679	-15458
chr6	144942355	144997332	Bcat1	0	-	MACS_peak_13680|MACS_peak_13680	-1489|-28834
chr6	145756902	145766104	Rassf8	0	+	MACS_peak_13681	-7129
chr6	146060001	146450434	Itpr2	0	-	MACS_peak_13736|MACS_peak_13736	-18|-18
chr6	146526432	146547720	Fgfr1op2	0	+	MACS_peak_13738	-29713
chr6	146550797	146583114	Tm7sf3	0	-	MACS_peak_13738	-26969
chr6	146498153	146526357	4933424B(	0	-	MACS_peak_13738	29788
chr6	146754051	146781168	Arntl2	0	+	MACS_peak_13741	-4178
chr6	146981058	146982040	Rep15	0	+	MACS_peak_13742	2851
chr6	146991291	147019424	Mrps35	0	+	MACS_peak_13742	13084
chr6	148719205	148779989	Ipo8	0	-	MACS_peak_13751	-19360
chr6	148869580	148894954	Fam60a	0	-	MACS_peak_13752|MACS_peak_13753	-24380|-20359
chr6	148791033	148844648	Caprin2	0	-	MACS_peak_13752|MACS_peak_13753	25926|29947
chr6	149090118	149099692	4833442J1	0	+	MACS_peak_13755	-5626
chr6	149106098	149137234	Amn1	0	-	MACS_peak_13758	-5279
chr6	149257935	149284185	2810474O	0	+	MACS_peak_13759	-8239
chr6	24547770	24555414	Lmod2	0	+	MACS_peak_12949	-17394
chr6	28392334	28399340	Pax4	0	-	MACS_peak_12953|MACS_peak_12953|M	-28444|-28444|-28444
chr6	28373639	28376499	Arf5	0	+	MACS_peak_12953	2743
chr6	28377900	28388622	Fscn3	0	+	MACS_peak_12953	7004
chr6	28366602	28371724	Gcc1	0	-	MACS_peak_12953	-828
chr6	29119229	29129584	Prrt4	0	-	MACS_peak_12959	-20702
chr6	29073572	29114724	Rbm28	0	-	MACS_peak_12959	-5842
chr6	29231140	29240676	Fam71f2	0	+	MACS_peak_12961|MACS_peak_12963	-4874|-17192
chr6	29222487	29225449	2310016C(	0	+	MACS_peak_12961|MACS_peak_12961|M	-13527|-13389|-25845|-25707
chr6	29269139	29286022	Fam71f1	0	+	MACS_peak_12963	20807
chr6	29326670	29330513	Opn1sw	0	-	MACS_peak_12966|MACS_peak_12972|M	-19121|18813|29636
chr6	29298118	29326675	Calu	0	+	MACS_peak_12966|MACS_peak_12966	-13274|-13274
chr6	29346635	29376954	Ccdc136	0	+	MACS_peak_12972|MACS_peak_12973	-2691|-13514
chr6	29383152	29411888	Flnc	0	+	MACS_peak_12973|MACS_peak_12974|M	23003|-388|-5423|-7070|-7318|-7764|-8025|-8380|-9797|-10281|-10574|-11937|-12287|-12568|-12939|-13161|-13787|-14538|-15458|-157(
chr6	29417782	29420509	Atp6v1f	0	+	MACS_peak_12975|MACS_peak_12976|M	29207|27560|27312|26866|26605|26250|24833|24349|24056|22693|22343|22062|21691|21469|20843|20092|19172|18866|16048|1552
chr6	29421436	29423428	Gm9047	0	+	MACS_peak_12980|MACS_peak_12981|M	29904|28487|28003|27710|26347|25997|25716|25345|25123|24497|23746|22826|22520|19702|19181|17773|17060|15973|15355|1502
chr6	29809595	29862309	Ahcyl2	0	+	MACS_peak_13009	-24080
chr6	29867012	29909680	Fam40b	0	+	MACS_peak_13010|MACS_peak_13010	5643|5643
chr6	30161288	30193803	Ube2h	0	-	MACS_peak_13012	-22069
chr6	30688062	30698457	Mest	0	+	MACS_peak_13015	-10680
chr6	30691298	30691395	Mir335	0	+	MACS_peak_13015	-7444
chr6	33199149	33922930	Exoc4	0	+	MACS_peak_13026	-28141
chr6	34992708	35083737	Cnot4	0	-	MACS_peak_13041|MACS_peak_13041|M	-136|-136|-136|-136|-136
chr6	38237393	38249259	Zc3hav1l	0	-	MACS_peak_13054|MACS_peak_13056|M	13140|20513|25979

<table>
<tr><td>chr6</td><td>38260496</td><td>38304603</td><td>Zc3hav1</td><td>0</td><td>-</td><td>MACS_peak_13057|MACS_peak_13057|M</td><td>-29365|-29365|-347|-347</td></tr>
<tr><td>chr6</td><td>38331523</td><td>38377647</td><td>Ttc26</td><td>0</td><td>+</td><td>MACS_peak_13059|MACS_peak_13060</td><td>27267|-13622</td></tr>
<tr><td>chr6</td><td>38501443</td><td>38559469</td><td>Luc7l2</td><td>0</td><td>+</td><td>MACS_peak_13061|MACS_peak_13061|M</td><td>-6507|-6507|-6507|-10436|-10436|-10436|-12401|-12401|-12401</td></tr>
<tr><td>chr6</td><td>38484860</td><td>38489449</td><td>1110001J0</td><td>0</td><td>+</td><td>MACS_peak_13061|MACS_peak_13062|M</td><td>-23090|-27019|-28984</td></tr>
<tr><td>chr6</td><td>38576659</td><td>38587239</td><td>Klrg2</td><td>0</td><td>-</td><td>MACS_peak_13064</td><td>-17457</td></tr>
<tr><td>chr6</td><td>39036410</td><td>39068348</td><td>Parp12</td><td>0</td><td>-</td><td>MACS_peak_13075|MACS_peak_13078</td><td>27156|29413</td></tr>
<tr><td>chr6</td><td>39086618</td><td>39156772</td><td>Jhdm1d</td><td>0</td><td>-</td><td>MACS_peak_13088</td><td>-285</td></tr>
<tr><td>chr6</td><td>39331426</td><td>39340378</td><td>Rab19</td><td>0</td><td>+</td><td>MACS_peak_13091</td><td>-23334</td></tr>
<tr><td>chr6</td><td>39347819</td><td>39370368</td><td>Mkrn1</td><td>0</td><td>-</td><td>MACS_peak_13091</td><td>-15608</td></tr>
<tr><td>chr6</td><td>39284771</td><td>39327706</td><td>Slc37a3</td><td>0</td><td>-</td><td>MACS_peak_13091</td><td>27054</td></tr>
<tr><td>chr6</td><td>39542581</td><td>39549470</td><td>Ndufb2</td><td>0</td><td>+</td><td>MACS_peak_13094|MACS_peak_13095</td><td>12867|5141</td></tr>
<tr><td>chr6</td><td>39412376</td><td>39507833</td><td>Dennd2a</td><td>0</td><td>-</td><td>MACS_peak_13094|MACS_peak_13095</td><td>21881|29607</td></tr>
<tr><td>chr6</td><td>39523874</td><td>39538768</td><td>Adck2</td><td>0</td><td>+</td><td>MACS_peak_13094|MACS_peak_13095</td><td>-5840|-13566</td></tr>
<tr><td>chr6</td><td>40275476</td><td>40346760</td><td>Agk</td><td>0</td><td>+</td><td>MACS_peak_13098</td><td>-4076</td></tr>
<tr><td>chr6</td><td>41555480</td><td>41570506</td><td>Ephb6</td><td>0</td><td>+</td><td>MACS_peak_13107|MACS_peak_13107|M</td><td>-9202|-9202|-11031|-11031|-12666|-12666|-13814|-13814</td></tr>
<tr><td>chr6</td><td>41570619</td><td>41586404</td><td>Trpv6</td><td>0</td><td>-</td><td>MACS_peak_13107|MACS_peak_13109|M</td><td>-21722|-19893|-18258|-17110</td></tr>
<tr><td>chr6</td><td>42622545</td><td>42643058</td><td>Fam115a</td><td>0</td><td>-</td><td>MACS_peak_13115|MACS_peak_13116</td><td>-22937|-19573</td></tr>
<tr><td>chr6</td><td>42573041</td><td>42595040</td><td>Fam115c</td><td>0</td><td>-</td><td>MACS_peak_13115|MACS_peak_13116</td><td>25081|28445</td></tr>
<tr><td>chr6</td><td>4455696</td><td>4491543</td><td>Col1a2</td><td>0</td><td>+</td><td>MACS_peak_12854</td><td>-13584</td></tr>
<tr><td>chr6</td><td>5170851</td><td>5206233</td><td>Pon3</td><td>0</td><td>-</td><td>MACS_peak_12887</td><td>-23</td></tr>
<tr><td>chr6</td><td>52139685</td><td>52141702</td><td>Hoxa4</td><td>0</td><td>-</td><td>MACS_peak_13149</td><td>12596</td></tr>
<tr><td>chr6</td><td>52119061</td><td>52163066</td><td>Hoxa3</td><td>0</td><td>-</td><td>MACS_peak_13149</td><td>-8768</td></tr>
<tr><td>chr6</td><td>52151752</td><td>52154586</td><td>Hoxa5</td><td>0</td><td>-</td><td>MACS_peak_13149</td><td>-288</td></tr>
<tr><td>chr6</td><td>52156363</td><td>52158623</td><td>Hoxa6</td><td>0</td><td>-</td><td>MACS_peak_13149</td><td>-4325</td></tr>
<tr><td>chr6</td><td>52165622</td><td>52168572</td><td>Hoxa7</td><td>0</td><td>-</td><td>MACS_peak_13149</td><td>-14274</td></tr>
<tr><td>chr6</td><td>52174052</td><td>52177369</td><td>Hoxa9</td><td>0</td><td>-</td><td>MACS_peak_13149</td><td>-23071</td></tr>
<tr><td>chr6</td><td>52151122</td><td>52163596</td><td>2700086A(</td><td>0</td><td>+</td><td>MACS_peak_13149</td><td>-3176</td></tr>
<tr><td>chr6</td><td>52180079</td><td>52180164</td><td>Mir196b</td><td>0</td><td>-</td><td>MACS_peak_13149</td><td>-25866</td></tr>
<tr><td>chr6</td><td>5214623</td><td>5248373</td><td>Pon2</td><td>0</td><td>-</td><td>MACS_peak_12888</td><td>-9324</td></tr>
<tr><td>chr6</td><td>52663722</td><td>52716771</td><td>Tax1bp1</td><td>0</td><td>+</td><td>MACS_peak_13150|MACS_peak_13151</td><td>-28337|-29031</td></tr>
<tr><td>chr6</td><td>54987994</td><td>55029498</td><td>Gars</td><td>0</td><td>+</td><td>MACS_peak_13157|MACS_peak_13158|M</td><td>-110|-10116|-22983|-25160|-27528</td></tr>
<tr><td>chr6</td><td>56727518</td><td>56747807</td><td>Kbtbd2</td><td>0</td><td>-</td><td>MACS_peak_13175</td><td>-18826</td></tr>
<tr><td>chr6</td><td>56782052</td><td>56829354</td><td>Fkbp9</td><td>0</td><td>+</td><td>MACS_peak_13176|MACS_peak_13177</td><td>-18599|-19369</td></tr>
<tr><td>chr6</td><td>57793557</td><td>57794451</td><td>Vmn1r21</td><td>0</td><td>-</td><td>MACS_peak_13185</td><td>-19454</td></tr>
<tr><td>chr6</td><td>57702257</td><td>57775119</td><td>Vopp1</td><td>0</td><td>-</td><td>MACS_peak_13185</td><td>-122</td></tr>
<tr><td>chr6</td><td>65067287</td><td>65094724</td><td>Hpgds</td><td>0</td><td>-</td><td>MACS_peak_13190</td><td>24455</td></tr>
<tr><td>chr6</td><td>70794520</td><td>70855234</td><td>Eif2ak3</td><td>0</td><td>+</td><td>MACS_peak_13200</td><td>-13922</td></tr>
<tr><td>chr6</td><td>71323420</td><td>71329165</td><td>Cd8a</td><td>0</td><td>+</td><td>MACS_peak_13210|MACS_peak_13210|M</td><td>-19337|-19337|-21286|-21286</td></tr>
<tr><td>chr6</td><td>71338627</td><td>71390631</td><td>Rmnd5a</td><td>0</td><td>-</td><td>MACS_peak_13212</td><td>-11544</td></tr>
<tr><td>chr6</td><td>72364326</td><td>72380701</td><td>Ggcx</td><td>0</td><td>+</td><td>MACS_peak_13214</td><td>-12243</td></tr>
<tr><td>chr6</td><td>72382792</td><td>72389552</td><td>Mat2a</td><td>0</td><td>-</td><td>MACS_peak_13214</td><td>-12983</td></tr>
<tr><td>chr6</td><td>72494432</td><td>72512972</td><td>Capg</td><td>0</td><td>+</td><td>MACS_peak_13216|MACS_peak_13217</td><td>29134|27306</td></tr>
<tr><td>chr6</td><td>7625170</td><td>7643182</td><td>Asns</td><td>0</td><td>-</td><td>MACS_peak_12889|MACS_peak_12891|M</td><td>-17817|-16905|-15901|-15175|-13002|-11870|-11155|-10819|-7867|-3795|-17</td></tr>
<tr><td>chr6</td><td>82675020</td><td>82724448</td><td>Hk2</td><td>0</td><td>-</td><td>MACS_peak_13234|MACS_peak_13235|M</td><td>-29531|-25191|-14274|-266</td></tr>
<tr><td>chr6</td><td>83462902</td><td>83486245</td><td>Actg2</td><td>0</td><td>-</td><td>MACS_peak_13260</td><td>14753</td></tr>
<tr><td>chr6</td><td>83493200</td><td>83522498</td><td>Stambp</td><td>0</td><td>-</td><td>MACS_peak_13260</td><td>-21500</td></tr>
<tr><td>chr6</td><td>85163045</td><td>85283416</td><td>Sfxn5</td><td>0</td><td>-</td><td>MACS_peak_13272</td><td>7814</td></tr>
<tr><td>chr6</td><td>85286285</td><td>85286367</td><td>Mir705</td><td>0</td><td>-</td><td>MACS_peak_13272</td><td>4863</td></tr>
<tr><td>chr6</td><td>86354212</td><td>86383399</td><td>Tia1</td><td>0</td><td>+</td><td>MACS_peak_13280|MACS_peak_13280|M</td><td>12465|12465|12465|11978|11978|11978|7166|7166|7166</td></tr>
<tr><td>chr6</td><td>86315676</td><td>86320052</td><td>Fam136a</td><td>0</td><td>+</td><td>MACS_peak_13280|MACS_peak_13281</td><td>-26071|-26558</td></tr>
<tr><td>chr6</td><td>86335999</td><td>86347144</td><td>Pcyox1</td><td>0</td><td>-</td><td>MACS_peak_13280|MACS_peak_13281|M</td><td>-5397|-4910|-98</td></tr>
<tr><td>chr6</td><td>86321533</td><td>86328896</td><td>Snrpg</td><td>0</td><td>+</td><td>MACS_peak_13280|MACS_peak_13281|M</td><td>-20214|-20701|-25513</td></tr>
<tr><td>chr6</td><td>86597038</td><td>86619153</td><td>Mxd1</td><td>0</td><td>-</td><td>MACS_peak_13285</td><td>-20551</td></tr>
<tr><td>chr6</td><td>86578167</td><td>86579698</td><td>Asprv1</td><td>0</td><td>+</td><td>MACS_peak_13285</td><td>-20435</td></tr>
<tr><td>chr6</td><td>87054739</td><td>87062997</td><td>D6Ertd527</td><td>0</td><td>+</td><td>MACS_peak_13291|MACS_peak_13291|M</td><td>22215|22215|14596|14596</td></tr>
<tr><td>chr6</td><td>87792608</td><td>87801100</td><td>Cnbp</td><td>0</td><td>-</td><td>MACS_peak_13298|MACS_peak_13298|M</td><td>-5374|-5374|-5374</td></tr>
<tr><td>chr6</td><td>87768441</td><td>87788753</td><td>Isy1</td><td>0</td><td>-</td><td>MACS_peak_13298</td><td>6973</td></tr>
<tr><td>chr6</td><td>87837933</td><td>87863588</td><td>Copg</td><td>0</td><td>+</td><td>MACS_peak_13299|MACS_peak_13299</td><td>-5936|-5904</td></tr>
<tr><td>chr6</td><td>87863969</td><td>87886607</td><td>8430410A1</td><td>0</td><td>+</td><td>MACS_peak_13299</td><td>20100</td></tr>
<tr><td>chr6</td><td>88785907</td><td>88791929</td><td>Abtb1</td><td>0</td><td>-</td><td>MACS_peak_13305</td><td>-93</td></tr>
<tr><td>chr6</td><td>88833467</td><td>88848774</td><td>Mcm2</td><td>0</td><td>-</td><td>MACS_peak_13309|MACS_peak_13310</td><td>11422|13186</td></tr>
<tr><td>chr6</td><td>88852244</td><td>88862234</td><td>Tpra1</td><td>0</td><td>+</td><td>MACS_peak_13309|MACS_peak_13310</td><td>-7952|-9716</td></tr>
<tr><td>chr6</td><td>89266309</td><td>89312607</td><td>Plxna1</td><td>0</td><td>-</td><td>MACS_peak_13314|MACS_peak_13315</td><td>-19614|-18577</td></tr>
<tr><td>chr6</td><td>89593981</td><td>89625523</td><td>Txnrd3</td><td>0</td><td>+</td><td>MACS_peak_13317|MACS_peak_13317|M</td><td>-19153|-19153|-19153|-19153</td></tr>
<tr><td>chr6</td><td>90319487</td><td>90335388</td><td>Zxdc</td><td>0</td><td>+</td><td>MACS_peak_13318|MACS_peak_13318|M</td><td>-14116|-14116|-23453|-23453|-24964|-24964</td></tr>
<tr><td>chr6</td><td>91423744</td><td>91438452</td><td>Tmem43</td><td>0</td><td>+</td><td>MACS_peak_13345|MACS_peak_13349|M</td><td>-5844|-8540|-11624|-19181|-25728</td></tr>
<tr><td>chr6</td><td>91414269</td><td>91423417</td><td>Chchd4</td><td>0</td><td>-</td><td>MACS_peak_13345|MACS_peak_13349|M</td><td>6171|8867|11951|19508|26055</td></tr>
<tr><td>chr6</td><td>91439301</td><td>91465882</td><td>Xpc</td><td>0</td><td>-</td><td>MACS_peak_13353|MACS_peak_13354</td><td>-22957|-16410</td></tr>
<tr><td>chr6</td><td>91466028</td><td>91472614</td><td>Lsm3</td><td>0</td><td>+</td><td>MACS_peak_13353|MACS_peak_13354</td><td>23103|16556</td></tr>
<tr><td>chr6</td><td>92119516</td><td>92134017</td><td>Mrps25</td><td>0</td><td>-</td><td>MACS_peak_13359|MACS_peak_13360</td><td>-19792|17525</td></tr>
<tr><td>chr6</td><td>92136705</td><td>92164805</td><td>Zfyve20</td><td>0</td><td>-</td><td>MACS_peak_13360</td><td>-13263</td></tr>
<tr><td>chr6</td><td>97101943</td><td>97129118</td><td>Tmf1</td><td>0</td><td>-</td><td>MACS_peak_13379|MACS_peak_13380|M</td><td>-10949|-6849|-5773|-2753</td></tr>
<tr><td>chr6</td><td>97060937</td><td>97098877</td><td>A130022J1</td><td>0</td><td>-</td><td>MACS_peak_13379|MACS_peak_13380|M</td><td>19292|23392|24468|27488</td></tr>
<tr><td>chr6</td><td>97133809</td><td>97155636</td><td>Uba3</td><td>0</td><td>-</td><td>MACS_peak_13382|MACS_peak_13382</td><td>-29271|-29271</td></tr>
<tr><td>chr7</td><td>104457889</td><td>104474474</td><td>Usp35</td><td>0</td><td>-</td><td>MACS_peak_14464|MACS_peak_14467</td><td>-25635|-21754</td></tr>
<tr><td>chr7</td><td>104480832</td><td>104498726</td><td>Kctd21</td><td>0</td><td>+</td><td>MACS_peak_14467</td><td>28112</td></tr>
<tr><td>chr7</td><td>104629465</td><td>104689907</td><td>Ints4</td><td>0</td><td>+</td><td>MACS_peak_14471</td><td>-10023</td></tr>
<tr><td>chr7</td><td>104845200</td><td>104867389</td><td>Clns1a</td><td>0</td><td>+</td><td>MACS_peak_14474</td><td>17685</td></tr>
<tr><td>chr7</td><td>105199569</td><td>105268003</td><td>Myo7a</td><td>0</td><td>-</td><td>MACS_peak_14480</td><td>-23801</td></tr>
<tr><td>chr7</td><td>105340924</td><td>105347985</td><td>B3gnt6</td><td>0</td><td>-</td><td>MACS_peak_14482|MACS_peak_14483</td><td>14731|16657</td></tr>
<tr><td>chr7</td><td>105499179</td><td>105509798</td><td>Tsku</td><td>0</td><td>-</td><td>MACS_peak_14485|MACS_peak_14485|M</td><td>-7750|-7790|-7750|-7750|-1833|-1873|-1833|-1833|-47|-87|-47|-47</td></tr>
<tr><td>chr7</td><td>105987354</td><td>106003257</td><td>Wnt11</td><td>0</td><td>+</td><td>MACS_peak_14488</td><td>-7599</td></tr>
<tr><td>chr7</td><td>106493884</td><td>106501749</td><td>Serpinh1</td><td>0</td><td>-</td><td>MACS_peak_14490|MACS_peak_14490|M</td><td>-6193|-6193|-6193|-217|-217|-217</td></tr>
<tr><td>chr7</td><td>106530058</td><td>106609494</td><td>Gdpd5</td><td>0</td><td>+</td><td>MACS_peak_14493</td><td>28526</td></tr>
<tr><td>chr7</td><td>106614513</td><td>106622528</td><td>Klhl35</td><td>0</td><td>+</td><td>MACS_peak_14494</td><td>27711</td></tr>
<tr><td>chr7</td><td>107520742</td><td>107618668</td><td>C2cd3</td><td>0</td><td>+</td><td>MACS_peak_14506|MACS_peak_14507</td><td>27203|-2162</td></tr>
<tr><td>chr7</td><td>107475246</td><td>107520406</td><td>Ppme1</td><td>0</td><td>-</td><td>MACS_peak_14506|MACS_peak_14507</td><td>-26867|2498</td></tr>
<tr><td>chr7</td><td>107792411</td><td>107806970</td><td>Plekhb1</td><td>0</td><td>-</td><td>MACS_peak_14508|MACS_peak_14508|M</td><td>-28564|-28564|-28564|-27073|-27073</td></tr>
<tr><td>chr7</td><td>107756099</td><td>107789781</td><td>Rab6</td><td>0</td><td>+</td><td>MACS_peak_14508|MACS_peak_14508</td><td>-22307|-22307</td></tr>
<tr><td>chr7</td><td>107697630</td><td>107756510</td><td>Mrpl48</td><td>0</td><td>-</td><td>MACS_peak_14508</td><td>21896</td></tr>
<tr><td>chr7</td><td>108145085</td><td>108160505</td><td>P2ry2</td><td>0</td><td>-</td><td>MACS_peak_14513</td><td>-14596</td></tr>
<tr><td>chr7</td><td>108438129</td><td>108450602</td><td>Atg16l2</td><td>0</td><td>-</td><td>MACS_peak_14516</td><td>-24560</td></tr>
<tr><td>chr7</td><td>108496582</td><td>108561100</td><td>Arap1</td><td>0</td><td>+</td><td>MACS_peak_14519|MACS_peak_14519|M</td><td>-25161|4892|12296|21158</td></tr>
<tr><td>chr7</td><td>108812281</td><td>108938682</td><td>Clpb</td><td>0</td><td>+</td><td>MACS_peak_14521</td><td>-214</td></tr>
<tr><td>chr7</td><td>109118356</td><td>109163472</td><td>Numa1</td><td>0</td><td>+</td><td>MACS_peak_14525</td><td>-7581</td></tr>
<tr><td>chr7</td><td>109259779</td><td>109265227</td><td>Chrna10</td><td>0</td><td>-</td><td>MACS_peak_14529</td><td>8502</td></tr>
<tr><td>chr7</td><td>109245392</td><td>109248743</td><td>Art5</td><td>0</td><td>-</td><td>MACS_peak_14529</td><td>24986</td></tr>
<tr><td>chr7</td><td>109250256</td><td>109259662</td><td>Art1</td><td>0</td><td>+</td><td>MACS_peak_14529</td><td>-23473</td></tr>
<tr><td>chr7</td><td>112706997</td><td>112730049</td><td>Apbb1</td><td>0</td><td>-</td><td>MACS_peak_14541</td><td>-15416</td></tr>
<tr><td>chr7</td><td>112702873</td><td>112706902</td><td>Smpd1</td><td>0</td><td>+</td><td>MACS_peak_14541</td><td>-11760</td></tr>
<tr><td>chr7</td><td>112740124</td><td>112748630</td><td>Hpx</td><td>0</td><td>-</td><td>MACS_peak_14543|MACS_peak_14544</td><td>13218|17991</td></tr>
<tr><td>chr7</td><td>112758983</td><td>112782013</td><td>Trim3</td><td>0</td><td>-</td><td>MACS_peak_14543|MACS_peak_14544</td><td>-20165|-15392</td></tr>
<tr><td>chr7</td><td>112789053</td><td>112790359</td><td>Fxc1</td><td>0</td><td>+</td><td>MACS_peak_14543|MACS_peak_14544</td><td>27205|22432</td></tr>
<tr><td>chr7</td><td>112782715</td><td>112788930</td><td>Arfip2</td><td>0</td><td>-</td><td>MACS_peak_14543|MACS_peak_14544</td><td>-27082|-22309</td></tr>
<tr><td>chr7</td><td>112885103</td><td>112891439</td><td>Ilk</td><td>0</td><td>+</td><td>MACS_peak_14546|MACS_peak_14546</td><td>-12408|-12273</td></tr>
<tr><td>chr7</td><td>112893360</td><td>112900721</td><td>Tpp1</td><td>0</td><td>-</td><td>MACS_peak_14546</td><td>-3210</td></tr>
<tr><td>chr7</td><td>112891407</td><td>112892852</td><td>Taf10</td><td>0</td><td>-</td><td>MACS_peak_14546</td><td>4659</td></tr>
<tr><td>chr7</td><td>112880722</td><td>112885014</td><td>Rrp8</td><td>0</td><td>-</td><td>MACS_peak_14546</td><td>12497</td></tr>
<tr><td>chr7</td><td>116282085</td><td>116313822</td><td>Lmo1</td><td>0</td><td>-</td><td>MACS_peak_14557</td><td>-26747</td></tr>
<tr><td>chr7</td><td>117205215</td><td>117238906</td><td>Zfp143</td><td>0</td><td>+</td><td>MACS_peak_14560|MACS_peak_14561|M</td><td>19654|14635|10276|8879|-18605</td></tr>
<tr><td>chr7</td><td>117161938</td><td>117198628</td><td>Ipo7</td><td>0</td><td>+</td><td>MACS_peak_14560|MACS_peak_14561</td><td>-23623|-28642</td></tr>
<tr><td>chr7</td><td>117189877</td><td>117190059</td><td>Snora23</td><td>0</td><td>+</td><td>MACS_peak_14560|MACS_peak_14561|M</td><td>4316|-703|-5062|-6459</td></tr>
<tr><td>chr7</td><td>117771182</td><td>117773333</td><td>Adm</td><td>0</td><td>+</td><td>MACS_peak_14571</td><td>12917</td></tr>
<tr><td>chr7</td><td>117451526</td><td>117758434</td><td>Sbf2</td><td>0</td><td>-</td><td>MACS_peak_14571</td><td>-169</td></tr>
<tr><td>chr7</td><td>117916117</td><td>117955909</td><td>Ampd3</td><td>0</td><td>+</td><td>MACS_peak_14577</td><td>-24789</td></tr>
<tr><td>chr7</td><td>118172464</td><td>118199891</td><td>Ctr9</td><td>0</td><td>+</td><td>MACS_peak_14588</td><td>-4846</td></tr>
<tr><td>chr7</td><td>120459157</td><td>120512853</td><td>Btbd10</td><td>0</td><td>-</td><td>MACS_peak_14601|MACS_peak_14602</td><td>-17502|-48</td></tr>
<tr><td>chr7</td><td>120529089</td><td>120532087</td><td>Pth</td><td>0</td><td>-</td><td>MACS_peak_14602</td><td>-19282</td></tr>
<tr><td>chr7</td><td>121359072</td><td>121398194</td><td>Copb1</td><td>0</td><td>-</td><td>MACS_peak_14605|MACS_peak_14606|M</td><td>-27988|-27620|-21398|-17823|-13985</td></tr>
<tr><td>chr7</td><td>123647887</td><td>123684102</td><td>Nucb2</td><td>0</td><td>+</td><td>MACS_peak_14616|MACS_peak_14616</td><td>-19976|-19976</td></tr>
<tr><td>chr7</td><td>126937700</td><td>126992455</td><td>2610020H(</td><td>0</td><td>+</td><td>MACS_peak_14632|MACS_peak_14632</td><td>-25301|-25358</td></tr>
<tr><td>chr7</td><td>126927340</td><td>126937572</td><td>Eri2</td><td>0</td><td>-</td><td>MACS_peak_14632</td><td>25429</td></tr>
</table>

chr7	127245939	127264500	Tmem159	0	+	MACS_peak_14639	-2304
chr7	12761541	12762460	V1rg10	0	+	MACS_peak_13795	-6417
chr7	12778313	12779240	Vmn1r80	0	+	MACS_peak_13795	10355
chr7	127778702	127803037	Uqcrc2	0	+	MACS_peak_14641	-84
chr7	127986396	128047515	Eef2k	0	+	MACS_peak_14644|MACS_peak_14645	-124|-15617
chr7	128061257	128090946	Polr3e	0	+	MACS_peak_14653|MACS_peak_14653|M	28021|28021|22496|22496|18288|18288|13920|13920
chr7	128785535	128850767	Usp31	0	-	MACS_peak_14663	-29092
chr7	129008624	129062030	Scnn1b	0	+	MACS_peak_14664	9748
chr7	129066352	129125207	Cog7	0	-	MACS_peak_14665	29000
chr7	129130235	129164712	Gga2	0	-	MACS_peak_14665|MACS_peak_14666	-10505|-9010
chr7	130114077	130146086	Rbbp6	0	+	MACS_peak_14669|MACS_peak_14669|M	-30|-30|-14460|-14460
chr7	132695795	132722988	Il4ra	0	+	MACS_peak_14673|MACS_peak_14674|M	-19|-12363|-20623
chr7	132851389	133018311	D430042O	0	+	MACS_peak_14686|MACS_peak_14687|M	28992|26692|8855|8443
chr7	132784467	132851202	Gtf3c1	0	-	MACS_peak_14686|MACS_peak_14687|M	-28805|-26505|-8668|-8256
chr7	133610512	133618644	Sh2b1	0	-	MACS_peak_14691|MACS_peak_14691|M	20377|20377|25063|25063
chr7	133630868	133634244	Tufm	0	+	MACS_peak_14691|MACS_peak_14691|M	-8153|-8153|-12839|-12839
chr7	133635221	133646816	Atxn2l	0	-	MACS_peak_14691|MACS_peak_14692	-7795|-3109
chr7	133690424	133709880	Eif3c	0	-	MACS_peak_14693|MACS_peak_14694|M	-14212|-9980|-9141|-8932|-8611|-7410|-3739|-1854
chr7	133714913	133727794	Cln3	0	-	MACS_peak_14694|MACS_peak_14694|M	-27894|-26850|-27055|-26011|-26846|-25802|-26525|-25481|-25324|-24280|-21653|-20609|-19768|-18724
chr7	133728521	133732606	Apob48r	0	+	MACS_peak_14694|MACS_peak_14695|M	28621|27782|27573|27252|26051|22380|20495
chr7	13353441	13361007	Zscan18	0	-	MACS_peak_13796	17689
chr7	13389126	13404209	Zfp329	0	-	MACS_peak_13796	-25513
chr7	133843287	133848268	Coro1a	0	-	MACS_peak_14703|MACS_peak_14704	-13167|-9231
chr7	133832440	133839297	Giyd2	0	-	MACS_peak_14703|MACS_peak_14703|M	-4196|-4196|-260|-260
chr7	133816383	133819871	Sult1a1	0	-	MACS_peak_14703|MACS_peak_14704	15230|19166
chr7	133839513	133840207	Bola2	0	+	MACS_peak_14703|MACS_peak_14704	4412|476
chr7	133938748	133942692	Aldoa	0	-	MACS_peak_14705|MACS_peak_14705|M	-22104|-23373|-22180
chr7	133924996	133929062	Tbx6	0	+	MACS_peak_14705	4408
chr7	133903139	133909330	Mapk3	0	+	MACS_peak_14705	-17449
chr7	133929381	133935985	Ppp4c	0	-	MACS_peak_14705	-15397
chr7	133909927	133919159	Gdpd3	0	+	MACS_peak_14705	-10661
chr7	133920488	133924028	Ypel3	0	+	MACS_peak_14705|MACS_peak_14705	-100|-100
chr7	134089521	134093095	Asphd1	0	-	MACS_peak_14714	-7336
chr7	134094242	134113783	Sez6l2	0	+	MACS_peak_14714	8483
chr7	134072392	134089123	Kctd13	0	+	MACS_peak_14714	-13367
chr7	134119862	134124013	Cdipt	0	+	MACS_peak_14716|MACS_peak_14716	-10644|-10605
chr7	134130374	134158108	Mvp	0	-	MACS_peak_14716	-27602
chr7	134525773	134530680	9130019O	0	-	MACS_peak_14731	-11249
chr7	134516050	134519564	Zfp747	0	-	MACS_peak_14731	-133
chr7	134534350	134537135	E430018J2	0	-	MACS_peak_14731	-17704
chr7	134628734	134635026	Fbrs	0	+	MACS_peak_14732|MACS_peak_14733|M	11336|9271|8959|6570|6226|5816
chr7	134615127	134620272	Prr14	0	+	MACS_peak_14732|MACS_peak_14733|M	-2271|-4336|-4648|-7037|-7381|-7791
chr7	134585649	134592672	Zfp689	0	-	MACS_peak_14732|MACS_peak_14733|M	24726|26791|27103|29492|29836
chr7	134653951	134656383	1700008J0	0	-	MACS_peak_14738	15571
chr7	134671410	134671517	Snora30	0	+	MACS_peak_14738	-544
chr7	134726601	134732109	Gm166	0	-	MACS_peak_14741|MACS_peak_14743	21733|23500
chr7	134732211	134747119	Rnf40	0	+	MACS_peak_14741|MACS_peak_14743	-21631|-23398
chr7	134750548	134757947	Zfp629	0	-	MACS_peak_14741|MACS_peak_14743	-4105|-2338
chr7	134747417	134747959	1700120K0	0	-	MACS_peak_14741|MACS_peak_14743	5883|7650
chr7	135076151	135090239	Prss36	0	-	MACS_peak_14749	-22086
chr7	135047586	135053178	Bckdk	0	+	MACS_peak_14749	-20567
chr7	135056030	135069276	Myst1	0	+	MACS_peak_14749	-12123
chr7	135069236	135073627	Prss8	0	-	MACS_peak_14749	-5474
chr7	135036576	135039131	Vkorc1	0	-	MACS_peak_14749	29022
chr7	135348948	135349880	Cox6a2	0	-	MACS_peak_14751|MACS_peak_14752|M	-650|-396|-31
chr7	135414893	135441645	BC017158	0	-	MACS_peak_14754	-612
chr7	137917582	137969411	Btbd16	0	+	MACS_peak_14760	22202
chr7	138009423	138056816	Plekha1	0	+	MACS_peak_14762	-11905
chr7	138514659	138531352	Pstk	0	+	MACS_peak_14771	28436
chr7	138452067	138465806	Cuzd1	0	-	MACS_peak_14771	20417
chr7	138486231	138506212	2310057M	0	-	MACS_peak_14771	-19989
chr7	138469444	138473013	1700007K0	0	-	MACS_peak_14771	13210
chr7	138478135	138480230	Fam24a	0	+	MACS_peak_14771	-8088
chr7	138554114	138589724	Acadsb	0	+	MACS_peak_14773	-13950
chr7	138532162	138553992	Ikzf5	0	-	MACS_peak_14773	14072
chr7	139749157	139768081	Oat	0	-	MACS_peak_14776	-12129
chr7	139903766	139978618	Fam53b	0	-	MACS_peak_14778	-27180
chr7	140019143	140044330	Mettl10	0	-	MACS_peak_14779	12361
chr7	140050907	140076794	Fam175b	0	+	MACS_peak_14779	-5784
chr7	140178693	140315166	Ctbp2	0	-	MACS_peak_14782	-282
chr7	140865952	140871744	Mmp21	0	-	MACS_peak_14783	-19840
chr7	140829357	140864700	2700050L0	0	+	MACS_peak_14783	-22547
chr7	141074882	141416780	Adam12	0	-	MACS_peak_14786	129
chr7	142840139	142843997	5830432E0	0	-	MACS_peak_14789|MACS_peak_14790|M	-28588|-28361|-27918|-27758|18829|29292
chr7	146434380	146453152	Pwwp2b	0	+	MACS_peak_14800|MACS_peak_14800	22226|22226
chr7	146399173	146414909	Lrrc27	0	+	MACS_peak_14800|MACS_peak_14800	-12981|-13268
chr7	146289536	146399206	Stk32c	0	-	MACS_peak_14800	12948
chr7	148031463	148032668	Scgb1c1	0	+	MACS_peak_14801|MACS_peak_14802	-13946|-17160
chr7	148049561	148067768	Sirt3	0	-	MACS_peak_14801|MACS_peak_14801|M	-22359|-22359|-22799|-19145|-19145|-19585
chr7	148068292	148084541	Psmd13	0	+	MACS_peak_14801|MACS_peak_14802	22883|19669
chr7	148039282	148042282	Bet1l	0	-	MACS_peak_14801|MACS_peak_14802	3127|6341
chr7	148033814	148036824	Odf3	0	+	MACS_peak_14801|MACS_peak_14802	-11595|-14809
chr7	148021394	148023867	1190003J1	0	+	MACS_peak_14801|MACS_peak_14802	-24015|-27229
chr7	148043295	148049630	Ric8	0	+	MACS_peak_14801|MACS_peak_14802	-2114|-5328
chr7	148264127	148276408	Pkp3	0	+	MACS_peak_14804|MACS_peak_14804	17079|18619
chr7	148247172	148258018	B4galnt4	0	+	MACS_peak_14804	124
chr7	148317184	148342053	Ptdss2	0	+	MACS_peak_14807|MACS_peak_14810	-132|-23623
chr7	148287117	148303705	Ano9	0	-	MACS_peak_14807	13611
chr7	148346225	148358750	Rnh1	0	-	MACS_peak_14810|MACS_peak_14810|M	-17943|-13778|-14028|-4095|70|-180
chr7	148375832	148379903	Hras1	0	-	MACS_peak_14819|MACS_peak_14819|M	-25248|-25248|-25248
chr7	148380028	148395954	Lrrc56	0	+	MACS_peak_14819|MACS_peak_14819|M	25373|25373|26386
chr7	148414686	148448650	Phrf1	0	+	MACS_peak_14822	-26143
chr7	148454983	148462685	Cdhr5	0	-	MACS_peak_14822|MACS_peak_14822	-21856|-21856
chr7	148464236	148465030	Sct	0	-	MACS_peak_14822	-24201
chr7	148449087	148452300	Irf7	0	-	MACS_peak_14822	-11471
chr7	148592433	148593223	Gm4535	0	+	MACS_peak_14824|MACS_peak_14826	14291|8036
chr7	148578058	148588875	Taldo1	0	+	MACS_peak_14824|MACS_peak_14826	-84|-6339
chr7	148594082	148600024	Pddc1	0	-	MACS_peak_14824|MACS_peak_14826	-21882|-15627
chr7	148545076	148551009	B230206H0	0	-	MACS_peak_14824	27133
chr7	149646774	149680504	Lsp1	0	+	MACS_peak_14848|MACS_peak_14848	-28524|-17551
chr7	149684740	149701913	Tnnt3	0	+	MACS_peak_14848|MACS_peak_14848|M	9442|9442|9442|9442|9442|9442|9442|9442
chr7	149676984	149692676	Gm14492	0	-	MACS_peak_14848	-17378
chr7	150699483	150734995	Nap1l4	0	-	MACS_peak_14856|MACS_peak_14858|M	8758|19936|20357|20831|21019|22449|25524|27485
chr7	150743609	150785947	Cars	0	-	MACS_peak_14861|MACS_peak_14862|M	-29933|-28503|-25428|-23467|-15314|-7503
chr7	150822629	150835543	Tnfrsf22	0	-	MACS_peak_14870|MACS_peak_14871|M	-12231|-6302|-4811
chr7	150793589	150813750	Tnfrsf26	0	-	MACS_peak_14870|MACS_peak_14871|M	9562|15491|16982
chr7	150851711	150871777	Tnfrsf23	0	-	MACS_peak_14875	-5863
chr7	151009071	151034315	Dhcr7	0	+	MACS_peak_14879	15128
chr7	150981498	151008746	Nadsyn1	0	-	MACS_peak_14879	-14803
chr7	150964269	150970405	Mrgpre	0	-	MACS_peak_14879	23538
chr7	16859392	16872144	Ccdc9	0	-	MACS_peak_13805|MACS_peak_13805	22980|25116
chr7	16894931	16903682	Bbc3	0	+	MACS_peak_13805	-193
chr7	16912402	16973134	Sae1	0	-	MACS_peak_13808	12028
chr7	16986544	17023045	Zc3h4	0	+	MACS_peak_13808	1382
chr7	17366694	17383623	Slc1a5	0	+	MACS_peak_13810|MACS_peak_13811|M	-573|-12416|-14595|-16538
chr7	17401237	17426279	Strn4	0	+	MACS_peak_13811|MACS_peak_13811|M	22127|22127|19948|19948|18005|18005|-29999|-29999
chr7	17394615	17402081	Fkrp	0	-	MACS_peak_13811|MACS_peak_13812|M	-22971|-20792|-18849|29155
chr7	17460665	17472650	Dact3	0	+	MACS_peak_13814|MACS_peak_13816	29429|21327
chr7	17428413	17455810	Prkd2	0	+	MACS_peak_13814|MACS_peak_13816	-2823|-10925
chr7	18298600	18346470	Ceacam5	0	+	MACS_peak_13831	28903
chr7	18257661	18267409	Psg-ps1	0	+	MACS_peak_13831	-12036
chr7	19470038	19475787	Pglyrp1	0	+	MACS_peak_13833|MACS_peak_13835|M	22110|20907|-6343

chr7	19425314	19444002	Mill2	0	+	MACS_peak_13833|MACS_peak_13833|M	-22614|-22614|-23817|-23817
chr7	19476232	19495753	Ccdc61	0	-	MACS_peak_13838	-19372
chr7	19640035	19659795	Rsph6a	0	+	MACS_peak_13840|MACS_peak_13840|M	20190|20190|14067|14067|11185|11185|5800|5800|1336|1336
chr7	19598631	19608888	Foxa3	0	-	MACS_peak_13840|MACS_peak_13843|M	10957|17080|19962|25347|29811
chr7	19609725	19639971	Sympk	0	+	MACS_peak_13840|MACS_peak_13843|M	-10120|-16243|-19125|-24510|-28974
chr7	19661548	19668124	Dmwd	0	+	MACS_peak_13847|MACS_peak_13848	27313|22849
chr7	19742473	19751476	Gipr	0	-	MACS_peak_13850|MACS_peak_13852|M	16215|20011|20189|25522
chr7	19766518	19791830	Eml2	0	+	MACS_peak_13850|MACS_peak_13850|M	-1173|-1173|-4969|-4969|-5147|-5147|-10480|-10480
chr7	19766813	19766911	Mir330	0	+	MACS_peak_13850|MACS_peak_13852|M	-878|-4674|-4852|-10185
chr7	19797886	19809525	Gpr4	0	+	MACS_peak_13852|MACS_peak_13853|M	26399|26221|20888
chr7	19842278	19857203	Vasp	0	-	MACS_peak_13858	-14510
chr7	19868015	19881509	Rtn2	0	+	MACS_peak_13858|MACS_peak_13859|M	25322|-11911|-20343|-13741|-22173
chr7	19862155	19865398	Ppm1n	0	-	MACS_peak_13858|MACS_peak_13859|M	-22705|22960|24790
chr7	19813737	19832166	Opa3	0	+	MACS_peak_13858	-28956
chr7	19888044	19895394	Fosb	0	-	MACS_peak_13859|MACS_peak_13860	-7036|-5206
chr7	19946564	19963882	Ppp1r13l	0	+	MACS_peak_13862|MACS_peak_13863|M	-7578|-8832|-10237|-22911
chr7	19930419	19940179	Ercc1	0	+	MACS_peak_13862|MACS_peak_13862|M	-23723|-23723|-24977|-24977|-26382|-26382
chr7	19967387	19981041	Ercc2	0	+	MACS_peak_13862|MACS_peak_13863|M	13245|11991|10586|-2088
chr7	19941359	19944832	Cd3eap	0	-	MACS_peak_13862|MACS_peak_13863|M	9310|10564|11969|24643
chr7	19971991	19972066	Mir343	0	+	MACS_peak_13862|MACS_peak_13863|M	17849|16595|15190|2516
chr7	19979788	19985205	Klc3	0	-	MACS_peak_13863|MACS_peak_13864|M	-29809|-28404|-15730
chr7	19996442	20006932	Ckm	0	+	MACS_peak_13866	26967
chr7	20011423	20043843	Mark4	0	-	MACS_peak_13868|MACS_peak_13869	-15276|193
chr7	20191570	20214787	Relb	0	-	MACS_peak_13871	-15608
chr7	20166391	20189817	Srsf16	0	-	MACS_peak_13871	9362
chr7	20286661	20300778	Tomm40	0	-	MACS_peak_13875|MACS_peak_13875|M	17630|17630|22763|22763
chr7	20309512	20334922	Pvrl2	0	-	MACS_peak_13875|MACS_peak_13875|M	-16514|-16514|-11381|-11381
chr7	20488928	20506492	Pvr	0	-	MACS_peak_13878|MACS_peak_13879|M	-11867|-10460|-6345|-4171|-104
chr7	20461090	20473314	Ceacam19	0	-	MACS_peak_13878|MACS_peak_13879|M	21311|22718|26833|29007
chr7	20522653	20536092	2210010C1	0	-	MACS_peak_13883	-29704
chr7	24593305	24594219	Vmn1r175	0	-	MACS_peak_13895	-8046
chr7	25247518	25260892	Plaur	0	+	MACS_peak_13900|MACS_peak_13901|M	-9477|-11833|-13080
chr7	25267041	25289552	Cadm4	0	+	MACS_peak_13900|MACS_peak_13901|M	10046|7690|6443
chr7	25216944	25230701	Irgc1	0	-	MACS_peak_13900|MACS_peak_13901|M	26294|28650|29897
chr7	25326676	25331024	2310033E0	0	-	MACS_peak_13909	-8260
chr7	25332168	25358457	Xrcc1	0	+	MACS_peak_13909	9404
chr7	25315666	25323618	Irgq	0	+	MACS_peak_13909	-7098
chr7	26006469	26012523	Zfp526	0	+	MACS_peak_13915	17818
chr7	25987858	26004878	Dedd2	0	-	MACS_peak_13915	-16227
chr7	26086377	26089152	Prr19	0	+	MACS_peak_13917	-15952
chr7	26102182	26150936	Megf8	0	+	MACS_peak_13917|MACS_peak_13919|M	-147|-17712|-22418|-24630
chr7	26080067	26082974	Pafah1b3	0	-	MACS_peak_13917	19355
chr7	26091126	26101214	Tmem145	0	+	MACS_peak_13917|MACS_peak_13919	-11203|-28768
chr7	26152634	26154743	Cnfn	0	-	MACS_peak_13921|MACS_peak_13921|M	-27931|-27931|-21408|-21408|-14313|-14313|-9892|-9892|-9011|-9011|10264|10264
chr7	26164545	26175125	Lipe	0	-	MACS_peak_13926|MACS_peak_13928|M	-29393|-10118|-15999|28655|22774
chr7	26180432	26351436	4732471J0	0	+	MACS_peak_13928|MACS_peak_13931	15425|-23348
chr7	26185071	26197905	Cxcl17	0	-	MACS_peak_13931	5875
chr7	26414870	26443780	Bckdha	0	-	MACS_peak_13938	28847
chr7	26472020	26490015	Tgfb1	0	+	MACS_peak_13938	-607
chr7	26444171	26453050	Exosc5	0	+	MACS_peak_13938	-28456
chr7	26466176	26471577	B9d2	0	+	MACS_peak_13938	-6451
chr7	26454160	26460185	Tmem91	0	-	MACS_peak_13938	12442
chr7	26541518	26573752	Axl	0	-	MACS_peak_13942|MACS_peak_13942|M	-24257|-24257|-24257|-14115|-14115|-14115|-2801|-2801|-2801
chr7	26507057	26539739	Hnrnpul1	0	-	MACS_peak_13942|MACS_peak_13942|M	9756|9775|19898|19917
chr7	28043779	28067169	Numbl	0	+	MACS_peak_13955|MACS_peak_13958	18676|-15283
chr7	28018031	28042968	Adck4	0	+	MACS_peak_13955	-7072
chr7	27992188	28013616	Itpkc	0	-	MACS_peak_13955	11487
chr7	28090159	28118667	Ltbp4	0	-	MACS_peak_13984|MACS_peak_13984|M	-4847|-8811|-4220|-8184
chr7	28127151	28141027	Shkbp1	0	-	MACS_peak_13984|MACS_peak_13986	-27207|-26580
chr7	28308279	28316193	Hipk4	0	+	MACS_peak_13999	-16154
chr7	28345494	28367117	2310022A1	0	+	MACS_peak_13999|MACS_peak_13999|M	21061|13880|-4061|-11242
chr7	28317036	28338131	Pld3	0	-	MACS_peak_13999|MACS_peak_14002	-13698|11424
chr7	28376578	28424472	Akt2	0	+	MACS_peak_14002|MACS_peak_14003	27023|-27371
chr7	28438942	28441226	Ttc9b	0	+	MACS_peak_14003	20693
chr7	28964501	28972313	Dyrk1b	0	+	MACS_peak_14009|MACS_peak_14009|M	-5650|-5639|-5870|-5859|-6480|-6469|-16883|-16872
chr7	28954766	28964288	Fbl	0	+	MACS_peak_14009|MACS_peak_14010|M	-15385|-15605|-16215|-26618
chr7	29078573	29086804	Dll3	0	-	MACS_peak_14024|MACS_peak_14025|M	15483|16000|27836
chr7	29099914	29123738	Supt5h	0	-	MACS_peak_14024|MACS_peak_14025|M	-21451|-20934|-9098
chr7	29090844	29097065	Timm50	0	-	MACS_peak_14024|MACS_peak_14025|M	5222|5739|17575
chr7	29069670	29076153	BC089491	0	-	MACS_peak_14024|MACS_peak_14025	26134|26651
chr7	29135707	29137717	Rps16	0	+	MACS_peak_14032	21067
chr7	29136558	29136651	LOC10030	0	+	MACS_peak_14032	21918
chr7	29144622	29157681	Plekhg2	0	-	MACS_peak_14035|MACS_peak_14035	28072|28072
chr7	29161802	29164247	Zfp36	0	-	MACS_peak_14035|MACS_peak_14036	21506|27304
chr7	29178014	29184402	Paf1	0	+	MACS_peak_14035|MACS_peak_14036	-7739|-13537
chr7	29170265	29177709	Med29	0	-	MACS_peak_14035|MACS_peak_14036	8044|13842
chr7	29184542	29221210	Samd4b	0	-	MACS_peak_14036|MACS_peak_14037	-29659|-12558
chr7	29225955	29231914	Gmfg	0	+	MACS_peak_14037|MACS_peak_14037	17303|13813
chr7	29237256	29252567	Lrfn1	0	+	MACS_peak_14037|MACS_peak_14037	28604|28351
chr7	29343837	29383203	Pak4	0	-	MACS_peak_14038	-91
chr7	29666674	29678604	Capn12	0	+	MACS_peak_14041|MACS_peak_14044	-20288|-29877
chr7	29937529	29941229	Fam98c	0	-	MACS_peak_14046	27144
chr7	29959205	29965692	Psmd8	0	-	MACS_peak_14046	2681
chr7	29955228	29958952	Ggn	0	+	MACS_peak_14046|MACS_peak_14046	-13145|-12965
chr7	29943847	29953666	Spred3	0	-	MACS_peak_14046	14707
chr7	30089023	30102605	Dpf1	0	+	MACS_peak_14050	-27411
chr7	30601090	30617061	Zfp790	0	+	MACS_peak_14056|MACS_peak_14056	-9680|-9680
chr7	30628954	30638667	BC027344	0	-	MACS_peak_14056	-27897
chr7	30821377	30836415	Zfp14	0	-	MACS_peak_14058|MACS_peak_14058	14911|14911
chr7	30880094	30892633	Zfp260	0	+	MACS_peak_14058	28768
chr7	30862355	30875529	Zfp566	0	-	MACS_peak_14058	-24203
chr7	30841052	30857842	Zfp82	0	-	MACS_peak_14058	-6516
chr7	30954940	30965228	Gm5113	0	+	MACS_peak_14060	7894
chr7	30969189	30971049	Cox7a1	0	+	MACS_peak_14060	22143
chr7	30946286	30954746	Zfp146	0	-	MACS_peak_14060	-7700
chr7	3128145	3144993	Speer9-ps1	0	+	MACS_peak_13765	16713
chr7	31278676	31298421	Prodh2	0	+	MACS_peak_14061	-28881
chr7	31307244	31320028	Arhgap33	0	-	MACS_peak_14061|MACS_peak_14062|M	-12471|-11256|-11006
chr7	31324152	31337291	BC053749	0	-	MACS_peak_14061|MACS_peak_14062|M	-29734|-28519|-28269
chr7	31338320	31340458	Hspb6	0	+	MACS_peak_14062|MACS_peak_14063	29548|29298
chr7	31883743	31900309	Hpn	0	-	MACS_peak_14066|MACS_peak_14066|M	15062|15062|24183|24183
chr7	31901542	31911964	Scn1b	0	-	MACS_peak_14066|MACS_peak_14069	3407|12528
chr7	31915145	31936069	Gramd1a	0	-	MACS_peak_14066|MACS_peak_14069	-20698|-11577
chr7	3289038	3299350	Myadm	0	+	MACS_peak_13766|MACS_peak_13766|M	-1621|-103|2369|-104
chr7	3303657	3331005	Prkcc	0	+	MACS_peak_13766	12998
chr7	35424035	35437860	Kctd15	0	-	MACS_peak_14093|MACS_peak_14096	-15585|-2796
chr7	35697425	35829727	Pepd	0	+	MACS_peak_14097	-9026
chr7	3581586	3594084	Prpf31	0	+	MACS_peak_13768|MACS_peak_13768	263|263
chr7	3571925	3581486	Tfpt	0	-	MACS_peak_13768	-163
chr7	3568974	3571763	Ndufa3	0	+	MACS_peak_13768	-12349
chr7	3596870	3612710	Cnot3	0	+	MACS_peak_13768	15547
chr7	3561416	3567735	Oscar	0	-	MACS_peak_13768	13588
chr7	35831440	35841585	Cebpg	0	-	MACS_peak_14098	-7377
chr7	36182111	36223703	Ccdc123	0	+	MACS_peak_14101	5226
chr7	36177170	36181786	C230052I1	0	-	MACS_peak_14101	-4901
chr7	3645211	3652637	Tsen34	0	+	MACS_peak_13769|MACS_peak_13769|M	1767|1767|2665
chr7	3664106	3671984	Lilrb3	0	-	MACS_peak_13769	-28540
chr7	3611708	3617442	Leng1	0	-	MACS_peak_13769	26002
chr7	3655642	3658499	Rps9	0	+	MACS_peak_13769	12198
chr7	3629390	3645127	Mboat7	0	-	MACS_peak_13769	-1683
chr7	3617355	3629155	Tmc4	0	-	MACS_peak_13769	14289
chr7	36557368	36588008	Zfp507	0	-	MACS_peak_14103	-1504

chr7	36587610	36623093	E130304I0	0	+	MACS_peak_14103	1106
chr7	4102861	4116301	Cdc42ep5	0	-	MACS_peak_13773|MACS_peak_13774	10752|12267
chr7	4099784	4101474	Leng9	0	-	MACS_peak_13773|MACS_peak_13774	25579|27094
chr7	4416343	4430844	Eps8l1	0	+	MACS_peak_13775	7824
chr7	4377266	4397259	Rdh13	0	-	MACS_peak_13775	11260
chr7	4418846	4422486	D630041G	0	-	MACS_peak_13775	-13967
chr7	4579641	4582084	Tmem86b	0	-	MACS_peak_13776	8608
chr7	4583096	4610552	Ppp6r1	0	-	MACS_peak_13776	-19860
chr7	46843887	46847070	Gm2058	0	+	MACS_peak_14107|MACS_peak_14108	24487|20032
chr7	4724661	4729743	Il11	0	-	MACS_peak_13779|MACS_peak_13781|M	-2180|-1732|-86
chr7	4744566	4746149	Rpl28	0	+	MACS_peak_13779|MACS_peak_13781|M	17003|16555|14909
chr7	4736386	4741162	2210411K1	0	-	MACS_peak_13779|MACS_peak_13781|M	-13599|-13151|-11505
chr7	4734541	4735940	Tmem190	0	+	MACS_peak_13779|MACS_peak_13781|M	6978|6530|4884
chr7	4704830	4722872	Fam71e2	0	-	MACS_peak_13779|MACS_peak_13781|M	4691|5139|6785
chr7	4703395	4704696	Cox6b2	0	-	MACS_peak_13779|MACS_peak_13779|M	22867|23059|23315|23507|24961|25153
chr7	5061388	5068513	Rfpl4	0	-	MACS_peak_13785|MACS_peak_13785	-24010|-24010
chr7	5031906	5049778	Epn1	0	+	MACS_peak_13785	-12597
chr7	51029986	51034630	Klk11	0	+	MACS_peak_14124|MACS_peak_14124	15574|17286
chr7	51024469	51028851	Klk12	0	+	MACS_peak_14124	10057
chr7	51036423	51040780	Klk10	0	+	MACS_peak_14124	22011
chr7	51827814	51842216	Napsa	0	+	MACS_peak_14130|MACS_peak_14134	21614|18569
chr7	51805002	51809293	Nr1h2	0	-	MACS_peak_14130|MACS_peak_14134	-3093|-48
chr7	51788113	51804185	Pold1	0	-	MACS_peak_14130|MACS_peak_14134	2015|5060
chr7	51781364	51787441	Spib	0	-	MACS_peak_14130|MACS_peak_14134	18759|21804
chr7	51757068	51780039	Mybpc2	0	-	MACS_peak_14130|MACS_peak_14134	26161|29206
chr7	52096145	52104449	Tbc1d17	0	-	MACS_peak_14142	-6387
chr7	52071739	52096171	Nup62-il4i1	0	+	MACS_peak_14142	-26323
chr7	52091657	52096178	Il4i1	0	+	MACS_peak_14142	-6405
chr7	52112550	52118299	Pnkp	0	+	MACS_peak_14142	14488
chr7	52104596	52110780	Akt1s1	0	+	MACS_peak_14142	6534
chr7	52067625	52071028	Atf5	0	-	MACS_peak_14142|MACS_peak_14142	27034|26034
chr7	52071789	52086176	Nup62	0	+	MACS_peak_14142	-26273
chr7	52118437	52125158	Ptov1	0	-	MACS_peak_14142	-27096
chr7	52105068	52105141	Mir707	0	+	MACS_peak_14142	7006
chr7	52258319	52271619	Scaf1	0	-	MACS_peak_14145	1832
chr7	52273376	52277014	Rras	0	+	MACS_peak_14145	-75
chr7	52253029	52258218	Irf3	0	+	MACS_peak_14145	-20422
chr7	52246592	52252949	Bcl2l12	0	-	MACS_peak_14145	20502
chr7	52317798	52333873	Nosip	0	+	MACS_peak_14150|MACS_peak_14150	12503|12503
chr7	52308976	52317022	Prrg2	0	-	MACS_peak_14150	-11727
chr7	52283076	52308251	Prr12	0	-	MACS_peak_14150	-2956
chr7	52381145	52384105	Rpl13a	0	-	MACS_peak_14163	20977
chr7	52386558	52391802	Flt3l	0	-	MACS_peak_14163	13280
chr7	52377757	52379759	Rps11	0	-	MACS_peak_14163	25323
chr7	52409767	52415434	Pih1d1	0	+	MACS_peak_14163	4685
chr7	52397209	52409908	Aldh16a1	0	-	MACS_peak_14163	-4826
chr7	52419290	52431508	Slc17a7	0	+	MACS_peak_14163	14208
chr7	52382752	52382833	Snord32a	0	-	MACS_peak_14163	22249
chr7	52381716	52381805	Snord35a	0	-	MACS_peak_14163	23277
chr7	52378395	52378481	Snord35b	0	-	MACS_peak_14163	26601
chr7	52382233	52382315	Snord33	0	-	MACS_peak_14163	22767
chr7	52381972	52382038	Snord34	0	-	MACS_peak_14163	23044
chr7	52377126	52377191	Mir150	0	+	MACS_peak_14163	-27956
chr7	52590638	52594342	Hrc	0	+	MACS_peak_14166	20234
chr7	52558524	52589150	Trpm4	0	-	MACS_peak_14166	-18746
chr7	52676315	52677224	Lhb	0	+	MACS_peak_14168	-13414
chr7	52713313	52715256	Ftl1	0	-	MACS_peak_14168	-25527
chr7	52661329	52666752	Kcna7	0	+	MACS_peak_14168	-28400
chr7	52677267	52689834	Ruvbl2	0	-	MACS_peak_14168	-105
chr7	52690208	52711987	Gys1	0	+	MACS_peak_14168	479
chr7	52669064	52672549	Ntf5	0	+	MACS_peak_14168	-20665
chr7	52769075	52777973	Tulp2	0	+	MACS_peak_14175|MACS_peak_14175	-12399|-12399
chr7	52748043	52765778	Nucb1	0	-	MACS_peak_14175|MACS_peak_14175	15696|15696
chr7	52778289	52781638	Ppp1r15a	0	-	MACS_peak_14175	-164
chr7	52810297	52822690	Hsd17b14	0	+	MACS_peak_14175	28823
chr7	52781699	52809599	Plekha4	0	+	MACS_peak_14175	225
chr7	52877180	52882612	Izumo1	0	+	MACS_peak_14176|MACS_peak_14177	7671|6561
chr7	52872975	52876427	Fut1	0	+	MACS_peak_14176|MACS_peak_14177	3466|2356
chr7	52869259	52870860	Fgf21	0	-	MACS_peak_14176|MACS_peak_14177	-1351|-241
chr7	52882906	52894462	Rasip1	0	+	MACS_peak_14176|MACS_peak_14177	13397|12287
chr7	52895346	52901138	Mamstr	0	+	MACS_peak_14176|MACS_peak_14177	25837|24727
chr7	52976589	52984862	Fam83e	0	+	MACS_peak_14182	7712
chr7	52939783	52949924	Ntn5	0	+	MACS_peak_14182	-29094
chr7	52964834	52973372	Sphk2	0	-	MACS_peak_14182|MACS_peak_14182|M	-4495|-1700|-4495
chr7	52973440	52976205	Rpl18	0	+	MACS_peak_14182	4563
chr7	52955336	52960031	Car11	0	+	MACS_peak_14182	-13541
chr7	52960616	52965573	Dbp	0	+	MACS_peak_14182	-8261
chr7	52980477	52981186	Spaca4	0	-	MACS_peak_14182	-12309
chr7	53698528	53895177	Sergef	0	-	MACS_peak_14183	-7105
chr7	54144396	54175300	Tsg101	0	-	MACS_peak_14185	6394
chr7	56886868	56892205	Dbx1	0	-	MACS_peak_14209	-27603
chr7	63495140	63791887	Oca2	0	+	MACS_peak_14237|MACS_peak_14238|M	26249|23853|11913|9969
chr7	71521426	71537122	Mphosph10	0	-	MACS_peak_14252	-6491
chr7	71537656	71557005	Mcee	0	+	MACS_peak_14252	7025
chr7	71491644	71518981	Fan1	0	-	MACS_peak_14252	11650
chr7	72838302	72846799	Tm2d3	0	+	MACS_peak_14258|MACS_peak_14258|M	-1816|-1816|-5707|-5707
chr7	73205221	73219472	Snrpa1	0	+	MACS_peak_14260	-9811
chr7	73224534	73234291	H47	0	+	MACS_peak_14260	9502
chr7	73254400	73318684	Chsy1	0	+	MACS_peak_14261	-15969
chr7	73834774	73862141	Lins2	0	+	MACS_peak_14266|MACS_peak_14266|M	29723|29723|29723|-18490|-18490|-18490
chr7	73789456	73834447	Asb7	0	-	MACS_peak_14266|MACS_peak_14266|M	-29396|-29396|18817|18817
chr7	74792295	74871462	Ttc23	0	+	MACS_peak_14271|MACS_peak_14271|M	29355|29355|29355|29355|-22707|-22707|-22707|-22707
chr7	74739284	74790122	Lrrc28	0	-	MACS_peak_14271|MACS_peak_14271|M	-27182|-27182|-27182|-27182|24880|24880|24880|24880
chr7	75097142	75378553	Igf1r	0	+	MACS_peak_14283	-346
chr7	75881879	75894124	Arrdc4	0	-	MACS_peak_14305|MACS_peak_14305	-10925|-10925
chr7	77496835	77505479	Nr2f2	0	-	MACS_peak_14312|MACS_peak_14312|M	-8354|-14507|947|-5206
chr7	86059120	86065282	Isg20	0	+	MACS_peak_14347|MACS_peak_14347|M	18250|17780|11656|11186|6440|5970|4731|4261
chr7	86040812	86053719	Aen	0	+	MACS_peak_14347|MACS_peak_14347|M	-58|-58|-6652|-6652|-11868|-11868|-13577|-13577
chr7	86033162	86033258	Mir7-2	0	+	MACS_peak_14347|MACS_peak_14348|M	-7708|-14302|-19518|-21227
chr7	86519756	86531913	Rlbp1	0	-	MACS_peak_14360|MACS_peak_14360	-28921|-28921
chr7	86594268	86611159	Polg	0	-	MACS_peak_14362	-92
chr7	86866049	86877662	Plin1	0	-	MACS_peak_14363|MACS_peak_14363	-25181|-25181
chr7	86842984	86859072	Kif7	0	-	MACS_peak_14363	-6591
chr7	86937127	86938476	Mesp1	0	-	MACS_peak_14364	-3365
chr7	86955612	86958317	Mesp2	0	+	MACS_peak_14364	20501
chr7	87331726	87371410	Sema4b	0	+	MACS_peak_14365|MACS_peak_14366	-11778|-26040
chr7	87372045	87377502	Cib1	0	-	MACS_peak_14366|MACS_peak_14367|M	-19736|-13637|-12388
chr7	87377778	87386306	D330012F2	0	+	MACS_peak_14366|MACS_peak_14367|M	20012|13913|12664
chr7	87391261	87405707	Ttll13	0	+	MACS_peak_14367|MACS_peak_14368	27396|26147
chr7	87406100	87410264	Ngrn	0	+	MACS_peak_14373|MACS_peak_14373	-24628|-24628
chr7	87439350	87461145	Prc1	0	+	MACS_peak_14373	8622
chr7	87414540	87436465	Vps33b	0	+	MACS_peak_14373	-16188
chr7	87488022	87490983	Hddc3	0	+	MACS_peak_14374|MACS_peak_14376|M	17515|6743|-15178|-16562|-19842|-20420|-20875|-24618|-25716|-27648
chr7	87470179	87485105	Unc45a	0	-	MACS_peak_14374|MACS_peak_14376|M	-14598|-3826|18095|19479|22759|23337|23792|27535|28633
chr7	87461501	87469340	Rccd1	0	-	MACS_peak_14374|MACS_peak_14376	1167|11939
chr7	87522643	87532832	Fes	0	-	MACS_peak_14379|MACS_peak_14380|M	-29632|-28248|-24968|-24390|-23935|-20192|-19094|-17162
chr7	87505335	87516012	Man2a2	0	-	MACS_peak_14379|MACS_peak_14380|M	-12812|-11428|-8148|-7570|-7115|-3372|-2274|-342
chr7	87731517	87833763	Crtc3	0	-	MACS_peak_14391	23896
chr7	88006993	88021399	Zscan2	0	+	MACS_peak_14396|MACS_peak_14397	-13358|-13578
chr7	88047114	88049957	Nmb	0	-	MACS_peak_14396|MACS_peak_14397	-29606|-29386
chr7	88035609	88046155	Wdr73	0	-	MACS_peak_14396|MACS_peak_14396|M	-25804|-25804|-25584|-25584
chr7	88716177	88741722	Whamm	0	+	MACS_peak_14400	-20515

chr7	88679239	88711867	Fsd2	0	-	MACS_peak_14400	24825
chr7	89111547	89137185	Bnc1	0	-	MACS_peak_14403	-13828
chr7	89484203	89762958	Adamtsl3	0	+	MACS_peak_14405	-1545
chr7	91733669	91754452	Fah	0	-	MACS_peak_14436	27996
chr7	92002080	92005827	Olfr291	0	+	MACS_peak_14437	19056
chr7	96552875	96558620	Fzd4	0	+	MACS_peak_14441|MACS_peak_14442|M	-3080|-3944|-4945|-5690|-7102|-7727
chr8	106774831	106788836	Cklf	0	+	MACS_peak_15468|MACS_peak_15468|M	23012|22983|12169|12140
chr8	106750590	106772458	Tk2	0	-	MACS_peak_15468|MACS_peak_15469	-20639|-9796
chr8	106941573	106966947	Dync1li2	0	-	MACS_peak_15470|MACS_peak_15471|M	-23222|-14938|-542
chr8	106878415	106919596	Cmtm4	0	-	MACS_peak_15470	24129
chr8	107064706	107074243	Car7	0	+	MACS_peak_15473|MACS_peak_15474	24116|6248
chr8	107034929	107058537	Nae1	0	-	MACS_peak_15473|MACS_peak_15474	-17947|-79
chr8	106970618	107033787	Ccdc79	0	-	MACS_peak_15473|MACS_peak_15474	6803|24671
chr8	107872157	107893781	Slc9a5	0	+	MACS_peak_15481	-20431
chr8	107899280	107906762	Plekhg4	0	+	MACS_peak_15481	6692
chr8	107853061	107871870	Fhod1	0	-	MACS_peak_15481	20718
chr8	108129128	108146118	Fam65a	0	+	MACS_peak_15482|MACS_peak_15484	-11725|-15259
chr8	108160437	108206822	Ctcf	0	+	MACS_peak_15482|MACS_peak_15484	19584|16050
chr8	108139365	108139472	Mir1966	0	+	MACS_peak_15482|MACS_peak_15484	-1488|-5022
chr8	108240012	108282507	Gfod2	0	-	MACS_peak_15485	21326
chr8	108379002	108380850	Thap11	0	+	MACS_peak_15486	-29477
chr8	108384533	108404301	Nutf2	0	+	MACS_peak_15486|MACS_peak_15487	-23946|-26716
chr8	108424373	108455702	Pskh1	0	+	MACS_peak_15486|MACS_peak_15487	15894|13124
chr8	108417469	108418915	Nrn1l	0	+	MACS_peak_15486|MACS_peak_15487	8990|6220
chr8	108404850	108417107	Edc4	0	+	MACS_peak_15486|MACS_peak_15487	-3629|-6399
chr8	108384559	108403236	Gm10333	0	+	MACS_peak_15486|MACS_peak_15487	-23920|-26690
chr8	108463456	108467302	Lcat	0	-	MACS_peak_15489|MACS_peak_15490|M	3373|4188|8506
chr8	108467492	108489939	Slc12a4	0	-	MACS_peak_15489|MACS_peak_15490|M	-19264|-18449|-14131
chr8	108459627	108462292	Psmb10	0	-	MACS_peak_15489|MACS_peak_15490|M	8383|9198|13516
chr8	108455893	108457762	Ctrl	0	-	MACS_peak_15489|MACS_peak_15490|M	12913|13728|18046
chr8	108497419	108503319	Dpep3	0	-	MACS_peak_15492	-27511
chr8	108535406	108577719	Dus2l	0	+	MACS_peak_15494	-4412
chr8	108533516	108535386	Ddx28	0	-	MACS_peak_15494	4432
chr8	108508956	108520323	Dpep2	0	-	MACS_peak_15494	19495
chr8	108674298	108688615	Pla2g15	0	+	MACS_peak_15495	-82
chr8	108655082	108660874	Esrp2	0	-	MACS_peak_15495	13506
chr8	108692774	108722604	Slc7a6	0	+	MACS_peak_15495	18394
chr8	109580776	109584826	Nip7	0	+	MACS_peak_15510|MACS_peak_15510|M	25475|25475|25475|14604|14604|14604
chr8	109570194	109572514	Pdf	0	-	MACS_peak_15510|MACS_peak_15511	-17213|-6342
chr8	109555225	109569656	Vps4a	0	+	MACS_peak_15510|MACS_peak_15511	-76|-10947
chr8	109572608	109580637	Cog8	0	-	MACS_peak_15510|MACS_peak_15511	-25336|-14465
chr8	109585383	109589544	Tmed6	0	-	MACS_peak_15511	-23372
chr8	10986963	11008430	Irs2	0	-	MACS_peak_14921	340
chr8	112363211	112363275	Snord71	0	+	MACS_peak_15522	20341
chr8	112476808	112486105	Marveld3	0	-	MACS_peak_15523|MACS_peak_15523	-28182|-28182
chr8	113245383	113265300	Mtss1l	0	+	MACS_peak_15524	23543
chr8	113265729	113329803	Il34	0	-	MACS_peak_15534|MACS_peak_15534|M	6111|6111|7835|7835|8701|8701|10356|10356|11726|11726|17545|17545|19675|19675|20267|20267|25460|25460|29716|29716
chr8	113362434	113362498	Snord111	0	-	MACS_peak_15534|MACS_peak_15535|M	-26584|-24860|-23994|-22339|-20969|-15150|-13020|-12428|-7235|-2979|-318|11306|12792|28044
chr8	113334391	113370703	Sf3b3	0	-	MACS_peak_15538|MACS_peak_15539|M	-29174|-23355|-21225|-20633|-15440|-11184|-8523|3101|4587|19839
chr8	113370923	113406134	Cog4	0	+	MACS_peak_15538|MACS_peak_15539|M	29394|23575|21445|20853|15660|11404|8743|-2881|-4367|-19619
chr8	113406367	113426359	Fuk	0	-	MACS_peak_15553|MACS_peak_15553|M	-22780|-22780|-19672|-19672|-2988|-2988
chr8	113443764	113496397	St3gal2	0	+	MACS_peak_15558	20393
chr8	113498890	113521723	Ddx19a	0	-	MACS_peak_15562	-28222
chr8	113527085	113555651	Ddx19b	0	-	MACS_peak_15563|MACS_peak_15563|M	5240|9221|9221|8474|12455|12455|8917|12898|12898|10981|14962|14962|11568|15549|15549|11918|15899|15899|13188|17169|171
chr8	113580238	113581499	Exosc6	0	+	MACS_peak_15563|MACS_peak_15564|M	19347|16113|15670|13606|13019|12669|11399|10741|9319|6538|2393|2126|1032
chr8	113557866	113580770	Aars	0	+	MACS_peak_15563|MACS_peak_15564|M	-3025|-6259|-6702|-8766|-9353|-9703|-10973|-11631|-13053|-15834|-19979|-20246|-21340
chr8	113593396	113605608	Clec18a	0	-	MACS_peak_15573|MACS_peak_15574|M	-27763|-27496|-26402
chr8	113618644	113659044	4930402E1	0	+	MACS_peak_15576|MACS_peak_15577	-22020|-23326
chr8	11497505	11513284	Carkd	0	+	MACS_peak_14982|MACS_peak_14982	-7971|-7909
chr8	11453976	11478499	Rab20	0	-	MACS_peak_14982	26977
chr8	118227152	118230794	Maf	0	-	MACS_peak_15605	-22900
chr8	124320207	124353469	Klhdc4	0	-	MACS_peak_15648	-146
chr8	124431732	124442328	BC048644	0	+	MACS_peak_15649|MACS_peak_15650|M	25688|24146|20959|11339|461
chr8	124405045	124431586	Slc7a5	0	-	MACS_peak_15649|MACS_peak_15650|M	-25542|-24000|-20813|-11193|-315
chr8	124474432	124553158	Banp	0	+	MACS_peak_15654|MACS_peak_15654	-23980|-23980
chr8	124440043	124468812	Car5a	0	-	MACS_peak_15654	29600
chr8	124806040	124861147	Zfpm1	0	+	MACS_peak_15661	-23284
chr8	124990046	124999964	Rnf166	0	-	MACS_peak_15663	840
chr8	124978105	124984592	Snai3	0	-	MACS_peak_15663	16212
chr8	125000042	125006992	Ctu2	0	+	MACS_peak_15663	-762
chr8	124969167	124975222	9330133O	0	-	MACS_peak_15663	25582
chr8	126547372	126572404	Urb2	0	+	MACS_peak_15669	-13220
chr8	126520215	126545209	Taf5l	0	-	MACS_peak_15669	15383
chr8	126546540	126546622	Mir1967	0	+	MACS_peak_15669	-14052
chr8	127044666	127075905	Cog2	0	+	MACS_peak_15673|MACS_peak_15674	-17077|-20269
chr8	127080486	127093607	Agt	0	-	MACS_peak_15674	-28672
chr8	127941962	128016610	Sipa1l2	0	-	MACS_peak_15675	-24084
chr8	128946400	128949334	1810063B(	0	+	MACS_peak_15676	-14274
chr8	131209553	131257479	Itgb1	0	+	MACS_peak_15679	-21467
chr8	13105720	13147939	Cul4a	0	+	MACS_peak_14986|MACS_peak_14987	-18192|-28019
chr8	13077524	13105343	Pcid2	0	-	MACS_peak_14986|MACS_peak_14987	18569|28396
chr8	13159134	13175338	Lamp1	0	+	MACS_peak_14987	25395
chr8	13255962	13288126	Dcun1d2	0	-	MACS_peak_14991|MACS_peak_14991|M	25762|25762|25762|25762
chr8	13339673	13377702	Tfdp1	0	+	MACS_peak_14991	25785
chr8	13288012	13322924	Tmco3	0	+	MACS_peak_14991	-25876
chr8	13465373	13494535	Gas6	0	-	MACS_peak_14992|MACS_peak_14994	-26273|-17434
chr8	13705888	13743066	4932443I1	0	+	MACS_peak_15001|MACS_peak_15002|M	28396|-5507|-8691|-9395
chr8	13567217	13677587	Rasa3	0	-	MACS_peak_15001	-95
chr8	13907805	13940521	Fbxo25	0	+	MACS_peak_15005	-27397
chr8	14911716	15001085	Arhgef10	0	+	MACS_peak_15006|MACS_peak_15006|M	22686|22686|16820|16820|13195|13195|11820|11820|11332|11332|-28566|-28566
chr8	14888535	14901719	Cln8	0	+	MACS_peak_15006|MACS_peak_15007|M	-495|-6361|-9986|-11361|-11849
chr8	19863863	19893010	6820431F2	0	-	MACS_peak_15020|MACS_peak_15021|M	-19369|-14176|-27157|-14154
chr8	19981359	20020392	2610005L(	0	-	MACS_peak_15022|MACS_peak_15023	-27271|-14268
chr8	23104819	23170546	Atp7b	0	-	MACS_peak_15024|MACS_peak_15025	1937|5394
chr8	23171191	23178761	Alg11	0	+	MACS_peak_15024|MACS_peak_15025	-1292|-4749
chr8	23687572	23704230	Vdac3	0	-	MACS_peak_15027	-24831
chr8	23738590	23763909	Polb	0	-	MACS_peak_15028	-13041
chr8	23734514	23738018	Dkk4	0	+	MACS_peak_15028	-16354
chr8	23744856	23747281	A930013F1	0	-	MACS_peak_15028	3587
chr8	25946614	26059276	Adam32	0	-	MACS_peak_15031|MACS_peak_15032|M	14170|16162|29720
chr8	26060082	26127394	Adam9	0	-	MACS_peak_15038|MACS_peak_15041	-25631|-21781
chr8	26127682	26133731	Tm2d2	0	+	MACS_peak_15038|MACS_peak_15041	25919|22069
chr8	26750755	26830139	Whsc1l1	0	+	MACS_peak_15064	-25815
chr8	26830519	26835359	Ppapdc1b	0	+	MACS_peak_15066|MACS_peak_15067|M	28997|9308|-4421
chr8	26835795	26864752	Ddhd2	0	-	MACS_peak_15068	-29812
chr8	27091075	27104593	4930444A(	0	-	MACS_peak_15070	-21223
chr8	27054930	27087216	Hgsnat	0	-	MACS_peak_15070	-3846
chr8	28153026	28160713	Prosc	0	+	MACS_peak_15073|MACS_peak_15073|M	12945|12945|12945
chr8	28134330	28149896	Erlin2	0	+	MACS_peak_15073	-5751
chr8	28234303	28239104	Brf2	0	-	MACS_peak_15077|MACS_peak_15083|M	-18896|-13285|-11215|-9843|-9439|-160|13171|25997
chr8	28196312	28233907	Gpr124	0	+	MACS_peak_15077|MACS_peak_15083	-23896|-29507
chr8	28249246	28285118	Rab11fip1	0	-	MACS_peak_15092|MACS_peak_15092|M	-20017|-20017|-339|-339
chr8	28307930	28313019	Got1l1	0	-	MACS_peak_15093	-28240
chr8	28370798	28386128	Eif4ebp1	0	+	MACS_peak_15094|MACS_peak_15096	-13092|-21272
chr8	28406516	28407672	Gm9731	0	+	MACS_peak_15094|MACS_peak_15096	22626|14446
chr8	32260787	32275175	BC019943	0	+	MACS_peak_15099|MACS_peak_15099|M	-584|-584|-3290|-3290|-8355|-8355
chr8	32269939	32279196	Mak16	0	-	MACS_peak_15099|MACS_peak_15100|M	-17825|-15119|-10054
chr8	32297849	32371955	Fut10	0	+	MACS_peak_15102|MACS_peak_15102	28707|28661
chr8	34496796	34527941	Purg	0	+	MACS_peak_15118|MACS_peak_15118|M	-307|-307|-711|-711
chr8	34344844	34495999	Wrn	0	-	MACS_peak_15118|MACS_peak_15118|M	1104|1104|1508|1508
chr8	35178083	35188239	Mboat4	0	+	MACS_peak_15132|MACS_peak_15133	-21677|-23948
chr8	35153476	35171565	Dctn6	0	-	MACS_peak_15132	28195

chr8	35217616	35233901	Tmem66	0	+	MACS_peak_15132|MACS_peak_15133|M	17856|15585|-6721|-13970
chr8	35198625	35209793	Leprotl1	0	-	MACS_peak_15132|MACS_peak_15133|M	-10033|-7762|14544|21793
chr8	35870663	35882948	Dusp4	0	+	MACS_peak_15139	-9654
chr8	3623373	3625545	Pcp2	0	-	MACS_peak_14901|MACS_peak_14901|M	-14939|-14443|-14676|-14088|-13592|-13825|-11893|-11397|-11630|-6945|-6449|-6682
chr8	3587449	3609074	2310057J1	0	-	MACS_peak_14901|MACS_peak_14901|M	-23157|-23157|-24008|-24008|-26203|-26203
chr8	3631159	3643644	Stxbp2	0	+	MACS_peak_14901|MACS_peak_14902|M	20553|19702|17507|12559
chr8	3610089	3621296	Xab2	0	-	MACS_peak_14901|MACS_peak_14902|M	-10690|-9839|-7644|-2696
chr8	37157881	37210841	D8Ertd82e	0	+	MACS_peak_15145	-8141
chr8	37279117	37312570	Lonrf1	0	-	MACS_peak_15147|MACS_peak_15148|M	-26821|-26563|-20336
chr8	4226826	4237469	Lrrc8e	0	+	MACS_peak_14909|MACS_peak_14910|M	19746|-16538|-26359
chr8	4209542	4217312	BC068157	0	-	MACS_peak_14909|MACS_peak_14910	-10232|26052
chr8	42102821	42109570	B430010I2	0	+	MACS_peak_15156	16661
chr8	42076265	42140148	Mtus1	0	-	MACS_peak_15157|MACS_peak_15157|M	-4744|8139|28673
chr8	42425996	42460051	Asah1	0	-	MACS_peak_15163|MACS_peak_15164	-27693|-77
chr8	4238739	4247896	Map2k7	0	+	MACS_peak_14910|MACS_peak_14910|M	-4625|-4625|-4625|-14446|-14446|-14446
chr8	4253101	4256220	Snapc2	0	+	MACS_peak_14910|MACS_peak_14911	9737|-84
chr8	4257645	4259274	Ctxn1	0	-	MACS_peak_14910|MACS_peak_14911|M	-15910|-6089|27599
chr8	4259730	4275905	Timm44	0	-	MACS_peak_14911|MACS_peak_14912	-22720|10968
chr8	4349587	4360019	Ccl25	0	+	MACS_peak_14913|MACS_peak_14913	8479|-15899
chr8	4284781	4325100	Elavl1	0	-	MACS_peak_14913	16008
chr8	46035561	46137611	Fat1	0	+	MACS_peak_15173	-2978
chr8	46146055	46150772	AY512931	0	-	MACS_peak_15176	-28351
chr8	47637422	47664938	Mlf1ip	0	+	MACS_peak_15177	16995
chr8	47825098	47932812	Irf2	0	+	MACS_peak_15178	-110
chr8	48575481	48618824	D030016E:	0	-	MACS_peak_15184	-18642
chr8	48618998	48638191	Rwdd4a	0	+	MACS_peak_15184	18816
chr8	48752531	48760513	Ing2	0	-	MACS_peak_15185	-568
chr8	54597079	54608776	Aga	0	+	MACS_peak_15186	-7220
chr8	63389185	63433596	Clcn3	0	-	MACS_peak_15198|MACS_peak_15199|M	-17407|10362|10362|10362|23381|23381|23381
chr8	63472016	63609031	Nek1	0	+	MACS_peak_15199|MACS_peak_15200	-454|-13473
chr8	67196941	67212375	Sc4mol	0	-	MACS_peak_15223	-5812
chr8	69000244	69010406	1810029B:	0	-	MACS_peak_15224|MACS_peak_15225|M	-11626|-10669|-4990
chr8	71612634	71637614	Atp6v1b2	0	+	MACS_peak_15227|MACS_peak_15229	-13467|-21209
chr8	71561606	71613121	Slc18a1	0	-	MACS_peak_15227|MACS_peak_15229	12980|20722
chr8	71732944	71754438	D10627	0	+	MACS_peak_15231	-17359
chr8	72356602	72396191	Pbx4	0	+	MACS_peak_15237	21123
chr8	72346463	72355001	Lpar2	0	+	MACS_peak_15237	10984
chr8	72315061	72331645	Atp13a1	0	+	MACS_peak_15237	-20418
chr8	72332585	72345769	Gmip	0	+	MACS_peak_15237	-2894
chr8	72676676	72691387	Mef2b	0	+	MACS_peak_15240|MACS_peak_15240	-21584|-21584
chr8	72696309	72707562	Tmem161a	0	+	MACS_peak_15240|MACS_peak_15242	-1951|-10733
chr8	72708238	72736180	Slc25a42	0	-	MACS_peak_15242	-29138
chr8	72855420	72877172	Upf1	0	-	MACS_peak_15243|MACS_peak_15243	-9890|-9890
chr8	72839673	72855486	Gdf1	0	+	MACS_peak_15243|MACS_peak_15243	-27609|-13619
chr8	72839673	72855486	Lass1	0	+	MACS_peak_15243	-27609
chr8	73051641	73059227	Fkbp8	0	+	MACS_peak_15244|MACS_peak_15244	28331|28331
chr8	72974126	73000842	Klhl26	0	-	MACS_peak_15244|MACS_peak_15244	22468|22468
chr8	73017054	73027980	Crlf1	0	+	MACS_peak_15244	-6256
chr8	73032164	73034266	Uba52	0	-	MACS_peak_15244	-10956
chr8	73028194	73030638	2810428I1	0	-	MACS_peak_15244	-7328
chr8	73037296	73047079	2810422J0	0	-	MACS_peak_15244	-23769
chr8	73007769	73011257	Tmem59l	0	-	MACS_peak_15244	12053
chr8	73332347	73345322	Il12rb1	0	+	MACS_peak_15249	19497
chr8	73292079	73300611	Pik3r2	0	-	MACS_peak_15249	12239
chr8	73286671	73290562	Ifi30	0	-	MACS_peak_15249	22288
chr8	73282547	73284820	Mpv17l2	0	-	MACS_peak_15249	28030
chr8	73302016	73316332	Mast3	0	-	MACS_peak_15249	-3482
chr8	73300760	73301505	2010320M	0	+	MACS_peak_15249	-12090
chr8	73796612	73884611	Myo9b	0	+	MACS_peak_15253|MACS_peak_15253|M	-17971|-17971|-17971
chr8	73775023	73796489	Haus8	0	-	MACS_peak_15253|MACS_peak_15253	18094|18094
chr8	74121544	74132048	Fam129c	0	+	MACS_peak_15264	-19970
chr8	74116082	74120166	Pgls	0	+	MACS_peak_15264	-25432
chr8	74134922	74148810	Glt25d1	0	+	MACS_peak_15264	-6592
chr8	74150613	74195656	Unc13a	0	-	MACS_peak_15267	20113
chr8	74200281	74212212	Jak3	0	+	MACS_peak_15267|MACS_peak_15267	-15488|-15488
chr8	74213150	74214475	Insl3	0	+	MACS_peak_15267	-2619
chr8	74215617	74225699	B3gnt3	0	-	MACS_peak_15267	-9930
chr8	74474964	74494232	BC049349	0	+	MACS_peak_15268	21902
chr8	74432504	74442750	Zfp882	0	+	MACS_peak_15268	-20558
chr8	74446723	74458529	Zfp617	0	+	MACS_peak_15268	-6339
chr8	74901315	74945373	Eps15l1	0	-	MACS_peak_15286|MACS_peak_15286|M	-24531|-24531|17072|17072
chr8	74967778	74984439	1700030K(	0	+	MACS_peak_15287	5333
chr8	74948081	74967677	Calr3	0	-	MACS_peak_15287|MACS_peak_15287	-5232|3390
chr8	75247186	75263810	Sin3b	0	+	MACS_peak_15288|MACS_peak_15288	-18596|-18596
chr8	75285778	75287784	F2rl3	0	+	MACS_peak_15288	19996
chr8	77517601	77555871	Hmgxb4	0	+	MACS_peak_15303	-6153
chr8	77557584	77594020	Tom1	0	+	MACS_peak_15304|MACS_peak_15304|M	4138|4138|-17908|-17908
chr8	77617516	77624491	Hmox1	0	+	MACS_peak_15306	24121
chr8	85287518	85298025	Zfp330	0	-	MACS_peak_15323	-1178
chr8	86649887	86656416	Podnl1	0	+	MACS_peak_15326	-20414
chr8	86671936	86696496	4930432K:	0	+	MACS_peak_15326|MACS_peak_15326	1635|1635
chr8	86656726	86671652	Cc2d1a	0	-	MACS_peak_15326	-1351
chr8	86770133	86773660	D8Ertd738	0	-	MACS_peak_15327	-12861
chr8	86774475	86781223	Mri1	0	-	MACS_peak_15327	-20424
chr8	86734840	86760941	Zswim4	0	-	MACS_peak_15327	-142
chr8	86732416	86732491	Mir23a	0	+	MACS_peak_15327	-28383
chr8	86732713	86732819	Mir24-2	0	+	MACS_peak_15327	-28086
chr8	86732570	86732657	Mir27a	0	+	MACS_peak_15327	-28229
chr8	87213145	87223707	Trmt1	0	+	MACS_peak_15329|MACS_peak_15329|M	-1616|-1616|-1616|-6014|-6014|-6014
chr8	87225355	87228615	Lyl1	0	+	MACS_peak_15329|MACS_peak_15330	10594|6196
chr8	87185229	87186751	Ier2	0	-	MACS_peak_15329	28010
chr8	87194378	87211761	Nacc1	0	-	MACS_peak_15329|MACS_peak_15330	3000|7398
chr8	87531101	87538230	Best2	0	-	MACS_peak_15333|MACS_peak_15333|M	-18522|-18599|-18599|23891|23814|23814
chr8	87514493	87527263	Hook2	0	+	MACS_peak_15333|MACS_peak_15333	-5215|-5215
chr8	87500810	87502617	Junb	0	-	MACS_peak_15333	17091
chr8	87493546	87498212	Prdx2	0	+	MACS_peak_15333	-26162
chr8	87541829	87549177	Asna1	0	-	MACS_peak_15333|MACS_peak_15334|M	-29469|12944|19754|26474
chr8	87480508	87489910	Rnaseh2a	0	-	MACS_peak_15333	29798
chr8	87561066	87581481	Tnpo2	0	+	MACS_peak_15334|MACS_peak_15334|M	-1055|-1308|-7865|-8118|-14585|-14838
chr8	87550731	87554185	2310036O:	0	+	MACS_peak_15334|MACS_peak_15335|M	-11390|-18200|-24920
chr8	87584017	87591019	Fbxw9	0	+	MACS_peak_15334|MACS_peak_15335|M	21896|15086|8366|-27131
chr8	87595655	87599060	Dhps	0	+	MACS_peak_15335|MACS_peak_15336|M	26724|20004|-15493
chr8	87604861	87606238	BC056474	0	+	MACS_peak_15336|MACS_peak_15337	29210|-6287
chr8	87598933	87604645	Wdr83	0	-	MACS_peak_15336|MACS_peak_15337	-28994|6503
chr8	87607167	87622638	Man2b1	0	+	MACS_peak_15337	-3981
chr8	88016515	88051457	Gpt2	0	+	MACS_peak_15353|MACS_peak_15354|M	-23|-421|-19590|-25433|-26922|-28801
chr8	90856210	90886090	Brd7	0	-	MACS_peak_15370	-25083
chr8	91551142	91568061	Sall1	0	-	MACS_peak_15372|MACS_peak_15373	-12249|-11776
chr8	93593992	93647743	Rbl2	0	+	MACS_peak_15386	16967
chr8	94322423	94325273	Irx3	0	-	MACS_peak_15397	499
chr8	94881694	94885355	Irx5	0	+	MACS_peak_15398	-1947
chr8	94849936	94880019	4933436C:	0	-	MACS_peak_15398	3622
chr8	95351226	95377319	Mmp2	0	+	MACS_peak_15399	-18837
chr8	95379248	95443178	Lpcat2	0	+	MACS_peak_15399	9185
chr8	95425293	95426306	Capns2	0	+	MACS_peak_15406	-12918
chr8	96591853	96622711	Bbs2	0	-	MACS_peak_15432	-16722
chr8	96910399	96919258	Herpud1	0	+	MACS_peak_15439|MACS_peak_15443	-94|-7312
chr8	97056927	97094429	Cpne2	0	+	MACS_peak_15445	-24423
chr8	97447593	97467190	Gpr114	0	+	MACS_peak_15451|MACS_peak_15451	10411|10411
chr8	97426770	97441998	Ccdc102a	0	-	MACS_peak_15451	-4816
chr8	97623727	97637242	Kifc3	0	-	MACS_peak_15452	-9377
chr8	97605100	97623774	Katnb1	0	+	MACS_peak_15452	-22765

chr8	97876236	97898193	Mmp15	0	+	MACS_peak_15453	28055
chr8	97850035	97855850	Zfp319	0	-	MACS_peak_15453	-7669
chr8	97835512	97845228	Tepp	0	+	MACS_peak_15453|MACS_peak_15453	-12669|-12767
chr8	97856183	97871413	AA960436	0	+	MACS_peak_15453	8002
chr8	97814493	97830485	BC016201	0	-	MACS_peak_15453	17696
chr8	97944151	97958769	Gtl3	0	-	MACS_peak_15456|MACS_peak_15457	15378|21064
chr8	97969995	98012720	Csnk2a2	0	-	MACS_peak_15458|MACS_peak_15459	-11546|-1173
chr8	98157458	98168959	Gins3	0	+	MACS_peak_15460	-23597
chr9	102620133	102635747	Amotl2	0	+	MACS_peak_16428	-5914
chr9	102641977	102659015	Gm5627	0	+	MACS_peak_16428	15930
chr9	103090362	103104395	Srprb	0	-	MACS_peak_16430	-17814
chr9	103948917	103965451	Uba5	0	-	MACS_peak_16432	-13836
chr9	103966032	104029976	Acad11	0	+	MACS_peak_16432	14417
chr9	105303117	105308084	Aste1	0	+	MACS_peak_16441|MACS_peak_16441|N	-20811|-26207|-22228|-27624
chr9	105064796	105297617	Nek11	0	-	MACS_peak_16441|MACS_peak_16442	26311|27728
chr9	105545324	105589985	Pik3r4	0	+	MACS_peak_16450|MACS_peak_16452	-15456|-24815
chr9	106270962	106278052	Dusp7	0	+	MACS_peak_16461	-424
chr9	106342381	106350009	Abhd14a	0	-	MACS_peak_16464|MACS_peak_16464|N	14207|14207|14207
chr9	106366291	106368271	Gpr62	0	-	MACS_peak_16464	-4055
chr9	106356187	106366340	Pcbp4	0	+	MACS_peak_16464	-8029
chr9	106335326	106340567	Acy1	0	-	MACS_peak_16464	23649
chr9	106350970	106355249	Abhd14b	0	+	MACS_peak_16464	-13246
chr9	106372683	106378982	Parp3	0	-	MACS_peak_16464	-14766
chr9	106379639	106387746	Rrp9	0	+	MACS_peak_16464	15423
chr9	106561076	106588279	Tex264	0	-	MACS_peak_16465|MACS_peak_16465	-459|-178
chr9	106546867	106558440	Grm2	0	-	MACS_peak_16465	29380
chr9	106724306	106783323	Vprbp	0	+	MACS_peak_16466|MACS_peak_16467	-14655|-25921
chr9	106786317	106789331	Rbm15b	0	-	MACS_peak_16468	-27315
chr9	107444539	107448037	Nprl2	0	+	MACS_peak_16469|MACS_peak_16472	7509|-12625
chr9	107436275	107440987	Tmem115	0	+	MACS_peak_16469|MACS_peak_16472	-755|-20889
chr9	107456986	107464591	Rassf1	0	+	MACS_peak_16469|MACS_peak_16472	19956|-178
chr9	107441341	107444196	Cyb561d2	0	-	MACS_peak_16469|MACS_peak_16472	-7166|12968
chr9	107465585	107468439	Tusc2	0	+	MACS_peak_16469|MACS_peak_16472	28555|8421
chr9	107449640	107453650	Zmynd10	0	+	MACS_peak_16469|MACS_peak_16472	12610|-7524
chr9	107479282	107482464	Hyal1	0	+	MACS_peak_16472|MACS_peak_16478	22118|-28075
chr9	107471493	107475109	Hyal2	0	+	MACS_peak_16472	14329
chr9	107482500	107486379	Nat6	0	+	MACS_peak_16472|MACS_peak_16478	25336|-24857
chr9	107483626	107489688	Hyal3	0	+	MACS_peak_16472|MACS_peak_16478	26462|-23731
chr9	107500445	107511572	Sema3b	0	-	MACS_peak_16478|MACS_peak_16478	-4215|-376
chr9	107495397	107499406	BY080835	0	-	MACS_peak_16478	7951
chr9	107490048	107495369	Ifrd2	0	+	MACS_peak_16478	-17309
chr9	107576804	107581923	Gnat1	0	-	MACS_peak_16485|MACS_peak_16490	7931|12852
chr9	107583832	107612806	Sema3f	0	-	MACS_peak_16485|MACS_peak_16490	-22952|-18031
chr9	107553485	107569705	Slc38a3	0	-	MACS_peak_16485|MACS_peak_16490	20149|25070
chr9	107642825	107673333	Rbm5	0	-	MACS_peak_16494	17140
chr9	107675890	107775150	Rbm6	0	-	MACS_peak_16495|MACS_peak_16495	-19981|-19981
chr9	107982766	107987358	Mst1	0	+	MACS_peak_16500|MACS_peak_16504|N	22227|11391|3038
chr9	107954170	107980134	Rnf123	0	-	MACS_peak_16500|MACS_peak_16504|N	-19595|-8759|-406
chr9	107955489	107958032	Amigo3	0	+	MACS_peak_16500|MACS_peak_16504|N	-5050|-15886|-24239
chr9	107951620	107954267	Gmppb	0	+	MACS_peak_16500|MACS_peak_16504|N	-8919|-19755|-28108
chr9	107987745	107996811	Apeh	0	-	MACS_peak_16504|MACS_peak_16506	-25436|-17083
chr9	108199252	108203928	Amt	0	-	MACS_peak_16508	-9540
chr9	108208535	108240270	Rhoa	0	+	MACS_peak_16508	-257
chr9	108192787	108198827	Nicn1	0	+	MACS_peak_16508	-16005
chr9	108205288	108208282	Tcta	0	-	MACS_peak_16508	510
chr9	108411809	108418272	Qars	0	+	MACS_peak_16511|MACS_peak_16511|N	29542|28068|28140|26666|26876|25402|15767|14293|13316|11842|12573|11099|10643|9169
chr9	108393006	108404666	Usp19	0	+	MACS_peak_16511|MACS_peak_16511|N	10739|10739|10739|10739|10739|9337|9337|9337|9337|9337|8073|8073|8073|8073|8073|-3036|-3036|-3036|-3036|-3036|-5487|-5487|
chr9	108382192	108392861	Lamb2	0	+	MACS_peak_16511|MACS_peak_16513|N	-75|-1477|-2741|-13850|-16301|-17044|-18974
chr9	108349969	108363912	Klhdc8b	0	-	MACS_peak_16511|MACS_peak_16513|N	18355|19757|21021
chr9	108362848	108368276	Ccdc71	0	+	MACS_peak_16511|MACS_peak_16513|N	-19419|-20821|-22085
chr9	108419417	108462498	Qrich1	0	+	MACS_peak_16520|MACS_peak_16520|N	23375|23375|20924|20924|20181|20181|18251|18251
chr9	108698324	108708663	Ip6k2	0	+	MACS_peak_16529|MACS_peak_16530	-9865|-15937
chr9	108710710	108720697	Nckipsd	0	+	MACS_peak_16529|MACS_peak_16530	2521|-3551
chr9	108728650	108755300	Celsr3	0	+	MACS_peak_16529|MACS_peak_16530	20461|14389
chr9	110034527	110142682	Smarcc1	0	+	MACS_peak_16532	-3605
chr9	109986823	110018086	Dhx30	0	-	MACS_peak_16532	20046
chr9	110287592	110310714	Ptpn23	0	-	MACS_peak_16534|MACS_peak_16535|N	-22688|-20539|-19376|-18889|-18580|-17522|-93
chr9	110322311	110325516	Ngp	0	+	MACS_peak_16539|MACS_peak_16540	29119|11690
chr9	110523638	110526897	Nradd	0	-	MACS_peak_16541|MACS_peak_16542|N	-2899|-2197|-29
chr9	110527292	110556665	Nbeal2	0	-	MACS_peak_16543	-29797
chr9	110782677	110803033	Als2cl	0	+	MACS_peak_16544|MACS_peak_16544|N	-5691|-5543|-5691|-19813|-19665|-19813
chr9	110760470	110767132	Prss50	0	+	MACS_peak_16544	-27898
chr9	110768549	110782587	Tmie	0	-	MACS_peak_16544|MACS_peak_16545	5781|19903
chr9	111020614	111128172	Lrrfip2	0	-	MACS_peak_16546|MACS_peak_16546	-174|-174
chr9	114310236	114383495	Glb1	0	+	MACS_peak_16553|MACS_peak_16554|N	-15927|-19314|-22867|-29285
chr9	114284248	114299830	Crtap	0	-	MACS_peak_16553|MACS_peak_16554	26333|29720
chr9	114640320	114658461	Cmtm6	0	+	MACS_peak_16561|MACS_peak_16562	7479|-8901
chr9	118387306	118395249	Eomes	0	+	MACS_peak_16569|MACS_peak_16569	-28229|-28229
chr9	118415435	118491637	Golga4	0	+	MACS_peak_16569	-100
chr9	118961895	118980643	Vill	0	+	MACS_peak_16586|MACS_peak_16586|N	-17872|-17872|-21707|-21707|-22195|-22195
chr9	118980645	119002614	Plcd1	0	-	MACS_peak_16586|MACS_peak_16589|N	-22847|-19012|-18524|-77
chr9	119011595	119056811	Dlec1	0	+	MACS_peak_16589|MACS_peak_16590|N	27993|27505|9058
chr9	119129608	119140810	9330176C(	0	-	MACS_peak_16594	8140
chr9	119149783	119213966	Oxsr1	0	-	MACS_peak_16595|MACS_peak_16596	-20229|17246
chr9	119245105	119249158	Myd88	0	-	MACS_peak_16596	-17946
chr9	119250411	119259413	Acaa1a	0	+	MACS_peak_16596	19199
chr9	119354040	119374635	Exog	0	+	MACS_peak_16597|MACS_peak_16597	12145|12145
chr9	119804012	119835697	Wdr48	0	+	MACS_peak_16598|MACS_peak_16599	-16159|-18781
chr9	119846723	119876911	Ttc21a	0	+	MACS_peak_16598|MACS_peak_16599|N	26552|23930|11984
chr9	119834790	119846676	Gorasp1	0	-	MACS_peak_16598|MACS_peak_16599|N	-26505|-23883|-11937
chr9	121669150	121680859	Zfp651	0	+	MACS_peak_16610	-7282
chr9	121686724	121692937	Kbtbd5	0	+	MACS_peak_16610	10292
chr9	121693134	121701625	Hhatl	0	-	MACS_peak_16610|MACS_peak_16610	-25193|-23806
chr9	121665757	121669061	E530011L2	0	-	MACS_peak_16610	7371
chr9	123715594	123720872	Cxcr6	0	+	MACS_peak_16625|MACS_peak_16627	8848|-15951
chr9	123698627	123761020	Fyco1	0	-	MACS_peak_16627|MACS_peak_16627|N	-29475|-29237|-79|159|16078|16316
chr9	123761435	123771246	Xcr1	0	-	MACS_peak_16634|MACS_peak_16635	-10305|5852
chr9	13424432	13513977	Maml2	0	+	MACS_peak_15728	-1201
chr9	14346410	14419444	Amotl1	0	-	MACS_peak_15739|MACS_peak_15740	-18474|-173
chr9	19426534	19454174	Zfp317	0	+	MACS_peak_15744	-7057
chr9	19461482	19463124	Olfr856-ps:	0	-	MACS_peak_15744	-29533
chr9	20299519	20325863	5730601F(	0	-	MACS_peak_15748|MACS_peak_15748|N	-25414|-25414|-372|-372
chr9	20272992	20297190	Zfp426	0	-	MACS_peak_15748|MACS_peak_15748|N	3259|3259|28301|28301
chr9	20442230	20449211	Fbxl12	0	-	MACS_peak_15753|MACS_peak_15753|N	-2711|-750|-1096|865
chr9	20456573	20471028	Pin1	0	+	MACS_peak_15753|MACS_peak_15754	10073|8458
chr9	20447761	20451233	Ubl5	0	+	MACS_peak_15753|MACS_peak_15754	1261|-354
chr9	20698792	20703034	Eif3g	0	-	MACS_peak_15756|MACS_peak_15757|N	-24635|-23287|-22987|-21259|-20943
chr9	20678252	20684154	Angptl6	0	-	MACS_peak_15756|MACS_peak_15757|N	-5755|-4407|-4107|-2379|-2063
chr9	20692618	20696623	Ppan	0	+	MACS_peak_15756|MACS_peak_15757|N	14219|12871|12571|10843|10527
chr9	20673085	20678751	A230050P2	0	+	MACS_peak_15756|MACS_peak_15757|N	-5314|-6662|-6962|-8690|-9006
chr9	21172446	21209805	Ilf3	0	+	MACS_peak_15763|MACS_peak_15763|N	13657|13657|13657|13657
chr9	21142295	21159472	Slc44a2	0	+	MACS_peak_15763	-16494
chr9	21229388	21311568	Dnm2	0	+	MACS_peak_15764	-162
chr9	21216280	21224723	Qtrt1	0	+	MACS_peak_15764	-13270
chr9	21300938	21301008	Mir199a-1	0	-	MACS_peak_15766|MACS_peak_15768|N	-28931|-21971|-20023|-2003|3749
chr9	21311823	21314630	Tmed1	0	-	MACS_peak_15771|MACS_peak_15773	-15625|-9873
chr9	21330720	21349237	AB124611	0	+	MACS_peak_15773	25963
chr9	21528037	21554362	Ldlr	0	+	MACS_peak_15779|MACS_peak_15782|N	-8828|-15324|-15879|-16165|-18651|-23634
chr9	21559885	21564730	Spc24	0	-	MACS_peak_15779|MACS_peak_15782|N	-27865|-21369|-20814|-20528|-18042|-13059|20347
chr9	21571216	21602990	Kank2	0	-	MACS_peak_15791	-17913
chr9	21731914	21740316	Ccdc159	0	+	MACS_peak_15802|MACS_peak_15802	-11012|-11012
chr9	21763342	21768020	Epor	0	-	MACS_peak_15802	-25094

chr9	21760196	21762714	2310047B:	0	+	MACS_peak_15802	17270
chr9	21711954	21722565	Rab3d	0	-	MACS_peak_15802	20361
chr9	21742717	21753351	BC018242	0	+	MACS_peak_15802	-209
chr9	21725452	21731906	Tmem205	0	-	MACS_peak_15802	11020
chr9	21794314	21807078	Ccdc151	0	-	MACS_peak_15806|MACS_peak_15806|M	415|415|832|832|1912|1912|7928|7928|8576|8576|9630|9630
chr9	21807478	21818666	Prkcsh	0	+	MACS_peak_15806|MACS_peak_15807|M	-15|-432|-1512|-7528|-8176|-9230
chr9	21775970	21793897	Rgl3	0	-	MACS_peak_15806|MACS_peak_15807|M	13596|14013|15093|21109|21757|22811
chr9	21819449	21856467	Elavl3	0	-	MACS_peak_15818|MACS_peak_15819|M	13771|21011|26142
chr9	21876689	21889826	Ecsit	0	-	MACS_peak_15818|MACS_peak_15819|M	-19588|-12348|-7217
chr9	21859903	21875789	Zfp653	0	-	MACS_peak_15818|MACS_peak_15819|M	-5551|1689|6820
chr9	21903696	21913665	Cnn1	0	+	MACS_peak_15819|MACS_peak_15820	26218|21087
chr9	24912574	24956280	Herpud2	0	-	MACS_peak_15835|MACS_peak_15837	-26890|-4347
chr9	26781725	26807134	Acad8	0	-	MACS_peak_15841	-21163
chr9	26807261	26814919	Thyn1	0	+	MACS_peak_15841	21290
chr9	30234913	30274311	Snx19	0	+	MACS_peak_15843	-976
chr9	30838228	30883470	Zbtb44	0	+	MACS_peak_15844|MACS_peak_15844	-23210|-23210
chr9	3335230	3385846	Alkbh8	0	+	MACS_peak_15694	-7978
chr9	3404084	3479236	Cwf19l2	0	+	MACS_peak_15695	-24644
chr9	34991975	35007876	Tirap	0	-	MACS_peak_15852|MACS_peak_15852|M	7235|7561|7621|7561|13704|14030|14090|14030|14355|14681|14741|14681|14633|14959|15019|14959
chr9	35018787	35024588	Srpr	0	+	MACS_peak_15852|MACS_peak_15853|M	3676|-2793|-3444|-3722
chr9	35011805	35018510	Foxred1	0	-	MACS_peak_15852|MACS_peak_15853|M	-3399|3070|3721|3999
chr9	35381222	35382248	Gm6762	0	-	MACS_peak_15858	-7859
chr9	35349432	35366055	Ddx25	0	-	MACS_peak_15858	8334
chr9	35367050	35374985	Pus3	0	+	MACS_peak_15858	-7339
chr9	35368405	35377654	Hyls1	0	-	MACS_peak_15858	-3265
chr9	36443338	36446187	A630095E1	0	-	MACS_peak_15860	-1373
chr9	36651243	36686225	Fez1	0	+	MACS_peak_15865|MACS_peak_15866|M	-6741|-17195|-26854
chr9	38525852	38550922	Vwa5a	0	+	MACS_peak_15891|MACS_peak_15891|M	-19508|-19508|-19684|-19684
chr9	38560380	38564448	Olfr920	0	+	MACS_peak_15891|MACS_peak_15892|M	15020|14844|4485
chr9	38580672	38583939	Olfr921	0	+	MACS_peak_15898	24777
chr9	43552658	43615544	Pvrl1	0	+	MACS_peak_15916	-26
chr9	44144430	44150464	Hmbs	0	-	MACS_peak_15926|MACS_peak_15926|M	-26215|-28062|-22414|-24261|12101|10254|17297|15450
chr9	44134927	44141683	Dpagt1	0	+	MACS_peak_15926|MACS_peak_15927|M	10678|6877|-27638
chr9	44142797	44144156	H2afx	0	+	MACS_peak_15926|MACS_peak_15927|M	18548|14747|-19768|-24964
chr9	44117319	44128365	C2cd2l	0	-	MACS_peak_15926|MACS_peak_15927	-4116|-315
chr9	44081272	44096327	Abcg4	0	-	MACS_peak_15926	27922
chr9	44103755	44113754	Hinfp	0	-	MACS_peak_15926|MACS_peak_15927	10495|14296
chr9	44187572	44200451	Hyou1	0	+	MACS_peak_15928|MACS_peak_15929|M	25007|19811|-1336|-1603|-2441|-5323|-5722
chr9	44156187	44169753	Vps11	0	-	MACS_peak_15928|MACS_peak_15929|M	-7188|-1992|19155|19422|20260|23142|23541
chr9	44206258	44211048	Slc37a4	0	+	MACS_peak_15930|MACS_peak_15931|M	17350|17083|16245|13363|12964
chr9	44211841	44215631	Trappc4	0	-	MACS_peak_15930|MACS_peak_15931|M	-26723|-26456|-25618|-22736|-22337
chr9	44215796	44218489	Rps25	0	+	MACS_peak_15930|MACS_peak_15931|M	26888|26621|25783|22901|22502
chr9	44412974	44448814	Ddx6	0	+	MACS_peak_15943|MACS_peak_15943|M	-25192|-25192|-25192
chr9	44481320	44494387	Treh	0	+	MACS_peak_15945	9078
chr9	44721332	44728825	Atp5l	0	-	MACS_peak_15950	16946
chr9	44731209	44773683	Ube4a	0	-	MACS_peak_15950|MACS_peak_15951	-27912|-2220
chr9	44777654	44788514	Cd3g	0	-	MACS_peak_15951	-17051
chr9	44789868	44795135	Cd3d	0	+	MACS_peak_15951	18405
chr9	45575028	45636721	Cep164	0	-	MACS_peak_15952	28644
chr9	45646611	45670567	Bace1	0	+	MACS_peak_15952|MACS_peak_15952	-18754|-18754
chr9	45737710	45744141	Tagln	0	-	MACS_peak_15953|MACS_peak_15956	2538|18876
chr9	45745957	45763332	Sidt2	0	-	MACS_peak_15953|MACS_peak_15956	-16653|-315
chr9	45773393	45792954	Pafah1b2	0	-	MACS_peak_15956	-29937
chr9	48743112	48773095	Htr3b	0	-	MACS_peak_15971	20457
chr9	48793489	48850622	Usp28	0	+	MACS_peak_15971|MACS_peak_15972	-63|-18517
chr9	49223326	49235126	Ankk1	0	-	MACS_peak_15973|MACS_peak_15974	18805|28878
chr9	49245065	49294330	Ttc12	0	-	MACS_peak_15981	29394
chr9	49326392	49376130	Gm11149	0	+	MACS_peak_15981|MACS_peak_15983	2668|-5298
chr9	50413344	50425569	AU019823	0	-	MACS_peak_15998|MACS_peak_15998	-7110|-7110
chr9	50412005	50413425	Timm8b	0	+	MACS_peak_15998	-6454
chr9	50404445	50411954	Sdhd	0	-	MACS_peak_15998	6505
chr9	50425425	50433097	Pih1d2	0	+	MACS_peak_15998	6966
chr9	50700905	50817178	Sik2	0	-	MACS_peak_16009	-13431
chr9	53213390	53240908	4930550C:	0	+	MACS_peak_16019	5234
chr9	53192127	53209972	Kdelc2	0	+	MACS_peak_16019	-16029
chr9	54434317	54452469	Idh3a	0	+	MACS_peak_16024|MACS_peak_16025	-18674|-23257
chr9	55056741	55072240	Fbxo22	0	+	MACS_peak_16035	23226
chr9	55889651	55906890	Rcn2	0	+	MACS_peak_16037	-16620
chr9	55983691	56008877	Tspan3	0	-	MACS_peak_16040	-13586
chr9	56266652	56344743	Hmg20a	0	+	MACS_peak_16052	74
chr9	56048935	56265857	C230081A:	0	-	MACS_peak_16052	721
chr9	56785306	56786205	Imp3	0	+	MACS_peak_16060|MACS_peak_16061|M	29243|28545|28253|24866|24106|23510
chr9	56765006	56776178	Snx33	0	-	MACS_peak_16060|MACS_peak_16061|M	-20115|-19417|-19125|-15738|-14978|-14382
chr9	56924182	56976175	Sin3a	0	+	MACS_peak_16079|MACS_peak_16079|M	19628|15292|19628
chr9	57002847	57006106	Commd4	0	-	MACS_peak_16081	-15092
chr9	56991062	56994841	Neil1	0	-	MACS_peak_16081	-3827
chr9	56978583	56990017	Man2c1	0	+	MACS_peak_16081	-12431
chr9	58336409	58347549	6030419C:	0	+	MACS_peak_16100|MACS_peak_16101	-1594|-10622
chr9	59139378	59164007	Adpgk	0	+	MACS_peak_16114|MACS_peak_16115|M	-6068|-18717|-21552|-23891
chr9	59465090	59498076	Parp6	0	+	MACS_peak_16131|MACS_peak_16132|M	-6686|-17632|-22807
chr9	59504414	59527182	Pkm2	0	+	MACS_peak_16132|MACS_peak_16133	21692|16517
chr9	59799100	59806850	Gm7616	0	+	MACS_peak_16138|MACS_peak_16139|M	-16022|-16464|-16774|-17921|-18366|-19222|-19401|-19550|-19817|-20283|-20665|-21009|-28601
chr9	59790577	59797886	Nr2e3	0	-	MACS_peak_16138|MACS_peak_16139|M	17236|17678|17988|19135|19580|20436|20615|20764|21031|21497|21879|22223|29815
chr9	61220172	61266304	Tle3	0	+	MACS_peak_16158|MACS_peak_16158|M	-20866|-20866|-20866
chr9	61905055	61918413	Glce	0	+	MACS_peak_16170|MACS_peak_16172	-2102|11251
chr9	63011576	63225659	Map2k5	0	-	MACS_peak_16177	-380
chr9	63242253	63247051	2300009A(	0	-	MACS_peak_16177	-21772
chr9	64021193	64026369	Rpl4	0	+	MACS_peak_16188	-14319
chr9	63984952	64020738	Zwilch	0	-	MACS_peak_16188	14774
chr9	64027103	64030495	Snapc5	0	+	MACS_peak_16188	-8409
chr9	64023238	64023327	Snord16a	0	+	MACS_peak_16188	-12274
chr9	64154562	64189064	Dis3l	0	-	MACS_peak_16193|MACS_peak_16193|M	-27274|-26790|-26474|-188|296|612
chr9	64658817	64767473	Dennd4a	0	+	MACS_peak_16196	-25008
chr9	65142101	65178465	Clpx	0	+	MACS_peak_16207|MACS_peak_16207	-23126|-23126
chr9	65193874	65207450	Pdcd7	0	+	MACS_peak_16207	28647
chr9	66365687	66440787	Usp3	0	-	MACS_peak_16213	-26108
chr9	70526807	70628036	Adam10	0	+	MACS_peak_16243	22239
chr9	70446820	70504981	Fam63b	0	-	MACS_peak_16243	-413
chr9	7184565	7207031	Dcun1d5	0	+	MACS_peak_15721|MACS_peak_15723	21768|9762
chr9	6928502	7177046	Dync2h1	0	-	MACS_peak_15721|MACS_peak_15723	-14249|-2243
chr9	72833357	72861745	Ccpg1	0	+	MACS_peak_16272|MACS_peak_16272|M	-13947|-13947|-16240|-16240
chr9	72863502	72887506	Pigb	0	-	MACS_peak_16276	74
chr9	72892671	72945399	Rab27a	0	+	MACS_peak_16276	5091
chr9	73327230	73781374	Unc13c	0	-	MACS_peak_16278|MACS_peak_16279|M	-1503|-1255|36
chr9	75159201	75192769	Gnb5	0	+	MACS_peak_16284|MACS_peak_16284	-2900|-53
chr9	75234707	75257821	Mapk6	0	-	MACS_peak_16290|MACS_peak_16290|M	-22049|-21394|-19748|-19093|-14406|-13751|-11850|-11195|-712|-57
chr9	75289330	75314239	Leo1	0	+	MACS_peak_16296	-5611
chr9	75345590	75407464	Tmod3	0	-	MACS_peak_16297	-27140
chr9	7764076	7794332	Tmem123	0	+	MACS_peak_15724	-27310
chr9	77929305	77956860	Fbxo9	0	-	MACS_peak_16311|MACS_peak_16311	-542|-145
chr9	77956998	78019914	Ick	0	+	MACS_peak_16311|MACS_peak_16311	680|4726
chr9	7818225	7835255	Birc2	0	-	MACS_peak_15726	15
chr9	78384315	78435834	Slc17a5	0	-	MACS_peak_16321|MACS_peak_16322	-27270|-10904
chr9	82759765	82869096	Phip	0	-	MACS_peak_16357|MACS_peak_16358	-15735|-124
chr9	85736458	85740662	Tpbg	0	+	MACS_peak_16361|MACS_peak_16361	362|-110
chr9	86465659	86468505	Rwdd2a	0	+	MACS_peak_16365|MACS_peak_16365|M	12616|12551|12616|9426|9361|9426|276|211|276
chr9	86446082	86465449	Pgm3	0	-	MACS_peak_16365|MACS_peak_16365|M	-12406|-12406|-9216|-9216|-66|-66
chr9	87086797	87150367	4922501C(	0	-	MACS_peak_16370	-17660
chr9	88271584	88333789	Snx14	0	-	MACS_peak_16385	-18664
chr9	89949104	89970933	Ctsh	0	+	MACS_peak_16392|MACS_peak_16393	-9937|-10276
chr9	90057815	90094940	Adamts7	0	+	MACS_peak_16396|MACS_peak_16397|M	-14849|-25744|-28916
chr9	90096886	90165607	Tbc1d2b	0	-	MACS_peak_16400|MACS_peak_16401	-29678|-172

chr9	92204214	92252714	1700057G(	0	+	MACS_peak_16403	-25517
chr9	98464149	98488794	Copb2	0	+	MACS_peak_16419|MACS_peak_16421|M	-39|-13297|-18605
chr9	98462878	98464030	4930579K:	0	-	MACS_peak_16419|MACS_peak_16421|M	158|13416|18724
chr9	98489151	98502098	Mrps22	0	-	MACS_peak_16421|MACS_peak_16422	-24652|-19344
chrX	101678120	101701601	Uprt	0	+	MACS_peak_16994|MACS_peak_16995|M	-19035|-25850|-28313
chrX	102695110	102695974	Cypt2-ps	0	+	MACS_peak_16999	17648
chrX	10294490	10296827	Mid1ip1	0	+	MACS_peak_16685|MACS_peak_16685|M	-220|-220|-1472|-1472
chrX	11613865	11657679	Bcor	0	-	MACS_peak_16687|MACS_peak_16687|M	-21610|-21610|-21610|-21610
chrX	12164884	12194175	Atp6ap2	0	+	MACS_peak_16688|MACS_peak_16689	-119|-19247
chrX	12232009	12250672	18100300(	0	-	MACS_peak_16691	13127
chrX	130994654	131011029	Taf7l	0	-	MACS_peak_17049	13763
chrX	131252476	131256449	Armcx1	0	+	MACS_peak_17053|MACS_peak_17053|M	-485|-485|-485|-485|-485
chrX	13209894	13217984	Gpr34	0	+	MACS_peak_16698	6516
chrX	132277230	132338007	Gprasp1	0	+	MACS_peak_17057|MACS_peak_17057|M	-20908|-20908|-20908|-23440|-23440|-23440
chrX	132277230	132281865	Armcx5	0	+	MACS_peak_17057|MACS_peak_17058	-20908|-23440
chrX	133267489	133275694	Morf4l2	0	-	MACS_peak_17061|MACS_peak_17061|M	-7427|-7624|-9190|-9958|-7624|-9958|-7881
chrX	133242603	133244405	Tceal1	0	+	MACS_peak_17061	-25664
chrX	133277495	133337898	BC065397	0	+	MACS_peak_17061	9228
chrX	137212566	137302254	Mid2	0	+	MACS_peak_17067	-15090
chrX	137342845	137347972	Tex13	0	-	MACS_peak_17068	-15264
chrX	138739295	138740741	Kcne1l	0	-	MACS_peak_17084	21852
chrX	141880966	141921589	Amot	0	-	MACS_peak_17092	-6891
chrX	147240963	147248825	Maged2	0	-	MACS_peak_17101|MACS_peak_17103	-6654|-3898
chrX	147417675	147451836	Gnl3l	0	-	MACS_peak_17106|MACS_peak_17106|M	-13707|-13736|-59|-88
chrX	147481775	147523629	Fgd1	0	+	MACS_peak_17107	29998
chrX	147521637	147531086	Tsr2	0	-	MACS_peak_17108|MACS_peak_17108|M	-9650|-9650|-9650
chrX	147955214	148068400	Phf8	0	+	MACS_peak_17109|MACS_peak_17109	-1855|-1855
chrX	148237824	148369961	Huwe1	0	+	MACS_peak_17114|MACS_peak_17115	38|-196
chrX	148346888	148346971	Mirlet7f-2	0	+	MACS_peak_17123|MACS_peak_17124|M	22317|20427|18427|13794|10758|8428|3545|-6309|-7525|-17065
chrX	148347756	148347864	Mir98	0	+	MACS_peak_17123|MACS_peak_17124|M	23185|21295|19295|14662|11626|9296|4413|-5441|-6657|-16197
chrX	148436438	148438985	Hsd17b10	0	+	MACS_peak_17133	-7683
chrX	148450970	148496515	Smc1a	0	+	MACS_peak_17133	6849
chrX	148439126	148450838	Ribc1	0	-	MACS_peak_17133	-6717
chrX	148779188	148802654	Gpr173	0	-	MACS_peak_17142	-27975
chrX	148771394	148777027	Tspyl2	0	-	MACS_peak_17142	-2348
chrX	154004050	154006917	Yy2	0	-	MACS_peak_17157	-27348
chrX	155810126	155823631	Eif1ax	0	+	MACS_peak_17158|MACS_peak_17159	20795|-10147
chrX	156940365	157036810	Phka2	0	+	MACS_peak_17160|MACS_peak_17160|M	9760|9492|9760
chrX	159216850	159267331	Txlng	0	-	MACS_peak_17164	-17945
chrX	159399923	159402531	S100g	0	-	MACS_peak_17166	10632
chrX	16196823	16264938	Maoa	0	+	MACS_peak_16702	8609
chrX	162676874	162826965	Gpm6b	0	+	MACS_peak_17171|MACS_peak_17171|M	-133|-133|-133|-28192|-28192|-28192|-28192|-28192|-28192
chrX	166412974	166416849	G530011O	0	-	MACS_peak_17179	6165
chrX	20126943	20139213	Rgn	0	+	MACS_peak_16708	20634
chrX	20240080	20259829	Uba1	0	+	MACS_peak_16709|MACS_peak_16709|M	-6242|-10776|-7852|-12386
chrX	20265618	20277003	Cdk16	0	+	MACS_peak_16709|MACS_peak_16710|M	19296|17686|-26140|-28355|-29635|-29894
chrX	20281034	20297665	Usp11	0	+	MACS_peak_16714|MACS_peak_16715|M	-10724|-12939|-14219|-14478
chrX	34414360	34424215	Ube2a	0	+	MACS_peak_16747	10688
chrX	34388538	34404241	C330007P(	0	-	MACS_peak_16747	-569
chrX	34690692	34708837	Akap14	0	-	MACS_peak_16748|MACS_peak_16749	-26974|-20576
chrX	34666757	34690741	Nkap	0	+	MACS_peak_16748|MACS_peak_16749	-15106|-21504
chrX	35542969	35550223	Zbtb33	0	+	MACS_peak_16750|MACS_peak_16750|M	8071|8071|7457|7457
chrX	45610110	45635626	Utp14a	0	+	MACS_peak_16768|MACS_peak_16769|M	12|-6623|-15920
chrX	45694534	45759905	Bcorl1	0	+	MACS_peak_16771	-27948
chrX	50698197	50723669	Mospd1	0	-	MACS_peak_16791|MACS_peak_16793	-25592|-24543
chrX	67647075	67647915	4930567H	0	-	MACS_peak_16822|MACS_peak_16823	25874|26987
chrX	67713230	67730218	BC023829	0	-	MACS_peak_16824	7
chrX	70918471	70927837	Slc6a8	0	+	MACS_peak_16837|MACS_peak_16837|M	-737|-737|-737|-3667|-3667|-3667|-4257|-4257|-4257
chrX	70901332	70905167	Pnck	0	-	MACS_peak_16837|MACS_peak_16838|M	14041|16971|17561
chrX	70961935	70983626	Abcd1	0	+	MACS_peak_16842	-12477
chrX	70931521	70961514	Bcap31	0	-	MACS_peak_16842	12898
chrX	71002441	71017849	Plxnb3	0	+	MACS_peak_16842	28029
chrX	71188133	71211654	Hcfc1	0	-	MACS_peak_16850	513
chrX	7156818	7172360	Foxp3	0	+	MACS_peak_16660	19477
chrX	7135687	7151246	Ppp1r3f	0	-	MACS_peak_16660	-13905
chrX	7150725	7158142	4930524L2	0	+	MACS_peak_16660	13384
chrX	71468799	71491873	Flna	0	-	MACS_peak_16854|MACS_peak_16855|M	-22773|-21694|-17743|-9664|-9028|-6789|-6521|-5066|-3932|-241
chrX	71500177	71503086	Emd	0	+	MACS_peak_16855|MACS_peak_16856|M	29998|26047|17968|17332|15093|14825|13370|12236|8545
chrX	71516154	71518473	Rpl10	0	+	MACS_peak_16861|MACS_peak_16862|M	29347|28213|24522|-18907|-29859
chrX	71517830	71517959	Snora70	0	+	MACS_peak_16862|MACS_peak_16863|M	29889|26198|-17231|-28183
chrX	71518555	71527672	Dnase1l1	0	-	MACS_peak_16867|MACS_peak_16867|M	7389|7986|18341|18938|28986|29583
chrX	71528035	71535489	Taz	0	+	MACS_peak_16867|MACS_peak_16867|M	-7026|-7026|-7026|-7026|-17978|-17978|-17978|-17978|-28623|-28623|-28623|-28623
chrX	71550350	71557206	Gdi1	0	+	MACS_peak_16867|MACS_peak_16868|M	15289|4337|-6308|-8192
chrX	71542435	71550060	Atp6ap1	0	+	MACS_peak_16867|MACS_peak_16868|M	7374|-3578|-14223|-16107
chrX	71558371	71565487	Fam50a	0	+	MACS_peak_16867|MACS_peak_16868|M	23310|12358|1713|-171|-27451
chrX	71574404	71590028	Plxna3	0	+	MACS_peak_16868|MACS_peak_16871|M	28391|17746|15862|-11418
chrX	71597500	71598957	Lage3	0	-	MACS_peak_16874	-13135
chrX	71612739	71613866	Ubl4	0	-	MACS_peak_16874|MACS_peak_16877|M	-28044|17974|18173
chrX	71638629	71699117	Ikbkg	0	+	MACS_peak_16877|MACS_peak_16877|M	6789|6789|6590|6590|-3208|-3208|1495|1861|1861
chrX	71630058	71638478	Fam3a	0	-	MACS_peak_16877|MACS_peak_16878	-6638|-6439
chrX	71614557	71618595	Slc10a3	0	-	MACS_peak_16877|MACS_peak_16878	13245|13444
chrX	71654824	71674500	G6pdx	0	-	MACS_peak_16882	-1342
chrX	72655780	72661882	Mtcp1	0	-	MACS_peak_16890|MACS_peak_16890|M	-11516|-11516|-6934|-6934|-453|-453
chrX	72418057	72625380	F8	0	-	MACS_peak_16890|MACS_peak_16890|M	24986|24986|24986|29568|29568|29568
chrX	72661966	72699339	Brcc3	0	+	MACS_peak_16890|MACS_peak_16890|M	11600|11600|11600|7018|7018|7018|537|537|537
chrX	72627737	72642496	Fundc2	0	+	MACS_peak_16890|MACS_peak_16891	-22629|-27211
chrX	73030992	73120509	Pls3	0	-	MACS_peak_16894|MACS_peak_16894	-86|510
chrX	7461369	7471153	Slc35a2	0	+	MACS_peak_16662|MACS_peak_16662	-8290|-8290
chrX	7476523	7484778	Timm17b	0	+	MACS_peak_16662	6864
chrX	7496947	7501534	Pcsk1n	0	+	MACS_peak_16662|MACS_peak_16664|M	27288|-11028|-16597
chrX	7471648	7476354	Pqbp1	0	-	MACS_peak_16662	-6695
chrX	7455431	7460558	Pim2	0	+	MACS_peak_16662	-14228
chrX	74920153	74920791	Gm6927	0	-	MACS_peak_16899	-14111
chrX	75007373	75041299	Prkx	0	-	MACS_peak_16900|MACS_peak_16901	-24675|-228
chrX	7507247	7525015	Hdac6	0	-	MACS_peak_16664|MACS_peak_16664|M	-17040|-17040|-11471|-11471
chrX	7501401	7505733	Eras	0	-	MACS_peak_16664|MACS_peak_16665	2242|7811
chrX	7719481	7722928	Rbm3	0	-	MACS_peak_16666|MACS_peak_16666|M	-15738|-15738|-15738|-15738
chrX	7700427	7709984	Wdr13	0	-	MACS_peak_16666|MACS_peak_16666	-2794|-2783
chrX	7722552	7725087	2900002K(	0	+	MACS_peak_16666	15362
chrX	8091177	8102080	Gm14459	0	-	MACS_peak_16674	-6713
chrX	82947275	83022158	Gyk	0	-	MACS_peak_16903|MACS_peak_16903	-280|-280
chrX	91780812	91787413	Maged1	0	-	MACS_peak_16921|MACS_peak_16922|M	-4121|-3850|-111
chrX	91969907	91975530	Zxdb	0	+	MACS_peak_16927	-3699
chrX	92615652	92640179	Fam123b	0	-	MACS_peak_16930|MACS_peak_16931	-23017|-20644
chrX	93651684	93769821	Heph	0	+	MACS_peak_16940|MACS_peak_16940|M	-16541|-17451|-17451|-16541|-19107|-20017|-20017|-19107
chrX	98599722	98615253	Zmym3	0	-	MACS_peak_16979|MACS_peak_16979|M	-9804|-9804|-10575|-10112|-10112|-6728|-6728|-7499|-7036|-7036|-3618|-3618|-4389|-3926|-3926
chrX	98624989	98643932	Nono	0	+	MACS_peak_16979|MACS_peak_16980|M	19540|16464|13354
chrX	98835402	98879688	Ogt	0	+	MACS_peak_16982|MACS_peak_16983|M	-3035|-12513|-14599

-29908|-29819

2

|-22091

'130|-18032|-17533|-17937

|69|56

17980|-17461|-16886|-16578|-16263

20964|-15250|-14478|-14049|-12852|-10353|-20376|-13890|-22308|-23220|-22708|-22308|-20376|-14662|-13890|-13461|-12264|-9765|-201

14432|-14282|-12529

.7

)49|29609|29609

11176|-10650

64|-18582|-19103|-20511|-21224|-22311|-22929|-23263|-23753|-24017|-25688|-25940|-26328|-27645
27|14119|13406|12319|11701|11367|10877|10613|8942|8690|8302|6985
21|14531|14267|12596|12344|11956|10639

69|13846|17827|17827|15268|19249|19249|18049|22030|22030|22194|26175|26175|22461|26442|26442|23555|27536|27536

-5487	-5487	-5487	-6230	-6230	-6230	-6230	-6230	-8160	-8160	-8160	-8160	-8160
