# Supplementary material for: MSI2 promotes translation of multiple IRES-containing oncogenes and virus to induce self-renewal of tumor initiating stem-like cells
Source: Cell Death Discov. 2023 Apr 28;9:141. doi: 10.1038/s41420-023-01427-9 (PMC10147607; doi:10.1038/s41420-023-01427-9)
Supplement: Supplementary file 4 — Supplementary Information [file 41420_2023_1427_MOESM4_ESM.pdf]

Supplementary Information

Supplementary Information Content:

o Supplementary Table 1. Overall peaks enriched in MSI2 Ip over control-3830 (Separate file)
o Supplementary Table 2. Peaks used for identification of motif with peak summits
o Supplementary Table 3. Overlap of RIP-seq from this publication with GSE93210, GSE54598 & GSE62115 (Separate file)
o Supplementary Table 4. List promoters enriched in RNA-seq data (Separate file)
o Supplementary Table 5. Key Resources Table
o Supplementary Table 6. Oligonucleotide sequences for qRT-PCR Sponge & shRNA targets
o Supplementary Table 7. Clinicopathological parameters of the HCC patients with Immunoreactivity score (IRS)

Figures
o Extended Data Fig. 1. RIP-seq analyses demonstrate that MSI2 is enriched in mRNAs of cellular genes. GRP137b and RSPH3a/b were among those cellular targets detected from MSI2 peaks.
o Extended Data Fig. 2A-S2C. UCSC Genome Browser for MYC loci and analyses of MSI2-binding motifs.
o Extended Data Fig. 2D. MSI2-binding motif analyses by use of MEME-ChIP.
o Extended Data Fig. 2E. Large database list used for MSI2-binding motif analyses by use of MEME-ChIP.
o Extended Data Fig. 2F. MSI2 silencing reduces IRES activity for pRMF while MSI2 overexpressing increases IRES activity.
o Extended Data Fig. 2G. The endogenous levels of MYC are positively correlated with MSI2 expression.
o Extended Data Fig. 2H, S2I & S2J. MSI2 regulates expression of cellular IRES-containing genes such as JUN and VEGFA.
o Extended Data Fig. 2K. MSI2 Binding Motif was identified using MEME algorithm.
o Extended Data Fig. 3A-S3B. MSI2 silencing reduces the luciferase activity for pRMF while MSI2 overexpression increases IRES activity. Production, purification and verification of His-tagged recombinant MSI2 and MSI2 RNA binding domain (RBD: aa8-193).
o Extended Data Fig. 3C-S3D. Production, purification and verification of His-tagged of recombinant hnRNPK and YB1.
o Extended Data Fig. 3E-S3IG. Production, purification and verification of His-tagged of recombinant PCBP 12 and MSI1RNA binding domain (RBD: aa7.aa192 with mutations containing R53E，R59E and R61).
o Extended Data Fig. 3H-S3I. DNA template preparation for in vitro transcription and EMSA assays to detect MSI2-MYC IRES RNA interaction.
o Extended Data Fig. 3J-S3L. Polysome assays revealed ribosome binding patterns were shifted by MSI2
o Extended Data Fig. 4A-S4B. Repression of miR-22 by MSI2 overexpression.
o Extended Data Fig. 4C-S4D. 3′-UTR of human MYC mRNA targeted by miR-22
o Extended Data Fig. 4E. 3′-UTR of mouse MYC mRNA targeted by miR-22
o Extended Data Fig. 5A-S5B. Immune Reactivity Score (IRS) of MYC in non-tumor and tumor regions of clinical HCC specimens.
o Extended Data Fig. 5C. Co-expression of MYC and MSI2 in human HCC specimens.

Supplementary Materials and Methods

Supplementary Discussion

Supplementary References

Supplementary Table 5. KEY RESOURCES TABLE

REAGENT or RESOURCE	SOURCE	IDENTIFIER
**Antibodies**		
MSI2 rabbit Monoclonal Antibody	EMD Millipore	03-115
MSI2 rabbit Polyclonal Antibody	Proteintech	10770-1-AP
Flag tag Monoclonal Antibody (M1)	Sigma Aldrich	F3040
β-Actin mouse Monoclonal Antibody	Santa Cruz	sc-69879
c-Myc mouse monoclonal Antibody	Santa Cruz	sc-40
**Chemicals, and Recombinant Proteins**		
Cycloheximide	Sigma Aldrich	239764-100MG
Triton X-100	Sigma Aldrich	T8787-50ML
Paraformaldehyde	Sigma Aldrich	P6148-500G
Polybrene	Sigma Aldrich	TR-1003
Puromycin Dihydrochloride	Thermo Fisher	A1113803
Protease Inhibitor	Roche	4693159001
Proteinase K	Roche	3115879001
One Shot™ Stbl3™ Chemically Competent E. coli	Thermo Fisher	C737303
Stellar™ Competent Cells	Takara	636766
Q5® High-Fidelity DNA Polymerase	NEB	M0491L
T4 DNA Ligase	NEB	M0202L
Quick Blunting™ Kit	NEB	E1201L
GenClone Fetal Bovine Serum, Heat Inactivated	Genesee Scientific	25-514H
Dulbecco's modified Eagle's medium (DMEM, High Glucose, with L-Glutamine, with Sodium Pyruvate)	Genesee Scientific	25-500
Glutamax	Thermo Fisher	35050061
Antibiotic:Antimycotic Solution	Gemini	400-101
DPBS	VWR	VWRL0117-0500
BioT	Bioland Scientific	B01-01
6X Laemmli Sample Buffer	Bioland Scientific	SAB03-01
LB Broth (Miller) Mix	Genesee Scientific	11-120
LB Agar (Miller) Mix	Genesee Scientific	11-122
PVDF membrane	EMD Millipore	ISEQ85R
Nylon membrane	Thermo Fisher	AM10104
X-ray film	Thermo Fisher	34091
TRIzol Reagent	Thermo Fisher	15596026
SuperScript III reverse transcriptase	Thermo Fisher	18080085
RNasin® Ribonuclease Inhibitors	Promega	N2511
EvaGreen miRNA qPCR Master Mix	Genomics-online	ABIN4219203
Brilliant II SYBR Green qPCR Master Mix	Stratagene	600828
SYBR Green PCR Master Mix	Thermo Fisher	4309155
Pierce ECL plus	Thermo Fisher	32132
Immobilon Western chemiluminescent HRP substrate	EMD Millipore	WPKLS0500
Protein A/G PLUS Agarose	Santa Cruz	Sc-2003

**Critical Commercial Assays**		
RIP-assay kit	MBL	RN1001
RNeasy mini kit	QIAGEN	74104
QIAprep Spin Miniprep Kit	QIAGEN	27106
MinElute Gel Extraction Kit	QIAGEN	28604
Lenti-X™ GoStix	Takara	631280
Quickchange Lightning site-directed mutagenesis kit	Agilent	210519
Dual-Luciferase Reporter Assay System	Promega	E1960
MAXIscript™ SP6/T7 Transcription Kit	Thermo Fisher	AM1322
mirPremier microRNA Isolation Kit	Sigma Aldrich	SNC50-1KT
miRNA cDNA Synthesis Kit	ABM	G270
Northern Blot Assay	Signosis	NB-3001
**Deposited Data**		
**Experimental Models: Cell Lines**		
Human: HEK 293T	Human: HEK 293T	Human: HEK 293T
Human: Huh7	Human: Huh7	Human: Huh7
Human: PH5CH	Human: PH5CH	Human: PH5CH
**Experimental Models: Organisms/Strains**		
Mouse: *Alb-CreERT2*	Gift from Dr. Daniel Metzger and Pierre Chambon, IGBM, Illkirch, France	N/A
Mouse: *Ns5aTg*	Gift from Dr. Ratna Ray (Saint Louis Univ.)	N/A
Mouse: *NOD.cg-Prkdc^{scid}Il2rg^{tm1Wjl}/SZJ*	Jackson Laboratory	005557
Mouse: FRG		
**Recombinant DNA**		
pPAX2	Addgene	Plasmid #12260
pMD2.G	Addgene	Plasmid #12259
scrambled shRNA	Addgene	Plasmid #1864
pRL c-Myc 3’UTR	Addgene	Plasmid #14806
pRetrosuper Myc shRNA	Addgene	Plasmid #15662
MSI2 Lentiviral Vector	AMB	LV712475
MSI2 shRNA Lentiviral Vector	AMB	I014309
pSK-ML	Gift from Dr. Anne Willis (Leicester Univ.)	N/A
pSK-GAP-L	Gift from Dr. Anne Willis (Leicester Univ.)	N/A
pRF	Gift from Dr. Anne Willis (Leicester Univ.)	N/A
pRMF	Gift from Dr. Anne Willis (Leicester Univ.)	N/A
pRHCV	Gift from Dr. Anne Willis (Leicester Univ.)	N/A
pRAF	Gift from Dr. Anne Willis (Leicester Univ.)	N/A
pRBF	Gift from Dr. Anne Willis (Leicester Univ.)	N/A
pRJ1..1020F	This study	N/A

pRJ1..397F	This study	N/A
pRJ520..1020F	This study	N/A
**Software and Algorithms**		
GraphPad Prism 6	GraphPad Software	https://www.graphpad.
ImageJ	N/A	https://imagej.nih.gov/ij/
MetaMorph	Molecular Devices LLC	MS-MM
ELDA	N/A	http://bioinf.wehi.edu.a
STATA	Stata corp LP College Station	Version 11.0

Supplementary Table 6. Oligonucleotide sequences for qRT-PCR Sponge & shRNA targets

Synthetic oligonucleotides used to generate miR-7014-3P sponge

**Xba1_Sense**	5′-ctagataaGGAGAAAGTtcGTTGGTGTCcgatGGAGAAAGTtcGTTGGTGTCg ctagcataaGGAGAAAGTtcGTTGGTGTCcgatGGAGAAAGTtcGTTGGTGTCg aattcataaGGAGAAAGTtcGTTGGTGTCcgatGGAGAAAGTtcGTTGGTGTCg-3′
**Antisense_ BamHI**	5′-gatccGACACCAACgaACTTTCTCCatcgGACACCAACgaACTTTCTCCttatgaatt cGACACCAACgaACTTTCTCCatcgGACACCAACgaACTTTCTCCttatgctag cGACACCAACgaACTTTCTCCatcgGACACCAACgaACTTTCTCCttat-3′

Synthetic oligonucleotides of miR-7014-3P mimic and miR-ctrl

**mmu-miR-7014-3p**	5′-CCUCUUUCAUACCAACCACAG-3′
**cel-mir-67 (miR-ctrl)**	5′-UCUACUCUUUCUAGGAGGUUGUGA-3′

Sequences of primer sets employed for qPCR analyses of gene expression

**JUN**	Fwd: 5′-GCTGCTCTGGGAAGTGAGTT-3′
	Rev: 5′-TTTCTCTAAGAGCGCACGCA-3′
**VEGFA**	Fwd: 5′-CTTGCCTTGCTGCTCTACCT-3′
	Rev: 5′-GCAGTAGCTGCGCTGATAGA-3′
**APP**	Fwd: 5′-GCCCTTCTCGTTCCTGACAA-3′
	Rev: 5′-GTCATCCTCCTCCGCATCAG-3′
**IL1RL1**	Fwd: 5′-CTGTGGGCAGAAAGTTGAGG-3′
	Rev: 5′-CGACAGTGAAGGTCACCACA-3′
**LAMB1**	Fwd: 5′-CGGCTTTCAAACAAAAGAGGCG-3′
	Rev: 5′-GTTTAGCCTTGGGAGGAACAGA-3′
**SQSTM1**	Fwd: 5′-GGATGTGGCTGTAACCTGCT-3′
	Rev: 5′-CTCTTTCAGGGACAGGCTGG-3′
**MSLN**	Fwd: 5′-ACGCACTCCTCTTTCTGCC-3′
	Rev: 5′-TGTGTAGATCCCAGGGAGGG-3′
**MYC**	Fwd: 5′-TTCGGGTAGTGGAAAACCAG-3′
	Rev: 5′-CAGCAGCTCGAATTTCTTCC-3′
**MT2A**	Fwd: 5′-TCCTGCAAATGCAAAGAGTGC-3′
	Rev: 5′-TAGCAAACGGTCACGGTCA-3′
**FN1**	Fwd: 5′-AACAAGCATGTCTCTCTGCCA-3′
	Rev: 5′-GCAATGTGCAGCCCTCATTT-3′
**RNASE4A**	Fwd: 5′-CAGAGGACCCATTCATTGCT-3′
	Rev: 5′-GAAGCGCTTGCAGTGATACA-3′
**CTH**	Fwd: 5′-TTCTCGCCTGATCCTTCTGT-3′
	Rev: 5′-CTTGAACGTGGTGGACAGTG-3′
**CMSS1**	Fwd: 5′-AGGAACTCCGGGGAGAATTA-3′
	Rev: 5′-AAGGCCCAGTTTCAAGGATT-3′
**NDRG1**	Fwd: 5′-ACAACCCTGAGATGGTGGAG-3′
	Rev: 5′-TGTGGACCACTTCCACGTTA-3′
**MSI2**	Fwd: 5′-GTATTAGGTCAGCCCCACCA-3′
	Rev: 5′-ACAAAGCCAAACCCTCTGTG-3′
**ACTB**	Fwd: 5′-CACTCTTCCAGCCTTCCTTC-3′
	Rev: 5′-GTACAGGTCTTTGCGGATGT-3′
	Fwd: 5′-GAGTCAACGGATTTGGTCGT-3′

GAPDH	Rev: 5'-GATCTCGCTCCTGGAAGATG-3'
Gapdh	Fwd: 5'-AACTTTGGCATTGTGGAAGG-3'
	Rev: 5'-ACACATTGGGGGTAGGAACA-3'
Actb	Fwd: 5'-AGCCATGTACGTAGCCATCC-3'
	Rev: 5'-CTCTCAGCTGTGGTGGTGAA-3'
Myc	Fwd: 5'-GCCCAGTGAGGATATCTGGA-3'
	Rev: 5'-ATCGCAGATGAAGCTCTGGT-3'
Vegfa	Fwd: 5'-CAGGCTGCTGTAACGATGAA-3'
	Rev: 5'-TTTCTTGCGCTTTCGTTTTT-3'
Jun	Fwd: 5'-AAAACCTTGAAAGCGCAAAA-3'
	Rev: 5'-CGCAACCAGTCAAGTTCTCA-3'
hsa-miR-22	Fwd: 5'-AGTTCTTCAGTGGCAAGCTTTA-3'
mmu-miR-7014-3P	Fwd: 5'-CCTCTTTCATACCAACCACAG-3'

Sequences of primer sets employed for dual luciferase reporter construction

SpeI-JUN1-F	5'-CTGACTAGTGCTCAGAGTTGCACTGAGTGTGGCTGAAGCAG-3'
SpeI-JUN530-F	5'-GCACTAGTCCTCCCCCTAGCAGCGGAGGAGG-3'
EcoRI-JUN1020-R	5'-GCATCGAATTCTGAGGGCATCGTCATAGAAGGTCGTTTCCATCTTTGC-3'
SpeI-VEGFA 1-F	5'-GCTTACTAGTGCGGAGGCTTGGGGCAGCC-3'
EcoRI-VEGFA 497-R	5'-GACTGGAATTCCGCGACTGGTCAGCTGCGGGA-3'

Target sequence for shRNA constructs targeting MSI2

Name	Clone ID	Target sequence
HUMAN shMSI2-1	V3SVHS00_5813953	CATGGGGATGCTGGGATAT
HUMAN shMSI2-2	V3SVHS00_5363372	CACAGTAGTGGAAGATGTA
HUMAN shMSI2-3	V3SVHS00_7912687	CTTTGTCACTTTTGAGAAT
Mouse shMsi2-1	TRCN0000062812	CCCAGCTTAATATCTAGTTAA
Mouse shMsi2-2	TRCN0000071973	AGATAGCCTTAGAGACTATTT

Table S6. Sequence of oligonucleotides employed in this study

TableS7_Clinicopathological parameters of the HCC patients with immunoreactivity score (IRS)

Frequency of Staining (FS) =
0=Negative
1 =positive staining in 0.01-1.0% 1-25%
2 =positive staining in 1.01-2.0% 26-50%
3 =positive staining in 2.01-3.0% 51-75%
4 =positive staining in 3.01% -....... 76-100%

IRS= Immuno reactivity score=FS X IS

Intensity of Staining (IS) = 0=negative; 1=weak staining; 2=moderate staining; 3=strongly staining

																						Percent of co-staining of MSI2 and MYC	
Method_staining	Protein	Patients	FS_nontumor	IS_nontumor	IRS_nontumor		FS_tumor	IS_tumor	IRS_tumor	Protein	FS_nontumor	IS_nontumor	IRS_nontumor	FS_tumor	IS_tumor	IRS_tumor	Age	Sex	Serum HBsAg	Ethnicity	Tumor Stage	Non-Tumor samples	Tumor samples
Immunofloursecence 20X	MYC	1	1	1	1		2	1	2	MSI2	0	0	0	1	2	2	45	M	0	Hispanic	I	0	10
Immunofloursecence 20X	MYC	2	1	1	1		3	2	6	MSI2	1	1	1	2	2	4	74	F	0	Black	IIIA	10	50
Immunofloursecence 20X	MYC	3	1	1	1		2	1	2	MSI2	1	1	1	3	1	3	52	M	+	Black	II	8	32
Immunofloursecence 20X	MYC	4	1	1	1		3	1	3	MSI2	0	0	0	2	2	4	45	M	0	White	II	0	41
Immunofloursecence 20X	MYC	5	1	1	1		2	1	2	MSI2	0	0	0	2	1	2	62	M	0	Hispanic	I	0	22
Immunofloursecence 20X	MYC	6	2	2	4		3	2	6	MSI2	1	1	1	3	2	6	54	F	0	Black	IIIB	12	45
Immunofloursecence 20X	MYC	7	1	1	1		3	1	3	MSI2	1	1	1	3	1	3	50	M	+	White	II	6	18
Immunofloursecence 20X	MYC	8	1	1	1		2	1	2	MSI2	1	1	1	3	1	3	44	F	+	Hispanic	II	5	17
Immunofloursecence 20X	MYC	9	1	1	1		2	2	4	MSI2	1	1	1	3	2	6	57	M	0	White	IIIB	9	47
Immunofloursecence 20X	MYC	10	1	1	1		4	2	8	MSI2	1	1	1	3	2	6	52	M	+	White	IIIB	10	75
Immunofloursecence 20X	MYC	11	1	1	1		3	2	6	MSI2	1	1	1	3	2	6	54	F	+	Black	IVA	9	65
Immunofloursecence 20X	MYC	12	1	1	1		3	2	6	MSI2	0	0	0	2	1	2	45	M	0	Black	II	0	47
Immunofloursecence 20X	MYC	13	1	1	1		3	1	3	MSI2	0	0	0	3	1	3	52	F	+	White	II	0	22
Immunofloursecence 20X	MYC	14	1	1	1		3	2	6	MSI2	1	1	1	2	2	4	61	M	0	White	I	12	48
Immunofloursecence 20X	MYC	15	1	1	1		3	2	6	MSI2	0	0	0	3	2	6	52	F	+	Black	IIIC	0	55
Immunofloursecence 20X	MYC	16	1	1	1		3	1	3	MSI2	0	0	0	2	1	2	51	M	0	White	II	0	34
Immunofloursecence 20X	MYC	17	1	1	1		2	2	4	MSI2	1	1	1	3	2	6	59	F	+	Black	IIIB	10	48
Immunofloursecence 20X	MYC	18	2	1	2		3	3	9	MSI2	0	0	0	3	2	6	49	M	0	Hispanic	II	0	42
Immunofloursecence 20X	MYC	19	1	1	1		3	2	6	MSI2	0	0	0	2	2	4	79	F	+	Black	II	0	45
Immunofloursecence 20X	MYC	20	1	1	1		3	2	6	MSI2	1	1	1	3	2	6	47	F	+	Black	IIIA	10	57
Immunofloursecence 20X	MYC	21	1	1	1		3	2	6	MSI2	1	1	1	3	2	6	72	M	+	White	IIIB	10	63
Immunofloursecence 20X	MYC	22	2	1	2		2	3	6	MSI2	1	1	1	2	2	4	64	F	+	White	II	8	58
Immunofloursecence 20X	MYC	23	1	1	1		3	2	6	MSI2	1	1	1	2	2	4	54	M	0	Black	IIIB	14	60
Immunofloursecence 20X	MYC	24	1	1	1		3	1	3	MSI2	1	1	1	2	1	2	50	F	+	Black	II	5	48
Immunofloursecence 20X	MYC	25	1	1	1		3	2	6	MSI2	1	1	1	2	2	4	65	F	+	Black	IIIA	10	56
Immunofloursecence 20X	MYC	26	1	1	1		3	3	9	MSI2	1	1	1	3	2	6	50	M	+	Black	IVB	4	65
Immunofloursecence 20X	MYC	27	1	1	1		2	2	4	MSI2	1	1	1	2	2	4	54	M	0	Hispanic	IIIA	8	70
Immunofloursecence 20X	MYC	28	1	1	1		3	1	3	MSI2	1	1	1	3	1	3	43	M	+	Hispanic	II	2	55
Immunofloursecence 20X	MYC	29	1	1	1		2	2	4	MSI2	1	1	1	2	1	2	76	M	0	Black	II	5	34
Immunofloursecence 20X	MYC	30	1	1	1		3	2	6	MSI2	1	1	1	2	3	6	52	M	0	Black	IVB	14	72

Charecteristi	Value	
Age	55 +9.7	
Gender		
Male	18	
Female	12	
	30	
Serum HBsAg		
Negative	14	
Positive	16	
	30	
Ethnicity		
White	9	
Black	15	
Hispanic	6	
	30	
Tumor stage		
I-II	16	
IIIA-IV	14	
	30	

- **CONTACT FOR REAGENT AND RESOURCE SHARING**

Contact for Reagent and Resource Sharing

Further information and requests for resources and reagents should be directed to and will be fulfilled by the Lead Contact, Keigo Machida (keigo.machida@med.usc.edu).

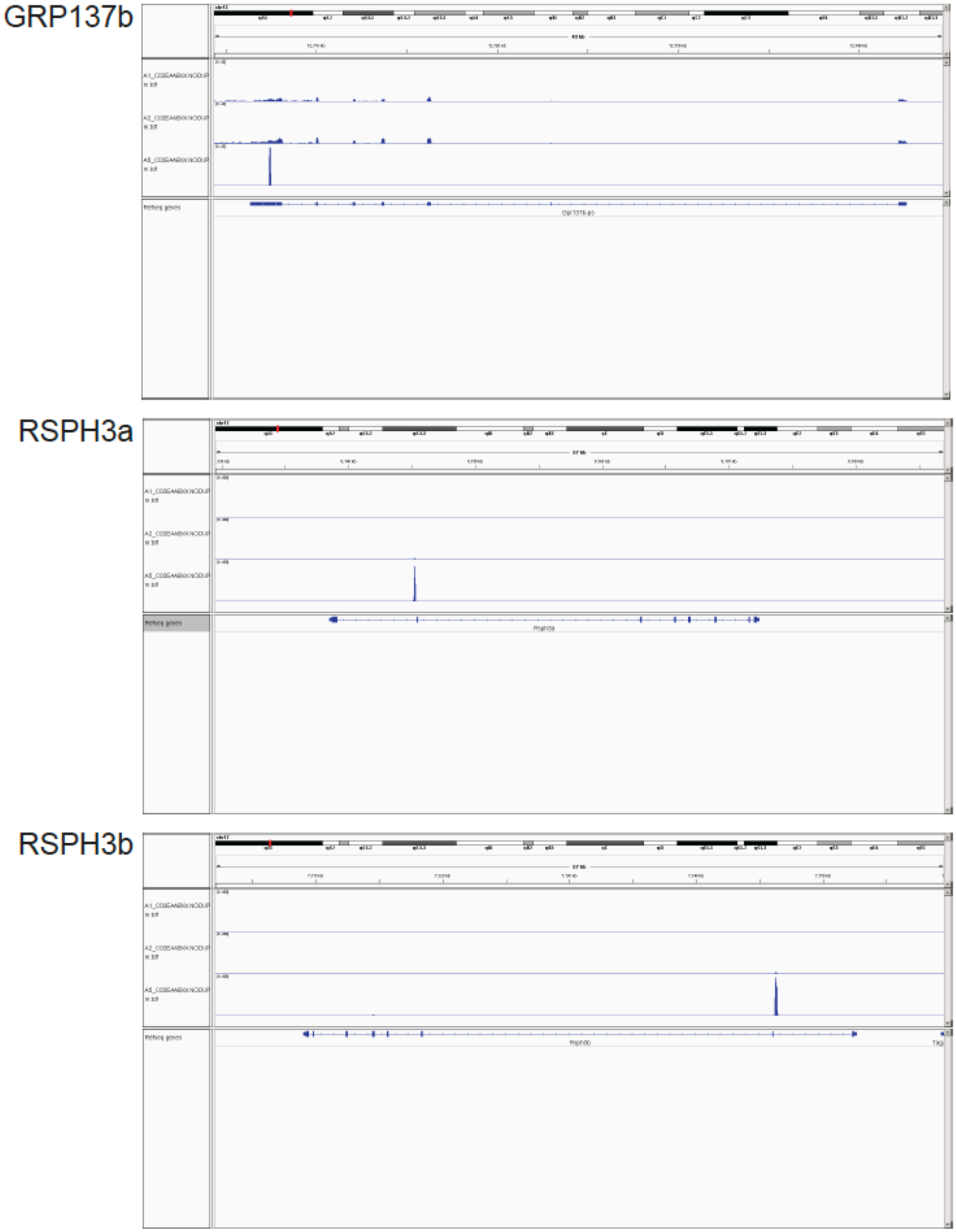

Figure S1
Yeh and Siddique et al.

Extended Data Fig. 1. RIP-seq analyses demonstrate that MSI2 is enriched in mRNAs of cellular genes. GRP137b and RSPH3a/b were among those cellular targets detected from MSI2 peaks.

MSI2-target IRES-containing gene list in current study

NCBI accession #	Gene name	IRESbase ID	IRES minimal length	Reference
NM_002228.4	JUN		277bp	PNAS 2012 E2875–E2884.
NM_000484	APP	hsa_ires_00684.1	147bp	Nucleic acids research, 2008, 36(21): 6835-6847.
NM_001171623	VEGFA	hsa_ires_00616.1	294bp	Molecular & Cellular Biology, 1998, 18(11):6178-6190.
NM_001171630		hsa_ires_00643.1	393bp	Oncogene, 1998, 17(2):227.
NM_003856	IL1RL1	hsa_ires_00584.1	237bp	Biochimica Et Biophysica Acta, 2016, 1859(7):848-859.
NM_002291	LAMB1	hsa_ires_00587.1	180bp	Nucleic Acids Research, 2007, 35(8):2473-82.
NM_001142298	SQSTM1	hsa_ires_00398.1	174bp	Science, 2016, 351(6270):240-240.
NM_002467	MYC	hsa_ires_00598.1	367bp	Oncogene, 1998, 16(3):423-8
NM_013404	MSLN	hsa_ires_00228.1	174bp	Science, 2016, 351(6270):240-240.
NM_005953.3	MT2A	hsa_ires_00294.1	174bp	Science, 2016, 351(6270):240-240.
NM_054034.2	FN1	hsa_ires_00521.1	174bp	Science, 2016, 351(6270):240-240.
NM_002937.4	RNASE4	hsa_ires_00490.1	174bp	Science, 2016, 351(6270):240-240.
NM_001902.5	CTH	hsa_ires_00001.1	174bp	Science, 2016, 351(6270):240-240.
NM_001167924.1	CMSS1	hsa_ires_00568.1	174bp	Science, 2016, 351(6270):240-240.
NM_006096.3	NDRG1	hsa_ires_00244.1	174bp	Science, 2016, 351(6270):240-240.

Figure S1B. **Yeh and Siddique *et al.***

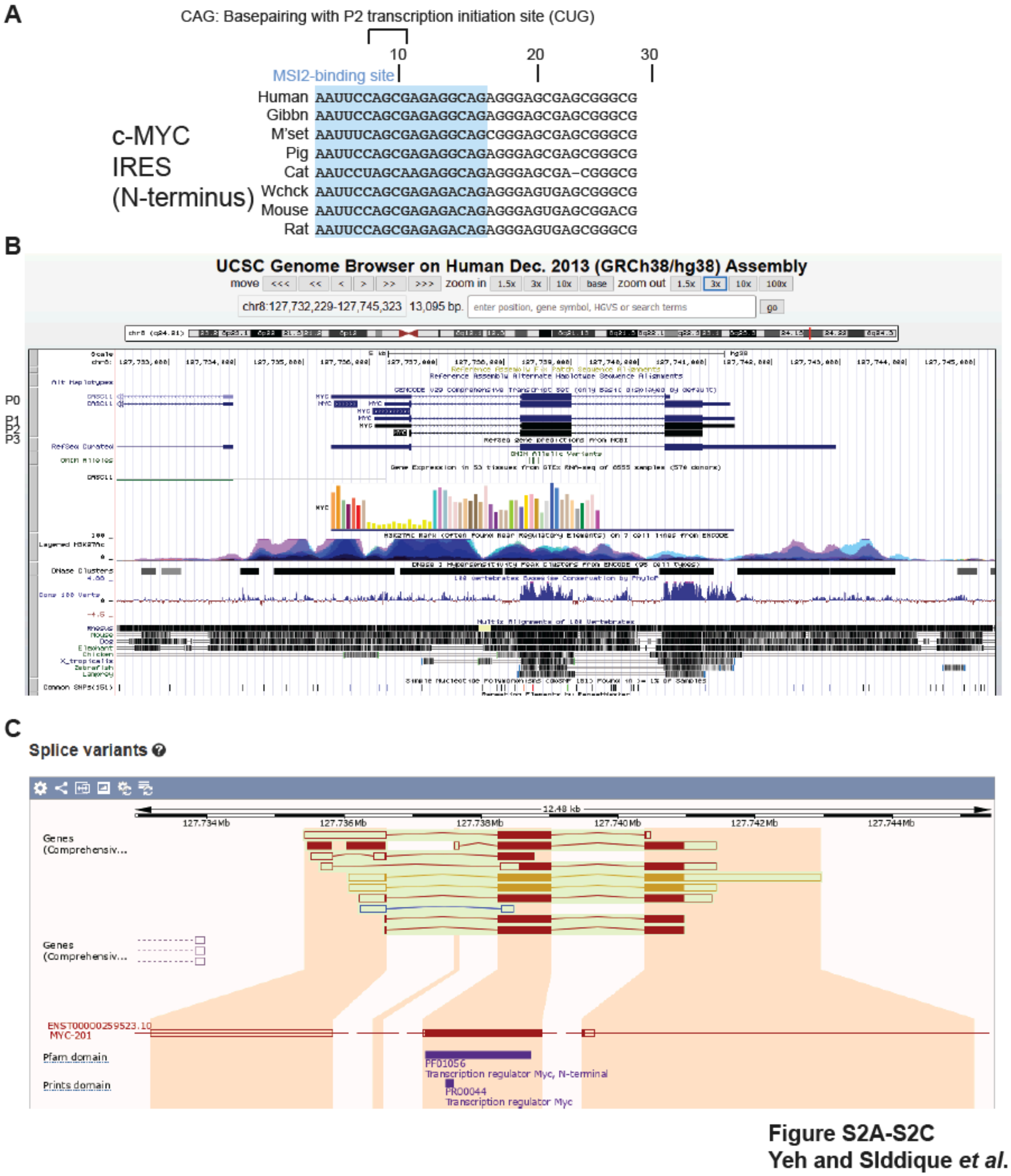

Extended Data Fig. 2A-S2C. UCSC Genome Browser for MYC loci and analyses of MSI2-binding motifs. For motif column as calculated in G (Supplementary Table 2) and 25bp +/- across this summit seq was extracted from Mm9. motif was identified using MEME algorithm (http://nar.oxfordjournals.org/content/37/suppl_2/W202.full) .

MEME-ChIP Logo

MEME-ChIP

Motif Analysis of Large Nucleotide Datasets

If you use MEME-ChIP in your research, please cite the following paper:
Philip Machanick and Timothy L. Bailey, "MEME-ChIP: motif analysis of large DNA datasets", *Bioinformatics*, 2712, 1696-1697, 2011. [full text]

Motifs | Programs | Input Files | Program information

Description

jasper core and uniproe

Motifs

The significant motifs (E-value ≤ 0.05) found by the programs MEME, DREME and CentriMo; clustered by similarity and ordered by E-value.

Expand All Clusters   Collapse All Clusters

Motif Found	Discovery/ Enrichment Program	E-value	Known or Similar Motifs	Distributio
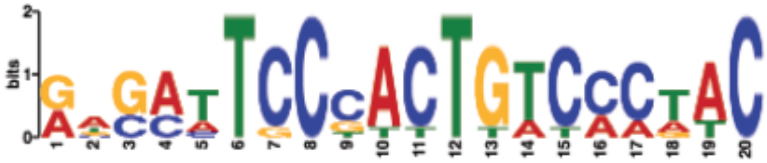	MEME	7.1e-008		Not Centrally Enriched

Reverse Complement ⇆

Motif Found	Discovery/ Enrichment Program	E-value	Known or Similar Motifs	Distribution	SpaM FIMO
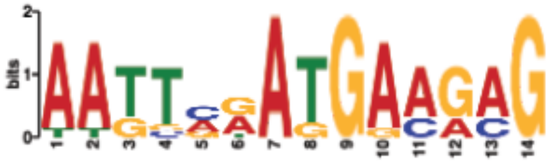	MEME	9.4e-003	Bbx_primary (UP00012_1)	Not Centrally Enriched	• Mo Sp Ar • Mo Si GF

Reverse Complement ⇆

Using RNA binding protein data base- Ray 2013 (http://www.nature.com/nature/journal/v499/n7457/full/nature12311.html?WT.ec_id=NATURE-20130711).

Extended Data Fig. 2D. MSI2-binding motif analyses by use of MEME-ChIP.

PROGRAMS

Command	Running Time	Status	Outputs
**fasta-get-markov** -nostatus -m 1 < ./Extract_Genomic_DNA_fc2_157.fasta 1> ./background	0.02s	Success	• Background
**getsize** ./Extract_Genomic_DNA_fc2_157.fasta 1> $metrics	0.02s	Success	
**fasta-most** -min 50 < ./Extract_Genomic_DNA_fc2_157.fasta 1> $metrics	0.08s	Success	
**fasta-center** -len 100 < ./Extract_Genomic_DNA_fc2_157.fasta 1> ./seqs-centered	0.07s	Success	• seqs-centered
**fasta-dinucleotide-shuffle** -f ./seqs-centered -t -dinuc 1> ./seqs-shuffled	0.14s	Success	• seqs-shuffled
**meme** ./seqs-centered -oc meme_out -dna -mod zoops -nmotifs 5 -minw 6 -maxw 20 -bfile ./background -minsites 2 -time 5082 -revcomp -nostatus	51.04s	Success	• MEME HTML<br>• MEME text<br>• MEME XML
**dreme** -v 1 -oc dreme_out -p ./seqs-centered -n ./seqs-shuffled -png -t 7119 -e 0.05	1.11s	Success	• DREME HTML<br>• DREME text<br>• DREME XML
**centrimo** -seqlen 50 -verbosity 1 -oc centrimo_out -bgfile ./background -local -score 5.0 -maxreg 200 -ethresh 10.0 ./Extract_Genomic_DNA_fc2_157.fasta meme_out/meme.xml db/uniprobe_mouse.meme db/JASPAR_CORE_2014.meme	3.20s	Warnings	• CentriMo HTML
**tomtom** -verbosity 1 -oc meme_tomtom_out -min-overlap 5 -dist pearson -evalue -thresh 1 -no-ssc -bfile ./background meme_out/meme.xml db/uniprobe_mouse.meme db/JASPAR_CORE_2014.meme	22.95s	Success	• TOMTOM HTML<br>• TOMTOM text<br>• TOMTOM XML
**tomtom** -verbosity 1 -text -thresh 0.1 ./combined.meme ./combined.meme 1> ./motif_alignment.txt	0.09s	Warnings	• Motif Alignment
**spamo** -verbosity 1 -oc spamo_out_1 -bgfile ./background -primary 1 ./Extract_Genomic_DNA_fc2_157.fasta meme_out/meme.xml meme_out/meme.xml dreme_out/dreme.xml db/uniprobe_mouse.meme db/JASPAR_CORE_2014.meme	1.06s	Warnings	• SpaMo HTML<br>• SpaMo XML

Command	Running Time	Status	Outputs
**spamo** -verbosity 1 -oc spamo_out_2 -bgfile ./background -primary 2 ./Extract_Genomic_DNA_fc2_157.fasta meme_out/meme.xml meme_out/meme.xml dreme_out/dreme.xml db/uniprobe_mouse.meme db/JASPAR_CORE_2014.meme	1.08s	Warnings	• SpaMo HTML<br>• SpaMo XML
**fimo** --parse-genomic-coord --verbosity 1 --oc fimo_out_1 --bgfile ./background --motif 1 meme_out/meme.xml ./Extract_Genomic_DNA_fc2_157.fasta	0.17s	Success	• FIMO GFF<br>• FIMO HTML<br>• FIMO Text
**fimo** --parse-genomic-coord --verbosity 1 --oc fimo_out_2 --bgfile ./background --motif 2 meme_out/meme.xml ./Extract_Genomic_DNA_fc2_157.fasta	0.17s	Success	• FIMO GFF<br>• FIMO HTML<br>• FIMO Text

INPUT FILES

Sequences

Database	Source	Sequence Count
Extract Genomic DNA fc2 157	Extract_Genomic_DNA_fc2_157.fasta	157

Motifs

Database	Source	Motif Count
uniprobe mouse	db/uniprobe_mouse.meme	386
JASPAR CORE 2014	db/JASPAR_CORE_2014.meme	593

MEME-ChIP version
4.10.0 (Release date: Wed May 21 10:35:36 2014 +1000)

Reference
Philip Machanick and Timothy L. Bailey, "MEME-ChIP: motif analysis of large DNA datasets", *Bioinformatics*, 2712, 1696-1697, 2011.

Command line summary

```
meme-chip -oc . -index-name meme-chip.html -time 300 -fdesc description -order 1 -db db/uniprobe_mouse.meme -db
```

Extended Data Fig. 2E. Large database list used for MSI2-binding motif analyses by use of MEME-ChIP.

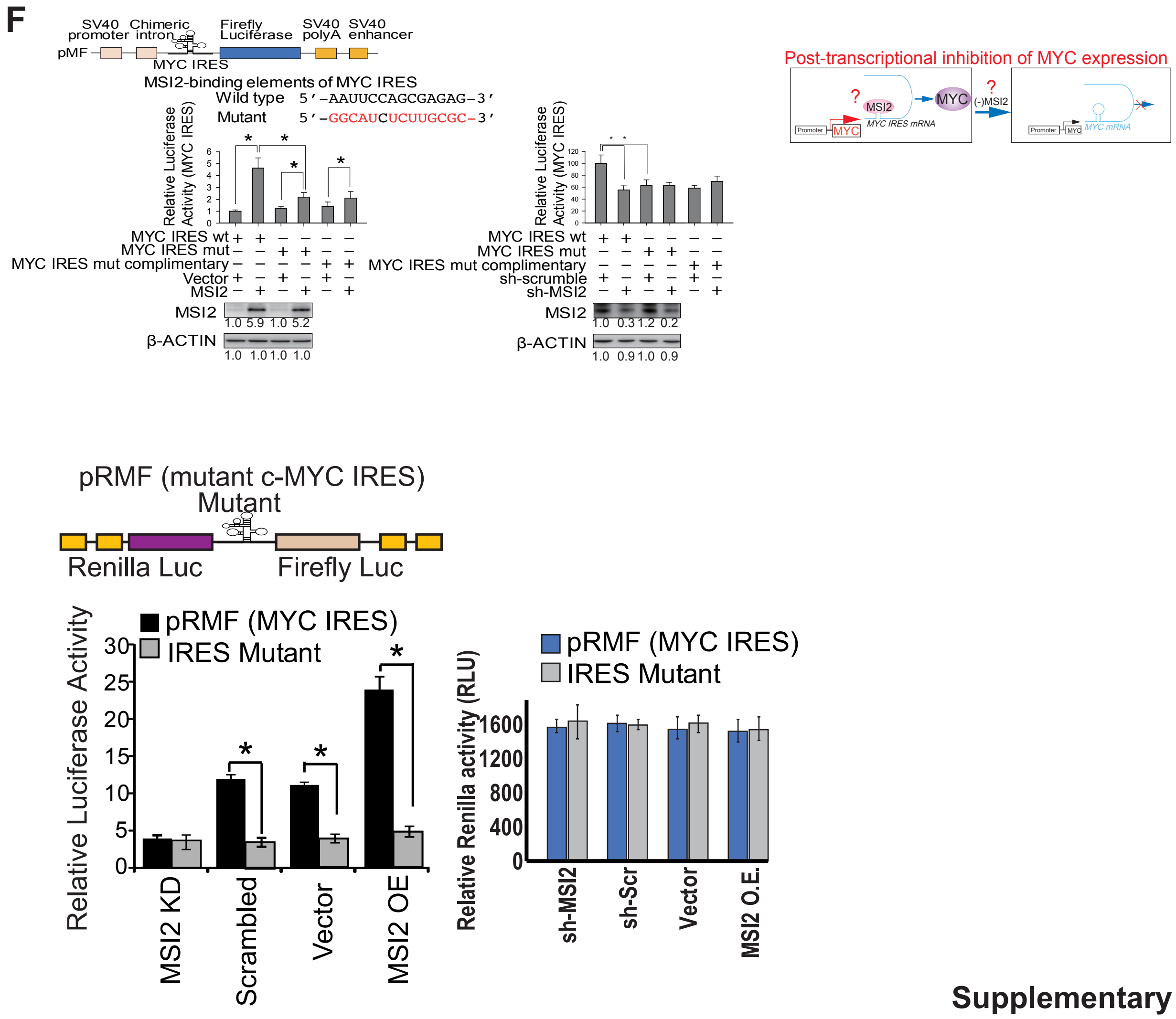

Supplementary Fig. 2F
Yeh and Zhao *et al.*

Extended Data Fig. 2F.
(**F, Top, Right**) MSI2 silencing reduces IRES activity for pRMF while MSI2 overexpressing increases IRES activity. (**F, Top**) Similar results were obtained with the single firefly luciferase construct without Renilla expression compared with relative luciferase activity normalized to Renilla luciferase values (**F, Bottom, Left**). The Dual luciferase constructs are temperamental and often result in changes in Renilla expression. We removed this confounding factor and repeated with single luciferase reporters. No significant difference was observed as well across the construct regarding Renilla expression (**F, Bottom, Right**).

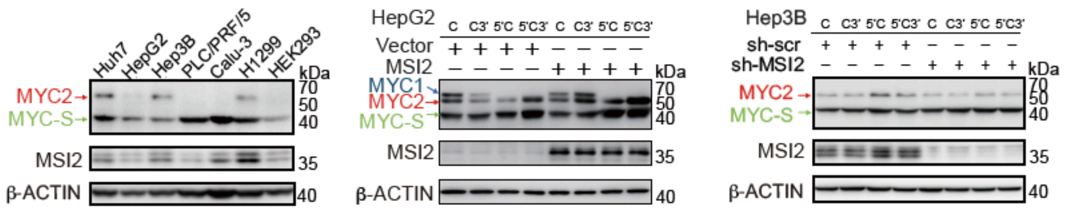

Supplemental Figure 2G
Yeh and Zhao

Extended Data Fig. 2G. The endogenous levels of MYC are positively correlated with MSI2 expression. (**G**) The endogenous levels of MYC and MSI2 protein in various cancer cell lines were examined by immunoblotting analysis (**Left panel**). HepG2 cells ectopically expressing either vector or MSI2 (**Middle panel**) and Hep3B cells stably transduced with sh-scramble or sh-MSI2 (**Right panel**) were co-transfected with the indicated MYC cDNA construct. Cell lysates were harvested 48 hr after transfection and subjected to immunoblotting with MYC and MSI2 antibodies.

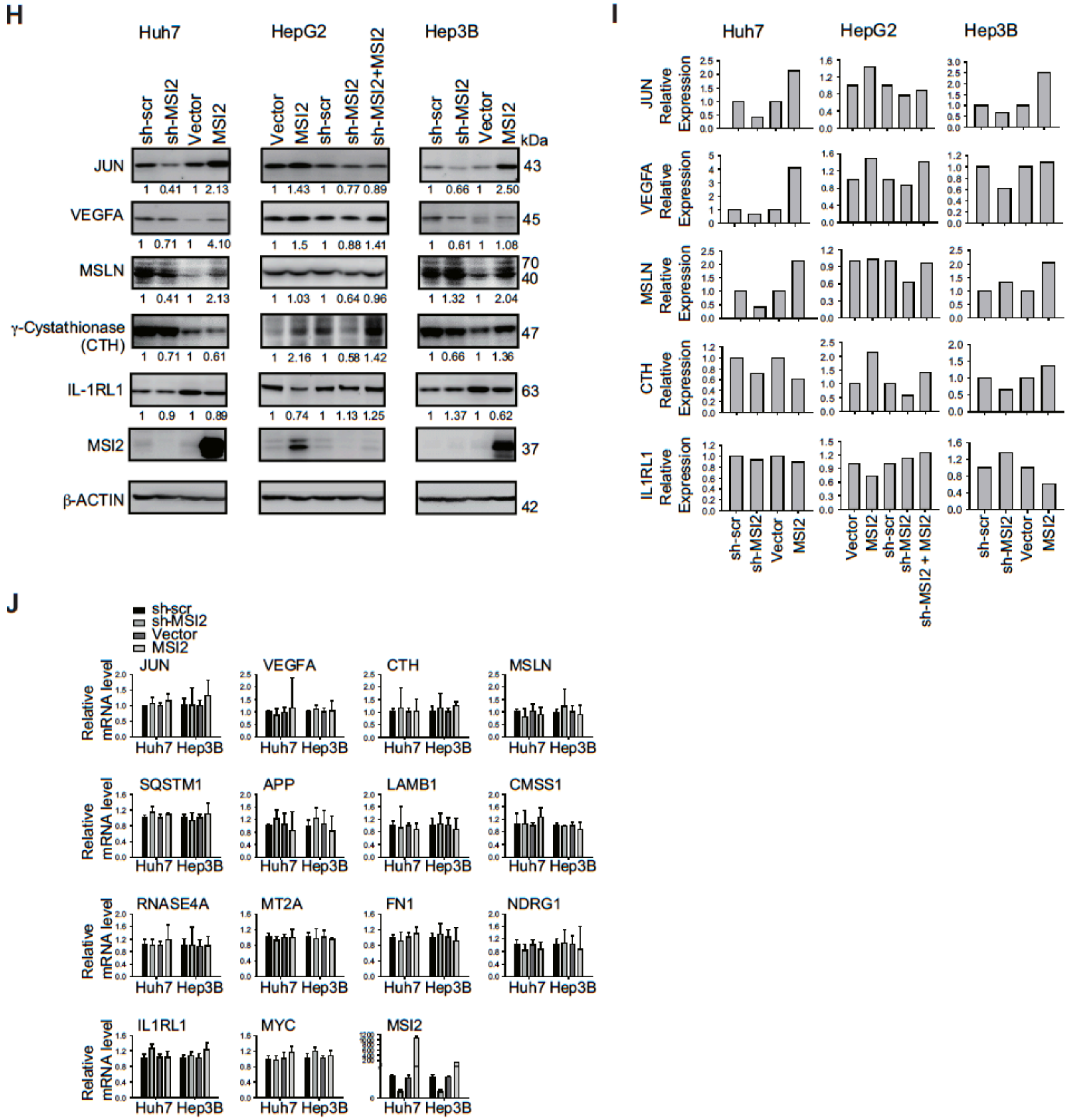

Supplemental Figure 2 H, I & J
Yeh and Zhao et al.

Extended Data Fig.2H, I & J. MSI2 regulates expression of cellular IRES-containing genes such as JUN and VEGFA. (**H**) MSI2 regulates translational activities of *JUN* and *VEGFA*. Protein levels of JUN and VEGFA as well as other MSI2-binding genes identified in our MSI2 RIP-seq results (*JUN, VEGFA, MSLN, CTH* and *IL1RL1*) were examined by immunoblotting with respective antibodies in MSI2 knockdown or/and overexpression HCC cell lines. (**I and J**) MSI2 silencing or overexpression does not affect transcription levels of *MSI2*-bound, cellular IRES-containing mRNAs. RT-qPCR analysis for a panel of 14 MSI2-interacting, cellular IRES-containing genes derived from this MSI2 RIP-seq study showed no significant change in relative mRNA levels in Huh7 and Hep3B cells upon MSI2 silencing or overexpression.

Motif	Sequence Name	Strand	Start	End	p-value	q-value	Matched Sequence
2	mm9_chr17	−	8145263	8145276	3.48e-08	0.000134	AATTCAATGAAGCG
2	mm9_chr1	−	46132978	46132991	3.48e-08	0.000134	AATTCAATGAAGCG
2	mm9_chr2	−	5300293	5300306	3.48e-08	0.000134	AATTCAATGAAGCG
2	mm9_chr4	+	45359460	45359473	6.55e-08	0.000152	AATTAGATGACGAG
2	mm9_chr3	+	120952830	120952843	6.55e-08	0.000152	AATTAGATGACGAG
2	mm9_chr7	+	127897651	127897664	1.53e-07	0.000295	AATTAGATGACAAG
2	mm9_chr17	−	46168981	46168994	2.77e-07	0.000458	AAGTCAAGGAAGAG
2	mm9_chr7	−	20489001	20489014	1.05e-06	0.00152	AAGCCAATGAAAAG
2	mm9_chr7	+	138105644	138105657	2.37e-06	0.00305	ATGTGGATGAAAAG
2	mm9_chr9	−	123371143	123371156	2.89e-06	0.00319	AATGATAGGAAGAG
2	mm9_chr9	+	77635672	77635685	3.03e-06	0.00319	TATTGGATGGAGAG
2	mm9_chr10	−	85809044	85809057	8.91e-05	0.0859	ATTGGGATGAAGAA

Fig. S2K. MSI2 Binding Motif was identified using MEME algorithm: For motif column as calculated in G and 25bp +/- across this summit seq was extracted from Mm9.

Motif was identified using MEME algorithm (http://nar.oxfordjournals.org/content/37/suppl_2/W202.full).

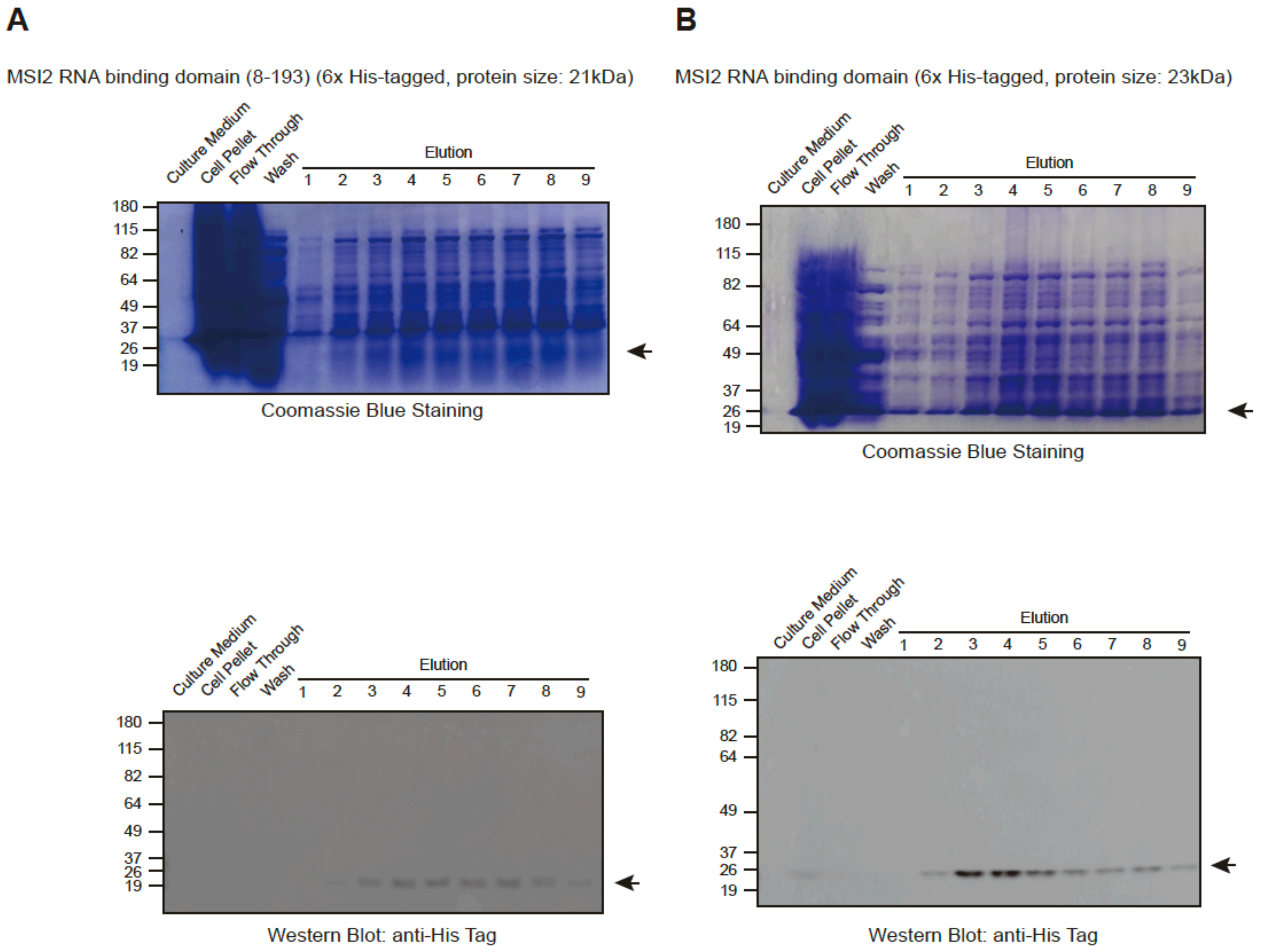

Figure S3A-S3B
Yeh and Siddique *et al.*

Extended Data Fig. 3A-3B. Production, purification and verification of His-tagged recombinant MSI2 and MSI2 RNA binding domain (RBD: aa8-193). Recombinant MSI2 (Left) and MSI2 RNA binding domain (RBD: aa8-193) (Right) with His-tag was purified from E. Coli with anti-His-conjugated beads and eluted by using imidazole. Fractionated samples in Elution #1-9 were loaded. Wash: represents washing buffer flowing through column. Coomassie Brilliant Blue staining (Top) and immunoblotting (bottom) were performed to verify recombinant proteins. His-tagged protein was detected in peak fractions between #3 to 8 for MSI2 (Left) and MSI2 RNA binding domain (RBD: aa8-193) (Right).

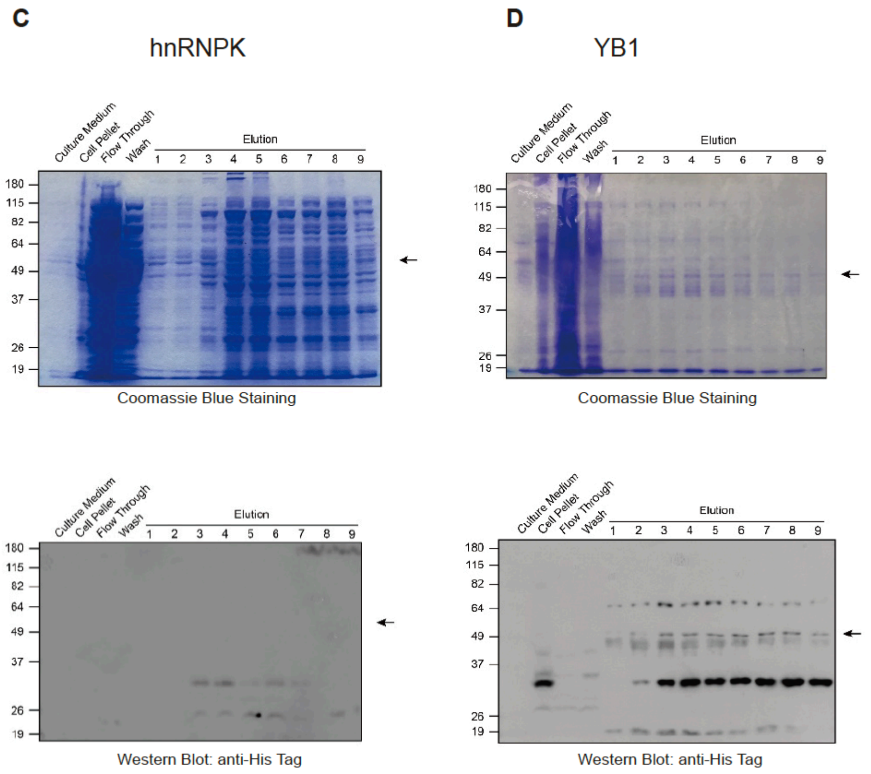

Figure S3C-S3D
Yeh and Siddique *et al.*

Extended Data Fig. 3C-S3D. Production, purification and verification of His-tagged of recombinant hnRNPK and YB1. Recombinant hnRNPK (Left) and YB1 (Right) with His-tag was purified from E. Coli with anti-His-conjugated beads and eluted by using imidazole. Fractionated samples in Elution #1-9 were loaded. Wash: represents washing buffer flowing through column. Coomassie Brilliant Blue staining (Top) and immunoblotting (bottom) were performed to verify recombinant proteins. His-tagged protein was detected in peak fractions between #3 to 6 for hnRNPK (Left) and #3 to 9 for YB1 (Right).

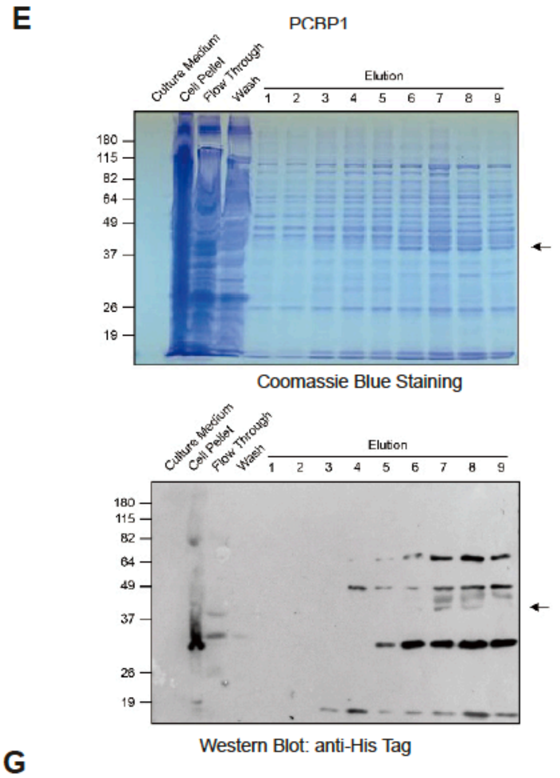

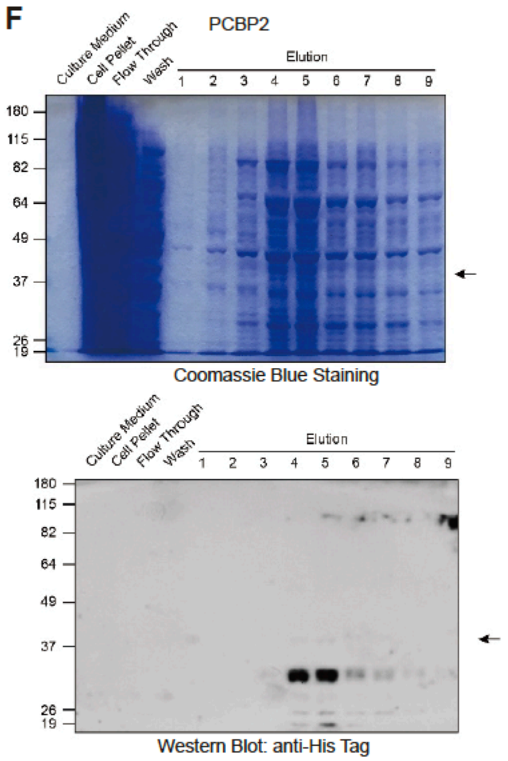

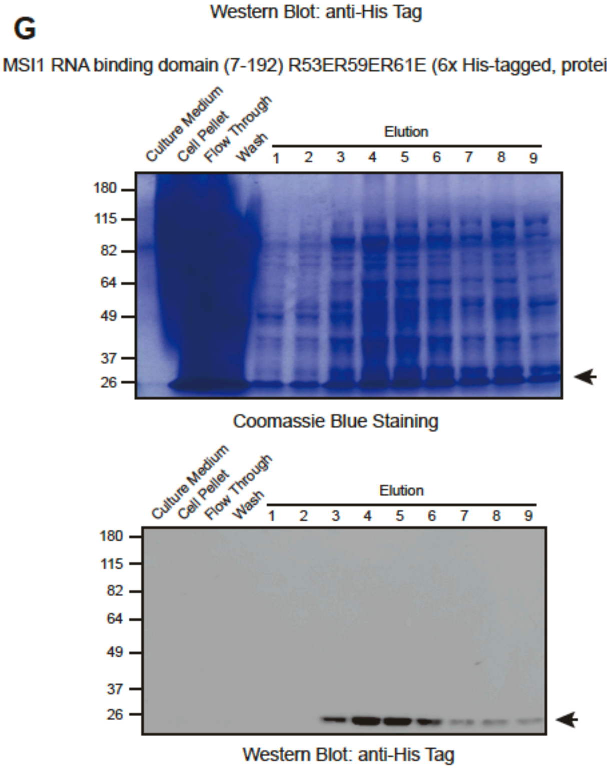

Figure S3E-S3G
Yeh and Siddique *et al.*

Extended Data Fig. 3E-S3G. Production, purification and verification of His-tagged of recombinant PCBP12 and MSI1 RNA binding domain (RBD: aa7.aa192 with mutations containing R53E，R59E and R61).

H

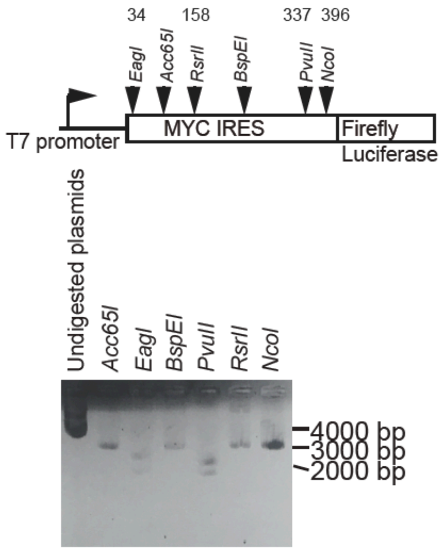

I

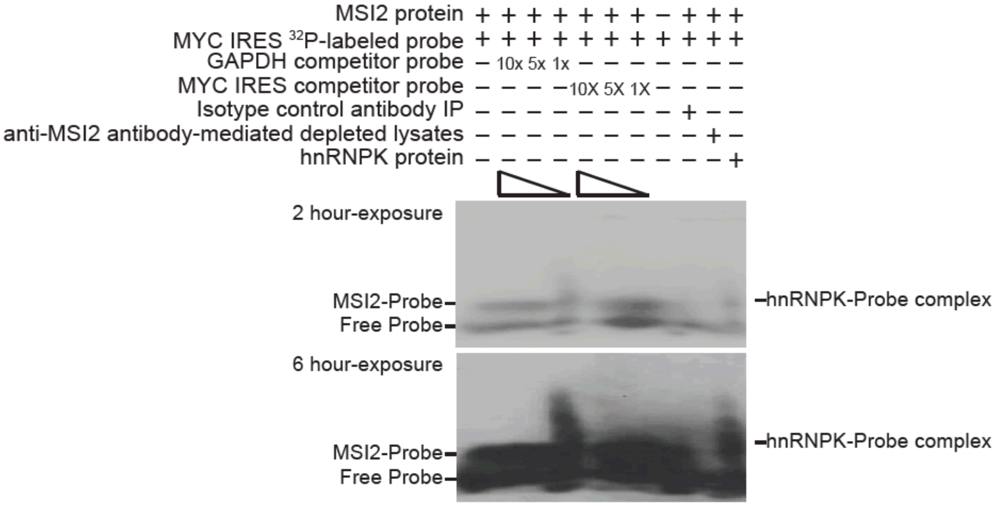

Figure S3H-S3I
Yeh and Siddique et al.

Extended Data Fig. 3H-S3I. DNA template preparation for in vitro transcription and EMSA assays to detect MSI2-MYC IRES RNA interaction. (H) DNA template preparation for in vitro transcription for RNA probe preparation in EMSA. (I) EMSA assays to detect MSI2-MYC IRES RNA interaction. The specificity of MSI2-MYC IRES was determined by using excessive amount of cold probes.

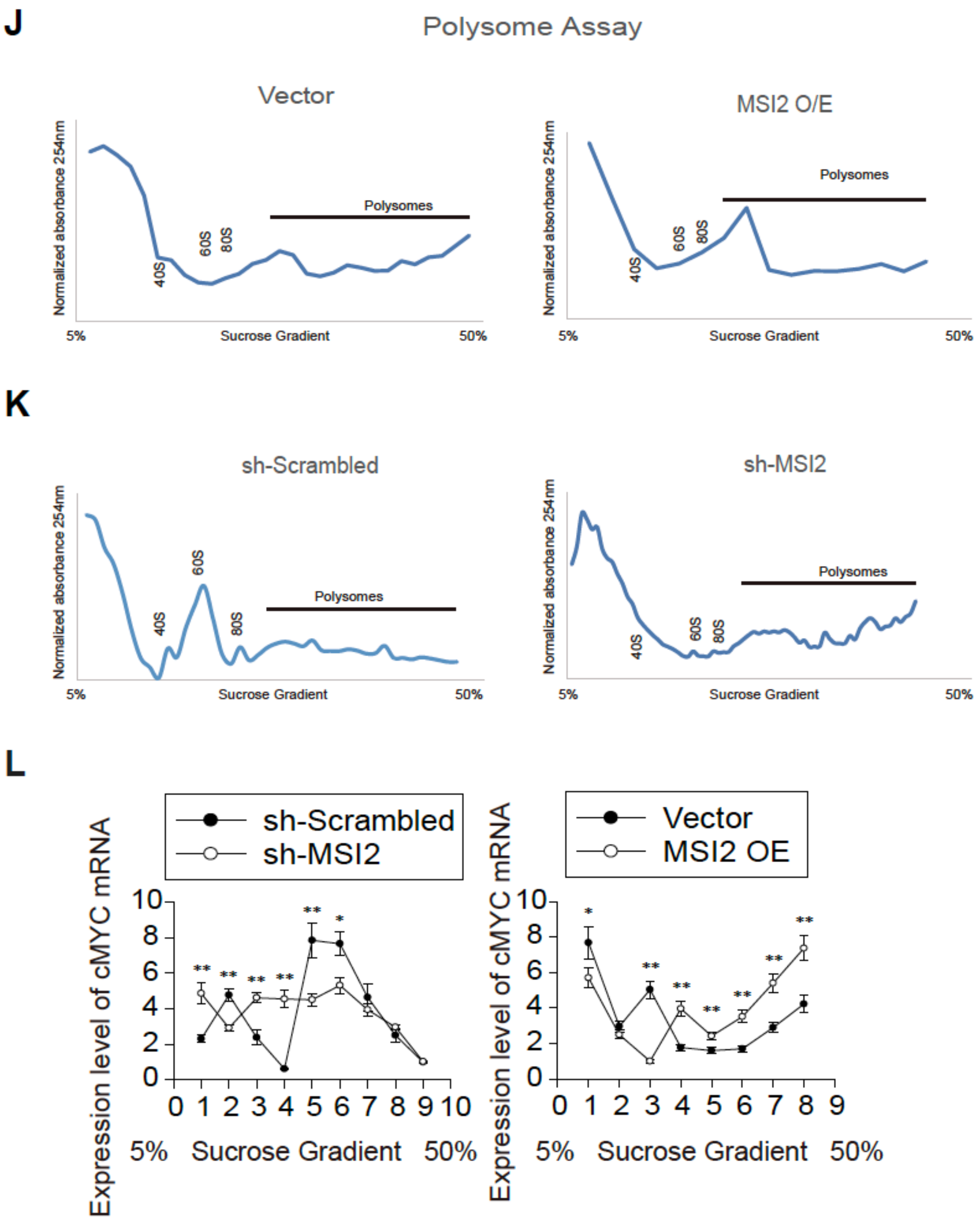

Figure S3J-S3L
Yeh and Siddique *et al.*

Extended Data Fig. 3J-S3L. Polysome assays revealed ribosome binding patterns were shifted by MSI2. Representative absorbance (OD254 nm) for each sucrose fraction was determined in polysome assay. (**J**) MSI2 overexpression increased enrichment at 80S peak. (**K**) MSI2 knockdown reduced enrichment at 80S peak. (**L**) Huh7 cells stably expressing sh-scramble or sh-MSI2 (**left**); Huh7 cells transfected with vector or MSI2 for 48 hr (**right**) were polysomes fractionated on sucrose gradients. Total RNA purified from each fraction was subjected to RT-qPCR analysis. The mRNA level of MYC in sh-scramble or sh-MSI2 cells (**left**); Vector or MSI2 overexpression cells (**right**) was determined, respectively. Data represent mean± S.D. from three independent experiments. *P<0.05; **P<0.01.

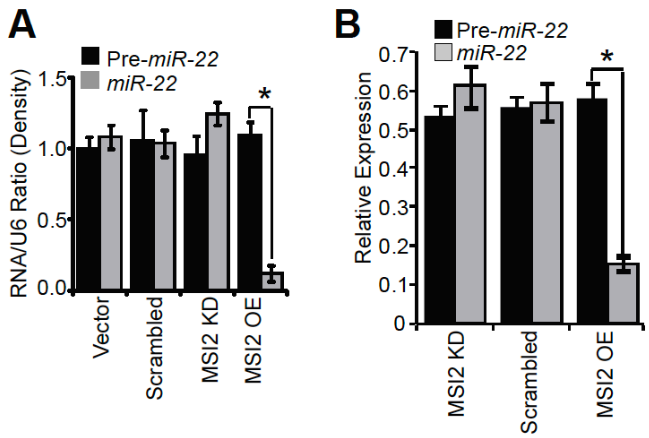

Figure S4A-S4B
Yeh and SIddique et al.

Extended Data Fig. 4A-4B. Repression of miR-22 by MSI2 overexpression. (**A**) Quantitative densitometry graph obtained from Northern blots of Fig. 4a and b. (**B**) RT-qPCR analysis of MSI2 Knockdown or overexpressing-Huh7 cells.

C Human *MYC* 3'UTR has one *miR-22* target site

http://34.236.212.39/microrna/getMrna.do?gene=4609&utr=15329&organism=9606#

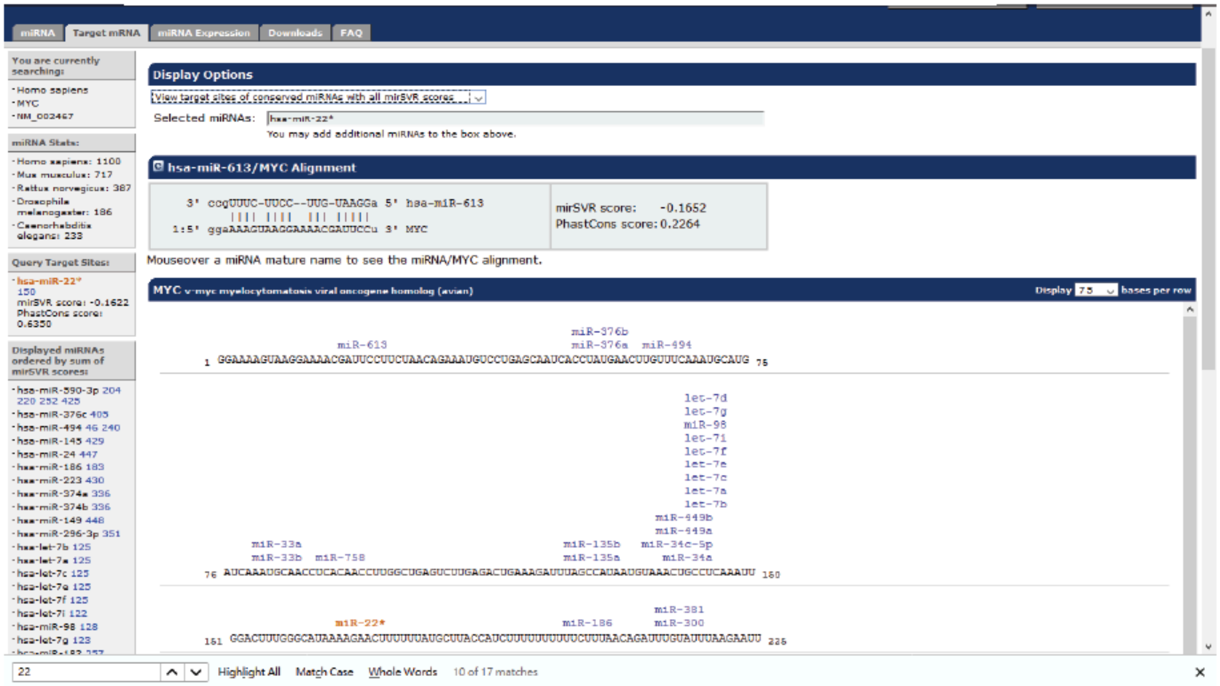

http://34.236.212.39/microrna/getMrna.do?gene=4609&utr=15329&organism=9606

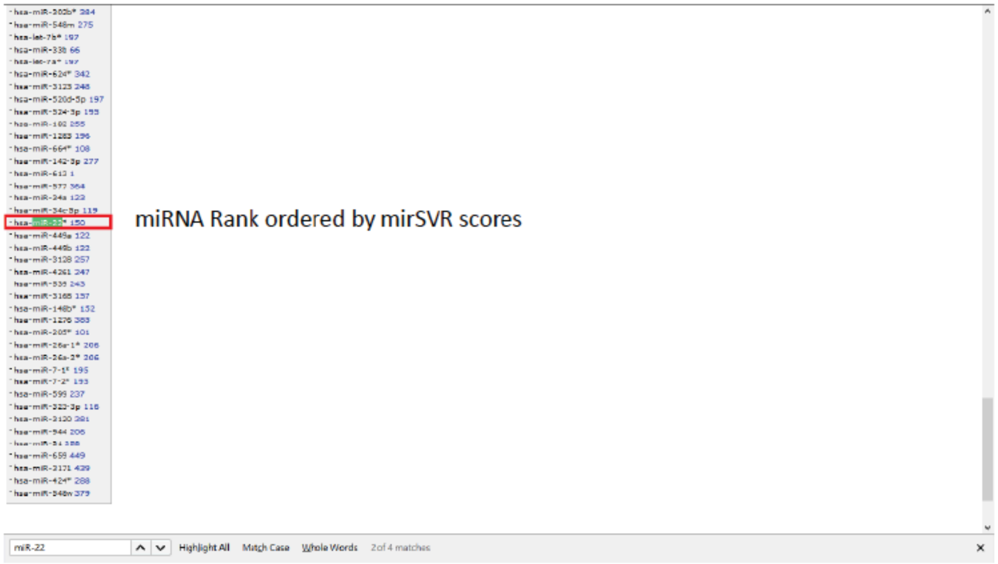

Figure S4C
Yeh and SIddique *et al.*

Extended Data Fig. 4C. 3′-UTR of human MYC mRNA is targeted by miR-22

D

Mouse *Myc* 3'UTR has two *miR-22* target site

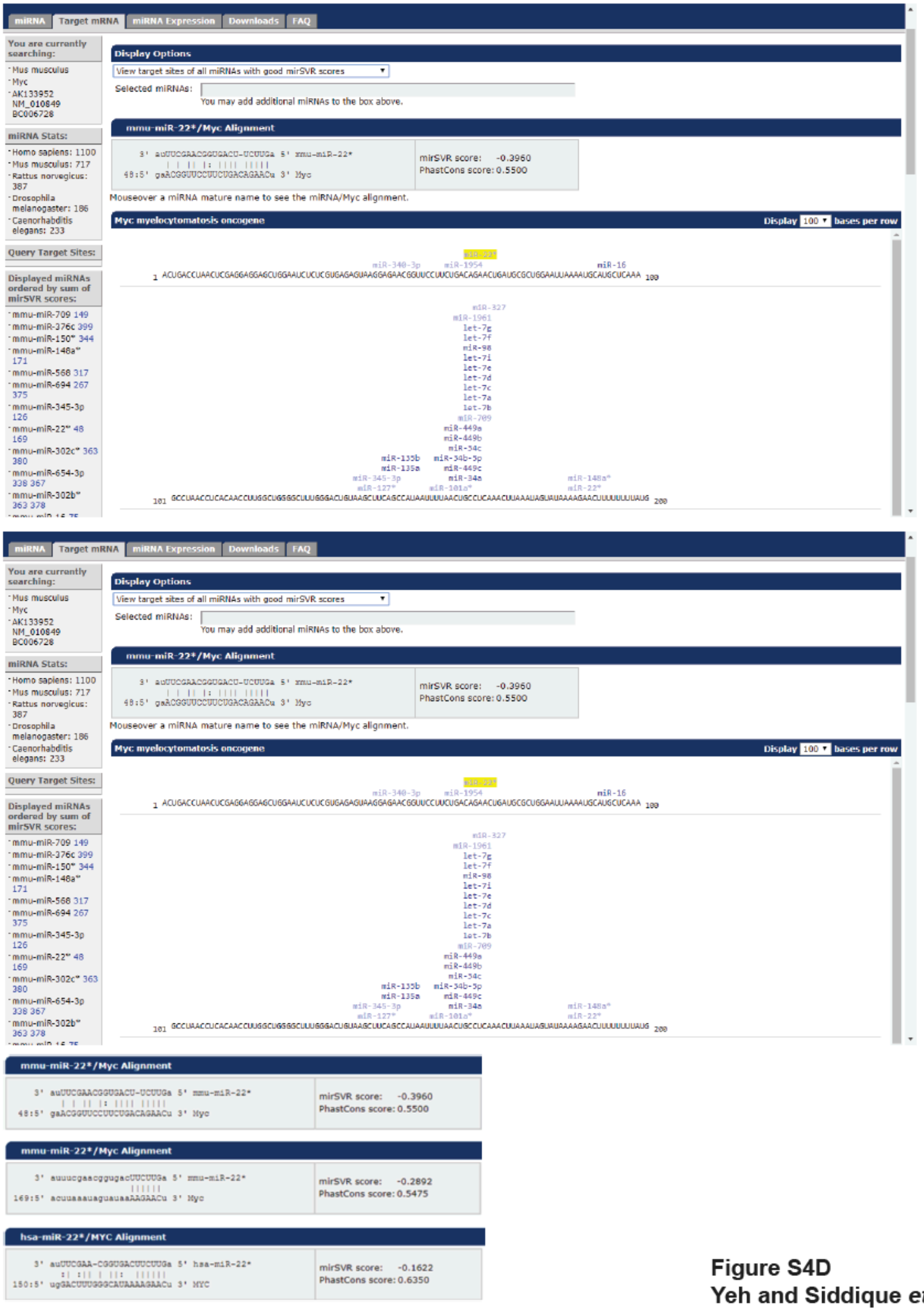

Figure S4D
Yeh and Siddique *et al.*

Extended Data Fig. 4D. 3′-UTR of mouse MYC mRNA is targeted by miR-22

3′-UTR of mouse VEGF mRNA targeted by miR-7014-3P

MicroRNA and Target Gene Description:

**miRNA Name**	mmu-miR-7014-3P	**miRNA Sequence**	CCUCUUUCAUACCAACCACAG
**Target Score**	81	**Seed Location**	678, 1123
**NCBI Gene ID**	22339	**GenBank Accession**	NM_001025250
**Gene Symbol**	Vegfa	**3' UTR Length**	1881
**Gene Description**	vascular endothelial growth factor A		

3' UTR Sequence

```
   1 gccaggctgc aggaaggagc ctccctcagg gtttcgggaa ccagacctct caccggaaag
  61 accgattaac catgtcacca ccacgccatc atcgtcaccg ttgacagaac agtccttaat
 121 ccagaaagcc tgacatgaag gaagaggaga ctcttcgagg agcactttgg gtccggaggg
 181 cgagactccg gcagacgcat tcccgggcag gtgaccaagc acggtccctc gtgggactgg
 241 attcgccatt ttcttatatc tgctgctaaa tcgccaagcc cggaagatta gggttgtttc
 301 tgggattcct gtagacacac ccacccacat acacacatat atatatatta tatatataaa
 361 taaatatata tgttttatat ataaaatata tatatattct ttttttaaa ttaactctgc
 421 taatgttatt ggtgtcttca ctggatatgt ttgactgctg tggacttgtg ttgggaggag
 481 gatgtcctca ctcggatgcc gacacgggag acaatgggat gaaaggcttc agtgtggtct
 541 gagagaggcc gaagtccttt tgcctgccgg ggagcaagca aggccagggc acgggggcac
 601 attggctcac ttccagaaac acgacaaacc cattcctggc cctgagtcaa gaggacagag
 661 agacagatga tgacagagaa agagataaag atgccggttc caaccagaag tttggggagc
 721 ctcaggacat ggcatgcttt gtggatcccc atgatagtct acaaaagcac cccgcccctc
 781 tgggcactgc ctggaagaat cgggagcctg gccagccttc agctcgctcc tccacttctg
 841 aggggcctag gaggcctccc acaggtgtcc cggcaagaga agacacggtg gtggaagaag
 901 aggcctggta atggcccctc ctcctgggac cccttcgtcc tctccttacc ccacctcctg
 961 ggtacagccc aggaggacct tgtgtgatca gaccattgaa accactaatt ctgtccccag
1021 gagacttggc tgtgtgtgtg agtggcttac ccttcctcat cttcccttcc caaggcacag
1081 agcaatgggg caggacccgc aagcccctca cggaggcaga gaaaagagaa agtgttttat
```

Figure S4E. **Yeh and Siddique *et al.***

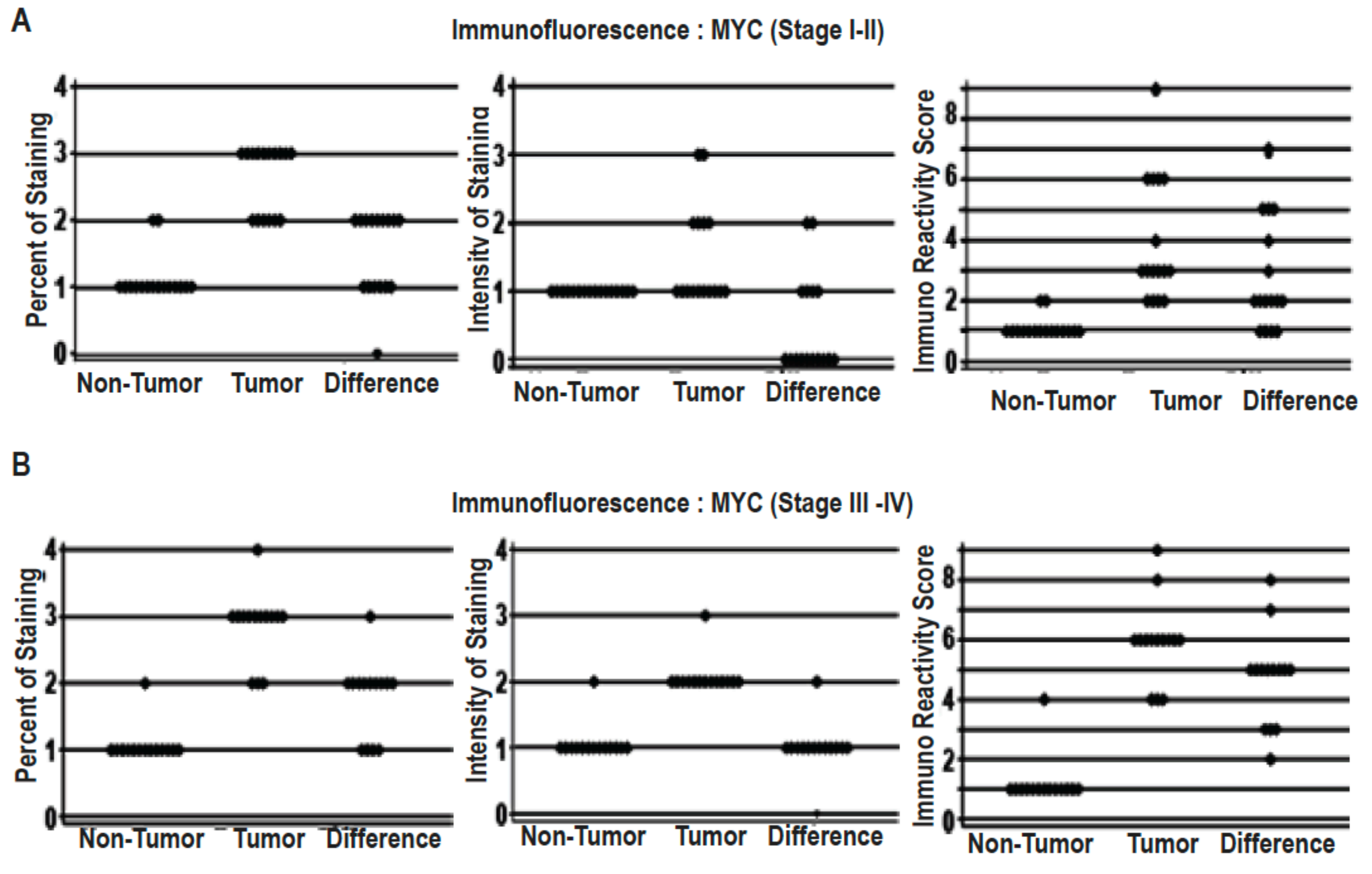

Figure S5A-S5B
Yeh and Siddique *et al.*

Extended Data Fig. 5A-5B. Immune Reactivity Score (IRS) of MYC in non-tumor and tumor regions of clinical HCC specimens. (**A-B**) Percent of staining, the intensity of staining, and Immune Reactivity Score (IRS: product of the two) for MSI-2 protein in Stages I-II (**A**) and Stages III-IV (**B**)**.**

C

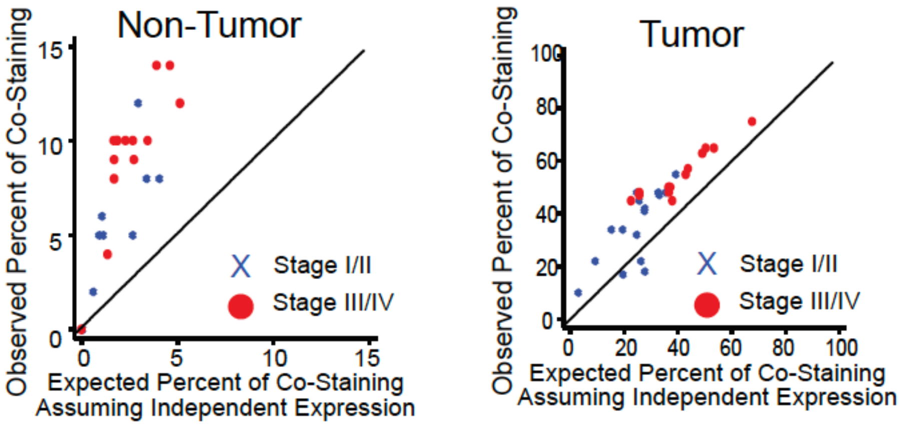

Figure S5C
Yeh and Siddique et al.

Extended Data Fig. 5C. Co-expression of MYC and MSI2 in human HCC specimens. Quantitation of MSI-MYC staining in (**Left**) Non-tumor areas and (**Right**) tumor area from HCC tissue specimens section staining. Observed percentage of co-staining vs. expected percentage of co-staining between MYC and MSI2, assuming independent expression marker expression in tumor samples. Scored at 20X magnification, N=30 fields, *p<0.05.

Extended Data Fig. 6. Uncropped films from each Figure.

Figure 2

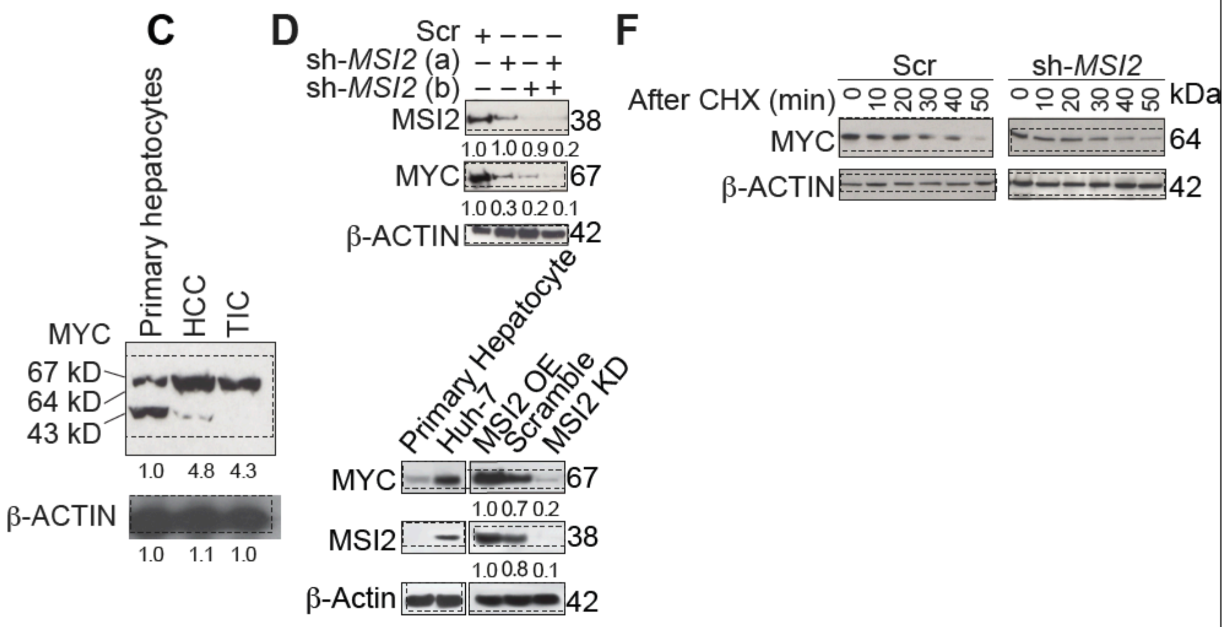

Figure 2g

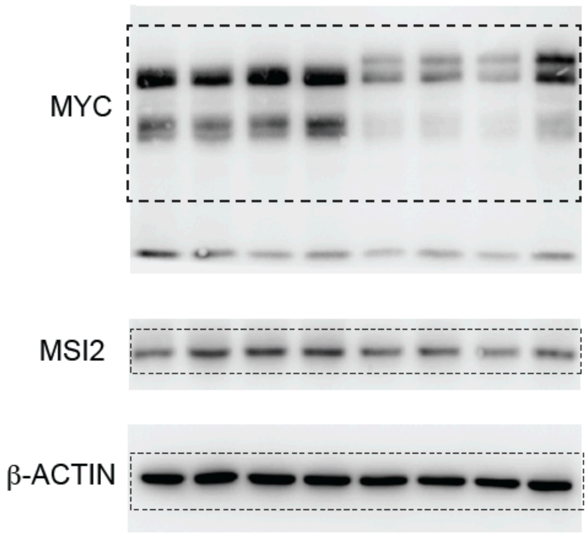

Figure 3L

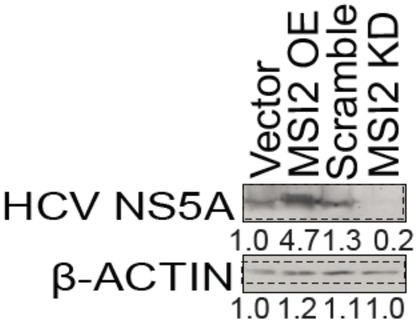
Vector
MSI2 OE
Scramble
MSI2 KD
HCV NS5A
1.0 4.7 1.3 0.2
β-ACTIN
1.0 1.2 1.1 1.0

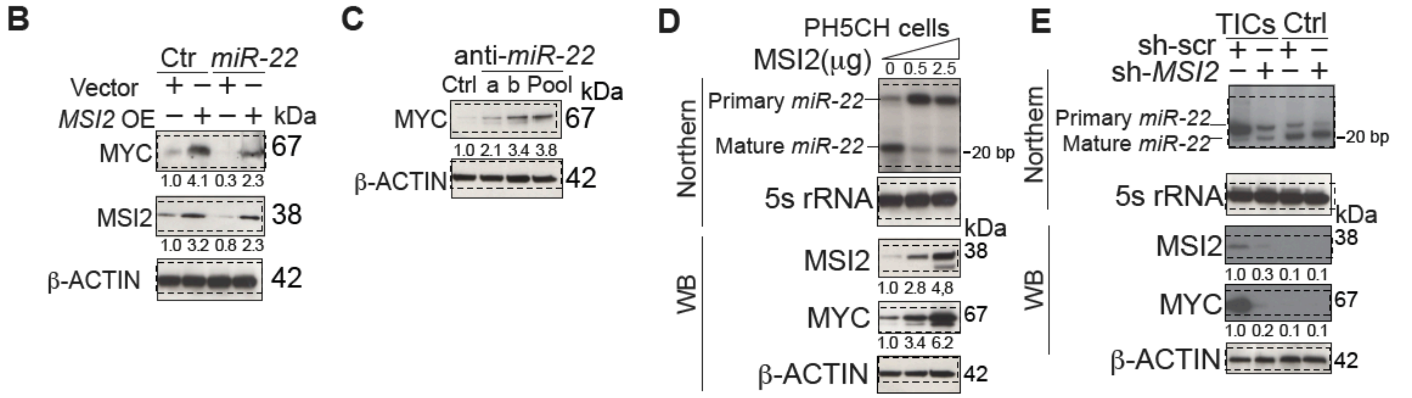
B
Ctr
miR-22
Vector
MSI2 OE
kDa
MYC
67
1.0 4.1 0.3 2.3
MSI2
38
1.0 3.2 0.8 2.3
β-ACTIN
42
C
anti-miR-22
Ctrl a b Pool
kDa
MYC
67
1.0 2.1 3.4 3.8
β-ACTIN
42
D
PH5CH cells
MSI2(μg)
0 0.5 2.5
Northern
Primary miR-22
Mature miR-22
20 bp
5s rRNA
kDa
WB
MSI2
38
1.0 2.8 4,8
MYC
67
1.0 3.4 6.2
β-ACTIN
42
E
TICs Ctrl
sh-scr
sh-MSI2
Northern
Primary miR-22
Mature miR-22
20 bp
5s rRNA
kDa
WB
MSI2
38
1.0 0.3 0.1 0.1
MYC
67
1.0 0.2 0.1 0.1
β-ACTIN
42

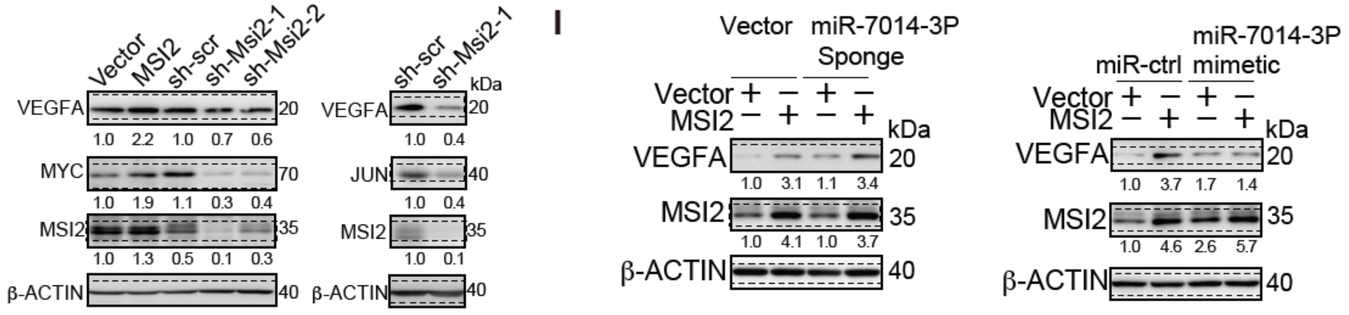
Vector
MSI2
sh-scr
sh-Msi2-1
sh-Msi2-2
VEGFA
20
1.0 2.2 1.0 0.7 0.6
MYC
70
1.0 1.9 1.1 0.3 0.4
MSI2
35
1.0 1.3 0.5 0.1 0.3
β-ACTIN
40
sh-scr
sh-Msi2-1
kDa
VEGFA
20
1.0 0.4
JUN
40
1.0 0.4
MSI2
35
1.0 0.1
β-ACTIN
40
I
Vector
miR-7014-3P Sponge
Vector
MSI2
kDa
VEGFA
20
1.0 3.1 1.1 3.4
MSI2
35
1.0 4.1 1.0 3.7
β-ACTIN
40
miR-ctrl
miR-7014-3P mimetic
Vector
MSI2
kDa
VEGFA
20
1.0 3.7 1.7 1.4
MSI2
35
1.0 4.6 2.6 5.7
β-ACTIN
40

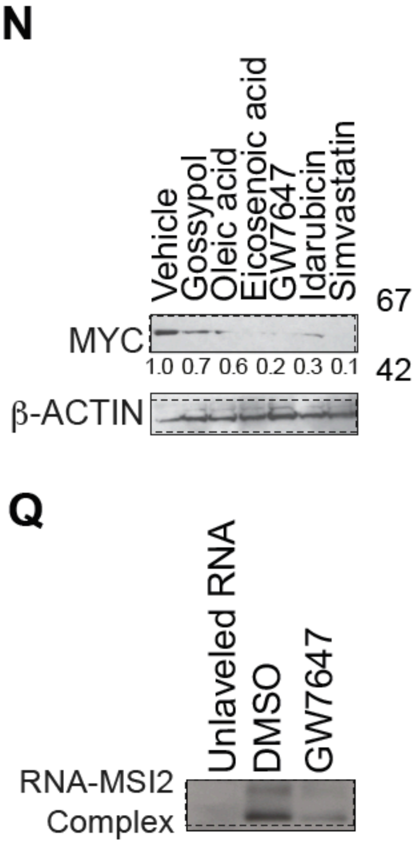

Figure 6
Yeh and Zhao et al.

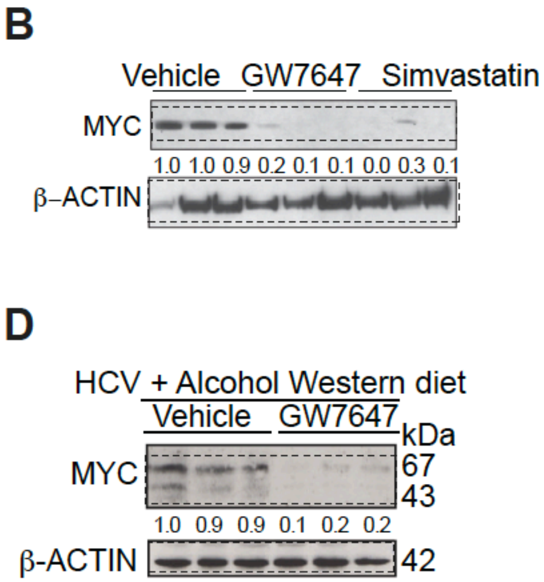

Figure 7
Yeh and Zhao et al.

- **EXPERIMENTAL MODEL AND SUBJECT DETAILS**

o In vivo animal studies

- **Genetically manipulated mouse models**
- **Mice**
- In the animal studies, NSG-SMG3 mice were bred at the USC mouse facility. The primary mouse fibroblast cultures were prepared from both core transgenic mouse and littermate embryos by trypsinizing the embryonic tissue and plating the dissociated cells. Littermates on FRG mice and NSG-SMG3 mice (Jackson Lab) were intercrossed at least six times. Lieber-DeCarli diet containing 3.5% ethanol or isocaloric dextrin (Bioserv, Frenchtown, NJ) was fed to all the mice in the alcohol feeding arm of the experiment. High-cholesterol high-fat diet was modified from TD.03350 (Harkan Teklad, Inc.) as previously described (Haluzik et al., 2004; Van Heek et al., 1997). Before treatment, mice were randomized to avoid the confounding effects.

Tumorigenicity studies in NSG mice

NOD;Shi-scid;*Il2rg* null (*NSG* and *NSG-SMG3*) mice were obtained from Jackson Laboratory and housed under pathogen-free conditions in accordance with approved Institutional Animal Care and Use Committee protocols. MSI2, sh-MSI2 and scrambled shRNA stable-transduced Huh7 cells were generated with the corresponding lentivirus construct. Transduced cells were resuspended in 100 μL PBS and 100 μl Matrigel (BD Biosciences) and injected subcutaneously into the dorsal hind flanks of anesthetized mice as previously described (Carlsson et al., 1983; Siddique et al., 2015; Siddique et al., 2011). Before treatment, mice were randomized to avoid the confounding effects. Tumor-bearing animals were euthanized at day 60 or at humane-end-point and tumors were collected and measured for volumes and weights. Tumor size was measured with calipers, and the volume was calculated according to the formula $V = (a \times b^2)/2$, where "V" represents tumor volume, 'a' represents the largest and 'b' the smallest superficial diameters (Carlsson et al., 1983).

Subcutaneous xenograft transplantation of the TICs into immunodeficient mice

All the animal experiments were approved by the IACUC Committee. MSI2, sh-MSI2 and scrambled stable-transduced Huh7 Cells (10,000) in 100 μl PBS were mixed with 100 μl Matrigel (BD Biosciences) and injected into the dorsal flanks of anesthetized *NOG* mice. The tumor volume was measured with a caliper and calculated according to the formula $V= (a \times b^2)/2$, where "V" represents tumor volume, "a" presents the largest, and "b" the smallest superficial diameter (Carlsson et al., 1983).

Tumor collection and analysis

Tumor-bearing animals were euthanized at day 35, and tumors were collected the volume and weight measured. The tumor tissues were divided for (1) fixation with neutrally buffered 10% formalin for H&E staining and histological evaluation of the tumor; (2) fixation with 4% paraformaldehyde followed by sucrose treatment for subsequent immune-staining; and (3) snap-freezing for mRNA and protein analysis of the targeted genes with shRNA.

- **METHOD DETAILS**

Human patients' tissue samples
The clinical and pathological characteristics of the patients were summarized in the **Table S8**. Thirty formalin-fixed, paraffin-embedded paired primary HCC tissues or non-tumor (adjacent liver) tissues were obtained. 30 patients were diagnosed as having HCC based on the clinicopathologic findings at the University of Southern California Norris Cancer Hospitals. At the time of surgical resection, the tumor area was dissected from the surrounding tissue. Part of the resected tissue was fixed in formalin and embedded in paraffin for histological diagnosis, another part of the resected material was snap frozen in liquid nitrogen for storage at –80°C for molecular analysis. All tissues were collected with patient informed consent that was granted before surgery by a protocol approved by the Institutional Review Board. These tissues were de-identified prior to use in this study. For immunostaining and immunoblotting analysis of MYC and MSI2 in human HCC, necropsy or surgically excised HCC tissues from 30 patients with or without HCV infection, with or without a history of alcoholism, with or without Obesity/Diabetes/ BMI >30 were obtained as cryo-preserved samples and paraffin embedded tissue sections according to the approved University Institutional Review Board (IRB) protocol. Many of the specimens were obtained from Liver Tissue Cell Distribution System at University of Minnesota. Samples were obtained from both genders between the ages of 42-80. Histologically, they all had varying degrees of steatosis (microvesicular and macrovesicular) and inflammation in addition to different stages of HCC. Completely normal liver tissues from 2 patients with accidental death or stroke, but without an apparent liver pathology, were also obtained for immunostaining or immunoblotting. Metastatic Brain tissue was provided by Dr. Zin Htway at HCA Los Robles Hospital and Medical Center. Allotransplants from 15 cryopreserved different mouse metastatic HCC cell lines were also studied for drug susceptibility.

Cells and cell lines
CD133+/CD49f+ murine hepatoma TICs were isolated using the anti-Prominin-1 (CD133) MACS affinity column (Miltenyi Biotech) following mechanical dissociation of liver tumors in sterile PBS and enzymatic digestion with 1 mg/ml collagenase/ dispase solution (Roche, IN) for 45 min at 37°C. The unbound column flow- through fraction (CD133-) was also recovered for propagation. Both fractions were maintained in the TIC culture medium (Dulbecco's modified Eagle's medium nutrient mixture F-12 (DMEM/F12) containing 10% fetal bovine serum (FBS), Nucleosides, 1 μM dexamethasone, epdimal growth factor (20 ng/ml), 1 % penicillin/ streptomycin and nonessential amino acid. CD133+/CD49f+ cells were sorted by FACS as previously described by Rountree et al (Rountree et al., 2009; Rountree et al., 2008). We quantitated the TIC population by the method of Extreme Limiting Dilution Analysis (ELDA; http://bioinf.wehi.edu.au/software/elda/). Huh7, human hepatoma cell line, PH5CH non-neoplastic human hepatocyte line, embryonic kidney cell line HEK293T were cultured in DMEM with 10% FBS and 1% Penicillin/Streptomycin at 37°C in 5% CO_2 humidified incubator.

Cell culture, reagents and antibodies
Human HCC cell lines Huh7, HepG2, Hep3B, PLC/PRF/5, and murine Hepa 1-6 HCC and HEK 293, were grown in DMEM supplemented with 10% FBS. Human H1299 NSCLC was grown in RPMI-1640 with 10% FBS. Human Calu-3 lung adenocarcinoma was grown in ATCC-formulated EMEM with 10% FBS. For enrichment of spheroid cells, HepG2 cells were seeded at a density of 5000 cells/ml in six-well ultra-low adhesion plates with serum-free DMEM/F12-K medium, N2 supplement (GIBCO; Carlsbad, CA, USA), 20 ng/ml human recombinant EGF, and bFGF (PeproTech; Rocky Hill, NJ, USA). True-Nuclear™ Transcription Factor Buffer Set was procured from BioLegend (San Diego, CA, USA); Anti-APP antibody was purchased from Sigma-Aldrich Co. (St. Louis, MO, USA); Anti-LAMB1 and anti-RNASE4a from Abcam (Cambridge, UK); Anti-MT2A from Novus Biologicals (Centennial, Colorado, USA); Anti-CMSS1 from Origene Technologies (Rockville, MD, USA); Anti-SQSTM1, anti-c-JUN and anti-NDRG1 from Cell Signaling Technology (Danvers, Massachusetts, USA); anti-VEGFA, anti-MSLN, anti-FN1, anti-NANOG (Alexa Fluor 647 conjugate), anti-MYC (Alexa Fluor 488 conjugate) and mouse and rabbit IgG isotype control antibody from Santa Cruz Biotechnology (Dallas, TX, USA). All the oligonucleotides for primer sets, gBlock gene fragments and the RNA mimics for control and miR-7014-3p were synthesized by IDTDNA (Coralville, IA, USA). NEBuilder HiFi DNA Assembly kit was procured from New England Biolabs (Ipswich, MA, USA). Antibodies against human MSI2, IL1RL1 and CTH were purchased from Proteintech (Chicago, IL, USA); human recombinant epidermal growth factor (EGF), and basic fibroblast growth factor (bFGF) from Peprotech (Rocky Hill, NJ, USA); and reagents for luciferase assay, from Promega (Medison, WI, USA).

Plasmids, lentivirus and retrovirus vectors, and production of lenti- & retro- viruses
All retroviruses were based on lentivirus (pPAX2: Addgene) or MMTV vectors (pVPack-GP: Stratagene). Lentivirus vectors were prepared by standard procedures in HEK293T cells. Three plasmids, packaging vector

pPAX2 (Addgene), ecotropic envelope gene (VSV glycoprotein) expression vector pMD2.G (Addgene), and shRNA expression cassette, were transfected in HEK293T cells using BioT transfection reagent (Bioland Scientific LLC). Retroviruses expressing were produced in Phoenix cells/ HEK293T (Saitoh, Akashi et al. 2004). After 48 hr transfection, the virus supernatants were harvested, purified, and mixed with polybrene (8 µg/ml), for infection of Huh7 cells / TICs. The virus supernatant was tested for their titer using Lenti-X™ GoStix (Takara). To increase silencing effects and to reduce off-target effects, a combination of shRNA lentiviruses was used to knock down target genes.

Construction of dual luciferase reporter plasmids

Dual reporter plasmids which contain firefly luciferase gene under control of the IRES from 5'UTR of either JUN or VEGFA mRNA were constructed to assess impact of MSI2 on IRES-driven luciferase activities. Briefly, for JUN IRES constructs, a 1020 bp and a 491 bp 5'UTR fragments spanning from −977 to +43 and -448 to +43 relative to the start codon of JUN mRNA were PCR amplified from HepG2 first-strand cDNA library. The corresponding PCR fragments were digested with SpeI and EcoRI and ligated into the pRL and phpRL linearized by SpeI and EcoRI digestion. For VEGFA IRES constructs, a 496 bp 5'UTR fragments were PCR amplified, digested with SpeI and EcoRI and ligated into SpeI /EcoRI sites of the linearized pRL and phpRL. The sequences of primer sets employed for generating dual luciferase reporter constructs were listed in Supplementary Tables S6.

Construction of Vegfa 3' UTR reporter, miR-7014-3P-sponge, and production of lentivirus for lentiviral transduction

To generate Vegfa 3' UTR reporter constructs, the gBlocks gene fragments for 3' UTR of Vegfa mRNA with wildtype or mutant miR-7014-3P binding sites were synthesized by IDTDNA and assembled into pMIR-report vector linearized with MluI and HindIII using NEBuilder HiFi DNA Assembly kit according to manufacturer's instruction. The sponge construct for mmu-miR-7014-3P containing six tandem repeats of the bulge-containing miR-7014-binding motif was generated by synthesizing a set of sense and antisense oligonucleotides with 5′-CTAG-3' overhang and 5′-GATC-3' overhang respectively. The annealed DNA fragments were ligated into XbaI/ BamHI sites of linearized pCDH plasmid. Lentivirus was produced by harvesting culture supernatants obtained upon co-transfection of HEK293T cells with the generated lentiviral constructs and pPAX2 and pMD2.G packaging plasmids. Hepa1-6 cells were plated in a 12-well plate and spin-infected by centrifugation at 1100 × g for 30 min in the presence of lentiviral supernatants and 8 µg/ml polybrene (Sigma-Aldrich Co.). Stably transduced cells were obtained under 2 µg/ml puromycin selection for 48 hours. The sequences of oligonucleotides employed for generating Vegfa 3' UTR reporter, miR-7014-3P sponge, and mimics were listed in supplementary Extended Data Fig. 4E and Tables S6.

Isolation of mouse TICs using MACS

Tumor-initiating Cells (TICs) were isolated from liver tumor acquired from our novel animal model, HCV- NS5A Tg mice with high cholesterol, high fat diet (HCFD)/Alcohol long-term (12 months) feeding. Liver tumors were mechanically dissociated, digested in dispase (Sigma) containing DNase I (Sigma) and incubated at 37°C for 2 hours. Single cell suspensions were incubated with CD133 microbeads (Miltenyi) and separated using an auto-MACS device (Miltenyi), according to manufacturer's instructions. TICs were maintained in Dulbecco's modified Eagle's medium nutrient mixture F-12 (DMEM/F12) containing 10% fetal bovine serum (FBS), Nucleosides, 1 µM dexamethasone, epidermal growth factor (20 ng/ml), 1 % penicillin/ streptomycin and nonessential amino acid.

RNA-Immunoprecipitation (RIP)

$2X10^7$ TICs overexpressing Flag-MSI2 and stably transduced shRNA against MSI2 were subjected to RNA immunoprecipitation (RIP) using the MBL RIP kit (MBL International, MA). In brief, cells were washed with cold PBS and lysed with provided RIP lysis buffer. Anti-Flag M2 Ab (Sigma-Aldrich), anti-rabbit Ab, or anti-MSI2 Ab (EMD Millipore), pre-incubated with magnetic beads (5µg), were used to immunoprecipitate Flag–MSI2–RNA complexes. Immunoprecipitated complexes were washed and treated with proteinase K. Finally, RNA was extracted with the reagents provided in the kit.

Bioinformatics analysis of RNA sequencing

Sequencing reads were trimmed of adaptors, and high-quality reads were used for further analysis using Burrows-Wheeler Aligner (BWA) and TopHat essentially following the procedure detailed by Huang et al. (Huang et al., 2013) except that we used a windowed approach to call the enriched regions in the genome. Further reads aligning to mitochondrial DNA, repetitive elements or unassigned sequences were discarded. This resulted in uniquely

aligned 13 million reads for further analysis. Briefly in the windowed approach signal density was calculated in sliding 50bp windows, and aligned reads were considered to be within a window of the midpoint of its estimated fragment. Midpoints in each window were counted, and empirical distributions of window counts were created. Genomic bins containing statistically significant regions were identified based on background distribution of randomized reads specific for each sample as detailed previously (Kim et al., 2013). Further, a Mann and Whitney U-test were used to determining the differential enrichment Msi2 IP from input sample.
Similarly, for RNA-seq high-quality reads were aligned to mm9 using Top-Hat in conjunction with a gene model from Ensemble release 61. Data were quantitated by counting the number of RPKM (reads per kilo base per million mapped reads) (Mortazavi et al., 2008). The read counts were adjusted globally by matching count distributions at the 75th percentile and then adjusting counts to a uniform distribution between samples. Differential expression was estimated by selecting transcripts that displayed significant changes (FDR, $P < 0.05$) using a null model constructed from 1% of transcripts showing the average closet level of observation to estimate experimental noise as explained previously (Mortazavi et al., 2008). Significantly enriched regions of RIP-seq were annotated with closest non-overlapping gene/ ncRNA (downloaded from UCSC genome browser on Jan.5, 2016). For visualization, alignment files were transformed into reading coverage files (25 bp bin and smooth length 50 bp) to generate bigwig or tdf files. IGV was used at different steps to visualize the data. For identification of motif center of RIP-seq window were considered as peak summit the sequence ± 25 bp across this submit was used to identify the consensus motif in different data sets. The link for data to be included: https://drive.google.com/folderview?id=0B3skVboh2QdZ1REUGtwaG11SG8&usp=sharing

Bioinformatics analysis of binding motif from MSI2 RIP-seq

The reads were aligned to mm9 genome using BWA (https://www.ncbi.nlm.nih.gov/pubmed/19451168) allowing single mismatch. The genome was divided into non-overlapping bins of 20 bp and a sliding window approach was used to find regions of enrichment. A read is considered to be within a window if the midpoint of its estimated fragment is within the window. The center of the peak will have maximal enrichment. Zero-truncated negative binomial model (ZNTB) was used to determine the statistically significantly enriched peaks / regions and a peak cut off of FDR adjusted p value ($p<0.05$) was used (represented as P value in Table). Further a Mann and Whitney U test was used determine the differential enrichment Msi2 IP from input sample. Further ranking of peaks/ genes were performed using Fold enrichment (>2 fold).

Reverse transcription and quantitative PCR (RT-qPCR)

Total RNA was extracted by using TRIzol Reagent (Invitrogen) and purified using the RNeasy mini kit (QIAGEN) according to the manufacturer's protocol. RNA concentration and purity were determined by A260 and A260/A280 ratios, respectively. The RNA samples were treated with DNase I (Invitrogen) to remove residual traces of DNA. cDNA was obtained from 1 μg of total RNA, using SuperScript III reverse transcriptase (Invitrogen) and oligo dT primers in a final volume of 20 μl. Quantitative real-time PCR was performed on an ABI 7900 HT Real-Time PCR machine using 2X SYBR Green Master Mix (Applied Biosystems). Specificity of PCR products was tested by dissociation curves. Gene expression was determined using the 2−ΔΔCt method described in the ABI user manual.

SDS-PAGE and immunoblot analysis

Huh7 Cells were lysed with lysis buffer (100 mM NaCl, 50 mM Tris-Cl pH 7.5, 0.5 mM EDTA, 1% NP-40) containing complete protease inhibitor cocktail (Roche Life Science, Indianapolis, IN, USA). Cell lysates were separated by SDS-PAGE and transferred onto PVDF membranes. The membranes were blocked for 30 min with 5% fat-free milk in Tris-buffered saline containing Tween-20 (TBST; 50 mM Tris-Cl pH 7.5, 150 mM NaCl, 0.1% Tween-20) and incubated for 2 h with the indicated antibody in TBST containing 2% BSA. The membranes were subsequently washed in TBST and incubated for 1 h with HRP- conjugated secondary antibody. The immunoreactive bands were visualized using chemiluminescent HRP substrate (Immobilon Western; Millipore, Temecula, CA, USA) and the ChemiDoc MP Imaging System (BIO- RAD; Hercules, CA, USA). The density of bands on images of western blots was analyzed by ImageJ.

Site-directed mutagenesis

This method was performed according to the manufacturer's protocol (Quick Change Lightning site-directed mutagenesis kit, Agilent Technologies, USA). The sequences containing putative IRES sites (5'-aattccagcgagag-3') of pRMF plasmids were mutated by using following mutagenic primers. The primers used in the experiments are Fwd- 5'-ctccctctgcctgccgctggatctaggggatcctgactagtgattc-3' and Rev- 5'-gaatcactagtcaggatcccctagatccagcggcaggcagagggag-3'. The plasmids containing mouse 3'UTR of MYC region (pRL-MYC 3'UTR) were obtained from Addgene. The sequences containing putative miR-22 binding sites (3'-auUucgaacggugacuucuug a-5') were mutated by using following mutagenic primers. The primers used in the experiments are: for Target 1-Fwd-5'-

cagccataattttaactgcctcaaacttaaatagtgtaaggggactttttttatgcttcccatctttttctttttccttttaac-3' and Rev-5'-gttaaaaggaaaaagaaaaaagatgggaagcataaaaaaaagtccccta cactatttaagtttgaggcagttaaaatta tggctg-3'. Target 2-Fwd-5'-agagtaaggagaacggggccttttgacatgactgatgcgctggaattaaaatgcatgctca-3’ and Rev-5'-tgagcatgcattttaattccagcgcatcagtcatgtca aaaggccccgttctccttactct-3'; the presence of the mutations was confirmed by direct sequencing.

Luciferase reporter assays

Huh7 cells were plated on 24-well plates, allowed to adhere overnight. The following day, cells were co-transfected with MYC IRES wildtype or mutant luciferase reporter construct, and expression plasmids for MSI2, as indicated, using BioT (Bioland Scientific, LLC.). Cells were lysed 48 hr post- transfection and luciferase activity was determined. Cell lysates were subjected to Western blotting analysis for detection of relative protein expression level of MSI2. Relative luciferase activities were calculated as fold induction relative to unstimulated control.

UV cross-linking and EMSA

To show an interaction between MSI2 protein and MYC mRNA, UV cross-linking and EMSA was performed with 32P-labeled MYC entire IRES or the MSI2-binding-motif mutant transcripts combined with purified MSI2-His6, in the presence or absence of cold probes as previously described (Paulin et al., 1998). Radiolabeled RNA transcript was generated from pSK-ML (MYC IRES expression construct) and pSK-GAP-L (GAPDH vector for specificity control) (plasmid gifts from Prof. Anne Willes in the UK) (Paulin et al., 1998) by using MAXIscript™ SP6/T7 Transcription Kit (Invitrogen). Radiolabeled RNA transcript was incubated with purified recombinant MSI2 and subjected to EMSA.

Sucrose density gradient centrifugation and RT-qPCR analysis

Sucrose density gradient (5%–50%) ultracentrifugation was used to separate ribosomes into polysomal and subpolysomal fractions. Briefly, Huh7 cells were treated with 100 ug/ml cycloheximide (CHX) for 15 min at 37°C then collected and lysed in polysome lysis buffer. Following cell disruption and centrifugation, cytoplasmic cell lysates (supernatant) were layered onto sucrose gradients 5–50% and spun at 45,000 rpm for 1.5 h at 4 °C in a SW55Ti rotor (Beckman Instruments). Gradients were then fractionated and total RNA was purified from each fraction using TRIzol (Invitrogen), according to the manufacturer’s instructions. Reverse transcription was performed using SuperScript III first-strand synthesis system (Invitrogen) with oligo-dT primer. RT-qPCR was carried out using ABI Step One Plus Real-Time PCR System and TaqMan universal PCR Master Mix (Applied Biosystems) for gene expression analysis. Data were analyzed using the $2^{-\Delta\Delta Ct}$ method described in the ABI user manual. The expression level of MYC mRNA was normalized to GAPDH. The primer sequences are listed below, MYC Fwd: 5'-ttcgggtagtggaaaaccag-3’, Rev: 5'- cagcagctcgaatttcttcc-3’; GAPDH Fwd: 5'-gagtcaacggatttggtcgt-3’, Rev: 5'-gatctcgctcctggaagatg-3’.

MicroRNA isolation, cDNA synthesis, and RT-qPCR

Small RNAs including microRNAs were extracted by using microRNA Isolation Kit (Sigma) followed by poly-A tailing reactions and miRNA Easy Script cDNA synthesis kit (Abm Good, BC, Canada). 250 ng small cellular RNA was used for cDNA synthesis. The specific microRNA was amplified using primers obtained from Abm Good (cat# MPH01277), The RT-qPCR was performed using Evagreen miRNA qPCR Master Mix (Genomics-online) for quantitative real-time analysis of miRNA samples. The results were normalized to *U6 snRNA* (MPH00001). The pre-miR22 was amplified by using the primers Fwd- 5’-ctgagccgcagtagttcttc-3’; Rev- 5’ cagagggcaacagttcttca-3’

Northern blots for miR22hg

Levels of *miR-22* in cells under different expression levels of MSI2 were measured by using Northern Blot Assay kit, according to manufacturer's instructions (Signosis). In brief, 10 µg of small RNAs were separated by electrophoresis in 16% Tris-borate-EDTA urea gels, transferred to a nylon membrane, and then immobilized by UV-crosslinking. Membranes were hybridized overnight at 42°C with a biotin-labeled, complementary *miR-22* and U6 probes. Membranes were blocked for 30 min and then incubated with streptavidin-HRP at room temperature. After washing, miRNAs were detected with a chemiluminescence substrate and the bands were detected by exposure to X-Ray film. The densities of the bands were measured by ImageJ software.

Spheroid Formation

Sorted Cells were cultured in 6 well ultra-low attachment cell culture plates (Corning) in DMEM/F12 media (Sigma) containing 10% FBS, Insulin (1 mg/ml), dexamethasone (1 µM), nicotinamide (10 mM), Hepes (5 mM), 1% penicillin/ streptomycin, and Epidermal Growth Factor (20 ng/ml). Spheroids were incubated for twelve days at

37°C in 5% CO2 incubator. Statistical significance was determined using ANOVA single factor. Asterisks (*) indicate a p-value < 0.05.

Serial Spheroid Formation
Huh7 cells were washed with 1x PBS and centrifuged at 150 g for 5 min. Cells were counted using a cell counter. 100 cells were serially diluted to three separate wells in a 6 well Ultra-low attachment cell culture plate by Corning. DMEM F12 media (Sigma) was used containing 10% FBS, Insulin (1 mg/ml), Dexamethasone (1 x 107 M), Nicotinamide (10 mM), HEPES (5 mM), 1% penicillin/ streptomycin, and Epidermal Growth Factor (20 ng/ml). Spheroids were allowed to grow for ten days in the 5% CO2 incubator and then were serially diluted to 100 cells per well twice more. Statistical significance was determined using ANOVA Single Factor. Asterisks (*) indicate a p-value < 0.05.

In silico 3D structure prediction
To simulate the docking site(s) between MSI2 and mRNA regions, local quality was estimated. Human MSI2 structure was simulated by using templates of 2mssA and 2lyvA. Non- redundant sets of PDB structures were compared. The simulated structure has three potential RNA binding sites. To predict the interfaces between RNA and MSI2, each binding site was simulated 1500 times with redundant radii searching for lowest energy, results were then combined to return the lowest energy from these parameters. Cavity 2 had the lowest calculated energy and was chosen for further analysis.

Fluorescence Polarization assay
FITC-tagged MYC IRES-MSI2 binding RNA (wild-type) or FITC-mutant IRES RNA was serial diluted in assay buffer (150 mM NaCl, 25 mM HEPES (pH 7.6), 0.05% CHAPS and 1 mg/ml BSA) in a range of 0.5–500 nM and aliquoted into a black 384-well non-binding microplate (Greiner Bio-One, Germany) in triplicates (20 ul). Buffer-only (20 ul) serves as blank controls and probe-only controls for assessing displacement by the unlabeled small molecules. Total fluorescence intensities of probe were recorded with an EnVision multilabels plate reader 2103 (PerkinElmer) using the following settings: mirror – FITC FP, excitation filter –FITC FP 480 nm, emission filter 1 – FITC FP p-pol 535 nm, emission filter 2 – FITC FP s-pol 535 nm. The resulting values were plotted with GraphPad Prism against the respective concentrations of probe.

Histology & Immunohistochemistry
Five micrometer-sections were stained with hematoxylin and eosin (H&E) or processed for other staining. Tissue samples were fixed on 10% neutral buffered formalin, embedded in paraffin, sectioned, and stained with hematoxylin and eosin or immunohistochemistry staining was performed using primary antibodies against MSI2 and/ or MYC based on the standard protocol with their respective secondary antibodies. Slides were then mounted using xylene based mounting media including hematoxylin for nuclei counterstaining (Vector Laboratories) according to the manufacturer's recommendations. The staining was subjected to morphometric analysis. To determine the specificity of immunohistochemistry staining, serial sections were similarly processed, except primary antibodies were omitted in controls. The areas of interest were quantified using MetaMorph software.

Immunofluorescence Staining
The staining intensity, percentage of stained cells and immuno-reactive score was determined as reported earlier (Koomagi and Volm, 1999). In brief, the intensity of staining was scored on a scale of 0 to 3, in which, negative staining = 0, weakly positive staining = 1, moderately positive staining = 2, and strongly positive staining =3. The extent of distribution of positive cells was estimated on a scale of 0 to 4, in which negative = 0, positive staining in 1-25.0% of cells = 1, positive staining in 26-50.0% =2; positive staining in 51.0-75.0% =3; and positive staining in more than 75.0% =4. The results were evaluated as immunoreactivity score (IRS), where the IRS = (percentage of positive cells) X (staining intensity). Two investigators separately and independently evaluated the immunohistochemical staining without knowledge of the clinical data. Significant differences in interpretation were resolved in conference. In cases without significant discordance, scores of two observers were averaged.Normal and Tumor tissues were provided by the University of Minnesota. Microscopy services were provided by the Cell and Tissue Imaging Core of the USC Research Center for Liver Diseases, HIH grants No. P30 DK048522 and S10 RR022508.

RT-qPCR analysis of mature microRNA expression
Total RNA including small RNA species was purified using TRIzol (Invitrogen) following the method provided in the TRIzol user manual. Reverse transcription and quantitative PCR (RT-qPCR) was carried out using miR-X miRNA qRT-PCR TB Green kit (TAKARA BIO) according to the manufacturer's instructions, and ABI

StepOnePlus™ Real-Time PCR System for microRNA detection. Data was analyzed using the $2^{-\Delta\Delta Ct}$ method. The expression of microRNA was normalized to RNU6. The primer sequences are listed in Supplementary Supplementary Table 6.

Flow cytometric analysis
For intracellular staining, HepG2 spheroid cells were first dissociated into single cells by StemPro Accutase (GIBCO, Carlsbad, CA, USA). The cells were subsequently fixed and permeabilized with Fix and Perm Buffers following instruction described in True-Nuclear™ Transcription Factor Buffer Set (Biolegend, San Diego, CA, USA), and were incubated in 1 × Perm Buffer containing MSI2 Ab, Alexa Fluor 647-conjugated NANOG, and Alexa Fluor 488-conjugated MYC Abs at 4 °C overnight. The next day, the cells were washed and incubated with Alexa Fluor 350-conjugated anti-rabbit Ab at 4 °C for 1 hour. The cells were washed and resuspended in PBS containing 0.2% BSA, and were subjected to flow cytometric analysis on a FACSCanto II flow cytometer with FACSDiva software (Becton Dickinson, San Jose, CA, USA).

Statistical Analysis
Statistical significance was estimated by unpaired, two-tailed Student's *t*-test. Bars represent the mean± S.D. *, $P < 0.05$. Paired *t*-tests were used to compare the marker expression levels between tumor vs. non-tumor tissues. For each of the markers, percent of staining and intensity of staining, as well as the product of the two measurements (immunoreactivity score: IRS), were presented as dot plots (Koomagi and Volm, 1999).
The differences in survival among the three groups of mice (scrambled, MSI2 overexpressing and sh- MSI2) were compared using log-rank tests. Given the small numbers of mice in this experiment, permutation tests were conducted to evaluate the significance of the observed differences among the three groups of mice. Statistical analyses were performed using STATA software (version 11.0; Stata Corp LP College Station, TX). Detailed analyses are explained in Supporting Materials.

Supplementary Discussion
MYC is a potent oncogene of liver tumors and inactivation of MYC is sufficient to induce sustained regression of MYC-initiated liver tumors in mice (Ryan and Birnie, 1996). MYC regulates several cellular processes and is crucial for stem cell maintenance. Down-regulation of MYC both *in vitro* and *in vivo* induces growth inhibition, and differentiation of HCC (Ryan and Birnie, 1996). Elevated expression of MYC occurs through multiple mechanisms in tumor cells. One such mechanism is IRES-mediated trans-activation. There are several IRES-trans acting factors (ITAFs) that bind to the MYC IRES such as- PSF (PTB-associated splicing factor) and YB-1 (Y-box binding protein 1) (Cobbold et al., 2008; Paulin et al., 1998). The 5'-UTR of the MYC is highly structured as it plays an important role in modulating levels of MYC protein. Transcription initiation of MYC starts from multiple promoters – P0, P1, P2, and P3 but the majority of mRNAs initiate from the P2 promoter (Zakaria et al., 2014). Two major MYC proteins MYC-1 (67kDa) and MYC-2 (64 kDa) are translated from the P2 promoter transcript. The *MYC* IRES is 341 nucleotide long (nt 2500-2840 exon 1) and is located downstream of the P2 promoter. In the present study, we observed that MSI2 selectively modulated the expression of the longer form of MYC, thus initiating translation from within exon1 and allowed IRES-dependent translation.
MSI2 promotes EMT in liver cancer through LIN28 expression (Fang et al., 2017). Other ways to target MSI2 includes antisense oligonucleotides (ASOs) (Fox et al., 2016) or small molecule inhibitor for MSI2 (Ro 08–2750) targets MYC (Minuesa et al., 2019). It was reported that MSI2 with HuR inhibits the processing of miR-7 (Choudhury et al., 2013). Another study showed that LIN28 blocks processing of *pre-let-7g* to mature *let-7g* (Viswanathan et al., 2008). Although we do not know if MSI2 interacts physically with the miRNA processing machinery or exerts its influence via other factors, this study represents yet another component of the complex regulation of *MYC*. In this context, MSI2 warrants further examination, especially if it functions similarly to LIN28, either directly or indirectly on MYC. Finally, the *MYC*-3'-UTR also interacts with different miRNAs expressed in other cancers; thus it will be of interest to learn whether MSI2 regulates *MYC* translation and similarly regulates other miRNAs. Although the functions of many identified long noncoding RNAs remain unknown, we demonstrated that *miR-22* availability is antagonized by MSI2. Our work identified the MSI2-MIR22HG-miR-22-MYC axis as an important regulator of HCC.

- **QUANTIFICATION AND STATISTICAL ANALYSIS**

Statistical Considerations: For this study, HCC from three etiological backgrounds was used for PDX models: HCC can be attributed to ALD and NASH as these metabolic liver diseases are increasingly contributing to HCC incidence and are primary diseases of the center's interest. HCC from HCV or HBV patients are not included in this study because different viral genotypes may exhibit confounding different phenotypes and HCV- or HBV-infected PDX models pose a significant logistical challenges from the biosafety standpoint. To account for patient

variability, we collected HCC from three patients per etiology. We expanded one patient HCC into at least 48 NSG mice by 2nd or 3rd level passages, collectively generating 144 or more mice from three patients per etiology. These 144 mice were randomly assigned to the two groups. The initial analysis considered the two etiology groups separately and the two treatment groups were compared by two-tailed, Student's t-test or Mann-Whitney U-test if abnormal data distribution exists.
For in vitro culture studies, Student's t-test was used to analyze data using Statistical software. **Statistical Analysis:** Experimental data are presented as the mean ±standard deviation (SD). All statistical analysis was performed using a two-tailed Student's t test and Chi squared test. Differences were considered statistically significant when P values were less than 0.05. Error bars reflect standard errors.

- **DATA AND CODE AVAILABILITY**
  - o **NCBI tracking system number:**
    - § The data used in this study has been deposited to NCBI under **GSE61435 (Microarray)** and **GSE68237 (ChIP-Seq)**.

Supplementary References

Carlsson, G., Gullberg, B., and Hafstrom, L. (1983). Estimation of liver tumor volume using different formulas - an experimental study in rats. J Cancer Res Clin Oncol *105*, 20-23.
Choudhury, N. R., de Lima Alves, F., de Andres-Aguayo, L., Graf, T., Caceres, J. F., Rappsilber, J., and Michlewski, G. (2013). Tissue-specific control of brain-enriched miR-7 biogenesis. Genes Dev *27*, 24-38.
Cobbold, L. C., Spriggs, K. A., Haines, S. J., Dobbyn, H. C., Hayes, C., de Moor, C. H., Lilley, K. S., Bushell, M., and Willis, A. E. (2008). Identification of internal ribosome entry segment (IRES)-trans-acting factors for the Myc family of IRESs. Mol Cell Biol *28*, 40-49.
Fang, T., Lv, H., Wu, F., Wang, C., Li, T., Lv, G., Tang, L., Guo, L., Tang, S., Cao, D., *et al.* (2017). Musashi 2 contributes to the
stemness and chemoresistance of liver cancer stem cells via LIN28A activation. Cancer Lett *384*, 50-59.
Fox, R. G., Lytle, N. K., Jaquish, D. V., Park, F. D., Ito, T., Bajaj, J., Koechlein, C. S., Zimdahl, B., Yano, M., Kopp, J., *et al.*
(2016). Image-based detection and targeting of therapy resistance in pancreatic adenocarcinoma. Nature *534*, 407-411.
