# Supplementary material for: MSI2 promotes translation of multiple IRES-containing oncogenes and virus to induce self-renewal of tumor initiating stem-like cells
Source: Cell Death Discov. 2023 Apr 28;9:141. doi: 10.1038/s41420-023-01427-9 (PMC10147607; doi:10.1038/s41420-023-01427-9)
Supplement: Supplementary file 7 — Supplementary Table 2. [file 41420_2023_1427_MOESM7_ESM.pdf]

Table S2. Peaks used for identification of motif with peak summits

Chromosom	Peak_Start	Peak_Stop	Peak summit	Motif start	motif end	FDR p-value	Scaled fold c	strand	Gene Symbol	Gene Sectic	Distance to TSS
17	8145113	8145394	8145254	8145204	8145279	1.72E-17	104.871	+	Rsph3a	Intron 2	6634
19	22516276	22516466	22516372	22516322	22516397	0.000394	101.961	+	Trpm3	Intron 2	302669
2	118649434	118649534	118649485	118649435	118649510	0.000105	101.379	+	Knstrn	Intron 12	9695
17	7146224	7146503	7146364	7146314	7146389	0	98.331	-	Rsph3b	Intron 14	6203
4	45359372	45359573	45359473	45359423	45359498	0	95.677	+	Dcaf10	Intron 2	4399
18	42412070	42412367	42412219	42412169	42412244	3.76E-05	94.9604	-	Lars	Intron 60	9353
X	70271970	70272118	70272045	70271995	70272070	0	93.0056	+	Zfp185	Intron 34	39292
19	39033547	39033677	39033613	39033563	39033638	0.004973	90.9369	+	Hells	Intron 36	28067
1	46132921	46133062	46132992	46132942	46133017	0.000646	90.6785	+	Dnah7b	Intron 4	9338
2	5300094	5300513	5300304	5300254	5300329	8.94E-15	88.7928	-	Camk1d	Intron 16	335198
3	120952743	120952932	120952838	120952788	120952863	5.76E-06	88.431	-	Tmem56	5' UTR	13261
11	17121293	17121486	17121390	17121340	17121415	2.45E-12	87.0851	+	Wdr92	Intron 4	9397
1	153210317	153210417	153210368	153210318	153210393	0.010454	86.6264	+	Ivns1abp	CDS 27	18689
5	147072577	147073026	147072802	147072752	147072827	0	86.2125	+	Cdk8	Intron 4	29326
4	138659707	138659948	138659828	138659778	138659853	0.000144	86.1809	-	Minos1	Intron 2	27081
2	102664412	102664574	102664494	102664444	102664519	0.000384	85.2042	-	Cd44	Promoter	134
16	57391233	57391881	57391558	57391508	57391583	0	85.0994	+	Filip1l	5' UTR	37843
1	102179024	102179168	102179097	102179047	102179122	0.004028	83.253	+	Cntnap5b	Intron 26	509682
3	37380432	37380775	37380604	37380554	37380629	3.73E-10	81.3966	+	Spata5	Intron 28	61230
6	145881279	145881638	145881459	145881409	145881484	0	80.9844	+	Sspn	Promoter	-1029
2	20821483	20821928	20821706	20821656	20821731	0	80.7815	-	Arhgap21	Intron 42	67421
14	105115009	105115285	105115148	105115098	105115173	0.001355	79.9078	+	D130009I18Rik	Non-coding	76636
1	94754438	94754641	94754540	94754490	94754565	0.005611	79.1872	+	Gpc1	Intron 16	26175
1	40497479	40497690	40497585	40497535	40497610	9.56E-06	78.9565	+	***Il1rl1***	CDS 3	670
10	104675385	104675669	104675528	104675478	104675553	1.78E-19	78.2731	-	Tmtc2	Intron 2	335867
13	113368871	113369030	113368951	113368901	113368976	2.63E-05	78.0149	-	Il31ra	5' UTR	2264
7	127897524	127897809	127897667	127897617	127897692	1.37E-08	77.6606	+	Vwa3a	Intron 6	14453
5	138043211	138043379	138043296	138043246	138043321	8.37E-08	76.8184	-	Mospd3	Promoter	-926
16	11144005	11144500	11144253	11144203	11144278	0	69.8232	-	Zc3h7a	Intron 8	31987
4	137067782	137067906	137067845	137067795	137067870	0.001127	69.7318	+	il	Intron 20	43064
9	105721643	105721971	105721808	105721758	105721833	0.000134	69.6151	-	Col6a6	5' UTR	8445
12	70259622	70259789	70259706	70259656	70259731	1.25E-05	67.8591	-	Rps29	Promoter	398
9	56108268	56108423	56108346	56108296	56108371	0.00826	66.3437	-	Peak1	Intron 2	194035
4	154931285	154931451	154931369	154931319	154931394	0.001511	64.5925	+	Gnb1	Intron 22	65815
9	78023133	78023285	78023210	78023160	78023235	4.31E-11	58.4502	+	C920006O11Rik	Promoter	-436
4	137796488	137796673	137796581	137796531	137796606	0.000132	39.1089	+	Hp1bp3	Intron 22	24829
2	71687443	71687543	71687494	71687444	71687519	0.000874	37.8601	+	Itga6	Intron 28	53633
8	87635776	87635915	87635846	87635796	87635871	0.000133	36.5308	-	Zfp791	Intron 2	11080
6	66985325	66985792	66985559	66985509	66985584	0.000165	10.5196	-	Gadd45a	Intron 2	1610
1	145848751	145848856	145848804	145848754	145848829	0.000254	9.33337	-	Rgs2	CDS 1	2424
1	71644940	71645122	71645032	71644982	71645057	0.000234	7.70047	-	Fn1	Promoter	0
1	71697143	71697359	71697252	71697202	71697277	1.47E-13	7.58851	-	Fn1	Promoter	0
X	84483702	84483851	84483777	84483727	84483802	1.97E-34	7.5081	-	Il1rapl1	Intron 12	877112
3	123209143	123209525	123209335	123209285	123209360	2.45E-15	7.22287	+	Prss12	CDS 21	59312
1	71692128	71692356	71692243	71692193	71692268	0.010727	7.08699	-	Fn1	Promoter	0
X	137077392	137077790	137077592	137077542	137077617	9.35E-06	7.03214	-	Tsc22d3	Intron 2	59354
6	4460690	4460839	4460765	4460715	4460790	3.67E-06	6.93808	+	Col1a2	Promoter	0
1	71694589	71694804	71694697	71694647	71694722	3.22E-16	6.64888	-	Fn1	Promoter	0
1	71642169	71642455	71642313	71642263	71642338	0.000967	6.62599	-	Fn1	Promoter	0
5	150423126	150423272	150423200	150423150	150423225	0.005703	6.61114	-	Hsph1	Intron 26	5838
1	71674626	71674804	71674716	71674666	71674741	3.21E-17	6.55322	-	Fn1	Promoter	0
1	71665965	71666208	71666087	71666037	71666112	0.001318	6.53232	-	Fn1	Promoter	0
1	71636679	71636869	71636775	71636725	71636800	0.006716	6.48742	-	Fn1	Promoter	0
1	71685039	71685226	71685133	71685083	71685158	2.76E-05	6.47908	-	Fn1	Promoter	0
11	75277217	75277312	75277265	75277215	75277290	1.71E-17	6.45	+	2210403K04Rik (mir22)	intron	4400
16	85056588	85056806	85056698	85056648	85056723	2.18E-06	6.35824	-	App	Intron 28	93643
1	71698101	71698289	71698196	71698146	71698221	5.03E-08	6.32261	+	Apol7d	Promoter	-83
1	71675897	71676149	71676024	71675974	71676049	8.78E-06	6.3092	-	Fn1	Promoter	0
1	71653285	71653528	71653407	71653357	71653432	0.002816	6.30568	-	Fn1	Promoter	0
1	71673741	71674009	71673876	71673826	71673901	0.000285	6.24395	-	Fn1	Promoter	0
1	82441124	82441367	82441246	82441196	82441271	0.001009	6.21431	+	Rhbdd1	Intron 12	60989
1	71683833	71684049	71683942	71683892	71683967	0.000207	6.12818	-	Fn1	Promoter	0
1	40498143	40498348	40498246	40498196	40498271	0.003664	6.03837	+	Il1rl1	Promoter	985
6	4477063	4477221	4477143	4477093	4477168	0.004109	6.00769	+	Col1a2	Promoter	0
2	164180619	164180797	164180709	164180659	164180734	0.000319	5.90563	-	Slpi	Intron 4	1447
6	4486605	4486767	4486687	4486637	4486712	9.68E-05	5.76707	+	Col1a2	Promoter	0
1	71646184	71646342	71646264	71646214	71646289	2.77E-07	5.73064	-	Fn1	Promoter	0
6	4478312	4478477	4478395	4478345	4478420	0.008054	5.63275	+	Col1a2	Promoter	0
1	71645793	71646046	71645920	71645870	71645945	0.000582	5.51901	-	Fn1	Promoter	0
6	4469835	4469973	4469905	4469855	4469930	0.00979	5.33815	+	Col1a2	Promoter	0
16	92111984	92112278	92112132	92112082	92112157	2.22E-05	5.29219	+	Mrps6	Promoter	0
1	71667304	71667471	71667388	71667338	71667413	0.000776	5.19607	-	Fn1	Promoter	0
6	4471402	4471548	4471476	4471426	4471501	0.001907	5.19357	+	Col1a2	Intron 2	0
1	71657510	71657626	71657569	71657519	71657594	0.012608	5.18874	-	Fn1	Promoter	0
2	152165488	152165715	152165602	152165552	152165627	0.00092	5.17045	-	Trib3	Intron 2	5343
1	82440715	82440905	82440811	82440761	82440836	0.003838	5.12601	+	Rhbdd1	Intron 12	60989
8	28223765	28223963	28223865	28223815	28223890	0.000508	5.10139	+	Gpr124	Intron 18	27452
14	66285263	66285606	66285435	66285385	66285460	1.15E-07	5.09326	+	Scara5	5' UTR	23
13	113368392	113368607	113368500	113368450	113368525	5.24E-42	5.08669	-	Il31ra	5' UTR	1841
2	22868729	22868906	22868818	22868768	22868843	0.009825	5.06385	-	Abi1	Intron 20	26856
3	50169831	50170523	50170178	50170128	50170203	0.000246	5.01284	-	Slc7a11	3' UTR	77013
19	5742400	5742524	5742463	5742413	5742488	0.004803	4.92974	+	Ltbp3	CDS 3	1496
6	124654424	124654705	124654565	124654515	124654590	1.44E-08	4.8615	+	Lpcat3	3' UTR	41302
7	28104649	28104820	28104735	28104685	28104760	0.005904	4.74043	-	Ltbp4	Intron 36	8360
11	50048118	50048297	50048208	50048158	50048233	0.001259	4.62333	+	Mgat4b	Intron 4	5383
14	51724529	51725120	51724825	51724775	51724850	0.000257	4.49571	+	***Rnase4***	CDS 3	13777
13	61036211	61036394	61036303	61036253	61036328	0.00068	4.48769	-	Ctla2a	Intron 2	1534
17	74256044	74256178	74256112	74256062	74256137	0.000937	4.41443	-	Xdh	Intron 30	43345
18	36913088	36913315	36913202	36913152	36913227	0.010271	4.34308	+	Ik	Intron 24	8778
16	15888006	15888528	15888268	15888218	15888293	4.69E-08	4.31702	+	Cebpd	CDS 1	627
10	76548019	76548199	76548110	76548060	76548135	0.004952	4.26868	-	Col18a1	Intron 8	57840
7	28110042	28110308	28110176	28110126	28110201	3.41E-05	4.22919	-	Ltbp4	Intron 36	8360
17	25885825	25886088	25885957	25885907	25885982	1.91E-05	4.22492	-	Msln	Promoter	0
16	85080090	85080310	85080201	85080151	85080226	0.004171	4.20002	-	***App***	Intron 2	218236
10	76529701	76529840	76529771	76529721	76529796	0.008815	4.1724	-	Col18a1	Intron 8	57840

7	28107049	28107221	28107136	28107086	28107161	0.007569	4.10103	-	Ltbp4	Intron 36	8360
15	101862277	101862515	101862397	101862347	101862422	3.14E-05	4.07275	+	Krt18	Promoter	0
14	66600159	66600346	66600253	66600203	66600278	4.65E-05	3.88715	+	Clu	3' UTR	12839
11	62418122	62418365	62418244	62418194	62418269	0.009551	3.84316	+	2410006H16Rik	Promoter	364
17	46161400	46161539	46161470	46161420	46161495	0.013498	3.81967	-	Vegfa	Promoter	575
18	38620175	38620335	38620256	38620206	38620281	4.57E-07	3.79739	+	Ndfip1	Intron 14	41546
2	152163190	152164454	152163823	152163773	152163848	4.95E-06	3.7753	-	Trib3	CDS 3	4082
2	179928692	179928815	179928754	179928704	179928779	0.00332	3.75721	-	Lama5	Intron 88	34444
11	62416743	62416844	62416794	62416744	62416819	0.005824	3.73335	+	2410006H16Rik	Promoter	364
4	131866804	131866919	131866862	131866812	131866887	0.000368	3.73335	+	Snhg12	Promoter	22
10	76543430	76543655	76543543	76543493	76543568	6.88E-05	3.70507	-	Col18a1	Intron 8	57840
7	20488946	20489059	20489003	20488953	20489028	2.65E-07	3.60738	-	Pvr	3' UTR	16681
17	46162288	46162532	46162411	46162361	46162436	0.000303	3.59212	-	Vegfa	Promoter	575
2	179912211	179912398	179912305	179912255	179912330	0.00722	3.59116	-	Lama5	Intron 88	34444
17	46168763	46169223	46168994	46168944	46169019	4.70E-11	3.56185	-	***Vegfa***	Intron 10	6795
8	96704010	96704303	96704157	96704107	96704182	1.44E-17	3.52754	+	***Mt1***	Intron 2	36
13	29044946	29045591	29045269	29045219	29045294	3.50E-15	3.50957	-	Sox4	Intergenic	?
10	127025472	127025709	127025591	127025541	127025616	0.005707	3.48849	-	Lrp1	Intron 74	64767
16	33919878	33920335	33920107	33920057	33920132	3.51E-05	3.47998	+	Itgb5	Intron 20	90127
2	33071572	33072012	33071793	33071743	33071818	3.15E-13	3.47653	+	Angptl2	5' UTR	91
1	82374143	82374290	82374217	82374167	82374242	0.0109	3.46947	+	Rhbdd1	Intron 12	60989
10	127012347	127012585	127012467	127012417	127012492	3.90E-06	3.41932	-	Lrp1	Intron 74	64767
10	76518336	76518611	76518474	76518424	76518499	7.88E-07	3.4183	-	Col18a1	Intron 8	57840
10	127021675	127021853	127021765	127021715	127021790	0.001453	3.41697	-	Lrp1	Intron 74	64767
10	127013874	127014160	127014018	127013968	127014043	4.19E-05	3.39111	-	Lrp1	Intron 74	64767
10	127005651	127005819	127005736	127005686	127005761	3.23E-06	3.36559	-	Lrp1	Intron 74	64767
2	179917070	179917221	179917146	179917096	179917171	0.014184	3.35894	-	Lama5	Intron 88	34444
17	56558833	56558989	56558912	56558862	56558937	0.010816	3.33398	-	Mir6977	Promoter	-500
10	127025106	127025282	127025195	127025145	127025220	0.002015	3.3052	-	Lrp1	Intron 74	64767
10	127045016	127045167	127045092	127045042	127045117	0.000478	3.2836	-	Lrp1	Intron 74	64767
2	179925937	179926121	179926030	179925980	179926055	5.16E-05	3.27401	-	Lama5	Intron 88	34444
1	55147946	55148089	55148018	55147968	55148043	0.000207	3.22458	+	Hspe1	Promoter	115
8	96696992	96697147	96697070	96697020	96697095	0.008149	3.20001	+	Mt2	Promoter	679
10	127013485	127013755	127013621	127013571	127013646	1.08E-08	3.18538	-	Lrp1	Intron 74	64767
17	25885567	25885817	25885693	25885643	25885718	1.42E-09	3.17963	-	Msln	Promoter	0
4	137071219	137071384	137071302	137071252	137071327	9.53E-06	3.17084	+	Hspg2	Intron 96	71178
4	137097562	137097723	137097643	137097593	137097668	0.012277	3.1679	+	Hspg2	Intron 96	71178
13	13455715	13455885	13455801	13455751	13455826	0.009047	3.12501	-	Gpr137b	Promoter	325
4	137063722	137063827	137063775	137063725	137063800	0.011803	3.12092	+	Hspg2	Intron 96	71178
1	151949733	151949992	151949863	151949813	151949888	1.82E-12	3.07024	+	Ptgs2	Promoter	0
2	179918036	179918210	179918124	179918074	179918149	0.013722	3.06006	-	Lama5	Intron 2	49048
4	155539556	155540041	155539799	155539749	155539824	5.35E-06	3.05868	-	Agrn	3' UTR	31557
3	95541736	95541857	95541797	95541747	95541822	0.002814	3.05265	-	Ecm1	Promoter	0
1	62862781	62862923	62862853	62862803	62862878	0.000627	3.04349	+	Nrp2	Intron 28	81735
4	137106262	137106434	137106349	137106299	137106374	0.002221	3.04176	+	Hspg2	Intron 96	71178
3	95541941	95542097	95542020	95541970	95542045	0.003869	3.0216	-	Ecm1	Promoter	0
10	127005417	127005588	127005503	127005453	127005528	0.001226	3.01513	-	Lrp1	Intron 74	64767
4	137108018	137108233	137108126	137108076	137108151	0.002199	2.98668	+	Hspg2	Intron 96	71178
1	151949734	151949951	151949843	151949793	151949868	0.008734	2.97609	+	Ptgs2	Promoter	0
17	71200226	71200519	71200373	71200323	71200398	0.000261	2.95456	-	Tgif1	Intron 2	8222
4	137074307	137074407	137074358	137074308	137074383	0.010418	2.94883	+	Hspg2	Intron 96	71178
12	31982499	31982681	31982591	31982541	31982616	0.000287	2.94239	+	Lamb1	Promoter	17
10	76541208	76541308	76541259	76541209	76541284	0.012843	2.92797	-	Col18a1	Intron 8	57840
4	137098152	137098321	137098237	137098187	137098262	0.001165	2.89387	+	Hspg2	Intron 96	71178
12	13461820	13462026	13461924	13461874	13461949	0.003713	2.88486	+	Nbas	Intron 80	185887
19	8783510	8783686	8783599	8783549	8783624	0.011257	2.86282	-	Slc3a2	Intron 6	14174
11	62416393	62416556	62416475	62416425	62416500	3.47E-07	2.86263	+	2410006H16Rik	Promoter	14
11	72261067	72261170	72261119	72261069	72261144	0.003585	2.83921	+	Mybbp1a	Promoter	0
11	88813728	88813918	88813824	88813774	88813849	1.13E-06	2.81363	-	Scpep1	Intron 4	26047
11	88790489	88790710	88790600	88790550	88790625	0.004017	2.80348	+	Scpep1os	Non-coding	23247
10	126987250	126987423	126987337	126987287	126987362	0.009524	2.80204	-	Lrp1	Intron 74	64767
2	166890940	166891040	166890991	166890941	166891016	9.72E-09	2.78331	+	1500012F01Rik	Intron 8	2506
2	179912730	179912951	179912841	179912791	179912866	0.000186	2.78315	-	Mir7005	Promoter	1578
3	95538081	95538452	95538267	95538217	95538292	4.65E-23	2.77173	-	**Mir7014**	Promoter	445
15	32963618	32963757	32963688	32963638	32963713	0.010618	2.76745	+	Sdc2	Intron 4	96387
15	102057825	102058071	102057949	102057899	102057974	0.01273	2.76333	-	Itgb7	Intron 4	15253
3	157557460	157557817	157557639	157557589	157557664	1.49E-11	2.74114	-	***Cth***	Intron 2	30211
10	85808476	85809631	85809054	85809004	85809079	0.001633	2.71314	+	Timp3	CDS 9	45319
15	102052527	102052671	102052600	102052550	102052625	0.007685	2.68818	-	Itgb7	Intron 4	15253
1	136302903	136303008	136302956	136302906	136302981	0.009006	2.68624	+	Cyb5r1	Promoter	336
14	73766136	73766408	73766273	73766223	73766298	0.000162	2.68509	-	Itm2b	Intron 8	18671
12	32008336	32008594	32008466	32008416	32008491	0.000199	2.68095	+	Lamb1	Promoter	17
11	50015909	50016174	50016042	50015992	50016067	0.003591	2.66403	-	Sqstm1	Promoter	0
4	137101438	137101606	137101523	137101473	137101548	1.12E-07	2.65923	+	Hspg2	Intron 96	71178
12	40929738	40930093	40929916	40929866	40929941	5.30E-13	2.65298	-	Ifrd1	Intron 2	19684
12	31950176	31950498	31950338	31950288	31950363	1.65E-06	2.62875	+	***Lamb1***	Intron 2	17
12	32011439	32011642	32011541	32011491	32011566	4.83E-06	2.62646	+	Lamb1	Promoter	17
1	59542297	59542397	59542348	59542298	59542373	0.0008	2.60466	+	Fzd7	3' UTR	2619
11	50021330	50021482	50021407	50021357	50021432	3.88E-05	2.59964	-	Sqstm1	Promoter	0
12	31963610	31963711	31963661	31963611	31963686	2.33E-06	2.58524	+	Lamb1	Promoter	17
12	31985877	31986134	31986006	31985956	31986031	0.000334	2.58259	+	Lamb1	Promoter	17
16	14472865	14473134	14473000	14472950	14473025	0.000312	2.57501	+	Abcc1	Intron 60	111214
13	24909864	24910039	24909952	24909902	24909977	1.19E-10	2.57096	-	Acot13	CDS 1	13320
4	125182078	125182304	125182192	125182142	125182217	0.005622	2.56776	+	Grik3	Intron 2	13255
6	91428206	91428384	91428296	91428246	91428321	1.95E-06	2.56605	+	Tmem43	Intron 6	4461
10	126992725	126992958	126992842	126992792	126992867	3.94E-06	2.5632	-	Lrp1	Intron 74	64767
14	45904769	45904893	45904832	45904782	45904857	0.014083	2.55943	-	Ero1l	Intron 30	11904
14	45926196	45926344	45926271	45926221	45926296	0.00182	2.54547	-	Ero1l	3' UTR	33355
8	85694621	85694753	85694688	85694638	85694713	3.51E-05	2.53882	+	Tbc1d9	Intron 2	5370
10	127004427	127004635	127004532	127004482	127004557	0.000285	2.52975	-	Lrp1	Intron 74	64767
1	151947244	151947361	151947303	151947253	151947328	3.62E-07	2.52712	-	7530420F21Rik	Promoter	-347
4	137105454	137105616	137105536	137105486	137105561	0.00615	2.51919	+	Hspg2	Intron 96	71178
15	76183282	76183453	76183368	76183318	76183393	0.011458	2.50804	+	Maf1	Intron 10	1558
10	59763530	59763703	59763617	59763567	59763642	0.000906	2.50491	+	Psap	Intron 8	15726
14	124245670	124245832	124245752	124245702	124245777	0.001848	2.49864	+	Itgbl1	CDS 21	313150
1	136307076	136307187	136307132	136307082	136307157	0.001235	2.49506	+	Cyb5r1	Promoter	336
11	5769383	5769670	5769527	5769477	5769552	0.010719	2.45784	+	Aebp1	Promoter	31

10	76551622	76551766	76551695	76551645	76551720	0.002119	2.45481	-	Col18a1	Intron 2	60245
1	151676995	151677365	151677181	151677131	151677206	0.000397	2.45477	-	Pla2g4a	Intron 2	131050
15	61820908	61821759	61821334	61821309	61821359	7.01E-22	2.61234	+	Myc	CDS 5	4012
4	131864615	131864863	131864740	131864690	131864765	4.41E-12	2.43985	+	Snhg12	Non-coding	2211
10	76016180	76016395	76016288	76016238	76016313	0.004284	2.4332	+	Lss	3' UTR	21808
10	126983064	126983268	126983167	126983117	126983192	0.000787	2.43119	-	Lrp1	Intron 74	64767
17	75760006	75760191	75760099	75760049	75760124	0.002237	2.42471	+	Ltbp1	Intron 56	355137
8	25548677	25549452	25549065	25549015	25549090	2.31E-11	2.42234	-	1810011O10Rik	CDS 1	0
4	142863848	142864019	142863934	142863884	142863959	3.31E-08	2.42197	-	Pdpn	Promoter	14
5	99372426	99372888	99372658	99372608	99372683	5.72E-17	2.41481	-	Prkg2	Intron 8	93211
10	126988730	126988875	126988803	126988753	126988828	0.000178	2.39954	-	Lrp1	Intron 74	64767
1	155102333	155102524	155102429	155102379	155102454	6.22E-05	2.39395	-	Lamc1	Promoter	442
11	51433249	51433453	51433352	51433302	51433377	1.40E-07	2.3878	+	Nhp2	Promoter	0
13	13557583	13557870	13557727	13557677	13557752	4.37E-05	2.38616	+	Nid1	Intron 6	27714
4	140857173	140857314	140857244	140857194	140857269	8.18E-07	2.38516	+	Epha2	Promoter	37
14	56290640	56290740	56290691	56290641	56290716	0.007795	2.36972	-	Nedd8	Promoter	4
13	13453143	13453339	13453242	13453192	13453267	6.49E-07	2.34258	-	Gpr137b	Intron 2	32553
4	137075804	137075904	137075855	137075805	137075880	0.005008	2.33782	+	Hspg2	Intron 96	71178
15	102048933	102049171	102049053	102049003	102049078	0.00269	2.33775	-	Itgb7	Intron 4	15253
10	126992008	126992235	126992122	126992072	126992147	0.002614	2.33199	-	Lrp1	Intron 74	64767
13	13485411	13485567	13485490	13485440	13485515	0.014064	2.33002	-	Gpr137b	Promoter	325
19	9076196	9076343	9076270	9076220	9076295	0.008107	2.32378	+	Ahnak	Intron 8	12994
4	142889098	142889397	142889248	142889198	142889273	7.40E-08	2.31391	-	Pdpn	Promoter	14
1	155076180	155076365	155076273	155076223	155076298	0.008687	2.31093	-	Lamc1	Promoter	442
15	102046910	102047114	102047013	102046963	102047038	8.16E-06	2.30732	-	Itgb7	Intron 2	15608
2	131737937	131738054	131737996	131737946	131738021	8.26E-05	2.29543	+	Prn	5' UTR	2273
10	98725888	98726749	98726319	98726269	98726344	7.09E-09	2.28795	+	Dusp6	Intron 2	23
9	118584078	118584193	118584136	118584086	118584161	7.28E-06	2.28709	+	Itga9	Intron 22	68251
5	122017808	122018054	122017932	122017882	122017957	1.07E-08	2.27958	-	Aldh2	Intron 4	24749
10	76173727	76173893	76173811	76173761	76173836	0.002486	2.27856	-	Col6a1	Intron 6	14897
10	126993272	126993438	126993356	126993306	126993381	0.000845	2.27762	-	Lrp1	Intron 74	64767
3	95538141	95538419	95538281	95538231	95538306	3.82E-13	2.27618	-	Ecm1	Promoter	0
5	122026476	122026608	122026543	122026493	122026568	0.00237	2.26657	-	Aldh2	Intron 4	24749
10	59757289	59757560	59757425	59757375	59757450	0.00012	2.2649	+	Psap	Intron 8	15726
7	57017439	57017552	57017496	57017446	57017521	0.000193	2.26438	+	Htatip2	Intron 4	2557
17	35402734	35402844	35402790	35402740	35402815	0.0088	2.2619	+	LOC547349	CDS 1	10
14	73764354	73764580	73764468	73764418	73764493	0.007232	2.26166	-	Itm2b	Promoter	0
7	28244293	28244428	28244361	28244311	28244386	0.00048	2.25985	+	Blvrb	CDS 3	11296
4	33333092	33333463	33333278	33333228	33333303	0.007125	2.25751	-	Pnrc1	CDS 1	2300
4	137095896	137096024	137095961	137095911	137095986	0.007407	2.22786	+	Hspg2	Intron 96	71178
11	88795902	88796044	88795974	88795924	88795999	0.005351	2.22397	+	Scpep1os	Non-coding	23247
17	75752034	75752226	75752131	75752081	75752156	0.001014	2.22035	+	Ltbp1	Intron 56	355137
6	7627238	7627390	7627315	7627265	7627340	0.012827	2.21961	-	Asns	Intron 8	15793
17	56762569	56762677	56762624	56762574	56762649	0.005661	2.21783	-	Lonp1	Intron 34	3650
10	59762202	59762391	59762297	59762247	59762322	7.08E-07	2.21405	+	Psap	Intron 8	15726
4	140878126	140878378	140878253	140878203	140878278	0.006415	2.21054	+	Epha2	Promoter	37
1	165191980	165192164	165192073	165192023	165192098	0.000982	2.20851	-	Prrx1	Promoter	873
8	34799791	34799960	34799876	34799826	34799901	2.98E-05	2.20346	+	Gsr	Intron 20	36081
6	91435318	91435465	91435392	91435342	91435417	0.006941	2.20254	+	Tmem43	Intron 6	4461
6	129491129	129492214	129491672	129491622	129491697	0.010912	2.19971	+	Gabarapl1	Intron 2	61
13	13594653	13594853	13594754	13594704	13594779	0.001801	2.19755	+	Nid1	Intron 6	27714
12	31984796	31984965	31984881	31984831	31984906	2.15E-07	2.19525	+	Lamb1	Promoter	17
4	140880155	140880352	140880254	140880204	140880279	2.43E-06	2.19185	+	Epha2	Promoter	37
16	57627128	57627393	57627261	57627211	57627286	0.000128	2.18411	-	Col8a1	CDS 1	127458
13	5868274	5868374	5868325	5868275	5868350	0.012979	2.17808	+	Klf6	3' UTR	7870
4	107552383	107552556	107552470	107552420	107552495	0.000971	2.17779	+	Magoh	Promoter	23
4	140876406	140876564	140876486	140876436	140876511	0.00206	2.17655	+	Epha2	Promoter	37
11	50020670	50020864	50020768	50020718	50020793	0.00321	2.17618	-	Sqstm1	Promoter	0
10	59756102	59756293	59756198	59756148	59756223	0.001696	2.17128	+	Psap	Intron 8	15726
4	137520607	137520773	137520691	137520641	137520716	0.009572	2.17102	+	Ece1	3' UTR	102455
1	87791182	87791320	87791252	87791202	87791277	1.59E-05	2.17102	+	Itm2c	Promoter	97
2	71279231	71279457	71279345	71279295	71279370	0.000204	2.16836	+	Hat1	CDS 21	51914
17	75689361	75689538	75689450	75689400	75689475	0.002171	2.14992	+	Ltbp1	Intron 44	173884
19	8782072	8782635	8782354	8782304	8782379	2.55E-07	2.13656	-	Slc3a2	Intron 2	15225
10	76171559	76172914	76172237	76172187	76172262	3.16E-08	2.13384	-	Col6a1	Intron 6	14897
11	50021333	50021455	50021395	50021345	50021420	3.66E-05	2.11869	-	Sqstm1	Promoter	0
10	59754394	59754578	59754487	59754437	59754512	0.001258	2.11512	+	Psap	Intron 8	15726
7	138127109	138127342	138127226	138127176	138127251	0.0001	2.10759	+	Htra1	Intron 8	43103
10	58810933	58811163	58811049	58810999	58811074	0.000105	2.10396	+	P4ha1	Intron 10	20367
11	88808429	88808608	88808519	88808469	88808544	0.001055	2.10065	-	Scpep1	Intron 4	26047
12	111858534	111858647	111858591	111858541	111858616	0.008336	2.09914	+	Dync1h1	Intron 126	57702
10	21717115	21717305	21717211	21717161	21717236	0.003732	2.0886	+	Sgk1	Intron 26	116431
10	59762189	59762356	59762273	59762223	59762298	0.010108	2.08632	+	Psap	Intron 8	15726
12	111868960	111869092	111869027	111868977	111869052	0.000794	2.08579	+	Dync1h1	Intron 126	57702
1	155099099	155099288	155099194	155099144	155099219	0.008942	2.07946	-	Lamc1	Promoter	442
13	44964948	44965485	44965217	44965167	44965242	0	2.07813	+	Jarid2	Intron 6	138308
1	155081584	155081827	155081706	155081656	155081731	0.002217	2.07635	-	Lamc1	Promoter	442
13	13599884	13600084	13599985	13599935	13600010	0.000864	2.07007	+	Nid1	Intron 6	27714
2	35170783	35171570	35171177	35171127	35171202	0.004844	2.06451	-	Stom	CDS 1	20960
5	32459259	32460113	32459687	32459637	32459712	8.05E-06	2.0634	+	Fosl2	3' UTR	20414
11	5769717	5769910	5769814	5769764	5769839	0.004311	2.06317	+	Aebp1	Promoter	31
4	137115011	137115191	137115102	137115052	137115127	2.57E-05	2.06125	+	Hspg2	Intron 96	71178
1	78695578	78695747	78695663	78695613	78695688	5.69E-06	2.05001	+	Acsl3	Intron 28	47416
14	37943832	37944018	37943926	37943876	37943951	0.000355	2.04763	-	Ghitm	Intron 16	1287
12	93047719	93048052	93047886	93047836	93047911	0.003515	2.03969	-	Sel1l	3' UTR	39546
9	77623940	77624144	77624043	77623993	77624068	5.76E-05	2.0266	+	Gclc	Intron 6	21598
12	8928863	8929103	8928984	8928934	8929009	9.57E-07	2.02428	+	Laptm4a	Promoter	754
17	56558832	56558948	56558891	56558841	56558916	0.000507	2.02411	-	Ptprs	Intron 22	56915
6	13035413	13037293	13036354	13036304	13036379	1.10E-08	2.01016	+	Tmem106b	CDS 17	14439
7	149568504	149568690	149568598	149568548	149568623	0.014236	2.00843	-	Ctsd	Intron 8	10342
17	35969456	35969646	35969552	35969502	35969577	1.36E-05	2.00422	+	Flot1	CDS 25	9154
2	167515594	167515775	167515685	167515635	167515710	0.009229	2.00237	+	Cebpb	3' UTR	1179
