# Supplementary material for: MSI2 promotes translation of multiple IRES-containing oncogenes and virus to induce self-renewal of tumor initiating stem-like cells
Source: Cell Death Discov. 2023 Apr 28;9:141. doi: 10.1038/s41420-023-01427-9 (PMC10147607; doi:10.1038/s41420-023-01427-9)
Supplement: Supplementary file 8 — Supplementary Table 3 [file 41420_2023_1427_MOESM8_ESM.pdf]

Table S3. Overlap of RIP-seq from this publication with GSE93210, GSE54598 & GSE62115

	MSI2_Clipseq_This publication							GSE93210_MSI2_iCLIP_K562 human converted to mouse genome		
chr peak	Peak start	Peak end	Gene Symb	Scaled FC	p-value	strand	Chr	start	end	score
chr11	62418122	62418365	2410006H1	3.73335	0.009551	+	chr11	62418154	62418197	110
chr13	24909802	24910102	Acot13	2.57096	1.19E-10	-	chr13	24910028	24910047	18
chr6	7625232	7625532	Asns	2.21961	7.17E-10	-	chr6	7625231	7625245	-127
chr19	5797579	5797879	Malat1	2.17779	0.00035	-	chr19	5797791	5798515	9981
chr2	35171027	35171327	Stom	2.06451	0.004844	-	chr2	35171019	35171051	-112
chr14	37943832	37944018	Ghitm	2.04763	0.000355	-	chr14	37943884	37943957	82
chr6	13036204	13036504	Tmem106b	2.01016	1.1E-08	+	chr6	13036349	13036372	35
chr17	35969402	35969702	Flot1	2.00422	1.36E-05	+	chr17	35969684	35969688	-14
chr5	74937948	74938248	Fip1l1	1.97981	0.004938	+	chr5	74937961	74937971	8
chr1	1.56E+08	1.56E+08	Glul	1.97109	0.000219	+	chr1	1.56E+08	1.56E+08	-377
chr8	26060729	26061029	Adam9	1.8827	0.000361	-	chr8	26061001	26061032	29
chr4	1.34E+08	1.34E+08	Tmem50a	1.8439	2.2E-12	-	chr4	1.34E+08	1.34E+08	357
chr7	1.5E+08	1.5E+08	Tssc4	1.81546	0.012635	+	chr7	1.5E+08	1.5E+08	39
chr17	28537671	28537971	Fkbp5	1.79135	1.72E-10	-	chr17	28537654	28537671	-26
chr15	96519129	96519429	Slc38a2	1.78346	4.43E-11	-	chr15	96519206	96519260	-340
chr2	1.63E+08	1.63E+08	Serinc3	1.76438	0.000123	-	chr2	1.63E+08	1.63E+08	-206
chr11	5424994	5425294	Xbp1	1.73346	0.00536	+	chr11	5425273	5425291	-32
chr8	96704007	96704307	Mt1	1.72441	1.44E-17	+	chr8	96704145	96704152	7
chrX	1.52E+08	1.52E+08	Sat1	1.63732	6.99E-09	-	chrX	1.52E+08	1.52E+08	50
chr2	1.03E+08	1.03E+08	Cat	1.56476	0.001071	-	chr2	1.03E+08	1.03E+08	150
chr12	40929766	40930066	Ifrd1	1.56304	5.3E-13	-	chr12	40929768	40929899	185
chr15	80085881	80086181	Atf4	1.53789	9.89E-12	+	chr15	80085928	80085986	87
chr5	1.09E+08	1.09E+08	Tmed5	1.48493	2.63E-06	-	chr5	1.09E+08	1.09E+08	-89
chr9	75347215	75347515	Tmod3	1.47591	7.63E-05	-	chr9	75347419	75347429	42
chr16	23113385	23113685	Eif4a2	1.45394	0.002508	+	chr16	23113558	23113583	73
chr8	35233346	35233646	Tmem66	1.42858	2.54E-05	+	chr8	35233602	35233616	-8
chr1	43180129	43180429	Fhl2	1.35464	0.001318	-	chr1	43180146	43180159	-67
chr14	27466406	27466569	Arf4	1.3139	0.012705	+	chr14	27466471	27466473	15
chr7	1.07E+08	1.07E+08	Rps3	1.26232	9.85E-41	-	chr7	1.07E+08	1.07E+08	-34
chr3	1.01E+08	1.01E+08	Atp1a1	1.25257	1.13E-07	-	chr3	1.01E+08	1.01E+08	28
chr3	1.38E+08	1.38E+08	Adh5	1.24466	4.04E-07	+	chr3	1.38E+08	1.38E+08	-46
chr10	82347241	82347358	Txnrd1	1.17623	5.16E-06	+	chr10	82347255	82347277	36
chr9	98488470	98488734	Copb2	1.11102	5.89E-09	+	chr9	98488590	98488592	-47
chr14	27466154	27466279	Arf4	1.28942	1.01E-06	+	chr14	27466192	27466200	7
chr4	1.01E+08	1.01E+08	Leprot	1.23828	1.15E-05	+	chr4	1.01E+08	1.01E+08	581
chr13	24894219	24894335	BC005537	1.22931	0.000371	+	chr13	24894224	24894240	-61
chr9	55995205	55995399	Tspan3	1.22457	1.04E-05	-	chr9	55995250	55995292	-49
chr9	77830970	77831126	Elovl5	1.22392	0.011788	+	chr9	77831070	77831129	-317
chr1	52242473	52242749	Gls	1.20847	1.52E-06	-	chr1	52242716	52242738	48
chr7	1.21E+08	1.21E+08	Copb1	1.20158	0.012192	-	chr7	1.21E+08	1.21E+08	-57
chr7	1.07E+08	1.07E+08	Rps3	1.20076	2.32E-07	-	chr7	1.07E+08	1.07E+08	90
chr3	1.22E+08	1.22E+08	Gclm	1.19346	0.012359	+	chr3	1.22E+08	1.22E+08	-139
chr19	5422830	5423054	4930481A1	1.19	0.006375	+	chr19	5422864	5422883	94
chr17	13139150	13139264	Acat2	1.19	0.007443	-	chr17	13139193	13139198	22
chr1	1.27E+08	1.27E+08	Actr3	1.19	0.009141	-	chr1	1.27E+08	1.27E+08	39
chr3	1.38E+08	1.38E+08	Adh5	1.19	0.001833	+	chr3	1.38E+08	1.38E+08	-46
chr5	31208904	31209062	Agbl5	1.19	9.52E-05	+	chr5	31208928	31209019	-193
chr14	21275541	21275838	Anxa7	1.19	2.09E-05	-	chr14	21275648	21275669	-45
chr2	94253016	94253125	Api5	1.19	0.000543	-	chr2	94253006	94253075	196
chr14	27472596	27472722	Arf4	1.19	5.93E-08	+	chr14	27472594	27472615	-71
chr12	70476129	70476233	Arf6	1.19	0.000291	+	chr12	70476127	70476169	141
chr5	1.46E+08	1.46E+08	Arpc1a	1.19	0.000444	+	chr5	1.46E+08	1.46E+08	26
chr5	1.23E+08	1.23E+08	Arpc3	1.19	1.57E-07	+	chr5	1.23E+08	1.23E+08	-23
chr15	1E+08	1E+08	Atf1	1.19	0.008255	+	chr15	1E+08	1E+08	54
chr15	80085953	80086113	Atf4	1.19	1.47E-07	+	chr15	80085928	80085986	87
chr3	1.01E+08	1.01E+08	Atp1a1	1.19	5.05E-07	-	chr3	1.01E+08	1.01E+08	28
chr15	1.02E+08	1.02E+08	Atp5g2	1.19	2.48E-13	-	chr15	1.02E+08	1.02E+08	-104
chrX	71549657	71549823	Atp6ap1	1.19	0.003366	+	chrX	71549629	71549814	198
chr10	1.12E+08	1.12E+08	Atxn7l3b	1.19	0.002017	-	chr10	1.12E+08	1.12E+08	39
chr7	1.46E+08	1.46E+08	Bnip3	1.19	0.004261	-	chr7	1.46E+08	1.46E+08	-103
chr13	99083057	99083309	Btf3	1.19	1.41E-07	-	chr13	99083074	99083108	51
chr7	1.39E+08	1.39E+08	Bub3	1.19	0.002031	+	chr7	1.39E+08	1.39E+08	303
chr11	50111626	50111809	Canx	1.19	0.003016	-	chr11	50111664	50111665	19
chr2	1.04E+08	1.04E+08	Caprin1	1.19	0.002913	-	chr2	1.04E+08	1.04E+08	69
chr3	36467842	36468050	Ccna2	1.19	5.48E-06	-	chr3	36467938	36467993	-195
chr13	1.02E+08	1.02E+08	Ccnb1	1.19	0.000238	-	chr13	1.02E+08	1.02E+08	98
chr11	40564737	40564913	Ccng1	1.19	1.1E-06	-	chr11	40564798	40564823	69
chr11	22895938	22896099	Cct4	1.19	0.008079	+	chr11	22896016	22896028	-10
chr11	22899043	22899223	Cct4	1.19	8.65E-10	+	chr11	22899100	22899132	-124
chr15	31520580	31520866	Cct5	1.19	0.005788	-	chr15	31520485	31520689	608
chr5	1.3E+08	1.3E+08	Cct6a	1.19	0.000326	+	chr5	1.3E+08	1.3E+08	23
chr5	1.3E+08	1.3E+08	Cct6a	1.19	2.65E-07	+	chr5	1.3E+08	1.3E+08	-16
chr16	87486422	87486655	Cct8	1.19	7.88E-05	-	chr16	87486493	87486512	-29
chr16	87487736	87488019	Cct8	1.19	3.96E-06	-	chr16	87487789	87487801	-29
chr10	68808832	68809034	Cdk1	1.19	0.003234	-	chr10	68808906	68808929	60
chr10	68805293	68805517	Cdk1	1.19	0.00184	-	chr10	68805447	68805461	23
chr13	81935661	81935859	Cetn3	1.19	6.94E-06	+	chr13	81935849	81935916	-329
chr5	1.3E+08	1.3E+08	Chchd2	1.19	3.45E-25	-	chr5	1.3E+08	1.3E+08	-241
chr2	18597472	18597750	Commd3	1.19	0.000682	+	chr2	18597745	18597759	9
chr7	1.21E+08	1.21E+08	Copb1	1.19	2.51E-05	-	chr7	1.21E+08	1.21E+08	-57
chr9	98488582	98488777	Copb2	1.19	2.38E-05	+	chr9	98488590	98488592	-47
chr6	87862173	87862457	Copg1	1.19	0.00034	+	chr6	87862456	87862475	50
chr5	1.01E+08	1.01E+08	Coq2	1.19	0.007217	-	chr5	1.01E+08	1.01E+08	-13
chr9	79603187	79603418	Cox7a2	1.19	0.000151	-	chr9	79603241	79603260	-73
chr2	1.67E+08	1.67E+08	Cse1l	1.19	3.83E-05	+	chr2	1.67E+08	1.67E+08	23
chr1	1.3E+08	1.3E+08	Dars	1.19	1.31E-05	-	chr1	1.3E+08	1.3E+08	-666
chr1	1.22E+08	1.22E+08	Dbi	1.19	3.61E-09	-	chr1	1.22E+08	1.22E+08	7
chr10	1.27E+08	1.27E+08	Ddit3	1.19	3.18E-07	+	chr10	1.27E+08	1.27E+08	85
chr12	13237105	13237207	Ddx1	1.19	0.008351	-	chr12	13237144	13237164	21
chr12	13250674	13250784	Ddx1	1.19	0.014556	-	chr12	13250735	13250745	18
chr10	62051391	62051604	Ddx21	1.19	0.003213	-	chr10	62051457	62051509	138
chrX	12866938	12867151	Ddx3x	1.19	0.0143	+	chrX	12866990	12867015	60
chrX	12865873	12866116	Ddx3x	1.19	0.001675	+	chrX	12865913	12865921	9
chr9	44448005	44448221	Ddx6	1.19	0.003005	+	chr9	44448128	44448221	-222
chr13	47194720	47194879	Dek	1.19	0.000135	-	chr13	47194744	47194814	-238
chr13	47183370	47183592	Dek	1.19	0.000373	-	chr13	47183480	47183515	-64
chr13	47193531	47193765	Dek	1.19	0.000141	-	chr13	47193597	47193607	-14
chr1	1.55E+08	1.55E+08	Dhx9	1.19	0.000433	-	chr1	1.55E+08	1.55E+08	53
chrX	72354423	72354651	Dkc1	1.19	0.002318	+	chrX	72354394	72354453	416
chr4	40679801	40680075	Dnaja1	1.19	0.003687	+	chr4	40680054	40680119	168
chr14	21204506	21204691	Dnajc9	1.19	0.000517	-	chr14	21204547	21204569	-69
chr17	74698952	74699172	Dpy30	1.19	0.000276	-	chr17	74699046	74699078	-41
chr12	79980953	79981060	Eif2s1	1.19	0.000709	+	chr12	79980949	79980984	40
chr7	1.16E+08	1.16E+08	Eif3f	1.19	4.11E-09	+	chr7	1.16E+08	1.16E+08	3
chr15	51621547	51621726	Eif3h	1.19	6.69E-05	-	chr15	51621572	51621603	-80

chr15	78924638	78924883	Eif3l	1.19	4.97E-06	+	chr15	78924694	78924708	26
chr7	1.18E+08	1.18E+08	Eif4g2	1.19	0.011282	-	chr7	1.18E+08	1.18E+08	-24
chr5	1.35E+08	1.35E+08	Eif4h	1.19	3.22E-18	-	chr5	1.35E+08	1.35E+08	-26
chr15	44267202	44267378	Eny2	1.19	6.22E-07	+	chr15	44267256	44267266	91
chr1	1.87E+08	1.87E+08	Eprs	1.19	0.000744	+	chr1	1.87E+08	1.87E+08	-45
chr1	1.87E+08	1.87E+08	Eprs	1.19	9.5E-08	+	chr1	1.87E+08	1.87E+08	-85
chr16	36043944	36044220	Fam162a	1.19	4.93E-06	-	chr16	36044036	36044058	22
chr1	34871692	34871988	Fam168b	1.19	0.011105	-	chr1	34871733	34871823	-227
chr19	6059307	6059596	Fau	1.19	3.94E-05	+	chr19	6059458	6059488	-161
chr1	1.78E+08	1.78E+08	Fh1	1.19	0.01088	-	chr1	1.78E+08	1.78E+08	-171
chr17	28543039	28543217	Fkbp5	1.19	0.002614	-	chr17	28543163	28543181	-12
chr7	1.35E+08	1.35E+08	Fus	1.19	4.71E-13	+	chr7	1.35E+08	1.35E+08	47
chr3	33963053	33963261	Fxr1	1.19	0.002852	+	chr3	33963135	33963136	5
chr6	54987995	54988209	Gars	1.19	0.000245	+	chr6	54988056	54988057	2
chr16	91623147	91623281	Gart	1.19	0.004971	-	chr16	91623196	91623278	-93
chr15	34370944	34371141	Gm12191	1.19	0.009463	-	chr15	34371100	34371105	-37
chrX	1.39E+08	1.39E+08	Gm5643	1.19	3.51E-07	+	chrX	1.39E+08	1.39E+08	-2
chr13	24843898	24844095	Gmnn	1.19	6.02E-05	-	chr13	24843877	24843920	299
chr11	48617662	48617887	Gnb2l1	1.19	3.94E-09	+	chr11	48617700	48617704	-9
chr11	48618969	48619243	Gnb2l1	1.19	9.02E-06	+	chr11	48619058	48619080	-133
chr10	79518926	79519194	Gpx4	1.19	0.001442	+	chr10	79518955	79518962	119
chr4	1.25E+08	1.25E+08	Grik3	1.19	0.005622	+	chr4	1.25E+08	1.25E+08	10
chr9	69870251	69870511	Gtf2a2	1.19	2.46E-07	+	chr9	69870476	69870494	-347
chr2	71279231	71279457	Hat1	1.19	0.000204	+	chr2	71279335	71279350	19
chr10	36721339	36721561	Hdac2	1.19	9.14E-08	+	chr10	36721502	36721539	-68
chr13	97428833	97429095	Hmgcr	1.19	6.46E-05	-	chr13	97429031	97429052	15
chr6	51419644	51419922	Hnrnpa2b1	1.19	1.87E-11	-	chr6	51419871	51419875	-4
chr5	1E+08	1E+08	Hnrnpd	1.19	0.00218	-	chr5	1E+08	1E+08	-19
chr5	1E+08	1E+08	Hnrnpd	1.19	1.72E-10	-	chr5	1E+08	1E+08	28
chr13	58494510	58494785	Hnrnpk	1.19	0.000165	-	chr13	58494567	58494575	28
chr17	33802442	33802629	Hnrnpm	1.19	0.000508	-	chr17	33802542	33802567	36
chr1	1.8E+08	1.8E+08	Hnrnpu	1.19	5.28E-05	-	chr1	1.8E+08	1.8E+08	-49
chr8	1.22E+08	1.22E+08	Hsbp1	1.19	0.007306	+	chr8	1.22E+08	1.22E+08	11
chr12	1.12E+08	1.12E+08	Hsp90aa1	1.19	0.001319	-	chr12	1.12E+08	1.12E+08	-18
chr17	45708721	45708948	Hsp90ab1	1.19	8.84E-14	-	chr17	45708864	45708865	11
chr10	86166196	86166429	Hsp90b1	1.19	2.18E-07	-	chr10	86166247	86166346	301
chr1	55147912	55148173	Hspe1	1.19	1.37E-07	+	chr1	55147917	55147988	164
chr12	40929875	40930070	Ifrd1	1.19	0.002078	-	chr12	40929768	40929899	185
chr7	1.17E+08	1.17E+08	Ipo7	1.19	0.002885	+	chr7	1.17E+08	1.17E+08	-31
chr7	1.17E+08	1.17E+08	Ipo7	1.19	0.000979	+	chr7	1.17E+08	1.17E+08	43
chr7	87888686	87888878	Iqgap1	1.19	0.006554	-	chr7	87888822	87888823	9
chr18	6213210	6213312	Kif5b	1.19	0.000148	-	chr18	6213219	6213248	-40
chr12	32014228	32014485	Lamb1	1.19	8.49E-07	+	chr12	32014319	32014334	-7
chr7	54109271	54109567	Ldha	1.19	1.88E-05	+	chr7	54109429	54109443	65
chr2	1.1E+08	1.1E+08	Lin7c	1.19	0.001471	+	chr2	1.1E+08	1.1E+08	-219
chr18	56888951	56889080	Lmnb1	1.19	0.007344	+	chr18	56889003	56889004	44
chr2	41844235	41844463	Lrp1b	1.19	0.000516	-	chr2	41844231	41844255	64
chr5	1.06E+08	1.06E+08	Lrrc8d	1.19	0.005962	+	chr5	1.06E+08	1.06E+08	100
chr17	75758636	75758823	Ltbp1	1.19	0.002169	+	chr17	75758629	75758647	103
chr17	35253151	35253341	Ly6g5b	1.19	3.93E-05	-	chr17	35253164	35253248	651
chr4	1.08E+08	1.08E+08	Magoh	1.19	0.002957	+	chr4	1.08E+08	1.08E+08	-107
chr19	5797085	5797256	Malat1	1.19	0.004278	-	chr19	5797042	5797769	8697
chr16	17026457	17026600	Mapk1	1.19	0.009204	+	chr16	17026594	17026651	-42
chr1	1.33E+08	1.33E+08	Mapkapk2	1.19	0.000135	-	chr1	1.33E+08	1.33E+08	27
chr2	1.54E+08	1.54E+08	Mapre1	1.19	0.003186	+	chr2	1.54E+08	1.54E+08	37
chr3	95466705	95466849	Mcl1	1.19	0.001923	+	chr3	95466780	95466787	-77
chr10	1.17E+08	1.17E+08	Mdm2	1.19	0.001076	-	chr10	1.17E+08	1.17E+08	148
chr5	45916399	45916699	Med28	1.19	4.43E-06	+	chr5	45916680	45916693	66
chr4	1.39E+08	1.39E+08	Minos1	1.19	0.000144	-	chr4	1.39E+08	1.39E+08	15
chr11	96909656	96909826	Mrpl10	1.19	0.00427	+	chr11	96909772	96909787	-19
chr15	55365672	55365900	Mrpl13	1.19	0.000714	-	chr15	55365680	55365718	-53
chr10	1.28E+08	1.28E+08	Myl6	1.19	0.000713	-	chr10	1.28E+08	1.28E+08	253
chr10	1.27E+08	1.27E+08	Naca	1.19	4.17E-05	+	chr10	1.27E+08	1.27E+08	-6
chr10	1.27E+08	1.27E+08	Naca	1.19	5.42E-08	+	chr10	1.27E+08	1.27E+08	-77
chr10	1.27E+08	1.27E+08	Naca	1.19	0.000619	+	chr10	1.27E+08	1.27E+08	-39
chr10	1.11E+08	1.11E+08	Nap1l1	1.19	0.003156	+	chr10	1.11E+08	1.11E+08	-53
chr1	88247388	88247674	Ncl	1.19	0.000803	-	chr1	88247592	88247598	-5
chr6	11850380	11850654	Ndufa4	1.19	2.03E-13	-	chr6	11850486	11850536	-102
chr15	82180607	82180818	Ndufa6	1.19	2.86E-07	-	chr15	82180632	82180653	-27
chr13	43497172	43497318	Nol7	1.19	1.28E-05	+	chr13	43497205	43497297	63
chr11	33053906	33054127	Npm1	1.19	0.004113	-	chr11	33054025	33054029	11
chr8	96543371	96543632	Nudt21	1.19	0.000321	-	chr8	96543439	96543460	-80
chr6	35197290	35197469	Nup205	1.19	0.000849	+	chr6	35197346	35197372	41
chr10	58834332	58834479	P4ha1	1.19	0.00758	+	chr10	58834393	58834404	-50
chr15	36530632	36530837	Pabpc1	1.19	8.8E-06	-	chr15	36530631	36530649	17
chr15	36532609	36532818	Pabpc1	1.19	3.67E-05	-	chr15	36532611	36532614	7
chr15	36530281	36530525	Pabpc1	1.19	0.013476	-	chr15	36530357	36530370	15
chr5	77385574	77385827	Paics	1.19	0.007594	+	chr5	77385623	77385628	15
chr16	32018941	32019051	Pak2	1.19	0.004271	-	chr16	32018985	32019040	284
chr4	1.5E+08	1.5E+08	Park7	1.19	0.00748	-	chr4	1.5E+08	1.5E+08	155
chr2	1.22E+08	1.22E+08	Patl2	1.19	0.000101	-	chr2	1.22E+08	1.22E+08	501
chr2	1.21E+08	1.21E+08	Pdia3	1.19	0.003107	+	chr2	1.21E+08	1.21E+08	91
chr12	17277205	17277398	Pdia6	1.19	5.09E-12	+	chr12	17277302	17277315	-59
chr4	1.3E+08	1.3E+08	Pef1	1.19	0.001385	+	chr4	1.3E+08	1.3E+08	-11
chr4	1.49E+08	1.49E+08	Pgd	1.19	0.004977	-	chr4	1.49E+08	1.49E+08	2
chr1	74334863	74335001	Pnkd	1.19	0.000124	+	chr1	74334883	74334892	-28
chr10	61129664	61129808	Ppa1	1.19	0.004721	+	chr10	61129726	61129740	-9
chr5	1.23E+08	1.23E+08	Ppp1cc	1.19	4.26E-05	+	chr5	1.23E+08	1.23E+08	-378
chr1	1.35E+08	1.35E+08	Ppp1r15b	1.19	6.13E-05	+	chr1	1.35E+08	1.35E+08	-51
chr12	1.12E+08	1.12E+08	Ppp2r5c	1.19	0.006439	+	chr12	1.12E+08	1.12E+08	125
chr11	17098809	17099024	Ppp3r1	1.19	0.00239	+	chr11	17098920	17098926	-23
chr3	1.09E+08	1.09E+08	Prpf38b	1.19	0.002316	-	chr3	1.09E+08	1.09E+08	15
chr17	35286137	35286367	Prrc2a	1.19	1.53E-07	-	chr17	35286152	35286161	7
chr13	14717625	14717846	Psma2	1.19	3.33E-07	+	chr13	14717783	14717784	-23
chr2	1.8E+08	1.8E+08	Psma7	1.19	0.0001	-	chr2	1.8E+08	1.8E+08	-28
chr5	21309331	21309601	Psmc2	1.19	1.92E-07	+	chr5	21309547	21309577	59
chr14	45963332	45963455	Psmc6	1.19	0.011827	+	chr14	45963326	45963338	11
chr14	45968268	45968501	Psmc6	1.19	0.00086	+	chr14	45968378	45968394	37
chr14	14944788	14944908	Psmd6	1.19	0.008981	-	chr14	14944806	14944814	-149
chr4	59485209	59485376	Ptbp3	1.19	0.000633	-	chr4	59485272	59485296	-113
chr11	20124376	20124542	Rab1	1.19	0.013019	+	chr11	20124479	20124489	-15
chr7	1.08E+08	1.08E+08	Rab6a	1.19	0.002426	+	chr7	1.08E+08	1.08E+08	-116
chr15	51798138	51798320	Rad21	1.19	0.004149	-	chr15	51798250	51798251	-12
chr10	1.17E+08	1.17E+08	Rap1b	1.19	0.006754	-	chr10	1.17E+08	1.17E+08	-18
chr15	81305528	81305756	Rbx1	1.19	0.003166	+	chr15	81305551	81305582	47
chr5	1.16E+08	1.16E+08	Rnf10	1.19	0.005962	-	chr5	1.16E+08	1.16E+08	21
chr5	1.16E+08	1.16E+08	Rnf10	1.19	7.57E-05	-	chr5	1.16E+08	1.16E+08	56
chr12	16992125	16992385	Rock2	1.19	0.005541	+	chr12	16992360	16992371	-15

chr4	1.36E+08	1.36E+08	Rpl11	1.19	2.68E-08	-	chr4	1.36E+08	1.36E+08	26
chr9	1.2E+08	1.2E+08	Rpl14	1.19	0.000768	+	chr9	1.2E+08	1.2E+08	21
chr11	68717857	68718120	Rpl26	1.19	1.53E-07	+	chr11	68717951	68717970	-30
chr9	64023879	64024114	Rpl4	1.19	2.6E-06	+	chr9	64023899	64023921	-185
chr9	64025082	64025363	Rpl4	1.19	2.48E-17	+	chr9	64025212	64025242	-205
chr10	1.28E+08	1.28E+08	Rpl41	1.19	1.28E-07	-	chr10	1.28E+08	1.28E+08	24
chr15	76736559	76736825	Rpl8	1.19	4.58E-18	+	chr15	76736689	76736711	-26
chr5	1.16E+08	1.16E+08	Rplp0	1.19	1.22E-18	+	chr5	1.16E+08	1.16E+08	-71
chr10	23506487	23506756	Rps12	1.19	1.93E-05	-	chr10	23506632	23506662	136
chr17	24858502	24858738	Rps2	1.19	7.33E-06	+	chr17	24858653	24858658	-13
chr13	91064126	91064387	Rps23	1.19	1.53E-07	+	chr13	91064276	91064305	-330
chr13	91063119	91063392	Rps23	1.19	4.08E-08	+	chr13	91063193	91063259	-264
chr14	25315006	25315167	Rps24	1.19	1.77E-05	+	chr14	25315005	25315050	71
chr9	66797142	66797371	Rps27l	1.19	0.001426	+	chr9	66797184	66797292	-219
chr7	1.07E+08	1.07E+08	Rps3	1.19	1.89E-05	-	chr7	1.07E+08	1.07E+08	26
chr7	1.07E+08	1.07E+08	Rps3	1.19	1.58E-33	-	chr7	1.07E+08	1.07E+08	-34
chr7	1.07E+08	1.07E+08	Rps3	1.19	6.74E-06	-	chr7	1.07E+08	1.07E+08	56
chr7	1.07E+08	1.07E+08	Rps3	1.19	0.000106	-	chr7	1.07E+08	1.07E+08	90
chrX	99380293	99380520	Rps4x	1.19	3.22E-12	-	chrX	99380334	99380347	-47
chr7	16953866	16954059	Sae1	1.19	0.001108	-	chr7	16953860	16953890	35
chr4	1.41E+08	1.41E+08	Sdhb	1.19	0.000458	+	chr4	1.41E+08	1.41E+08	-72
chr7	88072622	88072787	Sec11a	1.19	0.002394	-	chr7	88072663	88072694	-236
chr7	88060526	88060801	Sec11a	1.19	0.000952	-	chr7	88060568	88060614	-126
chr6	67221888	67222089	Serbp1	1.19	0.002509	+	chr6	67221993	67222012	-19
chr2	29927955	29928128	Set	1.19	0.000158	+	chr2	29928012	29928051	288
chr6	6508352	6508526	Shfm1	1.19	6.79E-06	-	chr6	6508401	6508415	-16
chr4	1.19E+08	1.19E+08	Slc2a1	1.19	0.001278	+	chr4	1.19E+08	1.19E+08	-396
chr8	83224836	83225021	Smarca5	1.19	2.44E-06	-	chr8	83224933	83224945	30
chr3	68833325	68833506	Smc4	1.19	0.000201	+	chr3	68833340	68833341	40
chr3	68838302	68838523	Smc4	1.19	1.15E-09	+	chr3	68838499	68838524	68
chr2	1.43E+08	1.43E+08	Snrpb2	1.19	5.16E-06	+	chr2	1.43E+08	1.43E+08	32
chr1	1.36E+08	1.36E+08	Snrpe	1.19	0.001824	-	chr1	1.36E+08	1.36E+08	121
chr6	86326462	86326650	Snrpg	1.19	3.81E-07	+	chr6	86326560	86326573	-25
chr10	42252971	42253104	Snx3	1.19	7.35E-05	+	chr10	42252985	42253004	-92
chr16	90226369	90226630	Sod1	1.19	0.000138	+	chr16	90226490	90226554	819
chr11	29999688	29999791	Sptbn1	1.19	1.26E-05	-	chr11	29999721	29999806	58
chr12	55747032	55747241	Sptssa	1.19	0.002772	-	chr12	55747161	55747192	-43
chr1	1.84E+08	1.84E+08	Srp9	1.19	8.38E-07	+	chr1	1.84E+08	1.84E+08	482
chr17	23952611	23952785	Srrm2	1.19	0.010868	+	chr17	23952751	23952779	67
chr5	1.16E+08	1.16E+08	Srsf9	1.19	0.001009	+	chr5	1.16E+08	1.16E+08	-191
chr2	69709192	69709463	Ssb	1.19	2.15E-06	+	chr2	69709245	69709254	12
chr6	1.38E+08	1.38E+08	Strap	1.19	5.44E-06	+	chr6	1.38E+08	1.38E+08	91
chr6	1.38E+08	1.38E+08	Strap	1.19	0.005652	+	chr6	1.38E+08	1.38E+08	37
chr12	52710253	52710528	Strn3	1.19	1.01E-09	-	chr12	52710508	52710532	-82
chr15	11916157	11916358	Sub1	1.19	0.000312	-	chr15	11916269	11916310	34
chr5	1.4E+08	1.4E+08	Sun1	1.19	0.000884	+	chr5	1.4E+08	1.4E+08	93
chr4	1.41E+08	1.41E+08	Szrd1	1.19	0.000379	-	chr4	1.41E+08	1.41E+08	80
chr11	83319960	83320131	Taf15	1.19	0.001291	+	chr11	83320114	83320144	82
chr13	95612613	95612876	Tbca	1.19	1.75E-13	+	chr13	95612732	95612752	-43
chr17	13113126	13113392	Tcp1	1.19	0.003507	+	chr17	13113172	13113195	-43
chr16	56690688	56690809	Tfg	1.19	7.68E-05	-	chr16	56690792	56690824	60
chr14	14781620	14781856	Thoc7	1.19	0.000742	-	chr14	14781648	14781661	-31
chr19	41322406	41322556	Tm9sf3	1.19	6.17E-05	-	chr19	41322468	41322484	-18
chr19	41321141	41321331	Tm9sf3	1.19	0.005753	-	chr19	41321195	41321203	-15
chr5	1.09E+08	1.09E+08	Tmed5	1.19	2.53E-08	-	chr5	1.09E+08	1.09E+08	-48
chr5	1.09E+08	1.09E+08	Tmed5	1.19	2.14E-05	-	chr5	1.09E+08	1.09E+08	-89
chr12	71568231	71568414	Tmx1	1.19	0.000489	+	chr12	71568305	71568326	107
chr17	47824120	47824289	Tomm6	1.19	0.014231	-	chr17	47824139	47824156	-7
chr17	47823736	47824001	Tomm6	1.19	3.83E-10	-	chr17	47823735	47823866	867
chr5	23345483	23345645	Tomm7	1.19	0.004291	-	chr5	23345540	23345548	-58
chr11	98864162	98864416	Top2a	1.19	0.000245	-	chr11	98864211	98864250	-84
chr9	66878755	66878995	Tpm1	1.19	6.53E-07	-	chr9	66878810	66878818	13
chr1	1.52E+08	1.52E+08	Tpr	1.19	0.008674	+	chr1	1.52E+08	1.52E+08	-22
chr1	1.46E+08	1.46E+08	Trove2	1.19	0.00496	-	chr1	1.46E+08	1.46E+08	124
chr9	55995204	55995393	Tspan3	1.19	0.007298	-	chr9	55995250	55995292	-49
chr17	35973109	35973305	Tubb5	1.19	7.53E-20	-	chr17	35973128	35973130	13
chr4	57956648	57956926	Txn1	1.19	6.22E-34	-	chr4	57956697	57956740	-77
chr18	63823750	63824021	Txnl1	1.19	8.73E-08	-	chr18	63823786	63823801	-22
chr10	82347240	82347349	Txnrd1	1.19	0.001513	+	chr10	82347255	82347277	36
chr11	51804884	51805015	Ube2b	1.19	0.000319	-	chr11	51804951	51804980	68
chr5	65986122	65986372	Ube2k	1.19	0.000728	+	chr5	65986144	65986160	63
chr13	58278149	58278257	Ubqln1	1.19	1.38E-06	-	chr13	58278255	58278266	-20
chr13	58278800	58278955	Ubqln1	1.19	0.000608	-	chr13	58278877	58278935	-258
chr11	4601991	4602230	Uqcr10	1.19	3.35E-07	-	chr11	4602005	4602030	37
chr11	53242466	53242672	Uqcrq	1.19	1.5E-07	-	chr11	53242627	53242646	55
chr1	1.73E+08	1.73E+08	Usp21	1.19	5.61E-05	-	chr1	1.73E+08	1.73E+08	334
chr2	1.74E+08	1.74E+08	Vapb	1.19	0.005133	+	chr2	1.74E+08	1.74E+08	70
chr4	42992933	42993095	Vcp	1.19	6.59E-07	-	chr4	42992933	42992987	-135
chr8	87802868	87803002	Vps35	1.19	0.000213	-	chr8	87802932	87802953	-31
chr18	66142034	66142187	Lman1	1.184928	0.007673	-	chr18	66142102	66142123	-59
chr7	1.16E+08	1.16E+08	Eif3f	1.18129	3.55E-05	+	chr7	1.16E+08	1.16E+08	3
chr3	30718352	30718634	Sec62	1.173882	0.003388	+	chr3	30718314	30718402	86
chr14	27472597	27472723	Arf4	1.167583	1.99E-07	+	chr14	27472594	27472615	-71
chr9	75237980	75238212	Mapk6	1.127012	4.75E-05	-	chr9	75238150	75238152	11
chr17	28543037	28543221	Fkbp5	1.122806	0.003198	-	chr17	28543163	28543181	-12
chr6	54988015	54988220	Gars	1.116685	3.74E-07	+	chr6	54988056	54988057	2
chr9	46089307	46089407	Zfp259	1.114142	0.009427	+	chr9	46089297	46089320	-32
chr15	82180610	82180824	Ndufa6	1.11262	0.001782	-	chr15	82180632	82180653	-27
chr11	50108098	50108365	Canx	1.091088	0.000172	-	chr11	50108361	50108421	368
chr5	54046819	54047064	Rbpj	1.082434	0.00084	+	chr5	54046901	54046916	52
chr11	20124377	20124542	Rab1	1.077476	1.32E-05	+	chr11	20124479	20124489	-15
chr9	55983735	55984019	Tspan3	1.073066	4.38E-08	-	chr9	55983713	55983909	-869
chr8	1.13E+08	1.13E+08	Sf3b3	1.056254	0.00107	-	chr8	1.13E+08	1.13E+08	187
chr9	64564553	64564802	Rab11a	1.040166	0.000032	-	chr9	64564610	64564611	136
chr16	32018920	32019051	Pak2	1.040119	0.000142	-	chr16	32018985	32019040	284
chr5	34974293	34974426	Add1	1.039087	0.007891	+	chr5	34974270	34974304	98
chr11	97639588	97639776	Rpl23	1.009567	0.001157	-	chr11	97639606	97639676	-295
chr9	1.21E+08	1.21E+08	Ctnnb1	0.992431	0.003965	+	chr9	1.21E+08	1.21E+08	8
chr15	36530595	36530835	Pabpc1	0.972408	0.003125	-	chr15	36530631	36530649	17
chr4	1.17E+08	1.17E+08	Rps8	0.97092	0.001291	-	chr4	1.17E+08	1.17E+08	420
chr17	35187175	35187475	Clic1	0.959972	8.58E-08	+	chr17	35187305	35187318	-14
chr19	6059359	6059585	Fau	0.942536	1.2E-06	+	chr19	6059458	6059488	-161
chr7	4746031	4746212	Rpl28	0.942297	0.008369	+	chr7	4746095	4746107	6
chr9	44448030	44448220	Ddx6	0.926514	0.00036	+	chr9	44448128	44448221	-222
chr7	88072632	88072785	Sec11a	0.923151	0.007834	-	chr7	88072663	88072694	-236
chr13	91064150	91064366	Rps23	0.891587	8.62E-06	+	chr13	91064276	91064305	-330
chr5	21309372	21309584	Psmc2	0.865769	0.000255	+	chr5	21309547	21309577	59
chr18	63823767	63824018	Txnl1	0.861294	1.71E-05	-	chr18	63823786	63823801	-22

chr10	86166213	86166412	Hsp90b1	0.857973	1.29E-11	-	chr10	86166247	86166346	301
chr15	31520581	31520860	Cct5	0.850011	0.000174	-	chr15	31520485	31520689	608
chr2	69709191	69709419	Ssb	0.848565	0.008018	+	chr2	69709245	69709254	12
chr3	1.04E+08	1.04E+08	Slc16a1	0.847043	0.000318	+	chr3	1.04E+08	1.04E+08	-81
chr13	91063201	91063386	Rps23	0.841678	4.04E-07	+	chr13	91063193	91063259	-264
chr15	51621549	51621716	Eif3h	0.829609	0.002319	-	chr15	51621572	51621603	-80
chr9	64022635	64022882	Rpl4	0.828073	1.49E-34	+	chr9	64022744	64022759	-40
chr13	99083052	99083302	Btf3	0.826986	1.55E-09	-	chr13	99083074	99083108	51
chr11	21457324	21457581	Mdh1	0.826884	1.25E-06	-	chr11	21457424	21457457	67
chr14	45968267	45968492	Psmc6	0.825989	5.85E-05	+	chr14	45968378	45968394	37
chr9	1.14E+08	1.14E+08	Pdcd6ip	0.821265	9.79E-05	-	chr9	1.14E+08	1.14E+08	61
chr4	42998033	42998147	Vcp	0.819131	0.002842	-	chr4	42998112	42998124	-6
chr12	1.13E+08	1.13E+08	2010107E(	0.818486	0.000626	-	chr12	1.13E+08	1.13E+08	-48
chr10	77890055	77890353	Cstb	0.81684	3.64E-11	+	chr10	77890279	77890342	-167
chr11	22899045	22899211	Cct4	0.809501	0.000151	+	chr11	22899100	22899132	-124
chr11	40564768	40564901	Ccng1	0.80505	0.000222	-	chr11	40564798	40564823	69
chr5	1.35E+08	1.35E+08	Eif4h	0.797565	3.71E-13	-	chr5	1.35E+08	1.35E+08	-26
chr15	51798135	51798306	Rad21	0.796715	0.000965	-	chr15	51798250	51798251	-12
chr7	1.07E+08	1.07E+08	Rps3	0.791957	0.003344	-	chr7	1.07E+08	1.07E+08	26
chr5	1.3E+08	1.3E+08	Cct6a	0.783177	0.001561	+	chr5	1.3E+08	1.3E+08	23
chr3	33967861	33968037	Fxr1	0.780522	0.001508	+	chr3	33967917	33967959	48
chr11	68717876	68718115	Rpl26	0.773267	7.38E-05	+	chr11	68717951	68717970	-30
chr6	6508303	6508526	Shfm1	0.763866	0.000687	-	chr6	6508401	6508415	-16
chr4	1.16E+08	1.16E+08	Uqcrh	0.749811	4.93E-05	-	chr4	1.16E+08	1.16E+08	591
chr15	51618113	51618405	Eif3h	0.74674	5.39E-17	-	chr15	51618145	51618153	-72
chr13	95612614	95612792	Tbca	0.741425	1.87E-06	+	chr13	95612732	95612752	-43
chr10	62043306	62043496	Ddx21	0.736286	0.000181	-	chr10	62043474	62043498	134
chr15	98568970	98569249	Arf3	0.732573	0.011699	-	chr15	98569101	98569103	-19
chr9	64023908	64024112	Rpl4	0.731155	5.73E-05	+	chr9	64023899	64023921	-185
chr17	45707622	45707828	Hsp90ab1	0.722937	9.5E-16	-	chr17	45707630	45707654	44
chr7	1.21E+08	1.21E+08	Psma1	0.719149	7.92E-07	-	chr7	1.21E+08	1.21E+08	-84
chr4	1.38E+08	1.38E+08	Ddost	0.71758	0.003633	+	chr4	1.38E+08	1.38E+08	-31
chr12	17277206	17277385	Pdia6	0.714036	1.3E-06	+	chr12	17277302	17277315	-59
chr16	36044015	36044200	Fam162a	0.712787	0.00547	-	chr16	36044036	36044058	22
chr15	43097698	43097837	Eif3e	0.698663	0.007855	-	chr15	43097788	43097804	-83
chr9	64025135	64025346	Rpl4	0.694864	9.72E-11	+	chr9	64025212	64025242	-205
chr6	17606245	17606469	Capza2	0.68896	0.00942	+	chr6	17606355	17606375	23
chr2	1.21E+08	1.21E+08	Pdia3	0.686409	4.69E-05	+	chr2	1.21E+08	1.21E+08	27
chr10	1.28E+08	1.28E+08	Atp5b	0.66881	8.74E-15	+	chr10	1.28E+08	1.28E+08	-38
chr7	1.18E+08	1.18E+08	Eif4g2	0.66281	0.000561	-	chr7	1.18E+08	1.18E+08	-460
chr9	1.2E+08	1.2E+08	Rpl14	0.662674	0.000213	+	chr9	1.2E+08	1.2E+08	21
chr17	13113149	13113376	Tcp1	0.662214	0.006782	+	chr17	13113172	13113195	-43
chr11	52059853	52060065	Skp1a	0.654314	0.003719	+	chr11	52059969	52059978	-43
chr1	1.55E+08	1.55E+08	Arpc5	0.653016	6.29E-06	+	chr1	1.55E+08	1.55E+08	-39
chr15	78924647	78924847	Eif3l	0.652994	2.57E-05	+	chr15	78924694	78924708	26
chr17	24858577	24858725	Rps2	0.652336	0.000032	+	chr17	24858653	24858658	-13
chr10	1.28E+08	1.28E+08	Myl6	0.652081	4.98E-05	-	chr10	1.28E+08	1.28E+08	253
chr10	1.27E+08	1.27E+08	Naca	0.651329	0.000945	+	chr10	1.27E+08	1.27E+08	-6
chr13	58494567	58494757	Hnrnpk	0.645002	0.00473	-	chr13	58494567	58494575	28
chr10	1.27E+08	1.27E+08	Naca	0.64361	0.010606	+	chr10	1.27E+08	1.27E+08	-77
chr1	1.27E+08	1.27E+08	Actr3	0.629713	0.011493	-	chr1	1.27E+08	1.27E+08	61
chr5	1.16E+08	1.16E+08	Rplp0	0.625427	2.16E-09	+	chr5	1.16E+08	1.16E+08	-71
chr2	1.55E+08	1.55E+08	Raly	0.625378	1.24E-07	+	chr2	1.55E+08	1.55E+08	10
chr16	87487778	87487998	Cct8	0.62356	0.001877	-	chr16	87487789	87487801	-29
chr16	92111984	92112278	Mrps6	0.623409	2.22E-05	+	chr16	92112176	92112197	20
chr4	1.36E+08	1.36E+08	Rpl11	0.619242	0.000457	-	chr4	1.36E+08	1.36E+08	26
chr1	1.8E+08	1.8E+08	Hnrnpu	0.605951	0.014746	-	chr1	1.8E+08	1.8E+08	-49
chr1	55147946	55148089	Hspe1	0.600327	0.000207	+	chr1	55147917	55147988	164
chr1	88247395	88247669	Ncl	0.593922	0.000212	-	chr1	88247592	88247598	-5
chr5	1.44E+08	1.44E+08	Fscn1	0.587646	2.69E-05	+	chr5	1.44E+08	1.44E+08	230
chr2	73746516	73746809	Atp5g3	0.585565	2.25E-13	-	chr2	73746556	73746568	-155
chr6	1.25E+08	1.25E+08	Gapdh	0.584524	0.003953	-	chr6	1.25E+08	1.25E+08	322
chr5	1.49E+08	1.49E+08	Pomp	0.58326	1.6E-08	+	chr5	1.49E+08	1.49E+08	27
chr6	11856025	11856152	Ndufa4	0.565089	0.000866	-	chr6	11856085	11856094	-13
chr5	31924794	31924977	Mrpl33	0.562004	4.49E-05	+	chr5	31924853	31924877	47
chr10	80291630	80291903	Oaz1	0.560729	0.00019	+	chr10	80291732	80291745	35
chr2	1.21E+08	1.21E+08	Serf2	0.558423	0.001699	+	chr2	1.21E+08	1.21E+08	324
chr8	1.26E+08	1.26E+08	Chmp1a	0.55077	1.72E-11	-	chr8	1.26E+08	1.26E+08	-3
chr9	66797141	66797323	Rps27l	0.550581	0.002805	+	chr9	66797184	66797292	-219
chr6	11850381	11850612	Ndufa4	0.534801	0.000845	-	chr6	11850486	11850536	-102
chr4	57956650	57956900	Txn1	0.529606	1.1E-14	-	chr4	57956697	57956740	-77
chr4	1.5E+08	1.5E+08	Rere	0.521051	0.001987	+	chr4	1.5E+08	1.5E+08	-3
chr17	33802452	33802598	Hnrnpm	0.50281	8.93E-05	-	chr17	33802542	33802567	36
chr5	1.3E+08	1.3E+08	Chchd2	0.501283	6.6E-07	-	chr5	1.3E+08	1.3E+08	-241
chr11	50199292	50199589	Hnrnph1	0.487374	0.005045	+	chr11	50199528	50199545	-110
chr17	35973111	35973283	Tubb5	0.466489	0.011736	-	chr17	35973128	35973130	13
chr9	1.06E+08	1.06E+08	Rpl29	0.450762	3.07E-05	+	chr9	1.06E+08	1.06E+08	-13
chr15	98868274	98868518	Tuba1c	0.448717	0.000214	+	chr15	98868469	98868506	135
chr6	51419686	51419888	Hnrnpa2b1	0.44576	0.00011	-	chr6	51419871	51419875	-4
chr11	6319124	6319297	Ppia	0.383252	0.001716	+	chr11	6319167	6319195	31
chr10	90579634	90579886	Slc25a3	0.369311	0.003442	-	chr10	90579709	90579778	109
chr15	98761899	98762192	Tuba1b	0.336846	2.01E-06	-	chr15	98761885	98761948	-354
